# Protective Effect of Bojungikki-Tang against Radiation-Induced Intestinal Injury in Mice: Experimental Verification and Compound-Target Prediction

**DOI:** 10.1155/2023/5417813

**Published:** 2023-01-04

**Authors:** Sohi Kang, A. Y. Lee, Hyun H. Nam, Soong-In Lee, Hyun-Yong Kim, Jeong M. Lee, Changjong Moon, In S. Shin, Sung-Wook Chae, Ji H. Lee, Yun-Soo Seo, Joong S. Kim

**Affiliations:** ^1^College of Veterinary Medicine and BK21 Plus Project Team, Chonnam National University, Gwangju, Republic of Korea; ^2^Herbal Medicine Resources Research Center, Korea Institute of Oriental Medicine, 111 Geonjae-ro, Naju-si, Republic of Korea; ^3^College of Oriental Medicine, Dongshin University, Naju-si, Jeollanam-do, Republic of Korea; ^4^Division of Radiation Effects, Korea Institute of Radiological and Medical Sciences, Seoul, Republic of Korea; ^5^Center for Companion Animal New Drug Development, Jeonbuk Branch Korea Institute of Toxicology (KIT), Jeongeup, Jeollabuk-do, Republic of Korea; ^6^College of Korean Medicine, Semyung University, Jecheon-si, Chungcheongbuk-do, Republic of Korea

## Abstract

Bojungikki-tang (BJIT) is a traditional herbal medicine used in Korea, Japan, and China to treat gastrointestinal disorders. In this study, we aimed to investigate whether BJIT has protective effects against radiation-induced intestinal injury and to predict the underlying therapeutic mechanisms and related pathways via network pharmacological analyses. BJIT was injected intraperitoneally (50 mg/kg body weight) to C_3_H/HeN mice at 36 and 12 h before exposure to partial abdominal irradiation (5 Gy and 13 Gy) to evaluate the apoptotic changes and the histological changes and variations in inflammatory cytokine mRNA levels in the jejunum, respectively. Through in silico network analysis, we predicted the mechanisms underlying BJIT-mediated regulation of radiation-induced intestinal injury. BJIT reduced the level of apoptosis in the jejunal crypts 12 h post 5-Gy irradiation. Histological assessment revealed intestinal morphological changes in irradiated mice 3.5 days post 13-Gy irradiation. Furthermore, BJIT decreased inflammatory cytokine levels following radiation exposure. Apoptosis, TNF, p53, VEGF, toll-like receptor, PPAR, PI3K-Akt, and MAPK signaling pathways, as well as inflammatory bowel disease (IBD), were found to be linked to the radioprotective effects of BJIT against intestinal injury. According to our results, BJIT exerted its potential protective effects by attenuating histopathological changes in jejunal crypts and suppressing inflammatory mediator levels. Therefore, BJIT is a potential therapeutic agent that can treat radiation-induced intestinal injury and its associated symptoms.

## 1. Introduction

Radiation therapy is crucial for the treatment of pelvic and abdominal malignancies, including carcinomas of the pancreas, cervix, ovary, prostate, uterus, and rectum [1, 2]. Radiation therapy improves the prognoses of patients with malignancies since it directly eliminates the diagnosed malignancies or palliates symptoms associated with advanced, recurrent disease [1]. However, this therapy can lead to various complications, including radiation-induced intestinal injury [1–3]. Intestinal injury, a common complication with an incidence rate of 50–70% [4, 5], leads to weight loss, diarrhea, intestinal strictures/fistulas, and even severe enteric septicemia, all of which can seriously affect the patient's quality of life [5, 6].

Several effective compounds targeting radiation-induced intestinal injuries have been identified [7–10]. Recently, natural products, especially herbal prescriptions in traditional medicine, have been assessed as potential radioprotective agents owing to their efficacy and low toxicity [11, 12]. According to the theory of traditional medicine, ionizing radiation, which induces acute radiation injury (ARI), belongs to the “heat toxin” category. Heat toxin burns off Qi and Yin, which are integral substances of the human body and essential for the physiological activity of all organ systems [13]. In this respect, “clearing heat and removing toxin” and “tonifying Qi and nourishing Yin” are usually used together as the main principles for effective treatment [14].

Bojungikki-tang (BJIT; Hochuekkito in Japanese, Bu-zhong-yi-qi-tang in Chinese) is a major traditional prescription in East Asian countries, which comprises eight herbal components: *Angelica gigas* Nakai, *Astragalus membranaceus* Bunge, *Atractylodes japonica* Koidz, *Bupleurum falcatum* Linné, *Cimicifuga heracleifolia* Komarov, *Citrus unshiu* Markovich, *Panax ginseng* C. A. Meyer, and *Glycyrrhiza uralensis* Fischer [15]. In traditional herbal medicine, BJIT refers to a decoction that “tonifies the middle” and augments Qi [16]; therefore, it is clinically used for the systematic treatment of complex gastrointestinal diseases [17]. Recent studies have demonstrated the favorable effects of BJIT on alcohol-induced gastric injury [18], gastric mucosal damage [19], and *Helicobacter pylori* infection [20].

Additionally, BJIT can be used for the treatment of radiotherapy-induced injuries. Many recent studies have indicated that the main herbal components in BJIT decoction have an antiradiation effect [11, 20, 21]. Moreover, a previous study that assessed intestinal crypt survival and apoptosis confirmed that BJIT treatment attenuates radiation-induced intestinal injury [11], indicating that BJIT might be a useful radioprotective agent. However, detailed mechanistic features remain unelucidated.

The identification of complex molecular mechanisms is a major challenge with herbal formulas. Traditional herbal medicines are composed of multiple compounds, rendering the underlying mechanisms more complex than those of a single active compound [17]. However, the conventional experimental approach to determine mechanisms is time-consuming, laborious, and expensive. Moreover, elucidating specific interactions between compounds and their respective targets is difficult with the conventional approach [21]. Therefore, new methods and strategies are urgently needed to address this problem. Network pharmacology [22], a new strategy, can independently identify compound-target pathways related to a particular disease while providing a systematic and holistic view [23].

In this study, we examined the protective effect of BJIT against intestinal injury in a murine model exposed to radiation. Then, to holistically evaluate the regulatory mechanisms of BJIT, we performed a pharmacological network analysis of BJIT to predict the potential active compounds and radiation-induced intestinal injury-related target genes via *in silico* network analysis.

## 2. Materials and Methods

### 2.1. Materials

Eight herbal medicines found in BJIT, namely, the roots of *Angelica gigas* Nakai, *Astragalus membranaceus* Bunge, *Atractylodes japonica* Koidz, *Bupleurum falcatum* Linné, *Cimicifuga heracleifolia* Komarov, *Panax ginseng* C. A. Meyer, and *Glycyrrhiza uralensis* Fischer and peel of *Citrus unshiu* Markovich, were purchased from Kwangmyungdang Medicinal Herbs (Ulsan, Korea) and then extracted in the Shin-Nong-Bon-Cho herbal pharmacy. The decoction was lyophilized to make a powder with a yield of 26.05%.

### 2.2. Animal Experiments

#### 2.2.1. Animal Maintenance

Pathogen-free male C_3_H/HeN mice (eight-week-old) were purchased from Central Lab Animal Inc. (Seoul, Korea). Animals were maintained in a room at 23 ± 2°C with a relative humidity of 50 ± 5%, artificial lighting from 08:00–20:00, and 13–18 air changes per hour. Mice were fed a standard animal diet. Experiments were performed one week after quarantine and acclimatization. All the animal procedures were approved by the Institutional Animal Care and Use Committee of the Korea Institute of Oriental Medicine (KIOM 20-032) and were performed in compliance with the National Institutes of Health Guidelines for the care and use of laboratory animals and the Korean national laws for animal welfare.

#### 2.2.2. Preparation of Sample Solutions

A decoction of BJIT was prepared in our laboratory from a mixture of chopped crude herbs (37.5 g of *A. gigas*, 112.5 g of *A. membranaceus*, 75 g of *A. japonica*, 22.5 g of *B. falcatum*, 22.5 g of *C. heracleifolia*, 37.5 g of *C. unshiu*, 75 g of *P. ginseng*, and 75 g of *G. uralensis*), which were extracted in 5 L of distilled water at 100°C for 2 h. The solution was evaporated to dryness and freeze-dried (extract: 119.2 g; yield: 26.05%). The lyophilized BJIT extract was dissolved in distilled water and mixed.

#### 2.2.3. HPLC Pattern Analysis

**Figure 1:**
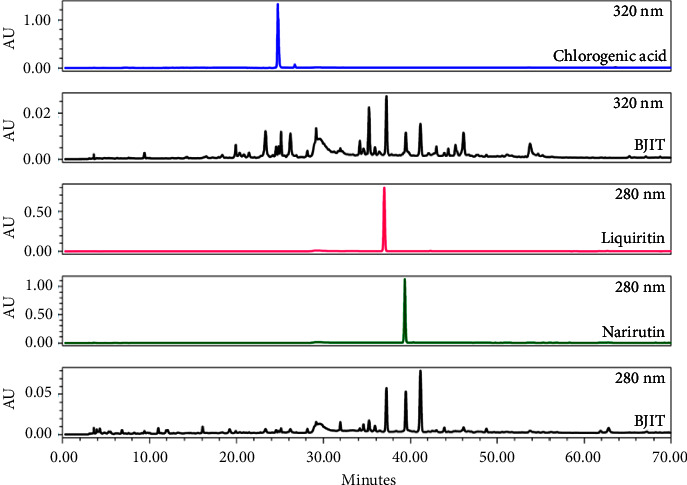
Chromatogram of BJIT (black), chlorogenic acid (blue, 24.7 min) at 320 nm, liquiritin (pink, 37.0 min), and narirutin (green, 39.4 min) at 280 nm.

The lyophilized BJIT extract (100.1 mg) was dissolved in 10 mL of distilled water and filtered through a GHP 0.2 *μ*m syringe filter (0.2 mm pore size, Woongki Science, Seoul, Republic of Korea). The HPLC system (Waters, Milford, MA) consisted of the Separations Module (Waters e2695) and the 2998 PDA detector (Waters). Chlorogenic acid (≥98%, ChemFaces, Wuhan ChemFaces Biochemical Co., Ltd., China), liquiritin (≥98%, ChemFaces, Wuhan ChemFaces Biochemical Co., Ltd., China), and narirutin (≥98%, ChemFaces, Wuhan ChemFaces Biochemical Co., Ltd., China) were dissolved in methanol and separated using the XSelectTM HSS T3 column (5 *μ*m, 4.6 × 250 mm, Waters). The mobile phase was prepared by mixing 0.05% aqueous formic acid (A), methanol (B), and acetonitrile (C) via the following linear gradient program: 100% A—95% A (3% B, 2% C) for 0–8 min, 95% A (3% B, 2% C)—80% A (12% B, 8% C) for 8–20 min, 80% A (12% B, 8% C)—50% A (30% B, 20% C) for 20–50 min, 50% A (30% B, 20% C)—45% A (33% B, 22% C) for 50–60 min, and 45% A (33% B, 22% C)—0% A (60% B, 40% C) for 60–80 min. The flow rate was 0.75 mL/min, the injected volume was 10 *μ*L, and the column was at room temperature. The UV wavelength was monitored from 210 to 400 nm; chlorogenic acid was detected at 320 nm, whereas liquiritin and narirutin were measured at 280 nm. Retention times of chlorogenic acid, liquiritin, and narirutin were 24.7, 37.0, and 39.4 min, respectively (Figure 1).

#### 2.2.4. Irradiation Exposure and Experimental Groups

**Figure 2:**
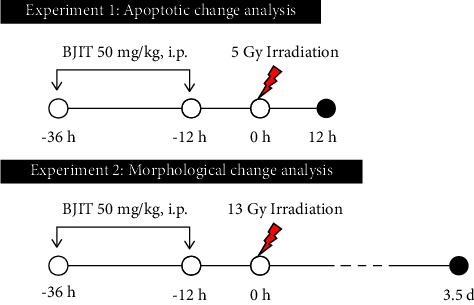
Schematic diagram of the experimental procedure. In experiment 1, the mice were pretreated with intraperitoneal injections of vehicle or BJIT decoction at 36 h and 12 h before irradiation. Then, the mice received partial abdominal irradiation at 0 (Sham) or 5 Gy and were euthanized after 12 h In experiment 2, the mice were pretreated with intraperitoneal injections of vehicle or BJIT decoction at 36 and 12 h before radiation exposure. Then, the mice were irradiated with 0 or 13 Gy and were euthanized for tissue sampling at 3.5 days postirradiation. Black circles indicate the times of tissue collection from the sham-irradiated (0 Gy) controls and irradiated (5 or 13 Gy) test animals.

Each mouse was anesthetized with 85 mg/kg of alfaxalone (Alfaxan®; Careside, Republic of Korea) and 10 mg/kg of xylazine (Rompun®, Bayer Korea, Republic of Korea) and restrained on a tray. Mice were exposed to abdominal radiation using 6 MV high-energy photon rays (ELEKTA, Stockholm, Sweden) at a dose of 3.8 Gy/min. Abdominal irradiation at doses of 5 Gy and 13 Gy was used to evaluate apoptotic changes (Experiment 1) and histological changes (Experiment 2) in the jejunum, respectively. Sham-irradiated mice were treated the same way as radiated animals but without radiation. The experimental timeline is summarized in Figure 2.

In the first set of experiments, to evaluate the effect of BJIT on apoptotic changes, mice were divided into four groups that received the following treatment regimens: (Exp. 1—Group 1) sham-irradiated control (sham) group; (Exp. 1—Group 2) BJIT 50 mg/kg-treated (BJIT) group; (Exp. 1—Group 3) 5 Gy-irradiated (5 Gy); and (Exp. 1—Group 4) BJIT 50 mg/kg-treated and 5 Gy-irradiated (BJIT + 5 Gy) group. Intestinal samples were collected 12 h postirradiation. The euthanasia time point was chosen as 12 h after radiation because the maximum number of apoptotic cells was observed at this time point in previous studies [9, 24].

In the second set of experiments, to evaluate the effect of BJIT on histological changes, the mice were divided into four groups receiving the following treatment regimens: (Exp. 2-Group 1) sham-irradiated control (sham) group; (Exp. 2-Group 2) BJIT 50 mg/kg-treated (BJIT) group; (Exp. 2-Group 3) 13 Gy-irradiated (13 Gy) group; and (Exp. 2-Group 4) BJIT 50 mg/kg-treated and 13 Gy-irradiated (BJIT + 13 Gy) group. The mice were euthanized 3.5 days after radiation exposure.

#### 2.2.5. Apoptotic Changes in the Jejunal Crypts

Small intestine tissues were preserved in 10% neutral buffered formaldehyde and paraffin-embedded. Using a terminal deoxynucleotidyl transferase-mediateddUTP-biotin Nick end labeling (TUNEL, ApopTag Plus™ Kit, InterGen, USA) approach, four micrometer-thick slices of the tissue were then labeled. Under a light microscopy (Nikon Eclipse 80i, Nikon Corporation), apoptotic cells in longitudinal crypt sections displaying a substantial proportion the crypt lumen, base, and at least 17 cells along the crypt column were counted. When many apoptotic pieces were thought to be the remains of a single cell, single cells were recorded based on the size and grouping. For each mouse, 30 crypts were examined [9, 24].

#### 2.2.6. Histological Changes in the Jejunal Crypts

For histological analysis, two slices from each mouce's jejunum were sectioned from four different spots. Hematoxylin and eosin (H&E) was used to stain intestinal slices in order to examine the morphology. Then the jejunal cross-sections were counted for the regenerating crypts and villi. All samples were sectioned and reoriented in successive slices to determine which ones had the longest villi in order to analyze the morphological changes. This method was chosen because it produced data that were more consistent than that produced by normal methods, which only measured the ten longest villi in a single slice per sample [8, 9]. The lengths of the ten longest villi and the heights of the basal lamina of ten small intestinal sections from each animal were measured. Using a polyclonal rabbit anti-KI-67 antibody (Acris Antibodies GmbH, Hiddenhausen, Germany; diluted 1 : 500), the proliferation was examined using immunohistochemistry to measure cell proliferation in jejunum samples. Avidin-biotin peroxidase (Elite Kit, Vector Laboratories, Burlingame, CA, USA) was used to identify the attached antibodies, and a diaminobenzidine substrate kit (Vector) was used to assess the peroxidase activity. In each experiment, the primary antibody was left out of a few test sections as a negative control. A digital camera mounted on a microscope was used to capture images of intestinal sections (Leica DM IRBE, Leica MicroSystems GmbH, Wetzlar, Germany). Utilizing software for image processing, quantification was performed (Leica QWin, Leica Microsystems, Wetzlar, Germany).

#### 2.2.7. Determination of mRNA Levels by Quantitative Reverse Transcription-Polymerase Chain Reaction (qRT-PCR)

Total RNA was extracted from small intestine tissues using an ISOGEN kit (Nippon Gene, Tokyo, Japan). Real-timeqRT-PCR analyses were performed as previously described [25]. The expression levels of mRNAs for *Bax*, *Bcl*2, *iNos* (*Nos*2), and *Cox-*2 (*Ptgs*2) were quantified, normalized to the expression level of *β-actin (Actb)* mRNA, and expressed relative to the corresponding mean value for the small intestine tissue of sham-irradiated control mice. The sequences of the PCR primers and TaqMan probes are listed in Table 1.

#### 2.2.8. Active Component Screening of BJIT from Public Databases

To understand the holistic characteristics of BJIT, an *in silico* integrative absorption, distribution, metabolism, and excretion (ADME) model was used, relying on the Traditional Chinese Medicine System Pharmacology Database and Analysis Platform (TCMSP; https://lsp.nwu.edu.cn/, version 2.3, accessed on August 6, 2020) and the Korean and Chinese Pharmacopoeia. The chemical name, synonyms, molecular weight, physicochemical properties, 2D structure, and 3D structure for each component were verified using PubChem (https://pubchem.ncbi.nlm.nih.gov/, accessed on August 10, 2020), ChemSpider (https://www.chemspider.com/, version 2020.0.18.0), and ChEMBL (https://www.ebi.ac.uk/chembl/, updated on May 18, 2020).

#### 2.2.9. Pharmacokinetic ADME Evaluation

Since herbal formulas often comprise several active compounds, potential active compounds were selected through ADME screening [23]. The ADME system used can predict oral bioavailability (OB) and drug-likeness (DL). Compounds were retained only if OB ≥ 30% and DL ≥ 0.18 to satisfy the criteria suggested by the TCMSP [14, 15].

#### 2.2.10. Target Genes Related to Active Compounds

Target genes associated with active compounds in BJIT were linked using the Search Tool for Interactions of Chemicals and Proteins (STITCH) database (https://stitch.embl.de/, ver. 5.0, accessed on August 24, 2020) with “*Homo sapiens*” selected as the organism [26]. This database provides a platform for exploring known interactions between small molecules, proteins, and organism-basedprotein-protein interactions [27]. Active compound-protein interactions with an interaction score ≥0.400 (medium confidence) were selected [28]. Gene information, including gene IDs and names, was verified in the UniProt database (https://www.uniprot.org/, accessed on September 15, 2020) by limiting to “*Homo sapiens*” as the species.

#### 2.2.11. Potential Target Genes and Protein-Protein Interaction

The public database GeneCards: The Human Gene Database (https://www.genecards.org/, version 5.0, accessed on October 14, 2020) was searched for information on potential target genes, limiting to those from “*Homo sapiens*.” The aforementioned target genes were matched to intestinal injury-related genes, and overlapping genes were consolidated as potential target genes. Using a high confidence score (≥0.700), a protein-protein interaction (PPI) network for “*Homo sapiens*” was created with the STITCH database (https://stitch.embl.de/, version 5.0) [29].

#### 2.2.12. Signaling Pathway Analysis

The compound-protein and PPI networks were visualized, and the topological parameters of the signaling pathway-protein network were analyzed using Cytoscape 3.7.2 (https://cytoscape.org) [29, 30]. After network analysis, functional annotation of genes was performed using the Database for Annotation, Visualization, and Integrated Discovery (DAVID; https://david.ncifcrf.gov/, version 6.8) system and the Kyoto Encyclopedia of Genes and Genomes (KEGG; https://www.genome.jp/kegg/, Release 96.0, October 1, 2020) database.

### 2.3. Statistical Analysis

The results are reported as the mean ± standard error of the mean (SEM) and were analyzed using one-way analysis of variance (ANOVA) followed by the Student–Newman–Keuls *post hoc* test for multiple comparisons. In all cases, a *p* value <0.05 was considered significant.

## 3. Results

### 3.1. Protective Effects of BJIT against Intestinal Apoptotic Changes in Irradiated Mice

**Figure 3:**
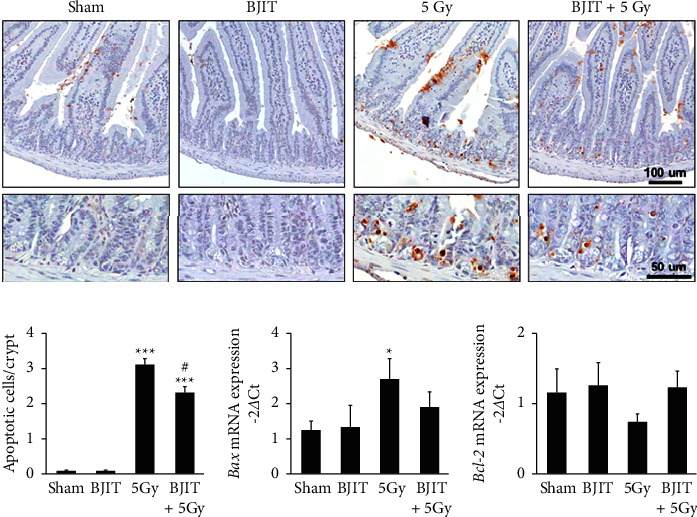
BJIT protects against radiation-induced intestinal apoptosis. (a) Representative photomicrographs of apoptotic changes within the crypts of the jejunal circumference in TUNEL-stained sections following vehicle or BJIT treatment at 12 h post-5 Gy irradiation. (b) Bar graphs showing the number of apoptotic cells per crypt in the jejunal sections as determined by the TUNEL method. Quantitative reverse transcription-PCR of mRNA encoding (c) *Bax* and (d) *Bcl-*2 in the small intestines of irradiated mice with the vehicle and BJIT treatment. Values are reported as mean ± SEM (*n* = 6 per group), ^*∗*^*p* < 0.05, ^*∗∗∗*^*p* < 0.01 vs. sham controls. ^#^*p* < 0.05 vs. 5 Gy-irradiated controls. BJIT, Bojungikki-tang.

In the jejunal crypts, apoptosis was easily recognized in apoptotic bodies by TUNEL staining of the tissue postradiation. Most apoptotic cells were observed in the putative stem cell zone located at the base of the jejunal crypts (Figure 3(a)). In the irradiated group, a marked increase in the number of apoptotic nuclei was observed at the base of the jejunal crypts (sham: 0.09 ± 0.02, 5 Gy: 3.10 ± 0.19, *p* < 0.001; Figure 3(b)). BJIT treatment significantly reduced this parameter (BJIT + 5 Gy: 2.31 ± 0.17, *p* < 0.05; Figure 3(b)).

We analyzed the expression levels of proapoptotic and antiapoptotic mRNAs, namely, *Bax* and *Bcl-*2, respectively, in the small intestine 12 h postirradiation. As shown in Figure 3(c), the irradiation-mediated apoptosis was characterized by a marked increase in the *Bax* mRNA level (sham: 1.22 ± 0.28, 5 Gy: 2.68 ± 0.59, *p* < 0.05). BJIT treatment did not significantly reduce this parameter (BJIT + 5 Gy: 1.88 ± 0.44, *p*=0.30; Figure 3(c)). As shown in Figure 3(d), the level of *Bcl-*2 mRNA remained unchanged in all groups, indicating that *Bcl-*2 mRNA was constitutively expressed in the jejunal tissue and was not significantly altered after 5-Gy irradiation (sham: 1.14 ± 0.35, 5 Gy: 0.72 ± 0.15, *p*=0.29). Although no statistically significant difference was detected, treatment of BJIT apparently increased the expression level of *Bcl-*2 mRNA postirradiation (BJIT + 5 Gy: 1.88 ± 0.44, *p*=0.10).

### 3.2. Protective Effects of BJIT against Intestinal Morphological Changes in Irradiated Mice

**Figure 4:**
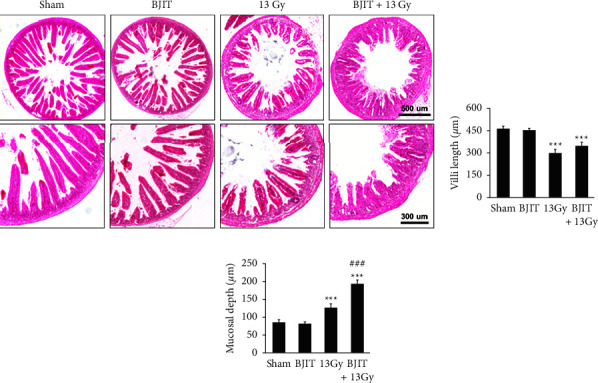
BJIT protects against radiation-induced intestinal histological changes. (a) Representative images of hematoxylin and eosin (H&E)-stained jejunal sections harvested from vehicle- or BJIT-treated mice at 3.5 days after 13-Gy abdominal irradiation. Bar graphs showing the (b) villi length and (c) mucosal depth of jejunum. Values are reported as mean ± SEM (*n* = 6 per group), ^*∗∗∗*^*p* < 0.001 vs. sham controls. ^###^*p* < 0.001 vs. 13 Gy-irradiated controls. BJIT, Bojungikki-tang.

As shown in Figure 4, the morphology of the jejunal mucosa of mice was altered 3.5 days postirradiation (13 Gy). The villus length of the jejunum after 13 Gy irradiation was significantly shorter than that in the sham-radiated control mice (sham: 464 ± 14.40 *μ*m, 13 Gy: 307 ± 18.03 *μ*m, *p* < 0.001), demonstrating the damaging effects of radiation on the jejunum. Although no statistically significant difference was detected, BJIT treatment seemingly attenuated the extent of reduction in villus length after irradiation (BJIT + 13 Gy: 347 ± 15.58 *μ*m, *p*=0.13; Figures 4(a) and 4(b)).

Compared to sham-radiated animals, in the 13 Gy-irradiated group, mucosal depth was significantly increased in the jejunum 3.5 days post-13 Gy irradiation (sham: 83 ± 6.03 *μ*m, 13 Gy: 128 ± 6.24 *μ*m, *p* < 0.001). BJIT treatment significantly increased this parameter (BJIT + 13 Gy: 195 ± 10.78 *μ*m, *p* < 0.001; Figures 4(a) and 4(c)).

**Figure 5:**
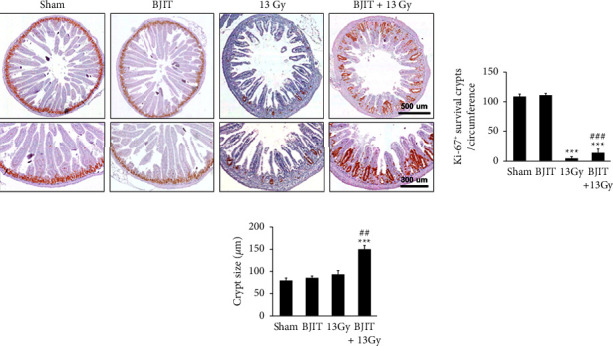
BJIT treatment rescues jejunal crypt survival after 13-Gy abdominal irradiation. (a) Representative photomicrographs of proliferative crypts in the jejunum circumference stained with an antibody for Ki-67. Bar graphs showing (b) the number of Ki-67-positive crypts per circumference and (c) the size of jejunal crypts in the sections, as determined by the Ki-67-positive stained cells. Values are reported as mean ± SEM (*n* = 6 per group), ^*∗∗∗*^*p* < 0.001 vs. sham controls. ^##^*p* < 0.01, ^###^*p* < 0.001 vs. 13 Gy-irradiated controls. BJIT, Bojungikki-tang.

Proliferative crypts cells were identified by immunohistochemical staining of Ki-67 (Figure 5(a)). The sham control group had a large number of Ki-67-positive crypts in the jejunum. Irradiated mice had a significantly decreased number of Ki-67-positive crypts 3.5 days postirradiation with 13 Gy (sham: 110 ± 5.92, 13 Gy: 6 ± 0.74, *p* < 0.001). Compared to the irradiated group, the BJIT-treated group had significantly more Ki-67-positive crypts 3.5 days postirradiation (BJIT + 13 Gy: 16 ± 1.35, *p* < 0.001; Figure 5(b)).

Although no statistically significant difference was observed, the crypt size of jejunal crypts after irradiation with 13 Gy seemingly increased compared to the sham-irradiated controls (sham: 80.86 ± 5.44 *μ*m, 13 Gy: 93.23 ± 9.84 *μ*m, *p*=0.30), demonstrating the injurious effects of radiation on the jejunum. Furthermore, treatment with BJIT significantly increased the crypt size postirradiation (BJIT + 13 Gy: 150.33 ± 11.20 *μ*m, *p* < 0.001). Although no statistically significant difference was detected, the crypt sizes were greater in mice that received partial-body irradiation, reflecting the intestinal response following injury (sham: 89.6 ± 5.8 vs. radiation: 107.2 ± 4.2; Figures 5(a) and 5(c)). The crypt sizes were significantly increased in the BJIT + 13 Gy group than in the radiation group (BJIT + 13 Gy: 150.33 ± 11.20, *p* < 0.01, Figures 5(a) and 5(c)).

### 3.3. Protective Effect of BJIT as Demonstrated by Inflammatory Cytokine Levels in Intestinal Samples from Irradiated Mice

**Figure 6:**
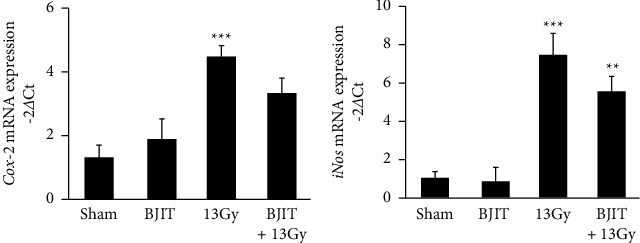
BJIT attenuates the change in the mRNA expression levels of (a) *Cox-*2 and (b) *iNos* in the mouse jejunum after partial abdominal irradiation. Values are reported as mean ± SEM (*n* = 6 per group), ^*∗∗*^*p* < 0.01, ^*∗∗∗*^*p* < 0.001 vs. sham controls. BJIT, Bojungikki-tang.

We studied the mRNA expression levels of inflammatory cytokines (i.e., Cox-2 and iNos) in the small intestine 3.5 days postirradiation. As shown in Figure 6(a), the Cox-2 mRNA levels in the 13 Gy-irradiated mice significantly increased postirradiation (sham: 1.36 ± 0.34, 13 Gy: 4.47 ± 0.45, *p* < 0.001). However, treatment with BJIT did not significantly decrease this parameter compared to the sham group (BJIT + 13 Gy: 3.37 ± 0.40, *p*=0.07; Figure 6(a)). As shown in Figure 6(b), the iNos mRNA levels in the 13 Gy-irradiated mice significantly increased postirradiation (sham: 1.12 ± 0.21, 13 Gy: 7.42 ± 1.23, *p* < 0.001). However, BJIT treatment did not significantly reduce this parameter (BJIT + 13 Gy: 5.31 ± 1.06, *p*=0.08; Figure 6(b)). Although no statistically significant difference was detected, BJIT treatment did attenuate the increase in Cox-2 and iNos mRNA levels.

### 3.4. Active Compounds in BJIT

The number of active compounds identified in BJIT in accordance with the TCMSP was 1,147. These included 128 compounds from Angelicae Gigantis Radix, 85 compounds from Astragali Radix, 55 compounds from Atractylodis Rhizoma Alba, 349 compounds from Bupleuri Radix, 172 compounds from Cimicifugae Rhizoma, 63 compounds from Citri Unshius Pericarpium, 191 compounds from Ginseng Radix, and 280 compounds from Glycyrrhizae Radix et Rhizoma, among which 176 active compounds were overlapping (Supplementary Table 1). ADME screening was limited to compounds with OB ≥ 30% and DL ≥ 0.18, as recommended by TCMSP guidelines [14, 15]. Although ten active compounds, namely, astragaloside IV; ferulic acid (cis and transforms); ginsenosides Rb1, Re, and Rg1; glycyrrhizin; hesperidin; isoferulic acid; and saikosaponin A, did not meet the ADME criteria, they were included because they are major active compounds according to the Korean and Chinese Pharmacopoeia. A total of 173 active compounds were selected through ADME screening and Pharmacopoeia guidelines (Supplementary Table 2).

### 3.5. Selection of Potential Target Genes

**Figure 7:**
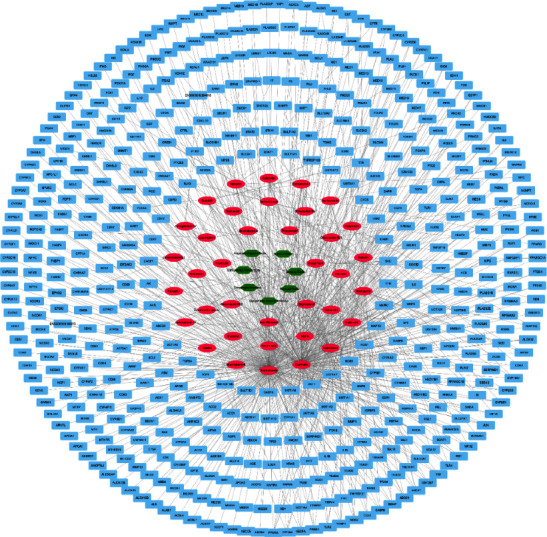
Compound-compound-target network of BJIT. Network of seven herbs (green hexagons), 37 active compounds (pink ovals), and 505 target genes (blue rectangles). This network is composed of 549 nodes and 808 edges. BJIT, Bojungikki-tang.

**Figure 8:**
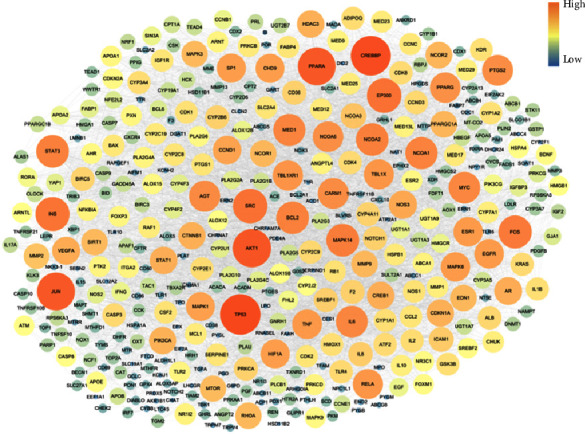
Protein-protein interaction (PPI) network of BJIT compound targets. Topology of PPIs in BJIT obtained from the STITCH database and Cytoscape program; the size of each node is representative of the edge counts with adjacent potential target genes. BJIT, Bojungikki-tang; and STITCH, Search Tool for Interactions of Chemicals and Proteins.

Forty-four active compounds were linked to 772 target genes in the STITCH database with scores ≥0.400 (medium confidence, Supplementary Table 3) [28]. Next, these genes were matched with intestinal injury-related genes (*n* = 8262) in the GeneCards database (Supplementary Table 4), and only genes with scores ≥0.700 were selected. Finally, seven herbs, 505 target genes, and 37 active compounds were selected, and this network consisting of herbs-compounds-genes (H-C-G) was composed of 549 nodes and 808 edges (Figure 7). Then, the target genes were matched with intestinal injury-related genes in the GeneCards database. In total, 406 potential target genes overlapped with disease-associated genes. These potential target genes helped produce a PPI network with the STITCH database, and their topology was analyzed in the Cytoscape program. A topological module represents a locally dense neighborhood in a network, such that nodes have a higher tendency to link to the nodes within the same local neighborhood than to the nodes outside of it [31]. The PPI network consisted of 505 potential target genes, and TP53, AKT1, PPARA, JUN, MAPK14, STAT3, BCL2, PPARG, TNF, EGFR, SP1, VEGFA, MMP9, and MAPK3 were determined to be core potential genes (Figure 8). These genes had high-degree edge counts and were closely related to intestinal injury.

### 3.6. Pathway Analysis Related to Intestinal Injury

**Figure 9:**
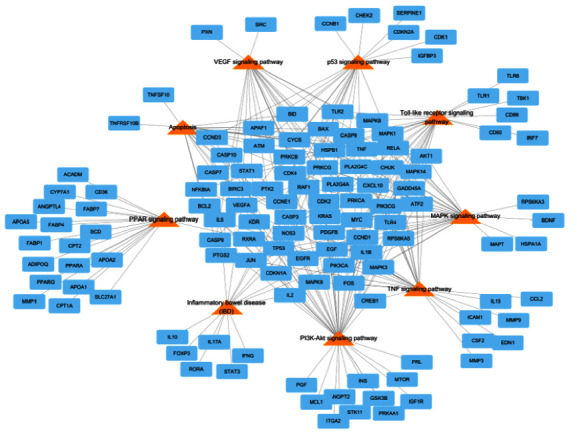
Compound-target gene network linking the protective effects of BJIT against irradiation to potential target genes and signaling pathways. Network of the KEGG pathway (orange triangles) and intestinal injury-related genes (blue rectangles). BJIT, Bojungikki-tang; and KEGG, Kyoto Encyclopedia of Genes and Genomes.

To examine the signaling pathways and functions of genes, we analyzed the gene ontology database and KEGG database and selected pathways with a cutoff *p* value <0.05. The nine pathways associated with intestinal injury were apoptosis, inflammatory bowel disease (IBD), tumor necrosis factor (TNF), p53, vascular endothelial growth factor (VEGF), toll-like receptor, peroxisome proliferator-activated receptors (PPAR), phosphatidylinositol 3′-kinase-Akt (PI3K-Akt), and mitogen-activated protein kinase (MAPK) (Figure 9).

## 4. Discussion

**Figure 10:**
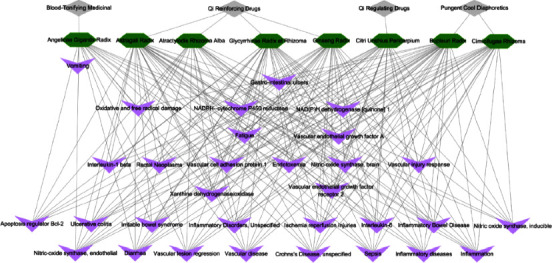
Relationship among the eight herbal medicines comprising BJIT, herbal medicine categories according to oriental medicine theories, and related diseases. Herbal medicines are shown as green hexagons, categories of herbal medicines are indicated by gray diamonds, and the main therapeutic effects of herbal medicines are shown as purple V-shaped structures. BJIT, Bojungikki-tang.

Bojungikki-tang is a popular traditional medicine in Korea, China, and Japan [14, 15]. According to the theory of traditional herbal medicine, each of the eight herbs in BJIT has medicinal effects. Briefly, Astragali Radix, Ginseng Radix, Atractylodis Rhizoma Alba, and Glycyrrhizae Radix et Rhizoma can reinforce Qi. Citri Unshius Pericarpium can regulate Qi, and Angelica Gigantis Radix can “tonify blood.” Cimicifugae Rhizoma and Bupleuri Radix can elevate Yang-Qi. The complex of eight herbs can “tonify the middle” and augment Qi (Figure 10).

Furthermore, a deficiency in middle Qi mainly results in gastrointestinal symptoms, including dyspepsia, inappetence, diarrhea, and epigastric discomfort. Thus, clinically, BJIT is generally used to treat various gastrointestinal disorders, including radiation therapy-induced intestinal injury [17].

In a previous study, we demonstrated that BJIT could attenuate radiation-induced intestinal injury [11]. To confirm the effects of this herbal prescription, we attempted to verify the efficacy of BJIT in protecting against radiation-induced intestinal injury and further explored the potential molecular mechanisms of BJIT components through a systematic approach using network pharmacology.

The pathogenesis of radiation-induced intestinal injury is multifactorial and mainly related to cell apoptosis in the crypt epithelium and inflammatory processes [5, 32]. Apoptosis is one of the most important outcomes of irradiation-mediated intestinal damage. Upon radiation exposure, an imbalance between apoptotic and antiapoptotic factors occurs within the cells [33–35]. The decreased level of *Bcl-*2, an antiapoptotic factor, and simultaneous accumulation of *Bax*, a proapoptotic factor, is associated with an augmentation of the apoptotic response [36]. In the PCR analysis of the present study, 5-Gy irradiation resulted in increased *Bax* expression and decreased *Bcl-*2 expression levels compared to sham mice. In this context, although BJIT elevated *Bcl-*2 levels and suppressed the increased *Bax* expression, no statistically significant differences were noted. The TUNEL assay revealed that BJIT treatment significantly mitigated the number of apoptotic nuclei within the jejunal crypts. Overall, these results show that BJIT administration attenuated the irradiation-induced apoptosis in the intestinal crypts.

Radiation exposure generally suppresses cell proliferation in the crypts, delaying the development of intestinal damage [31, 37]. This loss of proliferative function could exacerbate mucosal inflammation and dysfunction by augmenting intestinal permeability to luminal antigens and bacteria [3]. Hence, the number of surviving crypts and villus length can be used as biodosimetry markers to investigate the adverse effects of radiation [10, 38]. In this study, the villus length of the irradiated mice was significantly shorter than that of the sham mice. Additionally, the irradiated mice showed significantly increased mucosal depth. Conversely, BJIT treatment ameliorated these radiation-induced histopathological changes. Furthermore, we investigated the changes in the expression level of Ki-67, a proliferation marker, in the jejunum via immunohistochemical staining. Irradiation suppressed the number of Ki-67-positive crypts, reflecting a decrease in surviving crypts. The BJIT-treated mice showed a greater number of Ki-67-positive crypts compared to the irradiated group. In this study, BJIT treatment resulted in larger crypt sizes following irradiation, which is one of the indications of crypt regeneration [39, 40].

There is growing evidence supporting the hypothesis that inflammation is involved in the development and pathogenesis of radiation-induced injury in normal tissue [41]. Radiation stimulates the translocation of nuclear factor-kappa B (NF-*κ*B) to the nucleus, increasing the expression of proinflammatory mediators, including iNOS [42, 43]. iNOS levels in the intestines of rats were reportedly elevated as early as 2 h after radiation treatment [44]. The elevated iNOS levels result in COX-2 overexpression, which produces prostaglandins via the metabolism of arachidonic acid [42, 43]. In this study, radiation exposure increased Cox-2 and iNos mRNA levels in the intestine. Although BJIT administration attenuated the upregulation of Cox-2 and iNos mRNA levels, the differences were not statistically significant.

Using network analysis, 1,319 active compounds were extracted from public databases, and 34 active compounds that passed ADME screening were linked to intestinal injury-related genes. BJIT consists of eight herbs, of which Atractylodis Rhizoma Alba was excluded from the network because its active compounds did not exhibit interactions with genes linked to intestinal injury, although it has been used to treat digestive disorders [45]. Beta-sitosterol and stigmasterol in BJIT are classified as plant sterols, and previous studies have reported that plant sterols reduce systemic inflammatory responses [46]. In this context, many flavonoids can influence chronic inflammatory disease at the cellular level and modulate the responses of protein pathways [47]. Seventeen of the 37 compounds in BJIT are categorized as flavonoids in this network. Notably, hesperidin is a flavanone glycoside mainly found in citrus fruits, which reportedly exhibit anti-inflammatory, antimicrobial, anticarcinogenic, and antioxidant effects and is effective in reducing the intensity of small intestine damage [48]. Additionally, flavonoid-rich fractions were able to modulate the NF-*κ*B signaling pathway in a previous study [49]. Thus, these flavonoids in the BJIT network might play a vital role in reducing inflammation.

The core node components linked to intestinal injury-related genes included TP53, AKT1, PPARA, JUN, MAPK14, STAT3, BCL2, PPARG, TNF, EGFR, SP1, VEGFA, MMP9, and MAPK3. In the DAVID and KEGG network pharmacology analysis, intestinal injury-related genes were associated with nine pathways. The apoptosis and p53 pathways are associated with radiation-induced gastrointestinal disturbances, particularly the damage to the small intestine. The antiapoptotic effect has been shown to play one of the most important roles in radioprotection [49–51]. Among the nine pathways, the PI3K-Akt and NF-*κ*B-mediated signaling pathways were closely correlated with the pathogenesis of gastric disease and intestinal mucosal injury [52]. Toll-like receptor (TLR) signaling plays important roles in maintaining intestinal epithelial homeostasis [53]. However, harmful factors in the intestinal tract, such as inflammatory cytokines, activate TLR signaling. TLR activates downstream signaling pathways involving Myd88 to induce nuclear translocation of NF-*κ*B, thereby increasing proinflammatory cytokine production [54]. Signaling by TLRs on intestinal epithelial cells is critical for intestinal injury [55]. TLRs and their ligands provide novel strategies for radiation protection during nuclear accidents as well as for the protection of normal tissues during cancer radiotherapy [56]. Targeting TLRs may represent a novel therapeutic approach in cancer therapy-induced intestinal mucositis. Peroxisome proliferator-activated receptor *γ* (PPAR*γ*) is a nuclear receptor highly expressed in the intestines and plays a key role in inflammation. Further studies should explore the effects of abdominal irradiation on PPARs, their roles and functions in irradiation toxicity, and the possibility of using their ligands for radioprotection [57].

## 5. Conclusions

In summary, BJIT exerts significant protective effects against radiation-induced intestinal injury in a murine model by alleviating the extent of histopathological changes in jejunal crypts and suppressing the levels of inflammatory mediators. Moreover, through subsequent network analysis, we identified TP53, AKT1, PPARA, JUN, MAPK14, STAT3, BCL2, PPARG, TNF, EGFR, SP1, VEGFA, MMP9, and MAPK3 as potential target genes playing pivotal roles in various signaling pathways related to the radioprotective effects of BJIT.

## Figures and Tables

**Table 1:** Primer sequences for qRT-PCR.

Gene	Primer sequences	
*Bax*	FWD	5′-ATGGACGGGTCCGGGGAGCAG-3′
	RVS	5′-CAGTTGAAGTTGCCGTCAGA-3′

*Bcl-*2	FWD	5′-AGCTGCACCTGACGCCCTTCA-3′
	RVS	5′-AGCCAGGAGAAATCACAGAGG-3′

*iNos*	FWD	5′-ATTGGCAACATCAGGTCGGCCATCACT-3′
	RVS	5′-GCTGTGTGTCACAGAAGTCTCGAAGTC-3′

*Cox-*2	FWD	5′-GGAGAGACTATCAAGATAGT-3′
	RVS	5′-ATGGTGAGTAGACTTTTACA-3′

*β-actin*	FWD	5′-TCATGAAGTGTGACGTTGACATCCGT-3′
	RVS	5′-CCTAGAAGCATTTGCGGTTCACGATG-3′

qRT-PCR, quantitative reverse transcription polymerase chain reaction; FWD, forward; RVS, reverse.

## Data Availability

The data that support the findings of this study are available from the corresponding author upon reasonable request.

## Supplementary Materials

Supplementary Materials See the Supplementary Tables (Tables 1–4).
