# Supplementary material for: Protective Effect of Bojungikki-Tang against Radiation-Induced Intestinal Injury in Mice: Experimental Verification and Compound-Target Prediction
Source: Evid Based Complement Alternat Med. 2023 Jan 4;2023:5417813. doi: 10.1155/2023/5417813 (PMC9833920; doi:10.1155/2023/5417813)
Supplement: Supplementary Materials — See the Supplementary Tables (Tables 1–4). [file 5417813.f1.zip › Supple_Tables_1.pdf]

Supplementary Table X1. The list of all active compounds in eight herbs which consisted of BJIT

No.	Herbs	Mol ID	Molecule Name	MW	AlogP	Hdon	Hacc	OB (%)	Caco-2	BBB	DL	FASA-	HL	Save
1	Glycyrrhizae Radix et Rhizoma	MOL000012	Arachic acid	312.6	8.19	1	2	16.66	1.18	1.09	0.19	0.18		
2	Atractylodis Rhizoma Alba	MOL000018	(+/-)-Isoborneol	154.28	1.98	1	1	86.98	1.27	1.6	0.05	0	11.36	
2	Bupleuri Radix	MOL000018	(+/-)-Isoborneol	154.28	1.98	1	1	86.98	1.27	1.6	0.05	0	11.36	
3	Atractylodis Rhizoma Alba	MOL000019	D-Camphene	136.26	2.93	0	0	34.98	1.81	2.19	0.04	0	11.29	
3	Bupleuri Radix	MOL000019	D-Camphene	136.26	2.93	0	0	34.98	1.81	2.19	0.04	0	11.29	
4	Atractylodis Rhizoma Alba	MOL000020	12-Senecioyl-2E,8E,10E-atractylentriol	312.39	2.5	0	4	62.4	0.01	-1.37	0.22	0.12	6.07	
5	Atractylodis Rhizoma Alba	MOL000021	14-Acetyl-12-senecioyl-2E,8E,10E-atractylentriol	355.44	3.21	0	5	60.31	0.33	-1.09	0.31	0.05	5.32	
6	Atractylodis Rhizoma Alba	MOL000022	14-Acetyl-12-senecioyl-2E,8Z,10E-atractylentriol	356.45	3.54	1	5	63.37	0.42	-1.14	0.3	0	6.43	
7	Angelicae Gigantis Radix	MOL000023	(+)-Carvene	136.26	3.5	0	0	39.84	1.83	2.12	0.02	0	11.68	
7	Atractylodis Rhizoma Alba	MOL000023	(+)-Carvene	136.26	3.5	0	0	39.84	1.83	2.12	0.02	0	11.68	
7	Bupleuri Radix	MOL000023	(+)-Carvene	136.26	3.5	0	0	39.84	1.83	2.12	0.02	0	11.68	
8	Atractylodis Rhizoma Alba	MOL000024	alpha-Humulene	204.39	5.04	0	0	22.83	1.84	2.07	0.06	0.28		
8	Bupleuri Radix	MOL000024	alpha-Humulene	204.39	5.04	0	0	22.83	1.84	2.07	0.06	0.28		
9	Atractylodis Rhizoma Alba	MOL000025	alpha-Longipinene	204.39	4.12	0	0	53.26	1.83	2.14	0.12	0	12.06	
10	Atractylodis Rhizoma Alba	MOL000026	Stigmast-22E-en-3beta-ol	414.79	7.89	1	1	10.39	1.43	1.13	0.75	0		
11	Atractylodis Rhizoma Alba	MOL000027	alpha-Curcumene	202.37	5.34	0	0	4.68	1.93	1.99	0.06	0		
11	Bupleuri Radix	MOL000027	alpha-Curcumene	202.37	5.34	0	0	4.68	1.93	1.99	0.06	0		
12	Atractylodis Rhizoma Alba	MOL000028	alpha-Amyrin	426.8	7.35	1	1	39.51	1.42	1.28	0.76	0	3.83	
13	Atractylodis Rhizoma Alba	MOL000029	beta-Humulene	204.39	5.09	0	0	26.87	1.82	2.01	0.06	0		
13	Bupleuri Radix	MOL000029	beta-Humulene	204.39	5.09	0	0	26.87	1.82	2.01	0.06	0		
13	Ginseng Radix	MOL000029	beta-Humulene	204.39	5.09	0	0	26.87	1.82	2.01	0.06	0		
14	Atractylodis Rhizoma Alba	MOL000030	(1R)-2-Methyl-1-phenylprop-2-en-1-ol	148.22	2.32	1	1	75.1	1.27	1.38	0.03	0	-2.13	
15	Atractylodis Rhizoma Alba	MOL000031	(3S)-3-[(1R)-1,5-Dimethylhex-4-enyl]-6-methylenecyclohex	204.39	5.14	0	0	19.86	1.88	1.99	0.06	0		
16	Atractylodis Rhizoma Alba	MOL000032	beta-Eudesmol	222.41	3.72	1	1	26.09	1.32	1.38	0.1	0		
16	Bupleuri Radix	MOL000032	beta-Eudesmol	222.41	3.72	1	1	26.09	1.32	1.38	0.1	0		
16	Cimicifugae Rhizoma	MOL000032	beta-Eudesmol	222.41	3.72	1	1	26.09	1.32	1.38	0.1	0		
17	Astragali Radix	MOL000033	(24S)-24-Propylcholesta-5-ene-3beta-ol	428.82	8.54	1	1	36.23	1.45	1.09	0.78	0	5.22	
17	Atractylodis Rhizoma Alba	MOL000033	(24S)-24-Propylcholesta-5-ene-3beta-ol	428.82	8.54	1	1	36.23	1.45	1.09	0.78	0	5.22	
18	Atractylodis Rhizoma Alba	MOL000034	2-[(1R,3S,4S)-3-Isopropenyl-4-methyl-4-vinylcyclohexyl]pro	222.41	3.7	1	1	19.03	1.37	1.46	0.07	0		
19	Angelicae Gigantis Radix	MOL000035	beta-Selinene	204.39	4.81	0	0	24.39	1.83	2.12	0.08	0		
19	Atractylodis Rhizoma Alba	MOL000035	beta-Selinene	204.39	4.81	0	0	24.39	1.83	2.12	0.08	0		
19	Ginseng Radix	MOL000035	beta-Selinene	204.39	4.81	0	0	24.39	1.83	2.12	0.08	0		
20	Atractylodis Rhizoma Alba	MOL000036	beta-Caryophyllene	204.39	4.75	0	0	29.7	1.83	2.07	0.09	0		
20	Bupleuri Radix	MOL000036	beta-Caryophyllene	204.39	4.75	0	0	29.7	1.83	2.07	0.09	0		
20	Ginseng Radix	MOL000036	beta-Caryophyllene	204.39	4.75	0	0	29.7	1.83	2.07	0.09	0		
21	Atractylodis Rhizoma Alba	MOL000037	gamma-Elemene	204.39	4.93	0	0	23.79	1.87	2.1	0.06	0		
22	Atractylodis Rhizoma Alba	MOL000038	Acridine	179.23	3.35	0	1	33.71	1.63	1.53	0.1	0	6.82	
23	Atractylodis Rhizoma Alba	MOL000039	(1S,2R,4R)-Neoiso-dihydrocarveol	154.28	2.58	1	1	52.4	1.38	1.69	0.03	0	11.46	
24	Angelicae Gigantis Radix	MOL000040	Scopoletol	192.18	1.62	1	4	27.77	0.71	0.3	0.08	0		
24	Atractylodis Rhizoma Alba	MOL000040	Scopoletol	192.18	1.62	1	4	27.77	0.71	0.3	0.08	0		
24	Bupleuri Radix	MOL000040	Scopoletol	192.18	1.62	1	4	27.77	0.71	0.3	0.08	0		
24	Glycyrrhizae Radix et Rhizoma	MOL000040	Scopoletol	192.18	1.62	1	4	27.77	0.71	0.3	0.08	0		
25	Atractylodis Rhizoma Alba	MOL000041	Phenylalanine	165.21	0.96	3	3	41.62	0.36	0.22	0.04	0	4.62	
26	Atractylodis Rhizoma Alba	MOL000042	(L)-Alanine	89.11	-0.6	3	3	87.69	-0.34	-0.77	0.01	0	11.58	
27	Atractylodis Rhizoma Alba	MOL000043	Atractylenolide i	230.33	3.32	0	2	37.37	1.3	1.29	0.15	0	7.1	
28	Atractylodis Rhizoma Alba	MOL000044	AtractylenolideII	232.35	3.57	0	2	47.5	1.3	1.37	0.15	0	7.21	
29	Atractylodis Rhizoma Alba	MOL000045	Atractylenolide iii	248.35	2.93	1	3	68.11	0.75	0.63	0.17	0	7.17	
30	Atractylodis Rhizoma Alba	MOL000046	Atractylone	216.35	4.11	0	1	41.1	1.76	1.85	0.13	0	1.74	
31	Atractylodis Rhizoma Alba	MOL000047	Juniper camphor	222.41	3.93	1	1	33.3	1.44	1.61	0.1	0	9.27	
32	Atractylodis Rhizoma Alba	MOL000048	(5E,9Z)-3,6,10-Trimethyl-4,7,8,11-tetrahydrocyclodeca[b]fur	216.35	4.63	0	1	43.17	1.77	1.83	0.1	0	-1.4	
33	Atractylodis Rhizoma Alba	MOL000049	3β-Acetoxyatractylone	274.39	3.39	0	3	54.07	1.13	1.08	0.22	0	-1.31	
34	Atractylodis Rhizoma Alba	MOL000050	2-Aminoacetic acid	75.08	-0.98	3	3	48.74	-0.56	-1.03	0	0	11.95	
35	Atractylodis Rhizoma Alba	MOL000051	Polymannose	180.18	-2.68	5	6	1.76	-1.94	-4.53	0.03	0		
36	Atractylodis Rhizoma Alba	MOL000052	Gulutamine	147.15	-0.92	4	5	6.66	-1.05	-1.97	0.02	0		
37	Atractylodis Rhizoma Alba	MOL000053	Methose	180.18	-2.69	5	6	1.68	-1.8	-4.58	0.03	0		
38	Astragali Radix	MOL000054	L-Arginin	174.24	-1.11	7	6	47.64	-0.49	-1.04	0.03	0	0.85	
38	Atractylodis Rhizoma Alba	MOL000054	L-Arginin	174.24	-1.11	7	6	47.64	-0.49	-1.04	0.03	0	0.85	
39	Atractylodis Rhizoma Alba	MOL000055	L-Lysin	146.22	-0.68	5	4	29.33	-0.66	-1.44	0.02	0		
40	Atractylodis Rhizoma Alba	MOL000056	(2S)-2-Amino-3-(4-hydroxyphenyl)propanoic acid	181.21	0.69	4	4	57.55	-0.1	-0.49	0.05	0	0.96	
41	Atractylodis Rhizoma Alba	MOL000057	Diisobutyl benzene-1,2-dicarboxylate	278.38	3.92	0	4	49.63	0.85	0.68	0.13	0	3.94	
41	Bupleuri Radix	MOL000057	Diisobutyl benzene-1,2-dicarboxylate	278.38	3.92	0	4	49.63	0.85	0.68	0.13	0	3.94	
41	Citri Unshius Pericarpium	MOL000057	Diisobutyl benzene-1,2-dicarboxylate	278.38	3.92	0	4	49.63	0.85	0.68	0.13	0	3.94	
41	Glycyrrhizae Radix et Rhizoma	MOL000057	Diisobutyl benzene-1,2-dicarboxylate	278.38	3.92	0	4	49.63	0.85	0.68	0.13	0	3.94	
42	Atractylodis Rhizoma Alba	MOL000058	Hinesol	222.41	3.67	1	1	38.59	1.34	1.42	0.09	0	7.61	
43	Atractylodis Rhizoma Alba	MOL000059	Uridine	244.23	-2.45	4	8	10.49	-1.14	-1.61	0.11	0		
44	Atractylodis Rhizoma Alba	MOL000060	Selina-4(14),7(11)-dien-8-one	218.37	3.81	0	1	32.31	1.42	1.57	0.1	0	7.68	
45	Astragali Radix	MOL000061	Prolinum	115.15	-0.06	2	3	77.57	0.22	0.29	0.01	0	11.13	
45	Atractylodis Rhizoma Alba	MOL000061	Prolinum	115.15	-0.06	2	3	77.57	0.22	0.29	0.01	0	11.13	
46	Atractylodis Rhizoma Alba	MOL000062	Biatractylolide	462.68	6.68	0	4	17.45	0.83	0.37	0.81	0		
47	Atractylodis Rhizoma Alba	MOL000063	Atractylodes macrocephala	462.68	6.68	0	4	14.6	0.88	0.6	0.81	0		
48	Atractylodis Rhizoma Alba	MOL000064	D-Serin	105.11	-1.49	4	4	83.59	-0.94	-1.75	0.01	0	11.51	
49	Atractylodis Rhizoma Alba	MOL000065	(2S)-2-Aminosuccinic acid	133.12	-1.25	4	5	79.74	-1.02	-1.53	0.02	0	11.38	
50	Atractylodis Rhizoma Alba	MOL000066	Alloaromadedrene	204.39	4.22	0	0	53.46	1.83	2.1	0.1	0	12.51	
50	Ginseng Radix	MOL000066	Alloaromadedrene	204.39	4.22	0	0	53.46	1.83	2.1	0.1	0	12.51	
51	Atractylodis Rhizoma Alba	MOL000067	L-Valin	117.17	0.24	3	3	53.33	0.04	-0.14	0.01	0	11.34	
52	Atractylodis Rhizoma Alba	MOL000068	(S)-Isoleucine	131.2	0.7	3	3	59.05	0.06	-0.11	0.02	0	11.21	
53	Angelicae Gigantis Radix	MOL000069	Palmitic acid	256.48	6.37	1	2	19.3	1.09	1	0.1	0		
53	Astragali Radix	MOL000069	Palmitic acid	256.48	6.37	1	2	19.3	1.09	1	0.1	0		
53	Atractylodis Rhizoma Alba	MOL000069	Palmitic acid	256.48	6.37	1	2	19.3	1.09	1	0.1	0		
53	Bupleuri Radix	MOL000069	Palmitic acid	256.48	6.37	1	2	19.3	1.09	1	0.1	0		
53	Cimicifugae Rhizoma	MOL000069	Palmitic acid	256.48	6.37	1	2	19.3	1.09	1	0.1	0		
53	Ginseng Radix	MOL000069	Palmitic acid	256.48	6.37	1	2	19.3	1.09	1	0.1	0		
54	Atractylodis Rhizoma Alba	MOL000070	Ethyl pivaloylacetate	172.25	1.69	0	3	40.52	0.82	0.83	0.03	0	1.92	
55	Atractylodis Rhizoma Alba	MOL000071	Istidina	155.18	-1.01	4	4	53.18	-0.25	-0.4	0.03	0	-5.72	
56	Atractylodis Rhizoma Alba	MOL000072	8β-Ethoxy atractylenolide Ⅲ	276.41	3.68	0	3	35.95	1.08	1.12	0.21	0	8.34	
57	Cimicifugae Rhizoma	MOL000087	beta-Sitosterol 3-O-glucoside	576.95	6.34	4	6	20.63	-0.45	-1.18	0.62	0.23		
58	Astragali Radix	MOL000098	Quercetin	302.25	1.5	5	7	46.43	0.05	-0.77	0.28	0.38	14.4	
58	Bupleuri Radix	MOL000098	Quercetin	302.25	1.5	5	7	46.43	0.05	-0.77	0.28	0.38	14.4	
58	Glycyrrhizae Radix et Rhizoma	MOL000098	Quercetin	302.25	1.5	5	7	46.43	0.05	-0.77	0.28	0.38	14.4	
59	Glycyrrhizae Radix et Rhizoma	MOL000105	Protocatechuic acid	154.13	0.9	3	4	25.37	0.1	-0.17	0.04	0.43		
60	Astragali Radix	MOL000114	Vanillic acid	168.16	1.15	2	4	35.47	0.43	0.09	0.04	0.34	11.62	
61	Angelicae Gigantis Radix	MOL000116	Nonanal	142.27	3.22	0	1	40.28	1.31	1.5	0.02	0.21	6.35	
61	Bupleuri Radix	MOL000116	Nonanal	142.27	3.22	0	1	40.28	1.31	1.5	0.02	0.21	6.35	
61	Cimicifugae Rhizoma	MOL000116	Nonanal	142.27	3.22	0	1	40.28	1.31	1.5	0.02	0.21	6.35	
62	Angelicae Gigantis Radix	MOL000117	Cymol	134.24	3.51	0	0	27.2	1.86	2.1	0.02	0.34		
62	Bupleuri Radix	MOL000117	Cymol	134.24	3.51	0	0	27.2	1.86	2.1	0.02	0.34		
63	Bupleuri Radix	MOL000118	(L)-alpha-Terpineol	154.28	2.42	1	1	48.8	1.39	1.72	0.03	0.27	11.35	
63	Cimicifugae Rhizoma	MOL000118	(L)-alpha-Terpineol	154.28	2.42	1	1	48.8	1.39	1.72	0.03	0.27	11.35	
63	Citri Unshius Pericarpium	MOL000118	(L)-alpha-Terpineol	154.28	2.42	1	1	48.8	1.39	1.72	0.03	0.27	11.35	
63	Glycyrrhizae Radix et Rhizoma	MOL000118	(L)-alpha-Terpineol	154.28	2.42	1	1	48.8	1.39	1.72	0.03	0.27	11.35	
64	Cimicifugae Rhizoma	MOL000119	(+)-Nerolidol	222.41	4.56	1	1	40.43	1.44	1.31	0.06	0.3	4.73	
65	Bupleuri Radix	MOL000120	Dec-2-enal	154.28	3.66	0	1	18.55	1.4	1.59	0.02	0.27		
66	Angelicae Gigantis Radix	MOL000121	Decanal	156.3	3.68	0	1	29.81	1.34	1.44	0.02	0.2		
66	Bupleuri Radix	MOL000121	Decanal	156.3	3.68	0	1	29.81	1.34	1.44	0.02	0.2		
66	Cimicifugae Rhizoma	MOL000121	Decanal	156.3	3.68	0	1	29.81	1.34	1.44	0.02	0.2		
66	Citri Unshius Pericarpium	MOL000121	Decanal	156.3	3.68	0	1	29.81	1.34	1.44	0.02	0.2		
67	Bupleuri Radix	MOL000122	1,8-Cineole	154.28	2.15	0	1	39.73	1.57	2.06	0.05	0.24	11.29	
68	Bupleuri Radix	MOL000123	Geraniol	154.28	2.93	1	1	23.93	1.19	1.14	0.02	0.27		
69	Cimicifugae Rhizoma	MOL000124	Citral	152.26	3.19	0	1	22.52	1.33	1.43	0.02	0.33		
70	Angelicae Gigantis Radix	MOL000125	(-)-alpha-Pinene	136.26	2.87	0	0	46.25	1.85	2.3	0.05	0.25	11.42	

70	Bupleuri Radix	MOL000125	(-)-alpha-Pinene	136.26	2.87	0	0	46.25	1.85	2.3	0.05	0.25	11.42
70	Citri Unshius Pericarpium	MOL000125	(-)-alpha-Pinene	136.26	2.87	0	0	46.25	1.85	2.3	0.05	0.25	11.42
71	Bupleuri Radix	MOL000126	(-)-Nopinene	136.26	2.93	0	0	44.84	1.8	2.12	0.05	0.27	11.32
72	Bupleuri Radix	MOL000127	(Z)-Citral	152.26	3.19	0	1	19.48	1.36	1.51	0.02	0.34	
72	Cimicifugae Rhizoma	MOL000127	(Z)-Citral	152.26	3.19	0	1	19.48	1.36	1.51	0.02	0.34	
72	Citri Unshius Pericarpium	MOL000127	(Z)-Citral	152.26	3.19	0	1	19.48	1.36	1.51	0.02	0.34	
73	Bupleuri Radix	MOL000128	Nerylacetate	196.32	3.31	0	2	25.94	1.28	1.28	0.04	0.27	
74	Astragali Radix	MOL000131	9Z,12Z-Linoleic acid	280.5	6.39	1	2	41.9	1.16	0.9	0.14	0.25	7.5
74	Bupleuri Radix	MOL000131	9Z,12Z-Linoleic acid	280.5	6.39	1	2	41.9	1.16	0.9	0.14	0.25	7.5
75	Angelicae Gigantis Radix	MOL000162	beta-Chamigrene	204.39	4.71	0	0	31.99	1.82	2.07	0.08	0	8.59
76	Citri Unshius Pericarpium	MOL000168	()-2-Carene	136.26	2.87	0	0	46.69	1.89	2.27	0.04	0.26	11.42
77	Bupleuri Radix	MOL000169	alpha-Guaiene	204.39	4.99	0	0	25.93	1.81	2.09	0.07	0.26	
78	Bupleuri Radix	MOL000172	Furol	96.09	0.99	0	2	34.35	1.08	1.51	0.01	0.2	4.53
78	Cimicifugae Rhizoma	MOL000172	Furol	96.09	0.99	0	2	34.35	1.08	1.51	0.01	0.2	4.53
79	Bupleuri Radix	MOL000193	(Z)-Caryophyllene	204.39	4.75	0	0	30.29	1.82	2.15	0.09	0.28	8
80	Bupleuri Radix	MOL000196	L-Bornyl acetate	196.32	2.35	0	2	65.52	1.29	1.59	0.08	0.23	6.94
81	Angelicae Gigantis Radix	MOL000197	Myrcene	136.26	3.69	0	0	24.96	1.84	1.98	0.02	0.37	
81	Bupleuri Radix	MOL000197	Myrcene	136.26	3.69	0	0	24.96	1.84	1.98	0.02	0.37	
82	Bupleuri Radix	MOL000198	(R)-Linalool	154.28	2.74	1	1	39.8	1.33	1.36	0.02	0.32	6.48
82	Cimicifugae Rhizoma	MOL000198	(R)-Linalool	154.28	2.74	1	1	39.8	1.33	1.36	0.02	0.32	6.48
82	Citri Unshius Pericarpium	MOL000198	(R)-Linalool	154.28	2.74	1	1	39.8	1.33	1.36	0.02	0.32	6.48
83	Angelicae Gigantis Radix	MOL000199	Safrol	162.2	2.61	0	2	45.34	1.44	1.29	0.05	0.38	5
84	Angelicae Gigantis Radix	MOL000201	p-Ocimene	136.26	3.63	0	0	15.06	1.85	1.99	0.02	0.39	
84	Bupleuri Radix	MOL000201	p-Ocimene	136.26	3.63	0	0	15.06	1.85	1.99	0.02	0.39	
84	Citri Unshius Pericarpium	MOL000201	p-Ocimene	136.26	3.63	0	0	15.06	1.85	1.99	0.02	0.39	
85	Angelicae Gigantis Radix	MOL000202	Moslene	136.26	3.45	0	0	33.02	1.88	2.05	0.02	0.27	11.08
86	Angelicae Gigantis Radix	MOL000206	Isoeugenol	164.22	2.5	1	2	70.1	1.38	1.28	0.04	0.33	0.65
87	Bupleuri Radix	MOL000207	Methyleugenol	178.25	2.81	0	2	73.36	1.47	1.41	0.04	0.27	2.92
87	Cimicifugae Rhizoma	MOL000207	Methyleugenol	178.25	2.81	0	2	73.36	1.47	1.41	0.04	0.27	2.92
88	Astragali Radix	MOL000211	Mairin	456.78	6.52	2	3	55.38	0.73	0.22	0.78	0.26	8.87
88	Glycyrrhizae Radix et Rhizoma	MOL000211	Mairin	456.78	6.52	2	3	55.38	0.73	0.22	0.78	0.26	8.87
89	Cimicifugae Rhizoma	MOL000223	Caffeic acid	180.17	1.37	3	4	25.76	0.21	-0.26	0.05	0.44	
90	Angelicae Gigantis Radix	MOL000232	alpha-Terpineol	154.28	2.42	1	1	46.3	1.28	1.4	0.03	0.26	10.75
91	Bupleuri Radix	MOL000233	delta-Terpineol	154.28	2.47	1	1	55.11	1.28	1.44	0.03	0.28	10.97
92	Bupleuri Radix	MOL000234	L-Limonen	136.26	3.5	0	0	38.09	1.83	2.13	0.02	0.29	11.64
93	Astragali Radix	MOL000239	Jaranol	314.31	2.09	2	6	50.83	0.61	-0.22	0.29	0.29	15.5
93	Glycyrrhizae Radix et Rhizoma	MOL000239	Jaranol	314.31	2.09	2	6	50.83	0.61	-0.22	0.29	0.29	15.5
94	Bupleuri Radix	MOL000244	Borneol	154.28	1.98	1	1	81.8	1.22	1.47	0.05	0.24	11.36
94	Cimicifugae Rhizoma	MOL000244	Borneol	154.28	1.98	1	1	81.8	1.22	1.47	0.05	0.24	11.36
95	Bupleuri Radix	MOL000247	(Z,Z)-Farnesol	222.41	4.76	1	1	41.14	1.25	1.15	0.06	0.28	6.29
96	Astragali Radix	MOL000251	Rhamnocitrin	300.28	2.02	3	6	12.9	0.48	-0.38	0.27	0.32	
97	Bupleuri Radix	MOL000254	Eugenol	164.22	2.55	1	2	56.24	1.35	1.32	0.04	0.32	0.92
97	Cimicifugae Rhizoma	MOL000254	Eugenol	164.22	2.55	1	2	56.24	1.35	1.32	0.04	0.32	0.92
98	Angelicae Gigantis Radix	MOL000259	O-Thymol	150.24	3.24	1	1	43.28	1.58	1.71	0.03	0.33	11.29
98	Bupleuri Radix	MOL000259	O-Thymol	150.24	3.24	1	1	43.28	1.58	1.71	0.03	0.33	11.29
98	Citri Unshius Pericarpium	MOL000259	O-Thymol	150.24	3.24	1	1	43.28	1.58	1.71	0.03	0.33	11.29
99	Bupleuri Radix	MOL000263	Oleanolic acid	456.78	6.42	2	3	29.02	0.59	0.07	0.76	0.25	
99	Glycyrrhizae Radix et Rhizoma	MOL000263	Oleanolic acid	456.78	6.42	2	3	29.02	0.59	0.07	0.76	0.25	
100	Citri Unshius Pericarpium	MOL000268	(-)-Sabinene	136.26	2.93	0	0	46.21	1.83	2.18	0.04	0.31	11.47
101	Cimicifugae Rhizoma	MOL000269	Elemicin	208.28	2.79	0	3	21.94	1.41	1.28	0.06	0.2	
101	Ginseng Radix	MOL000269	Elemicin	208.28	2.79	0	3	21.94	1.41	1.28	0.06	0.2	
102	Angelicae Gigantis Radix	MOL000270	(+)-3-Carene	136.26	2.87	0	0	45.2	1.84	2.15	0.04	0.27	11.44
102	Bupleuri Radix	MOL000270	(+)-3-Carene	136.26	2.87	0	0	45.2	1.84	2.15	0.04	0.27	11.44
103	Bupleuri Radix	MOL000271	l-Carvone	150.24	2.36	0	1	49.47	1.35	1.65	0.03	0.33	11.75
104	Astragali Radix	MOL000296	Hederagenin	414.79	8.08	1	1	36.91	1.32	0.96	0.75	0	5.35
105	Bupleuri Radix	MOL000302	Undecanoic acid	186.33	4.09	1	2	30.14	0.98	0.94	0.03	0	4.93
106	Bupleuri Radix	MOL000303	Caprylic acid	144.24	2.72	1	2	16.4	0.9	1.02	0.02	0	
106	Cimicifugae Rhizoma	MOL000303	Caprylic acid	144.24	2.72	1	2	16.4	0.9	1.02	0.02	0	
107	Bupleuri Radix	MOL000305	Lauric acid	200.36	4.54	1	2	23.59	1.02	1.1	0.04	0	
107	Cimicifugae Rhizoma	MOL000305	Lauric acid	200.36	4.54	1	2	23.59	1.02	1.1	0.04	0	
107	Citri Unshius Pericarpium	MOL000305	Lauric acid	200.36	4.54	1	2	23.59	1.02	1.1	0.04	0	
108	Angelicae Gigantis Radix	MOL000346	Succinic acid	118.1	-0.41	2	4	29.62	-0.44	-0.71	0.01	0.42	
109	Astragali Radix	MOL000354	Isorhamnetin	316.28	1.76	4	7	49.6	0.31	-0.54	0.31	0.32	14.34
109	Bupleuri Radix	MOL000354	Isorhamnetin	316.28	1.76	4	7	49.6	0.31	-0.54	0.31	0.32	14.34
109	Glycyrrhizae Radix et Rhizoma	MOL000354	Isorhamnetin	316.28	1.76	4	7	49.6	0.31	-0.54	0.31	0.32	14.34
110	Astragali Radix	MOL000356	Lupeol	426.8	7.4	1	1	12.12	1.46	1.29	0.78	0.24	
111	Angelicae Gigantis Radix	MOL000357	Sitogluside	576.95	6.34	4	6	20.63	-0.14	-0.93	0.62	0.23	
111	Cimicifugae Rhizoma	MOL000357	Sitogluside	576.95	6.34	4	6	20.63	-0.14	-0.93	0.62	0.23	
112	Angelicae Gigantis Radix	MOL000358	beta-Sitosterol	414.79	8.08	1	1	36.91	1.32	0.99	0.75	0.23	5.36
112	Ginseng Radix	MOL000358	beta-Sitosterol	414.79	8.08	1	1	36.91	1.32	0.99	0.75	0.23	5.36
112	Cimicifugae Rhizoma	MOL000359	beta-Sitosterol	414.79	8.08	1	1	36.91	1.32	0.87	0.75	0.22	5.37
112	Citri Unshius Pericarpium	MOL000359	beta-Sitosterol	414.79	8.08	1	1	36.91	1.32	0.87	0.75	0.22	5.37
112	Glycyrrhizae Radix et Rhizoma	MOL000359	beta-Sitosterol	414.79	8.08	1	1	36.91	1.32	0.87	0.75	0.22	5.37
113	Angelicae Gigantis Radix	MOL000360	trans-Ferulic Acid	194.2	1.62	2	4	39.56	0.47	-0.03	0.06	0.34	2.38
113	Cimicifugae Rhizoma	MOL000360	trans-Ferulic Acid	194.2	1.62	2	4	39.56	0.47	-0.03	0.06	0.34	2.38
114	Astragali Radix	MOL000371	3,9-di-O-Methylnissolin	314.36	2.89	0	5	53.74	1.18	0.63	0.48	0	9
115	Astragali Radix	MOL000372	3-Hydroxy-2-picoline	109.14	0.7	1	2	62.47	1.05	1.02	0.02	0	11.95
116	Astragali Radix	MOL000373	5-O-Methylvisammioside	452.5	0.27	4	10	5.38	-0.8	-1.14	0.81	0	
117	Astragali Radix	MOL000374	5'-Hydroxyiso-muronulatol-2',5'-di-O-glucoside	642.67	-0.95	9	16	41.72	-2.47	-3.62	0.69	0	2.52
118	Astragali Radix	MOL000375	5'-Hydroxyiso-muronulatol-2',5'-di-O-glucoside_qt	480.51	0.96	6	11	3.65	-1.11	-1.89	0.8	0	
119	Astragali Radix	MOL000376	7,2'-Dihydroxy-3',4'-dimethoxyisoflavone-7-O-β-D-glucosi	476.47	0.4	5	11	16.16	-0.87	-1.85	0.86	0	
120	Astragali Radix	MOL000377	7-Hydroxy-3-(2-hydroxy-3,4-dimethoxy-phenyl)chromone	314.31	2.3	2	6	5.45	0.58	-0.21	0.3	0	
121	Astragali Radix	MOL000378	7-O-Methylisomucronulatol	316.38	3.38	1	5	74.69	1.08	0.84	0.3	0	2.98
122	Astragali Radix	MOL000379	9-O-Methylnissolin 3-O-glucoside	462.49	0.74	4	10	36.74	-0.63	-1.5	0.92	0	13.06
123	Astragali Radix	MOL000380	Astrapterocarpan	300.33	2.64	1	5	64.26	0.93	0.55	0.42	0	8.49
124	Astragali Radix	MOL000381	13-Hydroxy-9,11-octadecadienoic acid	296.5	5.29	2	3	35.6	0.44	-0.38	0.17	0	4.63
125	Astragali Radix	MOL000382	Arabinose,d	150.15	-2.17	4	5	1.87	-1.59	-3.98	0.02	0	
125	Bupleuri Radix	MOL000382	Arabinose,d	150.15	-2.17	4	5	1.87	-1.59	-3.98	0.02	0	
126	Angelicae Gigantis Radix	MOL000383	D-Galacturonic acid, homopolymer	194.16	-2.47	5	7	29.75	-2	-4.71	0.04	0	
126	Astragali Radix	MOL000383	D-Galacturonic acid, homopolymer	194.16	-2.47	5	7	29.75	-2	-4.71	0.04	0	
127	Astragali Radix	MOL000384	DL-Glucuronic acid	194.16	-2.47	5	7	3.35	-1.91	-4.72	0.04	0	
128	Astragali Radix	MOL000386	Fucopyranose, L-	164.18	-1.8	4	5	42.51	-1.32	-3.58	0.03	0	11.41
129	Astragali Radix	MOL000387	Bifendate	418.38	2.56	0	10	31.1	0.15	-0.06	0.67	0	17.96
130	Astragali Radix	MOL000388	gamma-Aminobutyric acid	103.14	-0.62	3	3	24.09	-0.26	-0.57	0.01	0	
131	Angelicae Gigantis Radix	MOL000389	cis-Ferulic acid	194.2	1.62	2	4	54.97	0.53	0.36	0.06	0	2.58
131	Astragali Radix	MOL000389	cis-Ferulic acid	194.2	1.62	2	4	54.97	0.53	0.36	0.06	0	2.58
132	Astragali Radix	MOL000390	Daidzein	254.25	2.33	2	4	19.44	0.59	-0.22	0.19	0	
133	Astragali Radix	MOL000391	Ononin	430.44	0.68	4	9	11.52	-0.74	-1.67	0.78	0	
133	Glycyrrhizae Radix et Rhizoma	MOL000391	Ononin	430.44	0.68	4	9	11.52	-0.74	-1.67	0.78	0	
134	Astragali Radix	MOL000392	Formononetin	268.28	2.58	1	4	69.67	0.78	0.02	0.21	0	17.04
134	Glycyrrhizae Radix et Rhizoma	MOL000392	Formononetin	268.28	2.58	1	4	69.67	0.78	0.02	0.21	0	17.04
135	Astragali Radix	MOL000393	Soyasaponin I	943.26	0.97	11	18	2.06	-2.75	-3.38	0.05	0	
136	Astragali Radix	MOL000394	Choline	104.2	-1.57	1	1	0.47	0.86	0.64	0.01	0	
137	Astragali Radix	MOL000395	(2S)-2-Amino-4-guanidinooxy-butanoic acid	176.21	-1.84	7	7	54.95	-0.72	-1.12	0.03	0	0.99
138	Astragali Radix	MOL000396	(+)-Syringaresinol	418.48	2.1	2	8	3.29	0.47	-0.34	0.72	0	
139	Astragali Radix	MOL000397	cis-p-Coumarate	164.17	1.64	2	3	45.98	0.46	0.36	0.04	0	4.89
140	Astragali Radix	MOL000398	Isoflavanone	316.33	2.42	2	6	109.99	0.53	0.17	0.3	0	15.51
141	Astragali Radix	MOL000399	Docosanoate	340.66	9.11	1	2	15.69	1.21	0.91	0.26	0	
142	Astragali Radix	MOL000400	Flavaxin	376.41	0.23	5	10	18.18	-1.52	-1.81	0.5	0	
143	Astragali Radix	MOL000401	AstragalosideI	869.17	0.41	7	16	46.79	-2.28	-3.58	0.11	0	13.14
144	Astragali Radix	MOL000402	AstragalosideI_qt	707.01	2.16	4	11	12.34	-1.15	-2.19	0.2	0	
145	Astragali Radix	MOL000403	AstragalosideII	827.13	0.03	8	15	46.06	-2.05	-3.26	0.13	0	10.58
146	Astragali Radix	MOL000404	AstragalosideII_qt	664.97	1.78	5	10	11.55	-1.05	-2.21	0.25	0	

147	Astragali Radix	MOL000405	AstragalosideⅢ	785.09	-0.35	9	14	31.83	-2.26	-3.37	0.1	0	9.05
148	Astragali Radix	MOL000406	AstragalosideⅢ_qt	622.93	1.4	6	9	5.35	-1.26	-2.18	0.32	0	
149	Astragali Radix	MOL000407	AstragalosideIV	785.09	-0.35	9	14	22.5	-2.11	-3.41	0.15	0	
150	Astragali Radix	MOL000408	AstragalosideIV_qt	622.93	1.4	6	9	7.07	-1.11	-2.07	0.32	0	
151	Astragali Radix	MOL000411	Astraisoflavanin	464.51	1.22	5	10	18.37	-0.76	-1.56	0.86	0	
152	Astragali Radix	MOL000412	Mucronulatol	302.35	3.13	2	5	4.22	0.93	0.4	0.26	0	
153	Astragali Radix	MOL000413	Astrachrysoside A	769.09	0.54	8	13	24.55	-1.93	-2.96	0.1	0	
154	Astragali Radix	MOL000414	Caffeate	180.17	1.37	3	4	54.97	0.27	0.11	0.05	0	1.63
155	Astragali Radix	MOL000415	Rutin	610.57	-1.45	10	16	3.2	-1.93	-2.75	0.68	0	
155	Bupleuri Radix	MOL000415	Rutin	610.57	-1.45	10	16	3.2	-1.93	-2.75	0.68	0	
155	Glycyrrhizae Radix et Rhizoma	MOL000415	Rutin	610.57	-1.45	10	16	3.2	-1.93	-2.75	0.68	0	
156	Astragali Radix	MOL000416	Lariciresinol	360.44	2.46	3	6	5.53	0.27	-0.49	0.38	0	
157	Astragali Radix	MOL000417	Calycosin	284.28	2.32	2	5	47.75	0.52	-0.43	0.24	0	17.1
157	Glycyrrhizae Radix et Rhizoma	MOL000417	Calycosin	284.28	2.32	2	5	47.75	0.52	-0.43	0.24	0	17.1
158	Astragali Radix	MOL000418	3'-Hydroxy-4'-methoxyisoflavone-7-O-beta-D-glucoside	446.44	0.41	5	10	10.05	-0.93	-1.82	0.81	0	
159	Astragali Radix	MOL000419	Astrasieversianin XV	901.22	-0.7	10	17	11.19	-2.72	-4.03	0.07	0	
160	Astragali Radix	MOL000420	DL-Xylose	150.15	-2.17	4	5	51.08	-1.27	-3.58	0.02	0	11.37
160	Bupleuri Radix	MOL000420	Xylose	150.15	-2.17	4	5	51.08	-1.27	-3.58	0.02	0	11.37
161	Angelicae Gigantis Radix	MOL000421	Nicotinic acid	123.12	0.28	1	3	47.65	0.34	0.21	0.02	0	11.98
161	Astragali Radix	MOL000421	Nicotinic acid	123.12	0.28	1	3	47.65	0.34	0.21	0.02	0	11.98
162	Astragali Radix	MOL000422	Kaempferol	286.25	1.77	4	6	41.88	0.26	-0.55	0.24	0	14.74
162	Bupleuri Radix	MOL000422	Kaempferol	286.25	1.77	4	6	41.88	0.26	-0.55	0.24	0	14.74
162	Ginseng Radix	MOL000422	Kaempferol	286.25	1.77	4	6	41.88	0.26	-0.55	0.24	0	14.74
162	Glycyrrhizae Radix et Rhizoma	MOL000422	Kaempferol	286.25	1.77	4	6	41.88	0.26	-0.55	0.24	0	14.74
163	Astragali Radix	MOL000423	Rhamnocitrin-3-O-glucoside	462.44	-0.07	6	11	2.87	-1.34	-1.97	0.76	0	
164	Astragali Radix	MOL000424	alpha-L-Rhamnoside	164.18	-1.62	4	5	50.5	-1.21	-3.21	0.04	0	11.27
165	Astragali Radix	MOL000425	Asernestioside A	931.25	-1.21	11	18	11.07	-3.03	-4.31	0.03	0	
166	Astragali Radix	MOL000426	Asernestioside A_qt	769.09	0.54	8	13	24.55	-1.95	-2.98	0.1	0	
167	Astragali Radix	MOL000427	Asernestioside B	973.29	-0.83	10	19	12.54	-2.86	-4.41	0.03	0	
168	Astragali Radix	MOL000428	Asernestioside B_qt	811.13	0.92	7	14	14.03	-1.81	-2.98	0.09	0	
169	Astragali Radix	MOL000429	Crystal VI	132.14	-1.85	5	5	83.96	-0.88	-1.15	0.02	0	11.59
170	Astragali Radix	MOL000430	Betaine	117.17	-2.04	0	2	40.92	-0.77	-1.31	0.01	0.08	10.78
171	Astragali Radix	MOL000431	Coumarin	146.15	1.9	0	2	29.17	1.2	1.3	0.04	0	
171	Bupleuri Radix	MOL000431	Coumarin	146.15	1.9	0	2	29.17	1.2	1.3	0.04	0	
172	Astragali Radix	MOL000432	Linolenic acid	278.48	5.95	1	2	45.01	1.21	0.84	0.15	0	5.54
173	Astragali Radix	MOL000433	Folic acid	441.45	0.01	7	13	68.96	-1.5	-2.59	0.71	0	24.81
174	Astragali Radix	MOL000434	Acetylastragaloside I	911.21	0.79	6	17	43.54	-2.18	-3.23	0.09	0	16.02
175	Astragali Radix	MOL000435	Acetylastragaloside I_qt	749.05	2.54	3	12	30.75	-1.07	-2.1	0.17	0	12.94
176	Astragali Radix	MOL000436	(Z)-1-(2,4-Dihydroxyphenyl)-3-(4-hydroxyphenyl)prop-2-er	256.27	2.9	3	4	87.51	0.2	-0.48	0.15	0	20.28
177	Astragali Radix	MOL000437	Hirsutrin	464.41	-0.59	8	12	1.86	-1.66	-2.31	0.77	0	
177	Bupleuri Radix	MOL000437	Hirsutrin	464.41	-0.59	8	12	1.86	-1.66	-2.31	0.77	0	
177	Glycyrrhizae Radix et Rhizoma	MOL000437	Hirsutrin	464.41	-0.59	8	12	1.86	-1.66	-2.31	0.77	0	
178	Astragali Radix	MOL000438	Isomucronulatol	302.35	3.13	2	5	67.67	0.96	0.34	0.26	0	2.9
179	Astragali Radix	MOL000439	Isomucronulatol-7,2'-di-O-glucosiole	626.67	-0.68	8	15	49.28	-2.22	-3.36	0.62	0	0.93
180	Astragali Radix	MOL000440	Isomucronulatol-7,2'-di-O-glucosiole_qt	464.51	1.22	5	10	23.42	-0.66	-1.48	0.79	0	
181	Astragali Radix	MOL000441	Lupenone	424.78	7.36	0	1	11.66	1.48	1.31	0.78	0	
182	Astragali Radix	MOL000442	1,7-Dihydroxy-3,9-dimethoxy pterocarpene	314.31	3.11	2	6	39.05	0.89	-0.04	0.48	0	7.95
183	Glycyrrhizae Radix et Rhizoma	MOL000445	8-Prenylwighteone	406.51	5.78	3	5	23.22	0.93	0	0.54	0.33	
184	Angelicae Gigantis Radix	MOL000449	Stigmasterol	412.77	7.64	1	1	43.83	1.44	1	0.76	0.22	5.57
184	Bupleuri Radix	MOL000449	Stigmasterol	412.77	7.64	1	1	43.83	1.44	1	0.76	0.22	5.57
184	Cimicifugae Rhizoma	MOL000449	Stigmasterol	412.77	7.64	1	1	43.83	1.44	1	0.76	0.22	5.57
184	Ginseng Radix	MOL000449	Stigmasterol	412.77	7.64	1	1	43.83	1.44	1	0.76	0.22	5.57
185	Bupleuri Radix	MOL000459	(3R)-Oct-1-en-3-ol	128.24	2.53	1	1	32.79	1.12	1.23	0.01	0	5.71
186	Glycyrrhizae Radix et Rhizoma	MOL000467	Castanin	298.31	2.57	1	5	23.54	0.77	-0.1	0.27	0	
187	Bupleuri Radix	MOL000474	(-)-Epoxycaryophyllene	220.39	3.52	0	1	35.94	1.57	1.83	0.13	0	6.09
188	Cimicifugae Rhizoma	MOL000475	Anethole	148.22	2.77	0	1	32.49	1.75	1.81	0.03	0	1.68
188	Glycyrrhizae Radix et Rhizoma	MOL000475	Anethole	148.22	2.77	0	1	32.49	1.75	1.81	0.03	0	1.68
189	Angelicae Gigantis Radix	MOL000478	Eucarvone	150.24	2.07	0	1	53.14	1.35	1.65	0.03	0	11.45
190	Angelicae Gigantis Radix	MOL000479	Farnesene	204.39	5.52	0	0	17.42	1.95	2.21	0.05	0	
190	Bupleuri Radix	MOL000479	Farnesene	204.39	5.52	0	0	17.42	1.95	2.21	0.05	0	
191	Cimicifugae Rhizoma	MOL000483	Cis-N-Feruloyltyramine	313.38	2.86	3	5	118.35	0.51	-0.27	0.26	0	4.26
192	Glycyrrhizae Radix et Rhizoma	MOL000486	Prunetin	284.28	2.32	2	5	5.41	0.65	-0.25	0.24	0	
193	Angelicae Gigantis Radix	MOL000489	(1S,4aR,8aR)-1-Isopropyl-7-methyl-4-methylene-2,3,4a,5,6,8	204.39	4.8	0	0	19.8	1.86	2.03	0.08	0	
194	Bupleuri Radix	MOL000490	Petunidin	317.29	1.65	5	7	30.05	0.16	-0.64	0.31	0	1.21
195	Glycyrrhizae Radix et Rhizoma	MOL000497	Licochalcone a	338.43	4.62	2	4	40.79	0.82	-0.21	0.29	0	16.2
196	Glycyrrhizae Radix et Rhizoma	MOL000500	Vestitol	272.32	3.15	2	4	74.66	0.86	0.3	0.21	0	3
197	Glycyrrhizae Radix et Rhizoma	MOL000511	Ursolic acid	456.78	6.47	2	3	16.77	0.67	0.07	0.75	0.26	
198	Glycyrrhizae Radix et Rhizoma	MOL000561	Astragalin	448.41	-0.32	7	11	14.03	-1.34	-1.97	0.74	0.34	
199	Citri Unshius Pericarpium	MOL000597	Neryl acetate	196.32	3.31	0	2	57.47	1.25	1.4	0.04	0.27	7.92
200	Bupleuri Radix	MOL000601	O-Methylthymol	164.27	3.49	0	1	47.65	1.75	1.87	0.04	0.28	11.49
201	Bupleuri Radix	MOL000607	Dehydro-p-cymene	132.22	3.31	0	0	11.63	1.88	1.93	0.02	0.44	
202	Bupleuri Radix	MOL000608	()-Terpinen-4-ol	154.28	2.55	1	1	81.41	1.36	1.66	0.03	0.25	10.81
202	Citri Unshius Pericarpium	MOL000608	Terpinen-4-ol	154.28	2.55	1	1	81.41	1.36	1.66	0.03	0.25	10.81
203	Bupleuri Radix	MOL000609	(1R,5S)-7,7-Dimethyl-4-bicyclo[3.1.1]hept-3-enecarboxalde	150.24	2.04	0	1	40.64	1.34	1.67	0.06	0.28	11.44
204	Bupleuri Radix	MOL000610	Tridecane	184.41	6.3	0	0	17.89	1.78	1.96	0.03	0.19	
205	Bupleuri Radix	MOL000615	delta-Amorphene	204.39	4.94	0	0	17.95	1.85	1.99	0.08	0.24	
205	Citri Unshius Pericarpium	MOL000615	delta-Amorphene	204.39	4.94	0	0	17.95	1.85	1.99	0.08	0.24	
206	Bupleuri Radix	MOL000628	Darutoside	574.93	5.89	4	6	21.32	-0.26	-0.96	0.63	0.22	
206	Ginseng Radix	MOL000628	Darutoside	574.93	5.89	4	6	21.32	-0.26	-0.96	0.63	0.22	
207	Angelicae Gigantis Radix	MOL000635	Vanillin	152.16	1.31	1	3	52	0.68	0.41	0.03	0.33	11.79
207	Bupleuri Radix	MOL000635	Vanillin	152.16	1.31	1	3	52	0.68	0.41	0.03	0.33	11.79
207	Citri Unshius Pericarpium	MOL000635	Vanillin	152.16	1.31	1	3	52	0.68	0.41	0.03	0.33	11.79
208	Bupleuri Radix	MOL000666	Hexanal	100.18	1.85	0	1	55.71	1.25	1.52	0.01	0.23	10.96
208	Cimicifugae Rhizoma	MOL000666	Hexanal	100.18	1.85	0	1	55.71	1.25	1.52	0.01	0.23	10.96
209	Bupleuri Radix	MOL000667	1-Hexanol	102.2	1.88	1	1	22.04	1.08	1.21	0.01	0.19	
210	Bupleuri Radix	MOL000668	Penylfuran	138.23	3.12	0	1	54.59	1.72	1.94	0.02	0.1	-1.96
210	Cimicifugae Rhizoma	MOL000668	Penylfuran	138.23	3.12	0	1	54.59	1.72	1.94	0.02	0.1	-1.96
210	Glycyrrhizae Radix et Rhizoma	MOL000668	Penylfuran	138.23	3.12	0	1	54.59	1.72	1.94	0.02	0.1	-1.96
211	Glycyrrhizae Radix et Rhizoma	MOL000671	()-Menthol	156.3	2.78	1	1	59.33	1.27	1.42	0.03	0.22	11.03
212	Bupleuri Radix	MOL000675	Oleic acid	282.52	6.84	1	2	33.13	1.17	0.78	0.14	0.2	4.99
213	Bupleuri Radix	MOL000676	Dibutyl benzene-1,2-dicarboxylate	278.38	4.2	0	4	64.54	0.8	0.56	0.13	0.34	5.41
213	Ginseng Radix	MOL000676	Dibutyl benzene-1,2-dicarboxylate	278.38	4.2	0	4	64.54	0.8	0.56	0.13	0.34	5.41
213	Glycyrrhizae Radix et Rhizoma	MOL000676	Dibutyl benzene-1,2-dicarboxylate	278.38	4.2	0	4	64.54	0.8	0.56	0.13	0.34	5.41
214	Citri Unshius Pericarpium	MOL000696	beta-Terpineol	154.28	2.47	1	1	47.89	1.29	1.48	0.03	0.28	11.22
215	Cimicifugae Rhizoma	MOL000700	Nerol	154.28	2.93	1	1	35.66	1.15	1.14	0.02	0.29	6.75
216	Bupleuri Radix	MOL000703	2-Heptanone	114.21	1.79	0	1	46.56	1.31	1.57	0.01	0.25	10.58
216	Glycyrrhizae Radix et Rhizoma	MOL000703	2-Heptanone	114.21	1.79	0	1	46.56	1.31	1.57	0.01	0.25	10.58
217	Angelicae Gigantis Radix	MOL000705	Heptanal	114.21	2.31	0	1	19.59	1.29	1.49	0.01	0.23	
217	Bupleuri Radix	MOL000705	Heptanal	114.21	2.31	0	1	19.59	1.29	1.49	0.01	0.23	
217	Glycyrrhizae Radix et Rhizoma	MOL000705	Heptanal	114.21	2.31	0	1	19.59	1.29	1.49	0.01	0.23	
218	Bupleuri Radix	MOL000707	Heptenal	112.19	2.29	0	1	37.16	1.3	1.56	0.01	0.3	3.96
219	Bupleuri Radix	MOL000708	Benzadehyde	106.13	1.59	0	1	32.63	1.32	1.66	0.01	0.44	12.07
219	Cimicifugae Rhizoma	MOL000708	Benzadehyde	106.13	1.59	0	1	32.63	1.32	1.66	0.01	0.44	12.07
220	Bupleuri Radix	MOL000709	(S)-Matsutake alcohol	128.24	2.53	1	1	40.11	1.19	1.35	0.01	0.28	5.62
221	Bupleuri Radix	MOL000710	Octanal	128.24	2.77	0	1	19.07	1.3	1.52	0.01	0.23	
221	Citri Unshius Pericarpium	MOL000710	Octanal	128.24	2.77	0	1	19.07	1.3	1.52	0.01	0.23	
222	Bupleuri Radix	MOL000711	2,4-Heptdienal	110.17	1.84	0	1	19.14	1.29	1.54	0.01	0.38	
223	Bupleuri Radix	MOL000713	(E)-Oct-3-en-2-one	126.22	2.23	0	1	30.5	1.34	1.56	0.01	0.31	1.12
224	Bupleuri Radix	MOL000714	Hyacinthin	120.16	1.52	0	1	38.65	1.31	1.58	0.02	0.43	-2.04
224	Cimicifugae Rhizoma	MOL000714	Hyacinthin	120.16	1.52	0	1	38.65	1.31	1.58	0.02	0.43	-2.04
225	Bupleuri Radix	MOL000715	l-Menthone	154.28	2.6	0	1	57.9	1.35	1.7	0.03	0.24	10.57
225	Cimicifugae Rhizoma	MOL000715	l-Menthone	154.28	2.6	0	1	57.9	1.35	1.7	0.03	0.24	10.57
226	Bupleuri Radix	MOL000716	trans-2-Nonenal	140.25	3.2	0	1	19.18	1.37	1.59	0.02	0.27	

226	Cimicifugae Rhizoma	MOL000716	trans-2-Nonenal	140.25	3.2	0	1	19.18	1.37	1.59	0.02	0.27	
227	Bupleuri Radix	MOL000721	Nonadienal	138.23	2.75	0	1	19.03	1.38	1.56	0.02	0.33	
227	Cimicifugae Rhizoma	MOL000721	Nonadienal	138.23	2.75	0	1	19.03	1.38	1.56	0.02	0.33	
228	Bupleuri Radix	MOL000723	trans-2,4-Decadienal	152.26	3.21	0	1	51.03	1.4	1.49	0.02	0.32	7.44
228	Cimicifugae Rhizoma	MOL000723	trans-2,4-Decadienal	152.26	3.21	0	1	51.03	1.4	1.49	0.02	0.32	7.44
229	Bupleuri Radix	MOL000724	Geranylacetone	194.35	3.62	0	1	18.66	1.5	1.55	0.04	0.29	
229	Cimicifugae Rhizoma	MOL000724	Geranylacetone	194.35	3.62	0	1	18.66	1.5	1.55	0.04	0.29	
230	Citri Unshius Pericarpium	MOL000748	5-(Hydroxymethyl)furan-2-carbaldehyde	126.12	0.67	1	3	45.07	0.05	-0.27	0.02	0.22	11.73
231	Bupleuri Radix	MOL000749	Linoleic acid	280.5	6.39	1	2	41.9	1.23	0.81	0.14	0.23	5.27
231	Ginseng Radix	MOL000749	Linoleic acid	280.5	6.39	1	2	41.9	1.23	0.81	0.14	0.23	5.27
232	Citri Unshius Pericarpium	MOL000771	p-Coumaric acid	164.17	1.64	2	3	43.29	0.46	0.13	0.04	0.45	4.43
233	Ginseng Radix	MOL000787	Fumarine	353.4	2.95	0	6	59.26	0.56	-0.13	0.83	0.3	23.46
234	Bupleuri Radix	MOL000860	Stearic acid	284.54	7.28	1	2	17.83	1.15	1.22	0.14	0.19	
234	Citri Unshius Pericarpium	MOL000860	Stearic acid	284.54	7.28	1	2	17.83	1.15	1.22	0.14	0.19	
235	Bupleuri Radix	MOL000864	Pentadecane	212.47	7.22	0	0	13.98	1.81	1.92	0.05	0.15	
235	Cimicifugae Rhizoma	MOL000864	Pentadecane	212.47	7.22	0	0	13.98	1.81	1.92	0.05	0.15	
235	Ginseng Radix	MOL000864	Pentadecane	212.47	7.22	0	0	13.98	1.81	1.92	0.05	0.15	
236	Bupleuri Radix	MOL000867	Heptadecane	240.53	8.13	0	0	8.64	1.84	1.87	0.07	0.14	
237	Bupleuri Radix	MOL000868	Icosane	282.62	9.5	0	0	8.46	1.83	1.8	0.13	0.13	
238	Bupleuri Radix	MOL000869	Henicosane	296.65	9.95	0	0	8.41	1.84	1.8	0.15	0.13	
238	Citri Unshius Pericarpium	MOL000869	Henicosane	296.65	9.95	0	0	8.41	1.84	1.8	0.15	0.13	
239	Bupleuri Radix	MOL000872	(6R)-2,6-Dimethyloctane	142.32	4.53	0	0	18.15	1.79	2.06	0.01	0.22	
240	Bupleuri Radix	MOL000873	Cyclohexanone	98.16	1.17	0	1	74.99	1.21	1.77	0.01	0.22	11.11
241	Bupleuri Radix	MOL000874	Paeonol	166.19	1.29	1	3	28.79	0.93	0.84	0.04	0.32	
241	Cimicifugae Rhizoma	MOL000874	Paeonol	166.19	1.29	1	3	28.79	0.93	0.84	0.04	0.32	
241	Ginseng Radix	MOL000874	Paeonol	166.19	1.29	1	3	28.79	0.93	0.84	0.04	0.32	
242	Bupleuri Radix	MOL000878	Farnesylacetone	262.48	5.45	0	1	37.84	1.61	1.56	0.1	0.29	5.22
243	Cimicifugae Rhizoma	MOL000879	Methyl palmitate	270.51	6.62	0	2	18.09	1.37	1.18	0.12	0.14	
243	Ginseng Radix	MOL000879	Methyl palmitate	270.51	6.62	0	2	18.09	1.37	1.18	0.12	0.14	
244	Cimicifugae Rhizoma	MOL000880	Tricosane	324.71	10.86	0	0	8.33	1.85	1.68	0.21	0.13	
245	Angelicae Gigantis Radix	MOL000885	Dodecane	170.38	5.85	0	0	17.74	1.79	1.96	0.02	0.16	
246	Bupleuri Radix	MOL000886	Tetradecane	198.44	6.76	0	0	15.94	1.79	1.78	0.04	0.15	
246	Ginseng Radix	MOL000886	Tetradecane	198.44	6.76	0	0	15.94	1.79	1.78	0.04	0.15	
247	Ginseng Radix	MOL000908	beta-Elemene	204.39	4.79	0	0	25.63	1.84	2.07	0.06	0.33	
248	Bupleuri Radix	MOL000918	2-Nonanone	142.27	2.7	0	1	8.51	1.34	1.62	0.02	0.24	
249	Bupleuri Radix	MOL000922	(R)-p-Menth-1-en-4-ol	154.28	2.55	1	1	32.16	1.33	1.52	0.03	0.26	11.39
249	Citri Unshius Pericarpium	MOL000922	(R)-p-Menth-1-en-4-ol	154.28	2.55	1	1	32.16	1.33	1.52	0.03	0.26	11.39
250	Bupleuri Radix	MOL000924	Undecan-2-one	170.33	3.62	0	1	17.66	1.4	1.64	0.03	0.22	
251	Bupleuri Radix	MOL000932	alpha-Farnesene	204.39	5.46	0	0	21.7	1.97	1.89	0.05	0.32	
252	Ginseng Radix	MOL000935	Hepanal	204.39	4.36	0	0	53.83	1.86	2.17	0.1	0.24	11.92
253	Bupleuri Radix	MOL000937	Isoledene	204.39	4.36	0	0	49.01	1.82	2.08	0.1	0.22	12.47
254	Ginseng Radix	MOL000942	trans-Cadinol	222.41	3.78	1	1	31.67	1.32	1.3	0.09	0.24	6.87
255	Angelicae Gigantis Radix	MOL000967	Bergamotene	204.39	4.75	0	0	28.51	1.86	2.06	0.09	0.29	
256	Ginseng Radix	MOL000968	beta-Bisabolene	204.39	5.33	0	0	29.59	1.88	2.13	0.06	0.29	
257	Angelicae Gigantis Radix	MOL000974	Cuminal	148.22	2.78	0	1	38.29	1.39	1.61	0.03	0.36	1.94
257	Bupleuri Radix	MOL000974	Cuminal	148.22	2.78	0	1	38.29	1.39	1.61	0.03	0.36	1.94
258	Cimicifugae Rhizoma	MOL000991	Cinnamaldehyde	132.17	1.95	0	1	31.99	1.35	1.48	0.02	0.48	4.73
259	Citri Unshius Pericarpium	MOL001055	(-)-alpha-Thujene	136.26	2.87	0	0	47.19	1.82	2.18	0.04	0.29	11.44
260	Glycyrrhizae Radix et Rhizoma	MOL001097	O-Xylene	106.18	2.8	0	0	45.55	1.85	2.08	0.01	0.41	11.86
261	Glycyrrhizae Radix et Rhizoma	MOL001098	m-Xylene	106.18	2.8	0	0	47.43	1.83	2.02	0.01	0.39	11.82
262	Glycyrrhizae Radix et Rhizoma	MOL001099	p-Xylene	106.18	2.8	0	0	48.74	1.83	2.06	0.01	0.39	11.71
263	Citri Unshius Pericarpium	MOL001101	alpha-Ocimene	136.26	3.69	0	0	21.43	1.87	1.99	0.02	0.38	
264	Bupleuri Radix	MOL001109	beta-Thujene	136.26	2.68	0	0	46.44	1.82	2.19	0.04	0.29	11.53
265	Citri Unshius Pericarpium	MOL001110	cis-beta-Ocimene	136.26	3.63	0	0	25.38	1.85	2.03	0.02	0.34	
266	Bupleuri Radix	MOL001121	(−)-Myrtenol	152.26	1.78	1	1	49.98	1.25	1.5	0.06	0.21	11.55
267	Bupleuri Radix	MOL001179	(-)-Alloaromadendrene	204.39	4.22	0	0	54.04	1.81	2.07	0.1	0.23	12.06
268	Bupleuri Radix	MOL001210	(S)-Phellandral	152.26	2.86	0	1	40.36	1.36	1.69	0.03	0.28	1.98
269	Angelicae Gigantis Radix	MOL001212	Loxanol V	214.44	5.53	1	1	14.19	1.27	0.98	0.05	0.14	
269	Ginseng Radix	MOL001212	Loxanol V	214.44	5.53	1	1	14.19	1.27	0.98	0.05	0.14	
270	Ginseng Radix	MOL001218	1-Hydroxydodecane	186.38	4.62	1	1	18.5	1.23	1.06	0.03	0.14	
271	Bupleuri Radix	MOL001223	(S)-2,2,3-Trimethylcyclopent-3-ene-1-acetaldehyde	152.26	2.19	0	1	45.18	1.32	1.71	0.03	0.31	11.43
272	Angelicae Gigantis Radix	MOL001224	Tridecylene	182.39	5.91	0	0	17.69	1.83	2.01	0.03	0.2	
273	Cimicifugae Rhizoma	MOL001246	(1R)-Nopinone	138.23	1.52	0	1	57.86	1.23	1.6	0.05	0.28	11.1
274	Angelicae Gigantis Radix	MOL001273	Verbenone	150.24	1.94	0	1	50.63	1.27	1.59	0.06	0.34	11.56
274	Bupleuri Radix	MOL001273	Verbenone	150.24	1.94	0	1	50.63	1.27	1.59	0.06	0.34	11.56
275	Bupleuri Radix	MOL001283	(3S)-3,7,11-Trimethyldodeca-1,6,10-trien-3-ol	222.41	4.56	1	1	29.56	1.37	1.34	0.06	0.31	
276	Bupleuri Radix	MOL001285	Octanol	130.26	2.8	1	1	21.06	1.16	1.23	0.01	0.17	
277	Cimicifugae Rhizoma	MOL001300	beta-Hydroxyethylbenzene	122.18	1.55	1	1	44.03	1.11	1.13	0.02	0.37	-2.41
278	Angelicae Gigantis Radix	MOL001302	Decanedioic acid	202.28	2.33	2	4	16.23	-0.01	-0.64	0.05	0.25	
279	Bupleuri Radix	MOL001303	(E)-β-Elemene	204.39	4.79	0	0	5.58	1.85	2.11	0.06	0.34	
280	Cimicifugae Rhizoma	MOL001304	2-Acetylpyrrole	109.14	0.96	1	1	58.37	1.16	1.42	0.01	0.12	17.58
281	Angelicae Gigantis Radix	MOL001306	o-Acetyl-p-cresol	150.19	1.79	1	2	24.96	1.02	0.88	0.03	0.39	
282	Bupleuri Radix	MOL001312	9-Hexadecenoic acid	254.46	5.92	1	2	35.78	1.1	0.85	0.1	0.2	5.61
282	Ginseng Radix	MOL001312	9-Hexadecenoic acid	254.46	5.92	1	2	35.78	1.1	0.85	0.1	0.2	5.61
283	Angelicae Gigantis Radix	MOL001314	Azelex	188.25	1.87	2	4	16.9	-0.04	-0.72	0.04	0.26	
284	Cimicifugae Rhizoma	MOL001335	Phenylmethanol	108.15	1.23	1	1	58.68	1.08	1.27	0.01	0.38	11.87
285	Cimicifugae Rhizoma	MOL001382	Methylheptadienone	124.2	1.77	0	1	24.05	1.35	1.5	0.01	0.42	
286	Bupleuri Radix	MOL001386	Methyl laurate	214.39	4.79	0	2	21.75	1.32	1.24	0.05	0.15	
287	Angelicae Gigantis Radix	MOL001388	(+)-Ledol	222.41	3.2	1	1	16.96	1.43	1.69	0.12	0.21	
288	Bupleuri Radix	MOL001392	Methyl myristate	242.45	5.71	0	2	19.68	1.36	1.19	0.08	0.14	
288	Ginseng Radix	MOL001392	Methyl myristate	242.45	5.71	0	2	19.68	1.36	1.19	0.08	0.14	
289	Bupleuri Radix	MOL001393	Myristic acid	228.42	5.46	1	2	21.18	1.07	0.99	0.07	0.19	
289	Cimicifugae Rhizoma	MOL001393	Myristic acid	228.42	5.46	1	2	21.18	1.07	0.99	0.07	0.19	
290	Bupleuri Radix	MOL001394	Octadecan	254.56	8.58	0	0	9.81	1.83	1.83	0.09	0.14	
291	Bupleuri Radix	MOL001396	Pentadecylic acid	242.45	5.91	1	2	20.18	1.08	0.88	0.08	0.18	
291	Cimicifugae Rhizoma	MOL001396	Pentadecylic acid	242.45	5.91	1	2	20.18	1.08	0.88	0.08	0.18	
291	Ginseng Radix	MOL001396	Pentadecylic acid	242.45	5.91	1	2	20.18	1.08	0.88	0.08	0.18	
292	Bupleuri Radix	MOL001397	cis-Hept-2-enal	112.19	2.29	0	1	40.19	1.29	1.64	0.01	0.31	4.03
293	Cimicifugae Rhizoma	MOL001401	Hexacosane	366.8	12.23	0	0	8.21	1.92	1.65	0.31	0.12	
294	Bupleuri Radix	MOL001402	Octacosane	394.86	13.15	0	0	8.15	1.91	1.46	0.37	0.12	
295	Bupleuri Radix	MOL001417	Octenal	126.22	2.74	0	1	19.41	1.34	1.59	0.01	0.28	
295	Cimicifugae Rhizoma	MOL001417	Octenal	126.22	2.74	0	1	19.41	1.34	1.59	0.01	0.28	
296	Glycyrrhizae Radix et Rhizoma	MOL001484	Inermine	284.28	2.44	1	5	75.18	0.89	0.4	0.54	0.3	11.72
297	Bupleuri Radix	MOL001487	Fitone	268.54	6.2	0	1	6.67	1.5	1.44	0.1	0.22	
297	Cimicifugae Rhizoma	MOL001487	Fitone	268.54	6.2	0	1	6.67	1.5	1.44	0.1	0.22	
298	Glycyrrhizae Radix et Rhizoma	MOL001543	Vicenin-2	594.57	-2.45	11	15	3.42	-3.15	-3.87	0.78	0.32	
299	Angelicae Gigantis Radix	MOL001578	Hypnon	120.16	1.57	0	1	48.19	1.36	1.54	0.02	0.45	25.2
299	Bupleuri Radix	MOL001578	Hypnon	120.16	1.57	0	1	48.19	1.36	1.54	0.02	0.45	25.2
300	Glycyrrhizae Radix et Rhizoma	MOL001599	alpha-Cubebol	208.38	3.28	1	1	64.81	1.32	1.43	0.09	0.26	7.24
301	Bupleuri Radix	MOL001600	Copaene	204.39	4.17	0	0	29.47	1.81	2.04	0.12	0.22	
302	Bupleuri Radix	MOL001606	(3R,4aS,5R)-4a,5-Dimethyl-3-prop-1-en-2-yl-2,3,4,5,6,7-hex	204.39	4.75	0	0	35.57	1.83	2.02	0.08	0.27	7.23
303	Bupleuri Radix	MOL001619	n-Nonadecane	268.59	9.04	0	0	8.52	1.83	1.7	0.11	0.16	
304	Angelicae Gigantis Radix	MOL001640	Decanoic acid	172.3	3.63	1	2	26.74	0.96	1.06	0.03	0.22	
305	Ginseng Radix	MOL001641	Methyl linoleate	294.53	6.64	0	2	41.93	1.44	1.08	0.17	0.21	6.05
306	Cimicifugae Rhizoma	MOL001644	Dodecanal	184.36	4.59	0	1	21.52	1.4	1.42	0.03	0.19	
307	Bupleuri Radix	MOL001645	Linoleyl acetate	308.56	6.85	0	2	42.1	1.36	1.08	0.2	0.21	7.48
308	Glycyrrhizae Radix et Rhizoma	MOL001696	Morusin	420.49	4.94	3	6	11.52	0.51	-0.22	0.76	0.31	
309	Ginseng Radix	MOL001706	2,6-Dimethyl-3,7-octadiene-2,6-diol	170.28	1.33	2	2	52.4	0.47	0.51	0.03	0.33	6.57
310	Bupleuri Radix	MOL001731	Dextrin Corn	180.18	-2.51	5	6	50.38	-1.93	-4.47	0.04	0.27	11.12
311	Bupleuri Radix	MOL001732	Glycerol	92.11	-1.41	3	3	72.87	-0.97	-2.63	0.01	0.2	11.45
312	Glycyrrhizae Radix et Rhizoma	MOL001737	1H-Indole-3-carboxylic acid	161.17	1.73	2	2	33.86	0.82	0.63	0.05	0.3	-7.7
313	Ginseng Radix	MOL001738	Malonic acid	104.07	-0.44	2	4	21.69	-0.23	-0.25	0.01	0.44	
314	Bupleuri Radix	MOL001752	2-Methoxy-4-vinylphenol	150.19	2.1	1	2	38.39	1.36	1.35	0.03	0.36	11.57

315	Cimicifugae Rhizoma	MOL001773	Indole	117.16	2.12	1	0	34.38	1.81	2.07	0.03	0.2	5.56
316	Angelicae Gigantis Radix	MOL001788	Adenine	135.15	-0.58	3	4	62.81	-0.3	-0.63	0.03	0	13.33
317	Bupleuri Radix	MOL001789	Isoliquiritigenin	256.27	2.9	3	4	85.32	0.44	-0.41	0.15	0.46	17.66
317	Glycyrrhizae Radix et Rhizoma	MOL001789	Isoliquiritigenin	256.27	2.9	3	4	85.32	0.44	-0.41	0.15	0.46	17.66
318	Glycyrrhizae Radix et Rhizoma	MOL001792	4',7-Dihydroxyflavanone	256.27	2.57	2	4	32.76	0.51	-0.29	0.18	0.42	17.89
319	Citri Unshius Pericarpium	MOL001797	Neohesperdin	610.62	-0.48	8	15	11.17	-2.17	-3.01	0.7	0	
320	Bupleuri Radix	MOL001816	Palmitamide	255.5	5.77	2	2	19.79	1.22	1.3	0.1	0.17	
321	Bupleuri Radix	MOL001817	Methyl stearate	298.57	7.53	0	2	16.8	1.41	1.29	0.16	0.16	
321	Ginseng Radix	MOL001817	Methyl stearate	298.57	7.53	0	2	16.8	1.41	1.29	0.16	0.16	
322	Ginseng Radix	MOL001818	Methyl palmitelaidate	268.49	6.17	0	2	34.61	1.4	1.22	0.12	0.17	5.58
323	Bupleuri Radix	MOL001819	Methyl pentadecanoate	256.48	6.16	0	2	18.82	1.37	1.28	0.1	0.17	
323	Ginseng Radix	MOL001819	Methyl pentadecanoate	256.48	6.16	0	2	18.82	1.37	1.28	0.1	0.17	
324	Glycyrrhizae Radix et Rhizoma	MOL001850	Izoforon	138.23	2.06	0	1	44.98	1.28	1.66	0.03	0.32	11.4
325	Ginseng Radix	MOL001949	Panaxynol	244.41	5.68	1	1	42.44	1.52	1.03	0.1	0.32	7.14
326	Astragali Radix	MOL001955	Heriguard	354.34	-0.42	6	9	11.93	-1.03	-1.71	0.33	0.37	
327	Ginseng Radix	MOL001965	Dauricine (8CI)	624.84	7.22	1	8	23.65	0.9	0.03	0.37	0.22	
328	Bupleuri Radix	MOL001972	Pulegone	152.26	2.75	0	1	51.6	1.39	1.74	0.03	0.26	11.19
328	Cimicifugae Rhizoma	MOL001972	Pulegone	152.26	2.75	0	1	51.6	1.39	1.74	0.03	0.26	11.19
329	Bupleuri Radix	MOL001999	Scoparone	206.21	1.87	0	4	74.75	0.85	0.46	0.09	0.23	0.73
330	Bupleuri Radix	MOL002002	cis-Carveol	152.26	2.4	1	1	45.61	1.39	1.62	0.03	0.27	11.73
331	Angelicae Gigantis Radix	MOL002029	Cuparene	202.37	4.72	0	0	38.26	1.88	2.15	0.07	0.3	-2.24
331	Bupleuri Radix	MOL002029	Cuparene	202.37	4.72	0	0	38.26	1.88	2.15	0.07	0.3	-2.24
331	Citri Unshius Pericarpium	MOL002029	Cuparene	202.37	4.72	0	0	38.26	1.88	2.15	0.07	0.3	-2.24
332	Angelicae Gigantis Radix	MOL002033	cis-Thujopsene	204.39	4.08	0	0	56.43	1.84	2.24	0.12	0.25	-1.45
333	Bupleuri Radix	MOL002042	Thymol	150.24	3.24	1	1	41.47	1.6	1.68	0.03	0.33	11.33
334	Bupleuri Radix	MOL002046	Hexanoic acid	116.18	1.81	1	2	73.08	0.8	0.93	0.01	0.27	10.81
334	Cimicifugae Rhizoma	MOL002046	Hexanoic acid	116.18	1.81	1	2	73.08	0.8	0.93	0.01	0.27	10.81
335	Citri Unshius Pericarpium	MOL002050	Isovanillic acid	168.16	1.15	2	4	39.42	0.47	0.38	0.04	0.35	2.23
336	Bupleuri Radix	MOL002085	alpha-Cubebene	204.39	4.17	0	0	16.73	1.83	2.1	0.11	0.25	
337	Citri Unshius Pericarpium	MOL002092	Antioxidant No. 33	206.36	4.36	1	1	26.74	1.68	1.81	0.06	0.28	
338	Bupleuri Radix	MOL002095	Ethyl phthalate	222.26	2.24	0	4	52.19	0.72	0.57	0.07	0.38	5.37
338	Citri Unshius Pericarpium	MOL002095	Ethyl phthalate	222.26	2.24	0	4	52.19	0.72	0.57	0.07	0.38	5.37
339	Angelicae Gigantis Radix	MOL002098	3-Butylidene-7-hydroxyphthalide	204.24	2.74	1	3	62.68	1	0.9	0.08	0.37	4.66
340	Angelicae Gigantis Radix	MOL002102	Levistolid A	380.52	4.97	0	4	2.15	0.94	0.45	0.82	0.27	
341	Bupleuri Radix	MOL002107	Valerophenone	162.25	3.15	0	1	42.58	1.46	1.53	0.03	0.35	19.74
342	Angelicae Gigantis Radix	MOL002110	Allocymene	136.26	3.58	0	0	14.89	1.85	1.91	0.02	0.38	
343	Angelicae Gigantis Radix	MOL002111	3-Butylidene phthalide	188.24	3	0	2	42.44	1.32	1.27	0.07	0.39	5.64
343	Bupleuri Radix	MOL002111	3-Butylidene phthalide	188.24	3	0	2	42.44	1.32	1.27	0.07	0.39	5.64
344	Bupleuri Radix	MOL002116	(1R,4S,5R)-4-Isopropenyl-1,8-dimethylspiro[4.5]dec-8-ene	204.39	4.75	0	0	40.01	1.85	2.11	0.07	0.29	-0.49
345	Ginseng Radix	MOL002121	(+)-Bicyclogermacrene	204.39	4.7	0	0	21.69	1.86	2.02	0.08	0.26	
346	Cimicifugae Rhizoma	MOL002122	(Z)-Ligustilide	188.24	3	0	2	53.72	1.3	1.25	0.07	0.38	5.61
347	Ginseng Radix	MOL002136	Neocnidilide	194.3	3.37	0	2	83.83	1.23	1.32	0.07	0.28	5.38
348	Ginseng Radix	MOL002137	Octane	114.26	4.02	0	0	29.72	1.78	2.02	0.01	0.19	
348	Glycyrrhizae Radix et Rhizoma	MOL002137	Octane	114.26	4.02	0	0	29.72	1.78	2.02	0.01	0.19	
349	Bupleuri Radix	MOL002138	p-Cymen-8-ol	150.24	2.29	1	1	32.26	1.33	1.35	0.03	0.35	11.04
349	Cimicifugae Rhizoma	MOL002138	p-Cymen-8-ol	150.24	2.29	1	1	32.26	1.33	1.35	0.03	0.35	11.04
349	Citri Unshius Pericarpium	MOL002138	p-Cymen-8-ol	150.24	2.29	1	1	32.26	1.33	1.35	0.03	0.35	11.04
350	Bupleuri Radix	MOL002141	3beta-Hydroxypregn-5-ene-20-one	316.53	3.49	1	2	14.07	0.69	0.24	0.43	0.25	
351	Angelicae Gigantis Radix	MOL002143	Senkyunolide-C	204.24	2.74	1	3	46.8	0.87	0.5	0.08	0.39	5.8
352	Angelicae Gigantis Radix	MOL002144	Senkyunolide-D	222.26	1.8	1	4	79.13	0.12	-0.07	0.1	0.37	5.37
353	Angelicae Gigantis Radix	MOL002145	Senkyunolide-E	204.24	1.9	1	3	34.4	0.55	0.06	0.08	0.39	7.18
354	Glycyrrhizae Radix et Rhizoma	MOL002166	Isoheptane	100.23	3.36	0	0	59.94	1.81	2.25	0.01	0.22	10.67
355	Angelicae Gigantis Radix	MOL002180	4-Octanone	128.24	2.46	0	1	19.37	1.37	1.55	0.01	0.22	
356	Angelicae Gigantis Radix	MOL002184	(6R)-6-Butylcyclohepta-1,4-diene	150.29	3.93	0	0	31.69	1.85	2.18	0.02	0.25	5.7
357	Bupleuri Radix	MOL002187	Amylbenzene	148.27	4.14	0	0	34.34	1.88	2.03	0.03	0.31	6.28
358	Glycyrrhizae Radix et Rhizoma	MOL002198	Heptan	100.23	3.57	0	0	41.8	1.77	2.04	0	0.2	10.36
359	Angelicae Gigantis Radix	MOL002201	cis-Ligustilide	190.26	2.94	0	2	51.3	1.3	1.24	0.07	0.34	5.53
359	Bupleuri Radix	MOL002201	cis-Ligustilide	190.26	2.94	0	2	51.3	1.3	1.24	0.07	0.34	5.53
360	Bupleuri Radix	MOL002307	20-Hexadecanoylingenol	586.94	7.38	3	6	28.2	0.3	-0.04	0.68	0.24	
360	Ginseng Radix	MOL002307	20-Hexadecanoylingenol	586.94	7.38	3	6	28.2	0.3	-0.04	0.68	0.24	
361	Glycyrrhizae Radix et Rhizoma	MOL002311	Glycyrol	366.39	4.85	2	6	90.78	0.71	-0.2	0.67	0.28	9.85
362	Ginseng Radix	MOL002312	[(3S,4R,5R)-5-[[(2R,3S,4S,5R,6S)-6-(2-Acetyl-5-methoxypher	612.59	-0.79	8	16	5.61	-2.17	-3.07	0.63	0.34	
363	Ginseng Radix	MOL002323	L-Adenosine	267.28	-2.02	5	8	18.06	-1.42	-1.99	0.18	0.22	
364	Bupleuri Radix	MOL002335	beta-Gurjunene	204.39	4.22	0	0	51.36	1.81	2.07	0.1	0.26	12.07
365	Citri Unshius Pericarpium	MOL002336	Farnesane	212.47	6.6	0	0	3.13	1.82	1.97	0.04	0.21	
366	Bupleuri Radix	MOL002338	(1aR,4aS,7R,7aR,7bR)-1,1,7-Trimethyl-4-methylidenedecahy	220.39	3.01	1	1	80.86	1.42	1.62	0.12	0.26	12.34
367	Bupleuri Radix	MOL002365	(s)-Carvone	150.24	2.36	0	1	47.43	1.34	1.62	0.03	0.33	11.8
368	Bupleuri Radix	MOL002375	(-)-Isomenthone	154.28	2.6	0	1	61.19	1.36	1.78	0.03	0.24	10.86
369	Cimicifugae Rhizoma	MOL002376	Pentacosane	352.77	11.78	0	0	8.25	1.9	1.85	0.27	0.14	
370	Ginseng Radix	MOL002377	Kaempferol-3-arabofuranoside	418.38	0.19	6	10	2.73	-1.08	-2.01	0.65	0.34	
371	Bupleuri Radix	MOL002378	Undecane	156.35	5.39	0	0	17.15	1.79	2.02	0.02	0.18	
372	Bupleuri Radix	MOL002379	Pentanal	86.15	1.4	0	1	59.53	1.21	1.52	0	0	11.32
373	Citri Unshius Pericarpium	MOL002456	[(3R)-3,7-Dimethyloct-6-enyl] butanoate	226.4	4.55	0	2	21.03	1.33	1.31	0.06	0.25	
374	Angelicae Gigantis Radix	MOL002480	Methylbutenol	86.15	0.84	1	1	54.58	1.12	1.32	0.01	0.33	11.47
375	Bupleuri Radix	MOL002484	Isovaleral	86.15	1.19	0	1	44.71	1.2	1.6	0	0.27	11.36
376	Cimicifugae Rhizoma	MOL002496	Sulcatone	126.22	1.79	0	1	26.36	1.35	1.52	0.01	0.31	
377	Ginseng Radix	MOL002526	delta-Guaiene	204.39	4.99	0	0	23.66	1.86	2.05	0.07	0.25	
378	Glycyrrhizae Radix et Rhizoma	MOL002547	(1S,3R)-cis-4-Carene	136.26	2.68	0	0	40.92	1.84	2.14	0.04	0.26	11.22
379	Glycyrrhizae Radix et Rhizoma	MOL002565	Medicarpin	270.3	2.66	1	4	49.22	1	0.53	0.34	0.31	8.46
380	Bupleuri Radix	MOL002579	Capsaicin	305.46	3.89	2	4	10.31	0.93	0.43	0.2	0.24	
381	Ginseng Radix	MOL002669	Campesteryl ferulate	576.94	9.86	1	4	22.1	1.1	0.06	0.59	0.26	
382	Bupleuri Radix	MOL002675	Hexenal	98.16	1.83	0	1	46.01	1.29	1.66	0.01	0.31	11.37
383	Glycyrrhizae Radix et Rhizoma	MOL002678	Ethylbenzene	106.18	2.77	0	0	49.38	1.83	2.15	0.01	0.38	11.76
384	Glycyrrhizae Radix et Rhizoma	MOL002693	Nicotiflorin	594.57	-1.18	9	15	3.64	-1.77	-2.55	0.73	0.3	
385	Bupleuri Radix	MOL002702	Nonacosanol	424.89	12.38	1	1	10.57	1.48	0.7	0.43	0.15	
386	Bupleuri Radix	MOL002776	Baicalin	446.39	0.64	6	11	40.12	-0.85	-1.74	0.75	0.36	17.36
387	Bupleuri Radix	MOL002786	Apocynin	166.19	1.29	1	3	31.71	0.74	0.52	0.04	0.33	24.75
388	Angelicae Gigantis Radix	MOL002830	4-Methylphenol	108.15	2.05	1	1	51.99	1.56	1.88	0.01	0.41	11.87
388	Bupleuri Radix	MOL002830	4-Methylphenol	108.15	2.05	1	1	51.99	1.56	1.88	0.01	0.41	11.87
388	Cimicifugae Rhizoma	MOL002830	4-Methylphenol	108.15	2.05	1	1	51.99	1.56	1.88	0.01	0.41	11.87
389	Glycyrrhizae Radix et Rhizoma	MOL002844	Pinocembrin	256.27	2.57	2	4	64.72	0.61	0.12	0.18	0.43	17.96
390	Glycyrrhizae Radix et Rhizoma	MOL002850	Butylated hydroxytoluene	220.39	4.85	1	1	40.02	1.75	1.8	0.07	0.3	10.36
391	Citri Unshius Pericarpium	MOL002868	1-Undecyne	152.31	5.58	0	0	33.99	1.86	2.11	0.02	0.28	6.88
392	Ginseng Radix	MOL002879	Diisooctyl 1,2-benzenedicarboxylate	390.62	7.44	0	4	43.59	0.79	0.26	0.39	0.28	3.6
393	Bupleuri Radix	MOL002885	Pentanoic acid	102.15	1.35	1	2	70.74	0.78	1.09	0.01	0.28	11.3
393	Cimicifugae Rhizoma	MOL002885	Pentanoic acid	102.15	1.35	1	2	70.74	0.78	1.09	0.01	0.28	11.3
394	Bupleuri Radix	MOL002936	5,8-Dihydroxy-6,7-dimethoxyflavone	314.31	2.57	2	6	5.74	0.97	0.09	0.29	0.28	
395	Glycyrrhizae Radix et Rhizoma	MOL002943	Butanol	74.14	0.97	1	1	22.02	0.94	1.05	0	0.21	
396	Citri Unshius Pericarpium	MOL002944	(E)-Linalol pyranoxide	170.28	1.43	1	2	44.25	0.94	0.94	0.04	0.29	11.42
397	Angelicae Gigantis Radix	MOL002972	Bisabolene	204.39	5.33	0	0	20.3	1.89	1.99	0.06	0.28	
397	Bupleuri Radix	MOL002972	Bisabolene	204.39	5.33	0	0	20.3	1.89	1.99	0.06	0.28	
398	Angelicae Gigantis Radix	MOL002983	Guasol	124.15	1.55	1	2	51.6	1.28	1.48	0.02	0.32	11.92
398	Bupleuri Radix	MOL002983	Guasol	124.15	1.55	1	2	51.6	1.28	1.48	0.02	0.32	11.92
398	Cimicifugae Rhizoma	MOL002983	Guasol	124.15	1.55	1	2	51.6	1.28	1.48	0.02	0.32	11.92
399	Angelicae Gigantis Radix	MOL002998	Phenol	94.12	1.56	1	1	36.05	1.5	1.86	0.01	0.44	12.07
399	Bupleuri Radix	MOL002998	Phenol	94.12	1.56	1	1	36.05	1.5	1.86	0.01	0.44	12.07
399	Cimicifugae Rhizoma	MOL002998	Phenol	94.12	1.56	1	1	36.05	1.5	1.86	0.01	0.44	12.07
400	Bupleuri Radix	MOL003040	Amylol	88.17	1.43	1	1	76.16	1.02	1.14	0	0.2	11.03
401	Bupleuri Radix	MOL003050	Nonanoic acid	158.27	3.17	1	2	40.51	0.92	1.08	0.02	0.23	4.15
401	Cimicifugae Rhizoma	MOL003050	Nonanoic acid	158.27	3.17	1	2	40.51	0.92	1.08	0.02	0.23	4.15
402	Bupleuri Radix	MOL003060	Hexadienal	96.14	1.39	0	1	38.55	1.25	1.57	0.01	0.42	11.68
403	Bupleuri Radix	MOL003127	Germacrene D	204.39	5.14	0	0	19.22	1.83	2.02	0.06	0	
404	Glycyrrhizae Radix et Rhizoma	MOL003218	Neouralenol	370.38	3.36	5	7	12.76	0.24	-0.75	0.46	0.37	

405	Ginseng Radix	MOL003346	Psuedohypericin	520.46	3.8	7	9	16.94	-0.2	-1.61	0.07	0.34	
406	Bupleuri Radix	MOL003367	Myricadiol	442.8	6.21	2	2	13.58	0.78	0.17	0.77	0.23	
407	Bupleuri Radix	MOL003443	2-Decenal	154.28	3.66	0	1	18.56	1.38	1.67	0.02	0.28	
408	Citri Unshius Pericarpium	MOL003450	Dodec-2-enal	182.34	4.57	0	1	31.95	1.42	1.5	0.03	0.23	7.11
409	Cimicifugae Rhizoma	MOL003484	Phenanthrene	178.24	3.65	0	0	25.7	1.88	1.79	0.1	0.52	
410	Bupleuri Radix	MOL003493	Naphthalene	128.18	2.74	0	0	27.55	1.87	1.92	0.03	0.49	
411	Bupleuri Radix	MOL003507	Heptanol	116.23	2.34	1	1	17.22	1.1	1.11	0.01	0.18	
412	Citri Unshius Pericarpium	MOL003508	1-Decanol	158.32	3.71	1	1	16.85	1.2	1.13	0.02	0.18	
413	Bupleuri Radix	MOL003509	Nonanol	144.29	3.25	1	1	33.19	1.17	1.15	0.01	0.18	6.07
414	Cimicifugae Rhizoma	MOL003520	Damascenone	190.31	3.4	0	1	36.43	1.34	1.6	0.05	0	8.2
415	Bupleuri Radix	MOL003529	m-Methylacetophenone	134.19	2.06	0	1	40.63	1.39	1.44	0.02	0.43	24.94
416	Angelicae Gigantis Radix	MOL003534	Cadinene	204.39	4.75	0	0	17.12	1.88	2.06	0.08	0.25	
417	Citri Unshius Pericarpium	MOL003538	Ledene	204.39	4.36	0	0	51.84	1.86	2.16	0.1	0.23	11.79
418	Bupleuri Radix	MOL003573	Calacorene	200.35	4.79	0	0	16.2	1.89	1.99	0.08	0	
419	Angelicae Gigantis Radix	MOL003587	Acoradiene	204.39	4.75	0	0	36.73	1.85	2.11	0.07	0.28	-0.84
419	Bupleuri Radix	MOL003587	Acoradiene	204.39	4.75	0	0	36.73	1.85	2.11	0.07	0.28	-0.84
420	Bupleuri Radix	MOL003590	Angelicin	186.17	2.2	0	3	19.6	1.05	0.86	0.1	0.26	
421	Bupleuri Radix	MOL003613	Silvan	82.11	1.08	0	1	44.65	1.69	2.07	0.01	0.06	4.36
422	Ginseng Radix	MOL003648	Inermin	284.28	2.44	1	5	65.83	0.91	0.36	0.54	0.3	11.73
423	Glycyrrhizae Radix et Rhizoma	MOL003656	Lupiwighteone	338.38	3.92	3	5	51.64	0.68	-0.23	0.37	0.36	15.63
424	Glycyrrhizae Radix et Rhizoma	MOL003662	7,4'-Dihydroxyflavone	254.25	2.6	2	4	19.18	0.56	-0.16	0.18	0.43	
425	Bupleuri Radix	MOL003686	Narcissoside	624.6	-1.19	9	16	5.09	-2.14	-2.74	0.65	0	
425	Glycyrrhizae Radix et Rhizoma	MOL003686	Narcissoside	624.6	-1.19	9	16	5.09	-2.14	-2.74	0.65	0	
426	Bupleuri Radix	MOL003786	Patchoulane	206.41	4.37	0	0	16.21	1.78	2.2	0.11	0.21	
427	Bupleuri Radix	MOL003837	Esculetin	178.15	1.37	2	4	22.97	0.44	0.02	0.07	0.36	
427	Cimicifugae Rhizoma	MOL003837	Esculetin	178.15	1.37	2	4	22.97	0.44	0.02	0.07	0.36	
428	Bupleuri Radix	MOL003839	(-)-Guaia-1(10),11-dien-15-al	218.37	4.16	0	1	29.71	1.48	1.58	0.09	0.27	
429	Ginseng Radix	MOL003845	Folinic acid	473.5	-0.04	8	14	23.6	-1.7	-2.58	0.74	0.34	
430	Glycyrrhizae Radix et Rhizoma	MOL003896	7-Methoxy-2-methyl isoflavone	266.31	3.36	0	3	42.56	1.16	0.56	0.2	0.33	16.89
431	Ginseng Radix	MOL003902	Methyl (Z)-icos-11-enoate	324.61	8	0	2	29.49	1.41	1.11	0.23	0.16	
432	Bupleuri Radix	MOL003937	(-)-gamma-Cadinene	204.39	4.8	0	0	20.21	1.87	2.06	0.08	0.26	
433	Citri Unshius Pericarpium	MOL003949	Dimethyl anthranilate	165.21	1.49	1	3	65.87	1.35	1.29	0.04	0.34	23.75
434	Glycyrrhizae Radix et Rhizoma	MOL003985	2-Caren-10-al	150.24	2.04	0	1	44.74	1.37	1.63	0.05	0.28	11.47
435	Bupleuri Radix	MOL003997	3-Furaldehyde	96.09	0.69	0	2	50.96	1.04	1.21	0.01	0.19	-3.08
436	Cimicifugae Rhizoma	MOL004029	Visnagin	230.23	2.19	0	4	44.25	1.1	0.65	0.15	0	9.5
437	Bupleuri Radix	MOL004067	Nootkatone	218.37	3.61	0	1	33.04	1.36	1.51	0.1	0	4.39
438	Bupleuri Radix	MOL004082	1,3-Di(octadecanoyloxy)propan-2-yl octadecanoate	891.67	22.26	0	6	15.13	0.54	-1.12	0.13	0	
439	Ginseng Radix	MOL004100	N-Salicylidene-salicylamine	227.28	2.9	2	3	95.46	1.12	0.48	0.11	0	3.84
440	Bupleuri Radix	MOL004119	5-Methylfurfural	110.12	1.13	0	2	43.92	1.07	1.48	0.01	0.16	11.9
440	Cimicifugae Rhizoma	MOL004119	5-Methylfurfural	110.12	1.13	0	2	43.92	1.07	1.48	0.01	0.16	11.9
441	Ginseng Radix	MOL004174	Epsilon-Cadinene	204.39	4.85	0	0	16.41	1.82	2.12	0.08	0.29	
442	Ginseng Radix	MOL004237	delta-Elemene	204.39	4.73	0	0	25.99	1.84	2.1	0.06	0.31	
443	Cimicifugae Rhizoma	MOL004265	Ethyl ferulate	222.26	2.22	1	4	10.24	0.87	0.49	0.08	0.29	
444	Ginseng Radix	MOL004275	Epoxyhumulene II	220.39	3.8	0	1	23.66	1.58	1.77	0.1	0.29	
445	Citri Unshius Pericarpium	MOL004328	Naringenin	272.27	2.3	3	5	59.29	0.28	-0.37	0.21	0.4	16.98
445	Glycyrrhizae Radix et Rhizoma	MOL004328	Naringenin	272.27	2.3	3	5	59.29	0.28	-0.37	0.21	0.4	16.98
446	Bupleuri Radix	MOL004368	Hyperin	464.41	-0.59	8	12	6.94	-1.42	-2.08	0.77	0	
447	Glycyrrhizae Radix et Rhizoma	MOL004385	Yinyanghuo D	338.38	4.19	3	5	13.99	0.61	-0.53	0.38	0	
448	Angelicae Gigantis Radix	MOL004474	Maruzen M	122.18	2.51	1	1	48.44	1.57	1.81	0.02	0.37	11.63
449	Angelicae Gigantis Radix	MOL004479	o-Cresol	108.15	2.05	1	1	62.45	1.57	1.9	0.02	0.4	11.96
449	Cimicifugae Rhizoma	MOL004479	o-Cresol	108.15	2.05	1	1	62.45	1.57	1.9	0.02	0.4	11.96
450	Bupleuri Radix	MOL004480	Acetic acid	60.06	-0.23	1	2	47.87	0.42	0.72	0	0.29	12.21
451	Ginseng Radix	MOL004492	Chrysanthemaxanthin	584.96	8.24	2	3	38.72	0.51	-0.98	0.58	0.3	17.47
452	Ginseng Radix	MOL004498	12-O-Nicotinoylisolineolone	469.63	1.32	3	7	20.7	-0.54	-1.19	0.83	0.31	
453	Bupleuri Radix	MOL004581	Myrtenyl acetate	194.3	2.16	0	2	24.6	1.22	1.43	0.08	0.22	
454	Bupleuri Radix	MOL004582	Methyl naphthalene	142.21	3.23	0	0	39.01	1.9	1.91	0.04	0.47	11.81
455	Bupleuri Radix	MOL004583	11α-Methoxysaikosaponin f	931.25	-0.32	11	18	2	-3.01	-3.8	0.08	0.22	
456	Bupleuri Radix	MOL004584	11α-Methoxysaikosaponin f_qt	460.77	4.03	3	4	28.53	0.09	-0.42	0.75	0.22	
457	Bupleuri Radix	MOL004585	2,4-Dodecadienal	180.32	4.12	0	1	17.79	1.46	1.57	0.03	0.29	
458	Bupleuri Radix	MOL004586	trans,trans-Nona-2,4-dienol	140.25	2.5	1	1	27.45	1.19	1.13	0.02	0.27	
459	Bupleuri Radix	MOL004587	Heptenoic acid	128.19	2.24	1	2	36.1	0.84	1	0.01	0.31	1.49
460	Bupleuri Radix	MOL004588	(2R)-2-Methylcyclopentan-1-one	98.16	1.18	0	1	60.04	1.21	1.66	0.01	0.27	11.19
461	Bupleuri Radix	MOL004589	Methylheptane	114.26	3.82	0	0	28.65	1.79	2.12	0.01	0.21	
461	Glycyrrhizae Radix et Rhizoma	MOL004589	Methylheptane	114.26	3.82	0	0	28.65	1.79	2.12	0.01	0.21	
462	Bupleuri Radix	MOL004590	2-Methylhexadecane	240.53	7.92	0	0	4.19	1.84	2.01	0.07	0.16	
463	Bupleuri Radix	MOL004591	Nonenoic acid	156.25	3.15	1	2	65.17	0.95	1.08	0.02	0.29	4.57
464	Bupleuri Radix	MOL004592	2-Octenic acid	142.22	2.7	1	2	43.49	0.88	1	0.02	0.3	3.55
465	Bupleuri Radix	MOL004593	3'-O-Acetylsaikosaponin D	823.14	1.49	7	14	48.6	-1.6	-2.25	0.07	0.22	14.71
466	Bupleuri Radix	MOL004594	3'-O-Acetylsaikosaponin D_qt	472.78	3.71	3	4	26.16	0.16	-0.68	0.63	0.21	
467	Bupleuri Radix	MOL004595	3',6'-O,O-Diacetylsaikosaponin b2	865.18	1.92	7	15	17.74	-1.81	-2.69	0.09	0.24	
468	Bupleuri Radix	MOL004596	3',6'-O,O-Diacetylsaikosaponin b2_qt	472.78	3.77	4	4	14.19	-0.09	-0.99	0.74	0.21	
469	Bupleuri Radix	MOL004597	3'-O-Acetylsaikosaponin b2	825.16	1.79	8	14	8.33	-1.71	-2.56	0.11	0.21	
470	Bupleuri Radix	MOL004598	3',4',5',3,5,6,7-Heptamethoxyflavone	432.46	2.54	0	9	31.97	0.75	0.08	0.59	0.13	15.54
471	Bupleuri Radix	MOL004599	3,3,5-Trimethylheptane	142.32	4.28	0	0	47.49	1.8	2.1	0.02	0.25	10.76
472	Bupleuri Radix	MOL004600	Quercetin 3,4'-dimethyl ether	330.31	1.82	3	7	12.7	0.37	-0.5	0.33	0.31	
473	Bupleuri Radix	MOL004601	(3R)-3,7-Dimethyloctan-3-ol	158.32	3.24	1	1	33.08	1.26	1.3	0.02	0.24	5.57
474	Bupleuri Radix	MOL004602	(3R)-3-Methylcyclotridecan-1-one	210.4	4.62	0	1	37.65	1.47	1.78	0.07	0.16	7.26
475	Bupleuri Radix	MOL004603	3-Ethyl-2-methyl-1,3-hexadiene	124.25	3.62	0	0	45.42	1.85	2.14	0.01	0.29	11.81
476	Bupleuri Radix	MOL004604	Heptan-3-on	114.21	2	0	1	68.44	1.29	1.66	0.01	0.25	11
477	Bupleuri Radix	MOL004605	3-Methyldodecane	184.41	6.1	0	0	5.73	1.81	2.03	0.03	0.19	
478	Bupleuri Radix	MOL004606	4,8-Dimethyltridecane	212.47	6.81	0	0	2.77	1.81	1.96	0.04	0.19	
479	Bupleuri Radix	MOL004607	(3S,4R)-4-Methylheptan-3-ol	130.26	2.65	1	1	26.21	1.23	1.39	0.01	0.23	
480	Bupleuri Radix	MOL004608	(5S)-5-Butyloxolan-2-one	142.22	2.09	0	2	65.08	1.16	1.43	0.02	0.26	-0.76
481	Bupleuri Radix	MOL004609	Areapillin	360.34	2.29	3	8	48.96	0.6	-0.29	0.41	0.16	16.52
482	Bupleuri Radix	MOL004610	gamma-Undecalactone	184.31	3.46	0	2	49.12	1.25	1.41	0.04	0.22	4.48
483	Bupleuri Radix	MOL004611	5-Methyl-5-ethyldecane	184.41	5.85	0	0	6.58	1.84	2.02	0.03	0.2	
484	Bupleuri Radix	MOL004612	5-Methyldecane	156.35	5.19	0	0	15.23	1.81	2.04	0.02	0.2	
485	Angelicae Gigantis Radix	MOL004613	6,7,3',8'-Diligustilide	380.52	5.17	0	4	9.83	0.79	0.48	0.7	0.32	
485	Bupleuri Radix	MOL004613	6,7,3',8'-Diligustilide	380.52	5.17	0	4	9.83	0.79	0.48	0.7	0.32	
486	Bupleuri Radix	MOL004614	Fraxidin methyl ether	236.24	1.85	0	5	18.15	0.77	0.66	0.12	0.21	
487	Bupleuri Radix	MOL004615	6-O-Vanilloylajugol	498.53	-1.31	6	12	12.83	-1.57	-2.33	0.86	0.28	
488	Bupleuri Radix	MOL004616	7-Octen-4-ol	128.24	2.39	1	1	31.46	1.13	1.15	0.01	0.28	3.91
489	Bupleuri Radix	MOL004617	Ayapanin	176.18	1.88	0	3	41.55	0.97	0.84	0.06	0.3	1.66
490	Bupleuri Radix	MOL004618	7-Methyltridecane	198.44	6.55	0	0	4.64	1.83	1.99	0.04	0.18	
491	Bupleuri Radix	MOL004619	8-Nonenoic acid	156.25	2.78	1	2	52.31	0.94	1	0.02	0.3	4.54
492	Bupleuri Radix	MOL004620	Ara-Furan-A	267.28	-2.02	5	8	20.39	-1.65	-2.13	0.18	0.21	
493	Bupleuri Radix	MOL004621	Adonitol	152.17	-2.43	5	5	17.61	-1.34	-3.48	0.02	0.24	
494	Bupleuri Radix	MOL004623	Encecalin	232.3	2.34	0	3	21.36	1.17	1	0.11	0.28	
495	Bupleuri Radix	MOL004624	Longikaurin A	348.48	1.16	3	5	47.72	0.08	0.09	0.53	0.27	1.71
496	Bupleuri Radix	MOL004625	Longispinogenin 3-O-beta-D-glucuronopyranoside	634.94	3.57	6	9	9.68	-1.08	-1.76	0.34	0.26	
497	Bupleuri Radix	MOL004626	Longispinogenin	458.8	5.11	3	3	14.48	0.23	-0.2	0.75	0.22	
498	Bupleuri Radix	MOL004627	Methyl hexoate	130.21	2.06	0	2	52.44	1.17	1.26	0.01	0.19	5.62
499	Bupleuri Radix	MOL004628	Octalupine	264.41	-0.07	1	4	47.82	0.48	0.3	0.28	0.17	4.17
500	Bupleuri Radix	MOL004629	2-Phenylacetic acid	136.16	1.47	1	2	72.35	0.84	0.97	0.02	0.43	-2.03
501	Bupleuri Radix	MOL004631	7,8,4'-Trihydroxyisoflavone	270.25	2.07	3	5	20.67	0.45	-0.37	0.22	0.41	
502	Bupleuri Radix	MOL004632	Pulsatillic acid	470.76	5.39	2	4	24.49	0.12	-0.47	0.77	0.27	
503	Bupleuri Radix	MOL004633	Saikogenin F	472.78	3.71	3	4	25.88	0.09	-0.71	0.63	0.21	
504	Bupleuri Radix	MOL004634	Saikogenin G	472.78	3.71	3	4	16.92	0.21	-0.55	0.63	0.21	
505	Bupleuri Radix	MOL004635	Saikosaponin a	781.1	1.11	8	13	32.39	-1.95	-2.93	0.09	0.25	15.37
506	Bupleuri Radix	MOL004636	Saikosaponin B	781.1	1.17	9	13	5.94	-2.15	-3.13	0.13	0.23	
507	Bupleuri Radix	MOL004637	Saikosaponin D	781.1	1.11	8	13	34.39	-1.94	-2.89	0.09	0.25	15.16
508	Bupleuri Radix	MOL004638	Saikosaponin K	913.23	-0.07	11	17	2.94	-3.07	-3.8	0.06	0.25	
509	Bupleuri Radix	MOL004639	Saikosaponin V	1,107.39	-3.28	16	24	3.23	-3.94	-4.91	0.03	0.24	

510	Bupleuri Radix	MOL004640	Saikosaponin f	929.28	0.76	11	17	1.76	-3.02	-4.02	0.08	0.25	
511	Bupleuri Radix	MOL004641	(3S,4aS,6aR,6bS,8S,8aS,12aS,14aR,14bR)-4,4,6a,6b,11,11,14b	458.8	5.11	3	3	12.07	0.23	-0.75	0.75	0.22	
512	Bupleuri Radix	MOL004642	Saikosaponin t	813.15	0.94	9	14	34.46	-2.26	-3.13	0.13	0.22	4.93
513	Bupleuri Radix	MOL004643	Saikosaponin t_qt	488.83	4.49	3	4	16.69	0.26	-0.23	0.71	0.19	
514	Bupleuri Radix	MOL004644	Sainfuran	286.3	3.38	2	5	79.91	0.9	0.23	0.23	0.22	8.58
515	Bupleuri Radix	MOL004646	Thymonin	360.34	2.29	3	8	1.97	0.65	-0.36	0.41	0.21	
516	Bupleuri Radix	MOL004647	Tridecanoic acid	214.39	5	1	2	22.32	1.04	0.89	0.05	0.21	
516	Ginseng Radix	MOL004647	Tridecanoic acid	214.39	5	1	2	22.32	1.04	0.89	0.05	0.21	
517	Bupleuri Radix	MOL004648	Troxerutin	346.56	5.89	3	3	31.6	0.35	-0.38	0.28	0.3	4.36
518	Bupleuri Radix	MOL004649	1-[(2R,3R,4S,5S)-3,4-Dihydroxy-5-(hydroxymethyl)oxolan-2-	244.23	-2.45	4	8	17.85	-1.19	-1.69	0.11	0.29	
519	Bupleuri Radix	MOL004650	Acetovanillin	194.2	1.34	0	4	28.17	0.59	0.38	0.06	0.28	
520	Bupleuri Radix	MOL004651	cis-2-Undecenal	168.31	4.11	0	1	47.07	1.39	1.66	0.03	0.26	7.22
521	Bupleuri Radix	MOL004652	alpha-Spinasterol glucoside	574.93	5.89	4	6	21.2	-0.2	-0.99	0.63	0.21	
522	Bupleuri Radix	MOL004653	(+)-Anomalin	426.5	5.05	0	7	46.06	0.46	0	0.66	0.36	1.03
523	Bupleuri Radix	MOL004654	2-Heptenol	114.21	2.03	1	1	26.46	1.11	1.23	0.01	0.22	
524	Bupleuri Radix	MOL004655	Phenylbenzene	154.22	3.35	0	0	29.79	1.9	1.89	0.04	0.47	
525	Bupleuri Radix	MOL004656	S-Carvotanaacetone	152.26	2.56	0	1	35.17	1.35	1.69	0.03	0.29	11.52
526	Bupleuri Radix	MOL004657	Caryophyllene oxide	220.39	3.52	0	1	29.06	1.57	1.7	0.13	0.27	
527	Bupleuri Radix	MOL004658	Cedrenol	220.39	2.92	1	1	108.56	1.3	1.47	0.12	0.25	7.48
528	Bupleuri Radix	MOL004659	Chikusaikoside II	943.26	-0.64	11	18	5.17	-3.14	-4.05	0.05	0.23	
529	Bupleuri Radix	MOL004660	Chikusaikoside II_qt	472.78	3.71	3	4	17.48	0.15	-0.66	0.63	0.2	
530	Bupleuri Radix	MOL004661	[(3R)-3,7-Dimethyloct-6-enyl] acetate	198.34	3.43	0	2	45.54	1.34	1.39	0.04	0.27	5.58
531	Bupleuri Radix	MOL004662	Cyclohexylisocyanate	125.19	1.97	0	2	41.41	1.1	1.31	0.02	0.41	-2.88
532	Bupleuri Radix	MOL004663	Vinylxylene	132.22	3.35	0	0	20.79	1.91	2.06	0.02	0.42	
533	Bupleuri Radix	MOL004664	Heptanoic acid	130.21	2.26	1	2	13.38	0.87	0.98	0.01	0.26	
533	Cimicifugae Rhizoma	MOL004664	Heptanoic acid	130.21	2.26	1	2	13.38	0.87	0.98	0.01	0.26	
534	Bupleuri Radix	MOL004665	Ethyl geranate	196.32	3.74	0	2	64.07	1.41	1.43	0.04	0.29	7.16
535	Bupleuri Radix	MOL004666	Ethyl protocatechuate	182.19	1.5	2	4	35.77	0.59	0.35	0.05	0.38	2.69
536	Bupleuri Radix	MOL004667	Fraxetin	208.18	1.35	2	5	23.04	0.51	0.36	0.09	0.28	
537	Bupleuri Radix	MOL004668	beta-D-Galactopyranose	180.18	-2.51	5	6	47.71	-1.89	-4.41	0.04	0.28	11
538	Bupleuri Radix	MOL004670	cis-Pinocampheol	154.28	1.95	1	1	53.92	1.19	1.5	0.06	0.24	11.36
539	Bupleuri Radix	MOL004671	Isopulegol	154.28	2.58	1	1	50.72	1.24	1.42	0.03	0.26	11.05
540	Bupleuri Radix	MOL004673	Kaempferitrin	578.57	-0.45	8	14	8.16	-1.74	-2.65	0.79	0.3	
541	Bupleuri Radix	MOL004674	Kaempferol-3,7-α-L-dirhamnoside	578.57	-0.45	8	14	8.16	-1.75	-2.98	0.79	0.28	
542	Bupleuri Radix	MOL004675	Kaempferol-7-O-rhamnoside	432.41	0.76	6	10	2.27	-0.91	-1.83	0.72	0.34	
543	Bupleuri Radix	MOL004676	Kaempferol-7-O-α-L-rhamnoside	432.41	0.76	6	10	22.14	-0.91	-2.02	0.72	0.33	
544	Bupleuri Radix	MOL004677	Ledol	222.41	3.2	1	1	82.78	1.32	1.55	0.12	0.22	12.11
545	Bupleuri Radix	MOL004678	Limetin	206.21	1.87	0	4	36.63	0.88	0.47	0.09	0.22	1.33
546	Bupleuri Radix	MOL004679	Longifolene	204.39	4.18	0	0	39.49	1.83	2.08	0.11	0.23	11.2
547	Bupleuri Radix	MOL004680	cis-p-2-Menthen-1-ol	154.28	2.36	1	1	35.3	1.22	1.43	0.03	0.26	10.66
548	Bupleuri Radix	MOL004681	(6S)-6-Pentyl-5,6-dihydropyran-2-one	168.26	2.98	0	2	21	1.22	1.41	0.03	0.26	
549	Bupleuri Radix	MOL004682	Methyl octylate	158.27	2.97	0	2	18.71	1.23	1.32	0.02	0.18	
550	Bupleuri Radix	MOL004683	Methyl (2E,4E)-octadeca-2,4-dienoate	294.53	7.06	0	2	38.77	1.45	1.39	0.17	0.21	5.98
551	Bupleuri Radix	MOL004684	Methyl (E)-octadec-2-enoate	296.55	7.51	0	2	29.84	1.42	1.4	0.17	0.18	
552	Bupleuri Radix	MOL004685	(E)-Non-2-en-4-one	140.25	2.89	0	1	37.78	1.35	1.55	0.02	0.28	6.81
553	Bupleuri Radix	MOL004686	Nonenone	140.25	2.68	0	1	19.62	1.36	1.58	0.02	0.3	
554	Bupleuri Radix	MOL004687	2-Octanone	128.24	2.25	0	1	19.29	1.35	1.57	0.01	0.25	
555	Bupleuri Radix	MOL004688	Cumic acid	164.22	2.63	1	2	45.78	1.03	1.1	0.04	0.38	-3.13
556	Bupleuri Radix	MOL004690	L-(+)-Rhamnose monohydrate	164.18	-1.8	4	5	40.73	-1.3	-3.7	0.03	0.31	11.06
557	Bupleuri Radix	MOL004691	Aldehydo-D-ribose	150.15	-2.17	4	5	40.76	-1.59	-4.17	0.02	0.3	11.26
558	Bupleuri Radix	MOL004693	Saikosaponin b1	781.1	1.17	9	13	6.7	-2.14	-2.96	0.13	0.25	
559	Bupleuri Radix	MOL004694	Saikosaponin b1_qt	472.78	3.77	4	4	13.45	-0.1	-1.03	0.74	0.21	
560	Bupleuri Radix	MOL004695	Saikosaponin b2	781.1	1.17	9	13	20.45	-2.02	-2.94	0.13	0.22	
561	Bupleuri Radix	MOL004696	Saikosaponin b2_qt	472.78	3.77	4	4	13.79	-0.15	-1.06	0.74	0.22	
562	Bupleuri Radix	MOL004697	Saikosaponin b3	813.15	0.79	9	14	8.84	-2.24	-3.27	0.12	0.22	
563	Bupleuri Radix	MOL004698	Saikosaponin b3_qt	504.83	3.4	4	5	18.92	-0.2	-1	0.69	0.2	
564	Bupleuri Radix	MOL004699	Saikosaponin b4	813.15	0.79	9	14	9.56	-1.87	-2.95	0.12	0.2	
565	Bupleuri Radix	MOL004700	Saikosaponin b4_qt	504.83	3.4	4	5	18.92	-0.22	-1.15	0.69	0.18	
566	Bupleuri Radix	MOL004701	Saikosaponin c	943.26	-0.64	11	18	5.12	-2.92	-4.04	0.05	0.2	
567	Bupleuri Radix	MOL004702	Saikosaponin c_qt	472.78	3.71	3	4	30.5	0.03	-0.85	0.63	0.2	6.12
568	Bupleuri Radix	MOL004703	Saikosaponin e	765.1	2.2	7	12	17.7	-1.29	-2.18	0.09	0.23	
569	Bupleuri Radix	MOL004704	Saikosaponin e_qt	456.78	4.8	2	3	17.62	0.65	-0.04	0.66	0.22	
570	Bupleuri Radix	MOL004705	(3E,6S,7R)-3-Butylidene-6,7-dihydroxy-4,5,6,7-tetrahydroisc	224.28	1.32	2	4	34.34	-0.02	-0.19	0.1	0.3	4.2
571	Bupleuri Radix	MOL004706	Spathulenol	220.39	3.01	1	1	80.01	1.26	1.31	0.12	0.26	12.19
572	Bupleuri Radix	MOL004707	tau-Cadinol	222.41	3.78	1	1	36.51	1.31	1.42	0.09	0.23	7.84
573	Bupleuri Radix	MOL004708	delta-Cadinol	222.41	3.78	1	1	14.03	1.33	1.45	0.09	0.23	
574	Bupleuri Radix	MOL004709	(+)-trans-Carveol	152.26	2.4	1	1	44.21	1.19	1.31	0.03	0.31	11.64
575	Bupleuri Radix	MOL004710	D-Tryptophan	204.25	1.25	4	3	75.63	0.29	0.05	0.08	0.29	-2.1
576	Bupleuri Radix	MOL004711	Veratryl alcohol	168.21	1.19	1	3	71.49	0.67	0.45	0.04	0.21	6.57
577	Bupleuri Radix	MOL004712	Xylitol	152.17	-2.43	5	5	27.37	-1.51	-3.8	0.02	0.21	
578	Bupleuri Radix	MOL004713	alpha-Eudesmol	222.41	3.67	1	1	25.02	1.31	1.29	0.1	0.25	
579	Bupleuri Radix	MOL004714	(1R,4S)-7,7-Dimethyl-2-methylenenorbornane	136.26	2.93	0	0	41.06	1.8	2.13	0.04	0.26	11.22
580	Bupleuri Radix	MOL004715	(4aS,9aS)-2,9,9-Trimethyl-5-methylene-4,4a,6,7,8,9a-hexahy	204.39	4.75	0	0	48.89	1.82	2.01	0.08	0.26	5.01
581	Bupleuri Radix	MOL004716	(1S,4R)-2,3-Dimethylbicyclo[2.2.1]hept-2-ene	122.23	2.86	0	0	43.09	1.84	2.26	0.03	0.25	11.4
582	Bupleuri Radix	MOL004717	(3R,4aR,8aR)-3-Isopropenyl-5,8a-dimethyl-2,3,4,4a,7,8-hexa	204.39	4.75	0	0	23.86	1.84	2.05	0.08	0.27	
583	Bupleuri Radix	MOL004718	alpha-Spinasterol	412.77	7.64	1	1	42.98	1.28	0.79	0.76	0.22	6.46
584	Bupleuri Radix	MOL004719	alpha-Spinasteryl glucoside	574.93	5.89	4	6	21.2	-0.15	-0.89	0.63	0.22	
585	Bupleuri Radix	MOL004720	beta-Cedrene	204.39	4.18	0	0	54.35	1.79	2.12	0.11	0.25	5.01
586	Bupleuri Radix	MOL004721	beta-Fenchene	136.26	2.63	0	0	39.16	1.8	2.22	0.04	0.25	11.13
587	Bupleuri Radix	MOL004722	beta-Oplopenone	220.39	3.49	0	1	42.08	1.42	1.67	0.09	0.29	12.17
588	Angelicae Gigantis Radix	MOL004723	beta-Terpinene	136.26	3.5	0	0	42.29	1.85	2.12	0.02	0.29	11.21
588	Bupleuri Radix	MOL004723	beta-Terpinene	136.26	3.5	0	0	42.29	1.85	2.12	0.02	0.29	11.21
588	Glycyrrhizae Radix et Rhizoma	MOL004723	beta-Terpinene	136.26	3.5	0	0	42.29	1.85	2.12	0.02	0.29	11.21
589	Bupleuri Radix	MOL004724	gamma-Cadinene	204.39	4.8	0	0	23.4	1.84	2.05	0.08	0.26	
590	Bupleuri Radix	MOL004725	(5S)-5-Hexyloxolan-2-one	170.28	3	0	2	19.71	1.24	1.3	0.03	0.22	
591	Bupleuri Radix	MOL004726	(5S)-5-Propyloxolan-2-one	128.19	1.63	0	2	78.49	1.13	1.36	0.02	0.26	10.75
592	Bupleuri Radix	MOL004727	gamma-Muurolene	204.39	4.8	0	0	21.35	1.84	2.1	0.08	0.27	
593	Bupleuri Radix	MOL004728	gamma-Patchoulene	204.39	4.18	0	0	54.63	1.81	2.24	0.11	0.25	12.23
594	Angelicae Gigantis Radix	MOL004734	Butal	72.12	0.94	0	1	68.66	1.18	1.55	0	0.27	11.61
595	Angelicae Gigantis Radix	MOL004791	Ethol	242.5	6.45	1	1	13.32	1.31	1.07	0.08	0.15	
596	Angelicae Gigantis Radix	MOL004792	Nodakenin	408.44	0.28	4	9	57.12	-0.79	-1.42	0.69	0.27	7.16
597	Glycyrrhizae Radix et Rhizoma	MOL004801	2',7-Dihydroxy-4'-methoxyisoflavan-7-O-β-d-glucopyranos	434.48	0.59	5	9	10.46	-1.02	-1.82	0.73	0.31	
598	Glycyrrhizae Radix et Rhizoma	MOL004802	(E)-1-Butoxyhex-2-ene	156.3	3.31	0	1	41.72	1.5	1.56	0.02	0	6.68
599	Glycyrrhizae Radix et Rhizoma	MOL004803	3-Hydroxyglabrol	408.53	5.73	3	5	4.73	0.48	-0.31	0.58	0.33	
600	Glycyrrhizae Radix et Rhizoma	MOL004804	18beta-Glycyrrhetinic acid	470.76	5.49	2	4	22.05	0.1	-0.53	0.74	0	
601	Glycyrrhizae Radix et Rhizoma	MOL004805	Shinflavanone	390.51	5.48	1	4	31.79	1	0.25	0.72	0.35	14.82
602	Glycyrrhizae Radix et Rhizoma	MOL004806	Euchrenone	406.56	6.35	1	4	30.29	1.09	0.39	0.57	0	15.89
603	Glycyrrhizae Radix et Rhizoma	MOL004807	Glucuronic acid	194.16	-2.31	5	7	46.18	-2.05	-4.58	0.06	0	11.13
604	Glycyrrhizae Radix et Rhizoma	MOL004808	Glyasperin B	370.43	4.02	3	6	65.22	0.47	-0.09	0.44	0	16.1
605	Glycyrrhizae Radix et Rhizoma	MOL004809	Glyasperin E	444.51	6.41	2	6	4.12	0.69	-0.41	0.75	0	
606	Glycyrrhizae Radix et Rhizoma	MOL004810	Glyasperin F	354.38	2.97	3	6	75.84	0.43	-0.15	0.54	0	15.64
607	Glycyrrhizae Radix et Rhizoma	MOL004811	Glyasperin C	356.45	4.73	3	5	45.56	0.71	0.07	0.4	0	3.13
608	Glycyrrhizae Radix et Rhizoma	MOL004812	Glyasperins D	370.48	4.99	2	5	29.91	0.89	0.25	0.43	0	
609	Glycyrrhizae Radix et Rhizoma	MOL004813	Glyasperins Z	340.45	5	2	4	4.17	1.09	0.53	0.36	0	
610	Glycyrrhizae Radix et Rhizoma	MOL004814	Isotrifoliol	298.26	2.99	2	6	31.94	0.53	-0.25	0.42	0	7.91
611	Glycyrrhizae Radix et Rhizoma	MOL004815	Kanzonol B	322.38	3.96	2	4	39.62	0.66	-0.12	0.35	0	16.16
612	Glycyrrhizae Radix et Rhizoma	MOL004816	Kanzonol Y	410.55	5.96	4	5	1.06	0.33	-0.35	0.48	0	
613	Glycyrrhizae Radix et Rhizoma	MOL004817	Kanzonols K	436.54	5.76	3	6	0.97	0.76	0.05	0.66	0	
614	Glycyrrhizae Radix et Rhizoma	MOL004818	Kanzonols L	490.64	7.04	3	6	0.98	0.7	0.11	0.78	0	
615	Glycyrrhizae Radix et Rhizoma	MOL004819	Kanzonols T	440.53	4.41	5	7	17.87	0.05	-0.66	0.67	0	
616	Glycyrrhizae Radix et Rhizoma	MOL004820	Kanzonols W	336.36	3.63	2	5	50.48	0.63	0.04	0.52	0	0.15
617	Glycyrrhizae Radix et Rhizoma	MOL004821	Kanzonols X	394.55	6.61	3	4	7.56	1.1	0.34	0.56	0	
618	Glycyrrhizae Radix et Rhizoma	MOL004822	Licoagrochalcone A	324.4	4.76	3	4	1.04	0.55	-0.22	0.27	0	

619	Glycyrrhizae Radix et Rhizoma	MOL004823	Licoagropin	320.46	5.77	0	2	27.14	1.63	1.08	0.51	0	
620	Glycyrrhizae Radix et Rhizoma	MOL004824	(2S)-6-(2,4-Dihydroxyphenyl)-2-(2-hydroxypropan-2-yl)-4-r	384.41	2.96	3	7	60.25	0	-0.76	0.63	0	4.31
621	Glycyrrhizae Radix et Rhizoma	MOL004825	Glyinflanin A	408.53	6.05	4	5	1.06	0.69	0.03	0.48	0.38	
622	Glycyrrhizae Radix et Rhizoma	MOL004827	Semilicoisoflavone B	352.36	2.85	3	6	48.78	0.45	-0.33	0.55	0	17.02
623	Glycyrrhizae Radix et Rhizoma	MOL004828	Glepidotin A	338.38	3.9	3	5	44.72	0.79	0.06	0.35	0	16.09
624	Glycyrrhizae Radix et Rhizoma	MOL004829	Glepidotin B	340.4	3.88	3	5	64.46	0.46	-0.09	0.34	0	15.98
625	Glycyrrhizae Radix et Rhizoma	MOL004830	Octadiene	110.22	3.19	0	0	34.53	1.81	2.07	0.01	0	3.75
626	Glycyrrhizae Radix et Rhizoma	MOL004831	Kanzonol C	392.53	6.61	3	4	1.02	0.81	0.01	0.45	0.37	
627	Glycyrrhizae Radix et Rhizoma	MOL004832	Butyl benzoate	178.25	3.01	0	2	48.41	1.31	1.42	0.04	0	7.28
628	Glycyrrhizae Radix et Rhizoma	MOL004833	Phaseolinisoflavan	324.4	3.95	2	4	32.01	1.01	0.46	0.45	0	2.66
629	Glycyrrhizae Radix et Rhizoma	MOL004834	3-(2-Hydroxy-4-methoxyphenyl)-2H-chromen-7-ol	270.3	2.96	2	4	4.66	0.89	0.16	0.21	0	
630	Glycyrrhizae Radix et Rhizoma	MOL004835	Glypallichalcone	284.33	3.4	1	4	61.6	0.76	0.23	0.19	0	17.01
631	Glycyrrhizae Radix et Rhizoma	MOL004836	Echinatin	270.3	3.15	2	4	66.58	0.38	-0.18	0.17	0	19.56
632	Glycyrrhizae Radix et Rhizoma	MOL004837	Karenzu DK2	224.27	3.16	0	2	62.26	0.94	0.64	0.1	0	33.72
633	Glycyrrhizae Radix et Rhizoma	MOL004838	Kanzonol U	308.35	4.2	2	4	58.44	1	0.34	0.38	0.34	8.71
634	Glycyrrhizae Radix et Rhizoma	MOL004839	(1S,2S)-1,2-Dimethylcyclopentane	98.21	2.79	0	0	41.78	1.78	2.26	0.01	0	10.93
635	Glycyrrhizae Radix et Rhizoma	MOL004840	Liconeolignan	354.43	5.23	2	5	4.41	1	0.04	0.4	0	
636	Glycyrrhizae Radix et Rhizoma	MOL004841	Licochalcone B	286.3	2.88	3	5	76.76	0.47	-0.46	0.19	0	17.02
637	Glycyrrhizae Radix et Rhizoma	MOL004842	Licochalcone C	338.43	5.01	2	4	4.44	0.63	0.05	0.29	0	
638	Glycyrrhizae Radix et Rhizoma	MOL004843	Licochalcone D	354.43	4.74	3	5	1.01	0.47	0.06	0.34	0	
639	Glycyrrhizae Radix et Rhizoma	MOL004844	Glabrol	392.53	6.28	2	4	4.25	0.84	0.06	0.54	0	
640	Glycyrrhizae Radix et Rhizoma	MOL004845	Apioglycyrrhizin	779.03	2.54	7	14	17.8	-1.91	-2.67	0.14	0	
641	Glycyrrhizae Radix et Rhizoma	MOL004846	Apioglycyrrhizin_qt	470.76	5.49	2	4	23.73	0.1	-0.56	0.74	0	
642	Glycyrrhizae Radix et Rhizoma	MOL004847	2,2-Dimethylphentane	100.23	3.11	0	0	55.33	1.79	2.11	0.01	0	11
643	Glycyrrhizae Radix et Rhizoma	MOL004848	Licochalcone G	354.43	4.35	3	5	49.25	0.64	-0.04	0.32	0.35	15.75
644	Glycyrrhizae Radix et Rhizoma	MOL004849	Licoarylcoumarin	368.41	4.03	3	6	59.62	0.4	-0.23	0.43	0	0.69
645	Glycyrrhizae Radix et Rhizoma	MOL004850	Liquoric acid	484.74	4.05	2	5	25.44	-0.01	-0.52	0.55	0	
646	Glycyrrhizae Radix et Rhizoma	MOL004851	Licoflavone	322.38	4.46	2	4	18.75	0.82	-0.31	0.33	0	
647	Glycyrrhizae Radix et Rhizoma	MOL004852	Licoflavone B	390.51	6.32	2	4	4.44	0.88	-0.17	0.56	0	
648	Glycyrrhizae Radix et Rhizoma	MOL004853	Licoflavonol	354.38	3.63	4	6	8.75	0.49	-0.3	0.4	0	
649	Glycyrrhizae Radix et Rhizoma	MOL004855	Licoricone	382.44	4.16	2	6	63.58	0.53	-0.14	0.47	0	16.37
650	Glycyrrhizae Radix et Rhizoma	MOL004856	Gancaonin A	352.41	4.17	2	5	51.08	0.8	0.13	0.4	0	16.82
651	Glycyrrhizae Radix et Rhizoma	MOL004857	Gancaonin B	368.41	3.91	3	6	48.79	0.58	-0.1	0.45	0	16.49
652	Glycyrrhizae Radix et Rhizoma	MOL004858	Gancaonin C	354.38	2.83	4	6	2.87	0.14	-0.87	0.42	0	
653	Glycyrrhizae Radix et Rhizoma	MOL004859	2,3-Dimethylhexane	114.26	3.61	0	0	46.24	1.78	2.16	0.01	0	11.01
654	Glycyrrhizae Radix et Rhizoma	MOL004860	Glycyrrhizae Radix et Rhizoma glycoside E	693.71	1.59	7	14	32.89	-2.06	-2.8	0.27	0.31	25.39
655	Glycyrrhizae Radix et Rhizoma	MOL004861	Gancaonin D	384.41	2.81	4	7	2.72	-0.11	-0.93	0.51	0	
656	Glycyrrhizae Radix et Rhizoma	MOL004862	(2R)-2-[3,4-Dihydroxy-5-(3-methylbut-2-enyl)phenyl]-5,7-d	424.53	5.74	4	6	1.21	0.51	-0.35	0.63	0	
657	Glycyrrhizae Radix et Rhizoma	MOL004863	Gancaonin L	354.38	3.65	4	6	66.37	0.52	-0.13	0.41	0	15.81
658	Glycyrrhizae Radix et Rhizoma	MOL004864	5,7-Dihydroxy-3-(4-methoxyphenyl)-8-(3-methylbut-2-enyl	352.41	4.17	2	5	30.49	0.9	0.21	0.41	0	14.99
659	Glycyrrhizae Radix et Rhizoma	MOL004865	Gancaonin N	368.41	3.91	3	6	2.47	0.58	-0.14	0.45	0	
660	Glycyrrhizae Radix et Rhizoma	MOL004866	Gancaonin O	354.38	3.92	4	6	44.15	0.48	-0.28	0.41	0	16.77
661	Glycyrrhizae Radix et Rhizoma	MOL004867	Gancaonin P	370.38	3.36	5	7	1.41	0.27	-0.4	0.45	0	
662	Glycyrrhizae Radix et Rhizoma	MOL004868	Gancaonin Q	406.51	6.05	3	5	8.98	0.86	-0.01	0.6	0	
663	Glycyrrhizae Radix et Rhizoma	MOL004869	Gancaonin R	382.54	6.9	4	4	1.26	1.03	0.25	0.37	0	
664	Glycyrrhizae Radix et Rhizoma	MOL004870	Gancaonin S	382.54	6.9	4	4	1.26	0.98	0.18	0.38	0	
665	Glycyrrhizae Radix et Rhizoma	MOL004871	(3S)-2,3-Dimethylpentane	100.23	3.16	0	0	35.57	1.78	2.23	0.01	0	11.19
666	Glycyrrhizae Radix et Rhizoma	MOL004872	Gancaonin T	398.54	5.45	4	5	1.04	0.5	-0.43	0.53	0	
667	Glycyrrhizae Radix et Rhizoma	MOL004873	Gancaonin U	380.52	6.59	4	4	14.53	1.08	0.6	0.53	0	
668	Glycyrrhizae Radix et Rhizoma	MOL004874	Gancaonin V	312.39	4.74	4	4	1.24	0.73	0.04	0.34	0	
669	Glycyrrhizae Radix et Rhizoma	MOL004875	Glicoricone	368.41	3.91	3	6	2.47	0.43	-0.3	0.44	0	
670	Cimicifugae Rhizoma	MOL004876	Glycyram	823.04	2.42	8	16	19.62	-2.66	-2.86	0.11	0	
670	Glycyrrhizae Radix et Rhizoma	MOL004876	Glycyram	823.04	2.42	8	16	19.62	-2.66	-2.86	0.11	0	
671	Glycyrrhizae Radix et Rhizoma	MOL004877	Licoricidin	424.58	6.59	3	5	0.99	0.96	0.3	0.62	0	
672	Glycyrrhizae Radix et Rhizoma	MOL004878	Glycycoumarin	368.41	4.42	3	6	23.56	0.52	-0.24	0.44	0	
673	Glycyrrhizae Radix et Rhizoma	MOL004879	Glycyrin	382.44	4.67	2	6	52.61	0.59	-0.13	0.47	0	1.31
674	Glycyrrhizae Radix et Rhizoma	MOL004880	5,6,7,8-Tetrahydro-2,4-dimethylquinoline	161.27	2.97	0	1	49.77	1.64	1.7	0.05	0	-2.84
675	Glycyrrhizae Radix et Rhizoma	MOL004881	Morachalcone A	340.4	4.49	4	5	1.36	0.5	-0.22	0.3	0.38	
676	Glycyrrhizae Radix et Rhizoma	MOL004882	Licocoumarone	340.4	4.98	3	5	33.21	0.84	0.06	0.36	0	9.66
677	Glycyrrhizae Radix et Rhizoma	MOL004883	Licoisoflavone	354.38	3.65	4	6	41.61	0.37	-0.27	0.42	0	16.09
678	Glycyrrhizae Radix et Rhizoma	MOL004884	Licoisoflavone B	352.36	2.85	3	6	38.93	0.46	-0.18	0.55	0	15.73
679	Glycyrrhizae Radix et Rhizoma	MOL004885	Licoisoflavanone	354.38	2.97	3	6	52.47	0.39	-0.22	0.54	0	15.67
680	Glycyrrhizae Radix et Rhizoma	MOL004886	Glycyrrhizae Radix et Rhizoma -saponin C2	807.04	3.1	8	15	59.66	-2.28	-2.89	0.11	0	3.56
681	Glycyrrhizae Radix et Rhizoma	MOL004887	Glycyrrhizae Radix et Rhizoma -saponin C2_qt	454.76	6.17	2	3	17.33	0.48	-0.3	0.76	0	
682	Glycyrrhizae Radix et Rhizoma	MOL004888	Glycyrrhizae Radix et Rhizoma -saponin F3	983.18	1.32	10	21	17.68	-2.82	-3.47	0.03	0	
683	Glycyrrhizae Radix et Rhizoma	MOL004889	Glycyrrhizae Radix et Rhizoma -saponin F3_qt	454.76	5.93	1	3	27.53	0.74	0.07	0.64	0	
684	Glycyrrhizae Radix et Rhizoma	MOL004890	(4S)-2,4-Dimethylhexane	114.26	3.61	0	0	37.13	1.77	2.19	0.01	0	10.95
685	Glycyrrhizae Radix et Rhizoma	MOL004891	Shinpterocarpin	322.38	3.46	1	4	80.3	1.1	0.68	0.73	0.32	6.5
686	Glycyrrhizae Radix et Rhizoma	MOL004892	Glycyrrhizae Radix et Rhizoma -saponin G2	839.04	1.33	9	17	6.39	-2.01	-2.84	0.11	0	
687	Glycyrrhizae Radix et Rhizoma	MOL004893	Glycyrrhizae Radix et Rhizoma -saponin G2_qt	486.76	4.4	3	5	22.78	-0.27	-0.74	0.72	0	
688	Glycyrrhizae Radix et Rhizoma	MOL004894	Glycyrrhizae Radix et Rhizoma -saponin H2	823.04	2.42	8	16	44.37	-2.08	-2.83	0.11	0	5.09
689	Glycyrrhizae Radix et Rhizoma	MOL004895	Glycyrrhizae Radix et Rhizoma -saponin H2_qt	470.76	5.49	2	4	22.91	0.01	-0.48	0.74	0	
690	Glycyrrhizae Radix et Rhizoma	MOL004896	Glycyrrhizae Radix et Rhizoma -saponin J2	825.06	2.26	9	16	6.25	-2.25	-2.84	0.11	0	
691	Glycyrrhizae Radix et Rhizoma	MOL004897	Glycyrrhizae Radix et Rhizoma -saponin J2_qt	472.78	5.33	3	4	28.3	0.04	-0.82	0.74	0	
692	Glycyrrhizae Radix et Rhizoma	MOL004898	2',3,4,4'-Tetrahydroxy-5-prenylchalcone	340.4	4.49	4	5	46.27	0.41	-0.4	0.31	0.43	15.24
693	Glycyrrhizae Radix et Rhizoma	MOL004899	Glycyrrhizae Radix et Rhizoma -saponin B2	809.06	3.35	8	15	58.55	-2.4	-2.96	0.11	0.3	4.24
694	Glycyrrhizae Radix et Rhizoma	MOL004900	Glycyrrhizae Radix et Rhizoma -saponin K2	823.04	2.01	9	16	7.82	-2.64	-3.25	0.11	0	
695	Glycyrrhizae Radix et Rhizoma	MOL004901	Glycyrrhizae Radix et Rhizoma -saponin K2_qt	470.76	5.08	3	4	27.79	0.05	-0.67	0.75	0	
696	Glycyrrhizae Radix et Rhizoma	MOL004902	Glycyrrhetol	456.78	5.28	2	3	14.66	0.36	-0.26	0.75	0	
697	Glycyrrhizae Radix et Rhizoma	MOL004903	Liquiritin	418.43	0.66	5	9	65.69	-1.06	-1.93	0.74	0	17.96
698	Glycyrrhizae Radix et Rhizoma	MOL004904	Licopyranocoumarin	384.41	3.04	3	7	80.36	0.13	-0.62	0.65	0	0.08
699	Glycyrrhizae Radix et Rhizoma	MOL004905	3,22-Dihydroxy-11-oxo-delta(12)-oleanene-27-alpha-meth	512.75	4.37	1	6	34.32	-0.06	-0.75	0.55	0	3.56
700	Glycyrrhizae Radix et Rhizoma	MOL004906	Hispaglabridin B	390.51	5	1	4	22.94	1.18	0.62	0.88	0.31	
701	Glycyrrhizae Radix et Rhizoma	MOL004907	Glyzaglabrin	298.26	2.1	2	6	61.07	0.34	-0.2	0.35	0	21.2
702	Glycyrrhizae Radix et Rhizoma	MOL004908	Glabridin	324.4	3.95	2	4	53.25	0.97	0.36	0.47	0	0.03
703	Glycyrrhizae Radix et Rhizoma	MOL004909	Glabrolide	468.74	5	1	4	17.46	0.29	-0.48	0.61	0	
704	Glycyrrhizae Radix et Rhizoma	MOL004910	Glabranin	324.4	4.42	2	4	52.9	0.97	0.31	0.31	0	16.24
705	Glycyrrhizae Radix et Rhizoma	MOL004911	Glabrene	322.38	3.77	2	4	46.27	0.99	0.04	0.44	0	3.63
706	Glycyrrhizae Radix et Rhizoma	MOL004912	Glabrone	336.36	3.12	2	5	52.51	0.59	-0.11	0.5	0	16.09
707	Glycyrrhizae Radix et Rhizoma	MOL004913	Hedysarimcoumestan B	298.26	2.99	2	6	48.14	0.48	-0.19	0.43	0	8.87
708	Glycyrrhizae Radix et Rhizoma	MOL004914	1,3-Dihydroxy-8,9-dimethoxy-6-benzofurano[3,2-c]chrome	328.29	2.98	2	7	62.9	0.4	-0.34	0.53	0	9.32
709	Glycyrrhizae Radix et Rhizoma	MOL004915	Eurycarpin A	338.38	3.92	3	5	43.28	0.43	-0.06	0.37	0	17.1
710	Glycyrrhizae Radix et Rhizoma	MOL004916	2-Methyl-5-propyl -nonane	184.41	5.89	0	0	15.28	1.81	2.03	0.03	0	
711	Glycyrrhizae Radix et Rhizoma	MOL004917	Glycyroside	562.57	-0.73	6	13	37.25	-1.58	-2.56	0.79	0	14.62
712	Glycyrrhizae Radix et Rhizoma	MOL004918	Hexane	86.2	3.11	0	0	52.5	1.78	2.14	0	0	10.8
713	Glycyrrhizae Radix et Rhizoma	MOL004919	Sextone B	98.21	2.99	0	0	56.2	1.77	2.14	0.01	0	10.85
714	Glycyrrhizae Radix et Rhizoma	MOL004920	Methylcyclopentane	84.18	2.53	0	0	55.78	1.79	2.22	0.01	0	10.97
715	Glycyrrhizae Radix et Rhizoma	MOL004921	Docosyl caffeate	488.83	11.16	2	4	3.14	1.01	0.31	0.59	0	
716	Glycyrrhizae Radix et Rhizoma	MOL004922	2-Methyl-6-ethyl decane	184.41	5.89	0	0	5.5	1.81	2	0.03	0	
717	Glycyrrhizae Radix et Rhizoma	MOL004924	(-)-Medicocarpin	432.46	0.75	4	9	40.99	-0.6	-1.34	0.95	0	13.2
718	Glycyrrhizae Radix et Rhizoma	MOL004925	Vitexin	432.41	-0.06	7	10	3.05	-1.52	-2.41	0.71	0	
719	Glycyrrhizae Radix et Rhizoma	MOL004926	Choerospondin	434.43	0.39	6	10	14.03	-1.13	-2.08	0.78	0	
720	Glycyrrhizae Radix et Rhizoma	MOL004927	Hispaglabridin A	392.53	5.81	2	4	14.6	1.12	0.43	0.73	0	
721	Glycyrrhizae Radix et Rhizoma	MOL004928	Violanthin	578.57	-1.56	10	14	4.17	-2.01	-2.77	0.81	0	
722	Glycyrrhizae Radix et Rhizoma	MOL004929	Pentadecanol	228.47	5.99	1	1	13.73	1.3	1.06	0.06	0	
723	Glycyrrhizae Radix et Rhizoma	MOL004930	Uralenol	370.38	3.36	5	7	8.55	0.35	-0.57	0.46	0	
724	Glycyrrhizae Radix et Rhizoma	MOL004931	Uralenol-3-methylether	384.41	3.43	4	7	1.41	0.39	-0.62	0.49	0	
725	Glycyrrhizae Radix et Rhizoma	MOL004932	Glycyrrhizin	823.04	2.42	8	16	9.06	-2.23	-3.13	0.11	0	
726	Glycyrrhizae Radix et Rhizoma	MOL004933	Uralsaponin B	823.04	2.42	8	16	7.92	-2.6	-3.17	0.11	0	
727	Glycyrrhizae Radix et Rhizoma	MOL004934	Isohexane	86.2	2.9	0	0	56.13	1.77	2.08	0	0	11.03
728	Glycyrrhizae Radix et Rhizoma	MOL004935	Sigmoidin-B	356.4	3.89	4	6	34.88	0.42	-0.41	0.41	0	14.49
729	Glycyrrhizae Radix et Rhizoma	MOL004936	Uralene	384.41	3.43	4	7	11.7	0.63	-0.22	0.49	0	
730	Glycyrrhizae Radix et Rhizoma	MOL004937	Uralenneoside	286.26	-0.49	5	8	24.96	-1.01	-1.61	0.17	0	

731	Glycyrrhizae Radix et Rhizoma	MOL004938	Schaftoside	596.54	-1.5	10	16	7.88	-2.46	-3.48	0.75	0	
732	Glycyrrhizae Radix et Rhizoma	MOL004939	Nortangeretin	302.25	1.8	5	7	17.9	0.24	-0.68	0.27	0	
733	Glycyrrhizae Radix et Rhizoma	MOL004940	Neoliquiritin	418.43	0.66	5	9	13.01	-1.08	-1.95	0.71	0	
734	Glycyrrhizae Radix et Rhizoma	MOL004941	Liquiritigenin	256.27	2.57	2	4	71.12	0.41	-0.25	0.18	0	18.09
735	Glycyrrhizae Radix et Rhizoma	MOL004942	(E)-Dodec-2-ene	168.36	5.4	0	0	17.74	1.83	2	0.02	0	
736	Glycyrrhizae Radix et Rhizoma	MOL004943	Neoisoliquiritin	418.43	1	6	9	21.18	-1.41	-2.05	0.58	0	
737	Glycyrrhizae Radix et Rhizoma	MOL004944	1-Ethyl-1-cyclobutanol	100.18	1.32	1	1	93.23	1.13	1.35	0.02	0	11.08
738	Glycyrrhizae Radix et Rhizoma	MOL004945	Isobavachin	324.4	4.42	2	4	36.57	0.72	-0.04	0.32	0	17.95
739	Glycyrrhizae Radix et Rhizoma	MOL004946	2-Tetradecanone	212.42	4.99	0	1	17.71	1.46	1.6	0.05	0	
740	Glycyrrhizae Radix et Rhizoma	MOL004947	Isoviolanthin	578.57	-1.56	10	14	18.79	-2.43	-3.14	0.81	0	
741	Glycyrrhizae Radix et Rhizoma	MOL004948	Isoglycyrol	366.39	4.36	1	6	44.7	0.91	0.05	0.84	0	6.69
742	Glycyrrhizae Radix et Rhizoma	MOL004949	Isolicoflavonol	354.38	3.63	4	6	45.17	0.54	-0.42	0.42	0	15.55
743	Glycyrrhizae Radix et Rhizoma	MOL004950	Isoglycycoumarin	368.41	3.92	2	6	22.09	0.55	-0.16	0.6	0	
744	Glycyrrhizae Radix et Rhizoma	MOL004951	Isoliquiritin	418.43	1	6	9	8.61	-1.36	-1.93	0.6	0	
745	Glycyrrhizae Radix et Rhizoma	MOL004952	Licuraside	550.56	-0.41	8	13	5.25	-1.92	-3	0.77	0	
746	Glycyrrhizae Radix et Rhizoma	MOL004953	Liquiritin apioside	550.56	-0.75	7	13	29.23	-1.88	-2.69	0.82	0.31	
747	Glycyrrhizae Radix et Rhizoma	MOL004954	Isograbrol	392.53	6.28	2	4	11.04	0.87	-0.07	0.5	0	
748	Glycyrrhizae Radix et Rhizoma	MOL004955	Isoglabrolide	468.74	5.15	1	4	14.77	0.32	-0.16	0.62	0	
749	Glycyrrhizae Radix et Rhizoma	MOL004956	Isoononin	430.44	0.68	4	9	8.29	-1	-1.57	0.79	0	
750	Glycyrrhizae Radix et Rhizoma	MOL004957	Isoformononetin	268.28	2.58	1	4	38.37	0.79	0.25	0.21	0	16.56
751	Glycyrrhizae Radix et Rhizoma	MOL004958	Isoschaftoside	564.54	-1.94	10	14	17.38	-2.62	-3.36	0.83	0	
752	Glycyrrhizae Radix et Rhizoma	MOL004959	1-Methoxyphaseollidin	354.43	4.25	2	5	69.98	1.01	0.48	0.64	0	9.53
753	Glycyrrhizae Radix et Rhizoma	MOL004960	22β-Acetylglabric acid	528.8	4.77	2	6	17.76	-0.21	-0.98	0.64	0	
754	Glycyrrhizae Radix et Rhizoma	MOL004961	3,3'-Dimethylquercetin	330.31	1.82	3	7	46.45	0.39	-0.44	0.33	0	16.61
755	Glycyrrhizae Radix et Rhizoma	MOL004962	24-Hydroxy-11-deoxyglycyrrhetic acid	458.75	5.13	3	4	17.57	0.26	-0.36	0.76	0	
756	Glycyrrhizae Radix et Rhizoma	MOL004963	24-Hydroxyglycyrrhetic acid	486.76	4.4	3	5	24.17	-0.1	-0.8	0.72	0	
757	Glycyrrhizae Radix et Rhizoma	MOL004964	(Z)-1-(2,4-Dihydroxyphenyl)-3-phenylprop-2-en-1-one	240.27	3.17	2	3	73.18	0.57	0.14	0.12	0	20.35
758	Glycyrrhizae Radix et Rhizoma	MOL004965	3'(γ,γ-Dimethylallyl)-kievitone	424.53	5.63	4	6	1.21	0.51	-0.15	0.63	0	
759	Glycyrrhizae Radix et Rhizoma	MOL004966	3'-Hydroxy-4'-O-Methylglabridin	354.43	3.93	2	5	43.71	1	0.73	0.57	0	-0.61
760	Glycyrrhizae Radix et Rhizoma	MOL004967	3,3-Dimethylpentane	100.23	3.11	0	0	41.97	1.75	2.1	0.01	0	11.09
761	Glycyrrhizae Radix et Rhizoma	MOL004968	3,4,3',4'-Tetrahydroxy-2-methoxychalcone	288.32	3.19	4	5	1.33	0.64	0.01	0.2	0	
762	Glycyrrhizae Radix et Rhizoma	MOL004969	2-Ethyl-p-xylene	134.24	3.75	0	0	20.6	1.89	2.03	0.02	0	
763	Glycyrrhizae Radix et Rhizoma	MOL004970	3-Methylheptane	114.26	3.82	0	0	36.61	1.79	2.16	0.01	0	10.56
764	Glycyrrhizae Radix et Rhizoma	MOL004971	3-Methylhexane	100.23	3.36	0	0	38.19	1.78	2.15	0.01	0	10.9
765	Glycyrrhizae Radix et Rhizoma	MOL004972	3-Methylpentane	86.2	2.9	0	0	35.77	1.76	2.14	0	0	11.1
766	Glycyrrhizae Radix et Rhizoma	MOL004973	3-Ethylpentane	100.23	3.36	0	0	35.74	1.79	2.16	0.01	0	11.5
767	Glycyrrhizae Radix et Rhizoma	MOL004974	3'-Methoxyglabridin	354.43	3.93	2	5	46.16	0.94	0.47	0.57	0	0.52
768	Glycyrrhizae Radix et Rhizoma	MOL004975	3β-Formylglabrolide	496.75	5.33	0	5	16.36	0.26	-0.5	0.55	0	
769	Glycyrrhizae Radix et Rhizoma	MOL004976	Daidzein dimethyl ether	282.31	2.83	0	4	24.29	0.98	0.17	0.24	0	
770	Glycyrrhizae Radix et Rhizoma	MOL004977	1-Methoxyficifolinol	422.56	6.1	2	5	14.61	1.09	0.2	0.86	0	
771	Glycyrrhizae Radix et Rhizoma	MOL004978	4'-Methoxyglabridin	338.43	4.2	1	4	36.21	1.12	0.61	0.52	0	-0.13
772	Glycyrrhizae Radix et Rhizoma	MOL004979	4,2',4',alpha-Tetrahydroxydihydrochalcone	274.29	2.24	4	5	2.45	0.1	-0.4	0.16	0	
773	Glycyrrhizae Radix et Rhizoma	MOL004980	Inflacoumarin A	322.38	4.7	2	4	39.71	0.73	-0.24	0.33	0	2.31
774	Glycyrrhizae Radix et Rhizoma	MOL004981	Isobavachromene	322.38	3.96	2	4	5.2	0.86	-0.01	0.34	0	
775	Glycyrrhizae Radix et Rhizoma	MOL004982	2,6,10-Trimethyl-dodecane	144.14	-1.02	2	4	37.8	0.08	0.04	0.03	0	11.49
776	Glycyrrhizae Radix et Rhizoma	MOL004983	5,6,7,8-Tetrahydro-4-methylquinoline	147.24	2.69	0	1	59.18	1.63	1.79	0.04	0	11.39
777	Glycyrrhizae Radix et Rhizoma	MOL004985	Icos-5-enoic acid	310.58	7.75	1	2	30.7	1.22	1.09	0.2	0	5.28
778	Glycyrrhizae Radix et Rhizoma	MOL004986	6"-O-Acetylliquiritin	444.47	2.33	3	9	6.26	-0.48	-1.24	0.82	0	
779	Glycyrrhizae Radix et Rhizoma	MOL004987	11-Deoxyglycyrrhetic acid	456.78	6.42	2	3	16.21	0.51	-0.22	0.76	0	
780	Glycyrrhizae Radix et Rhizoma	MOL004988	Kanzonol F	420.54	5.3	1	5	32.47	1.18	0.56	0.89	0.28	9.98
781	Glycyrrhizae Radix et Rhizoma	MOL004989	6-Prenylated eriodictyol	356.4	3.89	4	6	39.22	0.4	-0.29	0.41	0	16.52
782	Glycyrrhizae Radix et Rhizoma	MOL004990	7,2',4'-Trihydroxy - 5-methoxy-3 - arylcoumarin	300.28	2.56	3	6	83.71	0.24	-0.59	0.27	0	0.99
783	Glycyrrhizae Radix et Rhizoma	MOL004991	7-Acetoxy-2-methylisoflavone	294.32	3.15	0	4	38.92	0.74	0.16	0.26	0	17.49
784	Glycyrrhizae Radix et Rhizoma	MOL004992	7-Hydroxy-2-methyl-3-phenyl-chromone	252.28	3.11	1	3	25.8	1	0.4	0.18	0	
785	Glycyrrhizae Radix et Rhizoma	MOL004993	8-Prenylated eriodictyol	356.4	3.89	4	6	53.79	0.43	-0.44	0.4	0	15.7
786	Glycyrrhizae Radix et Rhizoma	MOL004994	12-Methyltetradecanoate	256.48	5.96	0	2	17.36	1.35	1.15	0.09	0	
787	Glycyrrhizae Radix et Rhizoma	MOL004995	Kanzonol H	424.58	6.1	2	5	16.92	0.96	0.38	0.8	0.27	
788	Glycyrrhizae Radix et Rhizoma	MOL004996	Gadelaidic acid	310.58	7.75	1	2	30.7	1.2	0.94	0.2	0	5.25
789	Glycyrrhizae Radix et Rhizoma	MOL004997	Araboglycyrrhizin	779.03	2.72	7	14	17.73	-2.46	-2.73	0.14	0	
790	Glycyrrhizae Radix et Rhizoma	MOL004998	Araboglycyrrhizin_qt	470.76	5.49	2	4	17.71	0.11	-0.52	0.74	0	
791	Glycyrrhizae Radix et Rhizoma	MOL004999	Artonin E	436.49	4.67	4	7	11.38	0.34	-0.33	0.8	0	
792	Glycyrrhizae Radix et Rhizoma	MOL005000	Gancaonin G	352.41	4.17	2	5	60.44	0.78	0.23	0.39	0	16.13
793	Glycyrrhizae Radix et Rhizoma	MOL005001	Gancaonin H	420.49	4.71	3	6	50.1	0.6	-0.14	0.78	0	16.64
794	Glycyrrhizae Radix et Rhizoma	MOL005002	beta-Glycyrrhetinic acid	470.76	5.49	2	4	17.41	0.19	-0.4	0.74	0	
795	Glycyrrhizae Radix et Rhizoma	MOL005003	Licoagrocarpin	338.43	4.51	1	4	58.81	1.23	0.61	0.58	0.27	9.45
796	Glycyrrhizae Radix et Rhizoma	MOL005004	Gancaonin I	354.43	5.23	2	5	21.9	0.93	0.37	0.39	0	
797	Glycyrrhizae Radix et Rhizoma	MOL005005	Glyasperin A	422.51	5.48	4	6	2.46	0.6	-0.26	0.63	0	
798	Glycyrrhizae Radix et Rhizoma	MOL005006	Glyasperins K	368.46	4.54	1	5	10.15	0.74	0.22	0.44	0	
799	Glycyrrhizae Radix et Rhizoma	MOL005007	Glyasperins M	368.41	3.22	2	6	72.67	0.49	-0.04	0.59	0	15.57
800	Glycyrrhizae Radix et Rhizoma	MOL005008	Glycyrrhiza flavonol A	370.38	2.17	4	7	41.28	-0.09	-0.81	0.6	0	13.71
801	Glycyrrhizae Radix et Rhizoma	MOL005009	Corylifolinin	324.4	4.76	3	4	1.04	0.81	-0.03	0.27	0	
802	Glycyrrhizae Radix et Rhizoma	MOL005010	Kanzonol E	388.49	5.51	1	4	5.77	0.98	0.06	0.71	0	
803	Glycyrrhizae Radix et Rhizoma	MOL005011	Kanzonol Z	406.51	4.93	2	5	21.77	0.5	-0.18	0.76	0	
804	Glycyrrhizae Radix et Rhizoma	MOL005012	Licoagroisoflavone	336.36	3.48	2	5	57.28	0.71	0.09	0.49	0	19.64
805	Glycyrrhizae Radix et Rhizoma	MOL005013	18alpha-Hydroxyglycyrrhetic acid	486.76	4.55	3	5	41.16	-0.29	-0.78	0.71	0	4.96
806	Glycyrrhizae Radix et Rhizoma	MOL005014	Glycyrrhizae Radix et Rhizoma glycoside A	726.74	1.81	8	16	5.95	-2.37	-3.26	0.35	0.32	
807	Glycyrrhizae Radix et Rhizoma	MOL005015	Licoriisoflavan A	438.61	6.84	2	5	3.68	1.1	0.28	0.66	0	
808	Glycyrrhizae Radix et Rhizoma	MOL005016	Odoratin	314.31	2.3	2	6	49.95	0.42	-0.24	0.3	0	16.35
809	Glycyrrhizae Radix et Rhizoma	MOL005017	Phaseol	336.36	4.87	2	5	78.77	0.76	-0.06	0.58	0	9.64
810	Glycyrrhizae Radix et Rhizoma	MOL005018	Xambioona	388.49	4.68	0	4	54.85	1.09	0.52	0.87	0	14.5
811	Glycyrrhizae Radix et Rhizoma	MOL005019	(2R)-7-Hydroxy-2-[4-hydroxy-3-(3-methylbut-2-enyl)pheny	324.4	4.42	2	4	5.99	0.74	-0.06	0.33	0	
812	Glycyrrhizae Radix et Rhizoma	MOL005020	Dehydroglyasperins C	340.4	4.3	4	5	53.82	0.68	-0.12	0.37	0	2.75
813	Bupleuri Radix	MOL005021	Dimethyl benzene-1,2-dicarboxylate	194.2	1.54	0	4	57.4	0.64	0.63	0.06	0	5.56
813	Glycyrrhizae Radix et Rhizoma	MOL005021	Dimethyl benzene-1,2-dicarboxylate	194.2	1.54	0	4	57.4	0.64	0.63	0.06	0	5.56
814	Citri Unshius Pericarpium	MOL005100	Hesperetin	302.3	2.28	3	6	47.74	0.28	-0.3	0.27	0.31	16.51
815	Bupleuri Radix	MOL005120	16-Hexadecanal	240.48	6.42	0	1	16.54	1.44	1.28	0.08	0.18	
816	Angelicae Gigantis Radix	MOL005125	4-Methoxybenzoic acid	152.16	1.42	1	3	29.69	0.69	0.51	0.03	0.36	
817	Ginseng Radix	MOL005155	Ginsenoside Ro_qt	455.77	5.74	1	3	17.62	0.38	0.05	0.76	0.04	
818	Ginseng Radix	MOL005269	(+)-Maalioxide	222.41	3.47	0	1	55.93	1.59	1.93	0.13	0.22	16.64
819	Ginseng Radix	MOL005270	n-Heptadecanol	256.53	6.9	1	1	12.97	1.31	0.98	0.09	0.15	
820	Ginseng Radix	MOL005271	1-Hexadecyne	222.46	7.86	0	0	3.94	1.89	1.87	0.06	0.23	
821	Ginseng Radix	MOL005272	13-Tetradecenyl acetate	254.46	5.52	0	2	36.76	1.36	1.06	0.1	0.2	5.71
822	Ginseng Radix	MOL005273	16-Oxoseratenediol	456.78	5.52	2	3	15.1	0.4	-0.25	0.75	0.24	
823	Ginseng Radix	MOL005274	Neohexane	86.2	2.65	0	0	37.81	1.77	2.16	0.01	0.25	11.05
824	Ginseng Radix	MOL005275	2,3,4-Trimethyldecane	184.41	5.69	0	0	16.15	1.82	2.08	0.03	0.21	
825	Ginseng Radix	MOL005276	2,3,8-Trimethyldecane	184.41	5.69	0	0	5.51	1.79	1.95	0.03	0.22	
826	Ginseng Radix	MOL005277	2,6,10,15-Tetramethylheptadecane	296.65	9.13	0	0	13.73	1.85	1.86	0.13	0.19	
827	Ginseng Radix	MOL005278	2-Methyltridecane	198.44	6.55	0	0	5.75	1.81	1.9	0.04	0.18	
828	Ginseng Radix	MOL005279	Ginsenoside-Rh1	638.98	2.87	7	9	3.86	-1.17	-2.14	0.57	0.25	
829	Ginseng Radix	MOL005280	Ginsenoside-Rh1_qt	476.82	4.62	4	4	20.13	-0.02	-0.9	0.78	0.24	
830	Ginseng Radix	MOL005281	20(S)-Ginsenoside-Rh1	638.98	2.87	7	9	3.94	-1.1	-2.04	0.57	0.25	
831	Ginseng Radix	MOL005282	20(S)-Ginsenoside-Rh1_qt	476.82	4.62	4	4	20.13	0.17	-0.56	0.78	0.24	
832	Ginseng Radix	MOL005283	20(S)-Ginsenoside-Rg2	785.14	2.02	9	13	10.09	-1.94	-2.78	0.26	0.23	
833	Ginseng Radix	MOL005284	Protopanaxatriol	476.82	4.62	4	4	20.13	0.01	-0.82	0.78	0.25	
834	Ginseng Radix	MOL005285	20(s)-Protopanaxadiol	460.82	5.79	3	3	29.69	0.57	-0.07	0.77	0.23	
835	Ginseng Radix	MOL005286	20(R)-Ginsenoside Rg2	785.14	2.02	9	13	10.09	-1.96	-2.95	0.26	0.27	
836	Ginseng Radix	MOL005287	20-(S)-Ginsenoside-Rg3	785.14	2.3	9	13	13.69	-1.65	-2.47	0.22	0.24	
837	Ginseng Radix	MOL005288	20-(S)-Ginsenoside-Rg3_qt	460.82	5.79	3	3	29.69	0.55	-0.12	0.77	0.24	
838	Ginseng Radix	MOL005289	3,4-Dimethylheptane	128.29	4.07	0	0	46.67	1.78	2.05	0.01	0.24	11.02
839	Ginseng Radix	MOL005290	3,5-Dimethyl-p-anisic acid	180.22	2.39	1	3	61.11	0.75	0.56	0.05	0.34	2.11
840	Ginseng Radix	MOL005291	3-O-beta-D-Glucuronopyranosyl gypsogenin	646.9	4.05	5	10	8.68	-0.78	-1.37	0.32	0.27	
841	Ginseng Radix	MOL005292	3-O-beta-D-Glucuronopyranosyl gypsogenin_qt	470.76	5.59	2	4	23.72	0.22	-0.4	0.75	0.26	
842	Ginseng Radix	MOL005293	3-Ethyl-3-methylheptane	142.32	4.48	0	0	37.33	1.81	2.16	0.02	0.23	10.45

843	Ginseng Radix	MOL005294	3-Methylheptane	114.26	3.82	0	0	37.1	1.8	2.18	0.01	0.22	10.55
844	Ginseng Radix	MOL005295	3-Methylundecane	170.38	5.64	0	0	6.57	1.79	1.96	0.02	0.19	
845	Ginseng Radix	MOL005296	4-Methyldodecane	184.41	6.1	0	0	6.39	1.81	2.05	0.03	0.17	
846	Ginseng Radix	MOL005297	5-Isobutylnonane	184.41	5.89	0	0	6.08	1.82	2.1	0.03	0.2	
847	Ginseng Radix	MOL005298	5-Heptadec-12-enylresorcinol	346.61	8.64	2	2	3.29	1.38	0.79	0.32	0.25	
848	Ginseng Radix	MOL005299	5-Methyl-tetradecane	212.47	7.01	0	0	16.15	1.83	2.02	0.05	0.18	
849	Ginseng Radix	MOL005300	6'-Malonylginsenoside Rd1	1,195.51	-1.03	15	26	6.7	-4.21	-5.39	0.03	0.29	
850	Ginseng Radix	MOL005301	6'-Malonylginsenoside Rd1_qt1	460.82	5.79	3	3	29.69	0.56	-0.13	0.77	0.25	
851	Ginseng Radix	MOL005302	7-(beta-Xylosyl)Cephalomannine	962.15	1.97	6	18	27.33	-1.67	-2.25	0.17	0.32	
852	Ginseng Radix	MOL005303	7-Tetradecyne	194.4	6.44	0	0	20.07	1.93	1.81	0.04	0.19	
853	Ginseng Radix	MOL005304	7alpha-L-Rhamnosyl-6-methoxylutcolin	462.44	1.03	6	11	15.03	-0.69	-1.99	0.79	0.29	
854	Ginseng Radix	MOL005305	Nepetin	316.28	2.05	4	7	26.75	0.37	-0.78	0.31	0.27	
855	Ginseng Radix	MOL005306	Acetal	118.2	0.78	0	2	26.4	1.25	1.46	0.01	0.23	
856	Ginseng Radix	MOL005307	Adenosine triphosphate	491.22	-4.3	8	16	8.23	-3.1	-3.75	0.58	0.38	
857	Ginseng Radix	MOL005308	Aposiopolamine	271.34	1.39	1	4	66.65	0.66	0.4	0.22	0.35	3.54
858	Ginseng Radix	MOL005309	Araloside A	927.21	1.74	10	18	16.96	-3.32	-3.74	0.06	0.26	
859	Ginseng Radix	MOL005310	(4aS,6aR,6aS,6bR,8aR,10S,12aR,14bR)-10-Hydroxy-2,2,6a,6b	456.78	6.42	2	3	14.36	0.6	0.1	0.76	0.25	
860	Ginseng Radix	MOL005311	Argininyl-fructosyl-glucose	498.56	-4.63	13	16	0.74	-3.12	-3.75	0.62	0.25	
861	Ginseng Radix	MOL005312	Argininyl-fructosyl-glucose_qt	336.4	-2.88	10	11	11.25	-1.74	-2.3	0.22	0.25	
862	Ginseng Radix	MOL005313	Vitamin H	244.35	0.65	3	5	75.75	-0.04	-0.28	0.1	0.32	3.72
863	Ginseng Radix	MOL005314	Celabenzine	379.55	2.29	2	5	101.88	0.77	0.05	0.49	0.35	8.15
864	Ginseng Radix	MOL005315	(R)-Citronellal	154.28	3.02	0	1	50.78	1.37	1.61	0.02	0.28	5.35
865	Ginseng Radix	MOL005316	alpha-D-Mannopyranuronic Acid	194.16	-2.31	5	7	56.17	-2.14	-4.77	0.06	0.34	11.27
866	Ginseng Radix	MOL005317	Deoxyharringtonine	515.66	3.13	1	9	39.27	0.19	-0.25	0.81	0.23	7.9
867	Ginseng Radix	MOL005318	Dianthramine	289.26	2.05	5	7	40.45	-0.23	-0.97	0.2	0.42	5.14
868	Ginseng Radix	MOL005319	Ditertbutyl phthalate	278.38	3.4	0	4	43.67	1.13	1.13	0.13	0.33	-1.91
869	Ginseng Radix	MOL005320	Arachidonate	304.52	6.41	1	2	45.57	1.27	0.58	0.2	0.26	7.56
870	Ginseng Radix	MOL005321	Frutinone A	264.24	2.7	0	4	65.9	0.89	0.46	0.34	0.47	19.1
871	Ginseng Radix	MOL005322	gamma-Selinene	204.39	4.95	0	0	22.58	1.84	2.11	0.08	0.26	
872	Ginseng Radix	MOL005323	Ginsenoside La	783.12	1.84	8	13	17.74	-1.9	-3.25	0.14	0.24	
873	Ginseng Radix	MOL005324	Ginsenoside La_qt	458.8	5.33	2	3	15.7	0.65	0.04	0.78	0.24	
874	Ginseng Radix	MOL005325	Ginsenoside Ro	957.24	1.23	11	19	1.98	-2.86	-3.92	0.05	0.26	
875	Ginseng Radix	MOL005326	Ginsenoside-Ra0	1,271.62	-2.94	18	28	7.3	-5.56	-6.76	0.01	0.22	
876	Ginseng Radix	MOL005327	Gypnoside V_qt	460.82	5.79	3	3	29.69	0.3	-0.57	0.77	0.25	
877	Ginseng Radix	MOL005328	Ginsenoside-Ra1	1,211.56	-1.92	16	26	7.09	-4.51	-6.41	0.02	0.22	
878	Ginseng Radix	MOL005329	Ginsenoside-Ra2	1,211.56	-1.92	16	26	7.62	-4.43	-6.28	0.02	0.24	
879	Ginseng Radix	MOL005330	Ginsenoside-Ra3	1,241.59	-2.43	17	27	7.19	-4.67	-6.3	0.02	0.26	
880	Ginseng Radix	MOL005331	Ginsenoside Rb1	1,109.46	-1.2	15	23	6.24	-3.99	-5.6	0.04	0.23	
881	Ginseng Radix	MOL005332	Betulafolientriol	460.82	5.79	3	3	29.69	0.35	-0.54	0.77	0.25	
882	Ginseng Radix	MOL005333	Ginsenoside-Rb2	1,079.43	-0.69	14	22	6.02	-3.92	-5.43	0.04	0.26	
883	Ginseng Radix	MOL005334	(20S)-Protopanaxadiol	460.82	5.79	3	3	29.69	0.37	-0.52	0.77	0.24	
884	Ginseng Radix	MOL005335	Gypenoside LXIX	1,079.43	-0.69	14	22	7.73	-3.58	-5.38	0.04	0.24	
885	Ginseng Radix	MOL005336	Ginsenoside-Rc	1,079.43	-0.69	14	22	8.16	-3.97	-5.69	0.04	0.25	
886	Ginseng Radix	MOL005337	Ginsenoside Rd	947.3	0.55	12	18	5.5	-3.17	-4.47	0.09	0.24	
887	Ginseng Radix	MOL005338	Ginsenoside Re	947.3	0.27	12	18	4.27	-3.2	-4.39	0.12	0.25	
888	Ginseng Radix	MOL005340	20(R)-Protopanaxatriol	476.82	4.62	4	4	20.13	0.21	-0.43	0.78	0.24	
889	Ginseng Radix	MOL005341	Sanchinoside C1	801.14	1.13	10	14	10.04	-2.27	-3.5	0.28	0.24	
890	Ginseng Radix	MOL005342	Ginsenoside-Rg3	785.14	2.3	9	13	17.75	-2.02	-2.75	0.22	0.25	
891	Ginseng Radix	MOL005343	Ginsenoside-Rg3_qt	460.82	5.79	3	3	29.69	0.31	-0.58	0.77	0.26	
892	Ginseng Radix	MOL005344	Ginsenoside Rh2	622.98	4.04	6	8	36.32	-0.51	-1.38	0.56	0.24	11.08
893	Ginseng Radix	MOL005345	Ginsenoside-Rh3	604.96	5.01	5	7	12.09	-0.35	-1.11	0.59	0.25	
894	Ginseng Radix	MOL005346	Ginsenoside-Rh3_qt	442.8	6.76	2	2	13.09	0.97	0.42	0.76	0.26	
895	Ginseng Radix	MOL005347	Ginsenoside-Rh4	620.96	3.84	6	8	5.22	-0.73	-1.65	0.6	0.25	
896	Ginseng Radix	MOL005348	Ginsenoside-Rh4_qt	458.8	5.59	3	3	31.11	0.5	-0.18	0.78	0.25	6.97
897	Ginseng Radix	MOL005349	Ginsenoside-Rs1	1,121.47	-0.31	13	23	6.27	-3.69	-5.37	0.04	0.24	
898	Ginseng Radix	MOL005350	Ginsenoside-Rs2	1,121.47	-0.31	13	23	8.14	-4.03	-5.58	0.04	0.24	
899	Ginseng Radix	MOL005351	Ginsenoyne A	258.39	4.3	1	2	66.22	0.99	0.02	0.13	0.36	5.14
900	Ginseng Radix	MOL005352	Ginsenoyne B	294.85	4.74	2	2	39.79	0.79	-0.1	0.13	0.42	5.82
901	Ginseng Radix	MOL005353	Ginsenoyne C	276.41	3.53	3	3	43.38	0.15	-0.81	0.13	0.36	5.5
902	Ginseng Radix	MOL005354	Ginsenoyne D	262.43	4.94	1	2	19.32	1.07	0.28	0.13	0.31	
903	Ginseng Radix	MOL005355	Ginsenoyne E	258.39	4.93	0	2	36.53	1.05	0.46	0.13	0.37	6.72
904	Ginseng Radix	MOL005356	Girinimbin	263.36	4.6	1	1	61.22	1.72	1.22	0.31	0.33	8.17
905	Ginseng Radix	MOL005357	Gomisin B	514.62	2.73	1	9	31.99	0.6	0.18	0.83	0.19	7.81
906	Ginseng Radix	MOL005358	L-Erythro-isocitric acid	192.14	-1.34	4	7	32.95	-1.46	-1.71	0.04	0.41	11.65
907	Ginseng Radix	MOL005359	D-Erythro-Isocitric acid	192.14	-1.34	4	7	65.43	-1.67	-2.22	0.04	0.4	11.43
908	Ginseng Radix	MOL005360	Malkangunin	432.56	1.84	2	7	57.71	0.22	-0.17	0.63	0.3	4.09
909	Ginseng Radix	MOL005361	Malonylginsenoside Rc	1,165.48	-0.52	14	25	7.84	-4.27	-5.65	0.03	0.25	
910	Ginseng Radix	MOL005362	Malonylginsenoside Rc_qt1	460.82	5.79	3	3	29.69	0.45	-0.44	0.77	0.25	
911	Ginseng Radix	MOL005363	Malonylginsenoside Rd	1,033.35	0.71	12	21	8.84	-3.72	-4.76	0.07	0.28	
912	Ginseng Radix	MOL005364	Malonylginsenoside Rd_qt	460.82	5.79	3	3	29.69	0.47	-0.23	0.77	0.26	
913	Ginseng Radix	MOL005365	D-(+)-Maltose	342.34	-4.26	8	11	1.8	-2.7	-6.56	0.24	0.21	
914	Ginseng Radix	MOL005366	Malvic acid	280.5	6.44	1	2	30.99	1.22	0.81	0.15	0.21	3.74
915	Ginseng Radix	MOL005367	beta-D-Mannose	180.18	-2.51	5	6	43.04	-1.82	-4.46	0.04	0.26	11.1
916	Ginseng Radix	MOL005368	Methyl tricosanoate	368.72	9.81	0	2	14.61	1.43	1.08	0.33	0.16	
917	Ginseng Radix	MOL005369	Mycosinol	214.23	1.46	1	3	82.12	0.87	-0.05	0.09	0.42	5.67
918	Ginseng Radix	MOL005370	NN-Dimethyldecanamide	199.38	3.44	0	2	55.5	1.51	1.72	0.04	0.14	3.58
919	Ginseng Radix	MOL005371	Nonacosanediol-6,8	188.35	2.79	2	2	17.79	0.56	0.2	0.03	0.22	
920	Ginseng Radix	MOL005372	Notoginsenoside R2	771.11	1.64	9	13	17.74	-2.22	-3.12	0.28	0.25	
921	Ginseng Radix	MOL005373	Notoginsenoside R2_qt	476.82	4.62	4	4	20.13	0.2	-0.49	0.78	0.24	
922	Ginseng Radix	MOL005374	Notoginsenoside R6	963.3	-0.62	13	19	4.7	-3.46	-4.39	0.12	0.24	
923	Ginseng Radix	MOL005375	Stearyl acetate	312.6	7.74	0	2	16.27	1.39	1.06	0.19	0.15	
924	Ginseng Radix	MOL005376	Panaxadiol	460.82	5.46	2	3	33.09	0.82	0.23	0.79	0.22	6.34
925	Ginseng Radix	MOL005378	Panaxytriol	278.43	3.92	3	3	33.76	0.06	-0.98	0.13	0.33	3.74
926	Ginseng Radix	MOL005379	Pancratistatin	325.3	-1.94	6	9	13.13	-1.17	-1.79	0.46	0.32	
927	Ginseng Radix	MOL005380	Pandamine	552.79	3.87	4	9	16.15	0.11	-0.34	0.79	0.35	
928	Ginseng Radix	MOL005381	2-Formylpyrrole	95.11	0.97	1	1	41.58	1.12	1.44	0.01	0.14	4.34
929	Ginseng Radix	MOL005382	Ramalic acid	346.36	3.77	3	7	6	0.35	-0.13	0.3	0.33	
930	Ginseng Radix	MOL005383	Methylselenocysteine	182.1	-0.27	3	3	35.74	0.01	-0.13	0.01	0.43	11.69
931	Ginseng Radix	MOL005384	Suchilactone	368.41	3.73	0	6	57.52	0.82	0.28	0.56	0.28	9.03
932	Ginseng Radix	MOL005385	Suffruticoside A_qt1	302.26	-0.93	6	9	13.9	-1.35	-2.1	0.2	0.35	
933	Ginseng Radix	MOL005386	Vulgarin	264.35	1.31	1	4	29.21	0.03	-0.27	0.2	0.36	
934	Ginseng Radix	MOL005388	Undecane, 3,6-dimethyl	184.41	5.89	0	0	12.85	1.79	2.03	0.03	0.21	
935	Ginseng Radix	MOL005389	Pangamic acid	436.62	1.11	5	10	10.08	-0.92	-0.96	0.32	0.31	
936	Ginseng Radix	MOL005390	3-[[(2S)-2,4-Dihydroxy-3,3-dimethylbutanoyl]amino]propan	219.27	-0.98	4	6	21.29	-0.85	-1.25	0.06	0.29	
937	Ginseng Radix	MOL005391	(Z,Z)-alpha-Farnesene	204.39	5.46	0	0	8.47	1.92	1.85	0.05	0.36	
938	Ginseng Radix	MOL005392	alpha-Guttiferin	452.59	6.41	3	6	4.43	0.59	0.24	0.65	0.3	
939	Ginseng Radix	MOL005394	(Z)-2-Methyl-5-[(1S,2R,4R)-2-methyl-3-methylene-2-norbo	220.39	3.66	1	1	35.28	1.28	1.25	0.09	0.27	1.94
940	Ginseng Radix	MOL005396	cis-Widdrol alpha-epoxide	238.41	2.6	1	2	69.04	1.07	1	0.15	0.22	6.86
941	Ginseng Radix	MOL005397	Dammarane	414.84	9.53	0	0	19.73	1.81	1.81	0.7	0.22	
942	Astragali Radix	MOL005398	Daucosterol	576.95	6.34	4	6	20.63	-0.2	-0.81	0.63	0	
942	Ginseng Radix	MOL005398	Daucosterol	576.95	6.34	4	6	20.63	-0.1	-0.79	0.63	0.22	
943	Ginseng Radix	MOL005399	Daucosterol_qt	414.79	8.08	1	1	36.91	1.3	0.88	0.75	0.23	5.53
944	Ginseng Radix	MOL005400	Ginsenoside Rg5	767.12	3.31	8	12	6.15	-1.92	-2.83	0.23	0.26	
945	Ginseng Radix	MOL005401	Ginsenoside Rg5_qt	442.8	6.8	2	2	39.56	0.88	0.21	0.79	0.24	5.65
946	Ginseng Radix	MOL005402	Methyl margarate	284.54	7.08	0	2	17.41	1.37	1.25	0.14	0.17	
947	Ginseng Radix	MOL005403	Oleanane	412.82	8.66	0	0	6.69	1.83	1.92	0.76	0.22	
948	Ginseng Radix	MOL005404	p-Glucosyloxymandelonitrile	298.37	-0.47	5	6	12.12	-0.92	-1.61	0.18	0.27	
949	Angelicae Gigantis Radix	MOL005449	(2S)-2-Amino-4-methylsulfanylbutanoic acid	149.24	-0.27	3	3	70.87	0.06	-0.17	0.01	0.37	11.69
950	Cimicifugae Rhizoma	MOL005513	Dibenzofuran	168.2	3.33	0	1	37.28	1.78	1.62	0.08	0.35	11.77
951	Citri Unshius Pericarpium	MOL005577	Undecanal	170.33	4.13	0	1	22.9	1.38	1.41	0.03	0.2	
952	Angelicae Gigantis Radix	MOL005589	3,4-Dimethylbenzaldehyde	134.19	2.56	0	1	39.99	1.4	1.51	0.02	0.39	11.76
953	Angelicae Gigantis Radix	MOL005590	Ethylbenzaldehyde	134.19	2.53	0	1	40.95	1.4	1.62	0.02	0.37	4.59
954	Angelicae Gigantis Radix	MOL005608	2,3,5-Trimethylquinol	152.21	2.75	2	2	54.42	1.21	1.04	0.03	0.35	11.67

955	Bupleuri Radix	MOL005649	4-Methoxysalicyaldehyde	152.16	1.31	1	3	29.67	0.88	1.04	0.03	0.32	
956	Citri Unshius Pericarpium	MOL005811	Hepta-3	432.46	2.54	0	9	23.91	1.05	0.14	0.58	0.1	
957	Citri Unshius Pericarpium	MOL005812	Naringin	580.59	-0.46	8	14	6.92	-1.99	-3	0.78	0.31	
957	Glycyrrhizae Radix et Rhizoma	MOL005812	Naringin	580.59	-0.46	8	14	6.92	-1.99	-3	0.78	0.31	
958	Citri Unshius Pericarpium	MOL005813	Germacrene A	204.39	5.33	0	0	19.21	1.88	2.11	0.06	0.29	
959	Citri Unshius Pericarpium	MOL005814	Tangeretin	372.4	3.06	0	7	21.38	1.23	0.09	0.43	0.16	
960	Citri Unshius Pericarpium	MOL005815	Citromitin	404.45	3	0	8	86.9	0.88	0.16	0.51	0.14	15.62
961	Citri Unshius Pericarpium	MOL005816	alpha-Sinensal	218.37	4.63	0	1	57.79	1.59	1.4	0.06	0.36	4.46
962	Citri Unshius Pericarpium	MOL005817	2-(2-Butynyl)-cyclohexanone	150.24	2.68	0	1	47.78	1.44	1.48	0.03	0.3	5.57
963	Citri Unshius Pericarpium	MOL005818	2,5,5-Trimethylhepta-1,6-diene	138.28	3.69	0	0	44.34	1.81	2.07	0.02	0.33	10.87
964	Citri Unshius Pericarpium	MOL005819	2,6,11-Trimethyldodecane	212.47	6.6	0	0	14.19	1.83	1.88	0.05	0.2	
965	Citri Unshius Pericarpium	MOL005820	2,7-Dimethylocta-2,6-dien-1-ol	154.28	2.93	1	1	39.91	1.23	1.18	0.02	0.26	-0.97
966	Citri Unshius Pericarpium	MOL005821	(2S)-2-Ethoxypentane	116.23	2.11	0	1	39.6	1.55	1.94	0.01	0.21	10.91
967	Citri Unshius Pericarpium	MOL005822	3-Decyn-2-ol	154.28	3.44	1	1	39.33	1.17	1.12	0.02	0.24	4.45
968	Citri Unshius Pericarpium	MOL005823	Isoprenol	86.15	1.03	1	1	65.72	1	1.14	0	0.31	11.53
969	Citri Unshius Pericarpium	MOL005824	4-Acetylbenzoic acid	164.17	1.17	1	3	28.66	0.33	-0.17	0.04	0.46	
970	Citri Unshius Pericarpium	MOL005825	6-Hepten-1-ol	114.21	1.95	1	1	21.34	1.11	1.2	0.01	0.26	
971	Citri Unshius Pericarpium	MOL005826	cis-2,6-Dimethyl-2,6-octadiene	138.28	4.03	0	0	21.1	1.91	2.13	0.01	0.3	
972	Citri Unshius Pericarpium	MOL005827	Eufin	118.15	1.36	0	3	0.26	1.07	1.27	0.01	0.27	
973	Citri Unshius Pericarpium	MOL005828	Nobiletin	402.43	3.04	0	8	61.67	1.05	-0.08	0.52	0.13	16.2
974	Citri Unshius Pericarpium	MOL005829	Undecyl acetate	214.39	4.54	0	2	19.92	1.3	1.24	0.05	0.18	
975	Astragali Radix	MOL005928	Isoferulic acid	194.2	1.62	2	4	50.83	0.49	0.01	0.06	0	2.45
975	Cimicifugae Rhizoma	MOL005928	Isoferulic acid	194.2	1.62	2	4	50.83	0.49	0.01	0.06	0	2.45
976	Cimicifugae Rhizoma	MOL006218	Methyl caffeate	194.2	1.62	2	4	30.68	0.54	0.06	0.06	0.36	2.9
977	Bupleuri Radix	MOL006287	alpha-Muurolene	204.39	4.75	0	0	15.64	1.84	2.11	0.08	0.25	
978	Angelicae Gigantis Radix	MOL006440	Bicycloelemene	204.39	4.89	0	0	20.89	1.88	2.07	0.08	0.25	
979	Ginseng Radix	MOL006651	Trifolirhizin	446.44	0.54	4	10	7.62	-0.83	-1.58	0.79	0.28	
980	Bupleuri Radix	MOL006735	Furfuranol	98.11	0.62	1	2	48.27	0.82	0.91	0.01	0.13	4.26
981	Angelicae Gigantis Radix	MOL006869	1-(2,4-Dihydroxyphenyl)ethanone	152.16	1.03	2	3	36.49	0.67	0.46	0.03	0.4	25.1
981	Cimicifugae Rhizoma	MOL006869	1-(2,4-Dihydroxyphenyl)ethanone	152.16	1.03	2	3	36.49	0.67	0.46	0.03	0.4	25.1
982	Cimicifugae Rhizoma	MOL006931	Isolariciresino	360.44	2.25	4	6	6.96	0.11	-0.62	0.39	0.21	
983	Bupleuri Radix	MOL007260	Isorhamnetin-3-mono-beta-D-glucoside	478.44	-0.34	7	12	4.11	-1.35	-2.07	0.8	0.34	
984	Cimicifugae Rhizoma	MOL007330	Menthol	156.3	2.78	1	1	43.31	1.27	1.49	0.03	0.2	10.59
985	Ginseng Radix	MOL007500	Panaxatriol	476.82	4.29	3	4	15.42	0.52	-0.07	0.79	0.22	
986	Bupleuri Radix	MOL007553	(-)-beta-Fenchol	154.28	2.11	1	1	79.24	1.3	1.6	0.05	0.23	11.19
987	Bupleuri Radix	MOL007587	Azole	67.1	0.92	1	0	27.75	1.76	2.32	0	0.01	
988	Cimicifugae Rhizoma	MOL007724	Senecic acid	100.13	1.32	1	2	62.32	0.84	1.02	0.01	0.33	11.79
989	Angelicae Gigantis Radix	MOL007745	Phthalic acid	166.14	1.04	2	4	17.74	-0.05	-0.23	0.04	0.46	
990	Cimicifugae Rhizoma	MOL007777	Stigmasta-5,22-dien-3-O-.beta.-D-glucopyranoside	574.93	5.89	4	6	21.32	-0.06	-0.69	0.63	0.21	
991	Citri Unshius Pericarpium	MOL007930	Hesperidin	610.62	-0.48	8	15	13.33	-2.03	-2.7	0.67	0.31	
992	Bupleuri Radix	MOL007934	2-Hexanoylfuran	166.24	3	0	2	56.2	1.3	1.45	0.03	0.18	12.95
993	Angelicae Gigantis Radix	MOL008244	(1R,2S,4R)-1-Ethyl-1-methyl-2,4-bis(1-methylethyl)cyclohex	210.45	5.57	0	0	15.01	1.8	2.2	0.06	0.24	
994	Angelicae Gigantis Radix	MOL008245	Dodecene	168.36	5.46	0	0	17.74	1.8	2.05	0.02	0.22	
995	Angelicae Gigantis Radix	MOL008246	Phosphatdic acid	228.11	-1.55	2	8	19.32	-1.22	-1.66	0.05	0.42	
996	Angelicae Gigantis Radix	MOL008247	L-beta,gamma-Dimyristoyl-alpha-cephalin	635.97	9.49	3	9	20.69	-0.43	-1.8	0.47	0.21	
997	Angelicae Gigantis Radix	MOL008248	Phosphatidylinositol	390.27	-3.85	6	13	4.63	-2.34	-2.96	0.29	0.34	
998	Angelicae Gigantis Radix	MOL008249	Phosphatidylinositol_qt	228.11	-1.55	2	8	12.66	-1.15	-1.77	0.05	0	
999	Angelicae Gigantis Radix	MOL008250	Isobenzofuran-1,3-quinone	148.12	1.3	0	3	47.31	0.56	0.3	0.04	0.54	13.73
1000	Angelicae Gigantis Radix	MOL008251	Sedanolide	194.3	3.37	0	2	62.46	1.24	1.4	0.07	0.28	5.05
1001	Angelicae Gigantis Radix	MOL008252	Senkyunolide	192.28	3.19	0	2	68.28	1.28	1.4	0.07	0.31	5.49
1002	Angelicae Gigantis Radix	MOL008253	Sphingomyelin	493.73	3.18	3	7	0.31	-0.46	-1.31	0.51	0.19	
1003	Angelicae Gigantis Radix	MOL008254	Isotetandrine	622.82	7.22	0	8	10.42	0.95	0.28	0.1	0.21	
1004	Angelicae Gigantis Radix	MOL008255	alpha-Acoradiene	204.39	4.51	0	0	40.98	1.82	2.09	0.07	0.25	-0.9
1005	Angelicae Gigantis Radix	MOL008256	(-)-alpha-Cedrene	204.39	4.12	0	0	55.56	1.79	2.05	0.1	0.25	4.82
1006	Angelicae Gigantis Radix	MOL008257	alpha-Copaene	204.39	4.17	0	0	29.33	1.83	2.04	0.12	0.23	
1007	Angelicae Gigantis Radix	MOL008258	(1R,4R,5S)-4-Isopropenyl-1,8-dimethylspiro[4.5]dec-8-ene	204.39	4.75	0	0	40.65	1.83	2.1	0.07	0.26	-0.18
1008	Angelicae Gigantis Radix	MOL008259	2,6-Di(phenyl)thiopyran-4-thione	280.43	4.62	0	0	69.13	1.74	1.29	0.15	0.58	6.76
1009	Angelicae Gigantis Radix	MOL008260	O-Xylenol	122.18	2.54	1	1	53.13	1.62	1.88	0.02	0.37	11.92
1010	Angelicae Gigantis Radix	MOL008261	2,4,6-Trimethyl-Octane	156.35	4.78	0	0	29.14	1.81	2.2	0.02	0.25	
1011	Angelicae Gigantis Radix	MOL008262	Mesitaldehyde	148.22	3.05	0	1	37.8	1.54	1.58	0.03	0.37	11.63
1012	Angelicae Gigantis Radix	MOL008263	Isoxylaldehyde	134.19	2.56	0	1	38.85	1.39	1.66	0.02	0.4	11.74
1013	Angelicae Gigantis Radix	MOL008264	(E)-Octadec-3-ene	252.54	8.14	0	0	19.5	1.86	2.07	0.09	0.19	
1014	Angelicae Gigantis Radix	MOL008265	2-Valerylbenzoic acid	206.26	2.75	1	3	78.26	0.61	0.45	0.06	0.36	17.78
1015	Angelicae Gigantis Radix	MOL008266	(Z)-2-Hexenyl hexanoate	198.34	3.99	0	2	19.39	1.26	1.26	0.04	0.23	
1016	Angelicae Gigantis Radix	MOL008267	2-Methylhexadecanoic acid	270.51	6.83	1	2	20.23	1.07	0.87	0.11	0.2	
1017	Angelicae Gigantis Radix	MOL008268	cis-Isoeugenol	164.22	2.5	1	2	20.73	1.43	1.39	0.04	0.32	
1018	Angelicae Gigantis Radix	MOL008269	2-Methyl-5-decanone	310.63	7.99	0	1	20.4	1.42	1.32	0.11	0.25	
1019	Angelicae Gigantis Radix	MOL008270	2-Methyldodecan-5-one	198.39	4.54	0	1	13.16	1.43	1.39	0.04	0.2	
1020	Angelicae Gigantis Radix	MOL008271	3,7-Dimethyl-nonane	156.35	4.98	0	0	16.97	1.78	2.05	0.02	0.22	
1021	Angelicae Gigantis Radix	MOL008272	1,1,5-Trimethyl-2-formylcyclohexa-2,5-diene-4-one	164.22	1.64	0	2	48.94	0.82	0.65	0.04	0.36	11.76
1022	Angelicae Gigantis Radix	MOL008273	4-Methyl-6-hepten-3-one	126.22	2.07	0	1	78.38	1.39	1.7	0.01	0.32	11.01
1023	Angelicae Gigantis Radix	MOL008274	6-Ethylresorcinol	138.18	2.24	2	2	46.45	1.13	1.07	0.03	0.37	11.56
1024	Angelicae Gigantis Radix	MOL008275	5-Indolol	133.16	1.86	2	1	63.14	1.38	1.44	0.03	0.19	5.74
1025	Angelicae Gigantis Radix	MOL008276	Undecanol-6	172.35	4.15	1	1	25.77	1.19	1.14	0.02	0.2	
1026	Angelicae Gigantis Radix	MOL008277	7,10-Pentadecadiynoic acid	234.37	5.27	1	2	41.5	1.32	0.57	0.09	0.27	6.38
1027	Angelicae Gigantis Radix	MOL008278	4-Chloro-N-[1-methyl-5-[[1-methyl-5-[[1-methyl-5-(2-mor	762.38	2.93	5	13	7.18	-0.21	-1.98	0.31	0.17	
1028	Angelicae Gigantis Radix	MOL008279	Amyl ketone	170.33	3.83	0	1	18.65	1.31	1.48	0.02	0.21	
1029	Angelicae Gigantis Radix	MOL008280	Isoamylbenzene	148.27	3.94	0	0	35.69	1.84	2.05	0.03	0.34	5.89
1030	Angelicae Gigantis Radix	MOL008281	(Z)-2-[[(Z)-2-Methylbut-2-enoyl]oxymethyl]but-2-enoic acid	198.24	2.15	1	4	77.1	0.49	0.32	0.04	0.31	3.97
1031	Angelicae Gigantis Radix	MOL008282	Tropone	106.13	1.14	0	1	47.41	1.25	1.52	0.01	0.5	12.06
1032	Angelicae Gigantis Radix	MOL008283	Aromadendrene	204.39	4.22	0	0	18.21	1.83	2.02	0.1	0.24	
1033	Angelicae Gigantis Radix	MOL008284	Butanoic acid	88.12	0.89	1	2	21.62	0.69	0.98	0	0.34	
1034	Angelicae Gigantis Radix	MOL008285	(3S)-3-Butyl-3H-isobenzofuran-1-one	190.26	3.25	0	2	55.05	1.3	1.37	0.07	0.37	5.65
1035	Angelicae Gigantis Radix	MOL008286	Camphoric acid	200.26	1.48	2	4	99.13	0.1	0.13	0.07	0.34	12.15
1036	Angelicae Gigantis Radix	MOL008287	(3E)-3-Butylidene-7-hydroxy-2-benzofuran-1-one	204.24	2.74	1	3	42.17	1.03	0.94	0.08	0.38	4.75
1037	Angelicae Gigantis Radix	MOL008288	Coniferyl ferulate	356.4	3.64	2	6	4.54	0.71	-0.16	0.39	0.33	
1038	Angelicae Gigantis Radix	MOL008290	Lecithin	678.06	8.77	0	8	0.31	0.16	-1.1	0.4	0.02	
1039	Angelicae Gigantis Radix	MOL008291	2,4-Xylylaldehyde	134.19	2.56	0	1	39.33	1.42	1.71	0.03	0.41	11.73
1040	Angelicae Gigantis Radix	MOL008292	m-Ethylphenol	122.18	2.51	1	1	51.3	1.55	1.81	0.02	0.38	11.7
1041	Angelicae Gigantis Radix	MOL008293	1,5,5,6-Tetramethyl-1,3-Cyclohexadiene	136.26	3	0	0	39.22	1.82	2.18	0.03	0.29	11.69
1042	Bupleuri Radix	MOL008653	Acetylfuran	110.12	0.97	0	2	49.63	1.09	1.29	0.01	0.14	17.67
1043	Bupleuri Radix	MOL008661	Vinyl amyl ketone	126.22	2.49	0	1	19.82	1.38	1.54	0.01	0.31	
1044	Bupleuri Radix	MOL008672	2-Methylbutyraldehyde	86.15	1.4	0	1	54.16	1.24	1.67	0	0.28	11.36
1045	Bupleuri Radix	MOL010621	2,4-Octadienal	124.2	2.3	0	1	21.67	1.34	1.6	0.01	0.36	
1046	Ginseng Radix	MOL011400	Ginsenoside Rf	801.14	1.13	10	14	17.74	-2.23	-3.35	0.24	0.27	
1047	Ginseing Radix	MOL011401	ginsenoside- Rg1	801.14	1.13	10	14	9.03	-2.24	-3.41	0.28	0.26	
1048	Bupleuri Radix	MOL011598	Nerylacetone	194.35	3.62	0	1	45.53	1.46	1.58	0.04	0.31	6.54
1049	Bupleuri Radix	MOL011602	Pyridine	79.11	0.68	0	1	42.32	1.48	1.92	0.01	0.31	12.12
1050	Cimicifugae Rhizoma	MOL011734	Cimifugin	306.34	1.13	2	6	13.49	-0.09	-1.09	0.29	0.18	
1051	Cimicifugae Rhizoma	MOL011782	Ligustilide	190.26	2.94	0	2	23.5	1.28	1.2	0.07	0.32	
1052	Cimicifugae Rhizoma	MOL011985	12beta-Hydroxylcimigenol,3-o-alpha-l-arabinopyranoside	636.91	1.19	6	10	31.89	-1.02	-1.88	0.14	0.24	13.39
1053	Cimicifugae Rhizoma	MOL011986	12beta-Hydroxylcimigenol,3-o-alpha-l-arabinopyranoside_	504.78	2.43	4	6	17.71	-0.39	-0.96	0.38	0.23	
1054	Cimicifugae Rhizoma	MOL011987	15-O-Acetylacerinol	528.8	4.34	1	6	18.49	0.46	0.13	0.36	0.26	
1055	Cimicifugae Rhizoma	MOL011988	16alpha,24alpha-Dihydroxy-12beta-acetoxy-25,26,27-trinor	620.86	0.98	5	10	9.36	-1.41	-2.16	0.27	0.27	
1056	Cimicifugae Rhizoma	MOL011989	3H-1,3-Benzothiazole-2-thione	167.27	2.82	0	1	47.73	1.17	1.23	0.03	0.43	13.57
1057	Cimicifugae Rhizoma	MOL011990	23-Epi-26-deoxyactein	602.89	2.88	3	8	8.32	-0.2	-1.01	0.13	0.23	
1058	Cimicifugae Rhizoma	MOL011991	23-Epi-26-deoxyactein_qt	470.76	4.11	1	4	47.64	0.69	0.14	0.35	0.24	8.51
1059	Cimicifugae Rhizoma	MOL011992	23-O-Acetylcimigenol-3-o-beta-d-xylopyranoside	662.95	2.53	4	10	21.09	-0.8	-1.73	0.31	0.25	
1060	Cimicifugae Rhizoma	MOL011993	23-O-Acetylcimigenol-3-o-beta-d-xylopyranoside_qt	530.82	3.77	2	6	28.81	0.06	-0.55	0.69	0.26	
1061	Cimicifugae Rhizoma	MOL011994	23-O-Acetylshengmanol-3-o-alpha-l-arabinopyranoside	662.95	2.53	4	10	21.09	-1	-2.01	0.31	0.25	
1062	Cimicifugae Rhizoma	MOL011995	23-O-Acetylshengmanol-3-o-alpha-l-arabinopyranoside_qt	530.82	3.77	2	6	28.81	0.02	-0.52	0.69	0.25	
1063	Cimicifugae Rhizoma	MOL011996	24-O-Acetyl-7,8-didehydrocimigenol-3-β-D-xyloside	678.95	1.66	6	11	5.36	-1.03	-2.02	0.19	0.26	
1064	Cimicifugae Rhizoma	MOL011997	24-O-Acetyl-7,8-didehydrocimigenol-3-β-D-xyloside_qt	546.82	2.89	4	7	18.21	-0.36	-1	0.49	0.27	

1065	Cimicifugae Rhizoma	MOL011998	24-Epi-7,8-didehydrocimigenol	486.76	3.28	3	5	17.15	0.19	-0.37	0.4	0.24	
1066	Cimicifugae Rhizoma	MOL011999	24-Epi-acerinol	486.76	3.97	2	5	31.31	0.44	0.09	0.42	0.23	8.76
1067	Cimicifugae Rhizoma	MOL012000	24-Hydroxy-12beta-acetoxy-25,26,27-trinorcycloartan-16,2	620.86	1.1	4	10	21.89	-1.28	-1.98	0.45	0.27	
1068	Cimicifugae Rhizoma	MOL012001	24-Hydroxy-12beta-acetoxy-25,26,27-trinorcycloartan-16,2	488.73	2.33	2	6	29.39	-0.36	-0.84	0.79	0.26	
1069	Cimicifugae Rhizoma	MOL012002	24-O-Acetylcimigenol-3-o-beta-d-xylopyranoside	680.97	1.91	6	11	17.74	-1.26	-2.04	0.19	0.25	
1070	Cimicifugae Rhizoma	MOL012003	24-O-Acetylcimigenol-3-o-beta-d-xylopyranoside_qt	548.84	3.14	4	7	16.63	-0.07	-0.51	0.49	0.23	
1071	Cimicifugae Rhizoma	MOL012004	24R-O-Acetyl-7,8-didehydrocimigenol-3-β-D-araloside	678.95	1.66	6	11	6.06	-1.04	-1.93	0.19	0.26	
1072	Cimicifugae Rhizoma	MOL012005	25-O-Acetyl-7,8-didehydrocimigenol-3-O-α-L-araloside	660.93	2.42	4	10	19.49	-0.66	-1.53	0.12	0.25	
1073	Cimicifugae Rhizoma	MOL012006	25-O-Acetyl-7,8-didehydrocimigenol-3-β-D-xyloside_qt	528.8	3.66	2	6	22.43	0.22	-0.47	0.33	0.26	
1074	Cimicifugae Rhizoma	MOL012007	25-O-Acetyl-7,8-didehydrocimigenol-3-β-D-xyloside	660.93	2.42	4	10	40.41	-0.53	-1.45	0.12	0.25	13.97
1075	Cimicifugae Rhizoma	MOL012008	25-O-Acetylcimigenol-3-o-beta-d-galactoyranoside	692.98	2.16	5	11	21.32	-0.76	-1.51	0.1	0.2	
1076	Cimicifugae Rhizoma	MOL012009	25-O-Acetylcimigenol-3-o-beta-d-galactoyranoside_qt	530.82	3.91	2	6	19.35	0.14	-0.33	0.32	0.27	
1077	Cimicifugae Rhizoma	MOL012010	25-O-Acetylcimigenol-3-o-beta-d-glc(1-2)beta-d-xylopyra	825.11	0.93	7	15	18.5	-1.78	-2.52	0.04	0.22	
1078	Cimicifugae Rhizoma	MOL012011	25-O-Acetylcimigenol-3-o-beta-d-glc(1-2)beta-d-xylopyra	530.82	3.91	2	6	30.04	0.08	-0.64	0.32	0.26	8.95
1079	Cimicifugae Rhizoma	MOL012012	25-O-Acetylcimigenol	528.85	4.13	2	5	13.26	0.37	-0.3	0.32	0.22	
1080	Cimicifugae Rhizoma	MOL012013	25-O-Anhydrocimigenol-3-beta-d-xylopyranoside	602.89	3.32	4	8	4.99	-0.08	-0.79	0.16	0.25	
1081	Cimicifugae Rhizoma	MOL012014	25-O-Anhydrocimigenol-3-beta-d-xylopyranoside_qt	470.76	4.55	2	4	17.65	0.49	-0.03	0.43	0.26	
1082	Cimicifugae Rhizoma	MOL012015	26-Deoxyactein	660.93	2.15	3	10	20.17	-0.66	-1.42	0.11	0.23	
1083	Cimicifugae Rhizoma	MOL012016	26-Deoxyactein_qt	528.8	3.39	1	6	27.9	0.2	-0.5	0.29	0.26	
1084	Cimicifugae Rhizoma	MOL012017	3'-O-Acetyl-24-epi-7,8-didehydrocimigenol-3-O-β-D-xylos	660.93	2.42	4	10	17.95	-0.52	-1.33	0.11	0.26	
1085	Cimicifugae Rhizoma	MOL012018	3-Keto-24-epi-7,8-didehydrocimi genol	484.74	3.24	2	5	23.62	0.28	-0.1	0.4	0.26	
1086	Cimicifugae Rhizoma	MOL012019	4-O-Acetyl-caffeic acid	222.21	1.4	2	5	31.74	0.04	-0.43	0.08	0.39	0.06
1087	Cimicifugae Rhizoma	MOL012020	7,8-Didehydro-27-deoxyactein	658.91	1.9	3	10	25.28	-0.6	-1.39	0.11	0.25	
1088	Cimicifugae Rhizoma	MOL012021	7,8-Didehydro-27-deoxyactein_qt	526.78	3.14	1	6	17.77	0.34	-0.34	0.29	0.25	
1089	Cimicifugae Rhizoma	MOL012022	7,8-Didehydrocimigenol-3-O-β-D-xyloside	618.89	2.05	5	9	17.62	-0.37	-1.2	0.15	0.24	
1090	Cimicifugae Rhizoma	MOL012023	7,8-Didehydrocimigenol	486.76	3.28	3	5	36.79	0.14	-0.36	0.4	0.24	8.62
1091	Cimicifugae Rhizoma	MOL012024	Cimiside C	843.13	0.16	9	16	9.99	-1.96	-2.7	0.09	0.23	
1092	Cimicifugae Rhizoma	MOL012025	Cimiside D_qt	548.84	3.14	4	7	17.87	-0.51	-0.83	0.49	0.25	
1093	Cimicifugae Rhizoma	MOL012026	Cimiside D	843.13	0.16	9	16	11.01	-2.1	-2.79	0.09	0.23	
1094	Cimicifugae Rhizoma	MOL012027	Ferulic acid-β-D-glucoside	356.36	-0.29	5	9	25.23	-0.85	-1.31	0.32	0.28	
1095	Cimicifugae Rhizoma	MOL012028	Heracleifolinoside A	797.05	-0.65	9	15	5.87	-2.31	-3.37	0.16	0.24	
1096	Cimicifugae Rhizoma	MOL012029	Heracleifolinoside A_qt	502.76	2.33	4	6	16.41	-0.6	-1.03	0.77	0.28	
1097	Cimicifugae Rhizoma	MOL012030	Heracleifolinoside B	781.05	-0.11	8	14	42.31	-2.05	-3.02	0.17	0.23	14.52
1098	Cimicifugae Rhizoma	MOL012031	Heracleifolinoside B_qt	486.76	2.88	3	5	27.16	-0.27	-0.92	0.79	0.28	
1099	Cimicifugae Rhizoma	MOL012032	Heracleifolinoside C	799.07	-0.47	10	15	28.4	-1.98	-3.16	0.09	0.22	
1100	Cimicifugae Rhizoma	MOL012033	Heracleifolinoside C_qt	504.78	2.52	5	6	11.78	-0.43	-0.96	0.57	0.25	
1101	Cimicifugae Rhizoma	MOL012034	Heracleifolinoside D	799.07	-0.47	10	15	5.61	-2.4	-3.37	0.09	0.23	
1102	Cimicifugae Rhizoma	MOL012035	Heracleifolinoside D_qt	504.78	2.52	5	6	17.16	-0.63	-1.13	0.57	0.25	
1103	Cimicifugae Rhizoma	MOL012036	Heracleifolinoside E	841.11	-0.09	9	16	10.89	-1.77	-2.5	0.09	0.24	
1104	Cimicifugae Rhizoma	MOL012037	Heracleifolinoside E_qt	546.82	2.89	4	7	13.62	-0.6	-0.93	0.49	0.27	
1105	Cimicifugae Rhizoma	MOL012038	Heracleifolinoside F	797.05	-0.65	9	15	47.98	-2.46	-3.17	0.18	0.24	12.67
1106	Cimicifugae Rhizoma	MOL012039	Heracleifolinoside F_qt	502.76	2.33	4	6	20.64	-0.66	-1.19	0.77	0.28	
1107	Cimicifugae Rhizoma	MOL012040	Norkhelloside	526.49	-2.11	7	14	31.31	-1.86	-2.9	0.84	0.2	15.17
1108	Cimicifugae Rhizoma	MOL012041	Norkhelloside_qt	232.2	1.05	2	5	37.81	0.23	-0.52	0.16	0.19	10.86
1109	Cimicifugae Rhizoma	MOL012042	Acerinol	486.76	3.97	2	5	29.57	0.43	0.17	0.42	0.23	
1110	Cimicifugae Rhizoma	MOL012043	Actein	676.93	1.81	4	11	50.82	-1.11	-1.82	0.1	0.25	13.77
1111	Cimicifugae Rhizoma	MOL012044	Actein_qt	544.8	3.05	2	7	15.95	0.03	-0.65	0.26	0.27	
1112	Cimicifugae Rhizoma	MOL012045	Cimicifoetiside a	664.92	2.91	5	11	5.42	-0.91	-1.74	0.13	0.25	
1113	Cimicifugae Rhizoma	MOL012046	Cimicifoetiside a_qt	532.79	4.3	3	7	17.35	-0.07	-0.63	0.33	0.28	
1114	Cimicifugae Rhizoma	MOL012047	Cimicifoetiside b	706.96	3.29	4	12	5.59	-0.87	-1.76	0.1	0.24	
1115	Cimicifugae Rhizoma	MOL012048	Cimicifoetiside b_qt	574.83	4.68	2	8	15.51	0.12	-0.57	0.26	0.28	
1116	Cimicifugae Rhizoma	MOL012049	Cimicifugadine	601.86	1.95	6	9	8.21	-1.14	-2.04	0.25	0.25	
1117	Cimicifugae Rhizoma	MOL012050	Cimicifugadine_qt	469.73	3.19	4	5	21.19	-0.38	-1.19	0.63	0.27	
1118	Cimicifugae Rhizoma	MOL012051	Cimicifugamide	505.57	0.94	6	11	22.01	-0.76	-1.6	0.73	0.26	
1119	Cimicifugae Rhizoma	MOL012052	Tuberosine A	343.41	2.84	3	6	102.67	0.69	-0.03	0.34	0.28	4.53
1120	Cimicifugae Rhizoma	MOL012053	Cimicifugic acid	372.4	2.37	3	7	83.02	-0.11	-1.25	0.45	0.32	7.39
1121	Cimicifugae Rhizoma	MOL012054	Cimicifugoside	616.87	1.31	4	9	20.13	-1.15	-2.03	0.36	0.27	
1122	Cimicifugae Rhizoma	MOL012055	Cimicifugoside_qt	484.74	2.54	2	5	33.84	-0.08	-0.79	0.74	0.28	7.78
1123	Cimicifugae Rhizoma	MOL012056	Cimicifugoside h-1	620.91	2.3	5	9	10.57	-0.55	-1.37	0.15	0.24	
1124	Cimicifugae Rhizoma	MOL012057	Cimicifugoside h-1_qt	488.78	3.53	3	5	17.84	0.29	-0.18	0.4	0.24	
1125	Cimicifugae Rhizoma	MOL012058	Cimidahurine	316.34	-0.89	6	8	9.83	-1.15	-1.48	0.21	0.25	
1126	Cimicifugae Rhizoma	MOL012060	Cimigenol-3-O-B-D-xylopyranoside	620.91	2.3	5	9	17.77	-0.66	-1.46	0.15	0.23	
1127	Cimicifugae Rhizoma	MOL012061	Cimigenol-3-O-beta-d-galactopyranoside	650.94	1.78	6	10	4.47	-0.95	-1.71	0.12	0.24	
1128	Cimicifugae Rhizoma	MOL012062	Cimigenol	488.78	3.53	3	5	37.19	-0.16	-0.59	0.4	0.24	7.91
1129	Cimicifugae Rhizoma	MOL012063	Cimilactone a	590.83	2.36	3	9	8.46	-0.59	-1.4	0.31	0.25	
1130	Cimicifugae Rhizoma	MOL012064	Cimilactone a_qt	458.7	3.6	1	5	17.71	0.15	-0.62	0.68	0.26	
1131	Cimicifugae Rhizoma	MOL012065	Cimilactone b	588.81	2.11	3	9	11.45	-0.82	-1.57	0.31	0.29	
1132	Cimicifugae Rhizoma	MOL012066	Cimilactone b_qt	456.68	3.35	1	5	18.46	0.27	-0.36	0.68	0.26	
1133	Cimicifugae Rhizoma	MOL012067	Cimiside e	602.89	3.32	4	8	16.49	-0.3	-0.9	0.16	0.26	
1134	Cimicifugae Rhizoma	MOL012068	Cimiside e_qt	470.76	4.55	2	4	23.37	0.59	0.21	0.43	0.27	
1135	Cimicifugae Rhizoma	MOL012069	Dahurinol	488.78	3.53	3	5	17.32	-0.01	-0.49	0.6	0.27	
1136	Cimicifugae Rhizoma	MOL012070	Demethoxycimicifugamide	475.54	0.96	6	10	19.8	-0.74	-1.63	0.71	0.31	
1137	Cimicifugae Rhizoma	MOL012071	Heracleifolinol	546.82	3.58	3	7	9.77	-0.01	-0.43	0.51	0.27	
1138	Cimicifugae Rhizoma	MOL012072	Methylcimicifugoside	688.94	1.97	3	11	17.99	-0.55	-1.33	0.09	0.24	
1139	Cimicifugae Rhizoma	MOL012073	Methylcimicifugoside_qt	556.81	3.21	1	7	30.19	0.37	-0.29	0.24	0.23	11.92
1140	Cimicifugae Rhizoma	MOL012074	Neocimiside	576.8	0.35	6	9	17.64	-1.35	-2.05	0.31	0.26	
1141	Bupleuri Radix	MOL012297	Puerarin	416.41	-0.06	6	9	24.03	-1.15	-2.06	0.69	0.37	
1142	Cimicifugae Rhizoma	MOL013022	3,5-Octadien-2-one, (E,E)-	124.2	1.78	0	1	18.21	1.31	1.53	0.01	0.41	
1143	Bupleuri Radix	MOL013068	Oroxindin	459.41	0.21	4	11	7.07	-1.68	-2.15	0.77	0.04	
1144	Angelicae Gigantis Radix	MOL013077	Decursin	328.39	3.96	0	5	39.27	0.77	0.25	0.38	0.29	-1.77
1145	Angelicae Gigantis Radix	MOL013077	Decursinol angelate	328.39	3.96	0	5	39.27	0.77	0.25	0.38	0.29	-1.77
1146	Bupleuri Radix	MOL013187	Cubebin	356.4	3.19	1	6	57.13	0.47	-0.41	0.64	0.31	12.4
1147	Cimicifugae Rhizoma	MOL013222	m-Acetoxyanisole	166.19	1.58	0	3	23.26	1.01	0.99	0.04	0.28	
