# Supplementary material for: Protective Effect of Bojungikki-Tang against Radiation-Induced Intestinal Injury in Mice: Experimental Verification and Compound-Target Prediction
Source: Evid Based Complement Alternat Med. 2023 Jan 4;2023:5417813. doi: 10.1155/2023/5417813 (PMC9833920; doi:10.1155/2023/5417813)
Supplement: Supplementary Materials — See the Supplementary Tables (Tables 1–4). [file 5417813.f1.zip › Supple_Tables_2.pdf]

Supplementary Table 2. Active compounds in BJIK through ADME screening

No.	Herbs	Mol ID	Molecule Name	MW	AlogP	Hdon	Hacc	OB (%)	Caco-2	BBB	DL	FASA-	HL	Save
1	Atractylodis Rhizoma Alba	MOL000020	12-Senecioyl-2E,8E,10E-atractylentriol	312.39	2.5	0	4	62.4	0.01	-1.37	0.22	0.12	6.07	
2	Atractylodis Rhizoma Alba	MOL000021	14-Acetyl-12-senecioyl-2E,8E,10E-atractylentriol	355.44	3.21	0	5	60.31	0.33	-1.09	0.31	0.05	5.32	
3	Atractylodis Rhizoma Alba	MOL000022	14-Acetyl-12-senecioyl-2E,8Z,10E-atractylentriol	356.45	3.54	1	5	63.37	0.42	-1.14	0.3	0	6.43	
4	Atractylodis Rhizoma Alba	MOL000028	alpha-Amyrin	426.8	7.35	1	1	39.51	1.42	1.28	0.76	0	3.83	
5	Astragali Radix	MOL000033	(24S)-24-Propylcholesta-5-ene-3beta-ol	428.82	8.54	1	1	36.23	1.45	1.09	0.78	0	5.22	
5	Atractylodis Rhizoma Alba	MOL000033	(24S)-24-Propylcholesta-5-ene-3beta-ol	428.82	8.54	1	1	36.23	1.45	1.09	0.78	0	5.22	
6	Atractylodis Rhizoma Alba	MOL000049	3β-Acetoxyatractylone	274.39	3.39	0	3	54.07	1.13	1.08	0.22	0	-1.31	
7	Atractylodis Rhizoma Alba	MOL000072	8β-Ethoxy atractylenolide Ⅲ	276.41	3.68	0	3	35.95	1.08	1.12	0.21	0	8.34	
8	Astragali Radix	MOL000098	Quercetin	302.25	1.5	5	7	46.43	0.05	-0.77	0.28	0.38	14.4	
8	Bupleuri Radix	MOL000098	Quercetin	302.25	1.5	5	7	46.43	0.05	-0.77	0.28	0.38	14.4	
8	Glycyrrhizae Radix et Rhizoma	MOL000098	Quercetin	302.25	1.5	5	7	46.43	0.05	-0.77	0.28	0.38	14.4	
9	Astragali Radix	MOL000211	Mairin	456.78	6.52	2	3	55.38	0.73	0.22	0.78	0.26	8.87	
9	Glycyrrhizae Radix et Rhizoma	MOL000211	Mairin	456.78	6.52	2	3	55.38	0.73	0.22	0.78	0.26	8.87	
10	Astragali Radix	MOL000239	Jaranol	314.31	2.09	2	6	50.83	0.61	-0.22	0.29	0.29	15.5	
10	Glycyrrhizae Radix et Rhizoma	MOL000239	Jaranol	314.31	2.09	2	6	50.83	0.61	-0.22	0.29	0.29	15.5	
11	Astragali Radix	MOL000296	Hederagenin	414.79	8.08	1	1	36.91	1.32	0.96	0.75	0	5.35	
12	Astragali Radix	MOL000354	Isorhamnetin	316.28	1.76	4	7	49.6	0.31	-0.54	0.31	0.32	14.34	
12	Bupleuri Radix	MOL000354	Isorhamnetin	316.28	1.76	4	7	49.6	0.31	-0.54	0.31	0.32	14.34	
12	Glycyrrhizae Radix et Rhizoma	MOL000354	Isorhamnetin	316.28	1.76	4	7	49.6	0.31	-0.54	0.31	0.32	14.34	
13	Angelicae Gigantis Radix	MOL000358	beta-Sitosterol	414.79	8.08	1	1	36.91	1.32	0.99	0.75	0.23	5.36	
13	Ginseng Radix	MOL000358	beta-Sitosterol	414.79	8.08	1	1	36.91	1.32	0.99	0.75	0.23	5.36	
13	Cimicifugae Rhizoma	MOL000359	beta-Sitosterol	414.79	8.08	1	1	36.91	1.32	0.87	0.75	0.22	5.37	
13	Citri Unshius Pericarpium	MOL000359	beta-Sitosterol	414.79	8.08	1	1	36.91	1.32	0.87	0.75	0.22	5.37	
13	Glycyrrhizae Radix et Rhizoma	MOL000359	beta-Sitosterol	414.79	8.08	1	1	36.91	1.32	0.87	0.75	0.22	5.37	
14	Angelicae Gigantis Radix	MOL000360	trans-Ferulic Acid	194.2	1.62	2	4	39.56	0.47	-0.03	0.06	0.34	2.38	
14	Cimicifugae Rhizoma	MOL000360	trans-Ferulic Acid	194.2	1.62	2	4	39.56	0.47	-0.03	0.06	0.34	2.38	
15	Astragali Radix	MOL000371	3,9-di-O-Methylnissolin	314.36	2.89	0	5	53.74	1.18	0.63	0.48	0	9	
16	Astragali Radix	MOL000374	5'-Hydroxyiso-muronulatol-2',5'-di-O-glucoside	642.67	-0.95	9	16	41.72	-2.47	-3.62	0.69	0	2.52	
17	Astragali Radix	MOL000378	7-O-Methylisomucronulatol	316.38	3.38	1	5	74.69	1.08	0.84	0.3	0	2.98	
18	Astragali Radix	MOL000379	9-O-Methylnissolin 3-O-glucoside	462.49	0.74	4	10	36.74	-0.63	-1.5	0.92	0	13.06	
19	Astragali Radix	MOL000380	Astrapterocarpan	300.33	2.64	1	5	64.26	0.93	0.55	0.42	0	8.49	
20	Astragali Radix	MOL000387	Bifendate	418.38	2.56	0	10	31.1	0.15	-0.06	0.67	0	17.96	
21	Angelicae Gigantis Radix	MOL000389	cis-Ferulic acid	194.2	1.62	2	4	54.97	0.53	0.36	0.06	0	2.58	
21	Astragali Radix	MOL000389	cis-Ferulic acid	194.2	1.62	2	4	54.97	0.53	0.36	0.06	0	2.58	
22	Astragali Radix	MOL000392	Formononetin	268.28	2.58	1	4	69.67	0.78	0.02	0.21	0	17.04	
22	Glycyrrhizae Radix et Rhizoma	MOL000392	Formononetin	268.28	2.58	1	4	69.67	0.78	0.02	0.21	0	17.04	
23	Astragali Radix	MOL000398	Isoflavanone	316.33	2.42	2	6	109.99	0.53	0.17	0.3	0	15.51	
24	Astragali Radix	MOL000407	AstragalosideIV	785.09	-0.35	9	14	22.5	-2.11	-3.41	0.15	0		
25	Astragali Radix	MOL000417	Calycosin	284.28	2.32	2	5	47.75	0.52	-0.43	0.24	0	17.1	
25	Glycyrrhizae Radix et Rhizoma	MOL000417	Calycosin	284.28	2.32	2	5	47.75	0.52	-0.43	0.24	0	17.1	
26	Astragali Radix	MOL000422	Kaempferol	286.25	1.77	4	6	41.88	0.26	-0.55	0.24	0	14.74	
26	Bupleuri Radix	MOL000422	Kaempferol	286.25	1.77	4	6	41.88	0.26	-0.55	0.24	0	14.74	
26	Ginseng Radix	MOL000422	Kaempferol	286.25	1.77	4	6	41.88	0.26	-0.55	0.24	0	14.74	
26	Glycyrrhizae Radix et Rhizoma	MOL000422	Kaempferol	286.25	1.77	4	6	41.88	0.26	-0.55	0.24	0	14.74	
27	Astragali Radix	MOL000433	Folic acid	441.45	0.01	7	13	68.96	-1.5	-2.59	0.71	0	24.81	
28	Astragali Radix	MOL000438	Isomucronulatol	302.35	3.13	2	5	67.67	0.96	0.34	0.26	0	2.9	
29	Astragali Radix	MOL000439	Isomucronulatol-7,2'-di-O-glucosiole	626.67	-0.68	8	15	49.28	-2.22	-3.36	0.62	0	0.93	
30	Astragali Radix	MOL000442	1,7-Dihydroxy-3,9-dimethoxy pterocarpene	314.31	3.11	2	6	39.05	0.89	-0.04	0.48	0	7.95	
31	Angelicae Gigantis Radix	MOL000449	Stigmasterol	412.77	7.64	1	1	43.83	1.44	1	0.76	0.22	5.57	
31	Bupleuri Radix	MOL000449	Stigmasterol	412.77	7.64	1	1	43.83	1.44	1	0.76	0.22	5.57	
31	Cimicifugae Rhizoma	MOL000449	Stigmasterol	412.77	7.64	1	1	43.83	1.44	1	0.76	0.22	5.57	
31	Ginseng Radix	MOL000449	Stigmasterol	412.77	7.64	1	1	43.83	1.44	1	0.76	0.22	5.57	
32	Cimicifugae Rhizoma	MOL000483	Cis-N-Feruloyltyramine	313.38	2.86	3	5	118.35	0.51	-0.27	0.26	0	4.26	
33	Bupleuri Radix	MOL000490	Petunidin	317.29	1.65	5	7	30.05	0.16	-0.64	0.31	0	1.21	
34	Glycyrrhizae Radix et Rhizoma	MOL000497	Licochalcone a	338.43	4.62	2	4	40.79	0.82	-0.21	0.29	0	16.2	
35	Glycyrrhizae Radix et Rhizoma	MOL000500	Vestitol	272.32	3.15	2	4	74.66	0.86	0.3	0.21	0	3	
36	Ginseng Radix	MOL000787	Fumarine	353.4	2.95	0	6	59.26	0.56	-0.13	0.83	0.3	23.46	
37	Glycyrrhizae Radix et Rhizoma	MOL001484	Inermine	284.28	2.44	1	5	75.18	0.89	0.4	0.54	0.3	11.72	
38	Bupleuri Radix	MOL001645	Linoleyl acetate	308.56	6.85	0	2	42.1	1.36	1.08	0.2	0.21	7.48	
39	Glycyrrhizae Radix et Rhizoma	MOL001792	4',7-Dihydroxyflavanone	256.27	2.57	2	4	32.76	0.51	-0.29	0.18	0.42	17.89	
40	Glycyrrhizae Radix et Rhizoma	MOL002311	Glycyrol	366.39	4.85	2	6	90.78	0.71	-0.2	0.67	0.28	9.85	
41	Glycyrrhizae Radix et Rhizoma	MOL002565	Medicarpin	270.3	2.66	1	4	49.22	1	0.53	0.34	0.31	8.46	
42	Bupleuri Radix	MOL002776	Baicalin	446.39	0.64	6	11	40.12	-0.85	-1.74	0.75	0.36	17.36	
43	Glycyrrhizae Radix et Rhizoma	MOL002844	Pinocembrin	256.27	2.57	2	4	64.72	0.61	0.12	0.18	0.43	17.96	
44	Ginseng Radix	MOL002879	Diisooctyl 1,2-benzenedicarboxylate	390.62	7.44	0	4	43.59	0.79	0.26	0.39	0.28	3.6	
45	Ginseng Radix	MOL003648	Inermin	284.28	2.44	1	5	65.83	0.91	0.36	0.54	0.3	11.73	
46	Glycyrrhizae Radix et Rhizoma	MOL003656	Lupiwighteone	338.38	3.92	3	5	51.64	0.68	-0.23	0.37	0.36	15.63	
47	Glycyrrhizae Radix et Rhizoma	MOL003896	7-Methoxy-2-methyl isoflavone	266.31	3.36	0	3	42.56	1.16	0.56	0.2	0.33	16.89	
48	Citri Unshius Pericarpium	MOL004328	Naringenin	272.27	2.3	3	5	59.29	0.28	-0.37	0.21	0.4	16.98	
48	Glycyrrhizae Radix et Rhizoma	MOL004328	Naringenin	272.27	2.3	3	5	59.29	0.28	-0.37	0.21	0.4	16.98	
49	Ginseng Radix	MOL004492	Chrysanthemaxanthin	584.96	8.24	2	3	38.72	0.51	-0.98	0.58	0.3	17.47	
50	Bupleuri Radix	MOL004598	3',4',5',3,5,6,7-Heptamethoxyflavone	432.46	2.54	0	9	31.97	0.75	0.08	0.59	0.13	15.54	
51	Bupleuri Radix	MOL004609	Areapillin	360.34	2.29	3	8	48.96	0.6	-0.29	0.41	0.16	16.52	
52	Bupleuri Radix	MOL004624	Longikaurin A	348.48	1.16	3	5	47.72	0.08	0.09	0.53	0.27	1.71	
53	Bupleuri Radix	MOL004628	Octalupine	264.41	-0.07	1	4	47.82	0.48	0.3	0.28	0.17	4.17	
54	Bupleuri Radix	MOL004635	Saikosaponin a	781.1	1.11	8	13	32.39	-1.95	-2.93	0.09	0.25	15.37	
55	Bupleuri Radix	MOL004644	Sainfuran	286.3	3.38	2	5	79.91	0.9	0.23	0.23	0.22	8.58	
56	Bupleuri Radix	MOL004648	Troxerutin	346.56	5.89	3	3	31.6	0.35	-0.38	0.28	0.3	4.36	
57	Bupleuri Radix	MOL004653	(+)-Anomalin	426.5	5.05	0	7	46.06	0.46	0	0.66	0.36	1.03	
58	Bupleuri Radix	MOL004702	Saikosaponin c_qt	472.78	3.71	3	4	30.5	0.03	-0.85	0.63	0.2	6.12	
59	Bupleuri Radix	MOL004718	alpha-Spinasterol	412.77	7.64	1	1	42.98	1.28	0.79	0.76	0.22	6.46	
60	Angelicae Gigantis Radix	MOL004792	Nodakenin	408.44	0.28	4	9	57.12	-0.79	-1.42	0.69	0.27	7.16	
61	Glycyrrhizae Radix et Rhizoma	MOL004805	Shinflavanone	390.51	5.48	1	4	31.79	1	0.25	0.72	0.35	14.82	
62	Glycyrrhizae Radix et Rhizoma	MOL004806	Euchrenone	406.56	6.35	1	4	30.29	1.09	0.39	0.57	0	15.89	
63	Glycyrrhizae Radix et Rhizoma	MOL004808	Glyasperin B	370.43	4.02	3	6	65.22	0.47	-0.09	0.44	0	16.1	
64	Glycyrrhizae Radix et Rhizoma	MOL004810	Glyasperin F	354.38	2.97	3	6	75.84	0.43	-0.15	0.54	0	15.64	
65	Glycyrrhizae Radix et Rhizoma	MOL004811	Glyasperin C	356.45	4.73	3	5	45.56	0.71	0.07	0.4	0	3.13	
66	Glycyrrhizae Radix et Rhizoma	MOL004814	Isotrifoliol	298.26	2.99	2	6	31.94	0.53	-0.25	0.42	0	7.91	
67	Glycyrrhizae Radix et Rhizoma	MOL004815	Kanzonol B	322.38	3.96	2	4	39.62	0.66	-0.12	0.35	0	16.16	
68	Glycyrrhizae Radix et Rhizoma	MOL004820	Kanzonols W	336.36	3.63	2	5	50.48	0.63	0.04	0.52	0	0.15	
69	Glycyrrhizae Radix et Rhizoma	MOL004824	(2S)-6-(2,4-Dihydroxyphenyl)-2-(2-hydroxypropan-2-yl)-	384.41	2.96	3	7	60.25	0	-0.76	0.63	0	4.31	
70	Glycyrrhizae Radix et Rhizoma	MOL004827	Semilicoisoflavone B	352.36	2.85	3	6	48.78	0.45	-0.33	0.55	0	17.02	
71	Glycyrrhizae Radix et Rhizoma	MOL004828	Glepidotin A	338.38	3.9	3	5	44.72	0.79	0.06	0.35	0	16.09	
72	Glycyrrhizae Radix et Rhizoma	MOL004829	Glepidotin B	340.4	3.88	3	5	64.46	0.46	-0.09	0.34	0	15.98	
73	Glycyrrhizae Radix et Rhizoma	MOL004833	Phaseolinisoflavan	324.4	3.95	2	4	32.01	1.01	0.46	0.45	0	2.66	
74	Glycyrrhizae Radix et Rhizoma	MOL004835	Glypallichalcone	284.33	3.4	1	4	61.6	0.76	0.23	0.19	0	17.01	
75	Glycyrrhizae Radix et Rhizoma	MOL004838	Kanzonol U	308.35	4.2	2	4	58.44	1	0.34	0.38	0.34	8.71	
76	Glycyrrhizae Radix et Rhizoma	MOL004841	Licochalcone B	286.3	2.88	3	5	76.76	0.47	-0.46	0.19	0	17.02	
77	Glycyrrhizae Radix et Rhizoma	MOL004848	Licochalcone G	354.43	4.35	3	5	49.25	0.64	-0.04	0.32	0.35	15.75	
78	Glycyrrhizae Radix et Rhizoma	MOL004849	Licoarylcoumarin	368.41	4.03	3	6	59.62	0.4	-0.23	0.43	0	0.69	
79	Glycyrrhizae Radix et Rhizoma	MOL004855	Licoricone	382.44	4.16	2	6	63.58	0.53	-0.14	0.47	0	16.37	
80	Glycyrrhizae Radix et Rhizoma	MOL004856	Gancaonin A	352.41	4.17	2	5	51.08	0.8	0.13	0.4	0	16.82	
81	Glycyrrhizae Radix et Rhizoma	MOL004857	Gancaonin B	368.41	3.91	3	6	48.79	0.58	-0.1	0.45	0	16.49	
82	Glycyrrhizae Radix et Rhizoma	MOL004860	Glycyrrhizae Radix et Rhizoma glycoside E	693.71	1.59	7	14	32.89	-2.06	-2.8	0.27	0.31	25.39	
83	Glycyrrhizae Radix et Rhizoma	MOL004863	Gancaonin L	354.38	3.65	4	6	66.37	0.52	-0.13	0.41	0	15.81	
84	Glycyrrhizae Radix et Rhizoma	MOL004864	5,7-Dihydroxy-3-(4-methoxyphenyl)-8-(3-methylbut-2-e	352.41	4.17	2	5	30.49	0.9	0.21	0.41	0	14.99	

85	Glycyrrhizae Radix et Rhizoma	MOL004866	Gancaonin O	354.38	3.92	4	6	44.15	0.48	-0.28	0.41	0	16.77
86	Glycyrrhizae Radix et Rhizoma	MOL004879	Glycyrin	382.44	4.67	2	6	52.61	0.59	-0.13	0.47	0	1.31
87	Glycyrrhizae Radix et Rhizoma	MOL004882	Licocoumarone	340.4	4.98	3	5	33.21	0.84	0.06	0.36	0	9.66
88	Glycyrrhizae Radix et Rhizoma	MOL004883	Licoisoflavone	354.38	3.65	4	6	41.61	0.37	-0.27	0.42	0	16.09
89	Glycyrrhizae Radix et Rhizoma	MOL004884	Licoisoflavone B	352.36	2.85	3	6	38.93	0.46	-0.18	0.55	0	15.73
90	Glycyrrhizae Radix et Rhizoma	MOL004885	Licoisoflavanone	354.38	2.97	3	6	52.47	0.39	-0.22	0.54	0	15.67
91	Glycyrrhizae Radix et Rhizoma	MOL004891	Shinpterocarpin	322.38	3.46	1	4	80.3	1.1	0.68	0.73	0.32	6.5
92	Glycyrrhizae Radix et Rhizoma	MOL004898	2',3,4,4'-Tetrahydroxy-5-prenylchalcone	340.4	4.49	4	5	46.27	0.41	-0.4	0.31	0.43	15.24
93	Glycyrrhizae Radix et Rhizoma	MOL004903	Liquiritin	418.43	0.66	5	9	65.69	-1.06	-1.93	0.74	0	17.96
94	Glycyrrhizae Radix et Rhizoma	MOL004904	Licopyranocoumarin	384.41	3.04	3	7	80.36	0.13	-0.62	0.65	0	0.08
95	Glycyrrhizae Radix et Rhizoma	MOL004905	3,22-Dihydroxy-11-oxo-delta(12)-oleanene-27-alpha-me	512.75	4.37	1	6	34.32	-0.06	-0.75	0.55	0	3.56
96	Glycyrrhizae Radix et Rhizoma	MOL004907	Glyzaglabrin	298.26	2.1	2	6	61.07	0.34	-0.2	0.35	0	21.2
97	Glycyrrhizae Radix et Rhizoma	MOL004908	Glabridin	324.4	3.95	2	4	53.25	0.97	0.36	0.47	0	0.03
98	Glycyrrhizae Radix et Rhizoma	MOL004910	Glabranin	324.4	4.42	2	4	52.9	0.97	0.31	0.31	0	16.24
99	Glycyrrhizae Radix et Rhizoma	MOL004911	Glabrene	322.38	3.77	2	4	46.27	0.99	0.04	0.44	0	3.63
100	Glycyrrhizae Radix et Rhizoma	MOL004912	Glabrone	336.36	3.12	2	5	52.51	0.59	-0.11	0.5	0	16.09
101	Glycyrrhizae Radix et Rhizoma	MOL004913	Hedysarimcoumestan B	298.26	2.99	2	6	48.14	0.48	-0.19	0.43	0	8.87
102	Glycyrrhizae Radix et Rhizoma	MOL004914	1,3-Dihydroxy-8,9-dimethoxy-6-benzofurano[3,2-c]chro	328.29	2.98	2	7	62.9	0.4	-0.34	0.53	0	9.32
103	Glycyrrhizae Radix et Rhizoma	MOL004915	Eurycarpin A	338.38	3.92	3	5	43.28	0.43	-0.06	0.37	0	17.1
104	Glycyrrhizae Radix et Rhizoma	MOL004917	Glycyroside	562.57	-0.73	6	13	37.25	-1.58	-2.56	0.79	0	14.62
105	Glycyrrhizae Radix et Rhizoma	MOL004924	(-)-Medicocarpin	432.46	0.75	4	9	40.99	-0.6	-1.34	0.95	0	13.2
106	Glycyrrhizae Radix et Rhizoma	MOL004932	Glycyrrhizin	823.04	2.42	8	16	9.06	-2.23	-3.13	0.11	0	
107	Glycyrrhizae Radix et Rhizoma	MOL004935	Sigmoidin-B	356.4	3.89	4	6	34.88	0.42	-0.41	0.41	0	14.49
108	Glycyrrhizae Radix et Rhizoma	MOL004941	Liquiritigenin	256.27	2.57	2	4	71.12	0.41	-0.25	0.18	0	18.09
109	Glycyrrhizae Radix et Rhizoma	MOL004945	Isobavachin	324.4	4.42	2	4	36.57	0.72	-0.04	0.32	0	17.95
110	Glycyrrhizae Radix et Rhizoma	MOL004948	Isoglycyrol	366.39	4.36	1	6	44.7	0.91	0.05	0.84	0	6.69
111	Glycyrrhizae Radix et Rhizoma	MOL004949	Isolicoflavonol	354.38	3.63	4	6	45.17	0.54	-0.42	0.42	0	15.55
112	Glycyrrhizae Radix et Rhizoma	MOL004957	Isoformononetin	268.28	2.58	1	4	38.37	0.79	0.25	0.21	0	16.56
113	Glycyrrhizae Radix et Rhizoma	MOL004959	1-Methoxyphaseollidin	354.43	4.25	2	5	69.98	1.01	0.48	0.64	0	9.53
114	Glycyrrhizae Radix et Rhizoma	MOL004961	3,3'-Dimethylquercetin	330.31	1.82	3	7	46.45	0.39	-0.44	0.33	0	16.61
115	Glycyrrhizae Radix et Rhizoma	MOL004966	3'-Hydroxy-4'-O-Methylglabridin	354.43	3.93	2	5	43.71	1	0.73	0.57	0	-0.61
116	Glycyrrhizae Radix et Rhizoma	MOL004974	3'-Methoxyglabridin	354.43	3.93	2	5	46.16	0.94	0.47	0.57	0	0.52
117	Glycyrrhizae Radix et Rhizoma	MOL004978	4'-Methoxyglabridin	338.43	4.2	1	4	36.21	1.12	0.61	0.52	0	-0.13
118	Glycyrrhizae Radix et Rhizoma	MOL004980	Inflacoumarin A	322.38	4.7	2	4	39.71	0.73	-0.24	0.33	0	2.31
119	Glycyrrhizae Radix et Rhizoma	MOL004985	Icos-5-enoic acid	310.58	7.75	1	2	30.7	1.22	1.09	0.2	0	5.28
120	Glycyrrhizae Radix et Rhizoma	MOL004988	Kanzonol F	420.54	5.3	1	5	32.47	1.18	0.56	0.89	0.28	9.98
121	Glycyrrhizae Radix et Rhizoma	MOL004989	6-Prenylated eriodictyol	356.4	3.89	4	6	39.22	0.4	-0.29	0.41	0	16.52
122	Glycyrrhizae Radix et Rhizoma	MOL004990	7,2',4'-Trihydroxy - 5-methoxy-3 - arylcoumarin	300.28	2.56	3	6	83.71	0.24	-0.59	0.27	0	0.99
123	Glycyrrhizae Radix et Rhizoma	MOL004991	7-Acetoxy-2-methylisoflavone	294.32	3.15	0	4	38.92	0.74	0.16	0.26	0	17.49
124	Glycyrrhizae Radix et Rhizoma	MOL004993	8-Prenylated eriodictyol	356.4	3.89	4	6	53.79	0.43	-0.44	0.4	0	15.7
125	Glycyrrhizae Radix et Rhizoma	MOL004996	Gadelaidic acid	310.58	7.75	1	2	30.7	1.2	0.94	0.2	0	5.25
126	Glycyrrhizae Radix et Rhizoma	MOL005000	Gancaonin G	352.41	4.17	2	5	60.44	0.78	0.23	0.39	0	16.13
127	Glycyrrhizae Radix et Rhizoma	MOL005001	Gancaonin H	420.49	4.71	3	6	50.1	0.6	-0.14	0.78	0	16.64
128	Glycyrrhizae Radix et Rhizoma	MOL005003	Licoagrocarpin	338.43	4.51	1	4	58.81	1.23	0.61	0.58	0.27	9.45
129	Glycyrrhizae Radix et Rhizoma	MOL005007	Glyasperins M	368.41	3.22	2	6	72.67	0.49	-0.04	0.59	0	15.57
130	Glycyrrhizae Radix et Rhizoma	MOL005008	Glycyrrhiza flavonol A	370.38	2.17	4	7	41.28	-0.09	-0.81	0.6	0	13.71
131	Glycyrrhizae Radix et Rhizoma	MOL005012	Licoagroisoflavone	336.36	3.48	2	5	57.28	0.71	0.09	0.49	0	19.64
132	Glycyrrhizae Radix et Rhizoma	MOL005013	18alpha-Hydroxyglycyrrhetic acid	486.76	4.55	3	5	41.16	-0.29	-0.78	0.71	0	4.96
133	Glycyrrhizae Radix et Rhizoma	MOL005016	Odoratin	314.31	2.3	2	6	49.95	0.42	-0.24	0.3	0	16.35
134	Glycyrrhizae Radix et Rhizoma	MOL005017	Phaseol	336.36	4.87	2	5	78.77	0.76	-0.06	0.58	0	9.64
135	Glycyrrhizae Radix et Rhizoma	MOL005018	Xambioona	388.49	4.68	0	4	54.85	1.09	0.52	0.87	0	14.5
136	Glycyrrhizae Radix et Rhizoma	MOL005020	Dehydroglyasperins C	340.4	4.3	4	5	53.82	0.68	-0.12	0.37	0	2.75
137	Citri Unshius Pericarpium	MOL005100	Hesperetin	302.3	2.28	3	6	47.74	0.28	-0.3	0.27	0.31	16.51
138	Ginseng Radix	MOL005308	Aposiopolamine	271.34	1.39	1	4	66.65	0.66	0.4	0.22	0.35	3.54
139	Ginseng Radix	MOL005314	Celabenzine	379.55	2.29	2	5	101.88	0.77	0.05	0.49	0.35	8.15
140	Ginseng Radix	MOL005317	Deoxyharringtonine	515.66	3.13	1	9	39.27	0.19	-0.25	0.81	0.23	7.9
141	Ginseng Radix	MOL005318	Dianthramine	289.26	2.05	5	7	40.45	-0.23	-0.97	0.2	0.42	5.14
142	Ginseng Radix	MOL005320	Arachidonate	304.52	6.41	1	2	45.57	1.27	0.58	0.2	0.26	7.56
143	Ginseng Radix	MOL005321	Frutinone A	264.24	2.7	0	4	65.9	0.89	0.46	0.34	0.47	19.1
144	Ginseng Radix	MOL005331	Ginsenoside Rb1	1,109.46	-1.2	15	23	6.24	-3.99	-5.6	0.04	0.23	
145	Ginseng Radix	MOL005338	Ginsenoside Re	947.3	0.27	12	18	4.27	-3.2	-4.39	0.12	0.25	
146	Ginseng Radix	MOL005344	Ginsenoside Rh2	622.98	4.04	6	8	36.32	-0.51	-1.38	0.56	0.24	11.08
147	Ginseng Radix	MOL005348	Ginsenoside-Rh4_qt	458.8	5.59	3	3	31.11	0.5	-0.18	0.78	0.25	6.97
148	Ginseng Radix	MOL005356	Girinimbin	263.36	4.6	1	1	61.22	1.72	1.22	0.31	0.33	8.17
149	Ginseng Radix	MOL005357	Gomisin B	514.62	2.73	1	9	31.99	0.6	0.18	0.83	0.19	7.81
150	Ginseng Radix	MOL005360	Malkangunin	432.56	1.84	2	7	57.71	0.22	-0.17	0.63	0.3	4.09
151	Ginseng Radix	MOL005376	Panaxadiol	460.82	5.46	2	3	33.09	0.82	0.23	0.79	0.22	6.34
152	Ginseng Radix	MOL005384	Suchilactone	368.41	3.73	0	6	57.52	0.82	0.28	0.56	0.28	9.03
153	Ginseng Radix	MOL005399	Daucosterol_qt	414.79	8.08	1	1	36.91	1.3	0.88	0.75	0.23	5.53
154	Ginseng Radix	MOL005401	Ginsenoside Rg5_qt	442.8	6.8	2	2	39.56	0.88	0.21	0.79	0.24	5.65
155	Citri Unshius Pericarpium	MOL005815	Citromitin	404.45	3	0	8	86.9	0.88	0.16	0.51	0.14	15.62
156	Citri Unshius Pericarpium	MOL005828	Nobiletin	402.43	3.04	0	8	61.67	1.05	-0.08	0.52	0.13	16.2
157	Astragali Radix	MOL005928	Isoferulic acid	194.2	1.62	2	4	50.83	0.49	0.01	0.06	0	2.45
157	Cimicifugae Rhizoma	MOL005928	Isoferulic acid	194.2	1.62	2	4	50.83	0.49	0.01	0.06	0	2.45
158	Citri Unshius Pericarpium	MOL007930	Hesperidin	610.62	-0.48	8	15	13.33	-2.03	-2.7	0.67	0.31	
159	Ginseing Radix	MOL011401	ginsenoside- Rg1	801.14	1.13	10	14	9.03	-2.24	-3.41	0.28	0.26	
160	Cimicifugae Rhizoma	MOL011991	23-Epi-26-deoxyactein_qt	470.76	4.11	1	4	47.64	0.69	0.14	0.35	0.24	8.51
161	Cimicifugae Rhizoma	MOL011999	24-Epi-acerinol	486.76	3.97	2	5	31.31	0.44	0.09	0.42	0.23	8.76
162	Cimicifugae Rhizoma	MOL012011	25-O-Acetylcimigenol-3-o-beta-d-glc(1-2)beta-d-xylop	530.82	3.91	2	6	30.04	0.08	-0.64	0.32	0.26	8.95
163	Cimicifugae Rhizoma	MOL012023	7,8-Didehydrocimigenol	486.76	3.28	3	5	36.79	0.14	-0.36	0.4	0.24	8.62
164	Cimicifugae Rhizoma	MOL012038	Heracleifolinoside F	797.05	-0.65	9	15	47.98	-2.46	-3.17	0.18	0.24	12.67
165	Cimicifugae Rhizoma	MOL012040	Norkhelloside	526.49	-2.11	7	14	31.31	-1.86	-2.9	0.84	0.2	15.17
166	Cimicifugae Rhizoma	MOL012052	Tuberosine A	343.41	2.84	3	6	102.67	0.69	-0.03	0.34	0.28	4.53
167	Cimicifugae Rhizoma	MOL012053	Cimicifugic acid	372.4	2.37	3	7	83.02	-0.11	-1.25	0.45	0.32	7.39
168	Cimicifugae Rhizoma	MOL012055	Cimicifugoside_qt	484.74	2.54	2	5	33.84	-0.08	-0.79	0.74	0.28	7.78
169	Cimicifugae Rhizoma	MOL012062	Cimigenol	488.78	3.53	3	5	37.19	-0.16	-0.59	0.4	0.24	7.91
170	Cimicifugae Rhizoma	MOL012073	Methylcimicifugoside_qt	556.81	3.21	1	7	30.19	0.37	-0.29	0.24	0.23	11.92
171	Angelicae Gigantis Radix	MOL013077	Decursin	328.39	3.96	0	5	39.27	0.77	0.25	0.38	0.29	-1.77
172	Angelicae Gigantis Radix	MOL013077	Decursinol angelate	328.39	3.96	0	5	39.27	0.77	0.25	0.38	0.29	-1.77
173	Bupleuri Radix	MOL013187	Cubebin	356.4	3.19	1	6	57.13	0.47	-0.41	0.64	0.31	12.4
