# Supplementary material for: Protective Effect of Bojungikki-Tang against Radiation-Induced Intestinal Injury in Mice: Experimental Verification and Compound-Target Prediction
Source: Evid Based Complement Alternat Med. 2023 Jan 4;2023:5417813. doi: 10.1155/2023/5417813 (PMC9833920; doi:10.1155/2023/5417813)
Supplement: Supplementary Materials — See the Supplementary Tables (Tables 1–4). [file 5417813.f1.zip › Supple_Tables_3.pdf]

Supplementary Table 3. Interactions between active compounds and genes based on STITCH DB with combined score ≥0.400

No.	Genes	Active compounds	Score
1	ABCA1	Arachidonate	0.911
	ABCA1	beta-Sitosterol	0.614
	ABCA1	Quercetin	0.853
	ABCA1	Stigmasterol	0.88
2	ABCB1	Isorhamnetin	0.7
	ABCB1	Kaempferol	0.752
	ABCB1	Naringenin	0.825
	ABCB1	Quercetin	0.953
3	ABCB11	beta-Sitosterol	0.815
4	ABCB4	Arachidonate	0.9
5	ABCC1	Isorhamnetin	0.715
	ABCC1	Kaempferol	0.725
	ABCC1	Nobiletin	0.8
	ABCC1	Quercetin	0.75
6	ABCC4	Quercetin	0.7
7	ABCC5	Quercetin	0.7
8	ABCG1	beta-Sitosterol	0.529
9	ABCG2	Folic acid	0.478
	ABCG2	Naringenin	0.402
	ABCG2	Quercetin	0.662
10	ABCG5	beta-Sitosterol	0.879
	ABCG5	Stigmasterol	0.711
11	ABCG8	beta-Sitosterol	0.92
	ABCG8	Stigmasterol	0.811
12	ABHD12	Ferulic acid	0.543
13	ABHD12B	Ferulic acid	0.543
14	ABHD13	Ferulic acid	0.543
15	ABL1	Quercetin	0.505
16	ABL2	Quercetin	0.505
17	ACACA	Arachidonate	0.8
	ACACA	Quercetin	0.7
18	ACADM	Arachidonate	0.908
19	ACE	Astragaloside IV	0.8
20	ACO1	Isoferulic acid	0.984
21	ACO2	Isoferulic acid	0.729
22	ACOT1	Arachidonate	0.8
	ACOT1	Ferulic acid	0.543
23	ACOT2	Arachidonate	0.8
	ACOT2	Ferulic acid	0.543
24	ACOT4	Arachidonate	0.8
	ACOT4	Ferulic acid	0.543
25	ACOT6	Ferulic acid	0.543
26	ACOT7	Arachidonate	0.8
27	ACP1	Folic acid	0.818
28	ACSL1	Arachidonate	0.929

29	ACSL4	Arachidonate	0.946
30	ADIPOQ	Hesperetin	0.824
	ADIPOQ	Naringenin	0.8
	ADIPOQ	Quercetin	0.824
31	AGT	Arachidonate	0.915
	AGT	Pinocembrin	0.786
32	AHR	Kaempferol	0.872
	AHR	Quercetin	0.711
33	AIFM1	Quercetin	0.8
34	AKR1B1	Quercetin	0.804
35	AKR1C3	Quercetin	0.745
36	AKT1	Astragaloside IV	0.8
	AKT1	Ginsenoside Rb1	0.945
	AKT1	Isorhamnetin	0.8
	AKT1	Kaempferol	0.856
	AKT1	Mairin	0.725
	AKT1	Quercetin	0.957
37	ALAS1	Arachidonate	0.9
38	ALB	Arachidonate	0.884
	ALB	Folic acid	0.473
39	ALDH1L1	Folic acid	0.834
40	ALOX12	Arachidonate	0.992
	ALOX12	Kaempferol	0.724
	ALOX12	Quercetin	0.879
41	ALOX12B	Arachidonate	0.949
42	ALOX15	Arachidonate	0.987
	ALOX15	Quercetin	0.482
43	ALOX15B	Arachidonate	0.988
44	ALOX5	Arachidonate	0.999
	ALOX5	Baicalin	0.818
	ALOX5	Quercetin	0.638
45	ALOX5AP	Arachidonate	0.964
46	ALOXE3	Arachidonate	0.444
47	ANGPT2	Decursin	0.8
48	ANGPTL4	Arachidonate	0.9
49	ANKRD1	Arachidonate	0.9
50	ANXA1	Arachidonate	0.426
51	AOAH	Arachidonate	0.411
52	AOX1	Quercetin	0.892
53	APAF1	Quercetin	0.845
54	APOA1	Arachidonate	0.98
55	APOA2	Arachidonate	0.9
56	APOA5	Arachidonate	0.9
57	APOB	Naringenin	0.822
	APOB	Nobiletin	0.786
	APOB	Quercetin	0.727
58	APOE	Arachidonate	0.403
	APOE	beta-Sitosterol	0.872

	APOE	Folic acid	0.443
59	AR	Quercetin	0.943
	AR	Decursin	0.725
60	ARNT	Quercetin	0.7
61	ARNTL	Arachidonate	0.9
62	ATF1	Ginsenoside Rb1	0.534
63	ATF2	Arachidonate	0.7
64	ATM	Kaempferol	0.7
65	ATP2A1	Quercetin	0.824
66	ATP5A1	Quercetin	0.957
67	ATP5B	Quercetin	0.961
68	ATP5C1	Quercetin	0.957
69	ATP6V1H	alpha-Spinasterol	0.544
70	ATR	Quercetin	0.582
71	AXL	Quercetin	0.519
72	bA251O17.4	Folic acid	0.726
73	BAAT	Arachidonate	0.8
	BAAT	Ferulic acid	0.543
74	BAK1	Quercetin	0.444
75	BAX	Quercetin	0.734
76	BCHE	Quercetin	0.409
77	BCL2	Quercetin	0.849
78	BCL2A1	Ginsenoside Rh2	0.8
	BCL2A1	Quercetin	0.444
79	BCL2L1	Quercetin	0.584
80	BCL2L14	Quercetin	0.444
81	BCL6	Hesperidin	0.8
82	BDH2	Glycyrrhizin	0.427
	BDNF	Folic acid	0.826
83	BDNF	Ginsenoside Rg1	0.819
	BDNF	Naringenin	0.8
	BDNF	Quercetin	0.815
84	BECN1	Baicalin	0.832
85	BHMT	Folic acid	0.439
86	BID	Arachidonate	0.7
	BID	Quercetin	0.7
87	BIRC3	Folic acid	0.8
88	BIRC5	Mairin	0.724
89	BLVRB	Calycosin	0.845
90	BOK	Quercetin	0.444
91	C2orf81	Glycyrrhizin	0.427
92	CARM1	Arachidonate	0.9
93	CASP10	Baicalin	0.712
	CASP3	Arachidonate	0.808
	CASP3	Baicalin	0.829
	CASP3	beta-Sitosterol	0.818
	CASP3	Formononetin	0.7
	CASP3	Ginsenoside Rb1	0.839

94	CASP3	Hesperidin	0.818
	CASP3	Kaempferol	0.743
	CASP3	Licochalcone B	0.7
	CASP3	Mairin	0.739
	CASP3	Quercetin	0.953
	CASP3	Saikosaponin D	0.723
95	CASP7	Mairin	0.7
	CASP7	Quercetin	0.7
96	CASP8	Arachidonate	0.722
	CASP8	Baicalin	0.784
	CASP8	Formononetin	0.7
	CASP8	Quercetin	0.736
97	CASP9	Arachidonate	0.726
	CASP9	Formononetin	0.7
	CASP9	Kaempferol	0.722
	CASP9	Quercetin	0.933
	CASP9	Saikosaponin D	0.7
98	CAT	Arachidonate	0.615
	CAT	Hesperetin	0.746
	CAT	Naringenin	0.404
	CAT	Quercetin	0.895
99	CBL	Folic acid	0.506
100	CBR1	Glycyrrhizin	0.427
101	CBR3	Glycyrrhizin	0.427
102	CBR4	Glycyrrhizin	0.427
	CBR4	Quercetin	0.611
103	CBS	Folic acid	0.562
104	CCK	Hesperetin	0.786
	CCK	Naringenin	0.8
105	CCL2	Folic acid	0.844
	CCL2	Kaempferol	0.818
	CCL2	Naringenin	0.816
	CCL2	Quercetin	0.952
106	CCNA1	Naringenin	0.519
107	CCNA2	Naringenin	0.519
108	CCNC	Arachidonate	0.9
109	CCNF	Naringenin	0.519
110	CD36	Arachidonate	0.915
111	CD38	Quercetin	0.8
112	CD69	Naringenin	0.8
113	CD80	Hesperidin	0.8
114	CD86	Naringenin	0.786
115	CD97	Quercetin	0.843
116	CDK1	Kaempferol	0.938
	CDK1	Quercetin	0.491
	CDK1	Troxerutin	0.8
117	CDK19	Arachidonate	0.909
	CDK2	Hesperetin	0.862

118	CDK2	Kaempferol	0.8
	CDK2	Quercetin	0.725
119	CDK4	Hesperetin	0.824
120	CDK8	Arachidonate	0.909
121	CDKN1A	Quercetin	0.733
122	CDKN2A	Quercetin	0.725
123	CDX1	Baicalin	0.8
124	CDX2	Baicalin	0.8
125	CES1	Arachidonate	0.7
126	CFTR	Nobiletin	0.8
	CFTR	Quercetin	0.7
127	CHAT	Arachidonate	0.8
128	CHD9	Arachidonate	0.9
129	CHEK2	Quercetin	0.7
130	CHRFAM7A	Ginsenoside Rg1	0.733
131	CHRNA2	Ginsenoside Rg1	0.456
132	CHRNA3	Ginsenoside Rg1	0.456
133	CHRNA4	Ginsenoside Rg1	0.456
134	CHRNA6	Ginsenoside Rg1	0.456
135	CHRNA7	Ginsenoside Rg1	0.733
136	CHRNB2	Ginsenoside Rg1	0.456
137	CHRNB4	Ginsenoside Rg1	0.456
138	CHUK	Kaempferol	0.7
	CHUK	Quercetin	0.7
139	CKB	Quercetin	0.7
140	CLCN2	Arachidonate	0.8
141	CLOCK	Arachidonate	0.9
142	CNR1	Arachidonate	0.576
143	COQ6	Kaempferol	0.424
144	CPT1A	Arachidonate	0.915
145	CPT2	Arachidonate	0.9
146	CRABP1	Arachidonate	0.57
147	CRABP2	Arachidonate	0.57
148	CREB1	Ginsenoside Rb1	0.786
149	CREBBP	Arachidonate	0.9
150	CREM	Ginsenoside Rb1	0.665
151	CRP	Folic acid	0.549
152	CSF2	Kaempferol	0.7
	CSF2	Quercetin	0.938
153	CSK	Arachidonate	0.7
154	CTGF	Arachidonate	0.908
155	CTNNB1	Quercetin	0.772
	CTNNB1	Decursin	0.8
156	CTRL	Quercetin	0.844
157	CTSE	Stigmasterol	0.436
158	CXCL1	Ferulic acid	0.629
	CXCL1	Isoferulic acid	0.629
159	CXCL10	Quercetin	0.7

160	CXCL2	Ferulic acid	0.657
	CXCL2	Isoferulic acid	0.657
161	CXCL3	Ferulic acid	0.657
	CXCL3	Isoferulic acid	0.657
162	CXCR4	Quercetin	0.8
163	CYBB	Quercetin	0.733
164	CYCS	Ferulic acid	0.819
	CYCS	Mairin	0.824
	CYCS	Quercetin	0.855
165	CYP19A1	Hesperetin	0.7
	CYP19A1	Isolicoflavonol	0.487
	CYP19A1	Naringenin	0.776
	CYP19A1	Quercetin	0.941
166	CYP1A1	Arachidonate	0.914
	CYP1A1	Ferulic acid	0.7
	CYP1A1	Formononetin	0.722
	CYP1A1	Isorhamnetin	0.912
	CYP1A1	Kaempferol	0.847
	CYP1A1	Nobiletin	0.943
	CYP1A1	Quercetin	0.963
167	CYP1A2	Arachidonate	0.911
	CYP1A2	Ferulic acid	0.7
	CYP1A2	Kaempferol	0.84
	CYP1A2	Naringenin	0.822
	CYP1A2	Nobiletin	0.7
	CYP1A2	Quercetin	0.954
168	CYP1B1	Arachidonate	0.919
	CYP1B1	Formononetin	0.7
	CYP1B1	Isorhamnetin	0.933
	CYP1B1	Kaempferol	0.885
	CYP1B1	Naringenin	0.817
	CYP1B1	Quercetin	0.975
169	CYP2A13	Arachidonate	0.8
170	CYP2A7	Arachidonate	0.8
171	CYP2B6	Arachidonate	0.941
	CYP2B6	Kaempferol	0.8
172	CYP2C18	Arachidonate	0.818
173	CYP2C19	Arachidonate	0.912
174	CYP2C8	Arachidonate	0.937
	CYP2C8	Quercetin	0.964
175	CYP2C9	Arachidonate	0.926
	CYP2C9	Astragaloside IV	0.8
	CYP2C9	Quercetin	0.728
176	CYP2D6	Arachidonate	0.815
	CYP2D6	Quercetin	0.722
177	CYP2E1	Arachidonate	0.926
	CYP2E1	Baicalin	0.8
	CYP2E1	Quercetin	0.823

178	CYP2F1	Arachidonate	0.8
179	CYP2J2	Arachidonate	0.975
180	CYP2S1	Arachidonate	0.8
181	CYP2U1	Arachidonate	0.928
182	CYP3A4	Arachidonate	0.731
	CYP3A4	Baicalin	0.8
	CYP3A4	Kaempferol	0.728
	CYP3A4	Quercetin	0.953
	CYP3A4	Vestitol	0.7
183	CYP3A5	Arachidonate	0.815
184	CYP3A7	Arachidonate	0.8
185	CYP4A11	Arachidonate	0.958
	CYP4A11	Quercetin	0.684
186	CYP4A22	Arachidonate	0.822
	CYP4A22	Quercetin	0.684
187	CYP4B1	Arachidonate	0.923
	CYP4B1	Quercetin	0.543
188	CYP4F11	Arachidonate	0.833
	CYP4F11	Quercetin	0.514
189	CYP4F12	Arachidonate	0.931
	CYP4F12	Quercetin	0.445
190	CYP4F2	Arachidonate	0.927
	CYP4F2	Quercetin	0.445
191	CYP4F22	Quercetin	0.445
192	CYP4F3	Arachidonate	0.952
	CYP4F3	Quercetin	0.445
193	CYP4V2	Quercetin	0.433
194	CYP4X1	Arachidonate	0.839
	CYP4X1	Quercetin	0.543
195	CYP4Z1	Arachidonate	0.8
	CYP4Z1	Quercetin	0.543
196	CYP7A1	Arachidonate	0.9
	CYP7A1	beta-Sitosterol	0.771
197	CYSLTR1	Arachidonate	0.529
198	DAGLA	Arachidonate	0.9
199	DAGLB	Arachidonate	0.9
200	DAK	Jaranol	0.414
201	DECR1	Ferulic acid	0.825
	DECR1	Folic acid	0.404
	DECR1	Glycyrrhizin	0.427
202	DECR2	Glycyrrhizin	0.427
203	DEGS1	Folic acid	0.8
204	DGAT1	Hesperetin	0.8
	DGAT1	Nobiletin	0.8
205	DHCR24	beta-Sitosterol	0.841
206	DHFR	Ferulic acid	0.7
	DHFR	Folic acid	0.999
207	DHFRL1	Folic acid	0.842

208	DHRS1	Glycyrrhizin	0.427
209	DHRS12	Glycyrrhizin	0.427
210	DHRS13	Glycyrrhizin	0.427
211	DHRS2	Glycyrrhizin	0.427
212	DHRS3	Glycyrrhizin	0.427
213	DHRS4	Glycyrrhizin	0.427
214	DHRS4L2	Glycyrrhizin	0.427
215	DHRS7B	Glycyrrhizin	0.583
216	DHRS7C	Glycyrrhizin	0.583
217	DHRSX	Glycyrrhizin	0.427
218	DIABLO	Quercetin	0.7
219	DIO2	Kaempferol	0.7
	DIO2	Quercetin	0.7
220	DMGDH	Folic acid	0.471
221	DNMT1	Folic acid	0.875
222	DRD4	Quercetin	0.525
223	DUOX1	Quercetin	0.524
224	DUOX2	Quercetin	0.524
225	EDN1	Arachidonate	0.837
	EDN1	Quercetin	0.7
226	EEF1A1	Quercetin	0.8
227	EGF	Baicalin	0.8
228	EGFR	Mairin	0.7
	EGFR	Quercetin	0.826
229	EIF2A	Quercetin	0.7
230	EIF2AK2	Quercetin	0.7
231	EIF4G3	Quercetin	0.441
232	ELANE	Arachidonate	0.445
233	ENO2	Glycyrrhizin	0.8
234	ENOX1	Arachidonate	0.8
235	ENSG00000168970	Arachidonate	0.917
236	ENSG00000258466	Glycyrrhizin	0.427
237	ENSG00000258643	Quercetin	0.444
238	ENSG00000261740	Quercetin	0.523
239	ENSG00000264813	Astragaloside IV	0.8
240	ENSG00000267149	Glycyrrhizin	0.427
241	ENSG00000268643	Astragaloside IV	0.556
	ENSG00000268643	Glycyrrhizin	0.556
242	EP300	Arachidonate	0.9
243	EPHX2	Arachidonate	0.929
244	EPX	Arachidonate	0.711
245	ERN1	Quercetin	0.734
246	ERN2	Quercetin	0.734
247	ERVFRD-1	Ferulic acid	0.809
248	ERVW-1	Ferulic acid	0.443
249	ESR1	Kaempferol	0.736
	ESR1	Quercetin	0.743
	ESR2	Kaempferol	0.725

250	ESR2	Liquiritigenin	0.727
	ESR2	Quercetin	0.726
251	F2	Fumarine	0.786
252	F3	Quercetin	0.84
253	F8	Bifendate	0.512
254	FAAH	Arachidonate	0.923
255	FAAH2	Arachidonate	0.9
256	FABP1	Arachidonate	0.96
257	FABP12	Arachidonate	0.57
258	FABP2	Arachidonate	0.609
259	FABP3	Arachidonate	0.61
260	FABP4	Arachidonate	0.981
261	FABP5	Arachidonate	0.617
262	FABP6	Arachidonate	0.57
263	FABP7	Arachidonate	0.792
264	FABP9	Arachidonate	0.667
265	FADS1	Arachidonate	0.959
266	FADS2	Arachidonate	0.644
267	FADS3	Arachidonate	0.419
268	FAM108A1	Ferulic acid	0.543
269	FAM108B1	Ferulic acid	0.543
270	FAM108C1	Ferulic acid	0.543
271	FAM91A1	Troxerutin	0.411
272	FAU	Quercetin	0.8
273	FDFT1	Arachidonate	0.9
	FDFT1	Ginsenoside Rg1	0.416
274	FES	Isoferulic acid	0.532
275	FEZF1	Isoferulic acid	0.492
276	FEZF2	Isoferulic acid	0.534
277	FGR	Quercetin	0.505
278	FHL2	Arachidonate	0.9
279	FLNA	Bifendate	0.409
280	FLT3	Quercetin	0.496
281	FNDC5	Quercetin	0.8
282	FOLH1	Folic acid	0.537
283	FOLR1	Folic acid	0.983
284	FOLR2	Folic acid	0.988
285	FOLR3	Folic acid	0.916
286	FOS	Arachidonate	0.952
	FOS	Quercetin	0.845
287	FOXM1	Quercetin	0.824
288	FOXP3	Baicalin	0.8
289	FPGS	Folic acid	0.664
290	FRA10AC1	Folic acid	0.508
291	FRK	Quercetin	0.567
292	FTCD	Folic acid	0.733
293	FXN	Isoferulic acid	0.657
294	FYN	Quercetin	0.505

295	G0S2	Arachidonate	0.9
296	G6PD	Ferulic acid	0.728
	G6PD	Hesperidin	0.7
297	GADD45A	Quercetin	0.824
298	GART	Folic acid	0.886
299	GCLC	Arachidonate	0.7
300	GGH	Folic acid	0.574
301	GHRL	Hesperidin	0.8
302	GIF	Folic acid	0.535
303	GJA1	Glycyrrhizin	0.8
304	GJA9	Glycyrrhizin	0.4
305	GLIPR1	Arachidonate	0.9
306	GLRA1	Quercetin	0.7
307	GNRH1	Arachidonate	0.786
308	GPER	Glycyrrhizin	0.554
309	GPX1	Quercetin	0.428
310	GPX2	Quercetin	0.427
311	GPX4	Arachidonate	0.918
312	GRHL1	Arachidonate	0.9
313	GSK3A	Astragaloside IV	0.683
	GSK3A	Glycyrrhizin	0.683
	GSK3A	Quercetin	0.459
314	GSK3B	Astragaloside IV	0.786
	GSK3B	Glycyrrhizin	0.786
	GSK3B	Quercetin	0.83
315	GSR	Quercetin	0.511
316	GSTP1	Kaempferol	0.7
	GSTP1	Quercetin	0.945
317	H2AFX	Kaempferol	0.7
318	HABP2	Bifendate	0.4
319	HAS1	Quercetin	0.534
320	HAS2	Quercetin	0.534
321	HAS3	Quercetin	0.534
322	HBEGF	Ginsenoside Rb1	0.8
323	HCK	Quercetin	0.969
324	HDAC3	Arachidonate	0.9
325	HELZ2	Arachidonate	0.9
326	HIBCH	Quercetin	0.958
327	HIF1A	Arachidonate	0.9
	HIF1A	Baicalin	0.8
	HIF1A	Quercetin	0.943
328	HIST3H3	Quercetin	0.7
329	HMGA1	Naringenin	0.8
330	HMGB1	Arachidonate	0.804
	HMGB1	Calycosin	0.824
331	HMGB2	Arachidonate	0.628
332	HMGCR	Arachidonate	0.924
	HMGCR	Ginsenoside Rg1	0.4

333	HMGCS1	Arachidonate	0.9
	HMGCS1	Naringenin	0.636
334	HMGCS2	Arachidonate	0.909
	HMGCS2	Naringenin	0.784
335	HMOX1	Hesperidin	0.8
	HMOX1	Isorhamnetin	0.8
	HMOX1	Kaempferol	0.818
	HMOX1	Naringenin	0.816
	HMOX1	Quercetin	0.879
336	HMOX2	Arachidonate	0.401
337	HOXD13	Bifendate	0.683
338	HPGD	Glycyrrhizin	0.427
339	HPGDS	Arachidonate	0.418
	HPGDS	Quercetin	0.871
340	HRH1	Fumarine	0.8
341	HSD11B1	Glycyrrhizin	0.926
342	HSD11B1L	Glycyrrhizin	0.681
343	HSD11B2	Glycyrrhizin	0.883
344	HSD17B1	Glycyrrhizin	0.618
345	HSD17B11	Glycyrrhizin	0.427
346	HSD17B13	Glycyrrhizin	0.427
347	HSD17B14	Glycyrrhizin	0.427
348	HSD17B8	Glycyrrhizin	0.427
349	HSD3B1	Quercetin	0.517
350	HSD3B2	Quercetin	0.517
351	HSD3B7	Quercetin	0.517
352	HSDL2	Glycyrrhizin	0.495
353	HSPA1A	Quercetin	0.7
354	HSPA4	Quercetin	0.895
355	HSPA8	Quercetin	0.573
356	HSPB1	Quercetin	0.824
357	HSPB2	Quercetin	0.529
358	HSPB3	Quercetin	0.416
359	HTR2A	Arachidonate	0.421
	HTR2A	Glabrene	0.8
360	ICAM1	Arachidonate	0.419
	ICAM1	beta-Sitosterol	0.8
	ICAM1	Glabridin	0.8
	ICAM1	Quercetin	0.847
361	IFNG	Arachidonate	0.833
362	IGF1R	Kaempferol	0.815
	IGF1R	Quercetin	0.593
363	IGF2	Kaempferol	0.8
364	IGFBP3	Folic acid	0.835
	IGFBP3	Quercetin	0.824
365	IL10	Stigmasterol	0.7
366	IL15	Arachidonate	0.551
	IL15	Quercetin	0.83

367	IL17A	Baicalin	0.8
	IL17A	Quercetin	0.816
368	IL1B	Arachidonate	0.511
	IL1B	Quercetin	0.738
369	IL2	Kaempferol	0.8
370	IL6	Arachidonate	0.955
	IL6	Quercetin	0.951
371	IL8	Arachidonate	0.807
	IL8	Licochalcone a	0.8
	IL8	Quercetin	0.778
	IL8	Stigmasterol	0.7
372	INS	Arachidonate	0.724
373	IREB2	Isoferulic acid	0.549
374	IRF7	Hesperidin	0.8
375	IRS1	Quercetin	0.462
376	ISCU	Isoferulic acid	0.634
377	ITGA2	Kaempferol	0.8
378	ITGA2B	Arachidonate	0.423
379	JAK2	Quercetin	0.498
380	JUN	Arachidonate	0.869
	JUN	Kaempferol	0.822
	JUN	Quercetin	0.948
381	KCNH2	Naringenin	0.8
382	KCNJ1	Arachidonate	0.816
383	KCNJ4	Arachidonate	0.8
384	KCNK18	Arachidonate	0.856
385	KCNMA1	Arachidonate	0.816
	KCNMA1	Naringenin	0.786
	KCNMA1	Quercetin	0.786
386	KCNU1	Naringenin	0.628
	KCNU1	Quercetin	0.628
387	KDM1A	Quercetin	0.5
388	KDM1B	Quercetin	0.562
389	KDR	Arachidonate	0.815
	KDR	Quercetin	0.557
390	KEAP1	Quercetin	0.402
391	KIAA0101	Arachidonate	0.621
	KIAA0101	Fumarine	0.8
392	KLHL2	Arachidonate	0.576
393	KLK3	Decursin	0.7
	KLK3	Decursinol angelate	0.8
394	KLK4	Arachidonate	0.462
395	KMO	Kaempferol	0.424
396	KNG1	Arachidonate	0.657
397	KRAS	Quercetin	0.853
398	LBH	Isolicoflavonol	0.482
	LBH	Licoricone	0.482
399	LCAT	Arachidonate	0.416

400	LDHA	Glabridin	0.498
401	LDHAL6A	Glabridin	0.498
402	LDHAL6B	Glabridin	0.498
403	LDHB	Glabridin	0.498
404	LDHC	Glabridin	0.498
405	LDLR	Naringenin	0.8
406	LEPR	Quercetin	0.786
407	LHCGR	Medicarpin	0.8
408	LMNB1	Mairin	0.8
409	LOX	Arachidonate	0.556
410	LPL	Arachidonate	0.494
411	LPO	Arachidonate	0.711
412	LTA4H	Arachidonate	0.452
413	LTB4R	Arachidonate	0.428
414	LTB4R2	Arachidonate	0.537
415	LTC4S	Arachidonate	0.937
416	MAOA	Quercetin	0.759
417	MAOB	Quercetin	0.616
418	MAP2K4	Isoflavanone	0.402
419	MAPK1	Calycosin	0.7
	MAPK1	Ferulic acid	0.7
	MAPK1	Ginsenoside Rb1	0.504
	MAPK1	Kaempferol	0.7
	MAPK1	Mairin	0.7
	MAPK1	Quercetin	0.87
420	MAPK11	Ginsenoside Rb1	0.684
	MAPK11	Quercetin	0.684
421	MAPK12	Ginsenoside Rb1	0.579
	MAPK12	Quercetin	0.579
422	MAPK13	Ginsenoside Rb1	0.579
	MAPK13	Quercetin	0.631
423	MAPK14	Ginsenoside Rb1	0.684
	MAPK14	Naringenin	0.8
	MAPK14	Quercetin	0.723
424	MAPK15	Ginsenoside Rb1	0.412
	MAPK15	Quercetin	0.412
425	MAPK3	Calycosin	0.7
	MAPK3	Ferulic acid	0.7
	MAPK3	Ginsenoside Rb1	0.504
	MAPK3	Kaempferol	0.7
	MAPK3	Mairin	0.7
	MAPK3	Quercetin	0.863
426	MAPK4	Ginsenoside Rb1	0.412
	MAPK4	Quercetin	0.412
427	MAPK6	Ginsenoside Rb1	0.412
	MAPK6	Quercetin	0.412
428	MAPK7	Ginsenoside Rb1	0.455
	MAPK7	Quercetin	0.455

429	MAPK8	Isorhamnetin	0.8
	MAPK8	Quercetin	0.951
430	MAPK9	Isorhamnetin	0.733
431	MAPT	Arachidonate	0.724
432	MCL1	Quercetin	0.987
433	ME1	Arachidonate	0.9
434	MED1	Arachidonate	0.9
435	MED10	Arachidonate	0.9
436	MED11	Arachidonate	0.9
437	MED12	Arachidonate	0.9
438	MED13	Arachidonate	0.9
439	MED13L	Arachidonate	0.9
440	MED14	Arachidonate	0.9
441	MED15	Arachidonate	0.9
442	MED16	Arachidonate	0.9
443	MED17	Arachidonate	0.9
444	MED18	Arachidonate	0.9
445	MED19	Arachidonate	0.9
446	MED20	Arachidonate	0.9
447	MED21	Arachidonate	0.9
448	MED22	Arachidonate	0.9
449	MED23	Arachidonate	0.9
450	MED24	Arachidonate	0.9
451	MED25	Arachidonate	0.9
452	MED26	Arachidonate	0.9
453	MED27	Arachidonate	0.9
454	MED29	Arachidonate	0.9
455	MED30	Arachidonate	0.9
456	MED31	Arachidonate	0.9
457	MED4	Arachidonate	0.9
458	MED6	Arachidonate	0.9
459	MED7	Arachidonate	0.9
460	MED8	Arachidonate	0.9
461	MED9	Arachidonate	0.9
462	MET	Quercetin	0.56
463	MGLL	Arachidonate	0.919
464	MMD	Folic acid	0.401
465	MME	Nobiletin	0.8
466	MMP1	Kaempferol	0.815
	MMP1	Quercetin	0.84
467	MMP13	Arachidonate	0.729
468	MMP2	Arachidonate	0.768
	MMP2	Kaempferol	0.815
	MMP2	Nobiletin	0.8
	MMP2	Quercetin	0.842
469	MMP3	Quercetin	0.839
	MMP9	Arachidonate	0.757
	MMP9	Ginsenoside Rg1	0.817

470	MMP9	Hesperidin	0.938
	MMP9	Nobiletin	0.818
	MMP9	Quercetin	0.956
	MMP9	Decursin	0.8
471	MPO	Arachidonate	0.975
	MPO	Quercetin	0.439
472	MT-CO2	Arachidonate	0.908
473	MTHFD1	Folic acid	0.894
474	MTHFD1L	Folic acid	0.811
475	MTHFR	Folic acid	0.997
476	MTHFS	Folic acid	0.506
477	MT-ND4	Isoferulic acid	0.425
478	MTOR	Quercetin	0.614
479	MTR	Folic acid	0.846
480	MTRR	Baicalin	0.457
	MTRR	Folic acid	0.826
	MTRR	Glabridin	0.473
	MTRR	Glycyrrhizin	0.473
	MTRR	Isorhamnetin	0.457
	MTRR	Kaempferol	0.473
	MTRR	Nodakenin	0.473
481	MTTP	Hesperetin	0.826
482	MVD	Ginsenoside Rg1	0.436
483	MYC	Baicalin	0.8
	MYC	Nobiletin	0.8
484	NAMPT	Arachidonate	0.8
	NAMPT	Quercetin	0.8
485	NAT1	Folic acid	0.809
486	NCF1	Arachidonate	0.75
487	NCOA1	Arachidonate	0.9
	NCOA1	Quercetin	0.422
488	NCOA2	Arachidonate	0.9
489	NCOA3	Arachidonate	0.9
490	NCOA6	Arachidonate	0.9
491	NCOR1	Arachidonate	0.9
492	NCOR2	Arachidonate	0.9
493	NF1	Arachidonate	0.673
494	NFE2L2	Ginsenoside Rb1	0.7
	NFE2L2	Quercetin	0.794
495	NFKBIA	Kaempferol	0.7
	NFKBIA	Quercetin	0.725
496	NFU1	Isoferulic acid	0.519
497	NFYA	Arachidonate	0.9
498	NFYB	Arachidonate	0.9
499	NFYC	Arachidonate	0.9
500	NKX3-1	Quercetin	0.824
	NOS1	Baicalin	0.596
	NOS1	Glabridin	0.786

	NOS1	Glycyrrhizin	0.786
501	NOS1	Isorhamnetin	0.596
	NOS1	Kaempferol	0.786
	NOS1	Quercetin	0.786
	NOS1	Nodakenin	0.786
	NOS2	Baicalin	0.733
	NOS2	Glabridin	0.613
	NOS2	Glycyrrhizin	0.613
502	NOS2	Isorhamnetin	0.733
	NOS2	Kaempferol	0.766
	NOS2	Quercetin	0.766
	NOS2	Nodakenin	0.613
	NOS3	Arachidonate	0.41
	NOS3	Baicalin	0.596
	NOS3	Folic acid	0.853
	NOS3	Ginsenoside Rb1	0.834
	NOS3	Ginsenoside Re	0.722
503	NOS3	Glabridin	0.613
	NOS3	Glycyrrhizin	0.613
	NOS3	Isorhamnetin	0.596
	NOS3	Kaempferol	0.596
	NOS3	Mairin	0.8
	NOS3	Quercetin	0.85
	NOS3	Nodakenin	0.613
504	NOTCH1	Baicalin	0.7
	NOTCH1	Hesperetin	0.8
505	NOTCH2	Liquiritigenin	0.821
506	NOX1	Arachidonate	0.735
	NOX1	Quercetin	0.62
507	NOX3	Quercetin	0.703
508	NOX4	Quercetin	0.663
509	NOX5	Quercetin	0.47
510	NPAS2	Arachidonate	0.9
511	NPC1L1	beta-Sitosterol	0.467
	NPC1L1	Quercetin	0.8
512	NPY1R	Quercetin	0.8
513	NQO1	Quercetin	0.654
514	NR1H2	beta-Sitosterol	0.58
	NR1H2	Stigmasterol	0.437
515	NR1H3	beta-Sitosterol	0.576
	NR1H3	Stigmasterol	0.476
	NR1I2	Hesperetin	0.7
516	NR1I2	Kaempferol	0.938
	NR1I2	Quercetin	0.7
517	NR1I3	Kaempferol	0.7
	NR1I3	Quercetin	0.7
518	NR3C1	Arachidonate	0.434
	NR3C1	Ginsenoside Re	0.7

519	NRF1	Arachidonate	0.9
520	NSDHL	Quercetin	0.517
521	NT5E	Baicalin	0.8
	NT5E	Quercetin	0.84
522	NUBPL	Isoferulic acid	0.481
523	ODC1	Quercetin	0.824
524	OXT	Arachidonate	0.844
525	OXTR	Arachidonate	0.47
526	P2RY1	Arachidonate	0.548
527	PARP1	Arachidonate	0.728
	PARP1	Folic acid	0.722
	PARP1	Quercetin	0.742
	PARP1	Decursin	0.7
528	PDE4A	Formononetin	0.8
529	PDGFB	Baicalin	0.8
530	PDX1	Quercetin	0.8
531	PECR	Glycyrrhizin	0.427
532	PEMT	Folic acid	0.402
533	PEX11A	Arachidonate	0.9
534	PFAS	Folic acid	0.532
535	PGD	Ferulic acid	0.7
536	PGF	Arachidonate	0.891
537	PI4KA	Quercetin	0.582
538	PIK3C2A	Quercetin	0.523
539	PIK3C2B	Quercetin	0.523
540	PIK3C2G	Quercetin	0.523
541	PIK3C3	Quercetin	0.523
542	PIK3CA	Quercetin	0.723
543	PIK3CB	Quercetin	0.654
544	PIK3CD	Quercetin	0.654
545	PIK3CG	Quercetin	0.934
546	PIM1	Quercetin	0.969
547	PKM	Baicalin	0.7
548	PLA2G10	Arachidonate	0.964
549	PLA2G12A	Arachidonate	0.916
550	PLA2G12B	Arachidonate	0.9
551	PLA2G16	Arachidonate	0.9
552	PLA2G1B	Arachidonate	0.997
553	PLA2G2A	Arachidonate	0.976
554	PLA2G2C	Arachidonate	0.9
555	PLA2G2D	Arachidonate	0.583
556	PLA2G2E	Arachidonate	0.911
557	PLA2G2F	Arachidonate	0.9
558	PLA2G3	Arachidonate	0.941
559	PLA2G4A	Arachidonate	0.985
560	PLA2G4B	Arachidonate	0.929
561	PLA2G4C	Arachidonate	0.956
562	PLA2G4D	Arachidonate	0.9

563	PLA2G4E	Arachidonate	0.9
564	PLA2G4F	Arachidonate	0.9
565	PLA2G5	Arachidonate	0.927
566	PLA2G6	Arachidonate	0.963
567	PLAA	Arachidonate	0.489
568	PLAT	Quercetin	0.727
569	PLAU	Quercetin	0.837
570	PLB1	Arachidonate	0.9
571	PLCB1	Arachidonate	0.9
572	PLD2	Arachidonate	0.462
573	PLIN2	Arachidonate	0.917
574	PMP2	Arachidonate	0.57
575	PNLIP	Mairin	0.8
576	PNPLA8	Arachidonate	0.441
577	PON1	Arachidonate	0.788
	PON1	Quercetin	0.944
578	PON2	Isorhamnetin	0.8
	PON2	Quercetin	0.8
579	POR	Baicalin	0.457
	POR	Glabridin	0.473
	POR	Glycyrrhizin	0.473
	POR	Isorhamnetin	0.457
	POR	Kaempferol	0.473
	POR	Quercetin	0.7
	POR	Nodakenin	0.473
580	PPARA	Arachidonate	0.951
	PPARA	Folic acid	0.84
	PPARA	Hesperetin	0.843
	PPARA	Naringenin	0.86
	PPARA	Quercetin	0.865
581	PPARG	Arachidonate	0.951
	PPARG	Hesperetin	0.824
582	PPARGC1A	Arachidonate	0.9
	PPARGC1A	Glycyrrhizin	0.8
583	PPARGC1B	Arachidonate	0.9
584	PPAT	Isoferulic acid	0.464
585	PPIG	Arachidonate	0.91
586	PRDX5	Quercetin	0.8
587	PRKAA1	Ginsenoside Re	0.7
588	PRKCA	Arachidonate	0.991
589	PRKCB	Arachidonate	0.916
590	PRKCD	Folic acid	0.407
	PRKCD	Isorhamnetin	0.7
591	PRKCG	Arachidonate	0.991
592	PRKDC	Quercetin	0.523
593	PRL	Arachidonate	0.816
594	PTGER1	Arachidonate	0.542
595	PTGER3	Arachidonate	0.536

596	PTGER4	Arachidonate	0.563
597	PTGES	Arachidonate	0.613
	PTGES	Kaempferol	0.8
598	PTGES2	Arachidonate	0.426
599	PTGES3	Arachidonate	0.55
600	PTGIR	Arachidonate	0.596
601	PTGS1	Arachidonate	0.999
	PTGS1	Quercetin	0.755
602	PTGS2	Arachidonate	0.999
	PTGS2	Quercetin	0.955
603	PTHLH	Bifendate	0.697
	PTHLH	Mairin	0.7
604	PTK2	Quercetin	0.785
605	PXDN	Arachidonate	0.711
606	PXDNL	Arachidonate	0.711
607	PYGB	Quercetin	0.748
608	PYGL	Quercetin	0.712
609	PYGM	Quercetin	0.795
610	QDPR	Folic acid	0.404
611	RAF1	Quercetin	0.836
612	RAPGEF1	Naringenin	0.8
613	RB1	Ferulic acid	0.7
	RB1	Ginsenoside Rb1	0.643
	RB1	Ginsenoside Rg1	0.643
	RB1	Kaempferol	0.8
	RB1	Quercetin	0.7
614	RBL2	Ginsenoside Rb1	0.403
	RBL2	Ginsenoside Rg1	0.49
615	RBP1	Arachidonate	0.57
616	RBP2	Arachidonate	0.57
617	RBP5	Arachidonate	0.57
618	RBP7	Arachidonate	0.57
619	RDH10	Glycyrrhizin	0.427
620	RDH11	Glycyrrhizin	0.427
621	RDH12	Glycyrrhizin	0.427
622	RDH13	Glycyrrhizin	0.427
623	RDH14	Glycyrrhizin	0.427
624	RDH8	Glycyrrhizin	0.583
625	REN	Arachidonate	0.873
	REN	Baicalin	0.8
626	RGL1	Arachidonate	0.9
627	RHAG	Ginsenoside Rg1	0.474
	RHAG	Ginsenoside Rh2	0.494
628	RHOA	Arachidonate	0.844
629	RNASE3	Hederagenin	0.512
630	RNASEL	Quercetin	0.7
631	RORA	Arachidonate	0.9
632	RPS6KA3	Kaempferol	0.88

633	RPS6KA5	Quercetin	0.7
634	RXRA	Arachidonate	0.907
635	SARDH	Folic acid	0.418
636	SCD	Arachidonate	0.415
	SCD	Naringenin	0.8
637	SCD5	Naringenin	0.733
638	SDR16C5	Glycyrrhizin	0.427
639	SDR42E1	Quercetin	0.517
640	SDR42E2	Quercetin	0.517
641	SELP	Arachidonate	0.412
642	SERPIND1	Quercetin	0.7
643	SERPINE1	Astragaloside IV	0.8
	SERPINE1	Folic acid	0.826
644	SESN2	Quercetin	0.824
645	SHMT1	Folic acid	0.772
646	SHMT2	Folic acid	0.674
647	SI	Isoferulic acid	0.8
648	SIN3A	Arachidonate	0.9
649	SIRT1	Quercetin	0.953
650	SIRT2	Quercetin	0.427
651	SIRT3	Quercetin	0.441
652	SIRT4	Quercetin	0.427
653	SIRT5	Quercetin	0.63
654	SIRT6	Quercetin	0.434
655	SIRT7	Quercetin	0.498
656	SLC12A2	Quercetin	0.824
657	SLC19A1	Folic acid	0.921
658	SLC19A3	Folic acid	0.542
659	SLC25A26	Isoflavanone	0.402
	SLC25A26	Jaranol	0.412
660	SLC25A32	Folic acid	0.459
661	SLC27A1	Arachidonate	0.907
662	SLC2A1	Kaempferol	0.7
	SLC2A1	Quercetin	0.813
663	SLC2A2	Quercetin	0.965
664	SLC2A3	Quercetin	0.422
665	SLC2A4	Quercetin	0.877
666	SLC2A9	Quercetin	0.422
667	SLC30A1	Quercetin	0.637
668	SLC30A10	Quercetin	0.637
669	SLC46A1	Folic acid	0.973
670	SLC6A3	Arachidonate	0.543
	SLC6A3	Nobiletin	0.471
671	SLCO1B1	Stigmasterol	0.7
672	SMARCD3	Arachidonate	0.9
673	SMG1	Quercetin	0.582
674	SNCA	Arachidonate	0.543
	SNCA	Nobiletin	0.471

675	SOAT1	Hesperetin	0.8
676	SOAT2	Hesperetin	0.8
677	SP1	Arachidonate	0.907
	SP1	Hesperidin	0.8
	SP1	Mairin	0.8
	SP1	Quercetin	0.7
678	SQLE	Kaempferol	0.424
	SQLE	Quercetin	0.42
679	SRC	Glycyrrhizin	0.8
	SRC	Kaempferol	0.8
	SRC	Quercetin	0.951
680	SREBF1	beta-Sitosterol	0.816
681	SREBF2	Arachidonate	0.9
	SREBF2	beta-Sitosterol	0.926
682	STAT1	Kaempferol	0.8
	STAT1	Quercetin	0.727
683	STAT3	Kaempferol	0.814
684	STK11	Ginsenoside Re	0.7
685	STK17B	Quercetin	0.958
686	STMN4	Ginsenoside Rg1	0.403
687	SULT1A1	Pinocembrin	0.7
	SULT1A1	Quercetin	0.734
688	SULT1A3	Pinocembrin	0.7
689	SULT1E1	Quercetin	0.723
690	SULT2A1	Arachidonate	0.9
691	TAC1	Arachidonate	0.866
692	TBK1	Quercetin	0.7
693	TBL1X	Arachidonate	0.9
694	TBL1XR1	Arachidonate	0.9
695	TBXA2R	Arachidonate	0.972
696	TBXAS1	Arachidonate	0.596
697	TCN2	Folic acid	0.689
698	TEAD1	Arachidonate	0.9
699	TEAD2	Arachidonate	0.9
700	TEAD3	Arachidonate	0.9
701	TEAD4	Arachidonate	0.9
702	TF	Folic acid	0.474
703	TFAM	Quercetin	0.8
704	TFRC	Folic acid	0.435
	TFRC	Isoferulic acid	0.462
705	TGDS	Naringenin	0.515
706	TGFA	Quercetin	0.8
707	TGM2	Quercetin	0.8
708	TGS1	Arachidonate	0.9
709	TIAM2	Arachidonate	0.9
710	TLR1	Quercetin	0.814
711	TLR10	Quercetin	0.816
	TLR2	Baicalin	0.816

712	TLR2	Glycyrrhizin	0.818
	TLR2	Naringenin	0.8
	TLR2	Quercetin	0.8
	TLR4	Astragaloside IV	0.8
713	TLR4	Baicalin	0.816
	TLR4	Quercetin	0.845
714	TLR6	Quercetin	0.814
715	TMEM30A	Ferulic acid	0.449
716	TMEM30B	Ferulic acid	0.449
717	TMEM30C	Ferulic acid	0.427
	TMPRSS11D	Arachidonate	0.786
718	TMPRSS11D	Hesperetin	0.786
	TMPRSS11D	Nobiletin	0.786
	TMPRSS11D	Quercetin	0.786
	TNF	Arachidonate	0.945
	TNF	Baicalin	0.697
719	TNF	Naringenin	0.679
	TNF	Quercetin	0.763
	TNF	Stigmasterol	0.7
720	TNFRSF10B	Quercetin	0.84
721	TNFRSF11B	Kaempferol	0.8
	TNFRSF11B	Medicarpin	0.8
722	TNFRSF21	Arachidonate	0.9
723	TNFSF10	Quercetin	0.742
724	TOP1	Mairin	0.721
725	TOP2A	Mairin	0.7
	TOP2A	Quercetin	0.674
726	TOP2B	Quercetin	0.674
	TP53	Astragaloside IV	0.8
	TP53	Folic acid	0.873
727	TP53	Hesperidin	0.8
	TP53	Kaempferol	0.738
	TP53	Quercetin	0.952
728	TPO	Arachidonate	0.711
729	TRIB3	Arachidonate	0.9
730	TRPM5	Arachidonate	0.8
731	TRPM7	Quercetin	0.8
732	TRPV1	Arachidonate	0.413
733	TRPV4	Arachidonate	0.969
734	TRRAP	Quercetin	0.582
735	TSTA3	Quercetin	0.517
736	TTR	Glabridin	0.8
737	TXNRD1	Arachidonate	0.911
738	TYMS	Folic acid	0.989
	TYR	Ferulic acid	0.784
739	TYR	Glabridin	0.859
	TYR	Quercetin	0.81
740	UEVLD	Glabridin	0.498

741	UGCG	Quercetin	0.476
742	UGT1A1	Arachidonate	0.7
	UGT1A1	Calycosin	0.7
	UGT1A1	Formononetin	0.731
	UGT1A1	Glabridin	0.755
	UGT1A1	Kaempferol	0.86
	UGT1A1	Quercetin	0.908
743	UGT1A10	Arachidonate	0.7
	UGT1A10	Calycosin	0.7
	UGT1A10	Ferulic acid	0.7
	UGT1A10	Formononetin	0.731
	UGT1A10	Glabridin	0.755
	UGT1A10	Kaempferol	0.86
	UGT1A10	Quercetin	0.898
744	UGT1A3	Arachidonate	0.7
	UGT1A3	Calycosin	0.7
	UGT1A3	Ferulic acid	0.7
	UGT1A3	Glabridin	0.784
	UGT1A3	Kaempferol	0.86
	UGT1A3	Quercetin	0.908
745	UGT1A4	Arachidonate	0.7
	UGT1A4	Kaempferol	0.547
	UGT1A4	Quercetin	0.696
746	UGT1A5	Kaempferol	0.547
	UGT1A5	Quercetin	0.703
747	UGT1A6	Kaempferol	0.547
	UGT1A6	Quercetin	0.678
748	UGT1A7	Calycosin	0.7
	UGT1A7	Ferulic acid	0.7
	UGT1A7	Formononetin	0.731
	UGT1A7	Kaempferol	0.86
	UGT1A7	Quercetin	0.906
749	UGT1A8	Calycosin	0.7
	UGT1A8	Ferulic acid	0.7
	UGT1A8	Formononetin	0.731
	UGT1A8	Glabridin	0.742
	UGT1A8	Kaempferol	0.86
	UGT1A8	Quercetin	0.886
750	UGT1A9	Arachidonate	0.9
	UGT1A9	Calycosin	0.7
	UGT1A9	Formononetin	0.731
	UGT1A9	Glabridin	0.742
	UGT1A9	Kaempferol	0.86
	UGT1A9	Quercetin	0.897
751	UGT2A1	Kaempferol	0.547
	UGT2A1	Quercetin	0.672
752	UGT2A3	Kaempferol	0.547
	UGT2A3	Quercetin	0.713

753	UGT2B10	Kaempferol	0.547
	UGT2B10	Quercetin	0.672
754	UGT2B11	Kaempferol	0.547
	UGT2B11	Quercetin	0.672
755	UGT2B15	Glabridin	0.755
	UGT2B15	Kaempferol	0.547
	UGT2B15	Quercetin	0.898
756	UGT2B17	Kaempferol	0.547
	UGT2B17	Quercetin	0.672
757	UGT2B28	Kaempferol	0.547
	UGT2B28	Quercetin	0.672
758	UGT2B4	Kaempferol	0.547
	UGT2B4	Quercetin	0.713
759	UGT2B7	Arachidonate	0.7
	UGT2B7	Calycosin	0.7
	UGT2B7	Glabridin	0.755
	UGT2B7	Kaempferol	0.547
	UGT2B7	Quercetin	0.672
760	UGT3A1	Kaempferol	0.906
	UGT3A1	Quercetin	0.924
761	UGT3A2	Kaempferol	0.547
	UGT3A2	Quercetin	0.632
762	UGT8	Kaempferol	0.547
	UGT8	Quercetin	0.632
763	UXS1	Quercetin	0.517
764	VEGFA	Arachidonate	0.424
	VEGFA	Baicalin	0.815
	VEGFA	Ginsenoside Rg1	0.817
	VEGFA	Nobiletin	0.8
	VEGFA	Quercetin	0.947
765	VR1	Arachidonate	0.405
766	VTN	Isoferulic acid	0.512
767	WDTC1	Arachidonate	0.582
768	WWTR1	Arachidonate	0.9
769	XBP1	Quercetin	0.732
770	XDH	Quercetin	0.907
771	YAP1	Arachidonate	0.9
772	YES1	Quercetin	0.505
