# Supplementary material for: Protective Effect of Bojungikki-Tang against Radiation-Induced Intestinal Injury in Mice: Experimental Verification and Compound-Target Prediction
Source: Evid Based Complement Alternat Med. 2023 Jan 4;2023:5417813. doi: 10.1155/2023/5417813 (PMC9833920; doi:10.1155/2023/5417813)
Supplement: Supplementary Materials — See the Supplementary Tables (Tables 1–4). [file 5417813.f1.zip › Supple_Tables_4.pdf]

Supplementary Table 4. Intestinal injury-related genes in GeneCards DB

	Symbol	Description	Category	GIFtS	GC id	Score ▼
1	IL6	Interleukin	Protein Co	50	GC07P022	82.33
2	TNF	Tumor Ne	Protein Co	51	GC06P047	73.14
3	TP53	Tumor Pro	Protein Co	54	GC17M007	68.86
4	IL10	Interleukin	Protein Co	47	GC01M206	68.14
5	IL1B	Interleukin	Protein Co	48	GC02M112	60.95
6	TLR4	Toll Like R	Protein Co	51	GC09P117	58.95
7	CTNNB1	Catenin Be	Protein Co	53	GC03P041	57.7
8	IFNG	Interferon	Protein Co	48	GC12M068	50.66
9	KRAS	KRAS Prot	Protein Co	51	GC12M025	49.17
10	APC	APC Regul	Protein Co	48	GC05P112	48.88
11	TGFB1	Transformi	Protein Co	52	GC19M041	48.26
12	PTEN	Phosphata	Protein Co	52	GC10P087	45.44
13	SMAD4	SMAD Fan	Protein Co	50	GC18P051	45.33
14	GDNF	Glial Cell D	Protein Co	47	GC05M037	44.9
15	FLNA	Filamin A	Protein Co	49	GC0XM154	44.6
16	NOD2	Nucleotide	Protein Co	48	GC16P050	43.98
17	RET	Ret Proto-	Protein Co	53	GC10P043	43.79
18	AKT1	AKT Serine	Protein Co	54	GC14M104	43.31
19	ABCB1	ATP Bindir	Protein Co	51	GC07M087	43.05
20	TLR2	Toll Like R	Protein Co	51	GC04P153	42.73
21	MPO	Myelopero	Protein Co	50	GC17M058	41.07
22	CXCL8	C-X-C Mot	Protein Co	41	GC04P073	40.58
23	PIK3CA	Phosphatic	Protein Co	52	GC03P179	40.29
24	HLA-DRB1	Major Hist	Protein Co	46	GC06M032	40.07
25	STAT3	Signal Trar	Protein Co	52	GC17M042	39.57
26	ICAM1	Intercellula	Protein Co	50	GC19P010	39.39
27	BDNF	Brain Deriv	Protein Co	47	GC11M027	39.07
28	INS	Insulin	Protein Co	48	GC11M002	38.94
29	KIT	KIT Proto-	Protein Co	53	GC04P054	38.37
30	CTLA4	Cytotoxic T	Protein Co	45	GC02P203	38.17
31	EGFR	Epidermal	Protein Co	54	GC07P055	37.9
32	CFTR	CF Transm	Protein Co	51	GC07P117	37.23
33	CLMP	CXADR Lik	Protein Co	40	GC11M123	37.22
34	VIP	Vasoactive	Protein Co	44	GC06P152	36.67
35	NOS2	Nitric Oxid	Protein Co	49	GC17M027	36.59
36	CDH1	Cadherin 1	Protein Co	50	GC16P068	36.27
37	SLC9A3	Solute Car	Protein Co	47	GC05M000	35.84
38	GFAP	Glial Fibrill	Protein Co	47	GC17M044	35.7
39	ERBB2	Erb-B2 Rec	Protein Co	54	GC17P039	35.53
40	FOXP3	Forkhead B	Protein Co	46	GC0XM049	35.09
41	MYLK	Myosin Lig	Protein Co	52	GC03M123	34.98

42	EDNRB	Endothelin	Protein Co	49	GC13M077	34.55
43	COMT	Catechol-C	Protein Co	51	GC22P019	34.48
44	NTRK1	Neurotrop	Protein Co	48	GC01P156	34.47
45	IL1RN	Interleukin	Protein Co	48	GC02P115	33.86
46	ALB	Albumin	Protein Co	50	GC04P073	33.8
47	MSH2	MutS Hom	Protein Co	48	GC02P047	33.05
48	TNFRSF1A	TNF Recep	Protein Co	49	GC12M006	32.79
49	FAS	Fas Cell Su	Protein Co	50	GC10P088	32.74
50	HRAS	HRas Proto	Protein Co	52	GC11M000	32.44
51	CDKN2A	Cyclin Dep	Protein Co	51	GC09M021	32.21
52	HLA-B	Major Hist	Protein Co	45	GC06M031	32.07
53	TGFBR2	Transformi	Protein Co	51	GC03P030	32.06
54	SI	Sucrase-Iso	Protein Co	43	GC03M164	32.04
55	PMS2	PMS1 Hon	Protein Co	48	GC07M005	31.94
56	CRP	C-Reactive	Protein Co	46	GC01M159	31.9
57	MYC	MYC Proto	Protein Co	51	GC08P127	31.78
58	STAT1	Signal Trar	Protein Co	53	GC02M190	31.74
59	SERPINE1	Serpin Fan	Protein Co	50	GC07P101	31.68
60	SOX10	SRY-Box Ti	Protein Co	45	GC22M046	31.58
61	IL10RA	Interleukin	Protein Co	45	GC11P117	31.56
62	CCND1	Cyclin D1	Protein Co	52	GC11P069	31.18
63	TSC2	TSC Comp	Protein Co	50	GC16P002	30.93
64	VWF	Von Willeb	Protein Co	48	GC12M005	30.89
65	TTC7A	Tetratricop	Protein Co	38	GC02P046	30.82
66	EDN3	Endothelin	Protein Co	47	GC20P059	30.73
67	TTR	Transthyre	Protein Co	49	GC18P031	30.57
68	CREBBP	CREB Bind	Protein Co	52	GC16M003	30.48
69	MTOR	Mechanisti	Protein Co	54	GC01M011	30.39
70	BRAF	B-Raf Proto	Protein Co	54	GC07M140	30.38
71	F5	Coagulatio	Protein Co	45	GC01M169	30.12
72	ZEB2	Zinc Finge	Protein Co	48	GC02M144	29.73
73	MIR21	MicroRNA	RNA Gene	24	GC17P059	29.7
74	HNF4A	Hepatocyte	Protein Co	50	GC20P044	29.53
75	EPCAM	Epithelial C	Protein Co	47	GC02P047	29.37
76	SERPINA1	Serpin Fan	Protein Co	49	GC14M094	29.32
77	PRTN3	Proteinase	Protein Co	44	GC19P000	29.32
78	TYMP	Thymidine	Protein Co	46	GC22M050	29.05
79	TGFB2	Transformi	Protein Co	50	GC01P218	28.92
80	CASP3	Caspase 3	Protein Co	50	GC04M184	28.77
81	GNAS	GNAS Con	Protein Co	50	GC20P058	28.6
82	EP300	E1A Bindin	Protein Co	50	GC22P041	28.32
83	MMP9	Matrix Met	Protein Co	52	GC20P046	28.28
84	IL10RB	Interleukin	Protein Co	44	GC21P033	28

85	PLG	Plasminog	Protein Co	48	GC06P160	27.87
86	POLG	DNA Polyr	Protein Co	45	GC15M089	27.85
87	PTPN22	Protein Ty	Protein Co	46	GC01M113	27.85
88	MYH11	Myosin He	Protein Co	45	GC16M015	27.48
89	IL4	Interleukin	Protein Co	46	GC05P132	27.45
90	PTCH1	Patched 1	Protein Co	50	GC09M095	27.43
91	STK11	Serine/Thr	Protein Co	49	GC19P001	27.42
92	THBD	Thrombom	Protein Co	44	GC20M023	27.4
93	IGF1	Insulin Like	Protein Co	50	GC12M102	27.28
94	MECP2	Methyl-Cp	Protein Co	45	GC0XM154	27.15
95	LBR	Lamin B R	Protein Co	47	GC01M225	27.06
96	TERT	Telomeras	Protein Co	51	GC05M001	27.04
97	IL2	Interleukin	Protein Co	45	GC04M122	27.03
98	PDGFRA	Platelet De	Protein Co	55	GC04P054	26.92
99	CCR6	C-C Motif	Protein Co	44	GC06P167	26.9
100	PPARG	Peroxisom	Protein Co	52	GC03P012	26.89
101	ENG	Endoglin	Protein Co	46	GC09M127	26.89
102	IL23R	Interleukin	Protein Co	44	GC01P067	26.82
103	VEGFA	Vascular E	Protein Co	48	GC06P043	26.77
104	ITGB2	Integrin Su	Protein Co	50	GC21M044	26.76
105	GUCY2C	Guanylate	Protein Co	45	GC12M014	26.76
106	XIAP	X-Linked In	Protein Co	49	GC0XP123	26.68
107	ENO2	Enolase 2	Protein Co	47	GC12P006	26.59
108	CD40LG	CD40 Liga	Protein Co	47	GC0XP136	26.59
109	HLA-A	Major Hist	Protein Co	46	GC06P047	26.58
110	MEN1	Menin 1	Protein Co	46	GC11M064	26.48
111	SMAD3	SMAD Fam	Protein Co	49	GC15P067	26.34
112	FGFR2	Fibroblast	Protein Co	54	GC10M121	26.33
113	NGF	Nerve Gro	Protein Co	50	GC01M115	26.23
114	TNFAIP3	TNF Alpha	Protein Co	48	GC06P137	26.22
115	IRF1	Interferon	Protein Co	47	GC05M132	26.12
116	NIPBL	NIPBL Coh	Protein Co	40	GC05P036	26.02
117	HLA-DQB1	Major Hist	Protein Co	44	GC06M032	25.81
118	PTGS2	Prostaglan	Protein Co	48	GC01M186	25.81
119	IL17A	Interleukin	Protein Co	42	GC06P052	25.68
120	TCF4	Transcripti	Protein Co	46	GC18M055	25.68
121	GCG	Glucagon	Protein Co	41	GC02M162	25.62
122	JAK2	Janus Kina	Protein Co	54	GC09P004	25.62
123	CDKN1A	Cyclin Dep	Protein Co	50	GC06P047	25.55
124	SST	Somatosta	Protein Co	42	GC03M187	25.53
125	DMD	Dystrophir	Protein Co	46	GC0XM031	25.53
126	NOTCH2	Notch Rec	Protein Co	50	GC01M119	25.53
127	IRF5	Interferon	Protein Co	48	GC07P128	25.48

128	CCL2	C-C Motif	Protein Co	48	GC17P034	25.35
129	PDGFRB	Platelet De	Protein Co	55	GC05M150	25.34
130	SLC2A1	Solute Car	Protein Co	52	GC01M042	25.31
131	LEP	Leptin	Protein Co	47	GC07P128	25.27
132	CD55	CD55 Mole	Protein Co	47	GC01P207	25.23
133	SKIV2L	Ski2 Like R	Protein Co	43	GC06P047	25.21
134	NOS3	Nitric Oxid	Protein Co	51	GC07P150	25.03
135	APOE	Apolipopro	Protein Co	50	GC19P044	24.94
136	SPP1	Secreted P	Protein Co	46	GC04P087	24.91
137	S100B	S100 Calci	Protein Co	45	GC21M047	24.83
138	CXCR4	C-X-C Mot	Protein Co	52	GC02M136	24.78
139	BAX	BCL2 Asso	Protein Co	48	GC19P048	24.59
140	MT-CO1	Mitochond	Protein Co	32	GCMTP005	24.5
141	ACTG2	Actin Gam	Protein Co	45	GC02P073	24.46
142	LCT	Lactase	Protein Co	43	GC02M135	24.46
143	MEFV	MEFV Inna	Protein Co	43	GC16M003	24.4
144	CDKN1B	Cyclin Dep	Protein Co	48	GC12P012	24.4
145	FABP2	Fatty Acid	Protein Co	43	GC04M119	24.39
146	NF1	Neurofibro	Protein Co	48	GC17P031	24.34
147	F2	Coagulatio	Protein Co	48	GC11P046	24.15
148	ITGAM	Integrin Su	Protein Co	46	GC16P031	24.12
149	CALCA	Calcitonin	Protein Co	43	GC11M014	24.09
150	PLAU	Plasminog	Protein Co	51	GC10P073	24.02
151	GREM1	Gremlin 1,	Protein Co	44	GC15P032	23.99
152	EGF	Epidermal	Protein Co	51	GC04P109	23.95
153	BRCA1	BRCA1 DN	Protein Co	50	GC17M043	23.91
154	SMAD7	SMAD Fam	Protein Co	43	GC18M048	23.9
155	RAD21	RAD21 Col	Protein Co	45	GC08M116	23.88
156	CDX2	Caudal Typ	Protein Co	43	GC13M027	23.82
157	SPINK5	Serine Pep	Protein Co	41	GC05P148	23.82
158	C4A	Compleme	Protein Co	42	GC06P047	23.77
159	ECE1	Endothelin	Protein Co	47	GC01M021	23.74
160	ATP8B1	ATPase Ph	Protein Co	40	GC18M057	23.73
161	DNMT1	DNA Meth	Protein Co	50	GC19M010	23.7
162	APOB	Apolipopro	Protein Co	45	GC02M020	23.7
163	NRAS	NRAS Prot	Protein Co	50	GC01M114	23.67
164	TSC1	TSC Comp	Protein Co	48	GC09M132	23.66
165	TTC37	Tetratricop	Protein Co	39	GC05M095	23.58
166	CD4	CD4 Molec	Protein Co	49	GC12P006	23.53
167	IL18	Interleukin	Protein Co	44	GC11M112	23.52
168	ODC1	Ornithine I	Protein Co	47	GC02M010	23.48
169	SLC5A1	Solute Car	Protein Co	49	GC22P032	23.47
170	SDHB	Succinate I	Protein Co	47	GC01M017	23.47

171	NFE2L2	Nuclear Fa	Protein Co	48	GC02M177	23.44
172	HFE	Homeostat	Protein Co	43	GC06P026	23.4
173	STAT4	Signal Trar	Protein Co	45	GC02M191	23.37
174	TH	Tyrosine H	Protein Co	51	GC11M002	23.34
175	GPT	Glutamic--	Protein Co	41	GC08P144	23.3
176	HLA-DQA1	Major Hist	Protein Co	42	GC06P047	23.28
177	ATG16L1	Autophagy	Protein Co	43	GC02P233	23.1
178	BRCA2	BRCA2 DN	Protein Co	49	GC13P032	22.9
179	IRGM	Immunity I	Protein Co	38	GC05P150	22.87
180	CD46	CD46 Mole	Protein Co	46	GC01P207	22.86
181	FOXF1	Forkhead E	Protein Co	41	GC16P086	22.85
182	ITGB4	Integrin Su	Protein Co	48	GC17P075	22.83
183	ACTA2	Actin Alph	Protein Co	48	GC10M088	22.68
184	PIK3R1	Phosphoin	Protein Co	51	GC05P068	22.62
185	FBN1	Fibrillin 1	Protein Co	45	GC15M048	22.61
186	MIR126	MicroRNA	RNA Gene	22	GC09P136	22.56
187	IL2RA	Interleukin	Protein Co	50	GC10M006	22.56
188	FGFR1	Fibroblast	Protein Co	55	GC08M038	22.49
189	IL21	Interleukin	Protein Co	43	GC04M122	22.47
190	FAT4	FAT Atypic	Protein Co	40	GC04P125	22.46
191	SOD1	Superoxide	Protein Co	51	GC21P031	22.41
192	MYO5B	Myosin VB	Protein Co	44	GC18M049	22.36
193	BMP2	Bone Morp	Protein Co	47	GC20P006	22.29
194	MIR155	MicroRNA	RNA Gene	18	GC21P025	22.28
195	PRF1	Perforin 1	Protein Co	45	GC10M070	22.19
196	MBL2	Mannose E	Protein Co	47	GC10M052	22.14
197	MAP2K1	Mitogen-A	Protein Co	54	GC15P066	22.11
198	DPYD	Dihydropyr	Protein Co	51	GC01M097	22.1
199	IGF2	Insulin Like	Protein Co	48	GC11M002	22.09
200	CHAT	Choline O-	Protein Co	47	GC10P049	22.03
201	CD79A	CD79a Mo	Protein Co	46	GC19P041	22.03
202	HTR2A	5-Hydroxy	Protein Co	47	GC13M046	21.97
203	FLCN	Folliculin	Protein Co	41	GC17M017	21.96
204	HPRT1	Hypoxanth	Protein Co	48	GC0XP134	21.92
205	MITF	Melanocyt	Protein Co	47	GC03P069	21.89
206	GRIN2B	Glutamate	Protein Co	50	GC12M013	21.85
207	SRC	SRC Proto-	Protein Co	51	GC20P037	21.83
208	TEK	TEK Recep	Protein Co	50	GC09P027	21.82
209	MIR146A	MicroRNA	RNA Gene	22	GC05P160	21.8
210	FCGR2A	Fc Fragme	Protein Co	45	GC01P161	21.79
211	MIR145	MicroRNA	RNA Gene	21	GC05P149	21.78
212	H19	H19 Imprir	RNA Gene	28	GC11M001	21.77
213	SDHD	Succinate I	Protein Co	44	GC11P112	21.63

214	SLC6A19	Solute Car	Protein Co	44	GC05P001	21.59
215	EDN1	Endothelin	Protein Co	47	GC06P012	21.58
216	ELANE	Elastase, N	Protein Co	46	GC19P000	21.56
217	HLA-DPB1	Major Hist	Protein Co	43	GC06P047	21.56
218	GAST	Gastrin	Protein Co	40	GC17P041	21.53
219	PLA2G2A	Phospholip	Protein Co	45	GC01M019	21.53
220	F7	Coagulatio	Protein Co	47	GC13P113	21.5
221	MT-ND1	Mitochond	Protein Co	32	GCMTP003	21.49
222	NPY	Neuropept	Protein Co	45	GC07P024	21.33
223	ABCB4	ATP Bindir	Protein Co	45	GC07M087	21.31
224	C3	Compleme	Protein Co	47	GC19M006	21.29
225	SLC6A4	Solute Car	Protein Co	47	GC17M030	21.27
226	BMPR1A	Bone Morp	Protein Co	51	GC10P086	21.26
227	IL13	Interleukin	Protein Co	44	GC05P132	21.25
228	MIR17	MicroRNA	RNA Gene	21	GC13P091	21.23
229	SLC10A2	Solute Car	Protein Co	43	GC13M103	21.19
230	CDK4	Cyclin Dep	Protein Co	54	GC12M057	21.19
231	TFF3	Trefoil Fac	Protein Co	41	GC21M042	21.07
232	SMC1A	Structural	Protein Co	45	GC0XM053	21.07
233	COL3A1	Collagen T	Protein Co	47	GC02P188	21.07
234	F8	Coagulatio	Protein Co	45	GC0XM154	21.05
235	TOR1A	Torsin Fam	Protein Co	44	GC09M129	20.9
236	FASLG	Fas Ligand	Protein Co	47	GC01P172	20.84
237	HLA-DPA1	Major Hist	Protein Co	40	GC06M033	20.83
238	SYP	Synaptoph	Protein Co	43	GC0XM049	20.76
239	MUC2	Mucin 2, C	Protein Co	38	GC11P001	20.74
240	CCK	Cholecystc	Protein Co	41	GC03M042	20.72
241	ITGA6	Integrin Su	Protein Co	49	GC02P172	20.63
242	F9	Coagulatio	Protein Co	46	GC0XP139	20.58
243	ACE	Angiotensi	Protein Co	49	GC17P063	20.55
244	HMOX1	Heme Oxy	Protein Co	52	GC22P035	20.54
245	MLH1	MutL Hom	Protein Co	48	GC03P036	20.54
246	APP	Amyloid Be	Protein Co	51	GC21M025	20.5
247	PTPN11	Protein Tyr	Protein Co	53	GC12P112	20.48
248	IQSEC2	IQ Motif A	Protein Co	37	GC0XM053	20.45
249	TAC1	Tachykinin	Protein Co	43	GC07P097	20.43
250	NCF2	Neutrophil	Protein Co	47	GC01M183	20.4
251	MYD88	MYD88 Inr	Protein Co	50	GC03P038	20.23
252	B2M	Beta-2-Mic	Protein Co	48	GC15P044	20.15
253	HDAC8	Histone De	Protein Co	47	GC0XM072	20.15
254	PRKAR1A	Protein Kir	Protein Co	51	GC17P068	20.14
255	CD36	CD36 Mole	Protein Co	48	GC07P080	20.13
256	RAF1	Raf-1 Prot	Protein Co	54	GC03M012	20.13

257	MIR223	MicroRNA	RNA Gene	21	GC0XP066	20.08
258	CAT	Catalase	Protein Co	50	GC11P034	20.07
259	PHOX2B	Paired Like	Protein Co	43	GC04M041	19.99
260	SDHC	Succinate I	Protein Co	44	GC01P161	19.84
261	MIR483	MicroRNA	RNA Gene	18	GC11M002	19.83
262	MUC5AC	Mucin 5AC	Protein Co	39	GC11P001	19.78
263	MT-CYB	Mitochond	Protein Co	31	GCMTP014	19.77
264	HIF1A	Hypoxia In	Protein Co	47	GC14P061	19.75
265	CDKN2B	Cyclin Dep	Protein Co	47	GC09M022	19.75
266	ACHE	Acetylcholi	Protein Co	45	GC07M100	19.71
267	MYO9B	Myosin IXE	Protein Co	44	GC19P023	19.7
268	BCL2	BCL2 Apop	Protein Co	51	GC18M063	19.67
269	SMC3	Structural	Protein Co	45	GC10P110	19.66
270	TGFBR1	Transformi	Protein Co	52	GC09P099	19.65
271	MIR143	MicroRNA	RNA Gene	22	GC05P149	19.64
272	ELN	Elastin	Protein Co	44	GC07P074	19.63
273	LCN2	Lipocalin 2	Protein Co	43	GC09P128	19.62
274	ADAMTS13	ADAM Me	Protein Co	45	GC09P133	19.6
275	MAPK1	Mitogen-A	Protein Co	51	GC22M021	19.57
276	IL1A	Interleukin	Protein Co	44	GC02M112	19.53
277	MIR192	MicroRNA	RNA Gene	21	GC11M064	19.52
278	F3	Coagulatio	Protein Co	45	GC01M094	19.49
279	MIR34A	MicroRNA	RNA Gene	22	GC01M009	19.46
280	RIPK1	Receptor I	Protein Co	49	GC06P003	19.45
281	KRT20	Keratin 20	Protein Co	41	GC17M040	19.44
282	IL5	Interleukin	Protein Co	44	GC05M132	19.34
283	AXIN1	Axin 1	Protein Co	47	GC16M000	19.31
284	CTNNA1	Catenin Al	Protein Co	47	GC05P138	19.28
285	CASP10	Caspase 10	Protein Co	48	GC02P201	19.28
286	HGF	Hepatocyte	Protein Co	52	GC07M081	19.23
287	ACVRL1	Activin A F	Protein Co	50	GC12P051	19.2
288	MAPT	Microtubul	Protein Co	50	GC17P045	19.09
289	H2AC18	H2A Cluste	Protein Co	26	GC01M149	19.06
290	FBN2	Fibrillin 2	Protein Co	41	GC05M128	19.05
291	JUN	Jun Proto-	Protein Co	49	GC01M058	18.97
292	TLR5	Toll Like R	Protein Co	47	GC01M223	18.96
293	FLT1	Fms Relate	Protein Co	51	GC13M028	18.96
294	ABCB11	ATP Bindir	Protein Co	45	GC02M168	18.91
295	RB1	RB Transcr	Protein Co	49	GC13P048	18.87
296	SDHA	Succinate I	Protein Co	46	GC05P000	18.86
297	UCHL1	Ubiquitin C	Protein Co	51	GC04P041	18.83
298	CYBB	Cytochrom	Protein Co	47	GC0XP037	18.83
299	MIR150	MicroRNA	RNA Gene	21	GC19M049	18.79

300	NKX2-1	NK2 Home	Protein Co	46	GC14M036	18.78
301	HP	Haptoglob	Protein Co	44	GC16P072	18.75
302	CYP3A4	Cytochrom	Protein Co	48	GC07M099	18.74
303	SCT	Secretin	Protein Co	35	GC11M000	18.7
304	FGF2	Fibroblast	Protein Co	47	GC04P122	18.66
305	CFH	Compleme	Protein Co	45	GC01P196	18.62
306	TLR9	Toll Like R	Protein Co	45	GC03M052	18.6
307	NOTCH3	Notch Rec	Protein Co	49	GC19M015	18.59
308	PDGFB	Platelet De	Protein Co	50	GC22M045	18.58
309	MUTYH	MutY DNA	Protein Co	44	GC01M045	18.56
310	ARID1A	AT-Rich In	Protein Co	44	GC01P026	18.53
311	GHRL	Ghrelin An	Protein Co	43	GC03M010	18.52
312	MT-ND4	Mitochond	Protein Co	31	GCMTP010	18.52
313	FUT2	Fucosyltrar	Protein Co	44	GC19P048	18.49
314	SELP	Selectin P	Protein Co	45	GC01M169	18.45
315	FGFR3	Fibroblast	Protein Co	55	GC04P001	18.45
316	SLC15A1	Solute Car	Protein Co	44	GC13M098	18.4
317	SFTPC	Surfactant	Protein Co	43	GC08P022	18.36
318	SERPINA3	Serpin Fan	Protein Co	43	GC14P094	18.35
319	GJB2	Gap Juncti	Protein Co	47	GC13M020	18.34
320	U2AF1	U2 Small N	Protein Co	41	GC21M043	18.33
321	PYY	Peptide YY	Protein Co	42	GC17M043	18.28
322	MYCN	MYCN Pro	Protein Co	46	GC02P015	18.26
323	FOS	Fos Proto-	Protein Co	50	GC14P075	18.21
324	MVK	Mevalonat	Protein Co	48	GC12P109	18.21
325	GH1	Growth Ho	Protein Co	44	GC17M063	18.19
326	C1S	Compleme	Protein Co	45	GC12P008	18.17
327	IL1R1	Interleukin	Protein Co	45	GC02P102	18.16
328	TBX1	T-Box Tran	Protein Co	42	GC22P019	18.14
329	BACH2	BTB Doma	Protein Co	40	GC06M089	18.13
330	MUC1	Mucin 1, C	Protein Co	47	GC01M155	18.13
331	VIPR1	Vasoactive	Protein Co	45	GC03P042	18.09
332	CBLIF	Cobalamin	Protein Co	34	GC11M059	18.04
333	TIMP1	TIMP Meta	Protein Co	45	GC0XP047	18.02
334	LIG4	DNA Ligas	Protein Co	48	GC13M108	18
335	ICOSLG	Inducible T	Protein Co	39	GC21M044	17.96
336	LMNA	Lamin A/C	Protein Co	47	GC01P156	17.9
337	COL1A1	Collagen T	Protein Co	50	GC17M050	17.85
338	FGA	Fibrinogen	Protein Co	47	GC04M154	17.83
339	ADCYAP1	Adenylate	Protein Co	41	GC18P000	17.83
340	HMGB1	High Mobi	Protein Co	44	GC13M030	17.82
341	KMT2D	Lysine Met	Protein Co	41	GC12M049	17.8
342	CD8A	CD8a Mole	Protein Co	46	GC02M086	17.79

343	TWNK	Twinkle M	Protein Co	33	GC10P100	17.79
344	CIITA	Class II Ma	Protein Co	45	GC16P010	17.77
345	KIFBP	Kinesin Fa	Protein Co	31	GC10P068	17.71
346	MIR221	MicroRNA	RNA Gene	20	GC0XM045	17.71
347	G6PD	Glucose-6-	Protein Co	50	GC0XM154	17.7
348	CR1	Compleme	Protein Co	44	GC01P207	17.69
349	IL2RB	Interleukin	Protein Co	48	GC22M037	17.69
350	SHH	Sonic Hed	Protein Co	50	GC07M155	17.68
351	WARS1	Tryptopha	Protein Co	37	GC14M100	17.67
352	MMP2	Matrix Me	Protein Co	53	GC16P055	17.64
353	TTC21B	Tetratricop	Protein Co	38	GC02M165	17.62
354	ATRX	ATRX Chro	Protein Co	45	GC0XM077	17.58
355	RYR1	Ryanodine	Protein Co	47	GC19P038	17.49
356	FGF7	Fibroblast	Protein Co	41	GC15P049	17.43
357	CCL5	C-C Motif	Protein Co	43	GC17M035	17.36
358	MNX1	Motor Neu	Protein Co	41	GC07M156	17.35
359	FMR1	FMRP Tran	Protein Co	44	GC0XP147	17.34
360	TNFRSF1B	TNF Recep	Protein Co	47	GC01P012	17.29
361	MIR200C	MicroRNA	RNA Gene	21	GC12P008	17.27
362	MMP3	Matrix Me	Protein Co	51	GC11M102	17.27
363	XDH	Xanthine D	Protein Co	47	GC02M031	17.24
364	LRP2	LDL Recep	Protein Co	45	GC02M169	17.21
365	FERMT1	Fermitin Fa	Protein Co	41	GC20M006	17.2
366	MLH3	MutL Hom	Protein Co	42	GC14M075	17.2
367	HSPB1	Heat Shoc	Protein Co	51	GC07P076	17.15
368	MAPK8	Mitogen-A	Protein Co	50	GC10P048	17.13
369	VDR	Vitamin D	Protein Co	51	GC12M047	17.08
370	SERPINC1	Serpin Fan	Protein Co	48	GC01M174	17.07
371	PRL	Prolactin	Protein Co	44	GC06M022	17.03
372	MIR15A	MicroRNA	RNA Gene	16	GC13M050	16.94
373	DHCR7	7-Dehydro	Protein Co	46	GC11M071	16.91
374	CXCL10	C-X-C Mot	Protein Co	44	GC04M076	16.9
375	SPINT2	Serine Pep	Protein Co	43	GC19P038	16.89
376	TNFRSF10E	TNF Recep	Protein Co	50	GC08M023	16.87
377	GABRD	Gamma-Ar	Protein Co	45	GC01P002	16.87
378	MAP2K2	Mitogen-A	Protein Co	53	GC19M004	16.81
379	PIGN	Phosphatic	Protein Co	40	GC18M061	16.79
380	EPO	Erythropoi	Protein Co	41	GC07P100	16.75
381	MAPK10	Mitogen-A	Protein Co	51	GC04M085	16.75
382	MYOCD	Myocardin	Protein Co	41	GC17P012	16.69
383	ARX	Aristaless I	Protein Co	42	GC0XM025	16.68
384	PSTPIP1	Proline-Se	Protein Co	45	GC15P076	16.66
385	SOX9	SRY-Box Tr	Protein Co	47	GC17P072	16.63

386	POLD1	DNA Polyr	Protein Co	45	GC19P050	16.62
387	IL15	Interleukin	Protein Co	40	GC04P141	16.62
388	NGFR	Nerve Gro	Protein Co	45	GC17P049	16.61
389	TERC	Telomeras	RNA Gene	28	GC03M169	16.6
390	EPX	Eosinophil	Protein Co	41	GC17P058	16.6
391	CXCL12	C-X-C Mot	Protein Co	45	GC10M044	16.57
392	MMP1	Matrix Me	Protein Co	51	GC11M102	16.57
393	C4B	Compleme	Protein Co	41	GC06P032	16.55
394	CCL3	C-C Motif	Protein Co	39	GC17M036	16.53
395	CASP8	Caspase 8	Protein Co	52	GC02P201	16.52
396	SBDS	SBDS Ribo	Protein Co	43	GC07M066	16.52
397	ETS1	ETS Proto-	Protein Co	49	GC11M128	16.51
398	NOS1	Nitric Oxid	Protein Co	49	GC12M117	16.48
399	MBP	Myelin Bas	Protein Co	44	GC18M076	16.48
400	CTC1	CST Telom	Protein Co	35	GC17M008	16.44
401	FCGR3B	Fc Fragme	Protein Co	42	GC01M161	16.42
402	BCOR	BCL6 Core	Protein Co	41	GC0XM040	16.41
403	CSF3	Colony Sti	Protein Co	40	GC17P040	16.39
404	POMC	Proopiome	Protein Co	48	GC02M025	16.38
405	ADCY10	Adenylate	Protein Co	45	GC01M167	16.36
406	DNMT3A	DNA Meth	Protein Co	51	GC02M025	16.35
407	TLR3	Toll Like R	Protein Co	52	GC04P186	16.33
408	SELE	Selectin E	Protein Co	44	GC01M169	16.33
409	ASCL1	Achaete-S	Protein Co	44	GC12P102	16.32
410	MTTP	Microsoma	Protein Co	44	GC04P099	16.31
411	IDH1	Isocitrate [	Protein Co	52	GC02M208	16.27
412	PSEN1	Presenilin	Protein Co	52	GC14P073	16.24
413	F10	Coagulatio	Protein Co	48	GC13P113	16.23
414	PTGS1	Prostaglan	Protein Co	46	GC09P122	16.23
415	HAMP	Hepcidin A	Protein Co	44	GC19P038	16.21
416	L1CAM	L1 Cell Ad	Protein Co	46	GC0XM153	16.2
417	BTNL2	Butyrophili	Protein Co	39	GC06M032	16.15
418	C1R	Compleme	Protein Co	46	GC12M007	16.15
419	MT-CO2	Mitochonc	Protein Co	32	GCMTP007	16.15
420	MTHFR	Methylene	Protein Co	47	GC01M011	16.05
421	KDR	Kinase Ins	Protein Co	53	GC04M055	16.05
422	NR1H4	Nuclear Re	Protein Co	48	GC12P100	16.04
423	TKT	Transketol	Protein Co	46	GC03M053	16.04
424	MIR29B1	MicroRNA	RNA Gene	21	GC07M130	16.03
425	MAPK14	Mitogen-A	Protein Co	51	GC06P047	15.99
426	MTR	5-Methylte	Protein Co	46	GC01P236	15.98
427	DRD2	Dopamine	Protein Co	50	GC11M113	15.97
428	CAV1	Caveolin 1	Protein Co	48	GC07P116	15.96

429	MIR27A	MicroRNA	RNA Gene	22	GC19M014	15.96
430	ADAM17	ADAM Me	Protein Co	51	GC02M009	15.95
431	F13A1	Coagulatio	Protein Co	45	GC06M006	15.94
432	CD34	CD34 Mol	Protein Co	43	GC01M207	15.94
433	ADAMTS3	ADAM Me	Protein Co	41	GC04M072	15.93
434	F11	Coagulatio	Protein Co	45	GC04P186	15.91
435	MIR140	MicroRNA	RNA Gene	22	GC16P069	15.86
436	CCR1	C-C Motif	Protein Co	45	GC03M046	15.82
437	NLRP3	NLR Family	Protein Co	47	GC01P247	15.78
438	CFI	Compleme	Protein Co	46	GC04M109	15.76
439	CENPF	Centromer	Protein Co	42	GC01P214	15.76
440	AURKA	Aurora Kin	Protein Co	50	GC20M056	15.75
441	NTS	Neurotens	Protein Co	40	GC12P085	15.75
442	HNF1B	HNF1 Hon	Protein Co	44	GC17M037	15.7
443	TJP1	Tight Junct	Protein Co	43	GC15M029	15.68
444	PLVAP	Plasmalem	Protein Co	38	GC19M017	15.66
445	IFNA1	Interferon	Protein Co	39	GC09P021	15.63
446	KMT2A	Lysine Met	Protein Co	44	GC11P118	15.63
447	GSTP1	Glutathion	Protein Co	50	GC11P067	15.63
448	NFKB1	Nuclear Fa	Protein Co	52	GC04P102	15.63
449	VANGL1	VANGL Pla	Protein Co	43	GC01P115	15.63
450	CYBA	Cytochrom	Protein Co	47	GC16M088	15.6
451	JAG1	Jagged Ca	Protein Co	50	GC20M010	15.57
452	WDR19	WD Repea	Protein Co	38	GC04P039	15.57
453	EPHB2	EPH Recep	Protein Co	51	GC01P022	15.56
454	NTF3	Neurotrop	Protein Co	43	GC12P005	15.56
455	PLA2G6	Phospholip	Protein Co	47	GC22M046	15.55
456	KRT7	Keratin 7	Protein Co	41	GC12P052	15.52
457	GGT1	Gamma-Gl	Protein Co	46	GC22P024	15.5
458	TNFSF15	TNF Super	Protein Co	45	GC09M114	15.46
459	CYCS	Cytochrom	Protein Co	48	GC07M025	15.45
460	CSF2	Colony Sti	Protein Co	44	GC05P132	15.45
461	TMEM67	Transmem	Protein Co	37	GC08P093	15.44
462	SIL1	SIL1 Nucle	Protein Co	41	GC05M138	15.43
463	SCN11A	Sodium Vo	Protein Co	41	GC03M038	15.43
464	SMARCA4	SWI/SNF R	Protein Co	50	GC19P010	15.42
465	PTH	Parathyroi	Protein Co	47	GC11M013	15.4
466	IFT140	Intraflagell	Protein Co	39	GC16M001	15.4
467	ABL1	ABL Proto-	Protein Co	52	GC09P130	15.39
468	GPC3	Glypican 3	Protein Co	45	GC0XM133	15.39
469	MPZ	Myelin Pro	Protein Co	44	GC01M161	15.39
470	KNG1	Kininogen	Protein Co	44	GC03P186	15.38
471	CD44	CD44 Mol	Protein Co	47	GC11P035	15.38

472	NCAM1	Neural Cel	Protein Co	45	GC11P112	15.38
473	MIR210	MicroRNA	RNA Gene	21	GC11M000	15.33
474	POLE	DNA Polyr	Protein Co	48	GC12M132	15.33
475	NOTCH1	Notch Rec	Protein Co	51	GC09M136	15.31
476	MIR122	MicroRNA	RNA Gene	21	GC18P058	15.3
477	MIR93	MicroRNA	RNA Gene	20	GC07M100	15.28
478	APOA1	Apolipopro	Protein Co	48	GC11M116	15.26
479	NLRP1	NLR Family	Protein Co	44	GC17M005	15.26
480	DPP4	Dipeptidyl	Protein Co	50	GC02M161	15.25
481	IL12A	Interleukin	Protein Co	44	GC03P159	15.25
482	VCAM1	Vascular C	Protein Co	45	GC01P100	15.24
483	MIR15B	MicroRNA	RNA Gene	18	GC03P160	15.24
484	MGAM	Maltase-Gl	Protein Co	41	GC07P145	15.24
485	GATA1	GATA Bind	Protein Co	46	GC0XP048	15.18
486	MIRLET7A1	MicroRNA	RNA Gene	21	GC09P094	15.17
487	MUC6	Mucin 6, C	Protein Co	37	GC11M001	15.15
488	PLEC	Plectin	Protein Co	42	GC08M143	15.14
489	DES	Desmin	Protein Co	48	GC02P219	15.13
490	COL5A1	Collagen T	Protein Co	45	GC09P134	15.1
491	WNT2B	Wnt Family	Protein Co	44	GC01P112	15.08
492	CCR9	C-C Motif	Protein Co	40	GC03P045	15.07
493	ALPI	Alkaline Ph	Protein Co	43	GC02P232	15.05
494	PIK3C2A	Phosphatic	Protein Co	47	GC11M017	15.05
495	SMARCB1	SWI/SNF R	Protein Co	45	GC22P023	15.04
496	ADIPOQ	Adiponecti	Protein Co	45	GC03P186	15.03
497	PLAT	Plasminog	Protein Co	49	GC08M042	15.02
498	SAA1	Serum Am	Protein Co	42	GC11P018	15.01
499	PPARA	Peroxisom	Protein Co	45	GC22P046	14.97
500	MIR205	MicroRNA	RNA Gene	20	GC01P209	14.96
501	TGM2	Transgluta	Protein Co	48	GC20M038	14.96
502	WFS1	Wolframin	Protein Co	45	GC04P006	14.93
503	RHOA	Ras Homo	Protein Co	46	GC03M049	14.91
504	SCN1A	Sodium Vo	Protein Co	47	GC02M165	14.91
505	FN1	Fibronectir	Protein Co	50	GC02M215	14.89
506	GAPDH	Glyceralde	Protein Co	48	GC12P008	14.88
507	BMP6	Bone Morp	Protein Co	43	GC06P007	14.86
508	CDKL5	Cyclin Dep	Protein Co	43	GC0XP018	14.82
509	PMP22	Peripheral	Protein Co	40	GC17M015	14.82
510	LTF	Lactotransf	Protein Co	43	GC03M046	14.81
511	CCR5	C-C Motif	Protein Co	46	GC03P046	14.79
512	GZMB	Granzyme	Protein Co	45	GC14M024	14.73
513	CD80	CD80 Mole	Protein Co	41	GC03M119	14.7
514	COMP	Cartilage C	Protein Co	47	GC19M018	14.7

515	SLC17A5	Solute Car	Protein Co	44	GC06M073	14.69
516	TP63	Tumor Pro	Protein Co	48	GC03P189	14.63
517	ERCC2	ERCC Excis	Protein Co	47	GC19M045	14.63
518	COL7A1	Collagen T	Protein Co	44	GC03M048	14.62
519	UBE2L3	Ubiquitin (	Protein Co	45	GC22P021	14.61
520	OCRL	OCRL Inos	Protein Co	44	GC0XP129	14.61
521	IGFBP3	Insulin Like	Protein Co	45	GC07M045	14.61
522	KIF1A	Kinesin Fa	Protein Co	43	GC02M240	14.61
523	LDLR	Low Densi	Protein Co	49	GC19P011	14.59
524	ATP7A	ATPase Co	Protein Co	45	GC0XP077	14.58
525	BUB1B	BUB1 Mito	Protein Co	49	GC15P040	14.58
526	BLVRB	Biliverdin F	Protein Co	40	GC19M040	14.55
527	LAMC2	Laminin Su	Protein Co	45	GC01P183	14.54
528	CDH17	Cadherin 1	Protein Co	41	GC08M094	14.53
529	ANXA5	Annexin A	Protein Co	46	GC04M121	14.49
530	MIR25	MicroRNA	RNA Gene	20	GC07M100	14.46
531	PAX6	Paired Box	Protein Co	47	GC11M031	14.45
532	ACTB	Actin Beta	Protein Co	49	GC07M005	14.45
533	HSPA4	Heat Shoc	Protein Co	41	GC05P133	14.44
534	CXCL1	C-X-C Mot	Protein Co	43	GC04P073	14.42
535	BIRC3	Baculoviral	Protein Co	46	GC11P102	14.4
536	ESR1	Estrogen R	Protein Co	53	GC06P151	14.38
537	SCN2A	Sodium Vo	Protein Co	47	GC02P165	14.34
538	TCN2	Transcobal	Protein Co	44	GC22P030	14.34
539	NDUFA13	NADH:Ubi	Protein Co	44	GC19P019	14.34
540	KRT18	Keratin 18	Protein Co	48	GC12P052	14.33
541	CFB	Compleme	Protein Co	45	GC06P031	14.3
542	CILK1	Ciliogenesi	Protein Co	35	GC06M053	14.29
543	CPT2	Carnitine F	Protein Co	48	GC01P053	14.27
544	GLS	Glutaminas	Protein Co	46	GC02P190	14.23
545	VIM	Vimentin	Protein Co	50	GC10P017	14.19
546	COL2A1	Collagen T	Protein Co	48	GC12M047	14.19
547	NFKBIA	NFKB Inhib	Protein Co	50	GC14M035	14.17
548	ABCG2	ATP Bindir	Protein Co	50	GC04M088	14.14
549	CEACAM5	CEA Cell A	Protein Co	41	GC19P041	14.13
550	RTEL1	Regulator	Protein Co	40	GC20P063	14.11
551	MUC5B	Mucin 5B,	Protein Co	41	GC11P001	14.09
552	MAPK3	Mitogen-A	Protein Co	49	GC16M030	14.08
553	IL22	Interleukin	Protein Co	41	GC12M068	14.08
554	CST3	Cystatin C	Protein Co	44	GC20M023	14.07
555	APRT	Adenine Pl	Protein Co	47	GC16M088	14.06
556	FOXP2	Forkhead I	Protein Co	43	GC07P114	14.05
557	FOXC1	Forkhead I	Protein Co	41	GC06P001	14.03

558	ITGA4	Integrin Su	Protein Co		48	GC02P181	14.01
559	ARG1	Arginase 1	Protein Co		50	GC06P131	13.99
560	ALOX5	Arachidona	Protein Co		48	GC10P045	13.99
561	MIR342	MicroRNA	RNA Gene		19	GC14P100	13.99
562	PRDM16	PR/SET Do	Protein Co		45	GC01P003	13.92
563	F12	Coagulatio	Protein Co		48	GC05M177	13.9
564	AOC1	Amine Oxi	Protein Co		40	GC07P150	13.89
565	MMP12	Matrix Me	Protein Co		44	GC11M102	13.88
566	SETD5	SET Domai	Protein Co		38	GC03P009	13.87
567	SOD2	Superoxide	Protein Co		51	GC06M159	13.84
568	GSK3B	Glycogen S	Protein Co		50	GC03M119	13.83
569	SP1	Sp1 Transc	Protein Co		44	GC12P053	13.83
570	PDCD1	Programm	Protein Co		48	GC02M241	13.83
571	CCL4	C-C Motif	Protein Co		40	GC17P036	13.81
572	GP6	Glycoprote	Protein Co		44	GC19M055	13.79
573	NPHP3	Nephrocys	Protein Co		39	GC03M132	13.75
574	IL23A	Interleukin	Protein Co		39	GC12P056	13.75
575	IFT80	Intraflagell	Protein Co		37	GC03M160	13.74
576	CCN2	Cellular Cc	Protein Co		39	GC06M131	13.73
577	TLR1	Toll Like R	Protein Co		47	GC04M038	13.73
578	S100A9	S100 Calci	Protein Co		43	GC01P153	13.71
579	MIR106B	MicroRNA	RNA Gene		21	GC07M100	13.69
580	CR2	Compleme	Protein Co		44	GC01P207	13.69
581	MIR29A	MicroRNA	RNA Gene		21	GC07M130	13.69
582	NAT2	N-Acetyltra	Protein Co		43	GC08P018	13.69
583	SLC2A2	Solute Car	Protein Co		48	GC03M170	13.69
584	FGF10	Fibroblast	Protein Co		47	GC05M044	13.67
585	RELA	RELA Proto	Protein Co		50	GC11M065	13.67
586	NBN	Nibrin	Protein Co		47	GC08M089	13.66
587	MIR107	MicroRNA	RNA Gene		19	GC10M089	13.65
588	TCF7L2	Transcripti	Protein Co		45	GC10P112	13.65
589	CYP1A1	Cytochrom	Protein Co		47	GC15M074	13.64
590	PITX2	Paired Like	Protein Co		47	GC04M110	13.64
591	PKD1	Polycystin	Protein Co		45	GC16M002	13.55
592	AIRE	Autoimmu	Protein Co		44	GC21P044	13.55
593	CALR	Calreticulir	Protein Co		51	GC19P012	13.52
594	HTR3A	5-Hydroxy	Protein Co		45	GC11P113	13.51
595	CTHRC1	Collagen T	Protein Co		41	GC08P103	13.5
596	SH3TC2	SH3 Doma	Protein Co		38	GC05M148	13.49
597	CD40	CD40 Mole	Protein Co		48	GC20P046	13.49
598	CASP9	Caspase 9	Protein Co		48	GC01M015	13.48
599	FOXP1	Forkhead I	Protein Co		45	GC03M070	13.47
600	MDM2	MDM2 Prc	Protein Co		52	GC12P068	13.46

601	CYP27B1	Cytochrom	Protein Co	47	GC12M057	13.46
602	ERAP1	Endoplasm	Protein Co	43	GC05M096	13.43
603	AFP	Alpha Fetc	Protein Co	45	GC04P073	13.42
604	CTSD	Cathepsin	Protein Co	52	GC11M001	13.42
605	IDH2	Isocitrate [	Protein Co	52	GC15M090	13.41
606	ALPP	Alkaline Ph	Protein Co	46	GC02P232	13.41
607	BCL2L1	BCL2 Like	Protein Co	47	GC20M031	13.4
608	PTPRC	Protein Ty	Protein Co	51	GC01P198	13.39
609	HLA-C	Major Hist	Protein Co	44	GC06M031	13.39
610	CASP1	Caspase 1	Protein Co	50	GC11M105	13.37
611	RERE	Arginine-G	Protein Co	41	GC01M008	13.36
612	MT-ND6	Mitochonc	Protein Co	32	GCMTM01	13.36
613	DHCR24	24-Dehydr	Protein Co	45	GC01M054	13.35
614	CEP57	Centrosom	Protein Co	41	GC11P095	13.35
615	KIAA1109	KIAA1109	Protein Co	35	GC04P122	13.32
616	CALB2	Calbindin 2	Protein Co	40	GC16P071	13.3
617	HSPG2	Heparan S	Protein Co	45	GC01M021	13.3
618	RAI1	Retinoic A	Protein Co	40	GC17P017	13.29
619	KCNQ1	Potassium	Protein Co	49	GC11P002	13.29
620	CXCL2	C-X-C Mot	Protein Co	40	GC04M074	13.28
621	MIF	Macropha	Protein Co	49	GC22P023	13.28
622	BMP4	Bone Morp	Protein Co	49	GC14M053	13.28
623	HRH2	Histamine	Protein Co	44	GC05P175	13.27
624	REN	Renin	Protein Co	48	GC01M204	13.25
625	ALK	ALK Recep	Protein Co	51	GC02M029	13.25
626	MIR320A	MicroRNA	RNA Gene	20	GC08M022	13.25
627	MB	Myoglobin	Protein Co	43	GC22M035	13.25
628	MIR32	MicroRNA	RNA Gene	19	GC09M109	13.25
629	IL33	Interleukin	Protein Co	40	GC09P006	13.23
630	SELL	Selectin L	Protein Co	42	GC01M169	13.22
631	CDK2	Cyclin Dep	Protein Co	52	GC12P055	13.22
632	WRN	WRN RecC	Protein Co	45	GC08P031	13.22
633	CLCN4	Chloride V	Protein Co	43	GC0XP010	13.2
634	TMEM216	Transmem	Protein Co	36	GC11P061	13.19
635	AXIN2	Axin 2	Protein Co	48	GC17M065	13.18
636	PRKCA	Protein Kir	Protein Co	50	GC17P066	13.16
637	MED12	Mediator (	Protein Co	44	GC0XP071	13.16
638	MST1	Macropha	Protein Co	44	GC03M049	13.13
639	PIK3CG	Phosphatic	Protein Co	48	GC07P106	13.12
640	PECAM1	Platelet An	Protein Co	40	GC17M064	13.11
641	CYP2C9	Cytochrom	Protein Co	48	GC10P094	13.11
642	TGFB3	Transformi	Protein Co	47	GC14M075	13.1
643	GATA6	GATA Bind	Protein Co	47	GC18P022	13.1

644	SLC7A7	Solute Car	Protein Co	47	GC14M022	13.09
645	GSTM1	Glutathion	Protein Co	41	GC01P109	13.08
646	MSR1	Macropha	Protein Co	46	GC08M016	13.07
647	NR1I2	Nuclear Re	Protein Co	45	GC03P119	13.07
648	HSPD1	Heat Shoc	Protein Co	47	GC02M197	13.04
649	G6PC	Glucose-6-	Protein Co	44	GC17P042	13.04
650	MEG3	Maternally	RNA Gene	29	GC14P104	13.04
651	NPPA	Natriuretic	Protein Co	46	GC01M011	13.02
652	ANPEP	Alanyl Ami	Protein Co	48	GC15M089	13
653	DBH	Dopamine	Protein Co	50	GC09P133	13
654	DNMT3B	DNA Meth	Protein Co	50	GC20P032	12.96
655	S100A8	S100 Calci	Protein Co	42	GC01M153	12.96
656	SAMD9	Sterile Alp	Protein Co	36	GC07M093	12.96
657	CRH	Corticotro	Protein Co	44	GC08M066	12.96
658	HSP90AA1	Heat Shoc	Protein Co	48	GC14M102	12.93
659	DNASE1	Deoxyribo	Protein Co	41	GC16P003	12.91
660	ATP12A	ATPase H+	Protein Co	43	GC13P024	12.9
661	KITLG	KIT Ligand	Protein Co	44	GC12M088	12.9
662	TACR1	Tachykinin	Protein Co	45	GC02M075	12.89
663	TF	Transferrin	Protein Co	49	GC03P133	12.87
664	TRPV4	Transient F	Protein Co	49	GC12M109	12.87
665	WAS	WASP Acti	Protein Co	48	GC0XP048	12.84
666	MT-TK	Mitochond	RNA Gene	14	GCMTP008	12.84
667	SLC22A4	Solute Car	Protein Co	43	GC05P132	12.83
668	STAT5B	Signal Trar	Protein Co	49	GC17M042	12.82
669	CBL	Cbl Proto-	Protein Co	50	GC11P119	12.81
670	HNF1A	HNF1 Hon	Protein Co	45	GC12P120	12.8
671	MIR30E	MicroRNA	RNA Gene	21	GC01P040	12.78
672	MIR34C	MicroRNA	RNA Gene	21	GC11P111	12.78
673	KDM6A	Lysine Den	Protein Co	46	GC0XP044	12.78
674	AMACR	Alpha-Met	Protein Co	45	GC05M033	12.78
675	TFRC	Transferrin	Protein Co	48	GC03M196	12.78
676	GJB1	Gap Juncti	Protein Co	47	GC0XP071	12.77
677	RHO	Rhodopsin	Protein Co	47	GC03P130	12.75
678	THBS1	Thrombos	Protein Co	44	GC15P039	12.72
679	PARP1	Poly(ADP-I	Protein Co	49	GC01M226	12.71
680	CELIAC13	Celiac Dise	Genetic Lo	2	GC12U901	12.71
681	DCHS1	Dachsous	Protein Co	39	GC11M006	12.69
682	NCF4	Neutrophil	Protein Co	48	GC22P036	12.69
683	MCL1	MCL1 Apo	Protein Co	47	GC01M150	12.68
684	CTSG	Cathepsin	Protein Co	44	GC14M024	12.66
685	TNFSF13B	TNF Super	Protein Co	45	GC13P108	12.65
686	ABCA3	ATP Bindir	Protein Co	48	GC16M002	12.65

687	BGLAP	Bone Gam	Protein Co	40	GC01P156	12.64
688	GRP	Gastrin Rel	Protein Co	41	GC18P059	12.62
689	MUSK	Muscle Ass	Protein Co	46	GC09P110	12.61
690	NTRK2	Neurotrop	Protein Co	53	GC09P084	12.61
691	CACNA1A	Calcium Vo	Protein Co	47	GC19M013	12.6
692	MIR127	MicroRNA	RNA Gene	20	GC14P104	12.59
693	MICA	MHC Class	Protein Co	39	GC06P031	12.59
694	ARID1B	AT-Rich In	Protein Co	44	GC06P156	12.58
695	CHEK2	Checkpoin	Protein Co	53	GC22M028	12.58
696	HAVCR1	Hepatitis A	Protein Co	41	GC05M157	12.57
697	HBEGF	Heparin Bi	Protein Co	43	GC05M140	12.57
698	FLVCR1	FLVCR Her	Protein Co	40	GC01P212	12.57
699	SFTPB	Surfactant	Protein Co	43	GC02M085	12.54
700	CYP1A2	Cytochrom	Protein Co	45	GC15P074	12.54
701	PLOD1	Procollage	Protein Co	41	GC01P011	12.53
702	CD14	CD14 Mole	Protein Co	44	GC05M140	12.53
703	CD274	CD274 Mo	Protein Co	44	GC09P005	12.53
704	RAC1	Rac Family	Protein Co	49	GC07P006	12.52
705	ASCC1	Activating	Protein Co	41	GC10M072	12.52
706	CXCR3	C-X-C Mot	Protein Co	44	GC0XM071	12.52
707	SFTPD	Surfactant	Protein Co	44	GC10M079	12.51
708	GGCX	Gamma-Gl	Protein Co	46	GC02M085	12.5
709	NRTN	Neurturin	Protein Co	40	GC19P005	12.49
710	SCN1B	Sodium Vo	Protein Co	44	GC19P035	12.49
711	TCTN2	Tectonic F	Protein Co	39	GC12P123	12.48
712	ADA	Adenosine	Protein Co	51	GC20M044	12.48
713	SMAD2	SMAD Fan	Protein Co	47	GC18M047	12.46
714	SGK1	Serum/Glu	Protein Co	48	GC06M134	12.46
715	CD86	CD86 Mole	Protein Co	43	GC03P122	12.46
716	CD28	CD28 Mole	Protein Co	47	GC02P203	12.45
717	KIF7	Kinesin Fa	Protein Co	39	GC15M089	12.44
718	IFNB1	Interferon	Protein Co	41	GC09M021	12.43
719	GCH1	GTP Cyclol	Protein Co	47	GC14M054	12.43
720	IL7	Interleukin	Protein Co	42	GC08M078	12.42
721	ATP4A	ATPase H+	Protein Co	41	GC19M042	12.4
722	MMP7	Matrix Me	Protein Co	48	GC11M102	12.39
723	CXCR2	C-X-C Mot	Protein Co	48	GC02P218	12.39
724	PLAUR	Plasminog	Protein Co	44	GC19M043	12.39
725	CYP1B1	Cytochrom	Protein Co	48	GC02M038	12.38
726	CDKN3	Cyclin Dep	Protein Co	42	GC14P054	12.37
727	LAMA3	Laminin Su	Protein Co	44	GC18P023	12.37
728	CREB1	CAMP Res	Protein Co	48	GC02P207	12.36
729	HBB	Hemoglob	Protein Co	45	GC11M005	12.35

730	RARB	Retinoic A	Protein Co	50	GC03P024	12.34
731	SMO	Smoothen	Protein Co	48	GC07P129	12.33
732	CDX1	Caudal Typ	Protein Co	36	GC05P150	12.33
733	CHST14	Carbohydr	Protein Co	41	GC15P040	12.33
734	CCL11	C-C Motif	Protein Co	43	GC17P034	12.33
735	CLDN2	Claudin 2	Protein Co	41	GC0XP106	12.29
736	ACTA1	Actin Alph	Protein Co	47	GC01M229	12.29
737	CP	Ceruloplas	Protein Co	47	GC03M149	12.29
738	SOX5	SRY-Box Tı	Protein Co	46	GC12M023	12.29
739	SERPING1	Serpin Fan	Protein Co	46	GC11P057	12.26
740	CELIAC2	Celiac Dise	Genetic Lo	3	GC05U900	12.26
741	CYP2D6	Cytochrom	Protein Co	48	GC22M042	12.26
742	NSD1	Nuclear Re	Protein Co	43	GC05P177	12.25
743	MET	MET Proto	Protein Co	54	GC07P116	12.25
744	SETBP1	SET Bindin	Protein Co	40	GC18P044	12.24
745	PTPN2	Protein Tyı	Protein Co	46	GC18M017	12.24
746	TLR7	Toll Like R	Protein Co	46	GC0XP012	12.23
747	MGMT	O-6-Methy	Protein Co	50	GC10P129	12.22
748	CELIAC10	Celiac Dise	Genetic Lo	2	GC03U901	12.21
749	CELIAC11	Celiac Dise	Genetic Lo	2	GC03U901	12.21
750	CELIAC12	Celiac Dise	Genetic Lo	2	GC06U901	12.21
751	CELIAC5	Celiac Dise	Genetic Lo	2	GC15U900	12.21
752	CELIAC6	Celiac Dise	Genetic Lo	2	GC04U900	12.21
753	CELIAC7	Celiac Dise	Genetic Lo	2	GC01U902	12.21
754	CELIAC8	Celiac Dise	Genetic Lo	2	GC02U901	12.21
755	CELIAC9	Celiac Dise	Genetic Lo	2	GC03U901	12.21
756	TLR6	Toll Like R	Protein Co	44	GC04M038	12.21
757	MIR451A	MicroRNA	RNA Gene	17	GC17M028	12.2
758	IFT81	Intraflagell	Protein Co	39	GC12P110	12.18
759	MME	Membrane	Protein Co	50	GC03P155	12.15
760	LGALS1	Galectin 1	Protein Co	43	GC22P037	12.15
761	LPL	Lipoprotei	Protein Co	49	GC08P019	12.15
762	ITGB1	Integrin Su	Protein Co	50	GC10M032	12.15
763	PLCB1	Phospholip	Protein Co	48	GC20P008	12.14
764	GATA4	GATA Binc	Protein Co	48	GC08P011	12.13
765	GAL	Galanin Ar	Protein Co	44	GC11P068	12.13
766	MIR10A	MicroRNA	RNA Gene	21	GC17M048	12.13
767	TFF1	Trefoil Fac	Protein Co	44	GC21M042	12.11
768	TMEM231	Transmem	Protein Co	37	GC16M075	12.1
769	LTA	Lymphotox	Protein Co	42	GC06P047	12.09
770	ERCC6	ERCC Excis	Protein Co	45	GC10M049	12.09
771	ADRB2	Adrenocep	Protein Co	48	GC05P148	12.06
772	COL1A2	Collagen T	Protein Co	47	GC07P094	12.05

773	AVP	Arginine V	Protein Co	45	GC20M003	12.05
774	CYP2E1	Cytochrom	Protein Co	45	GC10P133	12.05
775	TNXB	Tenascin X	Protein Co	43	GC06M032	12.01
776	MIR199A1	MicroRNA	RNA Gene	18	GC19M010	12.01
777	HSPA8	Heat Shocl	Protein Co	47	GC11M123	12
778	MIR26A1	MicroRNA	RNA Gene	20	GC03P037	12
779	PTK2	Protein Ty	Protein Co	47	GC08M140	11.99
780	CC2D2A	Coiled-Coi	Protein Co	39	GC04P015	11.99
781	LGALS3	Galectin 3	Protein Co	44	GC14P055	11.98
782	CASK	Calcium/C	Protein Co	48	GC0XM041	11.98
783	OCLN	Occludin	Protein Co	44	GC05P069	11.97
784	CSPP1	Centrosom	Protein Co	38	GC08P067	11.96
785	KLF6	Kruppel Lil	Protein Co	44	GC10M003	11.92
786	BCHE	Butyrylcho	Protein Co	48	GC03M165	11.92
787	ANGPT1	Angiopoiet	Protein Co	45	GC08M107	11.91
788	IL12RB1	Interleukin	Protein Co	44	GC19M018	11.9
789	UGT1A1	UDP Glucu	Protein Co	48	GC02P233	11.9
790	WT1	WT1 Trans	Protein Co	49	GC11M032	11.89
791	EGR2	Early Grow	Protein Co	43	GC10M062	11.89
792	NRG1	Neuregulir	Protein Co	46	GC08P031	11.88
793	SLC26A3	Solute Car	Protein Co	45	GC07M107	11.88
794	HMGCR	3-Hydroxy	Protein Co	45	GC05P075	11.88
795	LMOD1	Leiomodin	Protein Co	40	GC01M201	11.87
796	NTRK3	Neurotrop	Protein Co	51	GC15M087	11.87
797	MBD5	Methyl-Cp	Protein Co	37	GC02P148	11.87
798	MYOD1	Myogenic	Protein Co	46	GC11P017	11.85
799	IL7R	Interleukin	Protein Co	47	GC05P035	11.84
800	TNFSF11	TNF Super	Protein Co	47	GC13P042	11.83
801	CSN1S1	Casein Alp	Protein Co	34	GC04P069	11.82
802	KCNAB2	Potassium	Protein Co	43	GC01P006	11.82
803	ATM	ATM Serin	Protein Co	54	GC11P108	11.81
804	NLRC4	NLR Family	Protein Co	44	GC02M032	11.81
805	ADA2	Adenosine	Protein Co	34	GC22M017	11.81
806	SLC4A1	Solute Car	Protein Co	47	GC17M044	11.79
807	GHRH	Growth Hc	Protein Co	40	GC20M037	11.78
808	ABCA1	ATP Bindir	Protein Co	48	GC09M104	11.78
809	ASXL1	ASXL Trans	Protein Co	43	GC20P032	11.77
810	CPLANE1	Ciliogenesi	Protein Co	28	GC05M037	11.76
811	DSP	Desmoplak	Protein Co	49	GC06P007	11.75
812	WNT4	Wnt Family	Protein Co	47	GC01M022	11.73
813	DMPK	DM1 Prote	Protein Co	48	GC19M045	11.73
814	NRXN1	Neurexin 1	Protein Co	47	GC02M049	11.73
815	CYP2C19	Cytochrom	Protein Co	46	GC10P094	11.72

816	TNFRSF10A	TNF Recep	Protein Co		45	GC08M023	11.72
817	IL3	Interleukin	Protein Co		44	GC05P132	11.72
818	EZH2	Enhancer (	Protein Co		54	GC07M148	11.7
819	EPRS1	Glutamyl-F	Protein Co		36	GC01M219	11.7
820	MASP2	Mannan Bi	Protein Co		44	GC01M011	11.68
821	ZFPM2	Zinc Finge	Protein Co		41	GC08P104	11.67
822	ALDH2	Aldehyde I	Protein Co		50	GC12P111	11.66
823	KRT14	Keratin 14	Protein Co		47	GC17M041	11.65
824	LAMB3	Laminin Su	Protein Co		45	GC01M209	11.64
825	GLI3	GLI Family	Protein Co		48	GC07M041	11.64
826	NQO1	NAD(P)H (	Protein Co		49	GC16M069	11.63
827	CHD7	Chromodo	Protein Co		45	GC08P060	11.62
828	TLR8	Toll Like R	Protein Co		47	GC0XP012	11.62
829	TIMP2	TIMP Meta	Protein Co		44	GC17M078	11.6
830	RARA	Retinoic A	Protein Co		50	GC17P040	11.6
831	RNASE3	Ribonuclea	Protein Co		40	GC14P020	11.6
832	FZD4	Frizzled Cl	Protein Co		51	GC11M086	11.59
833	SIRT1	Sirtuin 1	Protein Co		49	GC10P067	11.59
834	SPTAN1	Spectrin Al	Protein Co		47	GC09P128	11.57
835	CTSB	Cathepsin	Protein Co		51	GC08M011	11.55
836	FLG	Filaggrin	Protein Co		40	GC01M152	11.54
837	KCNMA1	Potassium	Protein Co		49	GC10M076	11.54
838	CDC42	Cell Divisic	Protein Co		51	GC01P022	11.54
839	RRM2B	Ribonuclec	Protein Co		49	GC08M102	11.51
840	SOCS1	Suppresso	Protein Co		43	GC16M011	11.51
841	MT-ND4L	Mitochond	Protein Co		28	GCMTP010	11.5
842	CD19	CD19 Mole	Protein Co		49	GC16P029	11.49
843	RPGRIP1L	RPGRIP1 L	Protein Co		39	GC16M053	11.48
844	CNR1	Cannabino	Protein Co		46	GC06M088	11.48
845	ITGA2	Integrin Su	Protein Co		45	GC05P052	11.47
846	APOH	Apolipopro	Protein Co		44	GC17M066	11.46
847	RMRP	RNA Comp	RNA Gene		25	GC09M035	11.45
848	MALAT1	Metastasis	RNA Gene		24	GC11P065	11.45
849	NCF1	Neutrophil	Protein Co		48	GC07P074	11.44
850	PRODH	Proline De	Protein Co		45	GC22M018	11.44
851	ABCC1	ATP Bindir	Protein Co		47	GC16P015	11.44
852	IRAK1	Interleukin	Protein Co		50	GC0XM154	11.41
853	ALG13	ALG13 UD	Protein Co		38	GC0XP111	11.41
854	MT-CO3	Mitochond	Protein Co		30	GCMTP009	11.39
855	POGZ	Pogo Tran	Protein Co		40	GC01M151	11.38
856	ESR2	Estrogen R	Protein Co		49	GC14M064	11.37
857	MFN2	Mitofusin 2	Protein Co		48	GC01P011	11.36
858	DOCK8	Dedicator	Protein Co		43	GC09P000	11.36

859	SEMA3C	Semaphori	Protein Co	41	GC07M080	11.36
860	CDK1	Cyclin Dep	Protein Co	45	GC10P060	11.35
861	KMT2C	Lysine Met	Protein Co	41	GC07M152	11.35
862	DICER1	Dicer 1, Ril	Protein Co	47	GC14M095	11.35
863	MT-TL1	Mitochond	RNA Gene	15	GCMTP003	11.34
864	GP9	Glycoprote	Protein Co	46	GC03P130	11.34
865	HYDIN	HYDIN Axo	Protein Co	37	GC16M070	11.34
866	CASR	Calcium Se	Protein Co	50	GC03P122	11.34
867	ACTC1	Actin Alph	Protein Co	42	GC15M034	11.33
868	GLI2	GLI Family	Protein Co	47	GC02P120	11.33
869	SLC40A1	Solute Car	Protein Co	46	GC02M189	11.31
870	ENO1	Enolase 1	Protein Co	47	GC01M008	11.3
871	DEFB4A	Defensin B	Protein Co	37	GC08P007	11.29
872	TCF3	Transcripti	Protein Co	45	GC19M001	11.28
873	ARAF	A-Raf Prot	Protein Co	45	GC0XP047	11.28
874	ACKR2	Atypical Cl	Protein Co	37	GC03P042	11.27
875	MIR19A	MicroRNA	RNA Gene	19	GC13P091	11.24
876	CAMP	Cathelicidi	Protein Co	41	GC03P048	11.23
877	BIRC5	Baculoviral	Protein Co	47	GC17P078	11.23
878	IARS2	Isoleucyl-T	Protein Co	41	GC01P220	11.22
879	AKT2	AKT Serine	Protein Co	54	GC19M040	11.22
880	PIGA	Phosphatic	Protein Co	44	GC0XM015	11.22
881	ITGAX	Integrin Su	Protein Co	44	GC16P031	11.22
882	EZR	Ezrin	Protein Co	45	GC06M158	11.21
883	NAGLU	N-Acetyl-A	Protein Co	43	GC17P042	11.21
884	GLMN	Glomulin, I	Protein Co	40	GC01M092	11.21
885	TXN	Thioredoxi	Protein Co	45	GC09M110	11.2
886	NOD1	Nucleotide	Protein Co	44	GC07M030	11.19
887	MIR142	MicroRNA	RNA Gene	20	GC17M058	11.18
888	DMBT1	Deleted In	Protein Co	40	GC10P122	11.18
889	HMGA2	High Mobi	Protein Co	44	GC12P065	11.17
890	TRPV1	Transient F	Protein Co	46	GC17M003	11.16
891	GRIN1	Glutamate	Protein Co	49	GC09P137	11.15
892	TNFSF10	TNF Super	Protein Co	46	GC03M172	11.14
893	ANGPT2	Angiopoiet	Protein Co	44	GC08M006	11.13
894	FRAS1	Fraser Extr	Protein Co	39	GC04P078	11.13
895	FLT4	Fms Relate	Protein Co	52	GC05M180	11.11
896	NEUROG3	Neurogeni	Protein Co	41	GC10M069	11.11
897	CXCL9	C-X-C Mot	Protein Co	39	GC04M076	11.11
898	STX1A	Syntaxin 1,	Protein Co	47	GC07M073	11.09
899	HSPA5	Heat Shoc	Protein Co	47	GC09M125	11.08
900	ANXA2	Annexin A2	Protein Co	48	GC15M060	11.08
901	HSD3B7	Hydroxy-D	Protein Co	41	GC16P030	11.04

902	CDKN1C	Cyclin Dep	Protein Co	47	GC11M002	11.04
903	FOXJ1	Forkhead I	Protein Co	36	GC17M076	11.03
904	PCCA	Propionyl-(	Protein Co	45	GC13P100	11.03
905	ABCC6	ATP Bindir	Protein Co	45	GC16M016	10.99
906	EHMT1	Euchromat	Protein Co	45	GC09P137	10.99
907	THBS4	Thrombosp	Protein Co	43	GC05P079	10.97
908	SNAI1	Snail Famil	Protein Co	44	GC20P049	10.97
909	CYP27A1	Cytochrom	Protein Co	47	GC02P218	10.96
910	ERBB3	Erb-B2 Rec	Protein Co	54	GC12P056	10.95
911	MMP8	Matrix Met	Protein Co	47	GC11M102	10.95
912	RREB1	Ras Respo	Protein Co	43	GC06P007	10.94
913	SDC1	Syndecan '	Protein Co	43	GC02M020	10.92
914	CFC1	Cripto, FRL	Protein Co	37	GC02M130	10.91
915	KIF1B	Kinesin Fa	Protein Co	44	GC01P010	10.91
916	MKI67	Marker Of	Protein Co	44	GC10M128	10.9
917	JAK3	Janus Kina	Protein Co	51	GC19M017	10.9
918	CHI3L1	Chitinase 3	Protein Co	43	GC01M203	10.89
919	APEX1	Apurinic/A	Protein Co	45	GC14P020	10.89
920	EPHX1	Epoxide Hy	Protein Co	47	GC01P225	10.86
921	MIR16-1	MicroRNA	RNA Gene	21	GC13M050	10.85
922	TFF2	Trefoil Fac	Protein Co	41	GC21M042	10.85
923	SKI	SKI Proto-(	Protein Co	45	GC01P002	10.84
924	MLN	Motilin	Protein Co	35	GC06M033	10.84
925	DOK7	Docking P	Protein Co	39	GC04P003	10.82
926	IHH	Indian Hec	Protein Co	47	GC02M219	10.82
927	SH2D1A	SH2 Doma	Protein Co	47	GC0XP124	10.82
928	PAX3	Paired Box	Protein Co	47	GC02M222	10.82
929	PLCG2	Phospholip	Protein Co	51	GC16P081	10.8
930	GLP2R	Glucagon I	Protein Co	44	GC17P009	10.77
931	GSR	Glutathion	Protein Co	48	GC08M030	10.77
932	MBTPS2	Membrane	Protein Co	43	GC0XP021	10.75
933	GJC2	Gap Juncti	Protein Co	41	GC01P228	10.75
934	SERPINF1	Serpin Fan	Protein Co	44	GC17P001	10.74
935	CISD2	CDGSH Iro	Protein Co	41	GC04P102	10.74
936	CFLAR	CASP8 And	Protein Co	46	GC02P201	10.73
937	NEAT1	Nuclear Pa	RNA Gene	23	GC11P065	10.73
938	SOX2	SRY-Box Tr	Protein Co	47	GC03P181	10.72
939	S100A4	S100 Calci	Protein Co	44	GC01M153	10.72
940	IL18R1	Interleukin	Protein Co	43	GC02P102	10.72
941	PMM2	Phosphom	Protein Co	47	GC16P008	10.71
942	RAPSN	Receptor A	Protein Co	42	GC11M061	10.7
943	MIR328	MicroRNA	RNA Gene	18	GC16M067	10.69
944	OPRM1	Opioid Rec	Protein Co	48	GC06P154	10.68

945	TPO	Thyroid Pe	Protein Co	48	GC02P001	10.68
946	MT-ATP6	Mitochonc	Protein Co	31	GCMTP008	10.68
947	COG6	Componer	Protein Co	37	GC13P039	10.67
948	TNFRSF13E	TNF Recep	Protein Co	46	GC17M016	10.67
949	EVC2	EvC Ciliary	Protein Co	39	GC04M005	10.66
950	GPR35	G Protein-(	Protein Co	43	GC02P240	10.65
951	TYMS	Thymidylat	Protein Co	47	GC18P000	10.65
952	LAX1	Lymphocyt	Protein Co	36	GC01P203	10.64
953	PGAP3	Post-GPI A	Protein Co	38	GC17M039	10.64
954	IGF1R	Insulin Like	Protein Co	54	GC15P098	10.63
955	NAA10	N-Alpha-A	Protein Co	42	GC0XM153	10.63
956	NPPB	Natriuretic	Protein Co	44	GC01M011	10.63
957	KRT8	Keratin 8	Protein Co	47	GC12M052	10.62
958	KAT6A	Lysine Ace	Protein Co	43	GC08M041	10.6
959	CFHR5	Compleme	Protein Co	40	GC01P196	10.59
960	ITGA2B	Integrin Su	Protein Co	50	GC17M044	10.59
961	CSF1	Colony Stii	Protein Co	43	GC01P109	10.59
962	AGTR1	Angiotensi	Protein Co	51	GC03P148	10.59
963	CD3G	CD3g Mol	Protein Co	47	GC11P118	10.57
964	ABCC3	ATP Bindir	Protein Co	45	GC17P050	10.57
965	KMT2B	Lysine Met	Protein Co	39	GC19P038	10.56
966	ABCG5	ATP Bindir	Protein Co	44	GC02M043	10.55
967	DLC1	DLC1 Rho	Protein Co	44	GC08M013	10.55
968	VCL	Vinculin	Protein Co	47	GC10P073	10.53
969	ABCG8	ATP Bindir	Protein Co	42	GC02P043	10.53
970	TLR10	Toll Like R	Protein Co	40	GC04M038	10.52
971	EGR1	Early Grow	Protein Co	44	GC05P138	10.52
972	MIR30A	MicroRNA	RNA Gene	20	GC06M071	10.51
973	CUBN	Cubilin	Protein Co	46	GC10M016	10.49
974	OFD1	OFD1 Cent	Protein Co	41	GC0XP013	10.49
975	LRBA	LPS Respo	Protein Co	41	GC04M150	10.49
976	IL9	Interleukin	Protein Co	43	GC05M135	10.47
977	SNCA	Synuclein /	Protein Co	50	GC04M089	10.47
978	WWOX	WW Doma	Protein Co	47	GC16P078	10.47
979	IFNA2	Interferon	Protein Co	41	GC09M021	10.44
980	CEP290	Centrosom	Protein Co	40	GC12M088	10.43
981	TBX21	T-Box Tran	Protein Co	45	GC17P047	10.43
982	TRAF6	TNF Recep	Protein Co	47	GC11M036	10.42
983	PTPN12	Protein Ty	Protein Co	45	GC07P077	10.41
984	FANCA	FA Comple	Protein Co	48	GC16M089	10.39
985	MIR125A	MicroRNA	RNA Gene	21	GC19P051	10.36
986	MMP13	Matrix Met	Protein Co	50	GC11M102	10.36
987	S100A12	S100 Calci	Protein Co	39	GC01M153	10.36

988	SIN3A	SIN3 Trans	Protein Co	45	GC15M075	10.35
989	SOS1	SOS Ras/R	Protein Co	47	GC02M038	10.34
990	AQP4	Aquaporin	Protein Co	45	GC18M026	10.33
991	CCBE1	Collagen A	Protein Co	40	GC18M059	10.32
992	KLRK1	Killer Cell I	Protein Co	40	GC12M013	10.31
993	NEK1	NIMA Rela	Protein Co	43	GC04M169	10.3
994	BTD	Biotinidase	Protein Co	44	GC03P015	10.3
995	NR1H2	Nuclear Re	Protein Co	48	GC19P050	10.29
996	GUSB	Glucuronic	Protein Co	47	GC07M065	10.27
997	PAH	Phenylalan	Protein Co	48	GC12M102	10.27
998	GFRA1	GDNF Fam	Protein Co	44	GC10M116	10.25
999	PRDM10	PR/SET Do	Protein Co	34	GC11M129	10.25
1000	LOX	Lysyl Oxida	Protein Co	44	GC05M122	10.25
1001	VCP	Valosin Co	Protein Co	48	GC09M035	10.23
1002	E2F1	E2F Transc	Protein Co	43	GC20M033	10.23
1003	GSN	Gelsolin	Protein Co	48	GC09P121	10.23
1004	PES1	Pescadillo	Protein Co	38	GC22M030	10.23
1005	AARS1	Alanyl-TRN	Protein Co	36	GC16M070	10.22
1006	ST14	ST14 Trans	Protein Co	45	GC11P130	10.21
1007	KLF5	Kruppel Lil	Protein Co	44	GC13P073	10.2
1008	LBP	Lipopolysa	Protein Co	44	GC20P038	10.2
1009	FOXM1	Forkhead I	Protein Co	44	GC12M002	10.2
1010	MIR196A1	MicroRNA	RNA Gene	19	GC17M048	10.19
1011	TCTN1	Tectonic Fa	Protein Co	37	GC12P110	10.18
1012	NDRG1	N-Myc Do	Protein Co	45	GC08M133	10.18
1013	HLA-DQA2	Major Hist	Protein Co	37	GC06P032	10.18
1014	SERPINF2	Serpin Fam	Protein Co	44	GC17P001	10.17
1015	PON1	Paraoxona	Protein Co	45	GC07M095	10.17
1016	OGG1	8-Oxoguar	Protein Co	47	GC03P009	10.16
1017	SOCS3	Suppresso	Protein Co	44	GC17M078	10.16
1018	FHIT	Fragile His	Protein Co	44	GC03M059	10.16
1019	TGFA	Transformi	Protein Co	46	GC02M070	10.15
1020	BAD	BCL2 Asso	Protein Co	46	GC11M064	10.15
1021	HOTAIR	HOX Trans	RNA Gene	25	GC12M053	10.14
1022	SCN9A	Sodium Vo	Protein Co	47	GC02M166	10.14
1023	IGHE	Immunogl	Protein Co	26	GC14M109	10.13
1024	FIG4	FIG4 Phosp	Protein Co	43	GC06P109	10.13
1025	SEMA3A	Semaphori	Protein Co	45	GC07M083	10.12
1026	NR3C1	Nuclear Re	Protein Co	50	GC05M143	10.12
1027	BTK	Bruton Tyr	Protein Co	53	GC0XM101	10.11
1028	MKS1	MKS Trans	Protein Co	40	GC17M058	10.1
1029	COX5A	Cytochrom	Protein Co	43	GC15M074	10.09
1030	IL11	Interleukin	Protein Co	41	GC19M055	10.09

1031	AGER	Advanced	Protein Co	44	GC06M032	10.09
1032	NOX1	NADPH O>	Protein Co	41	GC0XM100	10.06
1033	TIMP3	TIMP Meta	Protein Co	45	GC22P032	10.04
1034	NPC1L1	NPC1 Like	Protein Co	44	GC07M044	10.03
1035	LAMA2	Laminin Su	Protein Co	43	GC06P128	10.02
1036	RUNX3	RUNX Fam	Protein Co	43	GC01M024	10.02
1037	KIDINS220	Kinase D Ir	Protein Co	41	GC02M008	10
1038	MIR9-1	MicroRNA	RNA Gene	20	GC01M156	10
1039	HTR1A	5-Hydroxy	Protein Co	47	GC05M063	10
1040	PTPN3	Protein Tyr	Protein Co	44	GC09M109	9.98
1041	IL6R	Interleukin	Protein Co	48	GC01P154	9.96
1042	GARS1	Glycyl-TRN	Protein Co	36	GC07P030	9.96
1043	FABP1	Fatty Acid	Protein Co	43	GC02M088	9.95
1044	TSLP	Thymic Str	Protein Co	38	GC05P111	9.94
1045	NES	Nestin	Protein Co	39	GC01M156	9.94
1046	TG	Thyroglobu	Protein Co	42	GC08P132	9.94
1047	MIR214	MicroRNA	RNA Gene	20	GC01M172	9.92
1048	LCK	LCK Proto-	Protein Co	52	GC01P032	9.92
1049	MT-TF	Mitochond	RNA Gene	14	GCMTP000	9.92
1050	ABCC2	ATP Bindir	Protein Co	47	GC10P099	9.92
1051	PANK2	Pantothen	Protein Co	43	GC20P003	9.91
1052	TREH	Trehalase	Protein Co	43	GC11M118	9.91
1053	MIR378A	MicroRNA	RNA Gene	18	GC05P149	9.91
1054	ITGAL	Integrin Su	Protein Co	45	GC16P030	9.9
1055	NPC1	NPC Intrac	Protein Co	47	GC18M023	9.89
1056	GDF15	Growth Dif	Protein Co	41	GC19P023	9.88
1057	MIR22	MicroRNA	RNA Gene	20	GC17M001	9.87
1058	ITGAE	Integrin Su	Protein Co	39	GC17M003	9.87
1059	DUOX2	Dual Oxida	Protein Co	43	GC15M045	9.87
1060	STAT6	Signal Trar	Protein Co	50	GC12M057	9.84
1061	CEBPB	CCAAT Enh	Protein Co	43	GC20P050	9.84
1062	IKBKB	Inhibitor C	Protein Co	52	GC08P042	9.83
1063	LRP5	LDL Recep	Protein Co	48	GC11P068	9.83
1064	ACE2	Angiotensi	Protein Co	48	GC0XM015	9.83
1065	TWIST1	Twist Fami	Protein Co	45	GC07M019	9.83
1066	FGB	Fibrinogen	Protein Co	45	GC04P154	9.82
1067	CDH2	Cadherin 2	Protein Co	50	GC18M027	9.82
1068	CCL20	C-C Motif	Protein Co	43	GC02P227	9.82
1069	TNFRSF25	TNF Recep	Protein Co	43	GC01M006	9.8
1070	BPI	Bactericida	Protein Co	41	GC20P038	9.8
1071	ITGB3	Integrin Su	Protein Co	49	GC17P047	9.8
1072	GRIN2A	Glutamate	Protein Co	51	GC16M009	9.79
1073	MICB	MHC Class	Protein Co	41	GC06P047	9.79

1074	APOA4	Apolipopro	Protein Co	42	GC11M116	9.79
1075	MAOA	Monoamin	Protein Co	50	GC0XP043	9.78
1076	SLC2A10	Solute Car	Protein Co	44	GC20P046	9.76
1077	SLC11A1	Solute Car	Protein Co	47	GC02P218	9.76
1078	IL16	Interleukin	Protein Co	42	GC15P081	9.75
1079	C5	Compleme	Protein Co	46	GC09M120	9.74
1080	SV2A	Synaptic V	Protein Co	43	GC01M149	9.74
1081	CLU	Clusterin	Protein Co	46	GC08M027	9.73
1082	PIEZO1	Piezo Type	Protein Co	37	GC16M088	9.73
1083	MMEL1	Membrane	Protein Co	36	GC01M002	9.73
1084	WASHC5	WASH Cor	Protein Co	32	GC08M128	9.72
1085	CCNB1	Cyclin B1	Protein Co	47	GC05P069	9.71
1086	SYK	Spleen Ass	Protein Co	50	GC09P091	9.71
1087	XRCC1	X-Ray Rep	Protein Co	43	GC19M043	9.71
1088	SLC6A8	Solute Car	Protein Co	45	GC0XP153	9.7
1089	GP1BB	Glycoprote	Protein Co	41	GC22P019	9.7
1090	ISX	Intestine S	Protein Co	32	GC22P035	9.7
1091	DDIT3	DNA Dam	Protein Co	45	GC12M057	9.69
1092	PRKCD	Protein Kir	Protein Co	53	GC03P053	9.69
1093	MIR24-1	MicroRNA	RNA Gene	18	GC09P095	9.68
1094	GATA3	GATA Bind	Protein Co	49	GC10P008	9.68
1095	MPV17	Mitochond	Protein Co	41	GC02M027	9.67
1096	CDK6	Cyclin Dep	Protein Co	54	GC07M092	9.67
1097	ALDH18A1	Aldehyde I	Protein Co	45	GC10M095	9.67
1098	DSG2	Desmoglei	Protein Co	45	GC18P031	9.65
1099	FGF8	Fibroblast	Protein Co	47	GC10M101	9.64
1100	IDO1	Indoleamir	Protein Co	45	GC08P039	9.64
1101	PPIG	Peptidylpro	Protein Co	41	GC02P169	9.63
1102	AQP1	Aquaporin	Protein Co	45	GC07P030	9.62
1103	PRUNE1	Prune Exo	Protein Co	32	GC01P151	9.61
1104	LPIN2	Lipin 2	Protein Co	42	GC18M002	9.61
1105	CLDN3	Claudin 3	Protein Co	40	GC07M073	9.61
1106	TNFRSF11E	TNF Recep	Protein Co	47	GC08M118	9.6
1107	DCN	Decorin	Protein Co	47	GC12M091	9.6
1108	JAK1	Janus Kina	Protein Co	50	GC01M064	9.6
1109	PRSS1	Serine Pro	Protein Co	45	GC07P144	9.59
1110	SCGB1A1	Secretoglo	Protein Co	40	GC11P062	9.59
1111	CHGA	Chromogra	Protein Co	42	GC14P092	9.58
1112	INPP5E	Inositol Po	Protein Co	41	GC09M136	9.57
1113	BCL2L11	BCL2 Like	Protein Co	45	GC02P111	9.56
1114	POLR2F	RNA Polyn	Protein Co	40	GC22P037	9.55
1115	OTC	Ornithine (	Protein Co	47	GC0XP038	9.55
1116	LEPR	Leptin Rec	Protein Co	49	GC01P065	9.54

1117	PROM1	Prominin 1	Protein Co	44	GC04M015	9.54
1118	SLC39A4	Solute Car	Protein Co	43	GC08M144	9.53
1119	DNAAF4	Dynein Axo	Protein Co	31	GC15M060	9.53
1120	RPS6KA3	Ribosomal	Protein Co	52	GC0XM020	9.52
1121	AGT	Angiotensi	Protein Co	49	GC01M230	9.5
1122	ALPL	Alkaline Ph	Protein Co	50	GC01P021	9.5
1123	DGKE	Diacylglyce	Protein Co	48	GC17P056	9.49
1124	BECN1	Beclin 1	Protein Co	46	GC17M042	9.48
1125	KEAP1	Kelch Like	Protein Co	48	GC19M010	9.48
1126	DHFR	Dihydrofol	Protein Co	49	GC05M080	9.48
1127	RAP1A	RAP1A, Me	Protein Co	47	GC01P111	9.46
1128	FCGR3A	Fc Fragme	Protein Co	44	GC01M161	9.45
1129	HCRT	Hypocretin	Protein Co	40	GC17M042	9.44
1130	CX3CR1	C-X3-C Mo	Protein Co	44	GC03M039	9.44
1131	BLOC1S1	Biogenesis	Protein Co	36	GC12P055	9.44
1132	SLPI	Secretory I	Protein Co	39	GC20M045	9.44
1133	B9D1	B9 Domain	Protein Co	36	GC17M019	9.44
1134	DNM2	Dynamin 2	Protein Co	49	GC19P010	9.43
1135	NFATC1	Nuclear Fa	Protein Co	47	GC18P079	9.42
1136	MYO5A	Myosin VA	Protein Co	44	GC15M060	9.42
1137	THRA	Thyroid Ho	Protein Co	48	GC17P040	9.41
1138	UBE3B	Ubiquitin F	Protein Co	43	GC12P109	9.39
1139	DAPK1	Death Asso	Protein Co	48	GC09P087	9.39
1140	MIR181A2	MicroRNA	RNA Gene	20	GC09P124	9.39
1141	NSD2	Nuclear Re	Protein Co	35	GC04P001	9.38
1142	RPS6KB1	Ribosomal	Protein Co	49	GC17P059	9.38
1143	FREM2	FRAS1 Rela	Protein Co	39	GC13P038	9.38
1144	NEU1	Neuramini	Protein Co	44	GC06M031	9.37
1145	PRSS3	Serine Pro	Protein Co	43	GC09P033	9.37
1146	BMPR2	Bone Morp	Protein Co	50	GC02P202	9.36
1147	NAGS	N-Acetylgl	Protein Co	42	GC17P044	9.36
1148	JUP	Junction Pl	Protein Co	47	GC17M041	9.35
1149	MIR137	MicroRNA	RNA Gene	18	GC01M098	9.35
1150	CPS1	Carbamoyl	Protein Co	45	GC02P210	9.34
1151	DKK1	Dickkopf W	Protein Co	45	GC10P052	9.34
1152	EFEMP2	EGF Conta	Protein Co	43	GC11M065	9.33
1153	SPINK1	Serine Pep	Protein Co	43	GC05M147	9.33
1154	TRIM8	Tripartite M	Protein Co	38	GC10P102	9.32
1155	ACKR1	Atypical Ch	Protein Co	37	GC01P159	9.31
1156	LAP3	Leucine Ar	Protein Co	43	GC04P017	9.3
1157	PTX3	Pentraxin 3	Protein Co	41	GC03P157	9.29
1158	RINT1	RAD50 Inte	Protein Co	36	GC07P105	9.29
1159	ANXA1	Annexin A1	Protein Co	49	GC09P073	9.29

1160	STK36	Serine/Thr	Protein Co	43	GC02P218	9.28
1161	PTCH2	Patched 2	Protein Co	44	GC01M044	9.27
1162	CHUK	Componer	Protein Co	52	GC10M100	9.27
1163	TCOF1	Treacle Rib	Protein Co	42	GC05P150	9.27
1164	TGIF1	TGFB Indu	Protein Co	45	GC18P003	9.26
1165	ADORA1	Adenosine	Protein Co	47	GC01P203	9.26
1166	KDM4C	Lysine Den	Protein Co	40	GC09P006	9.26
1167	LMOD3	Leiomodin	Protein Co	39	GC03M069	9.25
1168	PSMD4	Proteasom	Protein Co	44	GC01P151	9.25
1169	LACC1	Laccase Do	Protein Co	35	GC13P043	9.24
1170	GPX2	Glutathion	Protein Co	44	GC14M064	9.24
1171	VEGFC	Vascular En	Protein Co	47	GC04M176	9.23
1172	CES1	Carboxyles	Protein Co	46	GC16M055	9.23
1173	IL18RAP	Interleukin	Protein Co	39	GC02P102	9.22
1174	SALL4	Spalt Like	Protein Co	43	GC20M051	9.22
1175	TIA1	TIA1 Cytot	Protein Co	43	GC02M070	9.21
1176	NKX2-5	NK2 Home	Protein Co	44	GC05M173	9.2
1177	ENPP1	Ectonuclec	Protein Co	47	GC06P131	9.19
1178	CCL17	C-C Motif	Protein Co	38	GC16P057	9.19
1179	MIR144	MicroRNA	RNA Gene	16	GC17M029	9.19
1180	GOT2	Glutamic-C	Protein Co	45	GC16M058	9.18
1181	FBXW7	F-Box And	Protein Co	44	GC04M152	9.18
1182	GJA1	Gap Juncti	Protein Co	50	GC06P121	9.18
1183	FBLN5	Fibulin 5	Protein Co	44	GC14M091	9.17
1184	PRKCZ	Protein Kir	Protein Co	48	GC01P002	9.17
1185	RTTN	Rotatin	Protein Co	36	GC18M070	9.16
1186	TREX1	Three Prim	Protein Co	42	GC03P048	9.15
1187	GLI1	GLI Family	Protein Co	47	GC12P057	9.14
1188	ADAMTS2	ADAM Me	Protein Co	42	GC05M179	9.14
1189	BAAT	Bile Acid-C	Protein Co	44	GC09M101	9.14
1190	GRB2	Growth Fa	Protein Co	49	GC17M075	9.13
1191	IRF3	Interferon	Protein Co	47	GC19M049	9.13
1192	WDR35	WD Repea	Protein Co	38	GC02M019	9.12
1193	FCGR1A	Fc Fragme	Protein Co	41	GC01P149	9.11
1194	SLC12A2	Solute Car	Protein Co	46	GC05P128	9.11
1195	HECTD4	HECT Dom	Protein Co	31	GC12M112	9.11
1196	CX3CL1	C-X3-C Mo	Protein Co	42	GC16P057	9.11
1197	BCL6	BCL6 Trans	Protein Co	45	GC03M187	9.1
1198	GAP43	Growth As	Protein Co	43	GC03P115	9.09
1199	PDGFA	Platelet De	Protein Co	44	GC07M000	9.08
1200	SH2B3	SH2B Adap	Protein Co	46	GC12P111	9.08
1201	CXCL5	C-X-C Mot	Protein Co	40	GC04M073	9.08
1202	SULT1A3	Sulfotransf	Protein Co	38	GC16P030	9.07

1203	CCND2	Cyclin D2	Protein Co	50	GC12P008	9.05
1204	SMARCA2	SWI/SNF R	Protein Co	49	GC09P001	9.05
1205	CHRNG	Cholinergi	Protein Co	40	GC02P232	9.04
1206	COL4A1	Collagen T	Protein Co	47	GC13M110	9.04
1207	TET2	Tet Methyl	Protein Co	44	GC04P105	9.03
1208	LEF1	Lymphoid	Protein Co	47	GC04M108	9.03
1209	SRCAP	Snf2 Relat	Protein Co	40	GC16P030	9.03
1210	DDX41	DEAD-Box	Protein Co	43	GC05M177	9.03
1211	VAMP2	Vesicle Ass	Protein Co	43	GC17M008	9.01
1212	CD59	CD59 Mol	Protein Co	46	GC11M033	9.01
1213	DPF2	Double PH	Protein Co	41	GC11P065	9
1214	FOXO3	Forkhead I	Protein Co	44	GC06P108	9
1215	EPHX2	Epoxide H	Protein Co	47	GC08P027	8.99
1216	SLC52A3	Solute Car	Protein Co	39	GC20M000	8.99
1217	RETN	Resistin	Protein Co	43	GC19P007	8.98
1218	NLRP6	NLR Family	Protein Co	38	GC11P000	8.97
1219	LTBP4	Latent Tra	Protein Co	40	GC19P040	8.97
1220	GAS5	Growth Ar	RNA Gene	23	GC01M173	8.97
1221	SALL1	Spalt Like	Protein Co	44	GC16M051	8.96
1222	HLA-G	Major Hist	Protein Co	44	GC06P047	8.95
1223	BMI1	BMI1 Prot	Protein Co	43	GC10P022	8.95
1224	LRRK2	Leucine Ri	Protein Co	49	GC12P040	8.93
1225	BSG	Basigin (Ol	Protein Co	44	GC19P000	8.93
1226	LMNB2	Lamin B2	Protein Co	43	GC19M002	8.93
1227	AIFM1	Apoptosis	Protein Co	49	GC0XM130	8.92
1228	KRT5	Keratin 5	Protein Co	47	GC12M052	8.91
1229	MED13L	Mediator (	Protein Co	39	GC12M115	8.9
1230	SCARB1	Scavenger	Protein Co	45	GC12M124	8.9
1231	AGXT	Alanine--G	Protein Co	45	GC02P240	8.89
1232	AP1S1	Adaptor R	Protein Co	39	GC07P101	8.89
1233	KIAA0586	KIAA0586	Protein Co	36	GC14P058	8.87
1234	MIR124-1	MicroRNA	RNA Gene	21	GC08M009	8.86
1235	FLNB	Filamin B	Protein Co	45	GC03P058	8.85
1236	FOLH1	Folate Hyd	Protein Co	46	GC11M061	8.84
1237	FCGRT	Fc Fragme	Protein Co	40	GC19P049	8.84
1238	LZTR1	Leucine Zi	Protein Co	43	GC22P020	8.83
1239	GNB1	G Protein	Protein Co	45	GC01M001	8.83
1240	UFD1	Ubiquitin F	Protein Co	35	GC22M019	8.83
1241	F2R	Coagulatio	Protein Co	46	GC05P076	8.82
1242	CASP2	Caspase 2	Protein Co	49	GC07P144	8.8
1243	MADCAM1	Mucosal V	Protein Co	37	GC19P000	8.8
1244	CRLF1	Cytokine R	Protein Co	41	GC19M018	8.78
1245	PREP	Prolyl End	Protein Co	42	GC06M105	8.78

1246	RUNX2	RUNX Fam	Protein Co	47	GC06P047	8.77
1247	SLC2A5	Solute Car	Protein Co	43	GC01M009	8.75
1248	AP1S2	Adaptor R	Protein Co	43	GC0XM015	8.74
1249	LPP	LIM Doma	Protein Co	42	GC03P188	8.73
1250	MIR423	MicroRNA	RNA Gene	18	GC17P030	8.72
1251	LGR5	Leucine Ri	Protein Co	43	GC12P071	8.72
1252	STS	Steroid Su	Protein Co	44	GC0XP007	8.71
1253	CYP19A1	Cytochrom	Protein Co	48	GC15M051	8.7
1254	IL12B	Interleukin	Protein Co	44	GC05M159	8.69
1255	NR3C2	Nuclear Re	Protein Co	48	GC04M148	8.69
1256	NUP98	Nucleopor	Protein Co	42	GC11M003	8.69
1257	FGF23	Fibroblast	Protein Co	45	GC12M004	8.69
1258	PLA2G4A	Phospholip	Protein Co	49	GC01P186	8.68
1259	SCG5	Secretogra	Protein Co	38	GC15P032	8.68
1260	THPO	Thrombop	Protein Co	42	GC03M184	8.68
1261	PDX1	Pancreatic	Protein Co	47	GC13P027	8.68
1262	KCNH5	Potassium	Protein Co	43	GC14M062	8.68
1263	CFAP43	Cilia And F	Protein Co	30	GC10M104	8.67
1264	TYR	Tyrosinase	Protein Co	47	GC11P089	8.67
1265	SEC23B	SEC23 Hor	Protein Co	43	GC20P018	8.66
1266	TBCD	Tubulin Fo	Protein Co	41	GC17P082	8.65
1267	DYRK1A	Dual Speci	Protein Co	50	GC21P037	8.65
1268	SLC3A1	Solute Car	Protein Co	47	GC02P044	8.64
1269	CLDN7	Claudin 7	Protein Co	41	GC17M007	8.64
1270	IGFBP2	Insulin Like	Protein Co	43	GC02P216	8.64
1271	STAT5A	Signal Trar	Protein Co	45	GC17P042	8.63
1272	IRS1	Insulin Rec	Protein Co	47	GC02M226	8.63
1273	DSE	Dermatan	Protein Co	41	GC06P116	8.63
1274	CTSL	Cathepsin	Protein Co	46	GC09P087	8.63
1275	FIP1L1	Factor Inte	Protein Co	38	GC04P053	8.63
1276	FRZB	Frizzled Re	Protein Co	41	GC02M182	8.63
1277	PPBP	Pro-Platele	Protein Co	43	GC04M073	8.62
1278	CDKN2B-A	CDKN2B A	RNA Gene	21	GC09P021	8.62
1279	CASP7	Caspase 7	Protein Co	50	GC10P113	8.61
1280	RPS27A	Ribosomal	Protein Co	43	GC02P055	8.61
1281	LGALS4	Galectin 4	Protein Co	38	GC19M042	8.6
1282	MIR195	MicroRNA	RNA Gene	19	GC17M007	8.59
1283	IKZF1	IKAROS Fa	Protein Co	47	GC07P050	8.59
1284	KLF4	Kruppel Lil	Protein Co	45	GC09M107	8.59
1285	AKT3	AKT Serine	Protein Co	52	GC01M243	8.59
1286	TRH	Thyrotropi	Protein Co	42	GC03P129	8.58
1287	PDE4A	Phosphodi	Protein Co	44	GC19P010	8.58
1288	PCSK9	Proprotein	Protein Co	51	GC01P055	8.56

1289	PRRT2	Proline Ric	Protein Co	39	GC16P029	8.56
1290	PCNA	Proliferatin	Protein Co	51	GC20M005	8.56
1291	RECK	Reversion	Protein Co	40	GC09P036	8.55
1292	XBP1	X-Box Binc	Protein Co	45	GC22M028	8.55
1293	STN1	STN1 Subu	Protein Co	32	GC10M103	8.54
1294	SLC22A5	Solute Car	Protein Co	46	GC05P132	8.53
1295	MT-ND3	Mitochond	Protein Co	31	GCMTP010	8.52
1296	GLA	Galactosid	Protein Co	48	GC0XM101	8.51
1297	MRAP	Melanocor	Protein Co	37	GC21P032	8.5
1298	CXCR1	C-X-C Mot	Protein Co	43	GC02M218	8.5
1299	HDAC9	Histone De	Protein Co	46	GC07P018	8.5
1300	S100A1	S100 Calci	Protein Co	41	GC01P153	8.5
1301	MIR20A	MicroRNA	RNA Gene	19	GC13P091	8.49
1302	GRHPR	Glyoxylate	Protein Co	46	GC09P037	8.49
1303	RBM8A	RNA Bindi	Protein Co	40	GC01M145	8.49
1304	SNRPB	Small Nucl	Protein Co	44	GC20M002	8.46
1305	DNAH11	Dynein Axo	Protein Co	41	GC07P021	8.46
1306	TMPRSS15	Transmem	Protein Co	41	GC21M018	8.46
1307	PRKG2	Protein Kir	Protein Co	45	GC04M081	8.45
1308	PRSS8	Serine Pro	Protein Co	43	GC16M031	8.45
1309	SATB2	SATB Hom	Protein Co	44	GC02M199	8.45
1310	MUC4	Mucin 4, C	Protein Co	38	GC03M195	8.44
1311	VDAC1	Voltage De	Protein Co	45	GC05M133	8.44
1312	LEMD3	LEM Doma	Protein Co	41	GC12P065	8.44
1313	HDAC1	Histone De	Protein Co	49	GC01P032	8.41
1314	CRYAA	Crystallin A	Protein Co	44	GC21P043	8.4
1315	TPMT	Thiopurine	Protein Co	47	GC06M018	8.39
1316	NR1I3	Nuclear Re	Protein Co	44	GC01M161	8.38
1317	C5AR1	Compleme	Protein Co	43	GC19P047	8.38
1318	KMT2E	Lysine Met	Protein Co	39	GC07P104	8.38
1319	EPAS1	Endothelia	Protein Co	48	GC02P046	8.37
1320	SLC18A3	Solute Car	Protein Co	43	GC10P049	8.37
1321	PARN	Poly(A)-Sp	Protein Co	45	GC16M014	8.36
1322	TREM1	Triggering	Protein Co	41	GC06M041	8.36
1323	MIR149	MicroRNA	RNA Gene	21	GC02P240	8.35
1324	DST	Dystonin	Protein Co	41	GC06M056	8.35
1325	HCCS	Holocytocl	Protein Co	41	GC0XP011	8.34
1326	NF2	Neurofibrc	Protein Co	48	GC22P029	8.34
1327	PNKP	Polynuclec	Protein Co	45	GC19M049	8.33
1328	CNTNAP1	Contactin /	Protein Co	42	GC17P042	8.33
1329	EIF4EBP1	Eukaryotic	Protein Co	47	GC08P038	8.33
1330	PYGM	Glycogen I	Protein Co	46	GC11M064	8.33
1331	P4HB	Prolyl 4-Hy	Protein Co	49	GC17M081	8.32

1332	CD69	CD69 Mole	Protein Co	40	GC12M013	8.32
1333	NT5E	5'-Nucleot	Protein Co	51	GC06P085	8.31
1334	RETREG1	Reticuloph	Protein Co	31	GC05M016	8.31
1335	CRHR1	Corticotrop	Protein Co	45	GC17P045	8.3
1336	RAD51	RAD51 Re	Protein Co	52	GC15P040	8.29
1337	PKD2	Polycystin	Protein Co	46	GC04P088	8.29
1338	NAMPT	Nicotinami	Protein Co	48	GC07M106	8.29
1339	PDE5A	Phosphodi	Protein Co	44	GC04M119	8.28
1340	LACTB	Lactamase	Protein Co	36	GC15P073	8.28
1341	CCR3	C-C Motif	Protein Co	47	GC03P046	8.27
1342	MIR200B	MicroRNA	RNA Gene	20	GC01P001	8.26
1343	CCN4	Cellular Co	Protein Co	31	GC08P133	8.26
1344	DYNC1H1	Dynein Cyt	Protein Co	44	GC14P104	8.26
1345	MIR29C	MicroRNA	RNA Gene	18	GC01M207	8.26
1346	ADORA2B	Adenosine	Protein Co	47	GC17P015	8.26
1347	NPHP1	Nephrocys	Protein Co	43	GC02M110	8.26
1348	SYT1	Synaptotag	Protein Co	46	GC12P078	8.25
1349	WNT3A	Wnt Family	Protein Co	47	GC01P228	8.24
1350	NOG	Noggin	Protein Co	47	GC17P056	8.24
1351	PLCG1	Phospholip	Protein Co	47	GC20P041	8.24
1352	XRCC6	X-Ray Rep	Protein Co	45	GC22P041	8.23
1353	LY96	Lymphocyt	Protein Co	42	GC08P073	8.23
1354	TUG1	Taurine Up	RNA Gene	22	GC22P030	8.21
1355	PRKD1	Protein Kir	Protein Co	50	GC14M029	8.21
1356	FOXG1	Forkhead B	Protein Co	43	GC14P028	8.2
1357	TBCE	Tubulin Fo	Protein Co	39	GC01P235	8.2
1358	COL17A1	Collagen T	Protein Co	43	GC10M104	8.2
1359	GAD2	Glutamate	Protein Co	46	GC10P026	8.2
1360	MIR338	MicroRNA	RNA Gene	17	GC17M081	8.19
1361	SLC19A3	Solute Car	Protein Co	46	GC02M227	8.19
1362	UMPS	Uridine Mo	Protein Co	45	GC03P124	8.19
1363	MAPK9	Mitogen-A	Protein Co	48	GC05M180	8.19
1364	IFI27	Interferon	Protein Co	37	GC14P094	8.18
1365	BSCL2	BSCL2 Lipi	Protein Co	42	GC11M063	8.16
1366	TOP2A	DNA Topo	Protein Co	50	GC17M040	8.14
1367	MAP3K7	Mitogen-A	Protein Co	51	GC06M090	8.13
1368	SREBF2	Sterol Reg	Protein Co	43	GC22P041	8.1
1369	IL4R	Interleukin	Protein Co	47	GC16P027	8.1
1370	IL17F	Interleukin	Protein Co	41	GC06M052	8.09
1371	EFTUD2	Elongation	Protein Co	42	GC17M044	8.09
1372	PEPD	Peptidase	Protein Co	45	GC19M033	8.09
1373	NDUFS3	NADH:Ubi	Protein Co	46	GC11P047	8.09
1374	CHKA	Choline Ki	Protein Co	41	GC11M068	8.08

1375	NME1	NME/NM2	Protein Co	47	GC17P051	8.08
1376	KCNJ11	Potassium	Protein Co	47	GC11M017	8.08
1377	GAA	Glucosidas	Protein Co	47	GC17P080	8.08
1378	PRKN	Parkin RBR	Protein Co	40	GC06M161	8.07
1379	DVL1	Dishevelle	Protein Co	47	GC01M001	8.07
1380	SHC1	SHC Adapt	Protein Co	45	GC01M154	8.06
1381	SLC10A1	Solute Car	Protein Co	43	GC14M069	8.06
1382	COX4I2	Cytochrom	Protein Co	41	GC20P031	8.05
1383	GBA	Glucosylce	Protein Co	47	GC01M155	8.05
1384	ZEB1	Zinc Finge	Protein Co	48	GC10P031	8.05
1385	ERF	ETS2 Repr	Protein Co	41	GC19M042	8.05
1386	TSHR	Thyroid Sti	Protein Co	47	GC14P080	8.05
1387	GATA2	GATA Binc	Protein Co	47	GC03M128	8.04
1388	CCDC103	Coiled-Coi	Protein Co	36	GC17P044	8.04
1389	PROCR	Protein C I	Protein Co	42	GC20P035	8.04
1390	NGLY1	N-Glycana	Protein Co	44	GC03M025	8.03
1391	TPH1	Tryptopha	Protein Co	44	GC11M018	8.02
1392	CACNA1B	Calcium V	Protein Co	48	GC09P137	8.02
1393	GP1BA	Glycoprote	Protein Co	45	GC17P004	8.02
1394	ALDH1A2	Aldehyde I	Protein Co	47	GC15M060	8.02
1395	FLT3	Fms Relate	Protein Co	52	GC13M028	8.01
1396	MUC16	Mucin 16,	Protein Co	36	GC19M008	8.01
1397	PNLIP	Pancreatic	Protein Co	47	GC10P116	8
1398	CCL25	C-C Motif	Protein Co	39	GC19P008	8
1399	TP73	Tumor Pro	Protein Co	45	GC01P003	8
1400	CYP2B6	Cytochrom	Protein Co	47	GC19P040	8
1401	CETP	Cholestery	Protein Co	46	GC16P056	7.99
1402	TEF	TEF Transc	Protein Co	38	GC22P041	7.99
1403	IKBKG	Inhibitor C	Protein Co	48	GC0XP154	7.99
1404	XRCC5	X-Ray Rep	Protein Co	44	GC02P216	7.98
1405	MIRLET7D	MicroRNA	RNA Gene	21	GC09P094	7.98
1406	SSTR2	Somatosta	Protein Co	47	GC17P073	7.98
1407	PIK3R3	Phosphoin	Protein Co	43	GC01M046	7.98
1408	DDX11	DEAD/H-B	Protein Co	43	GC12P031	7.98
1409	VTN	Vitronectin	Protein Co	44	GC17M029	7.98
1410	CFHR3	Compleme	Protein Co	39	GC01P196	7.97
1411	H4-16	H4 Histone	Protein Co	33	GC12M014	7.97
1412	HPS6	HPS6 Biog	Protein Co	39	GC10P102	7.96
1413	TRPM7	Transient F	Protein Co	45	GC15M050	7.96
1414	HMOX2	Heme Oxy	Protein Co	47	GC16P004	7.96
1415	RAC2	Rac Family	Protein Co	51	GC22M037	7.95
1416	PLA2G7	Phospholip	Protein Co	50	GC06M046	7.94
1417	ERCC1	ERCC Excis	Protein Co	45	GC19M045	7.94

1418	FUS	FUS RNA E	Protein Co	44	GC16P031	7.94
1419	MMP11	Matrix Me	Protein Co	45	GC22P023	7.94
1420	EIF2B2	Eukaryotic	Protein Co	44	GC14P075	7.94
1421	GC	GC Vitamir	Protein Co	43	GC04M071	7.94
1422	TOP1	DNA Topo	Protein Co	48	GC20P041	7.93
1423	PHOX2A	Paired Like	Protein Co	41	GC11M072	7.93
1424	GALC	Galactosyl	Protein Co	43	GC14M087	7.92
1425	SLC22A12	Solute Car	Protein Co	44	GC11P064	7.92
1426	AMBP	Alpha-1-M	Protein Co	41	GC09M114	7.92
1427	MTHFD1	Methylene	Protein Co	44	GC14P064	7.91
1428	PGR	Progesterc	Protein Co	50	GC11M100	7.9
1429	CELA3B	Chymotryp	Protein Co	37	GC01P021	7.9
1430	CACNA1G	Calcium Vo	Protein Co	48	GC17P050	7.89
1431	CCNA2	Cyclin A2	Protein Co	44	GC04M121	7.89
1432	TINF2	TERF1 Inte	Protein Co	41	GC14M024	7.88
1433	ERCC4	ERCC Excis	Protein Co	45	GC16P013	7.88
1434	REG4	Regenerati	Protein Co	40	GC01M119	7.88
1435	CFP	Compleme	Protein Co	43	GC0XM047	7.86
1436	SLC1A4	Solute Car	Protein Co	44	GC02P064	7.85
1437	CCR7	C-C Motif	Protein Co	45	GC17M040	7.85
1438	P2RY12	Purinergic	Protein Co	48	GC03M151	7.84
1439	SMARCC2	SWI/SNF R	Protein Co	44	GC12M056	7.83
1440	CLCN5	Chloride V	Protein Co	42	GC0XP049	7.83
1441	ACAN	Aggrecan	Protein Co	45	GC15P088	7.83
1442	PIGP	Phosphatic	Protein Co	38	GC21M037	7.82
1443	TRIP13	Thyroid Ho	Protein Co	40	GC05P000	7.82
1444	CAMSAP2	Calmodulir	Protein Co	33	GC01P200	7.82
1445	RGS6	Regulator	Protein Co	40	GC14P071	7.81
1446	EYA1	EYA Transc	Protein Co	45	GC08M071	7.81
1447	CYP7A1	Cytochrom	Protein Co	42	GC08M058	7.8
1448	SREBF1	Sterol Reg	Protein Co	44	GC17M017	7.79
1449	ZAP70	Zeta Chain	Protein Co	51	GC02P097	7.78
1450	IL37	Interleukin	Protein Co	37	GC02P115	7.78
1451	ARSA	Arylsulfata	Protein Co	47	GC22M050	7.78
1452	SLC30A10	Solute Car	Protein Co	42	GC01M219	7.77
1453	RFX5	Regulatory	Protein Co	41	GC01M151	7.77
1454	MAP1B	Microtubul	Protein Co	43	GC05P072	7.77
1455	SPARC	Secreted P	Protein Co	50	GC05M151	7.77
1456	CEL	Carboxyl E	Protein Co	46	GC09P133	7.77
1457	SPTLC1	Serine Palr	Protein Co	47	GC09M092	7.76
1458	REG3A	Regenerati	Protein Co	39	GC02M079	7.76
1459	CDH5	Cadherin 5	Protein Co	47	GC16P066	7.76
1460	PLA2G1B	Phospholip	Protein Co	46	GC12M120	7.76

1461	PLK1	Polo Like K	Protein Co	49	GC16P023	7.75
1462	CLDN4	Claudin 4	Protein Co	41	GC07P073	7.75
1463	CCR2	C-C Motif	Protein Co	45	GC03P046	7.74
1464	CFHR1	Compleme	Protein Co	41	GC01P196	7.74
1465	ADNP	Activity De	Protein Co	41	GC20M050	7.74
1466	LCAT	Lecithin-Cl	Protein Co	47	GC16M067	7.74
1467	SLC11A2	Solute Car	Protein Co	47	GC12M050	7.72
1468	CD68	CD68 Mol	Protein Co	40	GC17P007	7.71
1469	SCN8A	Sodium Vo	Protein Co	47	GC12P051	7.71
1470	AVPR2	Arginine V	Protein Co	47	GC0XP153	7.71
1471	CHGB	Chromogra	Protein Co	39	GC20P005	7.7
1472	CYP2C8	Cytochrom	Protein Co	48	GC10M095	7.69
1473	AGA	Aspartylglu	Protein Co	45	GC04M177	7.69
1474	PAGR1	PAXIP1 As	Protein Co	33	GC16P030	7.67
1475	SLC20A2	Solute Car	Protein Co	46	GC08M042	7.66
1476	MIRLET7B	MicroRNA	RNA Gene	20	GC22P046	7.66
1477	SEC24C	SEC24 Hor	Protein Co	44	GC10P073	7.64
1478	DCTN1	Dynactin S	Protein Co	46	GC02M074	7.64
1479	AFAP1-AS1	AFAP1 Ant	RNA Gene	17	GC04P007	7.64
1480	MIR141	MicroRNA	RNA Gene	21	GC12P008	7.63
1481	CES2	Carboxyles	Protein Co	43	GC16P066	7.63
1482	ITGAV	Integrin Su	Protein Co	46	GC02P186	7.63
1483	HPGD	15-Hydrox	Protein Co	48	GC04M174	7.62
1484	EDNRA	Endothelin	Protein Co	49	GC04P147	7.62
1485	RFXANK	Regulatory	Protein Co	42	GC19P019	7.62
1486	RNU4ATAC	RNA, U4at	RNA Gene	20	GC02P121	7.61
1487	GNRH1	Gonadotro	Protein Co	41	GC08M025	7.61
1488	IL6ST	Interleukin	Protein Co	45	GC05M055	7.61
1489	GRN	Granulin P	Protein Co	47	GC17P044	7.61
1490	CCNO	Cyclin O	Protein Co	41	GC05M055	7.61
1491	CSK	C-Terminal	Protein Co	48	GC15P074	7.61
1492	APOC3	Apolipopro	Protein Co	43	GC11P116	7.6
1493	NRP1	Neuropilin	Protein Co	47	GC10M033	7.6
1494	LRP1	LDL Recep	Protein Co	47	GC12P057	7.59
1495	PKHD1	PKHD1 Cili	Protein Co	39	GC06M051	7.59
1496	KRT19	Keratin 19	Protein Co	45	GC17M041	7.59
1497	GPBAR1	G Protein-(	Protein Co	39	GC02P218	7.59
1498	ADAM10	ADAM Me	Protein Co	52	GC15M058	7.58
1499	CXCL13	C-X-C Mot	Protein Co	41	GC04P077	7.58
1500	ADSL	Adenylosu	Protein Co	47	GC22P040	7.58
1501	MUC3A	Mucin 3A,	Protein Co	34	GC07P100	7.58
1502	SIX1	SIX Homeo	Protein Co	44	GC14M060	7.58
1503	ACVR2A	Activin A F	Protein Co	45	GC02P147	7.57

1504	OXT	Oxytocin/N	Protein Co	40	GC20P003	7.56
1505	AHI1	Abelson H	Protein Co	41	GC06M135	7.56
1506	POLA1	DNA Polyr	Protein Co	46	GC0XP024	7.55
1507	MIR27B	MicroRNA	RNA Gene	21	GC09P095	7.55
1508	AHDC1	AT-Hook D	Protein Co	36	GC01M027	7.55
1509	ADAMTSL1	ADAMTS L	Protein Co	41	GC09P017	7.54
1510	SLIT2	Slit Guidar	Protein Co	45	GC04P020	7.54
1511	ATP7B	ATPase Co	Protein Co	47	GC13M051	7.54
1512	ITGA8	Integrin Su	Protein Co	42	GC10M015	7.53
1513	VIL1	Villin 1	Protein Co	39	GC02P218	7.53
1514	MIR23A	MicroRNA	RNA Gene	20	GC19M014	7.53
1515	PVT1	Pvt1 Onco	RNA Gene	25	GC08P127	7.52
1516	BAK1	BCL2 Anta	Protein Co	44	GC06M033	7.52
1517	MLXIPL	MLX Intera	Protein Co	41	GC07M073	7.51
1518	WIPF1	WAS/WAS	Protein Co	44	GC02M174	7.51
1519	IRF8	Interferon	Protein Co	45	GC16P085	7.51
1520	SLC29A1	Solute Car	Protein Co	48	GC06P044	7.51
1521	RTN4	Reticulon 4	Protein Co	44	GC02M054	7.51
1522	EPHA2	EPH Recep	Protein Co	52	GC01M016	7.5
1523	FDFT1	Farnesyl-D	Protein Co	44	GC08P011	7.49
1524	PMPCA	Peptidase,	Protein Co	43	GC09P136	7.49
1525	MAP2K7	Mitogen-A	Protein Co	45	GC19P007	7.48
1526	PTGDS	Prostaglan	Protein Co	45	GC09P136	7.48
1527	VPS13A	Vacuolar P	Protein Co	40	GC09P077	7.48
1528	RASGRP1	RAS Guany	Protein Co	46	GC15M038	7.48
1529	NDUFB9	NADH:Ubi	Protein Co	44	GC08P124	7.47
1530	CA9	Carbonic A	Protein Co	44	GC09P035	7.47
1531	TMSB4X	Thymosin	Protein Co	40	GC0XP012	7.45
1532	PCSK1	Proprotein	Protein Co	45	GC05M096	7.45
1533	ASS1	Argininosu	Protein Co	48	GC09P130	7.45
1534	TPI1	Triosephos	Protein Co	48	GC12P008	7.45
1535	TNFRSF13	TNF Recep	Protein Co	44	GC22M045	7.44
1536	BDKRB2	Bradykinin	Protein Co	44	GC14P096	7.43
1537	LIF	LIF Interleu	Protein Co	43	GC22M030	7.43
1538	RXRA	Retinoid X	Protein Co	49	GC09P134	7.43
1539	MT-ND2	Mitochond	Protein Co	32	GCMTP004	7.43
1540	PTPRN	Protein Ty	Protein Co	44	GC02M219	7.42
1541	UBE3A	Ubiquitin F	Protein Co	47	GC15M025	7.42
1542	ESS2	Ess-2 Splic	Protein Co	28	GC22M019	7.42
1543	MIR222	MicroRNA	RNA Gene	21	GC0XM045	7.42
1544	RSPH4A	Radial Spo	Protein Co	38	GC06P116	7.42
1545	GNAO1	G Protein S	Protein Co	47	GC16P056	7.41
1546	SLC30A2	Solute Car	Protein Co	43	GC01M026	7.41

1547	PPP2R1A	Protein Ph	Protein Co	47	GC19P052	7.41
1548	VPS11	VPS11 Cor	Protein Co	41	GC11P119	7.41
1549	TYK2	Tyrosine K	Protein Co	52	GC19M010	7.39
1550	TNFRSF11A	TNF Recep	Protein Co	45	GC18P062	7.39
1551	IL1RL1	Interleukin	Protein Co	41	GC02P102	7.39
1552	SLC8A1	Solute Car	Protein Co	44	GC02M040	7.38
1553	NPM1	Nucleophc	Protein Co	50	GC05P171	7.38
1554	CEP104	Centrosom	Protein Co	36	GC01M003	7.38
1555	CYP21A2	Cytochrom	Protein Co	45	GC06P047	7.36
1556	IFNGR1	Interferon	Protein Co	50	GC06M137	7.36
1557	PI3	Peptidase	Protein Co	39	GC20P045	7.36
1558	FUT4	Fucosyltrar	Protein Co	36	GC11P094	7.36
1559	NDUFAF3	NADH:Ubi	Protein Co	40	GC03P049	7.34
1560	ACP5	Acid Phosp	Protein Co	47	GC19M011	7.34
1561	CD7	CD7 Molec	Protein Co	39	GC17M082	7.34
1562	SP140	SP140 Nuc	Protein Co	37	GC02P230	7.34
1563	RPS19	Ribosomal	Protein Co	48	GC19P041	7.33
1564	SERPINA4	Serpin Fan	Protein Co	41	GC14P094	7.33
1565	KRT1	Keratin 1	Protein Co	44	GC12M052	7.32
1566	CD209	CD209 Mo	Protein Co	43	GC19M007	7.32
1567	WNK1	WNK Lysin	Protein Co	46	GC12P000	7.32
1568	SMAD9	SMAD Fan	Protein Co	45	GC13M036	7.32
1569	HPSE	Heparanas	Protein Co	44	GC04M083	7.32
1570	IFT172	Intraflagell	Protein Co	39	GC02M027	7.31
1571	COL5A2	Collagen T	Protein Co	41	GC02M189	7.31
1572	RIPK2	Receptor I	Protein Co	46	GC08P089	7.3
1573	CHRM3	Cholinergi	Protein Co	48	GC01P239	7.3
1574	PIK3R2	Phosphoin	Protein Co	49	GC19P018	7.29
1575	APAF1	Apoptotic	Protein Co	47	GC12P098	7.28
1576	PRICKLE2	Prickle Pla	Protein Co	39	GC03M064	7.27
1577	PIK3CB	Phosphatic	Protein Co	47	GC03M138	7.27
1578	RETNLB	Resistin Lik	Protein Co	35	GC03M108	7.27
1579	KLRD1	Killer Cell I	Protein Co	41	GC12P010	7.26
1580	MORC2	MORC Fan	Protein Co	40	GC22M030	7.26
1581	HLA-E	Major Hist	Protein Co	41	GC06P047	7.26
1582	NEFH	Neurofilam	Protein Co	45	GC22P029	7.25
1583	UMOD	Uromoduli	Protein Co	41	GC16M020	7.25
1584	TAGAP	T Cell Acti	Protein Co	37	GC06M159	7.24
1585	GTF2I	General Tr	Protein Co	42	GC07P074	7.24
1586	DAXX	Death Don	Protein Co	44	GC06M033	7.24
1587	RHOD	Ras Homo	Protein Co	39	GC11P067	7.24
1588	MIR675	MicroRNA	RNA Gene	17	GC11M001	7.24
1589	JMJD1C	Jumonji D	Protein Co	40	GC10M063	7.24

1590	MIR99A	MicroRNA	RNA Gene	21	GC21P016	7.24
1591	SERPINB2	Serpin Fan	Protein Co	44	GC18P063	7.23
1592	MIR185	MicroRNA	RNA Gene	21	GC22P020	7.23
1593	PTPA	Protein Ph	Protein Co	35	GC09P129	7.23
1594	LRRC56	Leucine Ri	Protein Co	36	GC11P000	7.23
1595	CTBP1	C-Terminal	Protein Co	47	GC04M001	7.23
1596	RAB11A	RAB11A, M	Protein Co	46	GC15P072	7.23
1597	TOR1B	Torsin Fan	Protein Co	38	GC09P129	7.23
1598	ZIC3	Zic Family	Protein Co	46	GC0XP137	7.22
1599	SOAT1	Sterol O-A	Protein Co	44	GC01P179	7.22
1600	AFF2	AF4/FMR2	Protein Co	41	GC0XP148	7.22
1601	CLCN2	Chloride V	Protein Co	45	GC03M184	7.22
1602	SCN3A	Sodium Vo	Protein Co	47	GC02M165	7.21
1603	PIGR	Polymeric	Protein Co	41	GC01M206	7.21
1604	ESCO2	Establishm	Protein Co	39	GC08P027	7.21
1605	SOX2-OT	SOX2 Over	RNA Gene	21	GC03P180	7.2
1606	E2F2	E2F Transc	Protein Co	42	GC01M023	7.2
1607	NR0B2	Nuclear Re	Protein Co	43	GC01M026	7.19
1608	SLC9A6	Solute Car	Protein Co	46	GC0XP135	7.19
1609	MAG	Myelin Ass	Protein Co	45	GC19P035	7.19
1610	EFEMP1	EGF Conta	Protein Co	44	GC02M055	7.19
1611	LTA4H	Leukotrien	Protein Co	46	GC12M096	7.19
1612	SERPINB5	Serpin Fan	Protein Co	43	GC18P063	7.18
1613	ACTG1	Actin Gam	Protein Co	50	GC17M081	7.18
1614	ZBTB24	Zinc Finge	Protein Co	38	GC06M109	7.18
1615	KLK3	Kallikrein F	Protein Co	46	GC19P050	7.17
1616	PKM	Pyruvate K	Protein Co	45	GC15M072	7.17
1617	SETX	Senataxin	Protein Co	41	GC09M132	7.17
1618	ROS1	ROS Proto	Protein Co	45	GC06M117	7.17
1619	PVALB	Parvalbum	Protein Co	39	GC22M036	7.17
1620	MCIDAS	Multiciliate	Protein Co	29	GC05M055	7.16
1621	KLRC1	Killer Cell I	Protein Co	40	GC12M013	7.15
1622	PGF	Placental G	Protein Co	43	GC14M074	7.15
1623	POSTN	Periostin	Protein Co	43	GC13M037	7.15
1624	PCCB	Propionyl-(	Protein Co	45	GC03P136	7.15
1625	OSM	Oncostatin	Protein Co	43	GC22M030	7.15
1626	STIM1	Stromal In	Protein Co	48	GC11P003	7.14
1627	LYZ	Lysozyme	Protein Co	47	GC12P069	7.14
1628	ATF6	Activating	Protein Co	47	GC01P161	7.14
1629	CHEK1	Checkpoin	Protein Co	50	GC11P125	7.14
1630	EFNA1	Ephrin A1	Protein Co	43	GC01P155	7.13
1631	MIR139	MicroRNA	RNA Gene	19	GC11M072	7.13
1632	CSPG4	Chondroiti	Protein Co	43	GC15M075	7.12

1633	REG1A	Regenerati	Protein Co	41	GC02P079	7.12
1634	C9orf72	C9orf72-SI	Protein Co	41	GC09M027	7.12
1635	CFHR4	Compleme	Protein Co	35	GC01P196	7.11
1636	SERPINH1	Serpin Fan	Protein Co	45	GC11P075	7.11
1637	STXBP1	Syntaxin B	Protein Co	48	GC09P127	7.11
1638	MAP2K4	Mitogen-A	Protein Co	46	GC17P012	7.11
1639	MIR10B	MicroRNA	RNA Gene	21	GC02P176	7.11
1640	MT-ND5	Mitochonc	Protein Co	31	GCMTP012	7.11
1641	DEAF1	DEAF1 Tra	Protein Co	40	GC11M000	7.1
1642	BCL10	BCL10 Imn	Protein Co	45	GC01M085	7.1
1643	GPX7	Glutathion	Protein Co	43	GC01P052	7.1
1644	DPH1	Diphthami	Protein Co	40	GC17P002	7.1
1645	CNTF	Ciliary Neu	Protein Co	41	GC11P058	7.09
1646	CABIN1	Calcineurir	Protein Co	41	GC22P024	7.08
1647	TRPA1	Transient F	Protein Co	45	GC08M072	7.08
1648	DIABLO	Diablo IAP	Protein Co	47	GC12M122	7.07
1649	H2AX	H2A.X Vari	Protein Co	35	GC11M119	7.07
1650	REL	REL Proto-	Protein Co	46	GC02P060	7.07
1651	SH3KBP1	SH3 Doma	Protein Co	41	GC0XM019	7.06
1652	FANCD2	FA Comple	Protein Co	47	GC03P010	7.06
1653	CCAT1	Colon Can	RNA Gene	14	GC08M127	7.05
1654	TGM1	Transgluta	Protein Co	45	GC14M024	7.05
1655	CAVIN1	Caveolae A	Protein Co	32	GC17M042	7.04
1656	PLA2G2D	Phospholip	Protein Co	42	GC01M020	7.04
1657	IRS2	Insulin Rec	Protein Co	44	GC13M109	7.03
1658	DAG1	Dystroglyc	Protein Co	46	GC03P049	7.03
1659	CD163	CD163 Mo	Protein Co	42	GC12M007	7.02
1660	PRSS2	Serine Pro	Protein Co	39	GC07P144	7.01
1661	SNAI2	Snail Famil	Protein Co	44	GC08M048	7.01
1662	MIR130A	MicroRNA	RNA Gene	20	GC11P057	7.01
1663	TRAF2	TNF Recep	Protein Co	44	GC09P136	7
1664	FLI1	Fli-1 Proto	Protein Co	49	GC11P128	7
1665	HINT1	Histidine T	Protein Co	45	GC05M131	7
1666	LRP6	LDL Recep	Protein Co	48	GC12M013	7
1667	WNT8B	Wnt Family	Protein Co	41	GC10P100	6.99
1668	MIR324	MicroRNA	RNA Gene	18	GC17M007	6.99
1669	PLAGL1	PLAG1 Like	Protein Co	43	GC06M143	6.99
1670	HEPACAM	Hepatic Ar	Protein Co	40	GC11M124	6.99
1671	ABHD5	Abhydrola	Protein Co	45	GC03P043	6.98
1672	DDX58	DExD/H-Bo	Protein Co	47	GC09M032	6.98
1673	ERBB4	Erb-B2 Rec	Protein Co	55	GC02M211	6.97
1674	HPS1	HPS1 Biog	Protein Co	41	GC10M098	6.97
1675	SEPTIN9	Septin 9	Protein Co	35	GC17P077	6.97

1676	ADAMTS4	ADAM Me	Protein Co	42	GC01M161	6.97
1677	ERN1	Endoplasm	Protein Co	45	GC17M064	6.97
1678	UBASH3A	Ubiquitin A	Protein Co	39	GC21P042	6.96
1679	CCNE1	Cyclin E1	Protein Co	48	GC19P029	6.96
1680	PTGER4	Prostaglan	Protein Co	46	GC05P040	6.95
1681	SLC39A14	Solute Car	Protein Co	43	GC08P022	6.94
1682	DDC	Dopa Deca	Protein Co	52	GC07M050	6.94
1683	SLITRK1	SLIT And N	Protein Co	40	GC13M083	6.94
1684	ICAM3	Intercellula	Protein Co	41	GC19M010	6.94
1685	MIR455	MicroRNA	RNA Gene	18	GC09P114	6.93
1686	BIRC2	Baculoviral	Protein Co	45	GC11P102	6.93
1687	ELAVL1	ELAV Like	Protein Co	41	GC19M007	6.93
1688	RFXAP	Regulatory	Protein Co	37	GC13P036	6.93
1689	MCM4	Minichrom	Protein Co	47	GC08P047	6.93
1690	LETM1	Leucine Zip	Protein Co	41	GC04M001	6.93
1691	UCA1	Urothelial	RNA Gene	24	GC19P015	6.92
1692	MIR191	MicroRNA	RNA Gene	20	GC03M049	6.92
1693	ATG5	Autophagy	Protein Co	44	GC06M106	6.91
1694	PKLR	Pyruvate K	Protein Co	47	GC01M155	6.91
1695	ANKRD55	Ankyrin Re	Protein Co	33	GC05M056	6.9
1696	E2F4	E2F Transc	Protein Co	45	GC16P067	6.9
1697	FZD7	Frizzled Cl	Protein Co	44	GC02P202	6.9
1698	SETD2	SET Domai	Protein Co	47	GC03M047	6.88
1699	APOC2	Apolipopro	Protein Co	44	GC19P044	6.88
1700	CCND3	Cyclin D3	Protein Co	47	GC06M041	6.88
1701	IDS	Iduronate	Protein Co	48	GC0XM149	6.87
1702	TOLLIP	Toll Intera	Protein Co	44	GC11M001	6.87
1703	UBAC2	UBA Doma	Protein Co	37	GC13P099	6.87
1704	RASA2	RAS P21 P	Protein Co	40	GC03P141	6.87
1705	DRD1	Dopamine	Protein Co	44	GC05M175	6.86
1706	RUNX1	RUNX Fam	Protein Co	48	GC21M034	6.86
1707	WNT9A	Wnt Family	Protein Co	41	GC01M227	6.86
1708	LALBA	Lactalbumi	Protein Co	39	GC12M048	6.85
1709	KRT15	Keratin 15	Protein Co	39	GC17M041	6.85
1710	IL21R	Interleukin	Protein Co	41	GC16P027	6.85
1711	CTTN	Cortactin	Protein Co	43	GC11P070	6.85
1712	KAT5	Lysine Ace	Protein Co	46	GC11P065	6.84
1713	NSUN2	NOP2/Sun	Protein Co	42	GC05M006	6.84
1714	TCF7	Transcripti	Protein Co	44	GC05P134	6.84
1715	BAZ2B	Bromodom	Protein Co	39	GC02M159	6.84
1716	GGT2	Gamma-Gl	Protein Co	31	GC22M021	6.84
1717	CELA1	Chymotryp	Protein Co	36	GC12M051	6.83
1718	IGFBP1	Insulin Like	Protein Co	43	GC07P046	6.83

1719	LAMB1	Laminin Su	Protein Co	47	GC07M107	6.83
1720	LIMK1	LIM Doma	Protein Co	49	GC07P074	6.83
1721	CBS	Cystathion	Protein Co	48	GC21M043	6.82
1722	TNNI3	Troponin I	Protein Co	48	GC19M055	6.82
1723	PSMB8	Proteasom	Protein Co	49	GC06M032	6.82
1724	RPLP0	Ribosomal	Protein Co	41	GC12M120	6.81
1725	GPR55	G Protein-(	Protein Co	40	GC02M230	6.81
1726	DNM1	Dynamin 1	Protein Co	50	GC09P128	6.81
1727	HSPA1A	Heat Shoc	Protein Co	43	GC06P047	6.81
1728	SLC12A1	Solute Car	Protein Co	46	GC15P048	6.81
1729	SLC3A2	Solute Car	Protein Co	42	GC11P062	6.8
1730	NTN1	Netrin 1	Protein Co	44	GC17P009	6.8
1731	FURIN	Furin, Paire	Protein Co	46	GC15P090	6.8
1732	F2RL1	F2R Like T	Protein Co	45	GC05P076	6.79
1733	NCOA3	Nuclear Re	Protein Co	45	GC20P047	6.79
1734	CFHR2	Compleme	Protein Co	37	GC01P196	6.79
1735	TBK1	TANK Bind	Protein Co	49	GC12P064	6.78
1736	MLNR	Motilin Re	Protein Co	40	GC13P049	6.78
1737	MIR532	MicroRNA	RNA Gene	16	GC0XP050	6.77
1738	CLIP2	CAP-Gly D	Protein Co	37	GC07P074	6.77
1739	SERPINA7	Serpin Fan	Protein Co	39	GC0XM106	6.76
1740	KPTN	Kaptin, Act	Protein Co	37	GC19M047	6.75
1741	PTHLH	Parathyroi	Protein Co	45	GC12M027	6.75
1742	BMP1	Bone Morp	Protein Co	45	GC08P022	6.75
1743	LPA	Lipoprotei	Protein Co	41	GC06M160	6.74
1744	B3GALT6	Beta-1,3-G	Protein Co	39	GC01P001	6.74
1745	SLC4A4	Solute Car	Protein Co	46	GC04P071	6.74
1746	MTRR	5-Methylte	Protein Co	43	GC05P007	6.73
1747	CTRC	Chymotryp	Protein Co	44	GC01P015	6.73
1748	PDYN	Prodynorp	Protein Co	43	GC20M001	6.73
1749	FXN	Frataxin	Protein Co	46	GC09P069	6.72
1750	PAPPA	Pappalysin	Protein Co	41	GC09P116	6.72
1751	MIR132	MicroRNA	RNA Gene	21	GC17M002	6.71
1752	CNTNAP2	Contactin /	Protein Co	44	GC07P146	6.71
1753	CSNK1A1	Casein Kin	Protein Co	49	GC05M149	6.7
1754	CANX	Calnexin	Protein Co	44	GC05P179	6.7
1755	DISP1	Dispatchec	Protein Co	37	GC01P222	6.7
1756	MIR181A1	MicroRNA	RNA Gene	18	GC01M198	6.7
1757	SOX11	SRY-Box T	Protein Co	41	GC02P005	6.69
1758	COG2	Componer	Protein Co	40	GC01P230	6.69
1759	EIF4G1	Eukaryotic	Protein Co	45	GC03P184	6.69
1760	CARTPT	CART Prep	Protein Co	44	GC05P071	6.68
1761	APPL1	Adaptor P	Protein Co	45	GC03P057	6.68

1762	CYP7B1	Cytochrom	Protein Co	45	GC08M064	6.67
1763	SPRY4-IT1	SPRY4 Intr	RNA Gene	14	GC05M142	6.67
1764	TNFSF13	TNF Super	Protein Co	44	GC17P007	6.67
1765	FOXE3	Forkhead I	Protein Co	35	GC01P047	6.67
1766	KIAA0753	KIAA0753	Protein Co	35	GC17M006	6.65
1767	H3-2	H3.2 Histo	Protein Co	17	GC01M143	6.65
1768	SLC25A4	Solute Car	Protein Co	48	GC04P185	6.65
1769	ARVCF	ARVCF Del	Protein Co	38	GC22M019	6.65
1770	SIGLEC5	Sialic Acid	Protein Co	37	GC19M051	6.64
1771	IREB2	Iron Respo	Protein Co	43	GC15P078	6.64
1772	FARSB	Phenylalan	Protein Co	44	GC02M222	6.64
1773	YAP1	Yes1 Asso	Protein Co	47	GC11P102	6.64
1774	DSG1	Desmoglei	Protein Co	43	GC18P031	6.63
1775	TNFSF12	TNF Super	Protein Co	40	GC17P008	6.63
1776	IRF4	Interferon	Protein Co	43	GC06P000	6.63
1777	DGAT1	Diacylglyce	Protein Co	47	GC08M144	6.63
1778	CLDN5	Claudin 5	Protein Co	41	GC22M019	6.63
1779	UBC	Ubiquitin C	Protein Co	43	GC12M124	6.62
1780	EDN2	Endothelin	Protein Co	40	GC01M041	6.62
1781	ACER3	Alkaline Ce	Protein Co	38	GC11P076	6.62
1782	YARS1	Tyrosyl-TR	Protein Co	36	GC01M032	6.62
1783	ADORA2A	Adenosine	Protein Co	45	GC22P024	6.62
1784	WNT10B	Wnt Family	Protein Co	48	GC12M048	6.62
1785	BBC3	BCL2 Bindi	Protein Co	41	GC19M047	6.62
1786	PCAT1	Prostate C	RNA Gene	17	GC08P126	6.61
1787	SERPINB1	Serpin Fam	Protein Co	40	GC06M002	6.61
1788	CAPN5	Calpain 5	Protein Co	43	GC11P077	6.61
1789	ECM1	Extracellula	Protein Co	43	GC01P150	6.61
1790	CARD9	Caspase Re	Protein Co	44	GC09M136	6.61
1791	BACE1	Beta-Secre	Protein Co	47	GC11M117	6.61
1792	ICAM2	Intercellula	Protein Co	45	GC17M064	6.61
1793	DKC1	Dyskerin P	Protein Co	46	GC0XP154	6.61
1794	TJP2	Tight Junct	Protein Co	45	GC09P069	6.6
1795	WRAP53	WD Repea	Protein Co	41	GC17P008	6.6
1796	MIR135A1	MicroRNA	RNA Gene	20	GC03M052	6.6
1797	APTX	Aprataxin	Protein Co	44	GC09M032	6.6
1798	AKR1A1	Aldo-Keto	Protein Co	44	GC01P045	6.6
1799	FAH	Fumarylace	Protein Co	47	GC15P080	6.6
1800	TAF6	TATA-Box	Protein Co	40	GC07M100	6.6
1801	RGS1	Regulator	Protein Co	38	GC01P192	6.6
1802	CACNA1C	Calcium Vo	Protein Co	48	GC12P001	6.59
1803	ACP1	Acid Phosp	Protein Co	44	GC02P000	6.59
1804	CCL7	C-C Motif	Protein Co	41	GC17P034	6.58

1805	MIR148A	MicroRNA	RNA Gene	18	GC07M025	6.58
1806	HULC	Hepatocell	RNA Gene	21	GC06P008	6.56
1807	OPRD1	Opioid Rec	Protein Co	44	GC01P028	6.56
1808	OPLAH	5-Oxoproli	Protein Co	41	GC08M144	6.56
1809	SRD5A3	Steroid 5 A	Protein Co	43	GC04P055	6.55
1810	MYB	MYB Proto	Protein Co	50	GC06P135	6.55
1811	ARF6	ADP Ribos	Protein Co	44	GC14P049	6.55
1812	KCNE1	Potassium	Protein Co	44	GC21M034	6.55
1813	TNFRSF9	TNF Recep	Protein Co	44	GC01M007	6.55
1814	PYCARD	PYD And C	Protein Co	43	GC16M031	6.54
1815	MTDH	Metadherii	Protein Co	40	GC08P097	6.54
1816	CLEC7A	C-Type Lec	Protein Co	44	GC12M013	6.53
1817	WNT6	Wnt Family	Protein Co	41	GC02P218	6.52
1818	FGF1	Fibroblast	Protein Co	48	GC05M142	6.52
1819	AREG	Amphiregu	Protein Co	43	GC04P074	6.52
1820	CALB1	Calbindin	Protein Co	41	GC08M090	6.52
1821	STH	Saitohin	Protein Co	30	GC17P045	6.52
1822	TGM6	Transgluta	Protein Co	39	GC20P002	6.52
1823	CD27	CD27 Mole	Protein Co	45	GC12P008	6.5
1824	MECOM	MDS1 And	Protein Co	47	GC03M169	6.5
1825	PPY	Pancreatic	Protein Co	40	GC17M043	6.5
1826	MIR98	MicroRNA	RNA Gene	17	GC0XM053	6.5
1827	MEF2C	Myocyte E	Protein Co	48	GC05M088	6.49
1828	RBP1	Retinol Bin	Protein Co	39	GC03M139	6.48
1829	MRE11	MRE11 Ho	Protein Co	41	GC11M094	6.48
1830	NAT9	N-Acetyltra	Protein Co	37	GC17M074	6.48
1831	NABP1	Nucleic Ac	Protein Co	35	GC02P191	6.48
1832	FCN2	Ficolin 2	Protein Co	40	GC09P134	6.47
1833	SLC51B	Solute Car	Protein Co	33	GC15P072	6.47
1834	WNT11	Wnt Family	Protein Co	45	GC11M076	6.47
1835	FANCC	FA Comple	Protein Co	47	GC09M095	6.46
1836	LITAF	Lipopolysa	Protein Co	44	GC16M011	6.46
1837	DGUOK	Deoxyguar	Protein Co	44	GC02P073	6.46
1838	SLC37A4	Solute Car	Protein Co	43	GC11M119	6.46
1839	ADM	Adrenome	Protein Co	44	GC11P010	6.45
1840	NDUFB11	NADH:Ubi	Protein Co	39	GC0XM047	6.45
1841	SHMT1	Serine Hyc	Protein Co	45	GC17M019	6.45
1842	ARPC1B	Actin Relat	Protein Co	43	GC07P099	6.45
1843	THBS2	Thrombosp	Protein Co	45	GC06M169	6.45
1844	MUC3B	Mucin 3B,	Protein Co	17	GC07U903	6.44
1845	MMP14	Matrix Met	Protein Co	51	GC14P025	6.44
1846	FOSL1	FOS Like 1	Protein Co	44	GC11M065	6.44
1847	GGTLC3	Gamma-Gl	Protein Co	20	GC22M018	6.43

1848	NALCN	Sodium Le	Protein Co	40	GC13M101	6.43
1849	GHR	Growth Hc	Protein Co	47	GC05P042	6.42
1850	MALT1	MALT1 Par	Protein Co	47	GC18P058	6.42
1851	TCF7L1	Transcripti	Protein Co	41	GC02P085	6.42
1852	CD3E	CD3e Mole	Protein Co	47	GC11P118	6.42
1853	CYP3A5	Cytochrom	Protein Co	47	GC07M099	6.41
1854	SLC9A3R2	SLC9A3 Re	Protein Co	39	GC16P002	6.41
1855	RSPO1	R-Spondin	Protein Co	44	GC01M037	6.41
1856	MAGI2	Membrane	Protein Co	43	GC07M078	6.4
1857	ITLN1	Intelectin 1	Protein Co	39	GC01M160	6.4
1858	CTSK	Cathepsin	Protein Co	48	GC01M150	6.39
1859	FTL	Ferritin Lig	Protein Co	47	GC19P048	6.38
1860	CD5	CD5 Molec	Protein Co	41	GC11P061	6.38
1861	CTNND1	Catenin De	Protein Co	45	GC11P057	6.38
1862	CHSY1	Chondroiti	Protein Co	43	GC15M101	6.37
1863	NTHL1	Nth Like D	Protein Co	44	GC16M002	6.36
1864	NOP10	NOP10 Rib	Protein Co	40	GC15M034	6.36
1865	PENK	Proenkeph	Protein Co	38	GC08M056	6.36
1866	MIRLET7C	MicroRNA	RNA Gene	21	GC21P016	6.36
1867	TIAM1	TIAM Rac1	Protein Co	44	GC21M031	6.35
1868	ADRB1	Adrenocep	Protein Co	48	GC10P114	6.35
1869	TRIM21	Tripartite M	Protein Co	42	GC11M004	6.35
1870	THY1	Thy-1 Cell	Protein Co	44	GC11M119	6.35
1871	ATAD2	ATPase Fa	Protein Co	39	GC08M123	6.35
1872	SSTR1	Somatosta	Protein Co	43	GC14P038	6.34
1873	H3-3A	H3.3 Histo	Protein Co	34	GC01P226	6.34
1874	MIRLET7E	MicroRNA	RNA Gene	20	GC19P051	6.34
1875	ZFP36L1	ZFP36 Ring	Protein Co	43	GC14M068	6.33
1876	ICOS	Inducible T	Protein Co	43	GC02P203	6.33
1877	RHOBTB2	Rho Relate	Protein Co	40	GC08P022	6.33
1878	MUC17	Mucin 17,	Protein Co	35	GC07P101	6.33
1879	FAM53B	Family Wit	Protein Co	34	GC10M124	6.32
1880	BAZ1B	Bromodom	Protein Co	40	GC07M073	6.32
1881	IRF7	Interferon	Protein Co	46	GC11M000	6.31
1882	IGHMBP2	Immunogl	Protein Co	41	GC11P068	6.3
1883	CGA	Glycoprote	Protein Co	43	GC06M087	6.3
1884	PRKCB	Protein Kir	Protein Co	47	GC16P023	6.3
1885	SLC9A1	Solute Car	Protein Co	51	GC01M027	6.3
1886	IFNAR1	Interferon	Protein Co	45	GC21P033	6.29
1887	NLRP12	NLR Family	Protein Co	44	GC19M053	6.29
1888	NR2F1	Nuclear Re	Protein Co	45	GC05P093	6.29
1889	SLCO2A1	Solute Car	Protein Co	43	GC03M133	6.29
1890	MAF	MAF BZIP	Protein Co	45	GC16M079	6.28

1891	SLC30A8	Solute Car	Protein Co		42	GC08P116	6.28
1892	OPRK1	Opioid Rec	Protein Co		45	GC08M053	6.28
1893	PAEP	Progestage	Protein Co		39	GC09P135	6.28
1894	WASF1	WASP Fam	Protein Co		43	GC06M110	6.28
1895	CLEC16A	C-Type Lec	Protein Co		37	GC16P010	6.27
1896	SLC34A1	Solute Car	Protein Co		44	GC05P177	6.27
1897	EIF2AK3	Eukaryotic	Protein Co		48	GC02M088	6.27
1898	NHP2	NHP2 Ribo	Protein Co		44	GC05M178	6.27
1899	CRIP1	Cysteine R	Protein Co		36	GC14P105	6.27
1900	RFC2	Replicatior	Protein Co		44	GC07M074	6.26
1901	MSN	Moesin	Protein Co		47	GC0XP065	6.25
1902	AK2	Adenylate	Protein Co		48	GC01M033	6.25
1903	RAB8A	RAB8A, Me	Protein Co		40	GC19P016	6.25
1904	SNAP25	Synaptoso	Protein Co		49	GC20P010	6.25
1905	GALNS	Galactosan	Protein Co		44	GC16M088	6.24
1906	AGR2	Anterior G	Protein Co		40	GC07M016	6.24
1907	ADD1	Adducin 1	Protein Co		44	GC04P002	6.24
1908	ELP1	Elongator	Protein Co		33	GC09M108	6.24
1909	LYVE1	Lymphatic	Protein Co		41	GC11M010	6.23
1910	FGFRL1	Fibroblast	Protein Co		42	GC04P001	6.22
1911	SRCIN1	SRC Kinase	Protein Co		35	GC17M038	6.22
1912	FABP12	Fatty Acid	Protein Co		31	GC08M081	6.22
1913	DLG3	Discs Larg	Protein Co		41	GC0XP070	6.21
1914	ASPM	Assembly I	Protein Co		39	GC01M197	6.21
1915	FANCB	FA Comple	Protein Co		40	GC0XM014	6.2
1916	WNT7A	Wnt Family	Protein Co		49	GC03M015	6.2
1917	NDUFS8	NADH:Ubi	Protein Co		45	GC11P068	6.2
1918	SUOX	Sulfite Oxi	Protein Co		45	GC12P055	6.2
1919	PSCA	Prostate St	Protein Co		39	GC08P142	6.2
1920	ST3GAL3	ST3 Beta-C	Protein Co		45	GC01P043	6.2
1921	SEPSECS	Sep (O-Ph	Protein Co		43	GC04M025	6.19
1922	PROS1	Protein S	Protein Co		47	GC03M093	6.19
1923	TTN	Titin	Protein Co		47	GC02M178	6.19
1924	CKB	Creatine Ki	Protein Co		45	GC14M103	6.19
1925	PCDH19	Protocadh	Protein Co		40	GC0XM100	6.19
1926	SELENON	Selenoprot	Protein Co		32	GC01P025	6.19
1927	HLA-DRA	Major Hist	Protein Co		47	GC06P032	6.18
1928	ATP6AP2	ATPase H+	Protein Co		44	GC0XP040	6.18
1929	P2RX7	Purinergic	Protein Co		45	GC12P122	6.18
1930	CFL1	Cofilin 1	Protein Co		45	GC11M065	6.18
1931	MMADHC	Metabolisr	Protein Co		42	GC02M149	6.18
1932	ITCH	Itchy E3 Ul	Protein Co		45	GC20P034	6.16
1933	CDK20	Cyclin Dep	Protein Co		40	GC09M087	6.16

1934	LYN	LYN Proto	Protein Co	50	GC08P055	6.15
1935	NONO	Non-POU	Protein Co	44	GC0XP071	6.15
1936	ATF3	Activating	Protein Co	44	GC01P212	6.15
1937	HOXA-AS2	HOXA Clus	RNA Gene	17	GC07P027	6.15
1938	NR4A1	Nuclear Re	Protein Co	46	GC12P052	6.15
1939	SLC7A8	Solute Car	Protein Co	41	GC14M023	6.14
1940	SPTLC2	Serine Palr	Protein Co	48	GC14M077	6.14
1941	GAS6	Growth Ar	Protein Co	44	GC13M113	6.14
1942	HTR4	5-Hydroxy	Protein Co	45	GC05M148	6.14
1943	CMA1	Chymase 1	Protein Co	43	GC14M024	6.14
1944	PUS10	Pseudouric	Protein Co	35	GC02M060	6.14
1945	ZMPSTE24	Zinc Metal	Protein Co	43	GC01P040	6.13
1946	HIRA	Histone Ce	Protein Co	42	GC22M019	6.13
1947	NDUFAF2	NADH:Ubi	Protein Co	40	GC05P060	6.13
1948	MMP10	Matrix Me	Protein Co	46	GC11M102	6.13
1949	HMBS	Hydroxym	Protein Co	44	GC11P119	6.13
1950	INSR	Insulin Rec	Protein Co	54	GC19M007	6.12
1951	SLC9A2	Solute Car	Protein Co	40	GC02P102	6.12
1952	E2F3	E2F Transc	Protein Co	43	GC06P020	6.1
1953	NEDD4	NEDD4 E3	Protein Co	45	GC15M055	6.1
1954	AGTR2	Angiotensi	Protein Co	43	GC0XP116	6.1
1955	PALLD	Palladin, C	Protein Co	42	GC04P168	6.1
1956	C12orf65	Chromoso	Protein Co	36	GC12P123	6.09
1957	WDR37	WD Repea	Protein Co	36	GC10P001	6.09
1958	IL18BP	Interleukin	Protein Co	39	GC11P071	6.09
1959	AEBP1	AE Binding	Protein Co	39	GC07P044	6.09
1960	CD248	CD248 Mo	Protein Co	39	GC11M066	6.09
1961	DHPS	Deoxyhypu	Protein Co	41	GC19M012	6.08
1962	BCR	BCR Activa	Protein Co	51	GC22P023	6.08
1963	PIGW	Phosphatic	Protein Co	37	GC17P036	6.08
1964	CCL26	C-C Motif	Protein Co	37	GC07M075	6.08
1965	SLC25A22	Solute Car	Protein Co	41	GC11M000	6.08
1966	KANSL1	KAT8 Regu	Protein Co	39	GC17M046	6.08
1967	NUDT10	Nudix Hyd	Protein Co	35	GC0XP051	6.08
1968	GPIHBP1	Glycosylph	Protein Co	35	GC08P143	6.08
1969	VLDLR	Very Low I	Protein Co	50	GC09P002	6.08
1970	FASN	Fatty Acid	Protein Co	49	GC17M082	6.07
1971	ACOX2	Acyl-CoA (	Protein Co	43	GC03M058	6.07
1972	IL1RAPL2	Interleukin	Protein Co	37	GC0XP104	6.07
1973	RBP4	Retinol Bin	Protein Co	44	GC10M093	6.07
1974	SYNE1	Spectrin R	Protein Co	41	GC06M152	6.07
1975	CYP26A1	Cytochrom	Protein Co	44	GC10P093	6.06
1976	ASL	Argininosu	Protein Co	45	GC07P066	6.06

1977	H2BC21	H2B Cluste	Protein Co		31	GC01M149	6.06
1978	BRD4	Bromodom	Protein Co		44	GC19M015	6.05
1979	STUB1	STIP1 Hom	Protein Co		45	GC16P001	6.05
1980	ADAR	Adenosine	Protein Co		44	GC01M154	6.05
1981	SERPINE2	Serpin Fan	Protein Co		41	GC02M223	6.04
1982	ADAMTS5	ADAM Me	Protein Co		42	GC21M026	6.04
1983	TRIM28	Tripartite M	Protein Co		43	GC19P058	6.04
1984	DCAF8	DDB1 And	Protein Co		38	GC01M160	6.04
1985	FZD3	Frizzled Cl	Protein Co		43	GC08P028	6.04
1986	DLL1	Delta Like	Protein Co		45	GC06M170	6.04
1987	CYBC1	Cytochrom	Protein Co		25	GC17M082	6.04
1988	INHA	Inhibin Sul	Protein Co		43	GC02P219	6.03
1989	PF4	Platelet Fa	Protein Co		41	GC04M073	6.02
1990	BRD3	Bromodom	Protein Co		41	GC09M134	6.02
1991	SIK1	Salt Induci	Protein Co		46	GC21M043	6.01
1992	CLDN1	Claudin 1	Protein Co		47	GC03M190	6.01
1993	PSMC6	Proteasom	Protein Co		40	GC14P052	6.01
1994	BLNK	B Cell Link	Protein Co		47	GC10M096	6.01
1995	S100G	S100 Calci	Protein Co		36	GC0XP016	6.01
1996	LMBRD1	LMBR1 Do	Protein Co		39	GC06M069	5.99
1997	RASA1	RAS P21 P	Protein Co		46	GC05P087	5.99
1998	PSMB9	Proteasom	Protein Co		46	GC06P047	5.99
1999	SHBG	Sex Hormo	Protein Co		40	GC17P007	5.98
2000	HJV	Hemojuvel	Protein Co		35	GC01M146	5.98
2001	IL17RA	Interleukin	Protein Co		44	GC22P017	5.98
2002	GADD45A	Growth Ar	Protein Co		44	GC01P067	5.98
2003	SLC1A1	Solute Car	Protein Co		47	GC09P004	5.97
2004	BMP7	Bone Morp	Protein Co		45	GC20M057	5.97
2005	ATP6V0A2	ATPase H+	Protein Co		44	GC12P123	5.97
2006	SCHIP1	Schwannor	Protein Co		35	GC03P159	5.97
2007	LLGL1	LLGL Scrib	Protein Co		39	GC17P018	5.97
2008	TSPO	Translocat	Protein Co		43	GC22P043	5.97
2009	RAD50	RAD50 Do	Protein Co		49	GC05P132	5.97
2010	CTCF	CCCTC-Bin	Protein Co		46	GC16P067	5.97
2011	CEACAM6	CEA Cell A	Protein Co		40	GC19P041	5.96
2012	WIF1	WNT Inhib	Protein Co		45	GC12M065	5.96
2013	BRD2	Bromodom	Protein Co		44	GC06P047	5.96
2014	CAST	Calpastatir	Protein Co		45	GC05P096	5.95
2015	CEACAM1	CEA Cell A	Protein Co		43	GC19M042	5.95
2016	DKK3	Dickkopf V	Protein Co		40	GC11M011	5.95
2017	KLLN	Killin, P53	Protein Co		29	GC10M087	5.95
2018	SLC5A2	Solute Car	Protein Co		47	GC16P031	5.94
2019	AGO2	Argonaute	Protein Co		39	GC08M140	5.94

2020	CEBPD	CCAAT Enl	Protein Co	38	GC08M047	5.93
2021	FABP6	Fatty Acid	Protein Co	42	GC05P160	5.93
2022	NCR1	Natural Cy	Protein Co	37	GC19P054	5.92
2023	SETDB1	SET Domai	Protein Co	41	GC01P150	5.92
2024	PTTG1	PTTG1 Reg	Protein Co	43	GC05P160	5.92
2025	PTS	6-Pyruvoyl	Protein Co	47	GC11P112	5.92
2026	LGALS3BP	Galectin 3	Protein Co	41	GC17M078	5.92
2027	PTGER3	Prostaglan	Protein Co	46	GC01M070	5.92
2028	NDUFV2	NADH:Ubi	Protein Co	44	GC18P009	5.91
2029	OPTN	Optineurin	Protein Co	44	GC10P013	5.91
2030	CARMIL2	Capping P	Protein Co	29	GC16P067	5.91
2031	TYROBP	Transmem	Protein Co	43	GC19M035	5.91
2032	PMAIP1	Phorbol-12	Protein Co	39	GC18P059	5.91
2033	ITGA3	Integrin Su	Protein Co	46	GC17P050	5.91
2034	GYPA	Glycophori	Protein Co	45	GC04M144	5.9
2035	GOLGB1	Golgin B1	Protein Co	37	GC03M121	5.9
2036	GTF2IRD1	GTF2I Repe	Protein Co	42	GC07P074	5.89
2037	KRT10	Keratin 10	Protein Co	42	GC17M040	5.89
2038	SOST	Sclerostin	Protein Co	44	GC17M043	5.89
2039	FLII	FLII Actin F	Protein Co	41	GC17M018	5.89
2040	TBL2	Transducin	Protein Co	39	GC07M073	5.89
2041	ATN1	Atrophin 1	Protein Co	43	GC12P008	5.89
2042	IFT122	Intraflagell	Protein Co	40	GC03P129	5.89
2043	FZD1	Frizzled Cl	Protein Co	45	GC07P091	5.89
2044	TNFRSF4	TNF Recep	Protein Co	43	GC01M001	5.89
2045	COL6A3	Collagen T	Protein Co	44	GC02M237	5.88
2046	UPF3B	UPF3B Reg	Protein Co	39	GC0XM119	5.88
2047	CD22	CD22 Mole	Protein Co	45	GC19P035	5.88
2048	SLC1A7	Solute Car	Protein Co	43	GC01M053	5.88
2049	OAT	Ornithine A	Protein Co	47	GC10M124	5.87
2050	SLIT3	Slit Guidar	Protein Co	41	GC05M168	5.87
2051	ATP1A1	ATPase Na	Protein Co	49	GC01P116	5.87
2052	IL12A-AS1	IL12A Anti	RNA Gene	14	GC03M159	5.87
2053	SLC2A9	Solute Car	Protein Co	45	GC04M009	5.87
2054	RIT1	Ras Like W	Protein Co	45	GC01M155	5.87
2055	HDC	Histidine D	Protein Co	44	GC15M050	5.86
2056	MGP	Matrix Gla	Protein Co	42	GC12M014	5.86
2057	ACADS	Acyl-CoA D	Protein Co	46	GC12P120	5.86
2058	NOX4	NADPH Ox	Protein Co	42	GC11M089	5.86
2059	DGAT2	Diacylglyce	Protein Co	43	GC11P075	5.86
2060	MIR133B	MicroRNA	RNA Gene	21	GC06P052	5.86
2061	LPAR1	Lysophosp	Protein Co	45	GC09M110	5.85
2062	NDUFA1	NADH:Ubi	Protein Co	44	GC0XP119	5.85

2063	CYP17A1	Cytochrom	Protein Co	48	GC10M102	5.85
2064	UCN	Urocortin	Protein Co	37	GC02M027	5.85
2065	FECH	Ferrochela	Protein Co	45	GC18M057	5.85
2066	MCM2	Minichrom	Protein Co	47	GC03P127	5.84
2067	FCAR	Fc Fragme	Protein Co	41	GC19P055	5.83
2068	CRKL	CRK Like P	Protein Co	46	GC22P020	5.83
2069	CD9	CD9 Molec	Protein Co	44	GC12P008	5.83
2070	ALLC	Allantoicas	Protein Co	35	GC02P003	5.82
2071	PRKG1	Protein Kir	Protein Co	51	GC10P050	5.82
2072	GCK	Glucokinas	Protein Co	50	GC07M044	5.82
2073	WDTC1	WD And T	Protein Co	34	GC01P027	5.82
2074	PIWIL1	Piwi Like R	Protein Co	41	GC12P130	5.82
2075	TMPO	Thymopoie	Protein Co	45	GC12P098	5.81
2076	CKM	Creatine Ki	Protein Co	44	GC19M045	5.81
2077	AHR	Aryl Hydro	Protein Co	48	GC07P016	5.81
2078	SSTR3	Somatosta	Protein Co	45	GC22M037	5.81
2079	ALDH5A1	Aldehyde I	Protein Co	46	GC06P024	5.81
2080	TBC1D24	TBC1 Dom	Protein Co	39	GC16P002	5.81
2081	CPB2	Carboxype	Protein Co	44	GC13M046	5.81
2082	MAP3K5	Mitogen-A	Protein Co	47	GC06M136	5.81
2083	KCNH2	Potassium	Protein Co	49	GC07M150	5.81
2084	FGL2	Fibrinogen	Protein Co	39	GC07M077	5.8
2085	UNC13D	Unc-13 Ho	Protein Co	43	GC17M075	5.8
2086	EXOSC3	Exosome C	Protein Co	42	GC09M037	5.8
2087	TUBB1	Tubulin Be	Protein Co	47	GC20P059	5.8
2088	F13B	Coagulatio	Protein Co	40	GC01M197	5.79
2089	EPOR	Erythropoi	Protein Co	48	GC19M011	5.78
2090	TRPC1	Transient F	Protein Co	42	GC03P142	5.78
2091	MIR335	MicroRNA	RNA Gene	18	GC07P130	5.78
2092	IGHM	Immunogl	Protein Co	31	GC14M109	5.78
2093	RRM2	Ribonuclec	Protein Co	48	GC02P010	5.77
2094	UBB	Ubiquitin E	Protein Co	43	GC17P016	5.77
2095	CSNK1D	Casein Kin	Protein Co	50	GC17M082	5.77
2096	PTK2B	Protein Ty	Protein Co	49	GC08P027	5.77
2097	RBP2	Retinol Bin	Protein Co	39	GC03M139	5.77
2098	ARSB	Arylsulfata	Protein Co	44	GC05M078	5.76
2099	DLAT	Dihydrolip	Protein Co	46	GC11P112	5.76
2100	MT-TS2	Mitochond	RNA Gene	13	GCMTP012	5.76
2101	ABCC8	ATP Bindir	Protein Co	45	GC11M017	5.76
2102	SLC39A8	Solute Car	Protein Co	43	GC04M102	5.75
2103	SP3	Sp3 Transc	Protein Co	42	GC02M173	5.75
2104	SMTN	Smoothelir	Protein Co	39	GC22P031	5.74
2105	RTN4R	Reticulon 4	Protein Co	43	GC22M020	5.74

2106	MEP1B	Meprin A S	Protein Co	40	GC18P032	5.74
2107	TACSTD2	Tumor Ass	Protein Co	43	GC01M058	5.74
2108	TOMM40	Translocas	Protein Co	40	GC19P044	5.74
2109	HELLS	Helicase, L	Protein Co	45	GC10P094	5.73
2110	DHODH	Dihydroor	Protein Co	45	GC16P072	5.73
2111	UBE2D1	Ubiquitin (	Protein Co	45	GC10P058	5.73
2112	DR1	Down-Reg	Protein Co	39	GC01P093	5.73
2113	MSMO1	Methylster	Protein Co	43	GC04P165	5.72
2114	MGME1	Mitochond	Protein Co	35	GC20P017	5.72
2115	CAPN1	Calpain 1	Protein Co	49	GC11P065	5.72
2116	FYN	FYN Proto	Protein Co	48	GC06M111	5.71
2117	CCR4	C-C Motif	Protein Co	45	GC03P032	5.71
2118	EFNB2	Ephrin B2	Protein Co	44	GC13M106	5.7
2119	NRP2	Neuropilin	Protein Co	43	GC02P205	5.7
2120	TMEM165	Transmem	Protein Co	39	GC04P055	5.7
2121	MIR31	MicroRNA	RNA Gene	20	GC09M021	5.7
2122	RUNX1T1	RUNX1 Pa	Protein Co	39	GC08M091	5.7
2123	DRD4	Dopamine	Protein Co	46	GC11P000	5.69
2124	TPSAB1	Tryptase A	Protein Co	43	GC16P001	5.69
2125	RDX	Radixin	Protein Co	48	GC11M109	5.69
2126	MIR125B1	MicroRNA	RNA Gene	21	GC11M122	5.69
2127	NACC1	Nucleus A	Protein Co	40	GC19P013	5.69
2128	HADHA	Hydroxyac	Protein Co	45	GC02M026	5.69
2129	CD79B	CD79b Mo	Protein Co	47	GC17M063	5.69
2130	HADHB	Hydroxyac	Protein Co	47	GC02P026	5.68
2131	ARL13B	ADP Ribos	Protein Co	37	GC03P093	5.68
2132	ANO5	Anoctamin	Protein Co	38	GC11P021	5.68
2133	AKR1B1	Aldo-Keto	Protein Co	47	GC07M134	5.68
2134	SLC51A	Solute Car	Protein Co	37	GC03P196	5.68
2135	RPS6KB2	Ribosomal	Protein Co	46	GC11P067	5.68
2136	TARDBP	TAR DNA I	Protein Co	45	GC01P011	5.67
2137	PCSK5	Proprotein	Protein Co	41	GC09P075	5.67
2138	HBA2	Hemoglob	Protein Co	42	GC16P001	5.67
2139	PNPO	Pyridoxam	Protein Co	45	GC17P047	5.67
2140	ALYREF	Aly/REF Ex	Protein Co	37	GC17M081	5.66
2141	SLC22A2	Solute Car	Protein Co	44	GC06M160	5.66
2142	CD151	CD151 Mo	Protein Co	45	GC11P000	5.66
2143	MCC	MCC Regu	Protein Co	40	GC05M113	5.66
2144	FOXH1	Forkhead I	Protein Co	41	GC08M144	5.66
2145	ABCC4	ATP Bindir	Protein Co	45	GC13M095	5.66
2146	SLC5A6	Solute Car	Protein Co	44	GC02M027	5.66
2147	FERMT3	Fermitin Fa	Protein Co	43	GC11P064	5.65
2148	DLG4	Discs Larg	Protein Co	47	GC17M007	5.65

2149	FGF19	Fibroblast	Protein Co	44	GC11M069	5.65
2150	ADGRG1	Adhesion (	Protein Co	39	GC16P057	5.65
2151	MIR345	MicroRNA	RNA Gene	19	GC14P100	5.64
2152	CD3D	CD3d Mol	Protein Co	47	GC11M118	5.64
2153	PRDM1	PR/SET Do	Protein Co	45	GC06P105	5.64
2154	C3AR1	Compleme	Protein Co	43	GC12M008	5.64
2155	KRIT1	KRIT1 Ank	Protein Co	41	GC07M092	5.64
2156	LINC01772	Long Inter	RNA Gene	10	GC01P016	5.64
2157	MIR215	MicroRNA	RNA Gene	18	GC01M220	5.64
2158	RICTOR	RPTOR Ind	Protein Co	44	GC05M038	5.63
2159	ACACA	Acetyl-CoA	Protein Co	48	GC17M037	5.63
2160	HBA1	Hemoglob	Protein Co	42	GC16P001	5.63
2161	TNFRSF8	TNF Recep	Protein Co	42	GC01P012	5.63
2162	AKR7A2	Aldo-Keto	Protein Co	42	GC01M019	5.63
2163	BLOC1S6	Biogenesis	Protein Co	39	GC15P045	5.62
2164	LRG1	Leucine Ri	Protein Co	39	GC19M004	5.61
2165	MARK4	Microtubul	Protein Co	44	GC19P045	5.61
2166	COPB2	COPI Coat	Protein Co	44	GC03M139	5.61
2167	LIPA	Lipase A, L	Protein Co	47	GC10M089	5.61
2168	HUWE1	HECT, UBA	Protein Co	44	GC0XM053	5.6
2169	LAMA4	Laminin Su	Protein Co	44	GC06M112	5.6
2170	KLRC2	Killer Cell I	Protein Co	37	GC12M013	5.6
2171	KRT17	Keratin 17	Protein Co	45	GC17M041	5.6
2172	INHBA	Inhibin Sul	Protein Co	45	GC07M041	5.6
2173	BBS1	Bardet-Bie	Protein Co	38	GC11P066	5.6
2174	SYMPK	Symplekin	Protein Co	39	GC19M045	5.59
2175	FBLIM1	Filamin Bir	Protein Co	38	GC01P015	5.59
2176	IFT88	Intraflagell	Protein Co	40	GC13P020	5.58
2177	KLRC4	Killer Cell I	Protein Co	36	GC12M013	5.58
2178	TALDO1	Transaldola	Protein Co	47	GC11P000	5.58
2179	WNT7B	Wnt Family	Protein Co	43	GC22M045	5.57
2180	AR	Androgen	Protein Co	53	GC0XP067	5.56
2181	MIR106A	MicroRNA	RNA Gene	18	GC0XM134	5.56
2182	SNRNP70	Small Nucl	Protein Co	38	GC19P049	5.56
2183	EIF2B4	Eukaryotic	Protein Co	44	GC02M027	5.56
2184	REG3G	Regenerati	Protein Co	35	GC02P078	5.56
2185	SP100	SP100 Nuc	Protein Co	41	GC02P230	5.56
2186	KLK6	Kallikrein F	Protein Co	42	GC19M050	5.55
2187	MIR183	MicroRNA	RNA Gene	18	GC07M129	5.55
2188	PSMD2	Proteasom	Protein Co	43	GC03P184	5.55
2189	EPHA3	EPH Recep	Protein Co	45	GC03P089	5.55
2190	PON2	Paraoxona	Protein Co	42	GC07M095	5.54
2191	DRD3	Dopamine	Protein Co	44	GC03M114	5.54

2192	SLC6A3	Solute Car	Protein Co	49	GC05M001	5.54
2193	RAB7A	RAB7A, Me	Protein Co	48	GC03P128	5.53
2194	WNT1	Wnt Family	Protein Co	46	GC12P049	5.53
2195	GALK1	Galactokin	Protein Co	48	GC17M075	5.53
2196	APOA5	Apolipopro	Protein Co	44	GC11M116	5.53
2197	SUFU	SUFU Neg	Protein Co	43	GC10P102	5.52
2198	AP1S3	Adaptor R	Protein Co	40	GC02M223	5.52
2199	ABCA2	ATP Bindir	Protein Co	41	GC09M137	5.52
2200	COX7B	Cytochrom	Protein Co	40	GC0XP077	5.52
2201	NPC2	NPC Intrac	Protein Co	41	GC14M074	5.52
2202	PLEK	Pleckstrin	Protein Co	40	GC02P068	5.51
2203	CXCR5	C-X-C Mot	Protein Co	41	GC11P118	5.51
2204	ORMDL3	ORMDL Sp	Protein Co	39	GC17M039	5.5
2205	PRKDC	Protein Kir	Protein Co	49	GC08M047	5.5
2206	WNT5B	Wnt Family	Protein Co	44	GC12P001	5.5
2207	ACIN1	Apoptotic	Protein Co	39	GC14M023	5.5
2208	MIR193B	MicroRNA	RNA Gene	20	GC16P014	5.5
2209	PGM3	Phosphogl	Protein Co	43	GC06M083	5.49
2210	WDR20	WD Repea	Protein Co	35	GC14P104	5.49
2211	MASP1	Mannan Bi	Protein Co	46	GC03M187	5.49
2212	BARX1	BARX Hom	Protein Co	37	GC09M093	5.49
2213	SLAMF1	Signaling L	Protein Co	40	GC01M160	5.49
2214	MIA2	MIA SH3 D	Protein Co	37	GC14P039	5.48
2215	PADI4	Peptidyl Ar	Protein Co	44	GC01P017	5.48
2216	FANCG	FA Comple	Protein Co	43	GC09M035	5.48
2217	SNRPN	Small Nucl	Protein Co	44	GC15P024	5.47
2218	SMAD1	SMAD Fam	Protein Co	44	GC04P145	5.47
2219	CYREN	Cell Cycle	Protein Co	25	GC07M135	5.47
2220	RPL6	Ribosomal	Protein Co	39	GC12M112	5.47
2221	GRIA3	Glutamate	Protein Co	50	GC0XP123	5.47
2222	AGL	Amylo-Alp	Protein Co	45	GC01P099	5.47
2223	MATN1	Matrilin 1	Protein Co	39	GC01M030	5.47
2224	CPVL	Carboxype	Protein Co	40	GC07M028	5.46
2225	GPC1	Glypican 1	Protein Co	43	GC02P240	5.46
2226	SPAG6	Sperm Ass	Protein Co	36	GC10P022	5.46
2227	CNP	2',3'-Cyclic	Protein Co	42	GC17P041	5.46
2228	ENTPD1	Ectonuclec	Protein Co	46	GC10P095	5.45
2229	PPARD	Peroxisom	Protein Co	47	GC06P047	5.45
2230	PRKCQ	Protein Kir	Protein Co	49	GC10M006	5.45
2231	FTH1	Ferritin He	Protein Co	50	GC11M061	5.45
2232	GNAI1	G Protein S	Protein Co	46	GC07P079	5.44
2233	HDAC6	Histone De	Protein Co	51	GC0XP048	5.44
2234	MIR28	MicroRNA	RNA Gene	19	GC03P188	5.44

2235	MARK1	Microtubul	Protein Co	41	GC01P220	5.44
2236	RAB5A	RAB5A, Me	Protein Co	45	GC03P019	5.44
2237	GABRG2	Gamma-Ar	Protein Co	46	GC05P162	5.43
2238	SLC9A7	Solute Car	Protein Co	37	GC0XM046	5.43
2239	HCFC1	Host Cell F	Protein Co	46	GC0XM153	5.42
2240	DCT	Dopachror	Protein Co	41	GC13M094	5.42
2241	ACSL5	Acyl-CoA S	Protein Co	42	GC10P112	5.42
2242	UGT2B7	UDP Glucu	Protein Co	44	GC04P069	5.42
2243	SAG	S-Antigen	Protein Co	44	GC02P233	5.42
2244	GJB6	Gap Juncti	Protein Co	44	GC13M020	5.42
2245	SIX5	SIX Homec	Protein Co	39	GC19M045	5.42
2246	CEBPA	CCAAT Enl	Protein Co	47	GC19M033	5.42
2247	HDAC4	Histone De	Protein Co	51	GC02M239	5.41
2248	GLUL	Glutamate	Protein Co	49	GC01M182	5.41
2249	DRD5	Dopamine	Protein Co	47	GC04P009	5.41
2250	MPL	MPL Proto	Protein Co	48	GC01P043	5.4
2251	ST6GAL1	ST6 Beta-G	Protein Co	43	GC03P186	5.39
2252	HP1BP3	Heterochro	Protein Co	37	GC01M020	5.39
2253	PCNT	Pericentrin	Protein Co	41	GC21P046	5.38
2254	PSMA5	Proteasom	Protein Co	43	GC01M109	5.37
2255	ARF4	ADP Ribos	Protein Co	43	GC03M057	5.37
2256	TMEFF2	Transmem	Protein Co	39	GC02M191	5.37
2257	MELK	Maternal E	Protein Co	45	GC09P036	5.37
2258	PKP1	Plakophilir	Protein Co	41	GC01P201	5.37
2259	DRC3	Dynein Re	Protein Co	28	GC17P017	5.37
2260	SLIT1	Slit Guidar	Protein Co	43	GC10M096	5.36
2261	ANO1	Anoctamin	Protein Co	41	GC11P069	5.35
2262	DPYSL5	Dihydropy	Protein Co	37	GC02P026	5.35
2263	MIR494	MicroRNA	RNA Gene	17	GC14P104	5.34
2264	MLANA	Melan-A	Protein Co	39	GC09P005	5.34
2265	SLC19A1	Solute Car	Protein Co	45	GC21M045	5.34
2266	COL18A1	Collagen T	Protein Co	45	GC21P045	5.34
2267	SMCR8	SMCR8-C9	Protein Co	32	GC17P018	5.34
2268	LAD1	Ladinin 1	Protein Co	36	GC01M201	5.33
2269	CXCL11	C-X-C Mot	Protein Co	40	GC04M076	5.33
2270	RPS3A	Ribosomal	Protein Co	41	GC04P151	5.33
2271	MT-TS1	Mitochond	RNA Gene	14	GCMTM00	5.33
2272	MT-TH	Mitochond	RNA Gene	13	GCMTP012	5.33
2273	MT-TW	Mitochond	RNA Gene	12	GCMTP005	5.33
2274	MT-TQ	Mitochond	RNA Gene	12	GCMTM00	5.33
2275	ADAD1	Adenosine	Protein Co	36	GC04P122	5.32
2276	LINC01193	Long Inter	RNA Gene	13	GC15P021	5.32
2277	MCFD2	Multiple C	Protein Co	43	GC02M046	5.32

2278	CAGE1	Cancer An	Protein Co	35	GC06M007	5.32
2279	ALOX12	Arachidon	Protein Co	43	GC17P006	5.32
2280	BICD2	BICD Carg	Protein Co	40	GC09M092	5.32
2281	NPTX2	Neuronal I	Protein Co	39	GC07P098	5.32
2282	FGD4	FYVE, Rho	Protein Co	43	GC12P032	5.31
2283	CCNA1	Cyclin A1	Protein Co	42	GC13P036	5.31
2284	NUP133	Nucleopor	Protein Co	40	GC01M229	5.31
2285	MIR625	MicroRNA	RNA Gene	14	GC14P065	5.31
2286	ETV6	ETS Varian	Protein Co	46	GC12P011	5.31
2287	SACS	Sacsin Mol	Protein Co	36	GC13M023	5.31
2288	RARS1	Arginyl-TR	Protein Co	35	GC05P168	5.31
2289	SLAMF7	SLAM Fam	Protein Co	41	GC01P160	5.3
2290	RNASEL	Ribonuclea	Protein Co	44	GC01M182	5.3
2291	CAPN3	Calpain 3	Protein Co	47	GC15P042	5.3
2292	KAT6B	Lysine Ace	Protein Co	41	GC10P074	5.3
2293	MIR203A	MicroRNA	RNA Gene	19	GC14P104	5.29
2294	CCL18	C-C Motif	Protein Co	36	GC17P036	5.29
2295	C2	Compleme	Protein Co	44	GC06P031	5.29
2296	SRP54	Signal Rec	Protein Co	43	GC14P034	5.29
2297	RELN	Reelin	Protein Co	43	GC07M103	5.29
2298	CAPN2	Calpain 2	Protein Co	47	GC01P223	5.29
2299	POLG2	DNA Polyr	Protein Co	41	GC17M064	5.29
2300	BGN	Biglycan	Protein Co	43	GC0XP153	5.28
2301	PDGFRL	Platelet De	Protein Co	41	GC08P017	5.28
2302	CCKBR	Cholecysto	Protein Co	44	GC11P006	5.28
2303	NDUFS4	NADH:Ubi	Protein Co	44	GC05P053	5.28
2304	NPSR1	Neuropept	Protein Co	40	GC07P034	5.28
2305	KLHL40	Kelch Like	Protein Co	37	GC03P042	5.27
2306	ORMDL1	ORMDL Sp	Protein Co	35	GC02M189	5.27
2307	GDAP1	Gangliosid	Protein Co	42	GC08P074	5.27
2308	ABCB7	ATP Bindir	Protein Co	43	GC0XM075	5.26
2309	NDUFB3	NADH:Ubi	Protein Co	42	GC02P201	5.26
2310	SLC7A5	Solute Car	Protein Co	44	GC16M087	5.26
2311	PXN	Paxillin	Protein Co	45	GC12M120	5.25
2312	POU2AF1	POU Class	Protein Co	37	GC11M111	5.25
2313	DBN1	Drebrin 1	Protein Co	39	GC05M177	5.25
2314	FOLR1	Folate Rec	Protein Co	47	GC11P072	5.25
2315	PPP1R12C	Protein Ph	Protein Co	36	GC19M055	5.25
2316	ISL1	ISL LIM Ho	Protein Co	45	GC05P051	5.25
2317	AKAP9	A-Kinase A	Protein Co	43	GC07P091	5.25
2318	SLC6A6	Solute Car	Protein Co	44	GC03P014	5.24
2319	EFL1	Elongation	Protein Co	32	GC15M082	5.24
2320	DDB1	Damage S	Protein Co	41	GC11M061	5.24

2321	FBL	Fibrillarin	Protein Co	43	GC19M039	5.23
2322	IQGAP1	IQ Motif C	Protein Co	43	GC15P090	5.23
2323	GNA11	G Protein !	Protein Co	47	GC19P003	5.23
2324	SPAG17	Sperm Ass	Protein Co	34	GC01M117	5.23
2325	SPIB	Spi-B Tran	Protein Co	40	GC19P050	5.23
2326	MYO1A	Myosin IA	Protein Co	40	GC12M057	5.22
2327	RTN4IP1	Reticulon 4	Protein Co	41	GC06M106	5.22
2328	CDC45	Cell Divisic	Protein Co	44	GC22P019	5.22
2329	KDM5C	Lysine Den	Protein Co	43	GC0XM053	5.22
2330	DIS3L2	DIS3 Like 3	Protein Co	43	GC02P231	5.22
2331	CD1D	CD1d Mol	Protein Co	42	GC01P158	5.22
2332	KCNA2	Potassium	Protein Co	45	GC01M110	5.22
2333	PTPRJ	Protein Ty	Protein Co	45	GC11P048	5.21
2334	IFNGR2	Interferon	Protein Co	43	GC21P033	5.21
2335	TUBB2A	Tubulin Be	Protein Co	45	GC06M003	5.21
2336	RBP3	Retinol Bin	Protein Co	40	GC10P047	5.2
2337	ANXA6	Annexin A	Protein Co	43	GC05M151	5.2
2338	FHL2	Four And A	Protein Co	45	GC02M105	5.2
2339	SLC18A2	Solute Car	Protein Co	47	GC10P117	5.19
2340	GRIA1	Glutamate	Protein Co	48	GC05P153	5.19
2341	MLF1	Myeloid Le	Protein Co	43	GC03P158	5.19
2342	AUTS2	Activator C	Protein Co	39	GC07P069	5.18
2343	VEGFD	Vascular E	Protein Co	34	GC0XM015	5.18
2344	FABP3	Fatty Acid	Protein Co	44	GC01M031	5.17
2345	GPR31	G Protein-(	Protein Co	34	GC06M167	5.17
2346	USP9X	Ubiquitin S	Protein Co	47	GC0XP041	5.17
2347	MYOM2	Myomesin	Protein Co	39	GC08P002	5.17
2348	VKORC1	Vitamin K	Protein Co	47	GC16M031	5.17
2349	KATNIP	Katanin Int	Protein Co	28	GC16P027	5.17
2350	MIR130B	MicroRNA	RNA Gene	17	GC22P024	5.16
2351	PDGFD	Platelet De	Protein Co	44	GC11M103	5.16
2352	FERMT2	Fermitin Fa	Protein Co	38	GC14M052	5.16
2353	DEFB1	Defensin B	Protein Co	39	GC08M006	5.16
2354	UBR1	Ubiquitin F	Protein Co	43	GC15M042	5.16
2355	ARSH	Arylsulfata	Protein Co	34	GC0XP003	5.16
2356	KCNQ2	Potassium	Protein Co	47	GC20M063	5.15
2357	WHCR	Wolf-Hirsc	Genetic Lo	4	GC04U990	5.15
2358	CA1	Carbonic A	Protein Co	47	GC08M085	5.15
2359	DYNLL1	Dynein Lig	Protein Co	42	GC12P120	5.15
2360	SELPLG	Selectin P	Protein Co	42	GC12M108	5.14
2361	FGF18	Fibroblast	Protein Co	41	GC05P171	5.14
2362	SCTR	Secretin Re	Protein Co	41	GC02M119	5.14
2363	MAGEA3	MAGE Fam	Protein Co	36	GC0XP152	5.14

2364	DPEP1	Dipeptidas	Protein Co	42	GC16P089	5.14
2365	WNT16	Wnt Family	Protein Co	41	GC07P121	5.14
2366	ASPRV1	Aspartic Pe	Protein Co	34	GC02M069	5.13
2367	TNS1	Tensin 1	Protein Co	39	GC02M217	5.13
2368	SLC26A6	Solute Carr	Protein Co	39	GC03M048	5.13
2369	PDZD3	PDZ Domai	Protein Co	35	GC11P119	5.13
2370	SPART	Spartin	Protein Co	31	GC13M036	5.13
2371	DNAJC21	DnaJ Heat	Protein Co	35	GC05P034	5.12
2372	MYH7	Myosin He	Protein Co	47	GC14M023	5.12
2373	SLC12A6	Solute Carr	Protein Co	48	GC15M034	5.12
2374	MSLN	Mesothelin	Protein Co	40	GC16P001	5.12
2375	UBE2N	Ubiquitin C	Protein Co	47	GC12M093	5.12
2376	KLK8	Kallikrein F	Protein Co	41	GC19M050	5.12
2377	GBE1	1,4-Alpha-	Protein Co	44	GC03M081	5.11
2378	HMGCS2	3-Hydroxy	Protein Co	44	GC01M119	5.11
2379	DGCR2	DiGeorge S	Protein Co	39	GC22M019	5.1
2380	ERCC5	ERCC Excis	Protein Co	45	GC13P102	5.1
2381	REST	RE1 Silenci	Protein Co	44	GC04P056	5.1
2382	PTH1R	Parathyroi	Protein Co	50	GC03P046	5.1
2383	SLC9A3R1	SLC9A3 Re	Protein Co	44	GC17P074	5.1
2384	KLKB1	Kallikrein B	Protein Co	47	GC04P186	5.09
2385	ANKRD50	Ankyrin Re	Protein Co	33	GC04M124	5.09
2386	CCN1	Cellular Co	Protein Co	31	GC01P085	5.09
2387	CYP11A1	Cytochrom	Protein Co	48	GC15M074	5.09
2388	PPIB	Peptidylpr	Protein Co	50	GC15M064	5.09
2389	ADGRV1	Adhesion G	Protein Co	35	GC05P090	5.09
2390	NDUFA5	NADH:Ubi	Protein Co	41	GC07M123	5.09
2391	UCP1	Uncoupling	Protein Co	44	GC04M140	5.08
2392	NID1	Nidogen 1	Protein Co	41	GC01M235	5.08
2393	KCNB1	Potassium	Protein Co	47	GC20M049	5.07
2394	EIF4E	Eukaryotic	Protein Co	50	GC04M098	5.07
2395	NXN	Nucleoredo	Protein Co	41	GC17M000	5.07
2396	ARTN	Artemin	Protein Co	40	GC01P043	5.07
2397	WNT10A	Wnt Family	Protein Co	45	GC02P218	5.07
2398	BID	BH3 Intera	Protein Co	45	GC22M017	5.07
2399	DMAP1	DNA Meth	Protein Co	37	GC01P044	5.07
2400	RHEB	Ras Homo	Protein Co	48	GC07M151	5.06
2401	TSPAN4	Tetraspanin	Protein Co	36	GC11P000	5.06
2402	DYSF	Dysferlin	Protein Co	43	GC02P071	5.06
2403	CLTRN	Collectrin,	Protein Co	28	GC0XM015	5.06
2404	SCN10A	Sodium Vo	Protein Co	45	GC03M038	5.06
2405	SLC39A13	Solute Carr	Protein Co	43	GC11P047	5.06
2406	PROC	Protein C,	Protein Co	49	GC02P127	5.06

2407	AP1M1	Adaptor R	Protein Co	40	GC19P023	5.06
2408	PLK4	Polo Like I	Protein Co	47	GC04P127	5.05
2409	TNPO3	Transportii	Protein Co	39	GC07M128	5.05
2410	MIR296	MicroRNA	RNA Gene	17	GC20M058	5.05
2411	RHOB	Ras Homo	Protein Co	44	GC02P020	5.04
2412	COL11A2	Collagen T	Protein Co	44	GC06M033	5.04
2413	NR4A2	Nuclear Re	Protein Co	46	GC02M156	5.03
2414	DCLRE1C	DNA Cross	Protein Co	44	GC10M014	5.03
2415	FBXO11	F-Box Prot	Protein Co	42	GC02M047	5.03
2416	GART	Phosphoril	Protein Co	41	GC21M033	5.03
2417	NEK9	NIMA Rela	Protein Co	45	GC14M075	5.03
2418	SIGIRR	Single Ig A	Protein Co	40	GC11M000	5.03
2419	MUC12	Mucin 12,	Protein Co	31	GC07P100	5.03
2420	RIPK3	Receptor I	Protein Co	43	GC14M024	5.03
2421	PAK1	P21 (RAC1	Protein Co	47	GC11M077	5.02
2422	WASL	WASP Like	Protein Co	41	GC07M123	5.02
2423	GPC4	Glypican 4	Protein Co	45	GC0XM133	5.02
2424	KIR3DL1	Killer Cell I	Protein Co	40	GC19P055	5.01
2425	SLC16A1	Solute Car	Protein Co	48	GC01M112	5.01
2426	SRSF2	Serine Anc	Protein Co	40	GC17M076	5.01
2427	TBX2	T-Box Tran	Protein Co	45	GC17P061	5.01
2428	GAMT	Guanidino	Protein Co	46	GC19M001	5
2429	KDM1A	Lysine Den	Protein Co	47	GC01P023	4.99
2430	NKX2-3	NK2 Home	Protein Co	37	GC10P099	4.99
2431	ALAD	Aminolevu	Protein Co	46	GC09M113	4.99
2432	SLAMF6	SLAM Fam	Protein Co	40	GC01M160	4.99
2433	FBLN2	Fibulin 2	Protein Co	43	GC03P013	4.98
2434	CDH3	Cadherin 3	Protein Co	47	GC16P068	4.98
2435	IPMK	Inositol Po	Protein Co	39	GC10M058	4.98
2436	H2AC20	H2A Cluste	Protein Co	31	GC01P149	4.98
2437	DNM1L	Dynamin 1	Protein Co	47	GC12P032	4.98
2438	PNPLA3	Patatin Lik	Protein Co	41	GC22P043	4.98
2439	EPM2AIP1	EPM2A Int	Protein Co	35	GC03M036	4.98
2440	SLC29A3	Solute Car	Protein Co	43	GC10P071	4.97
2441	CD47	CD47 Mole	Protein Co	43	GC03M108	4.97
2442	IFI16	Interferon	Protein Co	42	GC01P158	4.97
2443	ACTN4	Actinin Alp	Protein Co	46	GC19P038	4.97
2444	UGT1A6	UDP Glucu	Protein Co	40	GC02P233	4.97
2445	HEPH	Hephaestir	Protein Co	39	GC0XP066	4.97
2446	RAG2	Recombina	Protein Co	42	GC11M036	4.97
2447	PIK3R5	Phosphoin	Protein Co	45	GC17M008	4.97
2448	WRNIP1	WRN Helic	Protein Co	36	GC06P002	4.97
2449	COL10A1	Collagen T	Protein Co	43	GC06M116	4.97

2450	FMO5	Flavin Con	Protein Co	41	GC01M147	4.96
2451	CD99	CD99 Mole	Protein Co	40	GC0XP002	4.96
2452	SERPINB3	Serpin Fan	Protein Co	42	GC18M063	4.96
2453	RORA	RAR Relate	Protein Co	48	GC15M060	4.96
2454	COG8	Componer	Protein Co	40	GC16M069	4.95
2455	CXADR	CXADR Ig-	Protein Co	43	GC21P017	4.95
2456	PRMT7	Protein Ar	Protein Co	44	GC16P068	4.95
2457	ACP6	Acid Phosp	Protein Co	40	GC01M147	4.95
2458	PPL	Periplakin	Protein Co	40	GC16M004	4.94
2459	SPTBN1	Spectrin B	Protein Co	43	GC02P054	4.94
2460	KCNN3	Potassium	Protein Co	44	GC01M154	4.94
2461	SLC36A1	Solute Car	Protein Co	39	GC05P151	4.94
2462	CTRL	Chymotryp	Protein Co	40	GC16M067	4.93
2463	EBP	EBP Chole	Protein Co	43	GC0XP048	4.92
2464	HK2	Hexokinas	Protein Co	47	GC02P074	4.92
2465	TMEM126E	Transmem	Protein Co	39	GC11P085	4.92
2466	TBX4	T-Box Tran	Protein Co	41	GC17P061	4.92
2467	EMP2	Epithelial M	Protein Co	42	GC16M010	4.91
2468	CHIT1	Chitinase 1	Protein Co	43	GC01M203	4.91
2469	TASP1	Taspase 1	Protein Co	36	GC20M013	4.91
2470	SDHAF2	Succinate I	Protein Co	41	GC11P061	4.91
2471	SLC27A4	Solute Car	Protein Co	45	GC09P128	4.91
2472	TMPRSS6	Transmem	Protein Co	44	GC22M037	4.91
2473	SPG11	SPG11 Ves	Protein Co	39	GC15M044	4.91
2474	TNFRSF6B	TNF Recep	Protein Co	43	GC20P063	4.9
2475	DNTT	DNA Nucle	Protein Co	42	GC10P096	4.9
2476	AIM2	Absent In	Protein Co	41	GC01M159	4.89
2477	SLC22A3	Solute Car	Protein Co	44	GC06P160	4.89
2478	CNBP	CCHC-Type	Protein Co	41	GC03M129	4.89
2479	AIMP2	Aminoacyl	Protein Co	41	GC07P006	4.89
2480	LINC-PINT	Long Inter	RNA Gene	17	GC07M130	4.89
2481	SLC35A3	Solute Car	Protein Co	42	GC01P099	4.88
2482	UHRF1	Ubiquitin L	Protein Co	40	GC19P004	4.88
2483	MMD	Monocyte	Protein Co	35	GC17M055	4.88
2484	CD63	CD63 Mole	Protein Co	41	GC12M055	4.88
2485	PIK3CD	Phosphatic	Protein Co	53	GC01P009	4.88
2486	KANK2	KN Motif A	Protein Co	39	GC19M011	4.88
2487	IRAK3	Interleukin	Protein Co	48	GC12P066	4.88
2488	DARS2	Aspartyl-TI	Protein Co	41	GC01P173	4.88
2489	SNHG3	Small Nucl	RNA Gene	17	GC01P028	4.87
2490	NAA15	N-Alpha-A	Protein Co	39	GC04P139	4.87
2491	ITGB7	Integrin Su	Protein Co	44	GC12M053	4.87
2492	SLC1A2	Solute Car	Protein Co	48	GC11M035	4.86

2493	MAP2	Microtubu	Protein Co	42	GC02P209	4.86
2494	SOD3	Superoxide	Protein Co	40	GC04P024	4.86
2495	PSMA6	Proteasom	Protein Co	46	GC14P035	4.86
2496	MIR30D	MicroRNA	RNA Gene	17	GC08M134	4.86
2497	RELB	RELB Prot	Protein Co	45	GC19P045	4.86
2498	LIPC	Lipase C, H	Protein Co	44	GC15P058	4.86
2499	NR1H3	Nuclear Re	Protein Co	47	GC11P047	4.86
2500	SCN5A	Sodium Vo	Protein Co	50	GC03M038	4.85
2501	SYNJ1	Synaptojar	Protein Co	45	GC21M032	4.85
2502	XRCC2	X-Ray Rep	Protein Co	42	GC07M152	4.85
2503	GABBR2	Gamma-Ar	Protein Co	47	GC09M098	4.85
2504	WNT5A	Wnt Family	Protein Co	49	GC03M055	4.84
2505	PCDH12	Protocadh	Protein Co	39	GC05M141	4.84
2506	SETD1A	SET Domai	Protein Co	40	GC16P030	4.84
2507	MSX2	Msh Home	Protein Co	47	GC05P174	4.84
2508	SOX17	SRY-Box Tr	Protein Co	43	GC08P054	4.84
2509	HOXD13	Homeobox	Protein Co	41	GC02P176	4.84
2510	SPI1	Spi-1 Prot	Protein Co	44	GC11M061	4.84
2511	FKRP	Fukutin Re	Protein Co	40	GC19P046	4.84
2512	GRIA2	Glutamate	Protein Co	48	GC04P157	4.84
2513	GFRA2	GDNF Fam	Protein Co	43	GC08M021	4.84
2514	DCDC2	Doublecor	Protein Co	39	GC06M024	4.84
2515	TBP	TATA-Box	Protein Co	48	GC06P170	4.84
2516	DCXR	Dicarbonyl	Protein Co	44	GC17M082	4.84
2517	PPARGC1A	PPARG Co	Protein Co	46	GC04M023	4.84
2518	GDF7	Growth Dif	Protein Co	39	GC02P020	4.83
2519	CCKAR	Cholecystc	Protein Co	45	GC04M026	4.83
2520	LMNB1	Lamin B1	Protein Co	47	GC05P126	4.83
2521	HGD	Homogent	Protein Co	44	GC03M120	4.83
2522	HYOU1	Hypoxia U	Protein Co	43	GC11M119	4.83
2523	RPS14	Ribosomal	Protein Co	44	GC05M150	4.83
2524	BCYRN1	Brain Cyto	RNA Gene	18	GC02P047	4.83
2525	HSPB2	Heat Shoc	Protein Co	40	GC11P111	4.82
2526	SLC1A3	Solute Car	Protein Co	50	GC05P036	4.82
2527	CNKSR2	Connector	Protein Co	39	GC0XP021	4.82
2528	GP2	Glycoprote	Protein Co	39	GC16M020	4.82
2529	CD72	CD72 Mole	Protein Co	40	GC09M035	4.82
2530	FANCL	FA Comple	Protein Co	45	GC02M058	4.81
2531	ECHS1	Enoyl-CoA	Protein Co	47	GC10M133	4.81
2532	TRB	T Cell Rece	Protein Co	14	GC07P145	4.81
2533	GDF5	Growth Dif	Protein Co	47	GC20M035	4.81
2534	ALDH9A1	Aldehyde I	Protein Co	41	GC01M165	4.81
2535	ERAP2	Endoplasm	Protein Co	37	GC05P096	4.81

2536	SPTBN2	Spectrin B	Protein Co	41	GC11M066	4.81
2537	RPTOR	Regulatory	Protein Co	44	GC17P080	4.8
2538	PNPLA1	Patatin Lik	Protein Co	37	GC06P047	4.8
2539	MIR375	MicroRNA	RNA Gene	19	GC02M219	4.8
2540	WNT3	Wnt Family	Protein Co	46	GC17M046	4.8
2541	TYRP1	Tyrosinase	Protein Co	46	GC09P012	4.79
2542	PRNP	Prion Prote	Protein Co	47	GC20P004	4.79
2543	PEX10	Peroxisom	Protein Co	41	GC01M002	4.79
2544	ACD	ACD Shelte	Protein Co	43	GC16M067	4.79
2545	NFKB2	Nuclear Fa	Protein Co	52	GC10P102	4.79
2546	MMAA	Metabolisr	Protein Co	40	GC04P145	4.79
2547	IMMT	Inner Mem	Protein Co	39	GC02M086	4.79
2548	MYH6	Myosin He	Protein Co	45	GC14M023	4.78
2549	TNC	Tenascin C	Protein Co	48	GC09M115	4.78
2550	VSIG4	V-Set And	Protein Co	36	GC0XM066	4.78
2551	MIR486-1	MicroRNA	RNA Gene	16	GC08M041	4.78
2552	GABRB3	Gamma-Ar	Protein Co	48	GC15M026	4.77
2553	TNIP1	TNFAIP3 Ir	Protein Co	41	GC05M151	4.77
2554	ELP2	Elongator	Protein Co	37	GC18P036	4.77
2555	BACE2	Beta-Secre	Protein Co	40	GC21P041	4.77
2556	C2CD3	C2 Domair	Protein Co	39	GC11M074	4.77
2557	MMP21	Matrix Me	Protein Co	40	GC10M125	4.76
2558	CGB7	Chorionic	Protein Co	30	GC19M049	4.76
2559	ZNF41	Zinc Finge	Protein Co	40	GC0XM047	4.76
2560	PHKA2	Phosphory	Protein Co	45	GC0XM018	4.76
2561	IFRD1	Interferon	Protein Co	40	GC07P112	4.76
2562	INVS	Inversin	Protein Co	44	GC09P100	4.76
2563	PSMB5	Proteasom	Protein Co	44	GC14M023	4.75
2564	COASY	Coenzyme	Protein Co	45	GC17P042	4.75
2565	MAOB	Monoamin	Protein Co	43	GC0XM043	4.75
2566	ARHGEF16	Rho Guani	Protein Co	39	GC01P003	4.75
2567	TCN1	Transcobal	Protein Co	40	GC11M061	4.75
2568	PQBP1	Polyglutan	Protein Co	40	GC0XP048	4.75
2569	DNAH8	Dynein Axo	Protein Co	37	GC06P047	4.75
2570	PRPS1L1	Phosphoril	Protein Co	37	GC07M018	4.75
2571	FARP2	FERM, ARH	Protein Co	40	GC02P241	4.75
2572	ANKH	ANKH Inor	Protein Co	40	GC05M014	4.74
2573	IL26	Interleukin	Protein Co	34	GC12M068	4.74
2574	PLD3	Phospholip	Protein Co	41	GC19P040	4.74
2575	PTGDR2	Prostaglan	Protein Co	41	GC11M060	4.74
2576	MIR186	MicroRNA	RNA Gene	18	GC01M071	4.73
2577	IGLL5	Immunogl	Protein Co	31	GC22P024	4.73
2578	ADCY1	Adenylate	Protein Co	48	GC07P045	4.73

2579	COL9A1	Collagen T	Protein Co	43	GC06M070	4.73
2580	TBL1XR1	TBL1X Rec	Protein Co	44	GC03M177	4.73
2581	GNAI3	G Protein S	Protein Co	46	GC01P109	4.73
2582	HAVCR2	Hepatitis A	Protein Co	43	GC05M157	4.72
2583	ITPR3	Inositol 1,4	Protein Co	45	GC06P033	4.72
2584	DCLK1	Doublecor	Protein Co	43	GC13M035	4.72
2585	MFGE8	Milk Fat Gl	Protein Co	44	GC15M088	4.72
2586	NFIX	Nuclear Fa	Protein Co	42	GC19P012	4.72
2587	CCT3	Chaperonii	Protein Co	41	GC01M156	4.71
2588	CCDC47	Coiled-Coi	Protein Co	35	GC17M063	4.71
2589	ILK	Integrin Li	Protein Co	46	GC11P006	4.71
2590	RPS6	Ribosomal	Protein Co	44	GC09M019	4.71
2591	JUND	JunD Protc	Protein Co	41	GC19M018	4.71
2592	CS	Citrate Syr	Protein Co	44	GC12M056	4.7
2593	DLK1	Delta Like	Protein Co	44	GC14P104	4.7
2594	CPLX1	Complexin	Protein Co	43	GC04M000	4.69
2595	SPEG	Striated M	Protein Co	41	GC02P219	4.69
2596	FOXD3	Forkhead I	Protein Co	40	GC01P063	4.69
2597	CACNA1S	Calcium Vo	Protein Co	48	GC01M201	4.69
2598	ABCE1	ATP Bindir	Protein Co	38	GC04P145	4.69
2599	GGCT	Gamma-Gl	Protein Co	39	GC07M030	4.68
2600	EVC	EvC Ciliary	Protein Co	38	GC04P005	4.68
2601	NAV2	Neuron Na	Protein Co	37	GC11P019	4.68
2602	B4GALT1	Beta-1,4-G	Protein Co	44	GC09M033	4.68
2603	HERC2	HECT And	Protein Co	45	GC15M028	4.68
2604	SHPK	Sedoheptu	Protein Co	38	GC17M003	4.68
2605	LAT2	Linker For	Protein Co	39	GC07P074	4.68
2606	STAG2	Stromal Ar	Protein Co	44	GC0XP123	4.67
2607	PGK1	Phosphogl	Protein Co	48	GC0XP077	4.67
2608	GIP	Gastric Inh	Protein Co	39	GC17M048	4.67
2609	ASPH	Aspartate I	Protein Co	42	GC08M061	4.67
2610	IGFBP5	Insulin Like	Protein Co	43	GC02M216	4.67
2611	IGFBP7	Insulin Like	Protein Co	45	GC04M057	4.67
2612	CENPB	Centromer	Protein Co	37	GC20M003	4.67
2613	SYVN1	Synoviolin	Protein Co	40	GC11M065	4.66
2614	MIAT	Myocardia	RNA Gene	23	GC22P026	4.66
2615	PTP4A1	Protein Ty	Protein Co	41	GC06P063	4.66
2616	COL14A1	Collagen T	Protein Co	42	GC08P120	4.66
2617	ARF1	ADP Ribos	Protein Co	46	GC01P228	4.66
2618	MYOC	Myocilin	Protein Co	42	GC01M171	4.65
2619	PMEL	Premelano	Protein Co	37	GC12M055	4.65
2620	PEX26	Peroxisom	Protein Co	41	GC22P018	4.65
2621	FMO3	Flavin Con	Protein Co	46	GC01P171	4.65

2622	HARS1	Histidyl-TR	Protein Co	36	GC05M140	4.65
2623	ASIC1	Acid Sensi	Protein Co	43	GC12P050	4.65
2624	ERO1A	Endoplasm	Protein Co	35	GC14M052	4.65
2625	MBNL1	Muscleblin	Protein Co	39	GC03P152	4.65
2626	TFR2	Transferrin	Protein Co	44	GC07M100	4.65
2627	ONECUT1	One Cut H	Protein Co	40	GC15M060	4.65
2628	TELO2	Telomere I	Protein Co	40	GC16P001	4.64
2629	UTS2	Urotensin	Protein Co	41	GC01M007	4.64
2630	MIR128-2	MicroRNA	RNA Gene	20	GC03P035	4.64
2631	ALKBH5	AlkB Hom	Protein Co	33	GC17P018	4.64
2632	SETD1B	SET Domai	Protein Co	35	GC12P122	4.64
2633	COPA	COPI Coat	Protein Co	41	GC01M160	4.64
2634	NCSTN	Nicastrin	Protein Co	47	GC01P160	4.63
2635	CD244	CD244 Mo	Protein Co	43	GC01M160	4.63
2636	VCAN	Versican	Protein Co	47	GC05P083	4.63
2637	HTR2B	5-Hydroxy	Protein Co	43	GC02M231	4.63
2638	FGG	Fibrinogen	Protein Co	48	GC04M154	4.63
2639	NCS1	Neuronal (	Protein Co	41	GC09P130	4.63
2640	ATP6V1A	ATPase H+	Protein Co	44	GC03P113	4.63
2641	AHNAK	AHNAK Nu	Protein Co	36	GC11M063	4.63
2642	CRTC1	CREB Regu	Protein Co	42	GC19P023	4.62
2643	RIN2	Ras And R	Protein Co	41	GC20P019	4.62
2644	TACR2	Tachykinin	Protein Co	43	GC10M069	4.62
2645	EWSR1	EWS RNA	Protein Co	43	GC22P029	4.62
2646	FCER2	Fc Fragme	Protein Co	45	GC19M007	4.62
2647	MYH9	Myosin He	Protein Co	49	GC22M036	4.61
2648	CD1A	CD1a Mole	Protein Co	41	GC01P158	4.61
2649	NR5A2	Nuclear Re	Protein Co	45	GC01P199	4.61
2650	SLC35C1	Solute Car	Protein Co	40	GC11P045	4.61
2651	ATOH7	Atonal BHI	Protein Co	39	GC10M068	4.61
2652	DEPDC5	DEP Doma	Protein Co	40	GC22P031	4.61
2653	GYPB	Glycophori	Protein Co	37	GC04M143	4.61
2654	DEL16P13.	Chromoso	Genetic Lo	2	GC16U901	4.61
2655	DEL1P36	Chromoso	Genetic Lo	2	GC01U902	4.61
2656	FAM187A	Family Wit	Protein Co	23	GC17P044	4.61
2657	PTF1A	Pancreas A	Protein Co	40	GC10P023	4.6
2658	ZIC2	Zic Family	Protein Co	42	GC13P099	4.6
2659	F11R	F11 Recep	Protein Co	43	GC01M160	4.6
2660	XPO1	Exportin 1	Protein Co	45	GC02M061	4.6
2661	MIR518A1	MicroRNA	RNA Gene	16	GC19P054	4.6
2662	RAD51B	RAD51 Par	Protein Co	37	GC14P067	4.6
2663	SEC31A	SEC31 Hor	Protein Co	42	GC04M082	4.6
2664	SLC15A2	Solute Car	Protein Co	39	GC03P121	4.6

2665	SMPD1	Sphingomy	Protein Co		47	GC11P006	4.59
2666	NINJ1	Ninjurin 1	Protein Co		39	GC09M093	4.59
2667	AIF1	Allograft Ir	Protein Co		39	GC06P047	4.59
2668	SOX3	SRY-Box Tı	Protein Co		44	GC0XM140	4.59
2669	ADORA3	Adenosine	Protein Co		45	GC01M111	4.59
2670	MEST	Mesoderm	Protein Co		39	GC07P130	4.59
2671	CDKN2C	Cyclin Dep	Protein Co		45	GC01P050	4.59
2672	DERL1	Derlin 1	Protein Co		36	GC08M123	4.58
2673	TUBA1B	Tubulin Al	Protein Co		42	GC12M049	4.58
2674	GNE	Glucosamii	Protein Co		43	GC09M036	4.58
2675	BLM	BLM RecQ	Protein Co		48	GC15P090	4.57
2676	HOTAIRM1	HOXA Trar	RNA Gene		18	GC07P027	4.57
2677	CYSLTR2	Cysteinyl L	Protein Co		47	GC13P048	4.57
2678	ARPC4	Actin Relat	Protein Co		38	GC03P009	4.57
2679	FGGY	FGGY Carb	Protein Co		37	GC01P059	4.57
2680	DSG3	Desmoglei	Protein Co		38	GC18P031	4.57
2681	OLR1	Oxidized L	Protein Co		44	GC12M013	4.56
2682	HABP2	Hyaluronai	Protein Co		44	GC10P113	4.56
2683	RCAN1	Regulator	Protein Co		43	GC21M034	4.56
2684	OPRL1	Opioid Rel	Protein Co		45	GC20P064	4.56
2685	KLK4	Kallikrein F	Protein Co		42	GC19M050	4.56
2686	GZMM	Granzyme	Protein Co		39	GC19P000	4.56
2687	SLC25A3	Solute Car	Protein Co		45	GC12P098	4.55
2688	KCNJ5	Potassium	Protein Co		47	GC11P128	4.55
2689	KL	Klotho	Protein Co		44	GC13P033	4.55
2690	MESP2	Mesoderm	Protein Co		35	GC15P089	4.55
2691	LRRC8A	Leucine Ri	Protein Co		40	GC09P128	4.55
2692	CD2	CD2 Mole	Protein Co		44	GC01P116	4.55
2693	IFT20	Intraflagell	Protein Co		36	GC17M029	4.55
2694	DDB2	Damage S	Protein Co		45	GC11P047	4.55
2695	PLOD2	Procollage	Protein Co		45	GC03M146	4.55
2696	ASPA	Aspartoacy	Protein Co		44	GC17P003	4.55
2697	TNNT2	Troponin T	Protein Co		48	GC01M201	4.55
2698	NLGN1	Neuroligin	Protein Co		44	GC03P173	4.54
2699	HDAC2	Histone De	Protein Co		51	GC06M113	4.54
2700	BAP1	BRCA1 Ass	Protein Co		45	GC03M052	4.54
2701	PTGER2	Prostaglan	Protein Co		48	GC14P052	4.54
2702	HVCN1	Hydrogen	Protein Co		39	GC12M110	4.54
2703	AP1B1	Adaptor R	Protein Co		44	GC22M029	4.53
2704	AKR1C2	Aldo-Keto	Protein Co		47	GC10M004	4.53
2705	CRBN	Cereblon	Protein Co		43	GC03M003	4.53
2706	PTAFR	Platelet Ac	Protein Co		43	GC01M028	4.52
2707	SAR1A	Secretion A	Protein Co		41	GC10M070	4.52

2708	P2RY2	Purinergic	Protein Co	45	GC11P073	4.52
2709	BTC	Betacellulin	Protein Co	44	GC04M074	4.52
2710	MMUT	Methylmal	Protein Co	35	GC06M049	4.52
2711	GNLY	Granulysin	Protein Co	38	GC02P085	4.52
2712	TCF21	Transcripti	Protein Co	36	GC06P133	4.52
2713	SUGCT	Succinyl-C	Protein Co	36	GC07P040	4.51
2714	EIF2S1	Eukaryotic	Protein Co	44	GC14P067	4.51
2715	BCS1L	BCS1 Hom	Protein Co	43	GC02P218	4.51
2716	PIGQ	Phosphatic	Protein Co	42	GC16P001	4.51
2717	ALDOB	Aldolase, F	Protein Co	45	GC09M101	4.5
2718	GPR89B	G Protein-l	Protein Co	32	GC01P147	4.5
2719	ENPP2	Ectonuclec	Protein Co	44	GC08M119	4.5
2720	TDRG1	Testis Dev	RNA Gene	22	GC06P047	4.5
2721	NEFM	Neurofilam	Protein Co	42	GC08P024	4.5
2722	FREM1	FRAS1 Rel	Protein Co	41	GC09M014	4.5
2723	ZNF148	Zinc Finge	Protein Co	40	GC03M125	4.5
2724	CHD2	Chromodo	Protein Co	43	GC15P092	4.5
2725	LAMC1	Laminin Su	Protein Co	43	GC01P182	4.49
2726	CD81	CD81 Mol	Protein Co	47	GC11P002	4.49
2727	PNP	Purine Nu	Protein Co	47	GC14P020	4.49
2728	SURF1	SURF1 Cyt	Protein Co	43	GC09M133	4.49
2729	SUMF1	Sulfatase M	Protein Co	43	GC03M003	4.49
2730	POMT1	Protein O-	Protein Co	45	GC09P131	4.49
2731	CFD	Compleme	Protein Co	44	GC19P000	4.49
2732	MN1	MN1 Prot	Protein Co	41	GC22M027	4.49
2733	AKR1D1	Aldo-Keto	Protein Co	46	GC07P138	4.49
2734	ATP2A2	ATPase Sa	Protein Co	51	GC12P110	4.48
2735	HK1	Hexokinas	Protein Co	51	GC10P069	4.48
2736	UPK3A	Uroplakin	Protein Co	37	GC22P045	4.48
2737	DYNC1I2	Dynein Cyt	Protein Co	38	GC02P171	4.48
2738	IFIH1	Interferon	Protein Co	47	GC02M162	4.48
2739	COL6A2	Collagen T	Protein Co	43	GC21P046	4.47
2740	STAR	Steroidoge	Protein Co	46	GC08M038	4.47
2741	TCF12	Transcripti	Protein Co	47	GC15P056	4.47
2742	BAG1	BAG Coch	Protein Co	43	GC09M033	4.47
2743	TCF20	Transcripti	Protein Co	37	GC22M042	4.47
2744	PSMA1	Proteasom	Protein Co	44	GC11M014	4.46
2745	JPH3	Junctophili	Protein Co	41	GC16P087	4.46
2746	RXRB	Retinoid X	Protein Co	47	GC06M033	4.46
2747	EMD	Emerin	Protein Co	45	GC0XP154	4.46
2748	ITGB6	Integrin Su	Protein Co	47	GC02M160	4.46
2749	GPX1	Glutathion	Protein Co	48	GC03M049	4.46
2750	GABBR1	Gamma-A	Protein Co	47	GC06M029	4.46

2751	NELFCD	Negative E	Protein Co	35	GC20P058	4.46
2752	WDR81	WD Repea	Protein Co	36	GC17P001	4.46
2753	ZBTB20	Zinc Finge	Protein Co	40	GC03M114	4.46
2754	TAGLN	Transgelin	Protein Co	43	GC11P117	4.46
2755	IGLL1	Immunogl	Protein Co	43	GC22M023	4.46
2756	H4C8	H4 Cluster	Protein Co	31	GC06M027	4.45
2757	AOC3	Amine Oxi	Protein Co	44	GC17P042	4.45
2758	KCNA1	Potassium	Protein Co	44	GC12P008	4.45
2759	ST3GAL5	ST3 Beta-C	Protein Co	48	GC02M085	4.45
2760	TREM2	Triggering	Protein Co	43	GC06M042	4.45
2761	B3GALNT2	Beta-1,3-N	Protein Co	40	GC01M235	4.44
2762	SQSTM1	Sequestos	Protein Co	48	GC05P179	4.44
2763	SLC39A1	Solute Car	Protein Co	39	GC01M153	4.44
2764	ATP2B1	ATPase Pla	Protein Co	44	GC12M089	4.44
2765	POMGNT1	Protein O-	Protein Co	45	GC01M046	4.44
2766	PLOD3	Procollage	Protein Co	45	GC07M101	4.44
2767	TRAPPC10	Trafficking	Protein Co	37	GC21P044	4.44
2768	NAALADL2	N-Acetylat	Protein Co	36	GC03P174	4.43
2769	SAT1	Spermidin	Protein Co	45	GC0XP023	4.43
2770	GPR89A	G Protein-	Protein Co	32	GC01P145	4.43
2771	NPL	N-Acetyln	Protein Co	39	GC01P182	4.43
2772	C1QB	Compleme	Protein Co	44	GC01P022	4.43
2773	HSP90B1	Heat Shoc	Protein Co	45	GC12P103	4.43
2774	DHRS9	Dehydroge	Protein Co	39	GC02P169	4.42
2775	MIR204	MicroRNA	RNA Gene	21	GC09M070	4.42
2776	LGALS9	Galectin 9	Protein Co	39	GC17P027	4.42
2777	CSTB	Cystatin B	Protein Co	45	GC21M043	4.42
2778	MRRF	Mitochond	Protein Co	39	GC09P122	4.42
2779	SEC61A1	SEC61 Tra	Protein Co	41	GC03P128	4.42
2780	GLP1R	Glucagon I	Protein Co	46	GC06P039	4.42
2781	SCARB2	Scavenger	Protein Co	44	GC04M076	4.42
2782	KIF5B	Kinesin Fa	Protein Co	43	GC10M032	4.42
2783	ABO	ABO, Alph	Protein Co	37	GC09M133	4.42
2784	CYP2A6	Cytochrom	Protein Co	47	GC19M040	4.42
2785	HES7	Hes Family	Protein Co	37	GC17M008	4.41
2786	AIP	Aryl Hydro	Protein Co	44	GC11P067	4.41
2787	PPP3CA	Protein Ph	Protein Co	52	GC04M101	4.41
2788	NEFL	Neurofilam	Protein Co	46	GC08M024	4.41
2789	MKKS	McKusick-I	Protein Co	39	GC20M010	4.41
2790	BBS4	Bardet-Bie	Protein Co	41	GC15P072	4.41
2791	TPK1	Thiamin Py	Protein Co	46	GC07M144	4.4
2792	GP5	Glycoprote	Protein Co	40	GC03M194	4.4
2793	RO60	Ro60, Y RN	Protein Co	31	GC01P193	4.4

2794	NEDD4L	NEDD4 Lik	Protein Co	46	GC18P058	4.4
2795	MARCKS	Myristoyla	Protein Co	39	GC06P113	4.4
2796	DDX39B	DExD-Box	Protein Co	39	GC06M031	4.4
2797	CPT1B	Carnitine F	Protein Co	44	GC22M050	4.39
2798	NTF4	Neurotrop	Protein Co	44	GC19M049	4.39
2799	FBXL7	F-Box And	Protein Co	35	GC05P015	4.39
2800	CLOCK	Clock Circa	Protein Co	43	GC04M055	4.39
2801	TCTN3	Tectonic Fa	Protein Co	40	GC10M095	4.39
2802	APOD	Apolipopro	Protein Co	43	GC03M195	4.39
2803	GPSM2	G Protein S	Protein Co	41	GC01P108	4.39
2804	ADRA2A	Adrenocep	Protein Co	46	GC10P111	4.39
2805	SCNN1A	Sodium Ch	Protein Co	47	GC12M006	4.38
2806	DNAJC5	DnaJ Heat	Protein Co	43	GC20P063	4.38
2807	USB1	U6 SnRNA	Protein Co	36	GC16P057	4.38
2808	CCL22	C-C Motif	Protein Co	38	GC16P057	4.38
2809	PDXK	Pyridoxal k	Protein Co	47	GC21P043	4.37
2810	PRX	Periaxin	Protein Co	39	GC19M040	4.37
2811	CYP24A1	Cytochrom	Protein Co	47	GC20M054	4.37
2812	PNMA2	PNMA Fan	Protein Co	36	GC08M026	4.37
2813	PABPN1	Poly(A) Bin	Protein Co	44	GC14P025	4.37
2814	GSTT1	Glutathion	Protein Co	32	GC22Mi00	4.37
2815	HTR3E	5-Hydroxy	Protein Co	36	GC03P184	4.37
2816	PSENEN	Presenilin	Protein Co	44	GC19P038	4.37
2817	SEMA3E	Semaphori	Protein Co	41	GC07M083	4.37
2818	DEGS1	Delta 4-De	Protein Co	40	GC01P224	4.36
2819	PEX6	Peroxisom	Protein Co	43	GC06M042	4.36
2820	TFAP2A	Transcripti	Protein Co	47	GC06M010	4.35
2821	HOXA1	Homeobox	Protein Co	44	GC07M027	4.35
2822	CAMTA1	Calmodulir	Protein Co	40	GC01P006	4.35
2823	DSC2	Desmocoll	Protein Co	45	GC18M031	4.35
2824	CXCL17	C-X-C Mot	Protein Co	33	GC19M042	4.35
2825	AP4M1	Adaptor R	Protein Co	39	GC07P100	4.35
2826	TUBB2B	Tubulin Be	Protein Co	44	GC06M003	4.34
2827	GHSR	Growth Ho	Protein Co	47	GC03M172	4.34
2828	SPATA5	Spermatog	Protein Co	40	GC04P122	4.34
2829	LTB4R	Leukotrien	Protein Co	44	GC14P024	4.34
2830	MIR135B	MicroRNA	RNA Gene	19	GC01M205	4.34
2831	HAND2	Heart And	Protein Co	43	GC04M173	4.34
2832	SUN2	Sad1 And	Protein Co	37	GC22M045	4.33
2833	RER1	Retention	Protein Co	35	GC01P002	4.33
2834	COX4I1	Cytochrom	Protein Co	44	GC16P085	4.33
2835	FBLN1	Fibulin 1	Protein Co	43	GC22P045	4.33
2836	WDR5	WD Repea	Protein Co	44	GC09P134	4.33

2837	KIAA0319L	KIAA0319	Protein Co	38	GC01M035	4.33
2838	GAD1	Glutamate	Protein Co	51	GC02P170	4.33
2839	SHKBP1	SH3KBP1 B	Protein Co	36	GC19P040	4.33
2840	ITIH4	Inter-Alpha	Protein Co	42	GC03M052	4.33
2841	SOX18	SRY-Box Tı	Protein Co	40	GC20M064	4.33
2842	CELF2	CUGBP Ela	Protein Co	41	GC10P010	4.32
2843	MAT1A	Methionin	Protein Co	47	GC10M080	4.32
2844	MIR582	MicroRNA	RNA Gene	17	GC05M059	4.32
2845	DSC1	Desmocoll	Protein Co	38	GC18M031	4.32
2846	SLCO2B1	Solute Car	Protein Co	44	GC11P075	4.32
2847	HAPLN1	Hyaluronai	Protein Co	42	GC05M083	4.32
2848	MCM5	Minichrom	Protein Co	44	GC22P035	4.32
2849	ADRA1A	Adrenocep	Protein Co	47	GC08M026	4.31
2850	PSMB1	Proteasom	Protein Co	43	GC06M170	4.31
2851	TFPI	Tissue Fact	Protein Co	44	GC02M187	4.31
2852	PDSS2	Decapreny	Protein Co	40	GC06M107	4.31
2853	HLCS	Holocarbo	Protein Co	43	GC21M036	4.31
2854	COL9A3	Collagen T	Protein Co	42	GC20P062	4.31
2855	HLA-DOA	Major Hist	Protein Co	40	GC06M033	4.3
2856	ATXN2	Ataxin 2	Protein Co	42	GC12M111	4.3
2857	NEK8	NIMA Rela	Protein Co	41	GC17P028	4.3
2858	ASAH1	N-Acylsphi	Protein Co	46	GC08M018	4.3
2859	SUCLG1	Succinate-(	Protein Co	45	GC02M084	4.3
2860	KAT2B	Lysine Ace	Protein Co	48	GC03P020	4.29
2861	LMO2	LIM Doma	Protein Co	42	GC11M033	4.29
2862	FOXC2	Forkhead B	Protein Co	44	GC16P086	4.29
2863	UPB1	Beta-Ureid	Protein Co	44	GC22P024	4.29
2864	SBF2	SET Bindin	Protein Co	40	GC11M009	4.29
2865	NDE1	NudE Neu	Protein Co	43	GC16P015	4.29
2866	TAOK3	TAO Kinas	Protein Co	40	GC12M118	4.28
2867	GBA3	Glucosylce	Protein Co	36	GC04P022	4.28
2868	SMAD6	SMAD Fam	Protein Co	46	GC15P066	4.28
2869	MS4A1	Membrane	Protein Co	46	GC11P060	4.28
2870	SLC6A20	Solute Car	Protein Co	41	GC03M045	4.28
2871	SFRP1	Secreted F	Protein Co	43	GC08M041	4.28
2872	LAT	Linker For	Protein Co	45	GC16P029	4.28
2873	EHMT2	Euchromat	Protein Co	45	GC06M031	4.27
2874	RBBP5	RB Binding	Protein Co	39	GC01M205	4.27
2875	NAA50	N-Alpha-A	Protein Co	37	GC03M113	4.27
2876	KDELR1	KDEL Endc	Protein Co	39	GC19M048	4.27
2877	NDST1	N-Deacety	Protein Co	45	GC05P150	4.27
2878	ZC4H2	Zinc Finge	Protein Co	37	GC0XM064	4.27
2879	H4C9	H4 Cluster	Protein Co	31	GC06P028	4.27

2880	RAB39B	RAB39B, M	Protein Co	39	GC0XM155	4.27
2881	PRKAA2	Protein Kir	Protein Co	50	GC01P056	4.26
2882	IFNL3	Interferon	Protein Co	35	GC19M039	4.26
2883	SRM	Spermidine	Protein Co	41	GC01M011	4.26
2884	XPNPEP1	X-Prolyl Ar	Protein Co	42	GC10M109	4.26
2885	LHX3	LIM Home	Protein Co	41	GC09M136	4.26
2886	MTA1	Metastasis	Protein Co	42	GC14P105	4.26
2887	PFKM	Phosphofru	Protein Co	50	GC12P048	4.26
2888	PC	Pyruvate C	Protein Co	47	GC11M066	4.26
2889	VNN1	Vanin 1	Protein Co	44	GC06M132	4.25
2890	ALMS1	ALMS1 Cer	Protein Co	41	GC02P073	4.25
2891	CYSLTR1	Cysteinyl L	Protein Co	43	GC0XM078	4.25
2892	ARNTL	Aryl Hydro	Protein Co	42	GC11P013	4.25
2893	ASXL3	ASXL Trans	Protein Co	35	GC18P033	4.25
2894	EFHC2	EF-Hand D	Protein Co	35	GC0XM044	4.25
2895	ART1	ADP-Ribos	Protein Co	35	GC11P003	4.24
2896	PEX1	Peroxisom	Protein Co	44	GC07M092	4.24
2897	NISCH	Nischarin	Protein Co	41	GC03P052	4.24
2898	STK39	Serine/Thr	Protein Co	44	GC02M167	4.23
2899	ABCA12	ATP Bindir	Protein Co	42	GC02M214	4.23
2900	PSMC2	Proteasom	Protein Co	40	GC07P103	4.23
2901	BAMBI	BMP And A	Protein Co	43	GC10P028	4.23
2902	EVPL	Envoplakin	Protein Co	35	GC17M076	4.22
2903	HES5	Hes Family	Protein Co	34	GC01M002	4.22
2904	NFIB	Nuclear Fa	Protein Co	43	GC09M014	4.22
2905	SP7	Sp7 Transc	Protein Co	41	GC12M053	4.22
2906	PEX3	Peroxisom	Protein Co	42	GC06P143	4.22
2907	SLC5A4	Solute Car	Protein Co	38	GC22M032	4.21
2908	BPIFB1	BPI Fold C	Protein Co	33	GC20P033	4.21
2909	KEL	Kell Metall	Protein Co	42	GC07M142	4.21
2910	PIWIL4	Piwi Like R	Protein Co	37	GC11P094	4.21
2911	HECW2	HECT, C2 A	Protein Co	41	GC02M196	4.21
2912	CASZ1	Castor Zin	Protein Co	36	GC01M010	4.21
2913	H4C15	H4 Cluster	Protein Co	27	GC01M149	4.2
2914	CAMK2G	Calcium/C	Protein Co	47	GC10M073	4.2
2915	SKP2	S-Phase Ki	Protein Co	44	GC05P036	4.19
2916	NTSR1	Neurotens	Protein Co	42	GC20P062	4.19
2917	FCGR2B	Fc Fragme	Protein Co	47	GC01P161	4.19
2918	BBS2	Bardet-Bie	Protein Co	41	GC16M056	4.19
2919	CRIP2	Cysteine R	Protein Co	36	GC14P105	4.19
2920	ARRB1	Arrestin Be	Protein Co	43	GC11M075	4.18
2921	EMX2	Empty Spir	Protein Co	43	GC10P117	4.18
2922	ALDH1A1	Aldehyde I	Protein Co	47	GC09M072	4.18

2923	C1QA	Compleme	Protein Co	45	GC01P022	4.18
2924	KCNV2	Potassium	Protein Co	40	GC09P002	4.18
2925	SHF	Src Homol	Protein Co	35	GC15M045	4.18
2926	ELOVL1	ELOVL Fatt	Protein Co	41	GC01M043	4.18
2927	PSMD14	Proteasom	Protein Co	43	GC02P161	4.18
2928	HRH1	Histamine	Protein Co	45	GC03P011	4.18
2929	FKBP1A	FKBP Proly	Protein Co	46	GC20M001	4.17
2930	NADSYN1	NAD Syntl	Protein Co	40	GC11P071	4.17
2931	BACE1-AS	BACE1 Ant	RNA Gene	13	GC11P117	4.17
2932	LFNG	LFNG O-Fu	Protein Co	46	GC07P002	4.17
2933	ADH1B	Alcohol De	Protein Co	43	GC04M099	4.17
2934	TSHB	Thyroid Sti	Protein Co	42	GC01P115	4.17
2935	TRAPPC11	Trafficking	Protein Co	36	GC04P183	4.17
2936	HTR1B	5-Hydroxy	Protein Co	45	GC06M077	4.17
2937	SGO1-AS1	SGO1 Anti	RNA Gene	13	GC03P020	4.16
2938	NEB	Nebulin	Protein Co	41	GC02M151	4.16
2939	FADD	Fas Associ	Protein Co	47	GC11P070	4.16
2940	FOXO1	Forkhead I	Protein Co	48	GC13M040	4.16
2941	ZFYVE26	Zinc Finge	Protein Co	39	GC14M067	4.16
2942	MED9	Mediator (	Protein Co	34	GC17P017	4.16
2943	SLC22A1	Solute Car	Protein Co	43	GC06P160	4.16
2944	ALG8	ALG8 Alph	Protein Co	44	GC11M078	4.16
2945	HLA-DRB5	Major Hist	Protein Co	40	GC06M032	4.16
2946	TXNRD2	Thioredoxi	Protein Co	46	GC22M019	4.16
2947	CD52	CD52 Mole	Protein Co	36	GC01P026	4.16
2948	PDPK1	3-Phospho	Protein Co	48	GC16P002	4.15
2949	RPA1	Replicatior	Protein Co	45	GC17P001	4.15
2950	UGCG	UDP-Glucc	Protein Co	43	GC09P111	4.15
2951	CDKAL1	CDK5 Regu	Protein Co	39	GC06P020	4.15
2952	PPAN	Peter Pan	Protein Co	33	GC19P010	4.15
2953	HAX1	HCLS1 Ass	Protein Co	43	GC01P154	4.15
2954	HIBCH	3-Hydroxy	Protein Co	43	GC02M190	4.15
2955	S100A10	S100 Calci	Protein Co	45	GC01M151	4.14
2956	PLP1	Proteolipic	Protein Co	43	GC0XP103	4.14
2957	IGFBP4	Insulin Like	Protein Co	43	GC17P040	4.14
2958	LY9	Lymphocyt	Protein Co	39	GC01P160	4.13
2959	ETFDH	Electron Tr	Protein Co	44	GC04P158	4.13
2960	HDAC3	Histone De	Protein Co	49	GC05M141	4.13
2961	GPR101	G Protein-(	Protein Co	39	GC0XM137	4.13
2962	UBE4B	Ubiquitina	Protein Co	40	GC01P010	4.13
2963	PCSK2	Proprotein	Protein Co	41	GC20P017	4.13
2964	MYH8	Myosin He	Protein Co	40	GC17M010	4.13
2965	ADCY3	Adenylate	Protein Co	47	GC02M024	4.13

2966	IVD	Isovaleryl-(	Protein Co	45	GC15P040	4.12
2967	NCOR1	Nuclear Re	Protein Co	43	GC17M016	4.12
2968	AQP2	Aquaporin	Protein Co	46	GC12P049	4.12
2969	BCL2L2	BCL2 Like	Protein Co	44	GC14P025	4.12
2970	LIFR	LIF Recept	Protein Co	47	GC05M038	4.11
2971	COQ8A	Coenzyme	Protein Co	36	GC01P226	4.11
2972	H4C11	H4 Cluster	Protein Co	30	GC06P028	4.11
2973	ID2	Inhibitor C	Protein Co	44	GC02P008	4.11
2974	FNIP1	Folliculin I	Protein Co	36	GC05M131	4.1
2975	FZD9	Frizzled Cl	Protein Co	44	GC07P073	4.1
2976	P2RX3	Purinergic	Protein Co	41	GC11P057	4.1
2977	NDUFB8	NADH:Ubi	Protein Co	43	GC10M100	4.1
2978	PAX5	Paired Box	Protein Co	46	GC09M036	4.1
2979	DAB2	DAB Adap	Protein Co	43	GC05M039	4.1
2980	FH	Fumarate I	Protein Co	45	GC01M241	4.1
2981	HLA-DRB3	Major Hist	Protein Co	28	GC06Mn03	4.1
2982	PARK7	Parkinsoni	Protein Co	45	GC01P007	4.1
2983	SLC13A5	Solute Car	Protein Co	42	GC17M006	4.09
2984	CCL13	C-C Motif	Protein Co	38	GC17P034	4.09
2985	DNAL1	Dynein Ax	Protein Co	41	GC14P073	4.09
2986	SCNN1G	Sodium Cl	Protein Co	46	GC16P023	4.09
2987	TIRAP	TIR Domai	Protein Co	43	GC11P126	4.09
2988	TMCO1	Transmem	Protein Co	40	GC01M165	4.09
2989	CASP6	Caspase 6	Protein Co	48	GC04M109	4.09
2990	PRKAA1	Protein Kir	Protein Co	47	GC05M040	4.09
2991	LAMA1	Laminin Su	Protein Co	45	GC18M006	4.09
2992	CXCL16	C-X-C Mot	Protein Co	40	GC17M004	4.09
2993	TTPA	Alpha Toc	Protein Co	41	GC08M063	4.08
2994	SPX	Spexin Ho	Protein Co	29	GC12P021	4.08
2995	CEACAM7	CEA Cell A	Protein Co	37	GC19M041	4.08
2996	ARFGEF2	ADP Ribos	Protein Co	41	GC20P048	4.08
2997	BFSP2	Beaded Fil	Protein Co	39	GC03P133	4.08
2998	PRDX5	Peroxiredo	Protein Co	46	GC11P064	4.08
2999	LPO	Lactoperox	Protein Co	39	GC17P058	4.07
3000	H4C3	H4 Cluster	Protein Co	31	GC06P028	4.07
3001	ARHGEF4	Rho Guani	Protein Co	40	GC02P130	4.07
3002	PPRC1	PPARG Rel	Protein Co	37	GC10P102	4.07
3003	STX5	Syntaxin 5	Protein Co	40	GC11M062	4.07
3004	SLC7A11	Solute Car	Protein Co	44	GC04M138	4.07
3005	OXTR	Oxytocin R	Protein Co	45	GC03M008	4.06
3006	CASTOR3	CASTOR F	Pseudoger	24	GC07M100	4.06
3007	S100A6	S100 Calci	Protein Co	43	GC01M153	4.06
3008	WASF2	WASP Fam	Protein Co	43	GC01M027	4.06

3009	CLEC4E	C-Type Le	Protein Co	37	GC12M008	4.06
3010	SLC25A6	Solute Car	Protein Co	43	GC0XM001	4.06
3011	HPSE2	Heparanas	Protein Co	41	GC10M098	4.06
3012	ARF3	ADP Ribos	Protein Co	40	GC12M048	4.05
3013	OSMR	Oncostatin	Protein Co	45	GC05P038	4.05
3014	DDIT4	DNA Dam	Protein Co	44	GC10P072	4.05
3015	CFAP65	Cilia And F	Protein Co	29	GC02M219	4.05
3016	NHEJ1	Non-Hom	Protein Co	41	GC02M219	4.05
3017	ORM1	Orosomuc	Protein Co	39	GC09P114	4.05
3018	KRT3	Keratin 3	Protein Co	39	GC12M052	4.05
3019	MADD	MAP Kinas	Protein Co	40	GC11P047	4.05
3020	AGAP2	ArfGAP Wi	Protein Co	41	GC12M057	4.04
3021	NRGN	Neurogran	Protein Co	39	GC11P124	4.04
3022	AQP9	Aquaporin	Protein Co	43	GC15P058	4.04
3023	ARL1	ADP Ribos	Protein Co	40	GC12M101	4.04
3024	ADAM12	ADAM Me	Protein Co	45	GC10M126	4.04
3025	HIVEP1	HIVEP Zinc	Protein Co	37	GC06P012	4.04
3026	ALS2	Alsin Rho (	Protein Co	44	GC02M201	4.04
3027	MPC1	Mitochonc	Protein Co	41	GC06M166	4.04
3028	WARS2	Tryptophai	Protein Co	44	GC01M119	4.04
3029	CPA1	Carboxype	Protein Co	45	GC07P130	4.04
3030	IRF2	Interferon	Protein Co	43	GC04M184	4.04
3031	TM2D1	TM2 Dom	Protein Co	33	GC01M061	4.04
3032	RPS20	Ribosomal	Protein Co	43	GC08M056	4.04
3033	LINC00538	Long Inter	RNA Gene	12	GC01P213	4.03
3034	LTBR	Lymphoto	Protein Co	41	GC12P006	4.03
3035	UCP2	Uncouplin	Protein Co	45	GC11M073	4.03
3036	CYP46A1	Cytochrom	Protein Co	41	GC14P099	4.03
3037	MIR92A1	MicroRNA	RNA Gene	19	GC13P091	4.03
3038	MSRB3	Methionin	Protein Co	43	GC12P065	4.03
3039	SPTA1	Spectrin A	Protein Co	43	GC01M158	4.03
3040	CA3	Carbonic A	Protein Co	41	GC08P085	4.03
3041	PACRG	Parkin Cor	Protein Co	37	GC06P162	4.03
3042	CELSR3	Cadherin E	Protein Co	39	GC03M048	4.02
3043	SLC31A1	Solute Car	Protein Co	42	GC09P113	4.02
3044	HSD3B1	Hydroxy-D	Protein Co	43	GC01P119	4.02
3045	CASP5	Caspase 5	Protein Co	44	GC11M104	4.02
3046	AURKB	Aurora Kin	Protein Co	48	GC17M008	4.02
3047	CCDC22	Coiled-Coi	Protein Co	39	GC0XP049	4.01
3048	NAT1	N-Acetyltra	Protein Co	44	GC08P018	4.01
3049	PDPN	Podoplanir	Protein Co	40	GC01P013	4.01
3050	ITGA1	Integrin Su	Protein Co	41	GC05P052	4.01
3051	FBXO42	F-Box Prot	Protein Co	33	GC01M016	4.01

3052	CD82	CD82 Mole	Protein Co	43	GC11P044	4.01
3053	ADK	Adenosine	Protein Co	50	GC10P074	4
3054	ABCG1	ATP Bindir	Protein Co	43	GC21P042	4
3055	BANF1	BAF Nucle	Protein Co	42	GC11P066	4
3056	KRT12	Keratin 12	Protein Co	39	GC17M040	4
3057	ARNT	Aryl Hydro	Protein Co	43	GC01M150	4
3058	CACNB1	Calcium V	Protein Co	42	GC17M039	4
3059	NUP62	Nucleopor	Protein Co	45	GC19M049	4
3060	MSI2	Musashi R	Protein Co	41	GC17P057	4
3061	NFIA	Nuclear Fa	Protein Co	44	GC01P060	3.99
3062	MUC13	Mucin 13,	Protein Co	36	GC03M124	3.99
3063	LRP8	LDL Recep	Protein Co	43	GC01M053	3.99
3064	TTN-AS1	TTN Antise	RNA Gene	15	GC02P178	3.99
3065	TTC3	Tetratricop	Protein Co	39	GC21P037	3.99
3066	MYO18B	Myosin XV	Protein Co	38	GC22P025	3.99
3067	EFNB1	Ephrin B1	Protein Co	47	GC0XP068	3.98
3068	MAN2B1	Mannosida	Protein Co	43	GC19M012	3.98
3069	TXN2	Thioredoxi	Protein Co	45	GC22M036	3.98
3070	SCNN1B	Sodium Ch	Protein Co	48	GC16P023	3.98
3071	SEC24D	SEC24 Hor	Protein Co	44	GC04M118	3.98
3072	ROCK2	Rho Assoc	Protein Co	47	GC02M011	3.98
3073	DEFB103B	Defensin B	Protein Co	30	GC08M007	3.98
3074	IRF2BP2	Interferon	Protein Co	36	GC01M234	3.98
3075	MAVS	Mitochond	Protein Co	40	GC20P003	3.98
3076	ATF2	Activating	Protein Co	45	GC02M175	3.97
3077	RPL34	Ribosomal	Protein Co	41	GC04P108	3.97
3078	PROP1	PROP Pair	Protein Co	40	GC05M177	3.97
3079	PHGDH	Phosphogl	Protein Co	49	GC01P119	3.97
3080	MYLK3	Myosin Lig	Protein Co	41	GC16M046	3.97
3081	MIR197	MicroRNA	RNA Gene	19	GC01P109	3.97
3082	RPS6KA5	Ribosomal	Protein Co	45	GC14M090	3.97
3083	HSD11B2	Hydroxyste	Protein Co	45	GC16P067	3.97
3084	PDCD10	Programm	Protein Co	42	GC03M167	3.97
3085	KIF3A	Kinesin Fa	Protein Co	41	GC05M132	3.96
3086	FGF9	Fibroblast	Protein Co	44	GC13P021	3.96
3087	COG7	Componer	Protein Co	39	GC16M023	3.96
3088	CRNDE	Colorectal	Protein Co	21	GC16M054	3.96
3089	GTPBP1	GTP Bindir	Protein Co	38	GC22P038	3.96
3090	GNAS-AS1	GNAS Anti	RNA Gene	22	GC20M058	3.96
3091	DLD	Dihydrolip	Protein Co	50	GC07P107	3.96
3092	MIR499A	MicroRNA	RNA Gene	21	GC20P034	3.96
3093	USH2A	Usherin	Protein Co	38	GC01M215	3.96
3094	SLC22A6	Solute Car	Protein Co	44	GC11M063	3.96

3095	TAP1	Transporte	Protein Co		47	GC06M032	3.96
3096	MC1R	Melanocor	Protein Co		47	GC16P089	3.96
3097	IL32	Interleukin	Protein Co		40	GC16P004	3.95
3098	OLIG2	Oligodend	Protein Co		40	GC21P033	3.95
3099	GATA5	GATA Bind	Protein Co		39	GC20M062	3.95
3100	MIR200A	MicroRNA	RNA Gene		21	GC01P001	3.95
3101	PTCHD1	Patched D	Protein Co		35	GC0XP023	3.95
3102	PSAP	Prosaposir	Protein Co		46	GC10M071	3.95
3103	GPX4	Glutathion	Protein Co		47	GC19P001	3.95
3104	STMN2	Stathmin 2	Protein Co		39	GC08P079	3.95
3105	CDK5	Cyclin Dep	Protein Co		52	GC07M151	3.95
3106	AGRN	Agrin	Protein Co		44	GC01P001	3.95
3107	SLC2A4	Solute Car	Protein Co		45	GC17P007	3.95
3108	NPHP4	Nephrocys	Protein Co		40	GC01M005	3.94
3109	EPHA4	EPH Recep	Protein Co		50	GC02M221	3.94
3110	H4C13	H4 Cluster	Protein Co		29	GC06M028	3.94
3111	KAT2A	Lysine Ace	Protein Co		47	GC17M042	3.94
3112	VAV1	Vav Guanii	Protein Co		47	GC19P006	3.94
3113	VPS33B	VPS33B La	Protein Co		41	GC15M090	3.94
3114	HNMT	Histamine	Protein Co		45	GC02P137	3.94
3115	PSMD5	Proteasom	Protein Co		38	GC09M120	3.94
3116	RPL10	Ribosomal	Protein Co		45	GC0XP154	3.94
3117	ELOVL6	ELOVL Fatt	Protein Co		40	GC04M110	3.94
3118	FGR	FGR Proto	Protein Co		48	GC01M027	3.94
3119	MDK	Midkine	Protein Co		43	GC11P046	3.94
3120	BLK	BLK Proto-	Protein Co		51	GC08P011	3.94
3121	SIM1	SIM BHLH	Protein Co		40	GC06M100	3.94
3122	XK	X-Linked K	Protein Co		40	GC0XP037	3.94
3123	CCL21	C-C Motif	Protein Co		43	GC09M034	3.94
3124	ATP1A2	ATPase Na	Protein Co		47	GC01P160	3.93
3125	LORICRIN	Loricrin Co	Protein Co		27	GC01P153	3.93
3126	MCU	Mitochond	Protein Co		36	GC10P072	3.93
3127	ADPRH	ADP-Ribos	Protein Co		37	GC03P119	3.93
3128	CASC3	CASC3 Exc	Protein Co		35	GC17P040	3.93
3129	SLC30A6	Solute Car	Protein Co		38	GC02P032	3.93
3130	MIR100	MicroRNA	RNA Gene		21	GC11M122	3.93
3131	CRYAB	Crystallin A	Protein Co		45	GC11M111	3.93
3132	RAG1	Recombina	Protein Co		45	GC11P036	3.93
3133	LDB3	LIM Doma	Protein Co		41	GC10P086	3.92
3134	CISH	Cytokine Ir	Protein Co		44	GC03M050	3.92
3135	LYST	Lysosomal	Protein Co		38	GC01M235	3.92
3136	FCN3	Ficolin 3	Protein Co		41	GC01M027	3.92
3137	CNR2	Cannabino	Protein Co		45	GC01M023	3.92

3138	CISD1	CDGSH Iro	Protein Co	38	GC10P058	3.92
3139	ADH1C	Alcohol De	Protein Co	42	GC04M099	3.92
3140	COL9A2	Collagen T	Protein Co	42	GC01M040	3.92
3141	COG5	Componer	Protein Co	39	GC07M107	3.92
3142	SGCE	Sarcoglyca	Protein Co	42	GC07M094	3.91
3143	ARF5	ADP Ribos	Protein Co	40	GC07P127	3.91
3144	SULT2A1	Sulfotransf	Protein Co	44	GC19M047	3.91
3145	CLDN18	Claudin 18	Protein Co	40	GC03P137	3.91
3146	PDIA4	Protein Dis	Protein Co	42	GC07M149	3.91
3147	UPK1A	Uroplakin	Protein Co	37	GC19P038	3.91
3148	CD24	CD24 Mole	Protein Co	33	GC06M106	3.91
3149	UGT1A	UDP Glucu	Uncategor	9	GC02P233	3.91
3150	IQCB1	IQ Motif C	Protein Co	41	GC03M121	3.9
3151	KRT4	Keratin 4	Protein Co	43	GC12M052	3.9
3152	AP1M2	Adaptor R	Protein Co	40	GC19M010	3.9
3153	MATN3	Matrilin 3	Protein Co	43	GC02M019	3.9
3154	TNFRSF10I	TNF Recep	Protein Co	43	GC08M023	3.9
3155	RNF113A	Ring Finge	Protein Co	37	GC0XM119	3.9
3156	RAPGEF3	Rap Guani	Protein Co	44	GC12M047	3.9
3157	ZBTB16	Zinc Finge	Protein Co	46	GC11P114	3.9
3158	HNRNPU	Heterogen	Protein Co	42	GC01M244	3.89
3159	SOX4	SRY-Box Tı	Protein Co	43	GC06P021	3.89
3160	CFAP126	Cilia And F	Protein Co	27	GC01M161	3.89
3161	CRYZL1	Crystallin Z	Protein Co	35	GC21M033	3.89
3162	FOXL2	Forkhead I	Protein Co	40	GC03M138	3.88
3163	CHRNA7	Cholinergi	Protein Co	45	GC15P031	3.88
3164	UGT1A9	UDP Glucu	Protein Co	43	GC02P233	3.88
3165	MOK	MOK Prote	Protein Co	35	GC14M102	3.88
3166	DIAPH1	Diaphanou	Protein Co	47	GC05M141	3.88
3167	S1PR1	Sphingosir	Protein Co	44	GC01P101	3.87
3168	AXL	AXL Recep	Protein Co	51	GC19P041	3.87
3169	KHK	Ketohexok	Protein Co	44	GC02P027	3.87
3170	GLB1	Galactosid	Protein Co	48	GC03M033	3.87
3171	AKT1S1	AKT1 Subs	Protein Co	40	GC19M049	3.87
3172	SMURF1	SMAD Spe	Protein Co	45	GC07M099	3.87
3173	JAZF1	JAZF Zinc I	Protein Co	39	GC07M027	3.87
3174	AQP3	Aquaporin	Protein Co	47	GC09M033	3.87
3175	TM2D3	TM2 Doma	Protein Co	33	GC15M101	3.87
3176	GPHN	Gephyrin	Protein Co	47	GC14P066	3.87
3177	UGT1A3	UDP Glucu	Protein Co	38	GC02P233	3.86
3178	SLC12A5	Solute Car	Protein Co	46	GC20P046	3.86
3179	ELOVL4	ELOVL Fatt	Protein Co	45	GC06M079	3.86
3180	PIEZO2	Piezo Type	Protein Co	36	GC18M010	3.86

3181	SLC1A5	Solute Car	Protein Co	43	GC19M046	3.86
3182	ATP2B4	ATPase Pla	Protein Co	44	GC01P203	3.86
3183	TERF2	Telomeric	Protein Co	40	GC16M069	3.85
3184	ITGA7	Integrin Su	Protein Co	47	GC12M055	3.85
3185	CSF1R	Colony Stii	Protein Co	52	GC05M150	3.85
3186	CHD8	Chromodo	Protein Co	41	GC14M021	3.85
3187	SC5D	Sterol-C5-I	Protein Co	40	GC11P121	3.85
3188	SYNGAP1	Synaptic R	Protein Co	43	GC06P033	3.85
3189	MMACHC	Metabolisr	Protein Co	43	GC01P045	3.85
3190	IL17B	Interleukin	Protein Co	41	GC05M149	3.85
3191	HTR6	5-Hydroxy	Protein Co	43	GC01P019	3.85
3192	TECPR1	Tectonin B	Protein Co	33	GC07M098	3.85
3193	TNFSF4	TNF Super	Protein Co	40	GC01M173	3.85
3194	PEX5	Peroxisom	Protein Co	42	GC12P008	3.85
3195	NIN	Ninein	Protein Co	41	GC14M050	3.85
3196	MIR23B	MicroRNA	RNA Gene	20	GC09P095	3.85
3197	ADRB3	Adrenocep	Protein Co	45	GC08M037	3.85
3198	IBSP	Integrin Bi	Protein Co	37	GC04P087	3.84
3199	ATP1A3	ATPase Na	Protein Co	47	GC19M041	3.84
3200	MAP3K14	Mitogen-A	Protein Co	44	GC17M045	3.84
3201	DCK	Deoxycytic	Protein Co	45	GC04P070	3.84
3202	CDC25C	Cell Divisic	Protein Co	48	GC05M138	3.84
3203	RAPGEF1	Rap Guani	Protein Co	42	GC09M131	3.83
3204	CD58	CD58 Mole	Protein Co	40	GC01M116	3.83
3205	FPGS	Folylpolygl	Protein Co	43	GC09P127	3.83
3206	IL27	Interleukin	Protein Co	38	GC16M028	3.83
3207	IL15RA	Interleukin	Protein Co	41	GC10M005	3.83
3208	GNAQ	G Protein S	Protein Co	49	GC09M077	3.83
3209	MT-TC	Mitochond	RNA Gene	10	GCMTM00	3.83
3210	ACKR3	Atypical Cl	Protein Co	40	GC02P236	3.83
3211	FAM111A	Family Wit	Protein Co	37	GC11P059	3.83
3212	NSD3	Nuclear Re	Protein Co	34	GC08M038	3.82
3213	PINK1	PTEN Indu	Protein Co	47	GC01P020	3.82
3214	SLC25A37	Solute Car	Protein Co	39	GC08P023	3.82
3215	LGR4	Leucine Ri	Protein Co	39	GC11M027	3.82
3216	PTPN1	Protein Ty	Protein Co	51	GC20P050	3.82
3217	DSTYK	Dual Serin	Protein Co	41	GC01M205	3.82
3218	IL17C	Interleukin	Protein Co	36	GC16P088	3.82
3219	FNIP2	Folliculin I	Protein Co	36	GC04P158	3.81
3220	H4C1	H4 Cluster	Protein Co	31	GC06P026	3.81
3221	MALRD1	MAM And	Protein Co	28	GC10P019	3.81
3222	NCR3	Natural Cy	Protein Co	39	GC06M031	3.81
3223	CYP2J2	Cytochrom	Protein Co	44	GC01M059	3.81

3224	IGHV4-38-	Immunogl	Protein Co	8	GC14U901	3.81
3225	TMPRSS2	Transmem	Protein Co	44	GC21M041	3.81
3226	DYNLT1	Dynein Lig	Protein Co	37	GC06M158	3.8
3227	H4C12	H4 Cluster	Protein Co	29	GC06M028	3.8
3228	MIR17HG	MiR-17-92	RNA Gene	28	GC13P091	3.8
3229	MAPKAPK2	MAPK Acti	Protein Co	48	GC01P206	3.8
3230	CPB1	Carboxype	Protein Co	42	GC03P148	3.8
3231	SLC28A2	Solute Car	Protein Co	42	GC15P045	3.8
3232	F2RL3	F2R Like Tl	Protein Co	45	GC19P016	3.79
3233	MYBPH	Myosin Bir	Protein Co	37	GC01M203	3.79
3234	DNA2	DNA Repli	Protein Co	41	GC10M068	3.79
3235	SYNM	Synemin	Protein Co	37	GC15P099	3.79
3236	CFAP47	Cilia And F	Protein Co	25	GC0XP035	3.79
3237	CACYBP	Calcyclin B	Protein Co	39	GC01P174	3.78
3238	STC1	Stanniocal	Protein Co	40	GC08M023	3.78
3239	EEA1	Early Endo	Protein Co	43	GC12M092	3.78
3240	NAB2	NGFI-A Bir	Protein Co	40	GC12P057	3.78
3241	FMOD	Fibromodu	Protein Co	40	GC01M203	3.78
3242	ROBO1	Roundabo	Protein Co	44	GC03M078	3.78
3243	CAPNS1	Calpain Sm	Protein Co	41	GC19P038	3.77
3244	PLCB3	Phospholip	Protein Co	47	GC11P064	3.77
3245	APOBEC1	Apolipopro	Protein Co	39	GC12M007	3.77
3246	CCT5	Chaperonii	Protein Co	43	GC05P010	3.77
3247	CFAP410	Cilia And F	Protein Co	29	GC21M044	3.77
3248	GLUD1	Glutamate	Protein Co	50	GC10M087	3.77
3249	MMP19	Matrix Met	Protein Co	47	GC12M055	3.77
3250	ERVMER34	Endogenou	Protein Co	29	GC04M052	3.77
3251	TIMM8A	Translocas	Protein Co	43	GC0XM101	3.77
3252	SRGAP2	SLIT-ROBC	Protein Co	36	GC01P206	3.77
3253	LIG3	DNA Ligas	Protein Co	44	GC17P034	3.76
3254	NR2F2	Nuclear Re	Protein Co	48	GC15P096	3.76
3255	KIF1C	Kinesin Fa	Protein Co	42	GC17P004	3.76
3256	LAPTM4A	Lysosomal	Protein Co	38	GC02M020	3.76
3257	TNNT1	Troponin T	Protein Co	43	GC19M055	3.76
3258	COLQ	Collagen L	Protein Co	39	GC03M015	3.76
3259	UROD	Uroporphy	Protein Co	45	GC01P045	3.76
3260	B4GALNT2	Beta-1,4-N	Protein Co	40	GC17P049	3.76
3261	ALOX15	Arachidon	Protein Co	44	GC17M004	3.75
3262	CTU2	Cytosolic T	Protein Co	33	GC16P088	3.75
3263	AGPAT2	1-Acylglyc	Protein Co	45	GC09M136	3.75
3264	TPD52	Tumor Pro	Protein Co	40	GC08M080	3.75
3265	COQ9	Coenzyme	Protein Co	41	GC16P057	3.75
3266	PROX1	Prospero H	Protein Co	42	GC01P213	3.75

3267	NTNG2	Netrin G2	Protein Co	40	GC09P132	3.74
3268	BNIP3	BCL2 Inter	Protein Co	42	GC10M131	3.74
3269	PRSS57	Serine Pro	Protein Co	31	GC19M000	3.74
3270	PHLPP2	PH Domai	Protein Co	41	GC16M071	3.74
3271	ACAD9	Acyl-CoA [	Protein Co	43	GC03P130	3.74
3272	CAPN9	Calpain 9	Protein Co	42	GC01P230	3.74
3273	OGDH	Oxoglutara	Protein Co	45	GC07P044	3.74
3274	SRPX2	Sushi Repe	Protein Co	40	GC0XP100	3.73
3275	MIR424	MicroRNA	RNA Gene	17	GC0XM134	3.73
3276	TJP3	Tight Junc	Protein Co	38	GC19P003	3.73
3277	SUMO4	Small Ubic	Protein Co	38	GC06P149	3.73
3278	FPR1	Formyl Pe	Protein Co	47	GC19M051	3.73
3279	H4C2	H4 Cluster	Protein Co	31	GC06M026	3.73
3280	GCDH	Glutaryl-C	Protein Co	47	GC19P012	3.73
3281	PTPN6	Protein Ty	Protein Co	50	GC12P008	3.72
3282	AGBL4	ATP/GTP B	Protein Co	33	GC01M048	3.72
3283	DCLK3	Doublecor	Protein Co	37	GC03M036	3.72
3284	KLRG1	Killer Cell I	Protein Co	37	GC12P008	3.72
3285	SNHG1	Small Nucl	RNA Gene	21	GC11M063	3.72
3286	POLR1H	RNA Polyn	Protein Co	31	GC06P048	3.72
3287	MX1	MX Dynam	Protein Co	41	GC21P041	3.72
3288	SLC52A2	Solute Car	Protein Co	37	GC08P144	3.72
3289	GLYATL1	Glycine-N-	Protein Co	35	GC11P058	3.72
3290	NEUROD2	Neuronal [	Protein Co	41	GC17M039	3.71
3291	WNT2	Wnt Famil	Protein Co	44	GC07M117	3.71
3292	EXOSC10	Exosome C	Protein Co	39	GC01M011	3.71
3293	DLGAP1	DLG Assoc	Protein Co	43	GC18M003	3.71
3294	CBR3-AS1	CBR3 Antis	RNA Gene	16	GC21M036	3.7
3295	CRK	CRK Proto	Protein Co	45	GC17M001	3.7
3296	CD247	CD247 Mo	Protein Co	49	GC01M167	3.7
3297	VTI1B	Vesicle Tra	Protein Co	42	GC14M067	3.7
3298	RAB35	RAB35, Me	Protein Co	41	GC12M120	3.7
3299	H4C14	H4 Cluster	Protein Co	28	GC01P149	3.7
3300	STK25	Serine/Thr	Protein Co	40	GC02M241	3.7
3301	ARRB2	Arrestin Be	Protein Co	44	GC17P004	3.7
3302	PAM16	Presequen	Protein Co	36	GC16M004	3.69
3303	RHOV	Ras Homo	Protein Co	33	GC15M040	3.69
3304	HSD11B1	Hydroxyste	Protein Co	49	GC01P209	3.69
3305	LTB	Lymphoto	Protein Co	40	GC06M032	3.69
3306	KRT16	Keratin 16	Protein Co	44	GC17M041	3.69
3307	ACR	Acrosin	Protein Co	38	GC22P050	3.69
3308	AQP10	Aquaporin	Protein Co	36	GC01P154	3.69
3309	PSPN	Persephin	Protein Co	39	GC19M006	3.69

3310	VANGL2	VANGL Pla	Protein Co	43	GC01P160	3.69
3311	PLEKHF1	Pleckstrin I	Protein Co	34	GC19P029	3.69
3312	S100P	S100 Calci	Protein Co	40	GC04P006	3.68
3313	CDK12	Cyclin Dep	Protein Co	40	GC17P039	3.68
3314	P2RY1	Purinergic	Protein Co	45	GC03P152	3.68
3315	EFNA2	Ephrin A2	Protein Co	41	GC19P001	3.68
3316	YES1	YES Proto-	Protein Co	48	GC18M000	3.68
3317	ITGA9	Integrin Su	Protein Co	43	GC03P037	3.68
3318	TGOLN2	Trans-Golg	Protein Co	37	GC02M085	3.68
3319	SLC5A11	Solute Car	Protein Co	40	GC16P024	3.67
3320	CENPE	Centromer	Protein Co	44	GC04M103	3.67
3321	DNMT3L	DNA Meth	Protein Co	41	GC21M044	3.67
3322	PSMC4	Proteasom	Protein Co	40	GC19P039	3.67
3323	ATL1	Atlastin GT	Protein Co	41	GC14P050	3.67
3324	ACVR1	Activin A F	Protein Co	51	GC02M157	3.67
3325	IL2RG	Interleukin	Protein Co	48	GC0XM071	3.66
3326	H4C4	H4 Cluster	Protein Co	28	GC06M027	3.66
3327	H4C5	H4 Cluster	Protein Co	28	GC06P028	3.66
3328	PEMT	Phosphatic	Protein Co	41	GC17M017	3.66
3329	FAM13A	Family Wit	Protein Co	37	GC04M088	3.66
3330	ADCYAP1R	ADCYAP R	Protein Co	45	GC07P031	3.66
3331	MAS1L	MAS1 Prot	Protein Co	35	GC06M029	3.66
3332	P2RX4	Purinergic	Protein Co	43	GC12P122	3.66
3333	PERP	P53 Apopt	Protein Co	41	GC06M138	3.66
3334	IGF2R	Insulin Like	Protein Co	45	GC06P159	3.65
3335	ATP2A1	ATPase Sa	Protein Co	48	GC16P029	3.65
3336	PRMT1	Protein Arg	Protein Co	48	GC19P049	3.65
3337	SAA4	Serum Am	Protein Co	39	GC11M018	3.65
3338	WNK4	WNK Lysin	Protein Co	44	GC17P043	3.65
3339	FGFR4	Fibroblast	Protein Co	51	GC05P177	3.64
3340	ZNF469	Zinc Finge	Protein Co	34	GC16P088	3.64
3341	MIR193A	MicroRNA	RNA Gene	18	GC17P031	3.64
3342	FFAR2	Free Fatty	Protein Co	40	GC19P038	3.64
3343	ADD3	Adducin 3	Protein Co	41	GC10P109	3.64
3344	CRHR2	Corticotrop	Protein Co	42	GC07M030	3.63
3345	CD83	CD83 Mole	Protein Co	39	GC06P014	3.63
3346	GJB4	Gap Juncti	Protein Co	40	GC01P034	3.63
3347	EXOSC1	Exosome C	Protein Co	37	GC10M097	3.63
3348	MAP3K1	Mitogen-A	Protein Co	49	GC05P056	3.63
3349	TUBB4A	Tubulin Be	Protein Co	45	GC19M006	3.62
3350	RHOF	Ras Homo	Protein Co	37	GC12M121	3.62
3351	DLG1	Discs Larg	Protein Co	43	GC03M197	3.62
3352	COL4A2	Collagen T	Protein Co	44	GC13P110	3.62

3353	GJA5	Gap Juncti	Protein Co	45	GC01M147	3.62
3354	COX6B1	Cytochrom	Protein Co	43	GC19P038	3.62
3355	ATP13A2	ATPase Ca	Protein Co	42	GC01M016	3.62
3356	MED25	Mediator (	Protein Co	39	GC19P049	3.62
3357	HSD17B1	Hydroxyste	Protein Co	43	GC17P042	3.62
3358	TSHZ1	Teashirt Zi	Protein Co	40	GC18P075	3.62
3359	LAMA5	Laminin Su	Protein Co	42	GC20M062	3.61
3360	ANGPTL4	Angiopoiet	Protein Co	45	GC19P008	3.61
3361	LARS1	Leucyl-TRN	Protein Co	36	GC05M146	3.61
3362	GRPR	Gastrin Rel	Protein Co	42	GC0XP016	3.61
3363	TEKT3	Tektin 3	Protein Co	35	GC17M015	3.61
3364	VHL	Von Hippe	Protein Co	47	GC03P010	3.61
3365	HSPB8	Heat Shocl	Protein Co	44	GC12P119	3.61
3366	CMAS	Cytidine M	Protein Co	38	GC12P022	3.6
3367	ACO1	Aconitase	Protein Co	44	GC09P032	3.6
3368	GORASP1	Golgi Reas	Protein Co	40	GC03M039	3.6
3369	MUC20	Mucin 20,	Protein Co	37	GC03P195	3.6
3370	SLC22A18	Solute Car	Protein Co	42	GC11P002	3.6
3371	FAAH	Fatty Acid	Protein Co	47	GC01P046	3.6
3372	TIMM13	Translocas	Protein Co	36	GC19M002	3.59
3373	SAA2	Serum Am	Protein Co	36	GC11M018	3.59
3374	ALDOA	Aldolase, F	Protein Co	48	GC16P030	3.59
3375	SP110	SP110 Nuc	Protein Co	40	GC02M230	3.59
3376	MIR301A	MicroRNA	RNA Gene	18	GC17M059	3.59
3377	CHRM1	Cholinergi	Protein Co	45	GC11M063	3.59
3378	SFTPA1	Surfactant	Protein Co	41	GC10P084	3.59
3379	PRKCI	Protein Kir	Protein Co	48	GC03P170	3.59
3380	CGB5	Chorionic (	Protein Co	32	GC19P049	3.59
3381	MLST8	MTOR Ass	Protein Co	42	GC16P002	3.59
3382	CADM1	Cell Adhes	Protein Co	43	GC11M115	3.59
3383	SLCO1B1	Solute Car	Protein Co	48	GC12P021	3.58
3384	ALCAM	Activated I	Protein Co	42	GC03P105	3.58
3385	ATRN	Attractin	Protein Co	36	GC20P003	3.58
3386	SLC26A5	Solute Car	Protein Co	43	GC07M103	3.58
3387	PAFAH1B1	Platelet Ac	Protein Co	45	GC17P002	3.58
3388	MIR654	MicroRNA	RNA Gene	17	GC14P104	3.58
3389	PAPPA2	Pappalysin	Protein Co	37	GC01P176	3.57
3390	SPON1	Spondin 1	Protein Co	36	GC11P013	3.57
3391	PAX2	Paired Box	Protein Co	46	GC10P100	3.57
3392	PROK1	Prokinetici	Protein Co	36	GC01P110	3.57
3393	EIF2AK2	Eukaryotic	Protein Co	45	GC02M037	3.57
3394	ACADM	Acyl-CoA I	Protein Co	47	GC01P075	3.57
3395	AMPD1	Adenosine	Protein Co	45	GC01M114	3.57

3396	FOSL2	FOS Like 2	Protein Co	41	GC02P028	3.57
3397	GAS1	Growth Ar	Protein Co	41	GC09M086	3.57
3398	CEP152	Centrosom	Protein Co	39	GC15M048	3.57
3399	TAP2	Transporte	Protein Co	44	GC06M032	3.57
3400	VAPA	VAMP Ass	Protein Co	43	GC18P009	3.56
3401	INF2	Inverted F	Protein Co	40	GC14P104	3.56
3402	CCM2	CCM2 Scal	Protein Co	42	GC07P044	3.56
3403	PRKCE	Protein Kir	Protein Co	50	GC02P045	3.56
3404	HTR1D	5-Hydroxy	Protein Co	45	GC01M023	3.56
3405	CARS1	Cysteinyl-T	Protein Co	32	GC11M003	3.56
3406	ANKS6	Ankyrin Re	Protein Co	38	GC09M098	3.56
3407	SOCS2	Suppresso	Protein Co	43	GC12P093	3.55
3408	INSRR	Insulin Rec	Protein Co	42	GC01M156	3.55
3409	H4C6	H4 Cluster	Protein Co	29	GC06P028	3.55
3410	HNRNPH2	Heterogen	Protein Co	37	GC0XP101	3.54
3411	MAPRE1	Microtubul	Protein Co	44	GC20P032	3.54
3412	SMARCA5	SWI/SNF R	Protein Co	40	GC04P143	3.54
3413	B9D2	B9 Domair	Protein Co	40	GC19M041	3.54
3414	AP2M1	Adaptor R	Protein Co	43	GC03P184	3.54
3415	SPTLC3	Serine Palr	Protein Co	40	GC20P013	3.53
3416	PNOC	Prepronoci	Protein Co	37	GC08P028	3.53
3417	XRCC3	X-Ray Rep	Protein Co	41	GC14M103	3.53
3418	PRG4	Proteoglyc	Protein Co	39	GC01P186	3.53
3419	ANXA8	Annexin A	Protein Co	35	GC10M047	3.53
3420	CPZ	Carboxype	Protein Co	39	GC04P008	3.53
3421	DLGAP3	DLG Assoc	Protein Co	37	GC01M034	3.53
3422	LDHA	Lactate De	Protein Co	51	GC11P018	3.52
3423	CERS1	Ceramide S	Protein Co	41	GC19M018	3.52
3424	IL13RA2	Interleukin	Protein Co	41	GC0XM115	3.52
3425	POU1F1	POU Class	Protein Co	43	GC03M087	3.51
3426	HOXA13	Homeobox	Protein Co	43	GC07M027	3.51
3427	IL3RA	Interleukin	Protein Co	42	GC0XP001	3.51
3428	TMEM237	Transmem	Protein Co	36	GC02M201	3.51
3429	MACROD2	Mono-ADF	Protein Co	35	GC20P013	3.51
3430	COX6C	Cytochrom	Protein Co	39	GC08M099	3.51
3431	C19orf12	Chromoso	Protein Co	36	GC19M029	3.51
3432	BDKRB1	Bradykinin	Protein Co	43	GC14P096	3.51
3433	HCP5	HLA Comp	RNA Gene	29	GC06P031	3.51
3434	JAG2	Jagged Ca	Protein Co	42	GC14M105	3.51
3435	GRK2	G Protein-(	Protein Co	37	GC11P067	3.51
3436	LHX4	LIM Home	Protein Co	41	GC01P180	3.5
3437	PRPH	Peripherin	Protein Co	44	GC12P049	3.5
3438	ASIC5	Acid Sensi	Protein Co	33	GC04M155	3.5

3439	AMELX	Amelogeni	Protein Co	36	GC0XP011	3.5
3440	CEACAM3	CEA Cell A	Protein Co	40	GC19P041	3.5
3441	ASAH2	N-Acylsphi	Protein Co	41	GC10M050	3.5
3442	MSTN	Myostatin	Protein Co	45	GC02M190	3.49
3443	NFATC2	Nuclear Fa	Protein Co	45	GC20M051	3.49
3444	WDR73	WD Repea	Protein Co	36	GC15M084	3.49
3445	TUBB3	Tubulin Be	Protein Co	49	GC16P089	3.49
3446	SLC4A11	Solute Car	Protein Co	40	GC20M003	3.49
3447	CLPB	Caseinolyti	Protein Co	43	GC11M072	3.49
3448	PTRH2	Peptidyl-TI	Protein Co	44	GC17M059	3.49
3449	GDA	Guanine D	Protein Co	43	GC09P072	3.48
3450	ACOT13	Acyl-CoA T	Protein Co	37	GC06P024	3.48
3451	MPDZ	Multiple PI	Protein Co	44	GC09M013	3.48
3452	TSPAN7	Tetraspanii	Protein Co	42	GC0XP038	3.48
3453	COQ2	Coenzyme	Protein Co	41	GC04M083	3.48
3454	NNAT	Neuronatir	Protein Co	32	GC20P037	3.48
3455	PDLIM1	PDZ And L	Protein Co	40	GC10M095	3.48
3456	RORC	RAR Relate	Protein Co	45	GC01M151	3.48
3457	GDF11	Growth Dif	Protein Co	40	GC12P055	3.48
3458	CLPX	Caseinolyti	Protein Co	39	GC15M065	3.48
3459	HESX1	HESX Hom	Protein Co	39	GC03M057	3.47
3460	DPYS	Dihydropyı	Protein Co	45	GC08M104	3.47
3461	MLKL	Mixed Line	Protein Co	41	GC16M074	3.47
3462	MAD2L2	Mitotic Arr	Protein Co	44	GC01M011	3.47
3463	GPR151	G Protein-(	Protein Co	34	GC05M146	3.47
3464	GHRHR	Growth Ho	Protein Co	43	GC07P030	3.47
3465	SHOX2	Short Statu	Protein Co	39	GC03M158	3.47
3466	FGF20	Fibroblast	Protein Co	40	GC08M016	3.47
3467	KRT9	Keratin 9	Protein Co	39	GC17M041	3.47
3468	KIR2DS4	Killer Cell I	Protein Co	32	GC19P055	3.47
3469	TRPM8	Transient F	Protein Co	43	GC02P233	3.47
3470	SMARCE1	SWI/SNF R	Protein Co	45	GC17M040	3.47
3471	AKR1B10	Aldo-Keto	Protein Co	43	GC07P134	3.46
3472	PDGFC	Platelet De	Protein Co	43	GC04M156	3.46
3473	ADIPOR2	Adiponecti	Protein Co	43	GC12P001	3.46
3474	PHF21A	PHD Finge	Protein Co	41	GC11M061	3.46
3475	STAT2	Signal Trar	Protein Co	47	GC12M056	3.46
3476	ST3GAL1	ST3 Beta-(	Protein Co	43	GC08M133	3.46
3477	FZD5	Frizzled Cl	Protein Co	47	GC02M207	3.46
3478	SLC27A5	Solute Car	Protein Co	43	GC19M058	3.46
3479	ENPEP	Glutamyl A	Protein Co	44	GC04P110	3.46
3480	ACADVL	Acyl-CoA I	Protein Co	45	GC17P007	3.46
3481	MEP1A	Meprin A S	Protein Co	42	GC06P046	3.46

3482	PDSS1	Decapreny	Protein Co	42	GC10P026	3.46
3483	SLC30A1	Solute Car	Protein Co	40	GC01M211	3.45
3484	MIR590	MicroRNA	RNA Gene	18	GC07P074	3.45
3485	PTGES	Prostaglan	Protein Co	41	GC09M129	3.45
3486	TERF1	Telomeric	Protein Co	41	GC08P073	3.45
3487	KCNJ10	Potassium	Protein Co	44	GC01M159	3.45
3488	NEUROD1	Neuronal [	Protein Co	44	GC02M181	3.45
3489	KIR2DL4	Killer Cell I	Protein Co	39	GC19P055	3.45
3490	IGF2BP2	Insulin Like	Protein Co	44	GC03M185	3.45
3491	TNFRSF17	TNF Recep	Protein Co	44	GC16P011	3.44
3492	KCNK5	Potassium	Protein Co	37	GC06M042	3.44
3493	CDC123	Cell Divisic	Protein Co	35	GC10P012	3.44
3494	ATF4	Activating	Protein Co	46	GC22P039	3.44
3495	NUS1	NUS1 Deh	Protein Co	39	GC06P117	3.44
3496	RARS2	Arginyl-TR	Protein Co	42	GC06M087	3.44
3497	HOGA1	4-Hydroxy	Protein Co	39	GC10P097	3.44
3498	SPHK1	Sphingosir	Protein Co	47	GC17P076	3.44
3499	GRHL3	Grainyhea	Protein Co	38	GC01P024	3.44
3500	PZP	PZP Alpha	Protein Co	39	GC12M009	3.44
3501	MTUS2	Microtubul	Protein Co	34	GC13P028	3.44
3502	CEP76	Centrosom	Protein Co	35	GC18M017	3.44
3503	FLT3LG	Fms Relate	Protein Co	40	GC19P049	3.44
3504	BANK1	B Cell Scaf	Protein Co	37	GC04P101	3.44
3505	MST1R	Macropha	Protein Co	48	GC03M049	3.44
3506	FREM3	FRAS1 Rel	Protein Co	31	GC04M143	3.44
3507	C5AR2	Compleme	Protein Co	39	GC19P047	3.43
3508	DPP6	Dipeptidyl	Protein Co	43	GC07P153	3.43
3509	MMP23B	Matrix Me	Protein Co	37	GC01P001	3.43
3510	ABCD1	ATP Bindir	Protein Co	45	GC0XP153	3.43
3511	CYTOR	Cytoskelet	RNA Gene	17	GC02P087	3.43
3512	MIR24-2	MicroRNA	RNA Gene	18	GC19M014	3.42
3513	WDR45	WD Repea	Protein Co	40	GC0XM049	3.42
3514	APEH	Acylamino	Protein Co	40	GC03P049	3.42
3515	ALX3	ALX Home	Protein Co	34	GC01M110	3.42
3516	WASHC4	WASH Cor	Protein Co	30	GC12P105	3.41
3517	CCL8	C-C Motif	Protein Co	39	GC17P034	3.41
3518	BLZF1	Basic Leuc	Protein Co	39	GC01P169	3.41
3519	CSN2	Casein Bet	Protein Co	36	GC04M069	3.41
3520	PKD2L1	Polycystin	Protein Co	41	GC10M100	3.41
3521	MCHR1	Melanin C	Protein Co	40	GC22P040	3.41
3522	LSS	Lanosterol	Protein Co	45	GC21M047	3.41
3523	SARM1	Sterile Alp	Protein Co	37	GC17P028	3.41
3524	NLGN3	Neuroligin	Protein Co	43	GC0XP071	3.41

3525	OXA1L	OXA1L Mit	Protein Co	39	GC14P022	3.41
3526	MIR363	MicroRNA	RNA Gene	15	GC0XM134	3.41
3527	APLNR	Apelin Rec	Protein Co	43	GC11M057	3.41
3528	AKAP12	A-Kinase A	Protein Co	40	GC06P151	3.41
3529	CHMP2B	Charged M	Protein Co	44	GC03P087	3.41
3530	DNAAF2	Dynein Axo	Protein Co	37	GC14M049	3.41
3531	SCG2	Secretogra	Protein Co	39	GC02M223	3.41
3532	NDP	Norrin Cys	Protein Co	43	GC0XM043	3.41
3533	NBEAL2	Neurobeac	Protein Co	40	GC03P046	3.41
3534	GLRX	Glutaredox	Protein Co	44	GC05M095	3.4
3535	EPHB4	EPH Recep	Protein Co	52	GC07M100	3.4
3536	EXTL3	Exostosin l	Protein Co	45	GC08P028	3.4
3537	FMN1	Formin 1	Protein Co	38	GC15M032	3.4
3538	SPINT1	Serine Pep	Protein Co	40	GC15P040	3.4
3539	CDON	Cell Adhes	Protein Co	45	GC11M125	3.39
3540	WLS	Wnt Ligan	Protein Co	39	GC01M068	3.39
3541	HADH	Hydroxyac	Protein Co	47	GC04P107	3.39
3542	SELENOP	Selenoprot	Protein Co	31	GC05M042	3.39
3543	TCEA1	Transcripti	Protein Co	37	GC08M053	3.39
3544	MIR637	MicroRNA	RNA Gene	16	GC19M003	3.39
3545	KANTR	KDM5C Ac	RNA Gene	17	GC0XP053	3.39
3546	DNAH10	Dynein Axo	Protein Co	35	GC12P123	3.38
3547	DUSP1	Dual Speci	Protein Co	47	GC05M172	3.38
3548	SFTPA2	Surfactant	Protein Co	40	GC10M079	3.38
3549	L2HGDH	L-2-Hydro	Protein Co	40	GC14M050	3.38
3550	GFI1B	Growth Fa	Protein Co	40	GC09P132	3.38
3551	LNPEP	Leucyl Anc	Protein Co	45	GC05P096	3.38
3552	GLE1	GLE1 RNA	Protein Co	40	GC09P128	3.38
3553	LBX1	Ladybird H	Protein Co	37	GC10M101	3.37
3554	BTG2	BTG Anti-P	Protein Co	37	GC01P203	3.37
3555	B3GAT1	Beta-1,3-G	Protein Co	43	GC11M134	3.37
3556	TRAP1	TNF Recep	Protein Co	43	GC16M003	3.37
3557	GANAB	Glucosidas	Protein Co	44	GC11M063	3.37
3558	IRF6	Interferon	Protein Co	43	GC01M209	3.37
3559	NLGN2	Neuroligin	Protein Co	40	GC17P007	3.37
3560	MIR103A1	MicroRNA	RNA Gene	17	GC05M168	3.37
3561	XRCC4	X-Ray Rep	Protein Co	43	GC05P083	3.37
3562	SP6	Sp6 Transc	Protein Co	33	GC17M047	3.37
3563	TSEN2	TRNA Splic	Protein Co	40	GC03P012	3.37
3564	SLC23A1	Solute Car	Protein Co	43	GC05M139	3.37
3565	CPOX	Coproporp	Protein Co	43	GC03M098	3.36
3566	CGN	Cingulin	Protein Co	39	GC01P151	3.36
3567	DAD1	Defender A	Protein Co	41	GC14M022	3.36

3568	CYP3A7	Cytochrom	Protein Co	43	GC07M099	3.36
3569	TMEM158	Transmem	Protein Co	32	GC03M045	3.36
3570	CHRM2	Cholinergi	Protein Co	48	GC07P136	3.36
3571	TFG	Trafficking	Protein Co	44	GC03P100	3.36
3572	ABAT	4-Aminobu	Protein Co	45	GC16P008	3.35
3573	CPQ	Carboxype	Protein Co	35	GC08P096	3.35
3574	FLNC	Filamin C	Protein Co	44	GC07P128	3.35
3575	DNAH1	Dynein Ax	Protein Co	37	GC03P052	3.35
3576	TRIO	Trio Rho G	Protein Co	45	GC05P014	3.35
3577	SLC26A4	Solute Car	Protein Co	43	GC07P107	3.35
3578	FHL1	Four And A	Protein Co	45	GC0XP136	3.35
3579	BCAR1	BCAR1 Sca	Protein Co	43	GC16M075	3.34
3580	SASS6	SAS-6 Cen	Protein Co	37	GC01M100	3.34
3581	GATM	Glycine An	Protein Co	45	GC15M045	3.34
3582	VAPB	VAMP Ass	Protein Co	45	GC20P058	3.34
3583	POU2F1	POU Class	Protein Co	44	GC01P167	3.34
3584	AGAP1	ArfGAP Wi	Protein Co	39	GC02P235	3.33
3585	PANK1	Pantothen	Protein Co	41	GC10M089	3.33
3586	AP5B1	Adaptor R	Protein Co	32	GC11M065	3.32
3587	SLC47A1	Solute Car	Protein Co	41	GC17P019	3.32
3588	HNF1A-AS	HNF1A An	RNA Gene	18	GC12M121	3.32
3589	SLC35A2	Solute Car	Protein Co	40	GC0XM048	3.32
3590	LPAR2	Lysophosp	Protein Co	44	GC19M019	3.32
3591	MBOAT2	Membrane	Protein Co	36	GC02M008	3.32
3592	PHC1	Polyhomec	Protein Co	40	GC12P008	3.32
3593	NFAT5	Nuclear Fa	Protein Co	42	GC16P069	3.32
3594	ITGA5	Integrin Su	Protein Co	48	GC12M054	3.32
3595	ANTXR1	ANTXR Cel	Protein Co	46	GC02P068	3.32
3596	CCL28	C-C Motif	Protein Co	40	GC05M043	3.31
3597	USP7	Ubiquitin S	Protein Co	47	GC16M008	3.31
3598	MAPK11	Mitogen-A	Protein Co	48	GC22M050	3.31
3599	SNAP29	Synaptoso	Protein Co	42	GC22P020	3.31
3600	GPX3	Glutathion	Protein Co	44	GC05P150	3.31
3601	APOL1	Apolipopro	Protein Co	42	GC22P036	3.31
3602	TLK2	Tousled Lil	Protein Co	45	GC17P062	3.31
3603	DNAJC3	DnaJ Heat	Protein Co	40	GC13P095	3.31
3604	PDCD4	Programm	Protein Co	43	GC10P110	3.31
3605	CDC73	Cell Divisic	Protein Co	44	GC01P193	3.3
3606	C1QC	Compleme	Protein Co	44	GC01P022	3.3
3607	RNASEH2C	Ribonuclea	Protein Co	39	GC11M065	3.3
3608	STING1	Stimulator	Protein Co	34	GC05M139	3.3
3609	NINJ2	Ninjurin 2	Protein Co	35	GC12M000	3.3
3610	NPY4R	Neuropept	Protein Co	35	GC10M046	3.3

3611	PEBP1	Phosphatic	Protein Co	44	GC12P118	3.3
3612	PGM1	Phosphogl	Protein Co	48	GC01P063	3.3
3613	CYLD	CYLD Lysir	Protein Co	48	GC16P050	3.3
3614	UBAC1	UBA Dom	Protein Co	36	GC09M135	3.3
3615	RPS11	Ribosomal	Protein Co	39	GC19P049	3.3
3616	MRI1	Methylthio	Protein Co	37	GC19P013	3.29
3617	POR	Cytochrom	Protein Co	48	GC07P075	3.29
3618	MNDA	Myeloid C	Protein Co	40	GC01P158	3.29
3619	AHSP	Alpha Hem	Protein Co	35	GC16P031	3.29
3620	MAP2K3	Mitogen-A	Protein Co	49	GC17P026	3.29
3621	HES4	Hes Family	Protein Co	31	GC01M001	3.29
3622	ADAM15	ADAM Me	Protein Co	41	GC01P155	3.28
3623	AMD1	Adenosylm	Protein Co	43	GC06P110	3.28
3624	SAMHD1	SAM And I	Protein Co	43	GC20M036	3.28
3625	MARK3	Microtubul	Protein Co	48	GC14P103	3.28
3626	COX10	Cytochrom	Protein Co	43	GC17P014	3.28
3627	ADAMTSL4	ADAMTS L	Protein Co	38	GC01P150	3.28
3628	PTOV1	PTOV1 Ext	Protein Co	36	GC19P049	3.28
3629	PSMA7	Proteasom	Protein Co	44	GC20M062	3.28
3630	ADTRP	Androgen	Protein Co	29	GC06M011	3.28
3631	SORL1	Sortilin Rel	Protein Co	43	GC11P121	3.28
3632	GSTA1	Glutathion	Protein Co	41	GC06M052	3.28
3633	SH2B1	SH2B Adap	Protein Co	43	GC16P029	3.27
3634	ADRA2B	Adrenocep	Protein Co	45	GC02M096	3.27
3635	RAP1B	RAP1B, Me	Protein Co	45	GC12P068	3.27
3636	ARHGAP15	Rho GTPas	Protein Co	40	GC02P143	3.27
3637	DNASE1L3	Deoxyribo	Protein Co	43	GC03M058	3.27
3638	ANK2	Ankyrin 2	Protein Co	41	GC04P112	3.27
3639	HCN1	Hyperpola	Protein Co	45	GC05M045	3.27
3640	F11-AS1	F11 Antise	RNA Gene	12	GC04M186	3.26
3641	TM7SF2	Transmem	Protein Co	41	GC11P065	3.26
3642	PRDM2	PR/SET Do	Protein Co	40	GC01P013	3.26
3643	LAMB2	Laminin Su	Protein Co	44	GC03M049	3.26
3644	HSPA9	Heat Shoc	Protein Co	47	GC05M138	3.26
3645	HSF1	Heat Shoc	Protein Co	44	GC08P144	3.26
3646	ROBO2	Roundabo	Protein Co	42	GC03P075	3.26
3647	SDHAF1	Succinate I	Protein Co	35	GC19P035	3.26
3648	TRAF3IP2	TRAF3 Inte	Protein Co	43	GC06M111	3.26
3649	MVB12A	Multivesicu	Protein Co	31	GC19P023	3.26
3650	LY6G5B	Lymphocyt	Protein Co	29	GC06P047	3.25
3651	KIF15	Kinesin Fa	Protein Co	36	GC03P045	3.25
3652	DNAI2	Dynein Axo	Protein Co	39	GC17P074	3.25
3653	FRAXA	Fragile Site	Biological	6	GC0XP147	3.25

3654	SRF	Serum Res	Protein Co	41	GC06P043	3.25
3655	ABCD4	ATP Bindir	Protein Co	43	GC14M074	3.25
3656	HLA-DMA	Major Hist	Protein Co	40	GC06M032	3.25
3657	SLC30A5	Solute Car	Protein Co	41	GC05P069	3.25
3658	CA4	Carbonic A	Protein Co	47	GC17P060	3.25
3659	MED17	Mediator (	Protein Co	40	GC11P093	3.25
3660	RHOC	Ras Homo	Protein Co	41	GC01M112	3.24
3661	FEN1	Flap Struct	Protein Co	46	GC11P061	3.24
3662	PAX8	Paired Box	Protein Co	46	GC02M113	3.24
3663	CTAG1B	Cancer/Tes	Protein Co	32	GC0XM154	3.24
3664	KCNJ13	Potassium	Protein Co	44	GC02M232	3.24
3665	NPRL3	NPR3 Like,	Protein Co	38	GC16M000	3.24
3666	FGF21	Fibroblast	Protein Co	40	GC19P048	3.24
3667	SCRIB	Scribble Pl	Protein Co	40	GC08M143	3.23
3668	JUNB	JunB Proto	Protein Co	41	GC19P012	3.23
3669	TUSC3	Tumor Sup	Protein Co	41	GC08P015	3.23
3670	SCIMP	SLP Adapt	Protein Co	31	GC17M005	3.23
3671	SLC25A13	Solute Car	Protein Co	46	GC07M096	3.23
3672	SORD	Sorbitol D	Protein Co	45	GC15P045	3.22
3673	MYORG	Myogenesi	Protein Co	27	GC09M034	3.22
3674	DEFA1	Defensin A	Protein Co	40	GC08M006	3.22
3675	P3H2	Prolyl 3-Hy	Protein Co	36	GC03M189	3.22
3676	NKX2-8	NK2 Home	Protein Co	37	GC14M036	3.22
3677	SNORA49	Small Nucl	RNA Gene	16	GC12P132	3.22
3678	TMPRSS4	Transmem	Protein Co	40	GC11P118	3.22
3679	NBAS	NBAS Subu	Protein Co	39	GC02M014	3.22
3680	POU3F1	POU Class	Protein Co	36	GC01M038	3.22
3681	PXK	PX Domain	Protein Co	38	GC03P058	3.22
3682	TNFSF14	TNF Super	Protein Co	41	GC19M006	3.22
3683	ATCAY	ATCAY Kin	Protein Co	38	GC19P003	3.22
3684	TUBA1A	Tubulin Al	Protein Co	48	GC12M049	3.21
3685	MT-ATP8	Mitochond	Protein Co	28	GCMTP008	3.21
3686	NRXN2	Neurexin 2	Protein Co	40	GC11M064	3.21
3687	CARD11	Caspase R	Protein Co	47	GC07M002	3.21
3688	CANT1	Calcium A	Protein Co	44	GC17M078	3.21
3689	CPT1A	Carnitine F	Protein Co	48	GC11M068	3.21
3690	SGPL1	Sphingosir	Protein Co	44	GC10P070	3.21
3691	SMPD4	Sphingomy	Protein Co	37	GC02M130	3.21
3692	GRIN2D	Glutamate	Protein Co	48	GC19P048	3.21
3693	RBMS2	RNA Bindi	Protein Co	36	GC12P056	3.21
3694	MT2A	Metallothi	Protein Co	42	GC16P056	3.21
3695	NUMA1	Nuclear M	Protein Co	43	GC11M072	3.21
3696	ALDH3A2	Aldehyde I	Protein Co	45	GC17P019	3.21

3697	C7	Compleme	Protein Co	42	GC05P040	3.2
3698	STMN1	Stathmin 1	Protein Co	44	GC01M025	3.2
3699	GLO1	Glyoxalase	Protein Co	45	GC06M042	3.2
3700	DIO3	Iodothyror	Protein Co	40	GC14P104	3.2
3701	PARVA	Parvin Alpl	Protein Co	44	GC11P012	3.2
3702	MGAT2	Alpha-1,6-	Protein Co	44	GC14P049	3.2
3703	CGB3	Chorionic (	Protein Co	31	GC19M049	3.2
3704	MFF	Mitochonc	Protein Co	37	GC02P227	3.19
3705	PLIN2	Perilipin 2	Protein Co	43	GC09M019	3.19
3706	SLC4A2	Solute Car	Protein Co	43	GC07P151	3.19
3707	MIR182	MicroRNA	RNA Gene	20	GC07M129	3.19
3708	CIZ1	CDKN1A Ir	Protein Co	37	GC09M128	3.19
3709	LPIN1	Lipin 1	Protein Co	48	GC02P011	3.19
3710	HSPH1	Heat Shocl	Protein Co	43	GC13M031	3.19
3711	KCNK2	Potassium	Protein Co	41	GC01P215	3.19
3712	CASP4	Caspase 4	Protein Co	46	GC11M104	3.18
3713	SCD	Stearoyl-C	Protein Co	48	GC10P100	3.18
3714	EPHB3	EPH Recep	Protein Co	44	GC03P184	3.18
3715	SIRPA	Signal Reg	Protein Co	43	GC20P001	3.18
3716	CSNK2A1	Casein Kin	Protein Co	51	GC20M000	3.18
3717	VSX1	Visual Syst	Protein Co	37	GC20M025	3.18
3718	PALM	Paralemmi	Protein Co	36	GC19P000	3.18
3719	THAP3	THAP Dom	Protein Co	35	GC01P006	3.18
3720	SFRP2	Secreted F	Protein Co	42	GC04M153	3.18
3721	ANGPTL3	Angiopoiet	Protein Co	45	GC01P062	3.18
3722	PLTP	Phospholip	Protein Co	43	GC20M045	3.18
3723	SNRPE	Small Nucl	Protein Co	41	GC01P203	3.18
3724	ITGB8	Integrin Su	Protein Co	41	GC07P020	3.18
3725	ATP2A3	ATPase Sa	Protein Co	44	GC17M003	3.18
3726	FABP5	Fatty Acid	Protein Co	41	GC08P081	3.17
3727	NDUFV1	NADH:Ubi	Protein Co	45	GC11P067	3.17
3728	SF3B1	Splicing Fa	Protein Co	44	GC02M197	3.17
3729	MSRA	Methionin	Protein Co	42	GC08P010	3.16
3730	LIPE	Lipase E, H	Protein Co	47	GC19M042	3.16
3731	KRT13	Keratin 13	Protein Co	43	GC17M041	3.16
3732	MIR29B2	MicroRNA	RNA Gene	18	GC01M207	3.16
3733	PCYT1A	Phosphate	Protein Co	46	GC03M196	3.16
3734	UBA5	Ubiquitin L	Protein Co	43	GC03P132	3.16
3735	SLCO1B3	Solute Car	Protein Co	44	GC12P020	3.16
3736	YY1	YY1 Transc	Protein Co	48	GC14P100	3.16
3737	PML	PML Nucle	Protein Co	45	GC15P073	3.16
3738	GALR1	Galanin Re	Protein Co	44	GC18P077	3.16
3739	DDR1	Discoidin I	Protein Co	47	GC06P047	3.16

3740	PON3	Paraoxona	Protein Co	44	GC07M095	3.16
3741	COX15	Cytochrom	Protein Co	42	GC10M099	3.16
3742	CYB5A	Cytochrom	Protein Co	44	GC18M074	3.15
3743	NEDD9	Neural Pre	Protein Co	42	GC06M011	3.15
3744	GPAM	Glycerol-3-	Protein Co	43	GC10M112	3.15
3745	CLCN1	Chloride V	Protein Co	44	GC07P143	3.15
3746	YWHAB	Tyrosine 3-	Protein Co	48	GC20P044	3.15
3747	PCK1	Phosphoer	Protein Co	48	GC20P057	3.15
3748	RACK1	Receptor F	Protein Co	37	GC05M181	3.15
3749	PRPS1	Phosphoril	Protein Co	46	GC0XP107	3.15
3750	AKR1C1	Aldo-Keto	Protein Co	43	GC10P004	3.15
3751	TRPC6	Transient F	Protein Co	48	GC11M101	3.15
3752	PLA2G10	Phospholip	Protein Co	44	GC16M014	3.15
3753	AGBL3	ATP/GTP B	Protein Co	34	GC07P134	3.14
3754	PLD1	Phospholip	Protein Co	48	GC03M171	3.14
3755	HES1	Hes Family	Protein Co	43	GC03P194	3.14
3756	CDH13	Cadherin 1	Protein Co	42	GC16P082	3.14
3757	JAM3	Junctional	Protein Co	43	GC11P134	3.14
3758	TSEN54	TRNA Splic	Protein Co	38	GC17P075	3.14
3759	PMP2	Peripheral	Protein Co	43	GC08M081	3.14
3760	MBD4	Methyl-Cp	Protein Co	42	GC03M129	3.14
3761	PLXND1	Plexin D1	Protein Co	40	GC03M129	3.14
3762	TNK1	Tyrosine K	Protein Co	41	GC17P007	3.13
3763	TGFBI	Transformi	Protein Co	45	GC05P136	3.13
3764	TNFAIP6	TNF Alpha	Protein Co	40	GC02P151	3.13
3765	SELENOS	Selenoprot	Protein Co	31	GC15M103	3.13
3766	PEG10	Paternally	Protein Co	40	GC07P094	3.13
3767	PAM	Peptidylgly	Protein Co	43	GC05P102	3.12
3768	DPYSL2	Dihydropyr	Protein Co	45	GC08P026	3.12
3769	PER2	Period Circ	Protein Co	43	GC02M238	3.12
3770	MATR3	Matrin 3	Protein Co	41	GC05P139	3.12
3771	TUBGCP2	Tubulin Ga	Protein Co	37	GC10M133	3.12
3772	TPM2	Tropomyos	Protein Co	44	GC09M035	3.11
3773	PPIA	Peptidylpro	Protein Co	47	GC07P044	3.11
3774	HSPE1	Heat Shock	Protein Co	40	GC02P197	3.11
3775	NEDD8	NEDD8 Ub	Protein Co	41	GC14M024	3.11
3776	COL12A1	Collagen T	Protein Co	41	GC06M075	3.11
3777	WDR6	WD Repea	Protein Co	35	GC03P049	3.11
3778	UQCRFS1	Ubiquinol-	Protein Co	45	GC19M029	3.11
3779	GCM2	Glial Cells	Protein Co	41	GC06M010	3.11
3780	PTPRS	Protein Tyr	Protein Co	43	GC19M005	3.11
3781	CSF2RA	Colony Stir	Protein Co	45	GC0XP001	3.11
3782	RPL7	Ribosomal	Protein Co	41	GC08M073	3.11

3783	DPP9	Dipeptidyl	Protein Co	40	GC19M00	3.1
3784	MMP26	Matrix Me	Protein Co	34	GC11P004	3.1
3785	ZBTB38	Zinc Finge	Protein Co	36	GC03P141	3.1
3786	SILC1	Sciatic Inju	RNA Gene	12	GC02P005	3.1
3787	ERCC3	ERCC Excis	Protein Co	47	GC02M127	3.1
3788	LGALS2	Galectin 2	Protein Co	41	GC22M037	3.1
3789	UNC80	Unc-80 Hc	Protein Co	33	GC02P209	3.1
3790	DNAAF4-C	DNAAF4-C	RNA Gene	9	GC15M060	3.1
3791	GSS	Glutathion	Protein Co	47	GC20M034	3.09
3792	LTBP3	Latent Trar	Protein Co	41	GC11M065	3.09
3793	TRAF3	TNF Recep	Protein Co	47	GC14P104	3.09
3794	MAP3K11	Mitogen-A	Protein Co	46	GC11M065	3.09
3795	ESX1	ESX Home	Protein Co	32	GC0XM104	3.09
3796	MSI1	Musashi R	Protein Co	39	GC12M120	3.09
3797	MTM1	Myotubula	Protein Co	44	GC0XP150	3.09
3798	MRPL49	Mitochond	Protein Co	36	GC11P065	3.09
3799	CD226	CD226 Mo	Protein Co	43	GC18M069	3.09
3800	RPS6KA1	Ribosomal	Protein Co	50	GC01P026	3.09
3801	PRKAG2	Protein Kir	Protein Co	50	GC07M151	3.09
3802	RPGR	Retinitis Pi	Protein Co	40	GC0XM038	3.08
3803	FOXA2	Forkhead I	Protein Co	45	GC20M022	3.08
3804	KCNK3	Potassium	Protein Co	49	GC02P026	3.08
3805	SCARNA6	Small Caja	RNA Gene	18	GC02P233	3.08
3806	LUZP1	Leucine Zi	Protein Co	34	GC01M023	3.08
3807	HMGA1	High Mobi	Protein Co	46	GC06P047	3.08
3808	DDX3X	DEAD-Box	Protein Co	47	GC0XP041	3.08
3809	LOXL1	Lysyl Oxid	Protein Co	43	GC15P073	3.08
3810	CD38	CD38 Mol	Protein Co	45	GC04P015	3.07
3811	LGMN	Legumain	Protein Co	42	GC14M092	3.07
3812	MRC1	Mannose I	Protein Co	39	GC10P017	3.07
3813	HEXB	Hexosamir	Protein Co	47	GC05P074	3.07
3814	NPHP3-AC	NPHP3-AC	RNA Gene	12	GC03M132	3.07
3815	GKN1	Gastrokine	Protein Co	38	GC02P068	3.07
3816	MERTK	MER Proto	Protein Co	50	GC02P111	3.07
3817	SOX6	SRY-Box Tı	Protein Co	42	GC11M015	3.07
3818	UGT1A7	UDP Glucu	Protein Co	39	GC02P233	3.07
3819	SUN1	Sad1 And	Protein Co	37	GC07P000	3.07
3820	PDIA2	Protein Dis	Protein Co	38	GC16P001	3.06
3821	FGF4	Fibroblast	Protein Co	44	GC11M069	3.06
3822	DUOX1	Dual Oxida	Protein Co	41	GC15P045	3.06
3823	TIE1	Tyrosine K	Protein Co	43	GC01P043	3.06
3824	HTT	Huntingtin	Protein Co	43	GC04P003	3.06
3825	RBM10	RNA Bindi	Protein Co	40	GC0XP047	3.06

3826	ACVR1B	Activin A F	Protein Co	47	GC12P051	3.06
3827	SIRT6	Sirtuin 6	Protein Co	45	GC19M004	3.06
3828	GUF1	GUF1 Hom	Protein Co	37	GC04P044	3.06
3829	RAB7B	RAB7B, Me	Protein Co	32	GC01M205	3.06
3830	MT3	Metallothi	Protein Co	40	GC16P056	3.06
3831	CAV2	Caveolin 2	Protein Co	42	GC07P116	3.06
3832	CCDC40	Coiled-Coi	Protein Co	37	GC17P080	3.05
3833	TOMM20	Translocas	Protein Co	39	GC01M235	3.05
3834	RPL5	Ribosomal	Protein Co	47	GC01P092	3.05
3835	PHB	Prohibitin	Protein Co	47	GC17M049	3.05
3836	PIAS1	Protein Inh	Protein Co	44	GC15P068	3.05
3837	NLGN4X	Neuroligin	Protein Co	40	GC0XM005	3.05
3838	REG1B	Regenerati	Protein Co	38	GC02M079	3.05
3839	AOX1	Aldehyde (	Protein Co	44	GC02P200	3.05
3840	ADAT3	Adenosine	Protein Co	35	GC19P001	3.05
3841	KCNJ1	Potassium	Protein Co	47	GC11M128	3.04
3842	TICAM1	Toll Like R	Protein Co	44	GC19M004	3.04
3843	MIR381	MicroRNA	RNA Gene	17	GC14P104	3.04
3844	DDR2	Discoidin [	Protein Co	51	GC01P162	3.04
3845	MAP3K6	Mitogen-A	Protein Co	44	GC01M027	3.04
3846	SIRT3	Sirtuin 3	Protein Co	48	GC11M000	3.03
3847	KCNN4	Potassium	Protein Co	48	GC19M043	3.03
3848	TMEM123	Transmem	Protein Co	33	GC11M102	3.03
3849	PEX2	Peroxisom	Protein Co	43	GC08M076	3.03
3850	H6PD	Hexose-6-I	Protein Co	41	GC01P009	3.03
3851	ERCC8	ERCC Excis	Protein Co	41	GC05M060	3.03
3852	LUC7L2	LUC7 Like	Protein Co	36	GC07P139	3.03
3853	NCOR2	Nuclear Re	Protein Co	43	GC12M124	3.03
3854	HSPA14	Heat Shoc	Protein Co	39	GC10P014	3.03
3855	KLRB1	Killer Cell I	Protein Co	41	GC12M013	3.03
3856	MIRLET7I	MicroRNA	RNA Gene	20	GC12P062	3.02
3857	ZMYND19	Zinc Finge	Protein Co	34	GC09M137	3.02
3858	MIR30C1	MicroRNA	RNA Gene	21	GC01P040	3.02
3859	TTF2	Transcripti	Protein Co	39	GC01P117	3.02
3860	PRKAB1	Protein Kir	Protein Co	47	GC12P119	3.02
3861	LGR6	Leucine Ri	Protein Co	41	GC01P202	3.02
3862	SKP1	S-Phase Ki	Protein Co	43	GC05M134	3.02
3863	SLC6A1	Solute Car	Protein Co	47	GC03P011	3.01
3864	MIR181B1	MicroRNA	RNA Gene	19	GC01M198	3.01
3865	CTNNA3	Catenin Al	Protein Co	39	GC10M065	3.01
3866	CD74	CD74 Mole	Protein Co	43	GC05M150	3.01
3867	PRDM6	PR/SET Do	Protein Co	39	GC05P123	3.01
3868	HCK	HCK Proto	Protein Co	49	GC20P032	3.01

3869	RSPH9	Radial Spo	Protein Co	37	GC06P047	3.01
3870	RRAS	RAS Relate	Protein Co	43	GC19M049	3.01
3871	FBP1	Fructose-B	Protein Co	50	GC09M094	3.01
3872	CREB5	CAMP Res	Protein Co	41	GC07P028	3.01
3873	PDE4D	Phosphodi	Protein Co	48	GC05M058	3
3874	STK4	Serine/Thr	Protein Co	48	GC20P044	3
3875	LOC11080	Solute Car	Biological	1	GC17P030	2.99
3876	ERLIN2	ER Lipid R	Protein Co	40	GC08P037	2.99
3877	CDSN	Corneodes	Protein Co	40	GC06M031	2.99
3878	IGLON5	IgLON Fan	Protein Co	33	GC19P051	2.99
3879	SLC39A7	Solute Car	Protein Co	41	GC06P033	2.99
3880	EPPK1	Epiplakin 1	Protein Co	34	GC08M143	2.99
3881	SERPIND1	Serpin Fan	Protein Co	45	GC22P020	2.99
3882	GPR39	G Protein-	Protein Co	40	GC02P132	2.99
3883	PTPRU	Protein Ty	Protein Co	41	GC01P029	2.99
3884	AIMP1	Aminoacyl	Protein Co	43	GC04P106	2.98
3885	MOG	Myelin Oli	Protein Co	45	GC06P047	2.98
3886	GGA3	Golgi Asso	Protein Co	39	GC17M075	2.98
3887	MIR181C	MicroRNA	RNA Gene	20	GC19P013	2.98
3888	MAPK8IP1	Mitogen-A	Protein Co	45	GC11P045	2.98
3889	CSPG5	Chondroiti	Protein Co	38	GC03M047	2.98
3890	PRORP	Protein On	Protein Co	27	GC14P035	2.98
3891	KCNK6	Potassium	Protein Co	39	GC19P038	2.98
3892	SRSF6	Serine Anc	Protein Co	39	GC20P043	2.98
3893	DPAGT1	Dolichyl-Pl	Protein Co	45	GC11M119	2.98
3894	DDOST	Dolichyl-D	Protein Co	45	GC01M020	2.98
3895	NCAPD3	Non-SMC	Protein Co	40	GC11M134	2.98
3896	PSEN2	Presenilin	Protein Co	49	GC01P226	2.98
3897	COPS8	COP9 Sign	Protein Co	37	GC02P237	2.98
3898	MAN1B1	Mannosida	Protein Co	46	GC09P137	2.98
3899	ABRA	Actin Bindi	Protein Co	33	GC08M106	2.98
3900	PHF13	PHD Finge	Protein Co	32	GC01P006	2.98
3901	OPA1	OPA1 Mitc	Protein Co	44	GC03P193	2.98
3902	SLC26A1	Solute Car	Protein Co	40	GC04M000	2.98
3903	IFIT3	Interferon	Protein Co	39	GC10P089	2.97
3904	SPAG1	Sperm Ass	Protein Co	39	GC08P100	2.97
3905	BCO1	Beta-Carot	Protein Co	36	GC16P081	2.97
3906	CERK	Ceramide I	Protein Co	42	GC22M046	2.97
3907	GTF2H3	General Tr	Protein Co	39	GC12P123	2.97
3908	RPS24	Ribosomal	Protein Co	43	GC10P078	2.97
3909	FUCA1	Alpha-L-Fu	Protein Co	47	GC01M023	2.97
3910	ARID2	AT-Rich In	Protein Co	41	GC12P045	2.97
3911	ITGB5	Integrin Su	Protein Co	45	GC03M124	2.96

3912	CSF3R	Colony Sti	Protein Co	47	GC01M036	2.96
3913	LRRFIP1	LRR Bindin	Protein Co	38	GC02P237	2.96
3914	CH25H	Cholestero	Protein Co	37	GC10M089	2.96
3915	RNASEH2A	Ribonuclea	Protein Co	43	GC19P012	2.95
3916	DNAH5	Dynein Axo	Protein Co	40	GC05M013	2.95
3917	MARS1	Methionyl-	Protein Co	35	GC12P057	2.95
3918	TRAPPC9	Trafficking	Protein Co	39	GC08M139	2.95
3919	LPAR3	Lysophosp	Protein Co	43	GC01M084	2.95
3920	SF1	Splicing Fa	Protein Co	38	GC11M064	2.95
3921	GOSR1	Golgi SNA	Protein Co	39	GC17P030	2.95
3922	CBR1	Carbonyl R	Protein Co	45	GC21P036	2.95
3923	RETREG3	Reticuloph	Protein Co	26	GC17M042	2.95
3924	PHACTR1	Phosphata	Protein Co	37	GC06P012	2.95
3925	CTF1	Cardiotrop	Protein Co	39	GC16P030	2.95
3926	UCN3	Urocortin 3	Protein Co	37	GC10P005	2.95
3927	COL6A1	Collagen T	Protein Co	43	GC21P045	2.94
3928	PSORS1C1	Psoriasis S	Protein Co	28	GC06P031	2.94
3929	TMEM138	Transmem	Protein Co	36	GC11P061	2.94
3930	PTGER1	Prostaglan	Protein Co	41	GC19M014	2.94
3931	PHEX	Phosphate	Protein Co	40	GC0XP022	2.94
3932	LONP1	Lon Peptid	Protein Co	43	GC19M005	2.94
3933	TMPRSS11	Transmem	Protein Co	37	GC04M067	2.94
3934	TUBB	Tubulin Be	Protein Co	49	GC06P030	2.94
3935	G6PC3	Glucose-6-	Protein Co	41	GC17P044	2.94
3936	HOMER1	Homer Sca	Protein Co	40	GC05M079	2.93
3937	CSNK2B	Casein Kin	Protein Co	47	GC06P047	2.93
3938	MARCKSL1	MARCKS L	Protein Co	37	GC01M032	2.93
3939	MIR99B	MicroRNA	RNA Gene	18	GC19P051	2.93
3940	DNAI1	Dynein Axo	Protein Co	41	GC09P034	2.93
3941	PAK3	P21 (RAC1	Protein Co	50	GC0XP110	2.93
3942	SLC5A5	Solute Car	Protein Co	45	GC19P023	2.93
3943	IL22RA1	Interleukin	Protein Co	39	GC01M024	2.93
3944	CARD10	Caspase Re	Protein Co	40	GC22M045	2.93
3945	MLX	MAX Dime	Protein Co	43	GC17P042	2.92
3946	NPS	Neuropept	Protein Co	32	GC10P127	2.92
3947	HBG2	Hemoglob	Protein Co	42	GC11M005	2.92
3948	CDK5RAP2	CDK5 Regu	Protein Co	42	GC09M120	2.92
3949	CUX2	Cut Like H	Protein Co	40	GC12P111	2.92
3950	ATL3	Atlastin GT	Protein Co	38	GC11M063	2.92
3951	CALM2	Calmodulin	Protein Co	44	GC02M047	2.92
3952	AP2S1	Adaptor Re	Protein Co	41	GC19M046	2.92
3953	ADCY5	Adenylate	Protein Co	48	GC03M123	2.92
3954	P2RY11	Purinergic	Protein Co	44	GC19P010	2.92

3955	JRK	Jrk Helix-T	Protein Co	36	GC08M142	2.92
3956	LEXM	Lymphocyt	Protein Co	24	GC01P054	2.91
3957	SMPD2	Sphingomy	Protein Co	40	GC06P109	2.91
3958	CLN5	CLN5 Intra	Protein Co	40	GC13P076	2.91
3959	IGH	Immunogl	Protein Co	15	GC14M109	2.91
3960	SNRPA	Small Nucl	Protein Co	40	GC19P040	2.91
3961	SHOC2	SHOC2 Le	Protein Co	41	GC10P110	2.91
3962	SCN2B	Sodium Vc	Protein Co	43	GC11M118	2.91
3963	CALML6	Calmodulir	Protein Co	32	GC01P001	2.91
3964	GABARAP	GABA Typ	Protein Co	44	GC17M007	2.91
3965	DUSP19	Dual Speci	Protein Co	39	GC02P183	2.9
3966	HBE1	Hemoglob	Protein Co	38	GC11M005	2.9
3967	HAR1B	Highly Acc	RNA Gene	17	GC20M063	2.9
3968	RHD	Rh Blood (	Protein Co	40	GC01P025	2.9
3969	LINC01194	Long Inter	RNA Gene	17	GC05P012	2.9
3970	PPP2CA	Protein Ph	Protein Co	48	GC05M134	2.9
3971	SPHK2	Sphingosir	Protein Co	43	GC19P048	2.9
3972	RARG	Retinoic A	Protein Co	47	GC12M053	2.9
3973	CCL27	C-C Motif	Protein Co	37	GC09M034	2.9
3974	GABRA1	Gamma-A	Protein Co	46	GC05P161	2.9
3975	ZMYND11	Zinc Finge	Protein Co	40	GC10P000	2.9
3976	RAB27A	RAB27A, M	Protein Co	48	GC15M055	2.9
3977	GPR15	G Protein-	Protein Co	38	GC03P098	2.9
3978	YARS2	Tyrosyl-TR	Protein Co	44	GC12M032	2.9
3979	ATAD3A	ATPase Fa	Protein Co	39	GC01P001	2.9
3980	LOXL2	Lysyl Oxid	Protein Co	44	GC08M023	2.89
3981	CELF1	CUGBP Ela	Protein Co	37	GC11M061	2.89
3982	QKI	QKI, KH D	Protein Co	43	GC06P163	2.89
3983	SNX9	Sorting Ne	Protein Co	41	GC06P157	2.89
3984	TMC8	Transmem	Protein Co	40	GC17P078	2.89
3985	TSC22D1	TSC22 Dor	Protein Co	40	GC13M044	2.88
3986	CHD5	Chromodo	Protein Co	40	GC01M006	2.88
3987	TMEM52	Transmem	Protein Co	28	GC01M001	2.88
3988	ITPA	Inosine Tri	Protein Co	47	GC20P003	2.88
3989	BCL2L15	BCL2 Like	Protein Co	33	GC01M113	2.88
3990	VPS52	VPS52 Sub	Protein Co	36	GC06M033	2.88
3991	SLC5A3	Solute Car	Protein Co	43	GC21P034	2.88
3992	B4GALT7	Beta-1,4-G	Protein Co	41	GC05P177	2.87
3993	SIX2	SIX Homec	Protein Co	40	GC02M045	2.87
3994	ACSL6	Acyl-CoA S	Protein Co	39	GC05M131	2.87
3995	MMP28	Matrix Me	Protein Co	37	GC17M035	2.87
3996	HLA-DOB	Major Hist	Protein Co	40	GC06M032	2.87
3997	IGFBP6	Insulin Like	Protein Co	42	GC12P053	2.87

3998	TPM4	Tropomyo:	Protein Co	40	GC19P023	2.87
3999	ITPR1	Inositol 1,4	Protein Co	48	GC03P004	2.87
4000	SLC9B1	Solute Car	Protein Co	33	GC04M102	2.87
4001	SLC7A2	Solute Car	Protein Co	42	GC08P017	2.87
4002	PBX1	PBX Home	Protein Co	49	GC01P164	2.87
4003	ID3	Inhibitor C	Protein Co	41	GC01M023	2.87
4004	ACTN1	Actinin Alp	Protein Co	50	GC14M068	2.86
4005	LNX1	Ligand Of	Protein Co	43	GC04M053	2.86
4006	ANAPC2	Anaphase	Protein Co	40	GC09M137	2.86
4007	TRAF1	TNF Recep	Protein Co	43	GC09M120	2.86
4008	CCL19	C-C Motif	Protein Co	41	GC09M034	2.86
4009	GIT1	GIT ArfGAI	Protein Co	43	GC17M029	2.86
4010	RLIM	Ring Finge	Protein Co	39	GC0XM074	2.86
4011	SGCB	Sarcoglyca	Protein Co	40	GC04M052	2.86
4012	GABRA2	Gamma-Ar	Protein Co	45	GC04M046	2.85
4013	SPINK4	Serine Pep	Protein Co	33	GC09P033	2.85
4014	VASP	Vasodilato	Protein Co	43	GC19P045	2.85
4015	ATE1	Arginyltrar	Protein Co	39	GC10M121	2.85
4016	WDR62	WD Repea	Protein Co	38	GC19P036	2.85
4017	UBE2C	Ubiquitin (	Protein Co	47	GC20P045	2.85
4018	PNMT	Phenyletha	Protein Co	44	GC17P039	2.85
4019	CEP135	Centrosom	Protein Co	38	GC04P055	2.84
4020	RSPH3	Radial Spo	Protein Co	36	GC06M158	2.84
4021	NPPC	Natriuretic	Protein Co	40	GC02M231	2.84
4022	ABCF1	ATP Bindir	Protein Co	39	GC06P030	2.84
4023	PCBD1	Pterin-4 Al	Protein Co	45	GC10M070	2.84
4024	SLC45A1	Solute Car	Protein Co	32	GC01P008	2.84
4025	RAPGEF4	Rap Guani	Protein Co	43	GC02P172	2.84
4026	ANKRD11	Ankyrin Re	Protein Co	38	GC16M089	2.84
4027	PRMT5	Protein Ar(	Protein Co	42	GC14M022	2.84
4028	SLC2A6	Solute Car	Protein Co	41	GC09M133	2.83
4029	BCL2L13	BCL2 Like	Protein Co	39	GC22P017	2.83
4030	SBF2-AS1	SBF2 Antis	RNA Gene	15	GC11P009	2.83
4031	ASPN	Asporin	Protein Co	39	GC09M092	2.83
4032	PIBF1	Progesterc	Protein Co	40	GC13P072	2.83
4033	GFER	Growth Fa	Protein Co	45	GC16P001	2.83
4034	NCK1	NCK Adap	Protein Co	45	GC03P136	2.83
4035	BAG3	BAG Cocha	Protein Co	44	GC10P119	2.83
4036	FADS1	Fatty Acid	Protein Co	44	GC11M061	2.83
4037	PSMB4	Proteasom	Protein Co	45	GC01P151	2.83
4038	NOTCH4	Notch Rec	Protein Co	45	GC06M032	2.83
4039	VSTM4	V-Set And	Protein Co	32	GC10M049	2.83
4040	SLC44A4	Solute Car	Protein Co	41	GC06M031	2.82

4041	MIR411	MicroRNA	RNA Gene	14	GC14P104	2.82
4042	LOC10678	Tenascin X	Biological	1	GC06P047	2.82
4043	RHBDD3	Rhomboid	Protein Co	34	GC22M029	2.82
4044	ID1	Inhibitor C	Protein Co	43	GC20P031	2.82
4045	DNAAF5	Dynein Axo	Protein Co	36	GC07P000	2.82
4046	PRDM5	PR/SET Do	Protein Co	39	GC04M120	2.82
4047	TRA	T Cell Rece	Protein Co	17	GC14P021	2.82
4048	KLHL17	Kelch Like	Protein Co	33	GC01P000	2.82
4049	C16orf95	Chromoso	Protein Co	26	GC16M087	2.82
4050	ATG7	Autophagy	Protein Co	43	GC03P011	2.81
4051	SLC7A1	Solute Car	Protein Co	43	GC13M029	2.81
4052	PEX19	Peroxisom	Protein Co	43	GC01M160	2.81
4053	PLCE1	Phospholip	Protein Co	44	GC10P093	2.81
4054	ECT2	Epithelial C	Protein Co	40	GC03P172	2.81
4055	RBPJ	Recombina	Protein Co	47	GC04P026	2.81
4056	TXNIP	Thioredoxi	Protein Co	37	GC01M145	2.81
4057	CLEC12A	C-Type Le	Protein Co	37	GC12P009	2.81
4058	ADSS1	Adenylosu	Protein Co	36	GC14P104	2.81
4059	EDAR	Ectodyspla	Protein Co	41	GC02M108	2.81
4060	USF2	Upstream	Protein Co	39	GC19P035	2.81
4061	CD2AP	CD2 Assoc	Protein Co	43	GC06P047	2.81
4062	CXCL6	C-X-C Mot	Protein Co	40	GC04P073	2.8
4063	CRAT	Carnitine C	Protein Co	44	GC09M129	2.8
4064	CCDC39	Coiled-Coi	Protein Co	36	GC03M180	2.8
4065	IDE	Insulin Deg	Protein Co	48	GC10M092	2.8
4066	FCER1G	Fc Fragme	Protein Co	43	GC01P161	2.8
4067	EREG	Epiregulin	Protein Co	41	GC04P074	2.8
4068	PANK4	Pantothen	Protein Co	40	GC01M002	2.8
4069	AGBL2	ATP/GTP B	Protein Co	37	GC11M061	2.8
4070	CISD3	CDGSH Iro	Protein Co	30	GC17P038	2.8
4071	TMEM238	Transmem	Protein Co	27	GC19M055	2.8
4072	AARS2	Alanyl-TRN	Protein Co	42	GC06M044	2.8
4073	HSPA1L	Heat Shoc	Protein Co	43	GC06M031	2.8
4074	P4HTM	Prolyl 4-Hy	Protein Co	36	GC03P049	2.8
4075	CA2	Carbonic A	Protein Co	51	GC08P085	2.79
4076	PEX13	Peroxisom	Protein Co	40	GC02P061	2.79
4077	TNFRSF10	TNF Recep	Protein Co	39	GC08P023	2.79
4078	MRGPRX2	MAS Relat	Protein Co	37	GC11M019	2.79
4079	HYLS1	HYLS1 Cer	Protein Co	37	GC11P125	2.79
4080	ALDH7A1	Aldehyde I	Protein Co	47	GC05M126	2.79
4081	TBX6	T-Box Tran	Protein Co	39	GC16M030	2.79
4082	FPR2	Formyl Pep	Protein Co	45	GC19P051	2.79
4083	GALT	Galactose-	Protein Co	47	GC09P034	2.79

4084	HTRA2	HtrA Serin	Protein Co	47	GC02P074	2.78
4085	INA	Internexin	Protein Co	39	GC10P103	2.78
4086	RAB11B	RAB11B, M	Protein Co	42	GC19P008	2.78
4087	GLYAT	Glycine-N-	Protein Co	41	GC11M061	2.78
4088	BCL9	BCL9 Trans	Protein Co	41	GC01P147	2.78
4089	SLC23A2	Solute Car	Protein Co	42	GC20M004	2.78
4090	PLCH2	Phospholip	Protein Co	39	GC01P002	2.78
4091	HGS	Hepatocyte	Protein Co	44	GC17P081	2.78
4092	PTPRF	Protein Ty	Protein Co	50	GC01P043	2.78
4093	MIR92A2	MicroRNA	RNA Gene	16	GC0XM134	2.78
4094	TPT1	Tumor Pro	Protein Co	46	GC13M045	2.78
4095	GSTA2	Glutathion	Protein Co	41	GC06M052	2.78
4096	SHANK3	SH3 And M	Protein Co	40	GC22P050	2.78
4097	SHARPIN	SHANK As	Protein Co	40	GC08M144	2.78
4098	ZNF423	Zinc Finge	Protein Co	40	GC16M049	2.78
4099	YWHAQ	Tyrosine 3	Protein Co	47	GC02M009	2.77
4100	FA2H	Fatty Acid	Protein Co	44	GC16M074	2.77
4101	ELMO1	Engulfmen	Protein Co	40	GC07M036	2.77
4102	GOSR2	Golgi SNA	Protein Co	43	GC17P046	2.77
4103	TXLNB	Taxilin Bet	Protein Co	35	GC06M139	2.77
4104	DERL2	Derlin 2	Protein Co	37	GC17M005	2.77
4105	SNAPIN	SNAP Assc	Protein Co	37	GC01P153	2.77
4106	BTG1	BTG Anti-P	Protein Co	41	GC12M092	2.77
4107	IL24	Interleukin	Protein Co	42	GC01P206	2.77
4108	LGI1	Leucine Ri	Protein Co	43	GC10P093	2.77
4109	PSD3	Pleckstrin /	Protein Co	39	GC08M018	2.77
4110	MFSD2A	Major Faci	Protein Co	40	GC01P039	2.76
4111	CD33	CD33 Mole	Protein Co	43	GC19P051	2.76
4112	SYN2	Synapsin II	Protein Co	39	GC03P012	2.76
4113	SPTBN4	Spectrin B	Protein Co	39	GC19P040	2.76
4114	STIL	STIL Centri	Protein Co	41	GC01M047	2.76
4115	MIR490	MicroRNA	RNA Gene	16	GC07P136	2.76
4116	PURA	Purine Rich	Protein Co	43	GC05P140	2.76
4117	PTBP1	Polypyrimi	Protein Co	41	GC19P000	2.76
4118	ZFP36	ZFP36 Ring	Protein Co	39	GC19P039	2.75
4119	IL36RN	Interleukin	Protein Co	41	GC02P115	2.75
4120	UPK1B	Uroplakin	Protein Co	36	GC03P119	2.75
4121	SCIN	Scinderin	Protein Co	37	GC07P012	2.75
4122	BOLA3	BolA Famil	Protein Co	38	GC02M074	2.75
4123	GIMAP5	GTPase, IM	Protein Co	36	GC07P150	2.75
4124	APIP	APAF1 Inte	Protein Co	39	GC11M034	2.75
4125	RSPH1	Radial Spo	Protein Co	39	GC21M042	2.75
4126	CDC25A	Cell Divisic	Protein Co	47	GC03M048	2.75

4127	TCIRG1	T Cell Imm	Protein Co	45	GC11P068	2.75
4128	TRIM33	Tripartite M	Protein Co	44	GC01M114	2.75
4129	TLE1	TLE Family	Protein Co	44	GC09M081	2.75
4130	RANBP1	RAN Bindi	Protein Co	43	GC22P020	2.75
4131	EIF2B1	Eukaryotic	Protein Co	43	GC12M123	2.74
4132	NAPEPLD	N-Acyl Phc	Protein Co	40	GC07M103	2.74
4133	LAMP1	Lysosomal	Protein Co	43	GC13P113	2.74
4134	GAS8	Growth Ar	Protein Co	39	GC16P090	2.74
4135	CEMIP2	Cell Migra	Protein Co	27	GC09M071	2.74
4136	ZEB1-AS1	ZEB1 Antis	RNA Gene	16	GC10M031	2.74
4137	TMEM126A	Transmem	Protein Co	39	GC11P085	2.74
4138	CFAP74	Cilia And F	Protein Co	27	GC01M001	2.74
4139	CREM	CAMP Res	Protein Co	41	GC10P035	2.74
4140	PISRT1	PISRT1 Lnc	RNA Gene	14	GC03M139	2.74
4141	FFAR4	Free Fatty	Protein Co	38	GC10P093	2.74
4142	PIN1	Peptidylpr	Protein Co	47	GC19P009	2.74
4143	UFC1	Ubiquitin-I	Protein Co	37	GC01P161	2.74
4144	FOXA1	Forkhead I	Protein Co	43	GC14M037	2.74
4145	BPTF	Bromodon	Protein Co	40	GC17P067	2.74
4146	RAB1A	RAB1A, M	Protein Co	41	GC02M065	2.74
4147	GRM1	Glutamate	Protein Co	50	GC06P145	2.73
4148	CNPY3	Canopy FG	Protein Co	40	GC06P047	2.73
4149	POU5F1	POU Class	Protein Co	47	GC06M031	2.73
4150	CD160	CD160 Mo	Protein Co	39	GC01P145	2.73
4151	A4GALT	Alpha 1,4-	Protein Co	43	GC22M042	2.73
4152	HNRNPA1	Heterogen	Protein Co	45	GC12P054	2.73
4153	RYR3	Ryanodine	Protein Co	41	GC15P033	2.73
4154	TXNRD1	Thioredoxi	Protein Co	47	GC12P104	2.73
4155	APELA	Apelin Rec	Protein Co	19	GC04P164	2.73
4156	NMT1	N-Myristoy	Protein Co	43	GC17P045	2.73
4157	RPL11	Ribosomal	Protein Co	48	GC01P023	2.73
4158	STOML2	Stomatin L	Protein Co	38	GC09M035	2.73
4159	SCN4A	Sodium Vc	Protein Co	44	GC17M063	2.73
4160	ATXN7	Ataxin 7	Protein Co	40	GC03P063	2.73
4161	SRSF1	Serine Anc	Protein Co	41	GC17M058	2.73
4162	SLC27A1	Solute Car	Protein Co	41	GC19P023	2.73
4163	FABP4	Fatty Acid	Protein Co	44	GC08M081	2.73
4164	C12orf57	Chromoso	Protein Co	37	GC12P008	2.73
4165	ANG	Angiogeni	Protein Co	45	GC14P020	2.72
4166	CREB3L1	CAMP Res	Protein Co	41	GC11P046	2.72
4167	TNFRSF18	TNF Recep	Protein Co	42	GC01M001	2.72
4168	CASQ1	Calsequest	Protein Co	43	GC01P160	2.72
4169	KCNAB1	Potassium	Protein Co	41	GC03P156	2.72

4170	NDUFB6	NADH:Ubi	Protein Co	40	GC09M032	2.72
4171	DOCK11	Dedicator	Protein Co	37	GC0XP118	2.72
4172	LRIG1	Leucine Ri	Protein Co	41	GC03M066	2.72
4173	COBL	Cordon-Bl	Protein Co	34	GC07M051	2.72
4174	GOLGA2	Golgin A2	Protein Co	40	GC09M128	2.72
4175	CLN3	CLN3 Lyso	Protein Co	43	GC16M028	2.72
4176	MFAP4	Microfibril	Protein Co	40	GC17M019	2.72
4177	PPID	Peptidylpr	Protein Co	43	GC04M158	2.71
4178	SPG7	SPG7 Matr	Protein Co	43	GC16P089	2.71
4179	TRIM72	Tripartite M	Protein Co	36	GC16P031	2.71
4180	GFM1	G Elongati	Protein Co	44	GC03P158	2.71
4181	P2RX1	Purinergic	Protein Co	43	GC17M003	2.71
4182	MAZ	MYC Asso	Protein Co	40	GC16P029	2.71
4183	SLCO3A1	Solute Car	Protein Co	40	GC15P091	2.71
4184	SNRPD1	Small Nucl	Protein Co	39	GC18P021	2.71
4185	ESRRB	Estrogen R	Protein Co	50	GC14P076	2.71
4186	CCNG1	Cyclin G1	Protein Co	42	GC05P163	2.71
4187	XIRP1	Xin Actin E	Protein Co	35	GC03M039	2.7
4188	ANGPTL2	Angiopoiet	Protein Co	36	GC09M127	2.7
4189	PLIN1	Perilipin 1	Protein Co	44	GC15M089	2.7
4190	APOA2	Apolipopr	Protein Co	44	GC01M161	2.7
4191	PTPN14	Protein Ty	Protein Co	43	GC01M214	2.7
4192	GRIN2C	Glutamate	Protein Co	44	GC17M074	2.7
4193	FST	Follistatin	Protein Co	46	GC05P053	2.7
4194	XIST	X Inactive	RNA Gene	24	GC0XM073	2.7
4195	MDH2	Malate De	Protein Co	48	GC07P076	2.7
4196	ESM1	Endothelia	Protein Co	40	GC05M054	2.7
4197	SLC25A18	Solute Car	Protein Co	40	GC22P017	2.7
4198	ZNF804A	Zinc Finge	Protein Co	34	GC02P184	2.7
4199	AQP5	Aquaporin	Protein Co	45	GC12P049	2.7
4200	MAP3K12	Mitogen-A	Protein Co	45	GC12M053	2.69
4201	USF1	Upstream	Protein Co	43	GC01M161	2.69
4202	VGF	VGF Nerve	Protein Co	38	GC07M101	2.69
4203	HNRNPA2I	Heterogen	Protein Co	45	GC07M026	2.69
4204	CLASRP	CLK4 Asso	Protein Co	32	GC19P045	2.69
4205	EHD1	EH Domair	Protein Co	41	GC11M064	2.69
4206	PLCB4	Phospholip	Protein Co	47	GC20P009	2.69
4207	CTSC	Cathepsin	Protein Co	45	GC11M088	2.69
4208	NCKAP1	NCK Assoc	Protein Co	39	GC02M182	2.69
4209	SZT2	SZT2 Subu	Protein Co	36	GC01P043	2.69
4210	ACOT7	Acyl-CoA T	Protein Co	41	GC01M006	2.69
4211	RBM3	RNA Bindi	Protein Co	39	GC0XP048	2.69
4212	COL5A3	Collagen T	Protein Co	36	GC19M009	2.68

4213	ZFHX2	Zinc Finge	Protein Co	32	GC14M023	2.68
4214	SLC26A9	Solute Car	Protein Co	40	GC01M205	2.68
4215	POMGNT2	Protein O-	Protein Co	36	GC03M043	2.68
4216	ETV7	ETS Varian	Protein Co	36	GC06M042	2.68
4217	PRLR	Prolactin R	Protein Co	48	GC05M035	2.68
4218	MTFR1	Mitochonc	Protein Co	36	GC08P065	2.68
4219	CDC25B	Cell Divisic	Protein Co	46	GC20P003	2.68
4220	LRP4	LDL Recep	Protein Co	43	GC11M061	2.68
4221	GYPE	Glycophori	Protein Co	31	GC04M143	2.68
4222	NT5M	5',3'-Nucle	Protein Co	36	GC17P017	2.68
4223	TOP3A	DNA Topo	Protein Co	42	GC17M018	2.67
4224	SULF1	Sulfatase 1	Protein Co	41	GC08P069	2.67
4225	HPGDS	Hematopo	Protein Co	42	GC04M094	2.67
4226	CTSE	Cathepsin	Protein Co	42	GC01M206	2.67
4227	ACTRT2	Actin Relat	Protein Co	35	GC01P003	2.67
4228	FAM72A	Family Wit	Protein Co	33	GC01M206	2.67
4229	RB1CC1	RB1 Induci	Protein Co	42	GC08M052	2.67
4230	TYRO3	TYRO3 Pro	Protein Co	47	GC15P041	2.67
4231	MYLK2	Myosin Lig	Protein Co	47	GC20P031	2.67
4232	ROCK1	Rho Assoc	Protein Co	50	GC18M020	2.67
4233	TAT	Tyrosine A	Protein Co	44	GC16M071	2.67
4234	REV3L	REV3 Like,	Protein Co	44	GC06M111	2.67
4235	GABRB2	Gamma-Ar	Protein Co	47	GC05M161	2.67
4236	CREBZF	CREB/ATF	Protein Co	36	GC11M085	2.67
4237	AFF4	AF4/FMR2	Protein Co	41	GC05M132	2.67
4238	MAT2A	Methionin	Protein Co	47	GC02P085	2.67
4239	TMIGD1	Transmem	Protein Co	31	GC17M030	2.67
4240	LY86	Lymphocyt	Protein Co	37	GC06P006	2.67
4241	SLC5A7	Solute Car	Protein Co	44	GC02P107	2.66
4242	FIS1	Fission, Mi	Protein Co	39	GC07M101	2.66
4243	POLB	DNA Polyr	Protein Co	47	GC08P042	2.66
4244	GAS2L2	Growth Ar	Protein Co	33	GC17M035	2.66
4245	YWHAZ	Tyrosine 3	Protein Co	48	GC08M100	2.66
4246	TOR3A	Torsin Fam	Protein Co	36	GC01P179	2.66
4247	ATP2C1	ATPase Se	Protein Co	45	GC03P130	2.66
4248	SULT2B1	Sulfotransf	Protein Co	45	GC19P048	2.66
4249	NRXN3	Neurexin 3	Protein Co	41	GC14P077	2.66
4250	RAB3GAP1	RAB3 GTP	Protein Co	40	GC02P135	2.66
4251	MYH10	Myosin He	Protein Co	46	GC17M008	2.66
4252	ADD2	Adducin 2	Protein Co	40	GC02M070	2.66
4253	YBX1	Y-Box Binc	Protein Co	39	GC01P042	2.66
4254	MEGF10	Multiple E	Protein Co	40	GC05P127	2.66
4255	STXBP5	Syntaxin B	Protein Co	38	GC06P147	2.65

4256	NPHS1	NPHS1 Ad	Protein Co	45	GC19M035	2.65
4257	INTS7	Integrator	Protein Co	36	GC01M211	2.65
4258	C1GALT1	Core 1 Syr	Protein Co	40	GC07P007	2.65
4259	IL25	Interleukin	Protein Co	38	GC14P025	2.65
4260	TAFA4	TAFA Cher	Protein Co	27	GC03M068	2.65
4261	MCM3AP	Minichrom	Protein Co	40	GC21M046	2.65
4262	FKBP14	FKBP Proly	Protein Co	40	GC07M030	2.65
4263	XPOT	Exportin F	Protein Co	39	GC12P064	2.65
4264	PRSS16	Serine Pro	Protein Co	37	GC06P027	2.65
4265	SYT10	Synaptotag	Protein Co	34	GC12M033	2.65
4266	AJAP1	Adherens J	Protein Co	33	GC01P004	2.65
4267	BMPR1B	Bone Morp	Protein Co	50	GC04P094	2.65
4268	CHRFAM7A	CHRNA7 (I	Protein Co	32	GC15M030	2.65
4269	IER3	Immediate	Protein Co	38	GC06M030	2.65
4270	MPG	N-Methylp	Protein Co	41	GC16P001	2.65
4271	DBP	D-Box Bind	Protein Co	38	GC19M048	2.64
4272	GLS2	Glutaminas	Protein Co	41	GC12M056	2.64
4273	CHD3	Chromodo	Protein Co	41	GC17P008	2.64
4274	P2RX2	Purinergic	Protein Co	43	GC12P132	2.64
4275	INHBB	Inhibin Sul	Protein Co	42	GC02P120	2.64
4276	ZNF81	Zinc Finge	Protein Co	33	GC0XP047	2.64
4277	CFAP298	Cilia And F	Protein Co	30	GC21M032	2.64
4278	MYO1E	Myosin IE	Protein Co	43	GC15M059	2.64
4279	UNC5C	Unc-5 Net	Protein Co	40	GC04M095	2.64
4280	RBL2	RB Transcr	Protein Co	43	GC16P053	2.64
4281	NDUFS2	NADH:Ubi	Protein Co	44	GC01P161	2.64
4282	PPM1D	Protein Ph	Protein Co	47	GC17P060	2.64
4283	KLK1	Kallikrein 1	Protein Co	45	GC19M050	2.64
4284	TRIT1	TRNA Isop	Protein Co	41	GC01M039	2.64
4285	TBCC	Tubulin Fo	Protein Co	36	GC06M042	2.63
4286	TRIP4	Thyroid Ho	Protein Co	40	GC15P072	2.63
4287	ERI1	Exoribonu	Protein Co	36	GC08P008	2.63
4288	RND3	Rho Family	Protein Co	41	GC02M150	2.63
4289	RGS5	Regulator	Protein Co	40	GC01M163	2.63
4290	NGDN	Neuroguid	Protein Co	33	GC14P023	2.63
4291	CD1C	CD1c Mole	Protein Co	39	GC01P158	2.63
4292	CTSH	Cathepsin	Protein Co	47	GC15M078	2.63
4293	MIR224	MicroRNA	RNA Gene	17	GC0XM151	2.63
4294	CALM1	Calmodulin	Protein Co	45	GC14P090	2.63
4295	NDUFA6	NADH:Ubi	Protein Co	44	GC22M042	2.63
4296	TACR3	Tachykinin	Protein Co	50	GC04M103	2.63
4297	CUL4B	Cullin 4B	Protein Co	43	GC0XM120	2.62
4298	CERS2	Ceramide S	Protein Co	40	GC01M150	2.62

4299	SORT1	Sortilin 1	Protein Co	43	GC01M109	2.62
4300	ZFYVE9	Zinc Finge	Protein Co	41	GC01P052	2.62
4301	LIN28A	Lin-28 Hor	Protein Co	40	GC01P026	2.62
4302	A2M	Alpha-2-M	Protein Co	45	GC12M009	2.62
4303	NSF	N-Ethylma	Protein Co	44	GC17P046	2.62
4304	RAI2	Retinoic A	Protein Co	35	GC0XM017	2.62
4305	DUSP5	Dual Speci	Protein Co	41	GC10P110	2.62
4306	FGF12	Fibroblast	Protein Co	43	GC03M192	2.62
4307	SLC24A2	Solute Car	Protein Co	39	GC09M019	2.62
4308	SEMA6A	Semaphori	Protein Co	39	GC05M116	2.62
4309	EBI3	Epstein-Ba	Protein Co	39	GC19P004	2.61
4310	PLAGL2	PLAG1 Like	Protein Co	37	GC20M032	2.61
4311	GPNMB	Glycoprote	Protein Co	44	GC07P023	2.61
4312	PEX12	Peroxisom	Protein Co	39	GC17M035	2.61
4313	CEP41	Centrosom	Protein Co	39	GC07M130	2.61
4314	RAD51D	RAD51 Par	Protein Co	40	GC17M035	2.61
4315	S100A13	S100 Calci	Protein Co	37	GC01M153	2.61
4316	KHDRBS1	KH RNA Bi	Protein Co	41	GC01P031	2.61
4317	RNASEH2B	Ribonuclea	Protein Co	37	GC13P050	2.61
4318	TRMT1	TRNA Met	Protein Co	41	GC19M013	2.61
4319	AZU1	Azurocidin	Protein Co	39	GC19P000	2.61
4320	VAMP1	Vesicle Ass	Protein Co	44	GC12M006	2.61
4321	PDE4B	Phosphodi	Protein Co	44	GC01P065	2.61
4322	ZFHX4	Zinc Finge	Protein Co	37	GC08P076	2.6
4323	LIPN	Lipase Fam	Protein Co	36	GC10P088	2.6
4324	SAE1	SUMO1 Ac	Protein Co	45	GC19P047	2.6
4325	ATXN10	Ataxin 10	Protein Co	42	GC22P045	2.6
4326	SLK	STE20 Like	Protein Co	43	GC10P103	2.6
4327	PTOV1-AS1	PTOV1 Ant	RNA Gene	12	GC19M049	2.6
4328	BCL11B	BAF Chron	Protein Co	42	GC14M099	2.6
4329	SNRPD3	Small Nucl	Protein Co	36	GC22P024	2.6
4330	GRIK2	Glutamate	Protein Co	47	GC06P101	2.6
4331	CIB1	Calcium An	Protein Co	41	GC15M090	2.6
4332	MAP1LC3B	Microtubul	Protein Co	43	GC16P087	2.6
4333	RBFOX3	RNA Bindi	Protein Co	35	GC17M079	2.6
4334	CHRNB2	Cholinergi	Protein Co	44	GC01P154	2.6
4335	PLEKHG5	Pleckstrin	Protein Co	40	GC01M006	2.59
4336	BATF	Basic Leuc	Protein Co	43	GC14P075	2.59
4337	DOCK1	Dedicator	Protein Co	44	GC10P126	2.59
4338	SNORA54	Small Nucl	RNA Gene	17	GC11M002	2.59
4339	CLTCL1	Clathrin He	Protein Co	41	GC22M019	2.59
4340	SERPINI2	Serpin Fam	Protein Co	39	GC03M167	2.59
4341	TOM1L2	Target Of	Protein Co	38	GC17M017	2.59

4342	PALM2AK/	PALM2 An	Protein Co	22	GC09P109	2.59
4343	PROK2	Prokineticı	Protein Co	44	GC03M071	2.59
4344	CYFIP2	Cytoplasm	Protein Co	43	GC05P157	2.58
4345	CLEC4M	C-Type Le	Protein Co	41	GC19P007	2.58
4346	GAN	Gigaxonin	Protein Co	39	GC16P081	2.58
4347	PDHA1	Pyruvate D	Protein Co	48	GC0XP019	2.58
4348	GNPTAB	N-Acetylgl	Protein Co	41	GC12M101	2.58
4349	CDK9	Cyclin Dep	Protein Co	45	GC09P127	2.58
4350	LUM	Lumican	Protein Co	41	GC12M091	2.58
4351	ZBTB8OS	Zinc Finge	Protein Co	36	GC01M032	2.58
4352	RAP2B	RAP2B, Me	Protein Co	39	GC03P153	2.58
4353	CLTC	Clathrin H	Protein Co	46	GC17P059	2.58
4354	RALGPS2	Ral GEF W	Protein Co	35	GC01P178	2.58
4355	RAD23B	RAD23 Ho	Protein Co	44	GC09P107	2.58
4356	PTPRM	Protein Ty	Protein Co	41	GC18P007	2.58
4357	CERS5	Ceramide	Protein Co	37	GC12M050	2.58
4358	LGALS7	Galectin 7	Protein Co	37	GC19M038	2.58
4359	DRG2	Developm	Protein Co	38	GC17P018	2.58
4360	MIR4435-2	MIR4435-2	RNA Gene	17	GC02M111	2.58
4361	TST	Thiosulfate	Protein Co	42	GC22M037	2.58
4362	ATIC	5-Aminoim	Protein Co	45	GC02P215	2.58
4363	IKBKE	Inhibitor C	Protein Co	45	GC01P206	2.58
4364	CCDC151	Coiled-Coi	Protein Co	37	GC19M011	2.58
4365	EIF2B5	Eukaryotic	Protein Co	43	GC03P184	2.58
4366	SCGN	Secretagoc	Protein Co	38	GC06P025	2.58
4367	SSRP1	Structure S	Protein Co	40	GC11M061	2.57
4368	TXNDC2	Thioredoxi	Protein Co	35	GC18P009	2.57
4369	KCNT1	Potassium	Protein Co	43	GC09P135	2.57
4370	IAPP	Islet Amylc	Protein Co	40	GC12P021	2.57
4371	CCDC114	Coiled-Coi	Protein Co	36	GC19M048	2.57
4372	TTC25	Tetratricop	Protein Co	35	GC17P041	2.57
4373	ROBO4	Roundabo	Protein Co	41	GC11M124	2.57
4374	IL19	Interleukin	Protein Co	40	GC01P206	2.56
4375	UGT8	UDP Glyco	Protein Co	43	GC04P114	2.56
4376	CREB3L4	CAMP Res	Protein Co	37	GC01P153	2.56
4377	P2RY6	Pyrimidine	Protein Co	42	GC11P073	2.56
4378	FBXO7	F-Box Prot	Protein Co	41	GC22P032	2.56
4379	SMARCC1	SWI/SNF R	Protein Co	43	GC03M047	2.56
4380	MYBPC3	Myosin Bir	Protein Co	46	GC11M061	2.56
4381	TRIL	TLR4 Inter	Protein Co	32	GC07M028	2.56
4382	ADRA2C	Adrenocep	Protein Co	45	GC04P003	2.56
4383	SPEF2	Sperm Fla	Protein Co	35	GC05P035	2.56
4384	DDX53	DEAD-Box	Protein Co	35	GC0XP022	2.56

4385	NARS1	Asparagin	Protein Co	34	GC18M057	2.56
4386	ETFA	Electron Tr	Protein Co	45	GC15M076	2.56
4387	ADAM28	ADAM Me	Protein Co	39	GC08P024	2.55
4388	HRG	Histidine R	Protein Co	41	GC03P186	2.55
4389	SLC4A7	Solute Car	Protein Co	42	GC03M027	2.55
4390	AMPD3	Adenosine	Protein Co	45	GC11P010	2.55
4391	UGDH	UDP-Glucc	Protein Co	45	GC04M039	2.55
4392	CHKB	Choline Ki	Protein Co	45	GC22M050	2.55
4393	AGFG1	ArfGAP Wi	Protein Co	39	GC02P227	2.55
4394	CLEC4A	C-Type Le	Protein Co	37	GC12P008	2.55
4395	STOX1	Storkhead	Protein Co	39	GC10P068	2.55
4396	PARD3	Par-3 Fami	Protein Co	41	GC10M034	2.55
4397	ACTR2	Actin Relat	Protein Co	43	GC02P065	2.55
4398	MEIS1	Meis Hom	Protein Co	42	GC02P066	2.55
4399	EEF1A2	Eukaryotic	Protein Co	45	GC20M063	2.55
4400	ATOX1	Antioxidan	Protein Co	40	GC05M151	2.54
4401	SESN2	Sestrin 2	Protein Co	39	GC01P028	2.54
4402	SLC16A2	Solute Car	Protein Co	45	GC0XP074	2.54
4403	SF3B2	Splicing Fa	Protein Co	38	GC11P066	2.54
4404	IL1R2	Interleukin	Protein Co	45	GC02P101	2.54
4405	SLC29A4	Solute Car	Protein Co	42	GC07P005	2.54
4406	RASD1	Ras Relate	Protein Co	39	GC17M017	2.54
4407	ZIC1	Zic Family	Protein Co	45	GC03P147	2.54
4408	IGF2-AS	IGF2 Antis	RNA Gene	24	GC11P002	2.54
4409	STPG3	Sperm-Tail	Protein Co	23	GC09P137	2.54
4410	MIR1304	MicroRNA	RNA Gene	16	GC11M094	2.54
4411	VEGFB	Vascular E	Protein Co	44	GC11P064	2.54
4412	CSNK1E	Casein Kin	Protein Co	47	GC22M045	2.54
4413	TRA2B	Transforme	Protein Co	39	GC03M185	2.54
4414	ALDH1B1	Aldehyde I	Protein Co	44	GC09P038	2.53
4415	ITK	IL2 Induci	Protein Co	51	GC05P157	2.53
4416	CD276	CD276 Mo	Protein Co	40	GC15P073	2.53
4417	ARMC4	Armadillo	Protein Co	37	GC10M027	2.53
4418	GTF3A	General Tr	Protein Co	36	GC13P027	2.53
4419	PABPC1	Poly(A) Bir	Protein Co	41	GC08M100	2.53
4420	ATAD1	ATPase Fa	Protein Co	41	GC10M087	2.53
4421	TBCK	TBC1 Dom	Protein Co	40	GC04M106	2.53
4422	TINAG	Tubulointe	Protein Co	39	GC06P054	2.53
4423	SERPINA6	Serpin Fan	Protein Co	43	GC14M094	2.53
4424	PCDH10	Protocadh	Protein Co	39	GC04P133	2.53
4425	EMP3	Epithelial M	Protein Co	39	GC19P048	2.53
4426	OAS1	2'-5'-Oligo	Protein Co	45	GC12P112	2.53
4427	PGD	Phosphogl	Protein Co	47	GC01P010	2.52

4428	BTRC	Beta-Trans	Protein Co	46	GC10P101	2.52
4429	PPIF	Peptidylpr	Protein Co	43	GC10P083	2.52
4430	MXRA8	Matrix Ren	Protein Co	35	GC01M001	2.52
4431	GRIK1	Glutamate	Protein Co	45	GC21M029	2.52
4432	MLYCD	Malonyl-C	Protein Co	43	GC16P083	2.52
4433	TNFRSF14	TNF Recep	Protein Co	43	GC01P002	2.52
4434	CDC5L	Cell Divisic	Protein Co	39	GC06P044	2.52
4435	IL17RC	Interleukin	Protein Co	40	GC03P009	2.52
4436	CACNA1D	Calcium V	Protein Co	47	GC03P053	2.52
4437	ABCC5	ATP Bindir	Protein Co	43	GC03M183	2.52
4438	SPIC	Spi-C Tran	Protein Co	35	GC12P101	2.52
4439	PUF60	Poly(U) Bir	Protein Co	41	GC08M143	2.52
4440	EBF3	EBF Transc	Protein Co	40	GC10M129	2.51
4441	CCL24	C-C Motif	Protein Co	36	GC07M075	2.51
4442	PLPBP	Pyridoxal F	Protein Co	32	GC08P037	2.51
4443	MT1E	Metallothi	Protein Co	39	GC16P056	2.51
4444	EDC4	Enhancer (	Protein Co	38	GC16P067	2.51
4445	CXCR6	C-X-C Mot	Protein Co	40	GC03P045	2.51
4446	ISG15	ISG15 Ubi	Protein Co	47	GC01P001	2.51
4447	MAN2A1	Mannosida	Protein Co	42	GC05P109	2.51
4448	VPS33A	VPS33A C	Protein Co	41	GC12M122	2.51
4449	CFAP221	Cilia And F	Protein Co	27	GC02P119	2.5
4450	FKTN	Fukutin	Protein Co	39	GC09P105	2.5
4451	GLIS3	GLIS Famil	Protein Co	39	GC09M003	2.5
4452	ARHGDIA	Rho GDP D	Protein Co	47	GC17M081	2.5
4453	RFC4	Replicatior	Protein Co	41	GC03M186	2.5
4454	KCNQ3	Potassium	Protein Co	45	GC08M132	2.5
4455	LRSAM1	Leucine Ri	Protein Co	39	GC09P127	2.5
4456	RALGAPA1	Ral GTPase	Protein Co	39	GC14M035	2.5
4457	VPS13B	Vacuolar P	Protein Co	39	GC08P099	2.5
4458	STOM	Stomatin	Protein Co	39	GC09M121	2.49
4459	ZDBF2	Zinc Finge	Protein Co	32	GC02P206	2.49
4460	PPP1R15B	Protein Ph	Protein Co	37	GC01M204	2.49
4461	MGAT3-AS	MGAT3 An	RNA Gene	13	GC22M045	2.49
4462	PEX16	Peroxisom	Protein Co	38	GC11M061	2.49
4463	ATF1	Activating	Protein Co	46	GC12P050	2.49
4464	ADRA1B	Adrenocep	Protein Co	46	GC05P159	2.49
4465	NOP53	NOP53 Rib	Protein Co	28	GC19P047	2.49
4466	PRKCSH	Protein Kir	Protein Co	43	GC19P011	2.49
4467	MYOG	Myogenin	Protein Co	39	GC01M203	2.49
4468	ITPKC	Inositol-Tri	Protein Co	43	GC19P040	2.49
4469	ST3GAL4	ST3 Beta-C	Protein Co	41	GC11P126	2.49
4470	CERT1	Ceramide	Protein Co	33	GC05M075	2.48

4471	RBBP8	RB Bindinç	Protein Co	44	GC18P022	2.48
4472	SLC44A2	Solute Car	Protein Co	40	GC19P010	2.48
4473	SRP68	Signal Rec	Protein Co	37	GC17M076	2.48
4474	ARCN1	Archain 1	Protein Co	41	GC11P118	2.48
4475	TP53BP2	Tumor Pro	Protein Co	42	GC01M223	2.48
4476	STX4	Syntaxin 4	Protein Co	42	GC16P031	2.48
4477	PKP4	Plakophilir	Protein Co	40	GC02P158	2.48
4478	ARHGEF7	Rho Guani	Protein Co	43	GC13P111	2.48
4479	DCTN2	Dynactin S	Protein Co	41	GC12M057	2.48
4480	SPRY2	Sprouty R1	Protein Co	46	GC13M080	2.48
4481	ACTR3	Actin Relat	Protein Co	42	GC02P113	2.48
4482	LAMP2	Lysosomal	Protein Co	44	GC0XM120	2.48
4483	ADAM19	ADAM Me	Protein Co	41	GC05M157	2.48
4484	SLC29A2	Solute Car	Protein Co	45	GC11M066	2.48
4485	KIF3B	Kinesin Fa	Protein Co	39	GC20P032	2.48
4486	TBXAS1	Thromboxa	Protein Co	50	GC07P139	2.48
4487	HNRNPK	Heterogen	Protein Co	45	GC09M084	2.48
4488	ABCC9	ATP Bindir	Protein Co	44	GC12M021	2.48
4489	PLXNA2	Plexin A2	Protein Co	42	GC01M208	2.47
4490	CALCRL	Calcitonin	Protein Co	43	GC02M187	2.47
4491	PCSK6	Proprotein	Protein Co	40	GC15M101	2.47
4492	IRF9	Interferon	Protein Co	43	GC14P024	2.47
4493	TNFRSF21	TNF Recep	Protein Co	45	GC06M047	2.47
4494	CIRBP	Cold Induc	Protein Co	38	GC19P001	2.47
4495	SHROOM3	Shroom Fa	Protein Co	37	GC04P076	2.47
4496	CYTH1	Cytohesin	Protein Co	41	GC17M078	2.47
4497	NCR2	Natural Cy	Protein Co	39	GC06P047	2.47
4498	CBSL	Cystathion	Protein Co	17	GC21M006	2.47
4499	CUL3	Cullin 3	Protein Co	46	GC02M224	2.47
4500	ACTN3	Actinin Alp	Protein Co	39	GC11P066	2.47
4501	CUX1	Cut Like H	Protein Co	43	GC07P101	2.47
4502	PLXNC1	Plexin C1	Protein Co	39	GC12P094	2.47
4503	DCX	Doublecor	Protein Co	45	GC0XM111	2.46
4504	SNIP1	Smad Nucl	Protein Co	39	GC01M037	2.46
4505	PPP1R1B	Protein Ph	Protein Co	43	GC17P039	2.46
4506	NRIP1	Nuclear Re	Protein Co	43	GC21M014	2.46
4507	HAS1	Hyaluronai	Protein Co	39	GC19M051	2.46
4508	SPRY1	Sprouty R1	Protein Co	42	GC04P123	2.46
4509	CORT	Cortistatin	Protein Co	36	GC01P010	2.46
4510	EIF4G3	Eukaryotic	Protein Co	42	GC01M020	2.46
4511	CORIN	Corin, Seri	Protein Co	44	GC04M047	2.46
4512	HSPA1B	Heat Shocl	Protein Co	40	GC06P047	2.46
4513	CAV3	Caveolin 3	Protein Co	43	GC03P008	2.46

4514	NCAN	Neurocan	Protein Co	41	GC19P023	2.46
4515	NFASC	Neurofasci	Protein Co	41	GC01P204	2.45
4516	AKAP13	A-Kinase A	Protein Co	44	GC15P085	2.45
4517	ADAMTS1	ADAM Me	Protein Co	44	GC21M026	2.45
4518	CACNA1H	Calcium V	Protein Co	50	GC16P001	2.45
4519	ABCA7	ATP Bindir	Protein Co	43	GC19P001	2.45
4520	CENPT	Centromer	Protein Co	37	GC16M067	2.45
4521	FKBP5	FKBP Proly	Protein Co	45	GC06M042	2.45
4522	HMGN4	High Mobi	Protein Co	33	GC06P026	2.45
4523	ECRG4	ECRG4 Au	Protein Co	27	GC02P106	2.45
4524	KLHL24	Kelch Like	Protein Co	35	GC03P183	2.45
4525	PRPF8	Pre-MRNA	Protein Co	41	GC17M001	2.45
4526	MIR151A	MicroRNA	RNA Gene	17	GC08M140	2.45
4527	RNF31	Ring Finge	Protein Co	41	GC14P024	2.45
4528	PKP2	Plakophilir	Protein Co	45	GC12M032	2.45
4529	CIDEC	Cell Death	Protein Co	41	GC03M009	2.44
4530	RAPGEF2	Rap Guani	Protein Co	41	GC04P159	2.44
4531	PTPRR	Protein Ty	Protein Co	40	GC12M070	2.44
4532	HMGB2	High Mobi	Protein Co	42	GC04M173	2.44
4533	CARS2	Cysteinyl-T	Protein Co	41	GC13M110	2.44
4534	KYNU	Kynurenina	Protein Co	46	GC02P142	2.44
4535	GRM5	Glutamate	Protein Co	47	GC11M088	2.44
4536	SLC15A4	Solute Car	Protein Co	39	GC12M128	2.44
4537	MIR331	MicroRNA	RNA Gene	18	GC12P095	2.44
4538	TNIK	TRAF2 Anc	Protein Co	44	GC03M171	2.44
4539	PPP1R15A	Protein Ph	Protein Co	40	GC19P048	2.44
4540	TAF1	TATA-Box	Protein Co	45	GC0XP071	2.44
4541	CCDC65	Coiled-Coi	Protein Co	36	GC12P048	2.43
4542	DRC1	Dynein Re	Protein Co	32	GC02P026	2.43
4543	TUBA4A	Tubulin Al	Protein Co	45	GC02M219	2.43
4544	MIP	Major Intri	Protein Co	41	GC12M056	2.43
4545	INPPL1	Inositol Po	Protein Co	49	GC11P072	2.43
4546	MIR19B1	MicroRNA	RNA Gene	17	GC13P091	2.43
4547	CACNB4	Calcium V	Protein Co	45	GC02M151	2.43
4548	SNCG	Synuclein	Protein Co	42	GC10P086	2.43
4549	SNX27	Sorting Ne	Protein Co	39	GC01P151	2.43
4550	E2F5	E2F Transc	Protein Co	40	GC08P085	2.43
4551	PDIA3	Protein Dis	Protein Co	44	GC15P043	2.43
4552	CDC20	Cell Divisic	Protein Co	44	GC01P043	2.43
4553	GALNT1	Polypeptid	Protein Co	41	GC18P035	2.43
4554	NSMCE2	NSE2 (MM	Protein Co	39	GC08P125	2.43
4555	IL13RA1	Interleukin	Protein Co	42	GC0XP118	2.43
4556	RGMA	Repulsive	Protein Co	41	GC15M093	2.43

4557	LINC00261	Long Inter	RNA Gene	18	GC20M022	2.43
4558	LGALS8	Galectin 8	Protein Co	41	GC01P236	2.43
4559	TNFRSF12A	TNF Recep	Protein Co	44	GC16P003	2.43
4560	DNAAF1	Dynein Axo	Protein Co	36	GC16P084	2.43
4561	TRIR	Telomeras	Protein Co	25	GC19M012	2.43
4562	WDR75	WD Repea	Protein Co	34	GC02P189	2.42
4563	GIPR	Gastric Inh	Protein Co	44	GC19P045	2.42
4564	PRDX4	Peroxiredo	Protein Co	43	GC0XP023	2.42
4565	DNAAF6	Dynein Axo	Protein Co	28	GC0XP107	2.42
4566	GNA15	G Protein S	Protein Co	41	GC19P003	2.42
4567	TRPM4	Transient F	Protein Co	45	GC19P049	2.42
4568	KALRN	Kalirin Rho	Protein Co	41	GC03P124	2.42
4569	PYGB	Glycogen I	Protein Co	44	GC20P025	2.42
4570	RBCK1	RANBP2-T	Protein Co	43	GC20P000	2.42
4571	DNAJB2	DnaJ Heat	Protein Co	43	GC02P219	2.42
4572	HSPB6	Heat Shoc	Protein Co	39	GC19M042	2.42
4573	ALDH16A1	Aldehyde I	Protein Co	36	GC19P049	2.42
4574	PSMA2	Proteasom	Protein Co	42	GC07M042	2.42
4575	PANK2-AS	PANK2 An	RNA Gene	8	GC20M003	2.42
4576	HGFAC	HGF Activa	Protein Co	40	GC04P003	2.42
4577	KTN1	Kinectin 1	Protein Co	39	GC14P055	2.41
4578	TRIB1	Tribbles Ps	Protein Co	37	GC08P125	2.41
4579	AHSG	Alpha 2-HS	Protein Co	44	GC03P186	2.41
4580	MTAP	Methylthio	Protein Co	47	GC09P021	2.41
4581	BICC1	BicC Famil	Protein Co	36	GC10P058	2.41
4582	GALNT3	Polypeptid	Protein Co	45	GC02M165	2.41
4583	HAS2	Hyaluronai	Protein Co	40	GC08M121	2.41
4584	MLEC	Malectin	Protein Co	37	GC12P120	2.4
4585	KDM2B	Lysine Den	Protein Co	41	GC12M121	2.4
4586	YEATS2	YEATS Dor	Protein Co	36	GC03P183	2.4
4587	RETREG2	Reticuloph	Protein Co	24	GC02P219	2.4
4588	TAF8	TATA-Box	Protein Co	37	GC06P042	2.4
4589	HTRA1	HtrA Serin	Protein Co	43	GC10P122	2.4
4590	PFN2	Profilin 2	Protein Co	41	GC03M149	2.4
4591	MAP3K8	Mitogen-A	Protein Co	47	GC10P030	2.4
4592	GM2A	GM2 Gang	Protein Co	43	GC05P151	2.4
4593	CACNA1E	Calcium Vo	Protein Co	46	GC01P181	2.4
4594	RAD9A	RAD9 Che	Protein Co	40	GC11P067	2.4
4595	CTH	Cystathion	Protein Co	50	GC01P070	2.4
4596	BFAR	Bifunctiona	Protein Co	37	GC16P014	2.4
4597	EFHC1	EF-Hand D	Protein Co	40	GC06P052	2.4
4598	USP36	Ubiquitin S	Protein Co	37	GC17M078	2.4
4599	NDUFA11	NADH:Ubi	Protein Co	37	GC19M005	2.4

4600	MCAM	Melanoma	Protein Co	40	GC11M119	2.4
4601	MIR146B	MicroRNA	RNA Gene	19	GC10P102	2.39
4602	UBE2B	Ubiquitin (	Protein Co	45	GC05P134	2.39
4603	F8A1	Coagulatio	Protein Co	34	GC0XP154	2.39
4604	SEMA3F	Semaphori	Protein Co	41	GC03P050	2.39
4605	GABARAPL	GABA Type	Protein Co	44	GC16P075	2.39
4606	PODXL	Podocalyxi	Protein Co	41	GC07M131	2.39
4607	ALDH6A1	Aldehyde I	Protein Co	46	GC14M074	2.39
4608	MTFMT	Mitochond	Protein Co	41	GC15M065	2.39
4609	TBC1D23	TBC1 Dom	Protein Co	36	GC03P100	2.39
4610	A2ML1	Alpha-2-M	Protein Co	39	GC12P008	2.39
4611	VMP1	Vacuole M	Protein Co	37	GC17P059	2.39
4612	ATR	ATR Serine	Protein Co	51	GC03M142	2.39
4613	PRICKLE1	Prickle Pla	Protein Co	42	GC12M042	2.39
4614	IL17D	Interleukin	Protein Co	38	GC13P020	2.39
4615	BRPF1	Bromodom	Protein Co	41	GC03P009	2.39
4616	ANXA4	Annexin A4	Protein Co	44	GC02P069	2.39
4617	C19orf48	Chromosol	Protein Co	33	GC19M050	2.38
4618	IVNS1ABP	Influenza \	Protein Co	39	GC01M185	2.38
4619	RASSF10	Ras Associ	Protein Co	31	GC11P012	2.38
4620	PPA1	Inorganic I	Protein Co	40	GC10M070	2.38
4621	CYFIP1	Cytoplasm	Protein Co	41	GC15M022	2.38
4622	RBM28	RNA Bindi	Protein Co	39	GC07M128	2.38
4623	EPM2A	EPM2A Gl	Protein Co	43	GC06M145	2.38
4624	TARS1	Threonyl-T	Protein Co	36	GC05P033	2.38
4625	IRAK4	Interleukin	Protein Co	48	GC12P043	2.38
4626	BVES	Blood Ves	Protein Co	40	GC06M105	2.38
4627	CXCL14	C-X-C Mot	Protein Co	39	GC05M135	2.38
4628	PTPRQ	Protein Ty	Protein Co	33	GC12P080	2.38
4629	HMCN1	Hemicentir	Protein Co	38	GC01P185	2.38
4630	SYNJ2	Synaptojar	Protein Co	40	GC06P157	2.38
4631	EXT2	Exostosin (	Protein Co	46	GC11P044	2.38
4632	MTMR2	Myotubula	Protein Co	43	GC11M095	2.38
4633	PPP1R12A	Protein Ph	Protein Co	42	GC12M079	2.37
4634	POMT2	Protein O-	Protein Co	43	GC14M077	2.37
4635	UNG	Uracil DNA	Protein Co	46	GC12P109	2.37
4636	SMN2	Survival Of	Protein Co	42	GC05P070	2.37
4637	ARHGEF10	Rho Guani	Protein Co	43	GC08P001	2.37
4638	CTDP1	CTD Phosp	Protein Co	41	GC18P079	2.37
4639	NPR3	Natriuretic	Protein Co	44	GC05P032	2.37
4640	ING1	Inhibitor C	Protein Co	43	GC13P110	2.37
4641	GDI1	GDP Disso	Protein Co	43	GC0XP154	2.37
4642	FLOT2	Flotillin 2	Protein Co	41	GC17M029	2.37

4643	ZCCHC14	Zinc Finge	Protein Co	33	GC16M087	2.37
4644	PVR	PVR Cell A	Protein Co	44	GC19P044	2.37
4645	SLC12A7	Solute Car	Protein Co	44	GC05M001	2.37
4646	TAF2	TATA-Box	Protein Co	40	GC08M119	2.37
4647	GSTO1	Glutathion	Protein Co	43	GC10P104	2.37
4648	TK2	Thymidine	Protein Co	42	GC16M066	2.37
4649	ARHGEF1	Rho Guani	Protein Co	44	GC19P041	2.36
4650	GGH	Gamma-Gl	Protein Co	42	GC08M063	2.36
4651	MPLKIP	M-Phase S	Protein Co	36	GC07M040	2.36
4652	ORAI1	ORAI Calci	Protein Co	44	GC12P122	2.36
4653	PRKCG	Protein Kir	Protein Co	51	GC19P053	2.36
4654	ALDH3A1	Aldehyde I	Protein Co	44	GC17M019	2.36
4655	NDUFS1	NADH:Ubi	Protein Co	45	GC02M206	2.36
4656	CHD9	Chromodo	Protein Co	37	GC16P053	2.36
4657	HMGCL	3-Hydroxy	Protein Co	46	GC01M023	2.36
4658	XPC	XPC Comp	Protein Co	45	GC03M015	2.36
4659	CCDC88A	Coiled-Coi	Protein Co	40	GC02M055	2.36
4660	HLA-DMB	Major Hist	Protein Co	41	GC06M032	2.36
4661	WAC	WW Doma	Protein Co	39	GC10P028	2.36
4662	KLC1	Kinesin Lig	Protein Co	41	GC14P104	2.36
4663	MLIP	Muscular L	Protein Co	33	GC06P053	2.36
4664	RNASET2	Ribonuclea	Protein Co	44	GC06M166	2.36
4665	KAT7	Lysine Ace	Protein Co	42	GC17P049	2.35
4666	TSPAN12	Tetraspani	Protein Co	43	GC07M120	2.35
4667	NKX6-1	NK6 Home	Protein Co	41	GC04M084	2.35
4668	PARD3B	Par-3 Fami	Protein Co	35	GC02P204	2.35
4669	RBX1	Ring-Box 1	Protein Co	42	GC22P040	2.35
4670	MED1	Mediator (	Protein Co	41	GC17M039	2.35
4671	PPP1R8	Protein Ph	Protein Co	39	GC01P027	2.35
4672	CAMK2B	Calcium/C	Protein Co	50	GC07M044	2.35
4673	NECTIN1	Nectin Cel	Protein Co	37	GC11M119	2.35
4674	GALE	UDP-Galac	Protein Co	45	GC01M023	2.35
4675	ATP11A	ATPase Ph	Protein Co	41	GC13P112	2.35
4676	NCAPD2	Non-SMC	Protein Co	40	GC12P006	2.35
4677	LRRC6	Leucine Ri	Protein Co	37	GC08M132	2.35
4678	CUL1	Cullin 1	Protein Co	44	GC07P148	2.35
4679	LRAT	Lecithin Re	Protein Co	44	GC04P154	2.35
4680	MIR509-1	MicroRNA	RNA Gene	15	GC0XM147	2.35
4681	SRSF7	Serine Anc	Protein Co	39	GC02M038	2.35
4682	CMKLR1	Chemerin	Protein Co	40	GC12M108	2.35
4683	APOC1	Apolipopro	Protein Co	40	GC19P044	2.35
4684	JHY	Junctional	Protein Co	25	GC11P122	2.35
4685	RAB31	RAB31, Me	Protein Co	40	GC18P009	2.35

4686	IDUA	Alpha-L-Id	Protein Co	42	GC04P000	2.35
4687	LGALS9C	Galectin 9(	Protein Co	32	GC17P018	2.34
4688	WTIP	WT1 Intera	Protein Co	35	GC19P034	2.34
4689	ACY1	Aminoacyl	Protein Co	46	GC03P051	2.34
4690	LIAS	Lipoic Acic	Protein Co	44	GC04P039	2.34
4691	USP4	Ubiquitin S	Protein Co	43	GC03M049	2.34
4692	EIF4A2	Eukaryotic	Protein Co	45	GC03P186	2.34
4693	ADAMTS9	ADAM Me	Protein Co	37	GC03M064	2.34
4694	SIKE1	Suppresso	Protein Co	35	GC01M114	2.34
4695	ZDHHC8	Zinc Finge	Protein Co	39	GC22P020	2.34
4696	C4BPA	Compleme	Protein Co	40	GC01P207	2.34
4697	PIM1	Pim-1 Prot	Protein Co	50	GC06P047	2.34
4698	SSB	Small RNA	Protein Co	42	GC02P169	2.34
4699	AHCY	Adenosylh	Protein Co	50	GC20M034	2.34
4700	ACO2	Aconitase	Protein Co	46	GC22P041	2.34
4701	MEF2A	Myocyte E	Protein Co	47	GC15P099	2.34
4702	FKBP4	FKBP Proly	Protein Co	44	GC12P002	2.34
4703	TUFM	Tu Transla	Protein Co	44	GC16M028	2.34
4704	MAP3K13	Mitogen-A	Protein Co	42	GC03P185	2.33
4705	SSTR5	Somatosta	Protein Co	41	GC16P001	2.33
4706	FMN2	Formin 2	Protein Co	40	GC01P240	2.33
4707	CERS6	Ceramide S	Protein Co	37	GC02P168	2.33
4708	NHLRC1	NHL Repea	Protein Co	40	GC06M018	2.33
4709	MFN1	Mitofusin 1	Protein Co	41	GC03P179	2.33
4710	PTPRA	Protein Ty	Protein Co	45	GC20P002	2.33
4711	NHS	NHS Actin	Protein Co	37	GC0XP017	2.33
4712	PIK3C3	Phosphatic	Protein Co	49	GC18P041	2.32
4713	LHX8	LIM Home	Protein Co	37	GC01P075	2.32
4714	PEX14	Peroxisom	Protein Co	43	GC01P010	2.32
4715	TRADD	TNFRSF1A	Protein Co	43	GC16M067	2.32
4716	SEPHS2	Selenopho	Protein Co	37	GC16M030	2.32
4717	TUBA1C	Tubulin Al	Protein Co	41	GC12P049	2.32
4718	EIF2AK4	Eukaryotic	Protein Co	44	GC15P039	2.32
4719	TSPAN3	Tetraspani	Protein Co	36	GC15M077	2.32
4720	MYZAP	Myocardia	Protein Co	31	GC15P057	2.32
4721	RPL7A	Ribosomal	Protein Co	42	GC09P133	2.32
4722	ITM2B	Integral M	Protein Co	43	GC13P048	2.32
4723	PNPLA2	Patatin Lik	Protein Co	44	GC11P000	2.32
4724	ARHGEF2	Rho/Rac G	Protein Co	45	GC01M155	2.32
4725	PPIC	Peptidylpr	Protein Co	41	GC05M123	2.32
4726	CACNA2D2	Calcium V	Protein Co	41	GC03M050	2.32
4727	AP4E1	Adaptor R	Protein Co	38	GC15P050	2.31
4728	NCOA2	Nuclear Re	Protein Co	44	GC08M070	2.31

4729	GREM2	Gremlin 2,	Protein Co	40	GC01M240	2.31
4730	MARVELD3	MARVEL D	Protein Co	34	GC16P071	2.31
4731	APH1A	Aph-1 Hor	Protein Co	43	GC01M150	2.31
4732	METAP2	Methionyl	Protein Co	44	GC12P095	2.31
4733	CPE	Carboxype	Protein Co	44	GC04P165	2.31
4734	CCL14	C-C Motif	Protein Co	36	GC17M036	2.31
4735	PDCD6IP	Programm	Protein Co	42	GC03P033	2.31
4736	CLEC3B	C-Type Le	Protein Co	40	GC03P045	2.31
4737	ATXN1	Ataxin 1	Protein Co	43	GC06M016	2.31
4738	FTMT	Ferritin Mi	Protein Co	38	GC05P121	2.31
4739	PATJ	PATJ Crum	Protein Co	31	GC01P061	2.31
4740	EML1	EMAP Like	Protein Co	40	GC14P099	2.31
4741	ETV4	ETS Varian	Protein Co	43	GC17M043	2.31
4742	REXO2	RNA Exon	Protein Co	37	GC11P114	2.3
4743	PRDX2	Peroxiredo	Protein Co	46	GC19M012	2.3
4744	SLITRK5	SLIT And N	Protein Co	37	GC13P087	2.3
4745	PATE1	Prostate A	Protein Co	31	GC11P125	2.3
4746	MT-RNR2	Mitochond	RNA Gene	18	GCMTP001	2.3
4747	MIR323A	MicroRNA	RNA Gene	16	GC14P104	2.3
4748	GPR1-AS	GPR1 Anti	RNA Gene	12	GC02P206	2.3
4749	MT-TG	Mitochond	RNA Gene	12	GCMTP009	2.3
4750	DGCR12	DiGeorge S	RNA Gene	9	GC22U900	2.3
4751	LOC10678	Tenascin X	Biological	1	GC06P047	2.3
4752	SART1	Spliceoson	Protein Co	40	GC11P065	2.3
4753	OTULIN	OTU Deub	Protein Co	38	GC05P014	2.3
4754	RPS26	Ribosomal	Protein Co	42	GC12P056	2.3
4755	GBP1	Guanylate	Protein Co	41	GC01M089	2.3
4756	LPAR5	Lysophosp	Protein Co	41	GC12M006	2.3
4757	ARID5B	AT-Rich In	Protein Co	40	GC10P061	2.3
4758	STAMBP	STAM Binc	Protein Co	45	GC02P073	2.3
4759	PTGIR	Prostaglan	Protein Co	47	GC19M046	2.3
4760	SMN1	Survival Of	Protein Co	41	GC05P070	2.3
4761	CHRNA4	Cholinergi	Protein Co	48	GC20M063	2.3
4762	ONECUT2	One Cut H	Protein Co	35	GC18P057	2.3
4763	PTK7	Protein Ty	Protein Co	43	GC06P043	2.3
4764	GFI1	Growth Fa	Protein Co	41	GC01M092	2.3
4765	CRABP2	Cellular Re	Protein Co	43	GC01M156	2.3
4766	GYS1	Glycogen S	Protein Co	49	GC19M048	2.29
4767	UFM1	Ubiquitin F	Protein Co	40	GC13P038	2.29
4768	VIPAS39	VPS33B Int	Protein Co	37	GC14M077	2.29
4769	NME8	NME/NM2	Protein Co	40	GC07P037	2.29
4770	DNAAF3	Dynein Axo	Protein Co	36	GC19M055	2.29
4771	CFAP300	Cilia And F	Protein Co	26	GC11P102	2.29

4772	BDH1	3-Hydroxyl	Protein Co	43	GC03M197	2.29
4773	CLCN3	Chloride V	Protein Co	41	GC04P169	2.29
4774	SYNPO	Synaptopo	Protein Co	38	GC05P150	2.29
4775	MIEF2	Mitochond	Protein Co	33	GC17P018	2.29
4776	FCRL3	Fc Receptc	Protein Co	36	GC01M157	2.29
4777	ATXN3	Ataxin 3	Protein Co	44	GC14M094	2.29
4778	LLGL2	LLGL Scrib	Protein Co	39	GC17P075	2.29
4779	NDUFAF1	NADH:Ubi	Protein Co	41	GC15M041	2.29
4780	PRDX6	Peroxiredo	Protein Co	46	GC01P173	2.29
4781	APLN	Apelin	Protein Co	37	GC0XM129	2.28
4782	KIF11	Kinesin Fa	Protein Co	47	GC10P092	2.28
4783	UTRN	Utrophin	Protein Co	40	GC06P144	2.28
4784	OTUD7B	OTU Deub	Protein Co	35	GC01M149	2.28
4785	FLAD1	Flavin Ade	Protein Co	41	GC01P154	2.28
4786	MAPK13	Mitogen-A	Protein Co	48	GC06P047	2.28
4787	NDUFS7	NADH:Ubi	Protein Co	46	GC19P001	2.28
4788	TIMD4	T Cell Imm	Protein Co	36	GC05M156	2.28
4789	P2RY4	Pyrimidine	Protein Co	43	GC0XM070	2.28
4790	BCL2L14	BCL2 Like	Protein Co	38	GC12P012	2.28
4791	MEPE	Matrix Extr	Protein Co	35	GC04P087	2.28
4792	CHD1	Chromodo	Protein Co	45	GC05M098	2.28
4793	C1GALT1C	C1GALT1 S	Protein Co	37	GC0XM120	2.27
4794	SLC2A13	Solute Car	Protein Co	41	GC12M039	2.27
4795	TGM3	Transgluta	Protein Co	42	GC20P002	2.27
4796	CD300A	CD300a M	Protein Co	40	GC17P074	2.27
4797	JMJD6	Jumonji D	Protein Co	42	GC17M076	2.27
4798	TAPBP	TAP Bindir	Protein Co	43	GC06M033	2.27
4799	EHD2	EH Domair	Protein Co	39	GC19P047	2.27
4800	VPS13D	Vacuolar P	Protein Co	34	GC01P012	2.27
4801	RFK	Riboflavin	Protein Co	39	GC09M076	2.27
4802	ROR2	Receptor T	Protein Co	48	GC09M091	2.27
4803	NR1D1	Nuclear Re	Protein Co	47	GC17M040	2.27
4804	CSRP1	Cysteine A	Protein Co	41	GC01M201	2.27
4805	SPTB	Spectrin B	Protein Co	40	GC14M064	2.27
4806	ATPSCKMT	ATP Synth	Protein Co	27	GC05M010	2.27
4807	ATG3	Autophagy	Protein Co	41	GC03M112	2.27
4808	KIAA0319	KIAA0319	Protein Co	39	GC06M024	2.27
4809	SCN3B	Sodium Vc	Protein Co	44	GC11M123	2.27
4810	TUBG1	Tubulin Ga	Protein Co	47	GC17P042	2.27
4811	NLRX1	NLR Family	Protein Co	40	GC11P119	2.26
4812	NUPR1	Nuclear Pr	Protein Co	35	GC16M028	2.26
4813	TNXA	Tenascin X	Pseudoger	23	GC06M032	2.26
4814	RCC1	Regulator	Protein Co	40	GC01P028	2.26

4815	KLF10	Kruppel Lil	Protein Co	42	GC08M102	2.26
4816	ABCB6	ATP Bindir	Protein Co	46	GC02M219	2.26
4817	FKBP10	FKBP Proly	Protein Co	40	GC17P041	2.26
4818	S100A11	S100 Calci	Protein Co	41	GC01M152	2.26
4819	ENSG00000268047		RNA Gene	8	GC19M049	2.26
4820	OS9	OS9 Endop	Protein Co	40	GC12P057	2.26
4821	NOLC1	Nucleolar /	Protein Co	38	GC10P102	2.26
4822	PPP1R14A	Protein Ph	Protein Co	40	GC19M038	2.26
4823	SUPT16H	SPT16 Hor	Protein Co	37	GC14M021	2.26
4824	GRK6	G Protein-(	Protein Co	45	GC05P177	2.26
4825	TNFAIP2	TNF Alpha	Protein Co	39	GC14P104	2.26
4826	AGK	Acylglycer(	Protein Co	41	GC07P141	2.26
4827	PSMD9	Proteasom	Protein Co	43	GC12P122	2.26
4828	ZMYND10	Zinc Finge	Protein Co	39	GC03M050	2.26
4829	CD1E	CD1e Mole	Protein Co	39	GC01P158	2.26
4830	BHLHE40	Basic Helix	Protein Co	41	GC03P004	2.26
4831	EIF5	Eukaryotic	Protein Co	41	GC14P103	2.25
4832	SHANK2	SH3 And N	Protein Co	41	GC11M070	2.25
4833	MAX	MYC Asso(	Protein Co	48	GC14M065	2.25
4834	TDP1	Tyrosyl-DN	Protein Co	44	GC14P089	2.25
4835	NAT8L	N-Acetyltra	Protein Co	37	GC04P002	2.25
4836	MYH14	Myosin He	Protein Co	45	GC19P050	2.25
4837	PRG2	Proteoglyc	Protein Co	40	GC11M057	2.25
4838	DNAH9	Dynein Axo	Protein Co	39	GC17P011	2.25
4839	PSIP1	PC4 And S	Protein Co	39	GC09M015	2.25
4840	NRF1	Nuclear Re	Protein Co	43	GC07P129	2.25
4841	GNAI2	G Protein S	Protein Co	47	GC03P050	2.24
4842	AADAC	Arylacetam	Protein Co	41	GC03P151	2.24
4843	KLK11	Kallikrein F	Protein Co	41	GC19M051	2.24
4844	GRK5	G Protein-(	Protein Co	43	GC10P119	2.24
4845	SLC30A7	Solute Car	Protein Co	39	GC01P100	2.24
4846	ARHGAP45	Rho GTPas	Protein Co	29	GC19P001	2.24
4847	AQP7	Aquaporin	Protein Co	44	GC09M033	2.24
4848	LIMS1	LIM Zinc F	Protein Co	40	GC02P108	2.24
4849	RPL7AP70	Ribosomal	Pseudoger	8	GC22P019	2.24
4850	BCL3	BCL3 Trans	Protein Co	41	GC19P044	2.24
4851	NDRG3	NDRG Fam	Protein Co	37	GC20M036	2.24
4852	SFPQ	Splicing Fa	Protein Co	43	GC01M035	2.24
4853	VAC14	VAC14 Co	Protein Co	43	GC16M070	2.24
4854	C19orf33	Chromoso	Protein Co	30	GC19P038	2.24
4855	CDC6	Cell Divisic	Protein Co	45	GC17P040	2.24
4856	GPS1	G Protein I	Protein Co	37	GC17P082	2.24
4857	CTU1	Cytosolic T	Protein Co	34	GC19M051	2.24

4858	DEPTOR	DEP Doma	Protein Co	40	GC08P119	2.24
4859	STEAP4	STEAP4 M	Protein Co	40	GC07M088	2.24
4860	AATF	Apoptosis	Protein Co	40	GC17P036	2.24
4861	IER3IP1	Immediate	Protein Co	38	GC18M047	2.24
4862	TPP1	Tripeptidyl	Protein Co	43	GC11M006	2.24
4863	EIF6	Eukaryotic	Protein Co	40	GC20M035	2.24
4864	TGFB1I1	Transformi	Protein Co	41	GC16P031	2.23
4865	USP8	Ubiquitin S	Protein Co	48	GC15P050	2.23
4866	STX16	Syntaxin 1	Protein Co	42	GC20P058	2.23
4867	SMOX	Spermine (	Protein Co	39	GC20P004	2.23
4868	BRAT1	BRCA1 Ass	Protein Co	38	GC07M002	2.23
4869	CCNC	Cyclin C	Protein Co	40	GC06M099	2.23
4870	CENPA	Centromer	Protein Co	39	GC02P026	2.23
4871	ATP2B3	ATPase Pla	Protein Co	47	GC0XP153	2.23
4872	SULF2	Sulfatase 2	Protein Co	40	GC20M047	2.23
4873	MPDU1	Mannose-I	Protein Co	41	GC17P007	2.23
4874	ARHGAP26	Rho GTPas	Protein Co	44	GC05P142	2.23
4875	VPS51	VPS51 Sub	Protein Co	35	GC11P065	2.23
4876	KCTD7	Potassium	Protein Co	38	GC07P066	2.22
4877	CCS	Copper Ch	Protein Co	42	GC11P066	2.22
4878	ABT1	Activator (	Protein Co	35	GC06P028	2.22
4879	NPTX1	Neuronal I	Protein Co	37	GC17M080	2.22
4880	PDP1	Pyruvate D	Protein Co	45	GC08P093	2.22
4881	RAP2C	RAP2C, M	Protein Co	37	GC0XM132	2.22
4882	SEMA7A	Semaphori	Protein Co	42	GC15M074	2.22
4883	KDM6B	Lysine Den	Protein Co	42	GC17P007	2.22
4884	DYNLRB1	Dynein Lig	Protein Co	39	GC20P034	2.22
4885	CMIP	C-Maf Indu	Protein Co	35	GC16P081	2.22
4886	MBD1	Methyl-Cp	Protein Co	40	GC18M050	2.22
4887	ZBP1	Z-DNA Bin	Protein Co	39	GC20M057	2.22
4888	EXT1	Exostosin (	Protein Co	48	GC08M117	2.21
4889	SRSF5	Serine Anc	Protein Co	38	GC14P069	2.21
4890	POLH	DNA Polyr	Protein Co	48	GC06P043	2.21
4891	IQSEC1	IQ Motif A	Protein Co	42	GC03M015	2.21
4892	TEAD4	TEA Doma	Protein Co	41	GC12P002	2.21
4893	DNAJB13	DnaJ Heat	Protein Co	35	GC11P073	2.21
4894	JAKMIP1	Janus Kina	Protein Co	37	GC04M006	2.21
4895	DNAJC19	DnaJ Heat	Protein Co	41	GC03M180	2.21
4896	CALM3	Calmodulin	Protein Co	42	GC19P046	2.21
4897	MDH1	Malate De	Protein Co	45	GC02P063	2.21
4898	FDPS	Farnesyl D	Protein Co	45	GC01P155	2.21
4899	XYLT1	Xylosyltran	Protein Co	43	GC16M017	2.21
4900	FAM3D	FAM3 Met	Protein Co	37	GC03M058	2.21

4901	NDUFB10	NADH:Ubi	Protein Co	41	GC16P002	2.21
4902	BARD1	BRCA1 Ass	Protein Co	46	GC02M214	2.21
4903	UGT2B17	UDP Glucu	Protein Co	40	GC04M068	2.21
4904	ZBTB33	Zinc Finge	Protein Co	36	GC0XP120	2.21
4905	ADRA1D	Adrenocep	Protein Co	45	GC20M004	2.2
4906	AQP8	Aquaporin	Protein Co	37	GC16P026	2.2
4907	MED29	Mediator (	Protein Co	34	GC19P039	2.2
4908	MG828730-062		RNA Gene	4	GC06M031	2.2
4909	NME2	NME/NM2	Protein Co	46	GC17P051	2.2
4910	TFAM	Transcripti	Protein Co	43	GC10P058	2.2
4911	FSTL1	Follistatin	Protein Co	41	GC03M120	2.2
4912	MRPS7	Mitochonc	Protein Co	39	GC17P075	2.2
4913	VPS16	VPS16 Cor	Protein Co	35	GC20P002	2.2
4914	KHSRP	KH-Type S	Protein Co	41	GC19M006	2.2
4915	TP53COR1	Tumor Pro	RNA Gene	8	GC06U903	2.2
4916	ELOA	Elongin A	Protein Co	29	GC01P023	2.2
4917	MAFB	MAF BZIP	Protein Co	43	GC20M040	2.2
4918	CILP	Cartilage I	Protein Co	40	GC15M065	2.2
4919	H1-2	H1.2 Linke	Protein Co	32	GC06M026	2.2
4920	MAP3K15	Mitogen-A	Protein Co	36	GC0XM019	2.2
4921	CD70	CD70 Mol	Protein Co	43	GC19M006	2.2
4922	SMPD3	Sphingom	Protein Co	41	GC16M068	2.2
4923	DGCR11	DiGeorge	RNA Gene	17	GC22M019	2.2
4924	RGN	Regucalcin	Protein Co	39	GC0XP047	2.2
4925	XPA	XPA, DNA	Protein Co	45	GC09M097	2.2
4926	DHX30	DExH-Box	Protein Co	40	GC03P047	2.19
4927	BTLA	B And T Ly	Protein Co	39	GC03M112	2.19
4928	STX2	Syntaxin 2	Protein Co	37	GC12M130	2.19
4929	ATL2	Atlastin G1	Protein Co	36	GC02M038	2.19
4930	NEXMIF	Neurite Ex	Protein Co	27	GC0XM074	2.19
4931	HLA-K	Major Hist	Pseudoger	12	GC06P047	2.19
4932	ATP6V1E1	ATPase H+	Protein Co	44	GC22M017	2.19
4933	MIOX	Myo-Inosit	Protein Co	39	GC22P050	2.19
4934	GSDMD	Gasdermin	Protein Co	39	GC08P143	2.19
4935	GRM7	Glutamate	Protein Co	44	GC03P006	2.19
4936	LOXL4	Lysyl Oxid	Protein Co	42	GC10M098	2.19
4937	CKAP5	Cytoskelet	Protein Co	40	GC11M061	2.19
4938	TP53RK	TP53 Regu	Protein Co	40	GC20M046	2.19
4939	PCMT1	Protein-L-I	Protein Co	40	GC06P149	2.19
4940	PRTFDC1	Phosphoril	Protein Co	33	GC10M024	2.19
4941	KIF2A	Kinesin Fa	Protein Co	42	GC05P062	2.19
4942	NPR1	Natriuretic	Protein Co	45	GC01P153	2.19
4943	SH3BP5	SH3 Doma	Protein Co	40	GC03M015	2.19

4944	NCL	Nucleolin	Protein Co	42	GC02M231	2.19
4945	CXorf56	Chromoso	Protein Co	35	GC0XM119	2.18
4946	AP3B1	Adaptor R	Protein Co	45	GC05M078	2.18
4947	THOC2	THO Comp	Protein Co	40	GC0XM123	2.18
4948	MICAL2	Microtubul	Protein Co	38	GC11P012	2.18
4949	NR2C2	Nuclear Re	Protein Co	43	GC03P014	2.18
4950	NANOG	Nanog Ho	Protein Co	39	GC12P007	2.18
4951	VPS35	VPS35 Ret	Protein Co	43	GC16M046	2.18
4952	ARG2	Arginase 2	Protein Co	45	GC14P067	2.18
4953	COL8A2	Collagen T	Protein Co	41	GC01M036	2.18
4954	MCOLN1	Mucolipin	Protein Co	44	GC19P007	2.18
4955	TIAL1	TIA1 Cytot	Protein Co	40	GC10M119	2.18
4956	SARS1	Seryl-TRNA	Protein Co	35	GC01P109	2.18
4957	OMG	Oligodend	Protein Co	41	GC17M031	2.18
4958	TPM1	Tropomyo	Protein Co	48	GC15P073	2.18
4959	TMEM106E	Transmem	Protein Co	38	GC07P012	2.18
4960	C1D	C1D Nucle	Protein Co	37	GC02M068	2.18
4961	CLIP1	CAP-Gly D	Protein Co	44	GC12M122	2.18
4962	TINAGL1	Tubulointe	Protein Co	39	GC01P031	2.18
4963	LOC11167	CFTR Prom	Biological	1	GC07P117	2.18
4964	KNL1	Kinetochor	Protein Co	33	GC15P040	2.18
4965	MYF5	Myogenic	Protein Co	40	GC12P080	2.18
4966	MS4A6A	Membrane	Protein Co	37	GC11M061	2.18
4967	IK	IK Cytokin	Protein Co	38	GC05P143	2.18
4968	CDK10	Cyclin Dep	Protein Co	44	GC16P089	2.18
4969	MIR103A2	MicroRNA	RNA Gene	18	GC20P003	2.18
4970	ATP5F1A	ATP Synth	Protein Co	36	GC18M046	2.17
4971	GSTM3	Glutathion	Protein Co	45	GC01M109	2.17
4972	CD1B	CD1b Mol	Protein Co	39	GC01M158	2.17
4973	ID4	Inhibitor C	Protein Co	39	GC06P019	2.17
4974	LINS1	Lines Hom	Protein Co	31	GC15M103	2.17
4975	ANK3	Ankyrin 3	Protein Co	44	GC10M060	2.17
4976	STEAP3	STEAP3 M	Protein Co	45	GC02P119	2.17
4977	AZIN2	Antizyme I	Protein Co	36	GC01P033	2.17
4978	UTP6	UTP6 Smal	Protein Co	37	GC17M031	2.17
4979	BACH1	BTB Doma	Protein Co	41	GC21P029	2.17
4980	NR5A1	Nuclear Re	Protein Co	50	GC09M124	2.17
4981	NDFIP1	Nedd4 Far	Protein Co	37	GC05P142	2.17
4982	SGF29	SAGA Com	Protein Co	30	GC16P028	2.17
4983	NUBPL	Nucleotide	Protein Co	39	GC14P031	2.17
4984	TMED10	Transmem	Protein Co	44	GC14M075	2.17
4985	GOLM1	Golgi Mem	Protein Co	39	GC09M086	2.17
4986	MEF2D	Myocyte E	Protein Co	43	GC01M156	2.16

4987	ADIPOR1	Adiponecti	Protein Co	44	GC01M202	2.16
4988	PRKD2	Protein Kir	Protein Co	45	GC19M046	2.16
4989	MLC1	Modulator	Protein Co	41	GC22M050	2.16
4990	DEGS2	Delta 4-De	Protein Co	38	GC14M100	2.16
4991	CAMK4	Calcium/C	Protein Co	45	GC05P111	2.16
4992	TRIB2	Tribbles Ps	Protein Co	37	GC02P012	2.16
4993	MORN4	MORN Rep	Protein Co	32	GC10M097	2.16
4994	CTSA	Cathepsin	Protein Co	45	GC20P045	2.16
4995	ALDH4A1	Aldehyde I	Protein Co	44	GC01M018	2.16
4996	RRAS2	RAS Relate	Protein Co	46	GC11M014	2.16
4997	PNKD	PNKD Met	Protein Co	42	GC02P218	2.16
4998	PODN	Podocan	Protein Co	36	GC01P053	2.16
4999	UBE2I	Ubiquitin C	Protein Co	48	GC16P001	2.16
5000	MNT	MAX Netw	Protein Co	36	GC17M002	2.15
5001	H3-3B	H3.3 Histo	Protein Co	33	GC17M075	2.15
5002	SPESP1	Sperm Equ	Protein Co	32	GC15P068	2.15
5003	JCHAIN	Joining Ch	Protein Co	35	GC04M070	2.15
5004	DAZAP2	DAZ Assoc	Protein Co	37	GC12P051	2.15
5005	COL11A1	Collagen T	Protein Co	43	GC01M102	2.15
5006	FAM20C	FAM20C G	Protein Co	41	GC07P000	2.15
5007	PRDX1	Peroxiredo	Protein Co	49	GC01M045	2.15
5008	SLC13A1	Solute Car	Protein Co	36	GC07M123	2.15
5009	ATP1B1	ATPase Na	Protein Co	47	GC01P169	2.15
5010	RFFL	Ring Finge	Protein Co	39	GC17M035	2.15
5011	SYNGR2	Synaptogy	Protein Co	37	GC17P078	2.15
5012	GPAA1	Glycosylph	Protein Co	42	GC08P144	2.15
5013	CSNK2A2	Casein Kin	Protein Co	47	GC16M058	2.15
5014	KMT5B	Lysine Met	Protein Co	32	GC11M068	2.15
5015	SLC44A1	Solute Car	Protein Co	43	GC09P105	2.15
5016	MYO15A	Myosin XV	Protein Co	36	GC17P018	2.15
5017	RPSA	Ribosomal	Protein Co	44	GC03P039	2.15
5018	TIMP4	TIMP Meta	Protein Co	40	GC03M012	2.15
5019	ACSS2	Acyl-CoA S	Protein Co	43	GC20P034	2.14
5020	TM6SF2	Transmem	Protein Co	29	GC19M019	2.14
5021	CALML4	Calmodulir	Protein Co	34	GC15M068	2.14
5022	CARD16	Caspase R	Protein Co	36	GC11M105	2.14
5023	ATG13	Autophagy	Protein Co	38	GC11P046	2.14
5024	ADM5	Adrenome	Protein Co	21	GC19P049	2.14
5025	MDS2	Myelodysp	RNA Gene	25	GC01P023	2.14
5026	SUCLA2	Succinate-(	Protein Co	46	GC13M047	2.14
5027	DFFA	DNA Fragr	Protein Co	45	GC01M010	2.14
5028	CACNB2	Calcium V	Protein Co	47	GC10P018	2.14
5029	H1-0	H1.0 Linke	Protein Co	31	GC22P037	2.14

5030	TMEM43	Transmem	Protein Co	40	GC03P014	2.14
5031	SEL1L	SEL1L Ada	Protein Co	41	GC14M081	2.13
5032	FRMD4A	FERM Dor	Protein Co	37	GC10M013	2.13
5033	MIR629	MicroRNA	RNA Gene	16	GC15M070	2.13
5034	GNA13	G Protein	Protein Co	44	GC17M065	2.13
5035	DSC3	Desmocoll	Protein Co	42	GC18M030	2.13
5036	FOSB	FosB Protc	Protein Co	41	GC19P045	2.13
5037	NUP214	Nucleopor	Protein Co	43	GC09P131	2.13
5038	PHRF1	PHD And I	Protein Co	35	GC11P000	2.13
5039	ADAMTS14	ADAM Me	Protein Co	37	GC10P070	2.13
5040	PIAS4	Protein Inh	Protein Co	43	GC19P004	2.13
5041	PRKACA	Protein Kir	Protein Co	51	GC19M014	2.12
5042	C1QBP	Compleme	Protein Co	45	GC17M005	2.12
5043	MIR128-1	MicroRNA	RNA Gene	19	GC02P135	2.12
5044	SOSTDC1	Sclerostin	Protein Co	40	GC07M016	2.12
5045	MUC7	Mucin 7, S	Protein Co	37	GC04P070	2.12
5046	ENSG00000237669		Pseudoger	4	GC06M030	2.12
5047	ATG4B	Autophagy	Protein Co	40	GC02P241	2.12
5048	PSAT1	Phosphose	Protein Co	48	GC09P078	2.12
5049	NOSTRIN	Nitric Oxid	Protein Co	37	GC02P168	2.11
5050	CHRNA2	Cholinergi	Protein Co	46	GC08M027	2.11
5051	NIPAL4	NIPA Like	Protein Co	39	GC05P157	2.11
5052	AMPD2	Adenosine	Protein Co	46	GC01P109	2.11
5053	EDC3	Enhancer (	Protein Co	41	GC15M074	2.11
5054	PTMS	Parathymo	Protein Co	37	GC12P006	2.11
5055	LAIR1	Leukocyte	Protein Co	41	GC19M054	2.11
5056	COQ7	Coenzyme	Protein Co	43	GC16P019	2.11
5057	XCL1	X-C Motif	Protein Co	37	GC01P168	2.11
5058	IFNLR1	Interferon	Protein Co	35	GC01M024	2.11
5059	CYP20A1	Cytochrom	Protein Co	36	GC02P203	2.11
5060	GYPC	Glycophori	Protein Co	43	GC02P126	2.11
5061	MLLT1	MLLT1 Sup	Protein Co	39	GC19M006	2.11
5062	WWP2	WW Doma	Protein Co	43	GC16P069	2.11
5063	PLCB2	Phospholip	Protein Co	46	GC15M040	2.11
5064	TGFBR3	Transformi	Protein Co	45	GC01M091	2.11
5065	ZNF318	Zinc Finge	Protein Co	35	GC06M043	2.11
5066	FMNL1	Formin Lik	Protein Co	37	GC17P045	2.11
5067	KANSL3	KAT8 Regu	Protein Co	34	GC02M096	2.11
5068	AMFR	Autocrine	Protein Co	43	GC16M056	2.11
5069	CD320	CD320 Mo	Protein Co	40	GC19M008	2.11
5070	TPM3	Tropomyo	Protein Co	47	GC01M154	2.11
5071	PTGDR	Prostaglan	Protein Co	47	GC14P052	2.11
5072	ARNT2	Aryl Hydro	Protein Co	43	GC15P080	2.11

5073	OGT	O-Linked N	Protein Co	44	GC0XP071	2.1
5074	GEMIN4	Gem Nucle	Protein Co	38	GC17M000	2.1
5075	NAV1	Neuron Na	Protein Co	37	GC01P201	2.1
5076	TRRAP	Transforma	Protein Co	45	GC07P098	2.1
5077	RUSF1	RUS Family	Protein Co	26	GC16M031	2.1
5078	KLF11	Kruppel Lil	Protein Co	39	GC02P010	2.1
5079	PLEKHG2	Pleckstrin I	Protein Co	38	GC19P039	2.1
5080	NBPF2P	NBPF Mem	Pseudoger	10	GC01M021	2.1
5081	CYP4F3	Cytochrom	Protein Co	42	GC19P015	2.1
5082	PICK1	Protein Int	Protein Co	41	GC22P038	2.1
5083	LTBP1	Latent Tra	Protein Co	43	GC02P032	2.1
5084	COL4A5	Collagen T	Protein Co	43	GC0XP108	2.1
5085	PPP2R5D	Protein Ph	Protein Co	44	GC06P047	2.1
5086	IDH3A	Isocitrate D	Protein Co	45	GC15P078	2.1
5087	HOXA9	Homeobox	Protein Co	39	GC07M027	2.1
5088	HEY2	Hes Relate	Protein Co	38	GC06P125	2.1
5089	SLC17A6	Solute Car	Protein Co	40	GC11P022	2.1
5090	ATP2C2	ATPase Se	Protein Co	40	GC16P084	2.1
5091	SMIM20	Small Integ	Protein Co	29	GC04P025	2.1
5092	GPR18	G Protein-(	Protein Co	39	GC13M099	2.1
5093	UBA7	Ubiquitin L	Protein Co	41	GC03M049	2.1
5094	ATP5PD	ATP Synth	Protein Co	31	GC17M075	2.1
5095	AGPAT1	1-Acylglyc	Protein Co	41	GC06M032	2.1
5096	ACSF3	Acyl-CoA S	Protein Co	43	GC16P089	2.1
5097	CHRDL1	Chordin Lil	Protein Co	40	GC0XM110	2.09
5098	CERS3	Ceramide S	Protein Co	39	GC15M103	2.09
5099	IP6K2	Inositol He	Protein Co	39	GC03M048	2.09
5100	MLXIP	MLX Intera	Protein Co	33	GC12P122	2.09
5101	KIF21A	Kinesin Fa	Protein Co	39	GC12M039	2.09
5102	KDM5B	Lysine Den	Protein Co	43	GC01M202	2.09
5103	FXYD1	FXYD Dom	Protein Co	37	GC19P038	2.09
5104	APLP2	Amyloid Be	Protein Co	41	GC11P130	2.09
5105	EEF1A1	Eukaryotic	Protein Co	43	GC06M073	2.09
5106	PPP3CB	Protein Ph	Protein Co	44	GC10M073	2.09
5107	PATZ1	POZ/BTB A	Protein Co	38	GC22M031	2.09
5108	S100PBP	S100P Bind	Protein Co	34	GC01P032	2.09
5109	BCAP31	B Cell Rece	Protein Co	41	GC0XM153	2.09
5110	OTUD6B	OTU Deub	Protein Co	40	GC08P091	2.09
5111	ITPR2	Inositol 1,4	Protein Co	45	GC12M026	2.09
5112	C1QTNF3	C1q And T	Protein Co	38	GC05M034	2.08
5113	NCOA6	Nuclear Re	Protein Co	39	GC20M034	2.08
5114	TEAD1	TEA Doma	Protein Co	47	GC11P012	2.08
5115	INTS11	Integrator	Protein Co	28	GC01M001	2.08

5116	HOXB2	Homeobo>	Protein Co	40	GC17M048	2.08
5117	M6PR	Mannose-(	Protein Co	43	GC12M008	2.08
5118	ADCY6	Adenylate	Protein Co	47	GC12M048	2.08
5119	ME3	Malic Enzy	Protein Co	39	GC11M086	2.08
5120	EIF3G	Eukaryotic	Protein Co	38	GC19M010	2.08
5121	DIRAS3	DIRAS Fan	Protein Co	32	GC01M068	2.08
5122	CARD14	Caspase R(	Protein Co	43	GC17P080	2.08
5123	WNT9B	Wnt Family	Protein Co	41	GC17P046	2.08
5124	PCSK1N	Proprotein	Protein Co	33	GC0XM048	2.08
5125	TLE3	TLE Family	Protein Co	43	GC15M070	2.08
5126	COX8A	Cytochrom	Protein Co	41	GC11P063	2.07
5127	RBM5	RNA Bindi	Protein Co	39	GC03P050	2.07
5128	NEO1	Neogenin	Protein Co	40	GC15P073	2.07
5129	PNPLA6	Patatin Lik	Protein Co	44	GC19P007	2.07
5130	GIGYF2	GRB10 Int	Protein Co	39	GC02P232	2.07
5131	LGALS7B	Galectin 7I	Protein Co	32	GC19P038	2.07
5132	CLDN6	Claudin 6	Protein Co	40	GC16M003	2.07
5133	MAP3K2	Mitogen-A	Protein Co	44	GC02M127	2.07
5134	LILRB2	Leukocyte	Protein Co	40	GC19M054	2.07
5135	PRRX1	Paired Rel	Protein Co	42	GC01P170	2.07
5136	CPT1C	Carnitine F	Protein Co	42	GC19P049	2.07
5137	ZNF175	Zinc Finge	Protein Co	38	GC19P051	2.07
5138	HSPB3	Heat Shoc	Protein Co	39	GC05P054	2.07
5139	NEXN	Nexilin F-A	Protein Co	39	GC01P077	2.07
5140	SLC2A3	Solute Car	Protein Co	48	GC12M007	2.07
5141	FTO	FTO Alpha	Protein Co	44	GC16P053	2.07
5142	RPE65	Retinoid Is	Protein Co	44	GC01M068	2.07
5143	MBD2	Methyl-Cp	Protein Co	40	GC18M054	2.07
5144	S1PR2	Sphingosir	Protein Co	45	GC19M010	2.07
5145	NLRP2	NLR Family	Protein Co	41	GC19P054	2.07
5146	SSBP3	Single Stra	Protein Co	38	GC01M054	2.06
5147	KMO	Kynurenine	Protein Co	44	GC01P241	2.06
5148	TRIM37	Tripartite M	Protein Co	42	GC17M058	2.06
5149	ERC1	ELKS/RAB6	Protein Co	41	GC12P000	2.06
5150	MIRLET7A3	MicroRNA	RNA Gene	19	GC22P046	2.06
5151	DLST	Dihydrolip	Protein Co	45	GC14P074	2.06
5152	RNLS	Renalase, I	Protein Co	38	GC10M088	2.06
5153	TMX1	Thioredoxi	Protein Co	38	GC14P051	2.06
5154	BCL2A1	BCL2 Relat	Protein Co	41	GC15M079	2.06
5155	GSTA4	Glutathion	Protein Co	43	GC06M052	2.06
5156	ID2-AS1	ID2 Antise	RNA Gene	12	GC02M008	2.06
5157	HLA-H	Major Hist	Pseudoger	25	GC06P047	2.06
5158	MIEF1	Mitochond	Protein Co	32	GC22P039	2.06

5159	CPSF6	Cleavage /	Protein Co	39	GC12P069	2.05
5160	AMPH	Amphiphy:	Protein Co	44	GC07M038	2.05
5161	HDGF	Heparin Bi	Protein Co	40	GC01M156	2.05
5162	RASGRP2	RAS Guany	Protein Co	45	GC11M064	2.05
5163	ME2	Malic Enzy	Protein Co	44	GC18P050	2.05
5164	LIG1	DNA Ligas	Protein Co	46	GC19M048	2.05
5165	STX1B	Syntaxin 1	Protein Co	40	GC16M030	2.05
5166	SEC61B	SEC61 Trar	Protein Co	37	GC09P099	2.05
5167	MAP4K2	Mitogen-A	Protein Co	46	GC11M064	2.05
5168	TBL1X	Transducin	Protein Co	36	GC0XP009	2.05
5169	IGF2BP3	Insulin Like	Protein Co	41	GC07M023	2.05
5170	UCN2	Urocortin 2	Protein Co	33	GC03M048	2.05
5171	NID2	Nidogen 2	Protein Co	37	GC14M052	2.05
5172	WWTR1	WW Doma	Protein Co	41	GC03M149	2.05
5173	SACM1L	SAC1 Like	Protein Co	39	GC03P045	2.05
5174	WDR26	WD Repea	Protein Co	40	GC01M224	2.05
5175	GIT2	GIT ArfGAI	Protein Co	41	GC12M109	2.05
5176	SLC38A5	Solute Car	Protein Co	38	GC0XM048	2.05
5177	SRSF3	Serine Anc	Protein Co	40	GC06P047	2.05
5178	GMPS	Guanine M	Protein Co	44	GC03P155	2.05
5179	KDSR	3-Ketodihy	Protein Co	42	GC18M063	2.05
5180	GADD45GI	GADD45G	Protein Co	35	GC19M012	2.05
5181	TNFRSF19	TNF Recep	Protein Co	39	GC13P023	2.04
5182	PLAAT3	Phospholip	Protein Co	33	GC11M063	2.04
5183	IL31	Interleukin	Protein Co	35	GC12M122	2.04
5184	PRDX3	Peroxiredo	Protein Co	43	GC10M119	2.04
5185	NDRG2	NDRG Fam	Protein Co	38	GC14M021	2.04
5186	ATP5F1C	ATP Synth	Protein Co	32	GC10P007	2.04
5187	GLDC	Glycine De	Protein Co	47	GC09M006	2.04
5188	GATAD2B	GATA Zinc	Protein Co	42	GC01M153	2.04
5189	WDR82	WD Repea	Protein Co	36	GC03M052	2.04
5190	DGKZ	Diacylglyce	Protein Co	44	GC11P046	2.04
5191	PKP3	Plakophilir	Protein Co	37	GC11P000	2.04
5192	HLA-W	Major Hist	Pseudoger	9	GC06P047	2.04
5193	MC4R	Melanocor	Protein Co	45	GC18M060	2.04
5194	DDAH2	Dimethylar	Protein Co	42	GC06M031	2.04
5195	IL22RA2	Interleukin	Protein Co	39	GC06M137	2.03
5196	EPC1	Enhancer (	Protein Co	39	GC10M032	2.03
5197	PTGIS	Prostaglan	Protein Co	47	GC20M049	2.03
5198	BCAM	Basal Cell /	Protein Co	40	GC19P044	2.03
5199	RPS6KC1	Ribosomal	Protein Co	39	GC01P213	2.03
5200	KARS1	Lysyl-TRNA	Protein Co	38	GC16M075	2.03
5201	GZMA	Granzyme	Protein Co	42	GC05P055	2.03

5202	CDH23	Cadherin F	Protein Co	41	GC10P071	2.03
5203	NCOA1	Nuclear Re	Protein Co	44	GC02P024	2.03
5204	MRTFA	Myocardin	Protein Co	34	GC22M045	2.03
5205	SLC38A2	Solute Car	Protein Co	41	GC12M046	2.03
5206	HMMR	Hyaluronar	Protein Co	42	GC05P163	2.03
5207	PTN	Pleiotroph	Protein Co	43	GC07M137	2.03
5208	IKZF3	IKAROS Fa	Protein Co	41	GC17M039	2.03
5209	BUD23	BUD23 RR	Protein Co	30	GC07P073	2.03
5210	AREL1	Apoptosis	Protein Co	33	GC14M074	2.03
5211	THUMPD2	THUMP Dc	Protein Co	32	GC02M039	2.03
5212	CDO1	Cysteine D	Protein Co	42	GC05M115	2.03
5213	APOM	Apolipopro	Protein Co	40	GC06P047	2.03
5214	SLC20A1	Solute Car	Protein Co	44	GC02P115	2.03
5215	CCDC89	Coiled-Coi	Protein Co	30	GC11M085	2.03
5216	GTF2E2	General Tr	Protein Co	43	GC08M030	2.02
5217	ZNF408	Zinc Finge	Protein Co	37	GC11P046	2.02
5218	SLC12A3	Solute Car	Protein Co	47	GC16P056	2.02
5219	VAMP7	Vesicle Ass	Protein Co	42	GC0XP155	2.02
5220	LEPQTL1	Leptin, Ser	Genetic Lo	3	GC02U903	2.02
5221	CNTN2	Contactin 2	Protein Co	45	GC01P205	2.02
5222	CHTF8	Chromoso	Protein Co	33	GC16M069	2.02
5223	BNIP3L	BCL2 Inter	Protein Co	42	GC08P026	2.02
5224	KCNA5	Potassium	Protein Co	45	GC12P005	2.02
5225	MIR30B	MicroRNA	RNA Gene	20	GC08M134	2.02
5226	ACAA2	Acetyl-CoA	Protein Co	43	GC18M049	2.02
5227	RILP	Rab Intera	Protein Co	36	GC17M001	2.02
5228	PI4KB	Phosphatic	Protein Co	45	GC01M151	2.02
5229	RGS10	Regulator	Protein Co	43	GC10M119	2.02
5230	HNRNPD	Heterogen	Protein Co	42	GC04M082	2.02
5231	WDFY3	WD Repea	Protein Co	40	GC04M084	2.02
5232	SLC25A24	Solute Car	Protein Co	43	GC01M108	2.02
5233	CBX5	Chromobo	Protein Co	44	GC12M054	2.02
5234	SLC4A3	Solute Car	Protein Co	40	GC02P219	2.02
5235	TAGLN2	Transgelin	Protein Co	40	GC01M159	2.02
5236	PPP3CC	Protein Ph	Protein Co	44	GC08P022	2.01
5237	CLDN11	Claudin 11	Protein Co	40	GC03P170	2.01
5238	RPL9	Ribosomal	Protein Co	41	GC04M039	2.01
5239	SYTL2	Synaptota	Protein Co	39	GC11M085	2.01
5240	CCDC88C	Coiled-Coi	Protein Co	40	GC14M091	2.01
5241	CACNG3	Calcium V	Protein Co	41	GC16P024	2.01
5242	SMURF2	SMAD Spe	Protein Co	43	GC17M064	2.01
5243	EPSTI1	Epithelial S	Protein Co	35	GC13M042	2.01
5244	LDHC	Lactate De	Protein Co	42	GC11P018	2.01

5245	BCL2L10	BCL2 Like	Protein Co	39	GC15M060	2.01
5246	LIPF	Lipase F, G	Protein Co	41	GC10P088	2.01
5247	MIR129-1	MicroRNA	RNA Gene	17	GC07P128	2.01
5248	MT1A	Metallothi	Protein Co	40	GC16P056	2.01
5249	ARL15	ADP Ribos	Protein Co	36	GC05M053	2.01
5250	NPEPPS	Aminopep	Protein Co	41	GC17P047	2.01
5251	CDK7	Cyclin Dep	Protein Co	46	GC05P069	2.01
5252	AP4S1	Adaptor R	Protein Co	38	GC14P031	2.01
5253	TMTC1	Transmem	Protein Co	36	GC12M029	2.01
5254	BANF2	BANF Fam	Protein Co	32	GC20P017	2.01
5255	FGFBP1	Fibroblast	Protein Co	40	GC04M015	2.01
5256	P4HA3	Prolyl 4-Hy	Protein Co	39	GC11M074	2
5257	CRABP1	Cellular Re	Protein Co	41	GC15P078	2
5258	CDK13	Cyclin Dep	Protein Co	41	GC07P040	2
5259	SENP1	SUMO Spe	Protein Co	42	GC12M048	2
5260	CRADD	CASP2 And	Protein Co	44	GC12P093	2
5261	MICD	MHC Class	Pseudoger	10	GC06M030	2
5262	CCDC80	Coiled-Coi	Protein Co	36	GC03M112	2
5263	PXDN	Peroxidasi	Protein Co	41	GC02M001	2
5264	HHIP	Hedgehog	Protein Co	41	GC04P144	2
5265	RNGTT	RNA Guan	Protein Co	43	GC06M088	2
5266	CSAD	Cysteine S	Protein Co	40	GC12M053	2
5267	TLN1	Talin 1	Protein Co	41	GC09M035	2
5268	CYTH2	Cytohesin	Protein Co	41	GC19P048	2
5269	UACA	Uveal Autc	Protein Co	36	GC15M070	2
5270	SENP3	SUMO Spe	Protein Co	40	GC17P008	2
5271	GSE1	Gse1 Coile	Protein Co	34	GC16P085	2
5272	COX6A1	Cytochrom	Protein Co	44	GC12P120	2
5273	NUB1	Negative R	Protein Co	37	GC07P151	1.99
5274	ADAM9	ADAM Me	Protein Co	47	GC08P038	1.99
5275	NAE1	NEDD8 Ac	Protein Co	41	GC16M066	1.99
5276	RASSF5	Ras Associ	Protein Co	40	GC01P206	1.99
5277	INTS8	Integrator	Protein Co	36	GC08P094	1.99
5278	SUMO1	Small Ubic	Protein Co	46	GC02M202	1.99
5279	SH3PXD2A	SH3 And F	Protein Co	37	GC10M103	1.99
5280	GOT1	Glutamic-C	Protein Co	47	GC10M099	1.99
5281	FBXO32	F-Box Prot	Protein Co	40	GC08M123	1.99
5282	BIN1	Bridging Ir	Protein Co	46	GC02M127	1.99
5283	SLC6A5	Solute Car	Protein Co	46	GC11P020	1.99
5284	EHHADH	Enoyl-CoA	Protein Co	44	GC03M185	1.99
5285	DPT	Dermatopo	Protein Co	37	GC01M168	1.99
5286	ATP5PF	ATP Synth	Protein Co	31	GC21M025	1.99
5287	P2RX5	Purinergic	Protein Co	39	GC17M003	1.99

5288	GABRA5	Gamma-A	Protein Co	46	GC15P026	1.99
5289	GDF6	Growth Di	Protein Co	43	GC08M096	1.99
5290	MIR422A	MicroRNA	RNA Gene	16	GC15M063	1.98
5291	NPR2	Natriuretic	Protein Co	47	GC09P035	1.98
5292	CBX3	Chromobo	Protein Co	43	GC07P026	1.98
5293	GORAB	Golgin, RA	Protein Co	37	GC01P170	1.98
5294	TRIM2	Tripartite M	Protein Co	40	GC04P153	1.98
5295	FCSK	Fucose Kin	Protein Co	31	GC16P070	1.98
5296	DUSP6	Dual Speci	Protein Co	48	GC12M089	1.98
5297	PRR16	Proline Ric	Protein Co	33	GC05P120	1.98
5298	ALOXE3	Arachidon	Protein Co	41	GC17M008	1.98
5299	ANXA3	Annexin A	Protein Co	42	GC04P078	1.98
5300	MIR497	MicroRNA	RNA Gene	17	GC17M007	1.98
5301	ITPK1	Inositol-Te	Protein Co	41	GC14M092	1.98
5302	CCL16	C-C Motif	Protein Co	38	GC17M035	1.98
5303	MPHOSPH	M-Phase P	Protein Co	37	GC13P019	1.98
5304	MUC15	Mucin 15,	Protein Co	35	GC11M026	1.98
5305	SVBP	Small Vasc	Protein Co	27	GC01M042	1.98
5306	TENT4A	Terminal N	Protein Co	30	GC05P006	1.98
5307	CLSTN1	Calsyntenii	Protein Co	38	GC01M009	1.98
5308	MRPL58	Mitochond	Protein Co	31	GC17P075	1.98
5309	TRAPPC6B	Trafficking	Protein Co	39	GC14M039	1.97
5310	FOXQ1	Forkhead I	Protein Co	33	GC06P001	1.97
5311	TNFSF9	TNF Super	Protein Co	37	GC19P006	1.97
5312	PDHX	Pyruvate D	Protein Co	47	GC11P034	1.97
5313	RNF146	Ring Finge	Protein Co	37	GC06P127	1.97
5314	SNX3	Sorting Ne	Protein Co	43	GC06M108	1.97
5315	NDUFC2	NADH:Ubi	Protein Co	39	GC11M078	1.97
5316	ENPP6	Ectonuclec	Protein Co	38	GC04M184	1.97
5317	NEUROG1	Neurogeni	Protein Co	40	GC05M135	1.97
5318	NPHS2	NPHS2 Stc	Protein Co	41	GC01M179	1.97
5319	RTN3	Reticulon 3	Protein Co	41	GC11P063	1.97
5320	SLC17A7	Solute Car	Protein Co	41	GC19M049	1.97
5321	YWHAE	Tyrosine 3	Protein Co	50	GC17M001	1.97
5322	LIMK2	LIM Doma	Protein Co	47	GC22P031	1.97
5323	HTR3B	5-Hydroxy	Protein Co	44	GC11P113	1.97
5324	SOS2	SOS Ras/R	Protein Co	45	GC14M050	1.97
5325	ENSG00000276418		Protein Co	8	GC08M079	1.97
5326	VARS1	Valyl-TRNA	Protein Co	35	GC06M032	1.97
5327	BSX	Brain Spec	Protein Co	32	GC11M122	1.97
5328	PTPRZ1	Protein Ty	Protein Co	43	GC07P121	1.97
5329	CEP170	Centrosom	Protein Co	39	GC01M243	1.97
5330	LMCD1	LIM And C	Protein Co	39	GC03P008	1.97

5331	AFDN	Afadin, Ad	Protein Co	34	GC06P167	1.97
5332	SLC6A17	Solute Car	Protein Co	41	GC01P110	1.97
5333	RPL12	Ribosomal	Protein Co	41	GC09M127	1.96
5334	SFRP5	Secreted F	Protein Co	39	GC10M097	1.96
5335	CDAN1	Codanin 1	Protein Co	39	GC15M042	1.96
5336	LSG1	Large 60S	Protein Co	34	GC03M194	1.96
5337	METTL23	Methyltran	Protein Co	35	GC17P076	1.96
5338	ATAT1	Alpha Tub	Protein Co	34	GC06P030	1.96
5339	AMH	Anti-Mulle	Protein Co	43	GC19P002	1.96
5340	ISLR	Immunogl	Protein Co	36	GC15P074	1.96
5341	SPTSSA	Serine Palr	Protein Co	34	GC14M034	1.96
5342	DGKA	Diacylglyce	Protein Co	44	GC12P055	1.96
5343	MSTO1	Misato Mit	Protein Co	39	GC01P155	1.96
5344	ARHGDIB	Rho GDP [	Protein Co	42	GC12M014	1.96
5345	EXOC5	Exocyst Co	Protein Co	41	GC14M057	1.96
5346	CTNNA2	Catenin Al	Protein Co	43	GC02P079	1.96
5347	ITFG1	Integrin Al	Protein Co	36	GC16M047	1.96
5348	RAB10	RAB10, Me	Protein Co	43	GC02P026	1.96
5349	CHPT1	Choline Ph	Protein Co	39	GC12P101	1.96
5350	SLC4A8	Solute Car	Protein Co	39	GC12P051	1.96
5351	AZGP1	Alpha-2-G	Protein Co	43	GC07M099	1.96
5352	TRAPPC3	Trafficking	Protein Co	39	GC01M036	1.96
5353	TOR2A	Torsin Fam	Protein Co	36	GC09M127	1.96
5354	TFCP2	Transcripti	Protein Co	40	GC12M051	1.96
5355	MIR612	MicroRNA	RNA Gene	17	GC11P065	1.96
5356	TSC22D3	TSC22 Dor	Protein Co	40	GC0XM107	1.96
5357	GLG1	Golgi Glyc	Protein Co	37	GC16M074	1.96
5358	IVL	Involucrin	Protein Co	37	GC01P152	1.96
5359	MIRLET7F1	MicroRNA	RNA Gene	18	GC09P094	1.96
5360	RNASE2	Ribonuclea	Protein Co	37	GC14P021	1.96
5361	SPARCL1	SPARC Lik	Protein Co	36	GC04M087	1.95
5362	MEOX1	Mesenchyr	Protein Co	42	GC17M043	1.95
5363	DCAF17	DDB1 And	Protein Co	36	GC02P171	1.95
5364	ERLIN1	ER Lipid Ra	Protein Co	41	GC10M100	1.95
5365	CXXC1	CXXC Fing	Protein Co	40	GC18M050	1.95
5366	MAEA	Macropha	Protein Co	38	GC04P001	1.95
5367	DENND6A	DENN Dor	Protein Co	33	GC03M057	1.95
5368	INPP5D	Inositol Po	Protein Co	45	GC02P233	1.95
5369	INPP5F	Inositol Po	Protein Co	37	GC10P119	1.95
5370	MPRIP	Myosin Ph	Protein Co	37	GC17P017	1.95
5371	MAP4	Microtubul	Protein Co	41	GC03M047	1.95
5372	MARCHF6	Membrane	Protein Co	29	GC05P010	1.95
5373	UNC5B	Unc-5 Net	Protein Co	37	GC10P071	1.95

5374	MCF2	MCF.2 Cell	Protein Co	40	GC0XM13	1.95
5375	GMNN	Geminin D	Protein Co	44	GC06P024	1.95
5376	PLAA	Phospholip	Protein Co	43	GC09M026	1.95
5377	HNRNPDL	Heterogen	Protein Co	39	GC04M082	1.95
5378	RHOG	Ras Homo	Protein Co	41	GC11M003	1.95
5379	HYAL1	Hyaluronic	Protein Co	45	GC03M050	1.95
5380	RRN3	RRN3 Hom	Protein Co	39	GC16M015	1.95
5381	ATPAF2	ATP Synth	Protein Co	37	GC17M017	1.95
5382	WDR4	WD Repea	Protein Co	38	GC21M042	1.94
5383	SEMA4D	Semaphori	Protein Co	44	GC09M089	1.94
5384	LTB4R2	Leukotrien	Protein Co	41	GC14P025	1.94
5385	MAP4K4	Mitogen-A	Protein Co	47	GC02P101	1.94
5386	CHP2	Calcineurir	Protein Co	33	GC16P023	1.94
5387	MYDGF	Myeloid D	Protein Co	34	GC19M004	1.94
5388	LIN28B	Lin-28 Hor	Protein Co	41	GC06P104	1.94
5389	ZSCAN9	Zinc Finge	Protein Co	31	GC06P028	1.94
5390	VAMP3	Vesicle Ass	Protein Co	41	GC01P007	1.94
5391	MIR874	MicroRNA	RNA Gene	18	GC05M137	1.94
5392	FABP7	Fatty Acid	Protein Co	43	GC06P122	1.94
5393	OSGEP	O-Sialogly	Protein Co	41	GC14M020	1.94
5394	OTULINL	OTU Deub	Protein Co	27	GC05P014	1.94
5395	TBR1	T-Box Brai	Protein Co	41	GC02P161	1.94
5396	MAP4K1	Mitogen-A	Protein Co	43	GC19M038	1.94
5397	COPS3	COP9 Sign	Protein Co	39	GC17M017	1.94
5398	EOMES	Eomesode	Protein Co	43	GC03M027	1.94
5399	KERA	Keratocan	Protein Co	41	GC12M091	1.94
5400	ZNF202	Zinc Finge	Protein Co	39	GC11M123	1.93
5401	MYPN	Myopallad	Protein Co	41	GC10P068	1.93
5402	APBA3	Amyloid B	Protein Co	38	GC19M003	1.93
5403	MAP1LC3A	Microtubul	Protein Co	43	GC20P034	1.93
5404	TAC3	Tachykinin	Protein Co	45	GC12M057	1.93
5405	SH3PXD2B	SH3 And F	Protein Co	38	GC05M172	1.93
5406	CKMT1B	Creatine K	Protein Co	39	GC15P043	1.93
5407	USP14	Ubiquitin S	Protein Co	45	GC18P000	1.93
5408	CHCHD2	Coiled-Coi	Protein Co	39	GC07M056	1.93
5409	RBFOX2	RNA Bindi	Protein Co	38	GC22M035	1.93
5410	POLR2A	RNA Polyn	Protein Co	44	GC17P008	1.93
5411	MAPK12	Mitogen-A	Protein Co	48	GC22M050	1.93
5412	FES	FES Proto-	Protein Co	48	GC15P090	1.93
5413	ATP5IF1	ATP Synth	Protein Co	30	GC01P028	1.93
5414	PRAC1	PRAC1 Sm	Protein Co	25	GC17M048	1.93
5415	SMIM14	Small Inte	Protein Co	30	GC04M039	1.93
5416	REV1	REV1 DNA	Protein Co	41	GC02M099	1.93

5417	TRAF5	TNF Recep	Protein Co	42	GC01P211	1.92
5418	SRP14	Signal Rec	Protein Co	38	GC15M040	1.92
5419	ARGLU1	Arginine A	Protein Co	36	GC13M106	1.92
5420	CRELD1	Cysteine R	Protein Co	39	GC03P009	1.92
5421	SPAG5	Sperm Ass	Protein Co	37	GC17M028	1.92
5422	HDAC5	Histone De	Protein Co	47	GC17M044	1.92
5423	PER3	Period Circ	Protein Co	43	GC01P007	1.92
5424	TP53BP1	Tumor Pro	Protein Co	44	GC15M043	1.92
5425	PRR5	Proline Ric	Protein Co	39	GC22P044	1.92
5426	GALR2	Galanin Re	Protein Co	41	GC17P076	1.92
5427	LTK	Leukocyte	Protein Co	41	GC15M041	1.92
5428	GTF2H5	General Tr	Protein Co	40	GC06P158	1.92
5429	ATP6V0C	ATPase H+	Protein Co	41	GC16P002	1.92
5430	H3C14	H3 Cluster	Protein Co	29	GC01M149	1.92
5431	STIM2	Stromal In	Protein Co	39	GC04P026	1.92
5432	KCNJ6	Potassium	Protein Co	46	GC21M037	1.91
5433	MTF1	Metal Regu	Protein Co	41	GC01M037	1.91
5434	PA2G4	Proliferatic	Protein Co	40	GC12P056	1.91
5435	SH2D3C	SH2 Doma	Protein Co	39	GC09M127	1.91
5436	PSMD12	Proteasom	Protein Co	41	GC17M067	1.91
5437	RGS19	Regulator	Protein Co	40	GC20M064	1.91
5438	GTPBP2	GTP Bindir	Protein Co	38	GC06M043	1.91
5439	CLCN7	Chloride V	Protein Co	45	GC16M001	1.91
5440	HAGLROS	HAGLR Op	RNA Gene	14	GC02P176	1.91
5441	MATN2	Matrilin 2	Protein Co	39	GC08P097	1.91
5442	AGBL1	ATP/GTP B	Protein Co	36	GC15P086	1.91
5443	TMC6	Transmem	Protein Co	42	GC17M078	1.91
5444	SCP2	Sterol Carr	Protein Co	47	GC01P052	1.91
5445	TPH2	Tryptophar	Protein Co	48	GC12P071	1.91
5446	HEPACAM	HEPACAM	Protein Co	32	GC07M093	1.91
5447	PPP1R26	Protein Ph	Protein Co	32	GC09P135	1.91
5448	MCF2L	MCF.2 Cell	Protein Co	42	GC13P112	1.91
5449	PPP1R35	Protein Ph	Protein Co	30	GC07M100	1.91
5450	SORBS3	Sorbin And	Protein Co	40	GC08P022	1.91
5451	NFKBIB	NFKB Inhib	Protein Co	41	GC19P038	1.9
5452	PAX7	Paired Box	Protein Co	44	GC01P018	1.9
5453	GSTM4	Glutathion	Protein Co	41	GC01P109	1.9
5454	PRCP	Prolylcarbo	Protein Co	45	GC11M082	1.9
5455	RF00017-5273		RNA Gene	4	GC06M011	1.9
5456	ANXA7	Annexin A	Protein Co	42	GC10M073	1.9
5457	FBP2	Fructose-B	Protein Co	43	GC09M094	1.9
5458	STC2	Stanniocal	Protein Co	40	GC05M173	1.9
5459	MARK2	Microtubul	Protein Co	44	GC11P063	1.9

5460	NDRG4	NDRG Fam	Protein Co	39	GC16P058	1.9
5461	LTBP2	Latent Tra	Protein Co	43	GC14M074	1.9
5462	VARS2	Valyl-TRNA	Protein Co	43	GC06P047	1.9
5463	NT5C3A	5'-Nucleot	Protein Co	40	GC07M033	1.89
5464	CDA	Cytidine D	Protein Co	43	GC01P020	1.89
5465	ARHGAP1	Rho GTPas	Protein Co	43	GC11M061	1.89
5466	SEC63	SEC63 Hor	Protein Co	43	GC06M107	1.89
5467	HDLBP	High Dens	Protein Co	40	GC02M241	1.89
5468	TMEM230	Transmem	Protein Co	36	GC20M005	1.89
5469	DDX1	DEAD-Box	Protein Co	43	GC02P015	1.89
5470	MIR211	MicroRNA	RNA Gene	18	GC15M031	1.89
5471	SUZ12	SUZ12 Pol	Protein Co	41	GC17P031	1.89
5472	ATP5PO	ATP Synth	Protein Co	33	GC21M033	1.89
5473	TMSB15A	Thymosin	Protein Co	29	GC0XM102	1.89
5474	STK24	Serine/Thr	Protein Co	44	GC13M098	1.89
5475	TRIM63	Tripartite M	Protein Co	40	GC01M026	1.89
5476	SHANK1	SH3 And M	Protein Co	37	GC19M050	1.89
5477	RPL39L	Ribosomal	Protein Co	31	GC03M187	1.89
5478	HCG4B	HLA Comp	RNA Gene	14	GC06M030	1.89
5479	AP4B1	Adaptor R	Protein Co	41	GC01M113	1.89
5480	DTNA	Dystrobrev	Protein Co	42	GC18P034	1.89
5481	FAP	Fibroblast	Protein Co	42	GC02M162	1.89
5482	ZYX	Zyxin	Protein Co	45	GC07P143	1.89
5483	RPL22	Ribosomal	Protein Co	43	GC01M006	1.89
5484	CBX1	Chromobo	Protein Co	40	GC17M048	1.89
5485	CD177	CD177 Mo	Protein Co	39	GC19P043	1.89
5486	KRBOX4	KRAB Box	Protein Co	30	GC0XP046	1.89
5487	ELMOD2	ELMO Dom	Protein Co	36	GC04P140	1.89
5488	SLC6A11	Solute Car	Protein Co	41	GC03P010	1.89
5489	FYB1	FYN Bindir	Protein Co	35	GC05M039	1.88
5490	ACVR2B	Activin A F	Protein Co	50	GC03P038	1.88
5491	HSPA6	Heat Shoc	Protein Co	44	GC01P161	1.88
5492	PIK3R4	Phosphoin	Protein Co	47	GC03M130	1.88
5493	SFN	Stratifin	Protein Co	46	GC01P026	1.88
5494	ARHGAP9	Rho GTPas	Protein Co	41	GC12M057	1.88
5495	PLXNA3	Plexin A3	Protein Co	38	GC0XP154	1.88
5496	CAP1	Cyclase As	Protein Co	41	GC01P040	1.88
5497	CCL1	C-C Motif	Protein Co	38	GC17M034	1.88
5498	FTX	FTX Transc	RNA Gene	18	GC0XM073	1.88
5499	ZNF335	Zinc Finge	Protein Co	37	GC20M045	1.88
5500	APBA2	Amyloid B	Protein Co	41	GC15P028	1.88
5501	AKTIP	AKT Intera	Protein Co	39	GC16M053	1.88
5502	TNRC6A	Trinucleoti	Protein Co	39	GC16P024	1.88

5503	HOXB7	Homeobo>	Protein Co	40	GC17M048	1.88
5504	H1-5	H1.5 Linke	Protein Co	32	GC06M028	1.88
5505	PIDD1	P53-Induc	Protein Co	35	GC11M000	1.88
5506	FOXRED1	FAD Depe	Protein Co	40	GC11P126	1.88
5507	ANKLE2	Ankyrin Re	Protein Co	39	GC12M132	1.88
5508	CLN6	CLN6 Tran	Protein Co	37	GC15M068	1.88
5509	PLEKHA7	Pleckstrin	Protein Co	38	GC11M016	1.88
5510	POC5	POC5 Cen	Protein Co	33	GC05M075	1.88
5511	CXXC5	CXXC Fing	Protein Co	39	GC05P139	1.87
5512	SDCCAG8	SHH Signa	Protein Co	41	GC01P243	1.87
5513	CRNN	Cornulin	Protein Co	37	GC01M152	1.87
5514	MAP2K6	Mitogen-A	Protein Co	47	GC17P069	1.87
5515	PDCD5	Programm	Protein Co	39	GC19P032	1.87
5516	UBE2A	Ubiquitin (	Protein Co	45	GC0XP119	1.87
5517	KANK1	KN Motif /	Protein Co	41	GC09P000	1.87
5518	SAMD12	Sterile Alp	Protein Co	37	GC08M118	1.87
5519	AGGF1	Angiogeni	Protein Co	41	GC05P077	1.87
5520	QDPR	Quinoid D	Protein Co	48	GC04M017	1.87
5521	ANAPC5	Anaphase	Protein Co	38	GC12M121	1.87
5522	IL34	Interleukin	Protein Co	39	GC16P070	1.87
5523	RHOH	Ras Homo	Protein Co	43	GC04P040	1.87
5524	SLC17A1	Solute Car	Protein Co	39	GC06M025	1.87
5525	HNRNPUL	Heterogen	Protein Co	37	GC19P041	1.87
5526	PCGF2	Polycomb	Protein Co	39	GC17M038	1.87
5527	WIPI1	WD Repea	Protein Co	39	GC17M068	1.87
5528	RANGAP1	Ran GTPas	Protein Co	41	GC22M041	1.86
5529	NFIC	Nuclear Fa	Protein Co	40	GC19P003	1.86
5530	ZWINT	ZW10 Inte	Protein Co	37	GC10M056	1.86
5531	LSR	Lipolysis S	Protein Co	40	GC19P038	1.86
5532	ENGASE	Endo-Beta	Protein Co	35	GC17P079	1.86
5533	AVIL	Advillin	Protein Co	36	GC12M057	1.86
5534	TXLNA	Taxilin Alp	Protein Co	36	GC01P032	1.86
5535	HPX	Hemopexi	Protein Co	39	GC11M006	1.86
5536	MYL1	Myosin Lig	Protein Co	41	GC02M210	1.86
5537	CST6	Cystatin E/	Protein Co	40	GC11P066	1.86
5538	WIPI2	WD Repea	Protein Co	41	GC07P005	1.86
5539	SPATA6	Spermatog	Protein Co	35	GC01M048	1.86
5540	AQP11	Aquaporin	Protein Co	35	GC11P077	1.86
5541	PRND	Prion Like	Protein Co	37	GC20P004	1.86
5542	PPP2CB	Protein Ph	Protein Co	45	GC08M030	1.86
5543	NORAD	Non-Codir	RNA Gene	14	GC20M036	1.86
5544	PTP4A2	Protein Ty	Protein Co	41	GC01M031	1.86
5545	TBXA2R	Thromboxa	Protein Co	48	GC19M003	1.86

5546	ACAP2	ArfGAP Wi	Protein Co	37	GC03M195	1.86
5547	TRD	T Cell Rece	Protein Co	11	GC14P022	1.86
5548	PHB2	Prohibitin	Protein Co	40	GC12M006	1.86
5549	GDF3	Growth Dif	Protein Co	41	GC12M007	1.86
5550	CD300LF	CD300 Mo	Protein Co	39	GC17M074	1.85
5551	SUV39H1	Suppresso	Protein Co	43	GC0XP048	1.85
5552	TRAK1	Trafficking	Protein Co	40	GC03P042	1.85
5553	NMNAT3	Nicotinami	Protein Co	38	GC03M139	1.85
5554	OGA	O-GlcNAca	Protein Co	32	GC10M101	1.85
5555	PARP2	Poly(ADP-I	Protein Co	46	GC14P020	1.85
5556	TSKU	Tsukushi, S	Protein Co	35	GC11P076	1.85
5557	ZNF473	Zinc Finge	Protein Co	37	GC19P050	1.85
5558	lnc-TPD52-3		RNA Gene	4	GC08M080	1.85
5559	PLA2G5	Phospholip	Protein Co	44	GC01P020	1.85
5560	ACOX1	Acyl-CoA (	Protein Co	45	GC17M075	1.85
5561	HSD17B12	Hydroxyste	Protein Co	40	GC11P043	1.85
5562	FADS2	Fatty Acid	Protein Co	44	GC11P061	1.85
5563	PRELP	Proline An	Protein Co	40	GC01P203	1.85
5564	ANKK1	Ankyrin Re	Protein Co	37	GC11P113	1.85
5565	C8G	Compleme	Protein Co	41	GC09P136	1.85
5566	NUDT6	Nudix Hyd	Protein Co	37	GC04M122	1.85
5567	PTMA	Prothymos	Protein Co	39	GC02P231	1.85
5568	ATG12	Autophagy	Protein Co	41	GC05M115	1.85
5569	COPS5	COP9 Sign	Protein Co	42	GC08M067	1.85
5570	ABCD3	ATP Bindir	Protein Co	44	GC01P094	1.85
5571	ECEL1	Endothelin	Protein Co	40	GC02M232	1.85
5572	TPPP3	Tubulin Po	Protein Co	37	GC16M067	1.85
5573	MRPL16	Mitochond	Protein Co	36	GC11M061	1.85
5574	MIR16-2	MicroRNA	RNA Gene	18	GC03P160	1.84
5575	HSP90AB1	Heat Shoc	Protein Co	45	GC06P044	1.84
5576	ARHGEF6	Rac/Cdc42	Protein Co	41	GC0XM136	1.84
5577	GUK1	Guanylate	Protein Co	42	GC01P228	1.84
5578	TRG	T Cell Rece	Protein Co	11	GC07M038	1.84
5579	APBA1	Amyloid B	Protein Co	37	GC09M069	1.84
5580	QARS1	Glutaminyl	Protein Co	26	GC03M049	1.84
5581	MSX1	Msh Home	Protein Co	45	GC04P004	1.84
5582	NDUFA2	NADH:Ubi	Protein Co	42	GC05M140	1.84
5583	DNAJC7	DnaJ Heat	Protein Co	38	GC17M041	1.84
5584	SPEN	Spen Fami	Protein Co	38	GC01P015	1.84
5585	SLC22A8	Solute Car	Protein Co	41	GC11M063	1.84
5586	GCLM	Glutamate	Protein Co	41	GC01M093	1.84
5587	NAXE	NAD(P)HX	Protein Co	34	GC01P156	1.84
5588	GPR180	G Protein-	Protein Co	35	GC13P094	1.83

5589	SERBP1	SERPINE1	Protein Co	35	GC01M067	1.83
5590	ZNF687	Zinc Finge	Protein Co	37	GC01P151	1.83
5591	CEP131	Centrosom	Protein Co	33	GC17M081	1.83
5592	GSK3A	Glycogen S	Protein Co	48	GC19M042	1.83
5593	GSTM2	Glutathion	Protein Co	41	GC01P109	1.83
5594	KCNJ12	Potassium	Protein Co	43	GC17P026	1.83
5595	NSMF	NMDA Rec	Protein Co	38	GC09M137	1.83
5596	CHN1	Chimerin 1	Protein Co	46	GC02M174	1.83
5597	STK33	Serine/Thr	Protein Co	40	GC11M008	1.83
5598	EIF1	Eukaryotic	Protein Co	37	GC17P041	1.83
5599	ZRSR2	Zinc Finge	Protein Co	35	GC0XP015	1.83
5600	EDA	Ectodyspla	Protein Co	41	GC0XP069	1.83
5601	KCNK15-A	KCNK15 A	RNA Gene	13	GC20M044	1.83
5602	ZNF3	Zinc Finge	Protein Co	37	GC07M100	1.83
5603	LEMD2	LEM Doma	Protein Co	39	GC06M033	1.83
5604	SFI1	SFI1 Centri	Protein Co	35	GC22P031	1.83
5605	TAB1	TGF-Beta A	Protein Co	42	GC22P039	1.83
5606	GINS1	GINS Com	Protein Co	39	GC20P025	1.83
5607	WDR1	WD Repea	Protein Co	37	GC04M010	1.83
5608	NACA	Nascent P	Protein Co	38	GC12M056	1.83
5609	IRS4	Insulin Rec	Protein Co	39	GC0XM108	1.83
5610	VAV3	Vav Guanii	Protein Co	43	GC01M107	1.83
5611	SNTA1	Syntrophin	Protein Co	43	GC20M033	1.83
5612	RCOR1	REST Core	Protein Co	40	GC14P102	1.82
5613	H1-4	H1.4 Linke	Protein Co	33	GC06P028	1.82
5614	RNF112	Ring Finge	Protein Co	34	GC17P019	1.82
5615	SMCR5	Smith-Mag	RNA Gene	19	GC17M017	1.82
5616	BHMT	Betaine--H	Protein Co	41	GC05P079	1.82
5617	KCNN1	Potassium	Protein Co	39	GC19P023	1.82
5618	TAB2	TGF-Beta A	Protein Co	47	GC06P149	1.82
5619	SMARCD2	SWI/SNF R	Protein Co	42	GC17M063	1.82
5620	MCEE	Methylmal	Protein Co	43	GC02M071	1.82
5621	CSF2RB	Colony Sti	Protein Co	48	GC22P036	1.82
5622	NUP107	Nucleopor	Protein Co	43	GC12P068	1.82
5623	SLC38A1	Solute Car	Protein Co	43	GC12M046	1.82
5624	PADI2	Peptidyl A	Protein Co	41	GC01M017	1.82
5625	GNL3	G Protein I	Protein Co	40	GC03P052	1.82
5626	SH2D3A	SH2 Doma	Protein Co	35	GC19M006	1.82
5627	TSG101	Tumor Sus	Protein Co	43	GC11M018	1.82
5628	CDYL	Chromodo	Protein Co	40	GC06P004	1.82
5629	MPEG1	Macropha	Protein Co	35	GC11M059	1.82
5630	PLEKHO1	Pleckstrin I	Protein Co	35	GC01P150	1.82
5631	TENT5C	Terminal N	Protein Co	28	GC01P117	1.82

5632	SNORA63E	Small Nucl	RNA Gene	10	GC03P186	1.82
5633	IL11RA	Interleukin	Protein Co	44	GC09P034	1.82
5634	FAR1	Fatty Acyl-	Protein Co	43	GC11P013	1.82
5635	RANBP2	RAN Bindi	Protein Co	45	GC02P108	1.81
5636	CKMT2	Creatine K	Protein Co	43	GC05P081	1.81
5637	POT1	Protection	Protein Co	43	GC07M124	1.81
5638	SERINC1	Serine Incc	Protein Co	40	GC06M122	1.81
5639	CACNA2D	Calcium V	Protein Co	45	GC07M081	1.81
5640	COQ8B	Coenzyme	Protein Co	32	GC19M042	1.81
5641	KPNB1	Karyopheri	Protein Co	43	GC17P047	1.81
5642	LSM4	LSM4 Hom	Protein Co	40	GC19M018	1.81
5643	LILRB1	Leukocyte	Protein Co	43	GC19P055	1.81
5644	GLIPR1	GLI Pathoc	Protein Co	40	GC12P075	1.81
5645	RNF220	Ring Finge	Protein Co	37	GC01P044	1.81
5646	ATP5PB	ATP Synth	Protein Co	30	GC01P111	1.81
5647	GSTA3	Glutathion	Protein Co	44	GC06M052	1.81
5648	ZC3H12A	Zinc Finge	Protein Co	36	GC01P037	1.81
5649	CACNB3	Calcium V	Protein Co	42	GC12P048	1.81
5650	MAPKAPK3	MAPK Acti	Protein Co	49	GC03P050	1.81
5651	SAFB	Scaffold A	Protein Co	38	GC19P005	1.81
5652	LMAN2L	Lectin, Ma	Protein Co	39	GC02M096	1.81
5653	PAPSS2	3'-Phosph	Protein Co	44	GC10P087	1.81
5654	ABR	ABR Activa	Protein Co	40	GC17M001	1.8
5655	ETV5	ETS Varian	Protein Co	39	GC03M186	1.8
5656	HOXA5	Homeobox	Protein Co	41	GC07M027	1.8
5657	ZFPM1	Zinc Finge	Protein Co	37	GC16P088	1.8
5658	IQCA1	IQ Motif C	Protein Co	34	GC02M236	1.8
5659	FRRS1L	Ferric Chel	Protein Co	34	GC09M109	1.8
5660	ATRIP	ATR Intera	Protein Co	41	GC03P048	1.8
5661	PPM1G	Protein Ph	Protein Co	43	GC02M027	1.8
5662	IFNAR2	Interferon	Protein Co	48	GC21P033	1.8
5663	SERPINI1	Serpin Fan	Protein Co	45	GC03P167	1.8
5664	PRSS21	Serine Pro	Protein Co	40	GC16P004	1.8
5665	MIR1-1	MicroRNA	RNA Gene	19	GC20P062	1.8
5666	TECPR2	Tectonin B	Protein Co	35	GC14P102	1.8
5667	RAN	RAN, Mem	Protein Co	45	GC12P130	1.8
5668	PPP1CA	Protein Ph	Protein Co	48	GC11M067	1.8
5669	CHRNE	Cholinergi	Protein Co	43	GC17M004	1.8
5670	BCAN	Brevican	Protein Co	40	GC01P156	1.79
5671	VDAC2	Voltage De	Protein Co	43	GC10P075	1.79
5672	TDP2	Tyrosyl-DN	Protein Co	40	GC06M024	1.79
5673	EEF1AKNM	EEF1A Lysi	Protein Co	30	GC01P171	1.79
5674	TAFA5	TAFA Cher	Protein Co	25	GC22P048	1.79

5675	DPYSL3	Dihydropyı	Protein Co	39	GC05M147	1.79
5676	PSME3	Proteasom	Protein Co	41	GC17P042	1.79
5677	EIF2S2	Eukaryotic	Protein Co	40	GC20M034	1.79
5678	DUSP2	Dual Speci	Protein Co	41	GC02M096	1.79
5679	USP6	Ubiquitin S	Protein Co	40	GC17P005	1.79
5680	CEP126	Centrosom	Protein Co	32	GC11P101	1.79
5681	EIF2S3	Eukaryotic	Protein Co	45	GC0XP024	1.79
5682	SNORA81	Small Nucl	RNA Gene	17	GC03P186	1.79
5683	CCDC85B	Coiled-Coi	Protein Co	33	GC11P065	1.79
5684	KLF2	Kruppel Lil	Protein Co	40	GC19P023	1.79
5685	NDUFA9	NADH:Ubi	Protein Co	43	GC12P004	1.79
5686	SP8	Sp8 Transc	Protein Co	36	GC07M020	1.79
5687	CTSS	Cathepsin	Protein Co	45	GC01M150	1.79
5688	NASP	Nuclear Au	Protein Co	36	GC01P045	1.79
5689	ANLN	Anillin Acti	Protein Co	41	GC07P036	1.79
5690	LRPAP1	LDL Recep	Protein Co	43	GC04M003	1.79
5691	CAMKK2	Calcium/C	Protein Co	45	GC12M121	1.79
5692	MS4A4A	Membrane	Protein Co	34	GC11P060	1.79
5693	VPS4B	Vacuolar P	Protein Co	42	GC18M063	1.79
5694	YRDC	YrdC N6-T	Protein Co	32	GC01M037	1.79
5695	RPS6KA2	Ribosomal	Protein Co	46	GC06M166	1.79
5696	ZNF280D	Zinc Finge	Protein Co	35	GC15M056	1.79
5697	LOC11136	NOS2 5' R	Biological	1	GC17P027	1.79
5698	CD180	CD180 Mo	Protein Co	39	GC05M067	1.78
5699	GABRB1	Gamma-A	Protein Co	44	GC04P046	1.78
5700	IPO5	Importin 5	Protein Co	39	GC13P097	1.78
5701	OPA3	Outer Mitc	Protein Co	39	GC19M045	1.78
5702	INO80	INO80 Cor	Protein Co	36	GC15M040	1.78
5703	HOXB13	Homeobo>	Protein Co	43	GC17M048	1.78
5704	SMARCAL1	SWI/SNF R	Protein Co	43	GC02P216	1.78
5705	IL1RAP	Interleukin	Protein Co	43	GC03P190	1.78
5706	IBA57	Iron-Sulfur	Protein Co	35	GC01P228	1.78
5707	MRTFB	Myocardin	Protein Co	29	GC16P014	1.78
5708	GADD45B	Growth Ar	Protein Co	39	GC19P002	1.78
5709	PSMB10	Proteasom	Protein Co	44	GC16M067	1.78
5710	PITX3	Paired Like	Protein Co	40	GC10M102	1.78
5711	TOX3	TOX High	Protein Co	37	GC16M052	1.78
5712	TECR	Trans-2,3-I	Protein Co	43	GC19P014	1.78
5713	RNF24	Ring Finge	Protein Co	36	GC20M003	1.78
5714	MTHFD2	Methylene	Protein Co	43	GC02P074	1.78
5715	DARS1	Aspartyl-TI	Protein Co	36	GC02M135	1.78
5716	FAM135A	Family Wit	Protein Co	35	GC06P070	1.78
5717	NMNAT1	Nicotinami	Protein Co	48	GC01P009	1.78

5718	NAP1L1	Nucleosom	Protein Co	39	GC12M076	1.78
5719	HELLPAR	HELLP Ass	RNA Gene	14	GC12P102	1.78
5720	NDN	Necdin, M	Protein Co	41	GC15M023	1.77
5721	EIF3I	Eukaryotic	Protein Co	39	GC01P032	1.77
5722	TUBB6	Tubulin Be	Protein Co	41	GC18P012	1.77
5723	MIR615	MicroRNA	RNA Gene	20	GC12P054	1.77
5724	PMVK	Phosphom	Protein Co	45	GC01M154	1.77
5725	YME1L1	YME1 Like	Protein Co	42	GC10M027	1.77
5726	DMTN	Dematin A	Protein Co	36	GC08P022	1.77
5727	SEPTIN1	Septin 1	Protein Co	30	GC16M030	1.77
5728	CARM1	Coactivato	Protein Co	47	GC19P010	1.77
5729	LMAN1	Lectin, Ma	Protein Co	45	GC18M059	1.77
5730	BRPF3	Bromodom	Protein Co	36	GC06P047	1.77
5731	LMO7	LIM Doma	Protein Co	40	GC13P075	1.77
5732	CUL4A	Cullin 4A	Protein Co	41	GC13P113	1.77
5733	ROBO3	Roundabo	Protein Co	43	GC11P124	1.77
5734	MICE	MHC Class	Pseudoger	9	GC06M030	1.76
5735	NECAP1	NECAP En	Protein Co	39	GC12P008	1.76
5736	TRPV2	Transient F	Protein Co	41	GC17P016	1.76
5737	EGLN3	Egl-9 Fami	Protein Co	46	GC14M033	1.76
5738	TMEM176A	Transmem	Protein Co	35	GC07P150	1.76
5739	TSPAN1	Tetraspani	Protein Co	37	GC01P046	1.76
5740	RPL23	Ribosomal	Protein Co	39	GC17M038	1.76
5741	LARGE1	LARGE Xyl	Protein Co	32	GC22M033	1.76
5742	CHRD	Chordin	Protein Co	41	GC03P184	1.76
5743	CDCA5	Cell Divisic	Protein Co	37	GC11M065	1.76
5744	GLDN	Gliomedin	Protein Co	39	GC15P051	1.76
5745	UNC13B	Unc-13 Ho	Protein Co	41	GC09P035	1.76
5746	MCTP2	Multiple C	Protein Co	40	GC15P097	1.76
5747	DLG2	Discs Larg	Protein Co	40	GC11M083	1.76
5748	VPS26A	VPS26, Ret	Protein Co	39	GC10P069	1.76
5749	GLRA1	Glycine Re	Protein Co	46	GC05M151	1.76
5750	HOXD10	Homeobox	Protein Co	40	GC02P176	1.76
5751	EZH1	Enhancer (	Protein Co	45	GC17M042	1.76
5752	PLA2G3	Phospholip	Protein Co	38	GC22M031	1.75
5753	PRPS2	Phosphoril	Protein Co	44	GC0XP012	1.75
5754	PICALM	Phosphatic	Protein Co	43	GC11M085	1.75
5755	LY6E	Lymphocyt	Protein Co	39	GC08P143	1.75
5756	IFIT1	Interferon	Protein Co	39	GC10P089	1.75
5757	ASB2	Ankyrin Re	Protein Co	38	GC14M093	1.75
5758	RPL7AP47	Ribosomal	Pseudoger	9	GC09P014	1.75
5759	CHST6	Carbohydr	Protein Co	43	GC16M075	1.75
5760	SDC4	Syndecan 4	Protein Co	44	GC20M045	1.75

5761	MICAL1	Microtubul	Protein Co	40	GC06M109	1.75
5762	GGA2	Golgi Asso	Protein Co	39	GC16M023	1.75
5763	PTPRT	Protein Tyı	Protein Co	41	GC20M042	1.75
5764	RAMP1	Receptor A	Protein Co	43	GC02P237	1.75
5765	GRINA	Glutamate	Protein Co	34	GC08P143	1.75
5766	NRBF2	Nuclear Re	Protein Co	38	GC10P063	1.75
5767	CXCL3	C-X-C Mot	Protein Co	39	GC04M074	1.75
5768	LDHAL6B	Lactate De	Protein Co	39	GC15P059	1.75
5769	S1PR3	Sphingosir	Protein Co	44	GC09P088	1.75
5770	CALCR	Calcitonin	Protein Co	48	GC07M093	1.74
5771	CIC	Capicua Tr	Protein Co	41	GC19P042	1.74
5772	ECI2	Enoyl-CoA	Protein Co	40	GC06M004	1.74
5773	ATP5MG	ATP Synth	Protein Co	30	GC11P118	1.74
5774	NDUFB2	NADH:Ubi	Protein Co	39	GC07P140	1.74
5775	MAFG	MAF BZIP	Protein Co	40	GC17M081	1.74
5776	DENND2C	DENN Dor	Protein Co	33	GC01M114	1.74
5777	POLR3A	RNA Polyn	Protein Co	43	GC10M077	1.74
5778	DDAH1	Dimethylar	Protein Co	43	GC01M085	1.74
5779	SLC6A2	Solute Car	Protein Co	48	GC16P055	1.74
5780	CNOT3	CCR4-NOT	Protein Co	41	GC19P055	1.74
5781	PSORS1C2	Psoriasis S	Protein Co	31	GC06M031	1.74
5782	CHST11	Carbohydr	Protein Co	41	GC12P104	1.74
5783	BEST1	Bestrophin	Protein Co	43	GC11P061	1.74
5784	APBB1	Amyloid B	Protein Co	43	GC11M006	1.74
5785	GCGR	Glucagon I	Protein Co	47	GC17P081	1.74
5786	LRCH1	Leucine Ri	Protein Co	36	GC13P046	1.74
5787	SEMA4F	Ssemapho	Protein Co	37	GC02P074	1.74
5788	NUCB2	Nucleobin	Protein Co	38	GC11P017	1.73
5789	TNPO1	Transportir	Protein Co	39	GC05P072	1.73
5790	MIR138-1	MicroRNA	RNA Gene	19	GC03P044	1.73
5791	SUMO2	Small Ubic	Protein Co	40	GC17M075	1.73
5792	YBX3	Y-Box Binc	Protein Co	39	GC12M013	1.73
5793	BIK	BCL2 Inter	Protein Co	40	GC22P043	1.73
5794	COL8A1	Collagen T	Protein Co	39	GC03P099	1.73
5795	PNPT1	Polyribonu	Protein Co	42	GC02M055	1.73
5796	TCERG1	Transcripti	Protein Co	39	GC05P146	1.73
5797	MYL12B	Myosin Lig	Protein Co	38	GC18P003	1.73
5798	WDR45B	WD Repea	Protein Co	37	GC17M082	1.73
5799	AKR1C4	Aldo-Keto	Protein Co	47	GC10P005	1.73
5800	ANP32A	Acidic Nuc	Protein Co	43	GC15M068	1.73
5801	SNRPB2	Small Nucl	Protein Co	37	GC20P016	1.73
5802	C6orf62	Chromoso	Protein Co	32	GC06M024	1.73
5803	SLC24A3	Solute Car	Protein Co	39	GC20P019	1.73

5804	TFPI2	Tissue Fact	Protein Co	41	GC07M093	1.73
5805	BCL11A	BAF Chron	Protein Co	43	GC02M060	1.73
5806	IFITM2	Interferon	Protein Co	37	GC11P000	1.73
5807	H3C12	H3 Cluster	Protein Co	29	GC06M028	1.73
5808	KIR2DL3	Killer Cell I	Protein Co	36	GC19P055	1.73
5809	HCG4	HLA Comp	RNA Gene	17	GC06M030	1.73
5810	CREB3L2	CAMP Res	Protein Co	39	GC07M137	1.72
5811	SPATA2	Spermatog	Protein Co	34	GC20M049	1.72
5812	MIR638	MicroRNA	RNA Gene	17	GC19P010	1.72
5813	ATF5	Activating	Protein Co	36	GC19P049	1.72
5814	APH1B	Aph-1 Hor	Protein Co	43	GC15P072	1.72
5815	ING5	Inhibitor C	Protein Co	38	GC02P241	1.72
5816	MTMR3	Myotubula	Protein Co	42	GC22P029	1.72
5817	ARSG	Arylsulfata	Protein Co	43	GC17P068	1.72
5818	NLRP5	NLR Family	Protein Co	37	GC19P056	1.72
5819	NFU1	NFU1 Iron	Protein Co	41	GC02M069	1.72
5820	TRMT10C	TRNA Met	Protein Co	36	GC03P101	1.72
5821	KLHL6	Kelch Like	Protein Co	37	GC03M183	1.72
5822	TERF2IP	TERF2 Inte	Protein Co	42	GC16P075	1.71
5823	PLIN5	Perilipin 5	Protein Co	33	GC19M004	1.71
5824	LDHD	Lactate De	Protein Co	40	GC16M075	1.71
5825	DKK4	Dickkopf V	Protein Co	38	GC08M042	1.71
5826	TNIP2	TNFAIP3 Ir	Protein Co	38	GC04M002	1.71
5827	MIR212	MicroRNA	RNA Gene	19	GC17M002	1.71
5828	RTN1	Reticulon ´	Protein Co	38	GC14M059	1.71
5829	TMBIM6	Transmem	Protein Co	36	GC12P049	1.71
5830	NSL1	NSL1 Com	Protein Co	37	GC01M212	1.71
5831	CASD1	CAS1 Dom	Protein Co	32	GC07P094	1.71
5832	PCA3	Prostate C	RNA Gene	22	GC09P076	1.71
5833	KCNC1	Potassium	Protein Co	43	GC11P017	1.71
5834	CCDC85A	Coiled-Coi	Protein Co	32	GC02P056	1.71
5835	MYL6	Myosin Lig	Protein Co	41	GC12P056	1.71
5836	MIR433	MicroRNA	RNA Gene	21	GC14P104	1.71
5837	NKX2-2	NK2 Home	Protein Co	40	GC20M021	1.71
5838	SERPINB9	Serpin Fan	Protein Co	39	GC06M002	1.71
5839	MIR370	MicroRNA	RNA Gene	20	GC14P104	1.71
5840	ACMSD	Aminocarb	Protein Co	39	GC02P134	1.71
5841	MED23	Mediator (	Protein Co	41	GC06M131	1.71
5842	NAXD	NAD(P)HX	Protein Co	31	GC13P110	1.71
5843	UBQLN2	Ubiquilin 2	Protein Co	42	GC0XP056	1.71
5844	NPRL2	NPR2 Like,	Protein Co	39	GC03M050	1.71
5845	MYOM1	Myomesin	Protein Co	38	GC18M003	1.71
5846	TNFAIP8	TNF Alpha	Protein Co	37	GC05P119	1.71

5847	LARP7	La Ribonuc	Protein Co	41	GC04P112	1.71
5848	PSMC1	Proteasom	Protein Co	42	GC14P090	1.7
5849	CACNA1F	Calcium V	Protein Co	45	GC0XM049	1.7
5850	EGLN1	Egl-9 Fami	Protein Co	48	GC01M231	1.7
5851	TRAT1	T Cell Rece	Protein Co	37	GC03P108	1.7
5852	AKR1E2	Aldo-Keto	Protein Co	37	GC10P004	1.7
5853	IL1RL2	Interleukin	Protein Co	41	GC02P102	1.7
5854	ICA1	Islet Cell A	Protein Co	40	GC07M008	1.7
5855	THEMIS2	Thymocyte	Protein Co	33	GC01P027	1.7
5856	BCKDHB	Branched (	Protein Co	43	GC06P080	1.7
5857	SNX10	Sorting Ne	Protein Co	41	GC07P026	1.7
5858	TRIM25	Tripartite M	Protein Co	44	GC17M056	1.7
5859	UCHL5	Ubiquitin (	Protein Co	42	GC01M193	1.7
5860	APBB1IP	Amyloid B	Protein Co	40	GC10P026	1.7
5861	LXN	Latexin	Protein Co	36	GC03M158	1.7
5862	TRIM13	Tripartite M	Protein Co	36	GC13P049	1.7
5863	BSPRY	B-Box And	Protein Co	34	GC09P113	1.7
5864	FASTK	Fas Activat	Protein Co	39	GC07M151	1.7
5865	ADCY9	Adenylate	Protein Co	44	GC16M003	1.7
5866	GLYCTK	Glycerate k	Protein Co	42	GC03P052	1.7
5867	DHRS7B	Dehydroge	Protein Co	35	GC17P026	1.7
5868	ENSG00000225472		RNA Gene	8	GC09P014	1.7
5869	TBX19	T-Box Tran	Protein Co	40	GC01P168	1.7
5870	TRAPPC12	Trafficking	Protein Co	37	GC02P003	1.7
5871	PGAM1	Phosphogl	Protein Co	43	GC10P097	1.7
5872	ERVW-1	Endogenou	Protein Co	32	GC07M092	1.7
5873	COX8C	Cytochrom	Protein Co	32	GC14P093	1.69
5874	CLK1	CDC Like k	Protein Co	41	GC02M200	1.69
5875	GLIS2	GLIS Famil	Protein Co	40	GC16P004	1.69
5876	DCD	Dermcidin	Protein Co	36	GC12M054	1.69
5877	MAFF	MAF BZIP	Protein Co	40	GC22P038	1.69
5878	EXOC1	Exocyst Co	Protein Co	36	GC04P055	1.69
5879	MIR194-2	MicroRNA	RNA Gene	19	GC11M064	1.69
5880	EXPH5	Exophilin 5	Protein Co	38	GC11M108	1.69
5881	NDUFAF8	NADH:Ubi	Protein Co	24	GC17P081	1.69
5882	SPR	Sepiapterin	Protein Co	50	GC02P072	1.69
5883	ARHGAP29	Rho GTPas	Protein Co	40	GC01M094	1.69
5884	NFATC4	Nuclear Fa	Protein Co	44	GC14P024	1.69
5885	ATP1A1-AS	ATP1A1 Ar	RNA Gene	20	GC01M116	1.69
5886	C9	Compleme	Protein Co	43	GC05M039	1.69
5887	GSTO2	Glutathion	Protein Co	43	GC10P104	1.68
5888	MIB1	Mindbomb	Protein Co	45	GC18P021	1.68
5889	EXOC7	Exocyst Co	Protein Co	40	GC17M076	1.68

5890	VWA1	Von Willeb	Protein Co	35	GC01P001	1.68
5891	GPRC5A	G Protein-(	Protein Co	41	GC12P012	1.68
5892	GMPPB	GDP-Manr	Protein Co	43	GC03M049	1.68
5893	KAT8	Lysine Ace	Protein Co	40	GC16P031	1.68
5894	TRPC4	Transient F	Protein Co	44	GC13M037	1.68
5895	RNPC3	RNA Bindi	Protein Co	35	GC01P103	1.68
5896	UVRAG	UV Radiati	Protein Co	43	GC11P075	1.68
5897	TNR	Tenascin R	Protein Co	41	GC01M175	1.68
5898	CER1	Cerberus 1	Protein Co	40	GC09M014	1.68
5899	EFS	Embryonal	Protein Co	35	GC14M023	1.68
5900	LEO1	LEO1 Hom	Protein Co	37	GC15M060	1.68
5901	ZNF483	Zinc Finge	Protein Co	35	GC09P111	1.68
5902	SRL	Sarcalume	Protein Co	34	GC16M004	1.68
5903	GNB3	G Protein S	Protein Co	47	GC12P006	1.68
5904	HCAR2	Hydroxyca	Protein Co	37	GC12M122	1.68
5905	ASGR2	Asialoglyc	Protein Co	40	GC17M007	1.68
5906	MRAS	Muscle RA	Protein Co	45	GC03P138	1.68
5907	DDX5	DEAD-Box	Protein Co	46	GC17M064	1.68
5908	SLC35A1	Solute Car	Protein Co	40	GC06P087	1.68
5909	ADH7	Alcohol De	Protein Co	44	GC04M099	1.67
5910	PDE2A	Phosphodi	Protein Co	45	GC11M072	1.67
5911	THSD4	Thrombosp	Protein Co	36	GC15P071	1.67
5912	PLN	Phosphola	Protein Co	44	GC06P118	1.67
5913	BOK	BCL2 Fami	Protein Co	40	GC02P241	1.67
5914	ARHGEF12	Rho Guani	Protein Co	43	GC11P120	1.67
5915	SEMA5A	Semaphori	Protein Co	41	GC05M009	1.67
5916	HSD17B10	Hydroxyste	Protein Co	45	GC0XM053	1.67
5917	SUB1	SUB1 Regu	Protein Co	37	GC05P032	1.67
5918	H3C4	H3 Cluster	Protein Co	31	GC06M027	1.67
5919	ODAPH	Odontoge	Protein Co	27	GC04P075	1.67
5920	CKMT1A	Creatine K	Protein Co	37	GC15P043	1.67
5921	REEP1	Receptor A	Protein Co	39	GC02M086	1.67
5922	SLC25A15	Solute Car	Protein Co	44	GC13P040	1.67
5923	PPARGC1B	PPARG Co	Protein Co	35	GC05P149	1.67
5924	BMP5	Bone Morp	Protein Co	41	GC06M055	1.67
5925	SDC2	Syndecan 2	Protein Co	45	GC08P096	1.67
5926	MSRB1	Methionin	Protein Co	40	GC16M001	1.67
5927	GJC1	Gap Juncti	Protein Co	42	GC17M044	1.67
5928	NELFA	Negative E	Protein Co	36	GC04M001	1.67
5929	NDUFS6	NADH:Ubi	Protein Co	43	GC05P001	1.67
5930	DZIP1L	DAZ Intera	Protein Co	36	GC03M138	1.67
5931	MTERF1	Mitochond	Protein Co	34	GC07M091	1.67
5932	TRIM29	Tripartite M	Protein Co	39	GC11M120	1.67

5933	GPER1	G Protein-	Protein Co	37	GC07P001	1.67
5934	RYK	Receptor L	Protein Co	41	GC03M134	1.67
5935	PMS2P2	PMS1 Hon	Pseudoger	18	GC07M075	1.67
5936	FXR1	FMR1 Aut	Protein Co	42	GC03P180	1.67
5937	IGSF3	Immunogl	Protein Co	39	GC01M116	1.66
5938	FYB2	FYN Bindir	Protein Co	24	GC01M056	1.66
5939	VDAC3	Voltage De	Protein Co	43	GC08P042	1.66
5940	EIF2A	Eukaryotic	Protein Co	39	GC03P150	1.66
5941	UHMK1	U2AF Hon	Protein Co	39	GC01P162	1.66
5942	ASNS	Asparagine	Protein Co	46	GC07M097	1.66
5943	TSEN15	TRNA Splic	Protein Co	40	GC01P184	1.66
5944	MRPL19	Mitochond	Protein Co	37	GC02P075	1.66
5945	DOCK9	Dedicator	Protein Co	39	GC13M098	1.66
5946	EED	Embryonic	Protein Co	42	GC11P086	1.66
5947	RIPK4	Receptor I	Protein Co	43	GC21M041	1.66
5948	RAP1GAP	RAP1 GTP	Protein Co	39	GC01M021	1.66
5949	WIZ	WIZ Zinc F	Protein Co	34	GC19M015	1.66
5950	HAP1	Huntingtin	Protein Co	39	GC17M041	1.66
5951	ENSG00000272221		RNA Gene	6	GC06M032	1.66
5952	IQGAP2	IQ Motif C	Protein Co	41	GC05P076	1.66
5953	HOXA7	Homeobo	Protein Co	40	GC07M027	1.66
5954	MIR608	MicroRNA	RNA Gene	17	GC10P100	1.66
5955	ZNF597	Zinc Finge	Protein Co	36	GC16M003	1.66
5956	NFKBIL1	NFKB Inhil	Protein Co	37	GC06P047	1.66
5957	IGF2BP1	Insulin Like	Protein Co	40	GC17P048	1.65
5958	MIR196A2	MicroRNA	RNA Gene	21	GC12P054	1.65
5959	MYBPC1	Myosin Bir	Protein Co	43	GC12P101	1.65
5960	PRKACB	Protein Kir	Protein Co	47	GC01P084	1.65
5961	SESN3	Sestrin 3	Protein Co	39	GC11M095	1.65
5962	PPHLN1	Periphilin	Protein Co	37	GC12P042	1.65
5963	DAND5	DAN Dom	Protein Co	36	GC19P012	1.65
5964	ELP6	Elongator	Protein Co	33	GC03M047	1.65
5965	RPS3	Ribosomal	Protein Co	44	GC11P075	1.65
5966	H1-10	H1.10 Link	Protein Co	28	GC03M129	1.65
5967	UQCRC1	Ubiquinol-	Protein Co	42	GC03M048	1.65
5968	GSTZ1	Glutathion	Protein Co	41	GC14P077	1.65
5969	CGAS	Cyclic GM	Protein Co	29	GC06M073	1.65
5970	RPL36AL	Ribosomal	Protein Co	37	GC14M049	1.65
5971	MYMX	Myomixer,	Protein Co	12	GC06P047	1.65
5972	ST8SIA4	ST8 Alpha-	Protein Co	40	GC05M100	1.65
5973	MTHFS	Methenylte	Protein Co	44	GC15M079	1.65
5974	CREG1	Cellular Re	Protein Co	38	GC01M167	1.65
5975	KLF13	Kruppel Lil	Protein Co	40	GC15P031	1.65

5976	ERCC6L2	ERCC Excis	Protein Co	37	GC09P095	1.65
5977	EPHA1	EPH Recep	Protein Co	47	GC07M143	1.65
5978	NNT	Nicotinami	Protein Co	45	GC05P043	1.65
5979	NSMCE4A	NSE4 Hom	Protein Co	35	GC10M121	1.65
5980	PARP4	Poly(ADP-I	Protein Co	43	GC13M024	1.65
5981	TRIM71	Tripartite M	Protein Co	39	GC03P032	1.64
5982	FARS2	Phenylalan	Protein Co	44	GC06P005	1.64
5983	FXR2	FMR1 Autc	Protein Co	39	GC17M007	1.64
5984	ENSG00000272501		RNA Gene	8	GC06M031	1.64
5985	BBS10	Bardet-Bie	Protein Co	40	GC12M076	1.64
5986	DTNB	Dystrobrev	Protein Co	39	GC02M025	1.64
5987	NDUFA10	NADH:Ubi	Protein Co	44	GC02M239	1.64
5988	DKK2	Dickkopf V	Protein Co	43	GC04M106	1.64
5989	SLC25A19	Solute Car	Protein Co	43	GC17M075	1.64
5990	CKS1B	CDC28 Prc	Protein Co	41	GC01P154	1.64
5991	NAGPA	N-Acetylgl	Protein Co	37	GC16M005	1.64
5992	KLF14	Kruppel Lil	Protein Co	32	GC07M130	1.64
5993	TSPAN16	Tetraspani	Protein Co	32	GC19P011	1.64
5994	PLSCR1	Phospholip	Protein Co	43	GC03M146	1.64
5995	LMX1A	LIM Home	Protein Co	42	GC01M165	1.64
5996	DAO	D-Amino A	Protein Co	45	GC12P108	1.64
5997	RASGEF1B	RasGEF Dc	Protein Co	36	GC04M081	1.64
5998	MYL2	Myosin Lig	Protein Co	49	GC12M110	1.64
5999	SBNO2	Strawberry	Protein Co	32	GC19M001	1.64
6000	CSNK1G1	Casein Kin	Protein Co	43	GC15M064	1.63
6001	PCSK7	Proprotein	Protein Co	43	GC11M117	1.63
6002	POMK	Protein O-	Protein Co	36	GC08P043	1.63
6003	OGN	Osteoglyci	Protein Co	39	GC09M092	1.63
6004	DHDDS	Dehydrodc	Protein Co	43	GC01P026	1.63
6005	BMP3	Bone Morp	Protein Co	42	GC04P081	1.63
6006	QPCT	Glutaminyl	Protein Co	43	GC02P037	1.63
6007	DIS3	DIS3 Hom	Protein Co	40	GC13M072	1.63
6008	NUP37	Nucleopor	Protein Co	37	GC12M102	1.63
6009	GPS2	G Protein I	Protein Co	36	GC17M007	1.63
6010	KLB	Klotho Bet	Protein Co	39	GC04P039	1.63
6011	CLCN6	Chloride V	Protein Co	41	GC01P011	1.63
6012	LOC643801	U3 Small N	Protein Co	16	GC16M053	1.63
6013	SNORA63	Small Nucl	RNA Gene	16	GC03P186	1.63
6014	MN298114-200		RNA Gene	4	GC06M031	1.63
6015	MEIS2	Meis Hom	Protein Co	42	GC15M036	1.63
6016	ANO3	Anoctamin	Protein Co	39	GC11P026	1.63
6017	CDK16	Cyclin Dep	Protein Co	42	GC0XP047	1.63
6018	HLA-DRB4	Major Hist	Protein Co	30	GC06Mo03	1.62

6019	POC1B	POC1 Cent	Protein Co	37	GC12M089	1.62
6020	EPG5	Ectopic P-(	Protein Co	36	GC18M045	1.62
6021	RASGRP3	RAS Guany	Protein Co	41	GC02P033	1.62
6022	MBOAT7	Membrane	Protein Co	40	GC19M054	1.62
6023	MYOF	Myoferlin	Protein Co	40	GC10M093	1.62
6024	ANK1	Ankyrin 1	Protein Co	43	GC08M041	1.62
6025	FRMD7	FERM Dom	Protein Co	39	GC0XM132	1.62
6026	KAAG1	Kidney Ass	Protein Co	28	GC06P024	1.62
6027	DGKB	Diacylglyce	Protein Co	45	GC07M014	1.62
6028	KCTD17	Potassium	Protein Co	40	GC22P037	1.62
6029	HCST	Hematopo	Protein Co	38	GC19P038	1.62
6030	SLC38A3	Solute Car	Protein Co	39	GC03P050	1.62
6031	SIRT4	Sirtuin 4	Protein Co	40	GC12P120	1.62
6032	ZC3H10	Zinc Finge	Protein Co	35	GC12P056	1.62
6033	NOTCH2N	Notch 2 N	Protein Co	26	GC01M146	1.62
6034	ST13	ST13 Hsp7	Protein Co	38	GC22M045	1.62
6035	GPR132	G Protein-(	Protein Co	40	GC14M105	1.62
6036	SLC38A4	Solute Car	Protein Co	40	GC12M046	1.62
6037	CREB3	CAMP Res	Protein Co	38	GC09P035	1.62
6038	LSP1	Lymphocyt	Protein Co	41	GC11P001	1.61
6039	SLC6A13	Solute Car	Protein Co	43	GC12M000	1.61
6040	RF00017-7010		RNA Gene	4	GC08M042	1.61
6041	PGPEP1	Pyroglutan	Protein Co	36	GC19P023	1.61
6042	PLCD1	Phospholip	Protein Co	48	GC03M038	1.61
6043	PPP3R2	Protein Ph	Protein Co	40	GC09M101	1.61
6044	ALOX12B	Arachidona	Protein Co	43	GC17M008	1.61
6045	SMS	Spermine S	Protein Co	44	GC0XP021	1.61
6046	GALP	Galanin Lik	Protein Co	34	GC19P056	1.61
6047	PTPRD	Protein Tyr	Protein Co	44	GC09M008	1.61
6048	CDT1	Chromatin	Protein Co	43	GC16P088	1.61
6049	CAPZA2	Capping A	Protein Co	41	GC07P116	1.61
6050	ETFB	Electron Tr	Protein Co	45	GC19M051	1.61
6051	SIK2	Salt Induci	Protein Co	45	GC11P111	1.61
6052	TRAM2	Translocati	Protein Co	35	GC06M052	1.61
6053	SULT1A4	Sulfotransf	Protein Co	30	GC16P029	1.61
6054	SCN1A-AS	SCN1A An	RNA Gene	12	GC02P165	1.61
6055	SNU13	Small Nucl	Protein Co	34	GC22M045	1.61
6056	JPH4	Junctophili	Protein Co	34	GC14M023	1.61
6057	CIAPIN1	Cytokine Ir	Protein Co	38	GC16M057	1.61
6058	PTBP2	Polypyrimi	Protein Co	39	GC01P096	1.61
6059	UHRF2	Ubiquitin l	Protein Co	39	GC09P006	1.61
6060	GLRB	Glycine Re	Protein Co	48	GC04P157	1.6
6061	ATG14	Autophagy	Protein Co	36	GC14M055	1.6

6062	ARMCX1	Armadillo	Protein Co	31	GC0XP101	1.6
6063	UNC119	Unc-119 Li	Protein Co	41	GC17M028	1.6
6064	CALML3	Calmodulir	Protein Co	42	GC10P005	1.6
6065	TXNL1	Thioredoxi	Protein Co	39	GC18M056	1.6
6066	PFN1	Profilin 1	Protein Co	47	GC17M004	1.6
6067	GABRA6	Gamma-Ar	Protein Co	44	GC05P161	1.6
6068	AMY1A	Amylase A	Protein Co	36	GC01P103	1.6
6069	KYAT1	Kynurenine	Protein Co	33	GC09M128	1.6
6070	KATNAL1	Katanin Ca	Protein Co	37	GC13M030	1.6
6071	CCDC12	Coiled-Coi	Protein Co	33	GC03M046	1.6
6072	ESD	Esterase D	Protein Co	44	GC13M046	1.6
6073	NDUFAF5	NADH:Ubi	Protein Co	36	GC20P013	1.6
6074	NLRC3	NLR Family	Protein Co	34	GC16M003	1.6
6075	KIF23	Kinesin Fa	Protein Co	43	GC15P069	1.6
6076	GORASP2	Golgi Reas	Protein Co	40	GC02P170	1.6
6077	IL4I1	Interleukin	Protein Co	37	GC19M049	1.6
6078	TMSB10	Thymosin	Protein Co	35	GC02P084	1.6
6079	ENSG00000255558		RNA Gene	6	GC11M013	1.59
6080	RIPOR2	RHO Famil	Protein Co	29	GC06M024	1.59
6081	ENDOG	Endonucle	Protein Co	43	GC09P128	1.59
6082	BCKDHA	Branched (	Protein Co	44	GC19P041	1.59
6083	HMBOX1	Homeobox	Protein Co	35	GC08P028	1.59
6084	ZNRF1	Zinc And R	Protein Co	39	GC16P075	1.59
6085	CCR8	C-C Motif	Protein Co	43	GC03P039	1.59
6086	CDR1	Cerebellar	Protein Co	30	GC0XM140	1.59
6087	SYNGR1	Synaptogy	Protein Co	41	GC22P039	1.59
6088	RCE1	Ras Conve	Protein Co	38	GC11P066	1.59
6089	CCDC85C	Coiled-Coi	Protein Co	33	GC14M099	1.59
6090	PIKFYVE	Phosphoin	Protein Co	47	GC02P208	1.59
6091	HEXD	Hexosamir	Protein Co	26	GC17P082	1.59
6092	ADAM8	ADAM Me	Protein Co	42	GC10M133	1.59
6093	AAMP	Angio Ass	Protein Co	36	GC02M218	1.59
6094	MYBL2	MYB Proto	Protein Co	42	GC20P043	1.59
6095	OTOF	Otoferlin	Protein Co	41	GC02M026	1.59
6096	CXorf21	Chromoso	Protein Co	30	GC0XM030	1.59
6097	SARDH	Sarcosine I	Protein Co	42	GC09M133	1.59
6098	MFHAS1	Malignant	Protein Co	35	GC08M008	1.59
6099	APBB3	Amyloid B	Protein Co	38	GC05M140	1.59
6100	MAP3K20	Mitogen-A	Protein Co	39	GC02P173	1.59
6101	PNN	Pinin, Desr	Protein Co	35	GC14P039	1.59
6102	NPFF	Neuropept	Protein Co	35	GC12M053	1.59
6103	HLA-DRB6	Major Hist	Pseudoger	16	GC06M032	1.58
6104	LCP2	Lymphocyt	Protein Co	42	GC05M170	1.58

6105	ALOX15B	Arachidon	Protein Co	41	GC17P008	1.58
6106	ADD3-AS1	ADD3 Anti	RNA Gene	13	GC10M109	1.58
6107	GINS2	GINS Com	Protein Co	36	GC16M085	1.58
6108	GCLC	Glutamate	Protein Co	43	GC06M053	1.58
6109	UBD	Ubiquitin I	Protein Co	39	GC06M029	1.58
6110	HIP1	Huntingtin	Protein Co	41	GC07M075	1.58
6111	COL4A3	Collagen T	Protein Co	44	GC02P227	1.58
6112	D2HGDH	D-2-Hydro	Protein Co	41	GC02P241	1.58
6113	MANF	Mesencepl	Protein Co	39	GC03P051	1.58
6114	COL15A1	Collagen T	Protein Co	38	GC09P098	1.58
6115	TNFSF18	TNF Super	Protein Co	38	GC01M173	1.58
6116	HEXA	Hexosamir	Protein Co	45	GC15M072	1.57
6117	RND1	Rho Family	Protein Co	38	GC12M048	1.57
6118	PDE3A	Phosphodi	Protein Co	48	GC12P020	1.57
6119	SEMA6C	Semaphori	Protein Co	37	GC01M151	1.57
6120	CACNA1I	Calcium V	Protein Co	44	GC22P039	1.57
6121	NDUFA12	NADH:Ubi	Protein Co	44	GC12M094	1.57
6122	INPP5B	Inositol Po	Protein Co	41	GC01M037	1.57
6123	POU2F3	POU Class	Protein Co	38	GC11P120	1.57
6124	NRN1	Neuritin 1	Protein Co	39	GC06M005	1.57
6125	SRP72	Signal Rec	Protein Co	40	GC04P056	1.57
6126	GEN1	GEN1 Holl	Protein Co	39	GC02P017	1.57
6127	FIBP	FGF1 Intra	Protein Co	41	GC11M065	1.57
6128	C1orf220	Chromoso	RNA Gene	24	GC01P178	1.57
6129	RNF168	Ring Finge	Protein Co	43	GC03M196	1.57
6130	EIF2B3	Eukaryotic	Protein Co	42	GC01M044	1.57
6131	NOX5	NADPH O>	Protein Co	37	GC15P072	1.57
6132	POU4F1	POU Class	Protein Co	38	GC13M078	1.57
6133	VPS53	VPS53 Sub	Protein Co	40	GC17M000	1.57
6134	DOCK3	Dedicator	Protein Co	41	GC03P050	1.56
6135	SYT7	Synaptota	Protein Co	39	GC11M061	1.56
6136	MMAB	Metabolisr	Protein Co	45	GC12M109	1.56
6137	NUDT15	Nudix Hyd	Protein Co	37	GC13P048	1.56
6138	UBQLN1	Ubiquilin 1	Protein Co	43	GC09M083	1.56
6139	CSTA	Cystatin A	Protein Co	44	GC03P122	1.56
6140	YBX1P6	Y-Box Binc	Pseudoger	8	GC09M109	1.56
6141	CLN8	CLN8 Tran	Protein Co	40	GC08P001	1.56
6142	PANX1	Pannexin 1	Protein Co	43	GC11P094	1.56
6143	ALAS1	5'-Aminole	Protein Co	44	GC03P052	1.56
6144	PAMR1	Peptidase	Protein Co	36	GC11M035	1.56
6145	CLDN12	Claudin 12	Protein Co	38	GC07P090	1.56
6146	AP3B2	Adaptor R	Protein Co	41	GC15M082	1.56
6147	UBR2	Ubiquitin I	Protein Co	39	GC06P047	1.56

6148	CTCFL	CCCTC-Bin	Protein Co	39	GC20M057	1.56
6149	KCNG1	Potassium	Protein Co	36	GC20M051	1.56
6150	MVD	Mevalonat	Protein Co	45	GC16M088	1.56
6151	EN1	Engrailed I	Protein Co	38	GC02M118	1.56
6152	LMLN	Leishmano	Protein Co	35	GC03P197	1.56
6153	TMEM119	Transmem	Protein Co	33	GC12M108	1.56
6154	ACTN2	Actinin Alp	Protein Co	47	GC01P236	1.56
6155	SGCG	Sarcoglyca	Protein Co	42	GC13P023	1.56
6156	TEX35	Testis Expr	Protein Co	32	GC01P178	1.55
6157	NOL3	Nucleolar I	Protein Co	43	GC16P067	1.55
6158	DRP2	Dystrophir	Protein Co	36	GC0XP101	1.55
6159	DYM	Dymeclin	Protein Co	39	GC18M049	1.55
6160	IFI35	Interferon	Protein Co	37	GC17P043	1.55
6161	PLXNA1	Plexin A1	Protein Co	42	GC03P126	1.55
6162	KPNA1	Karyopheri	Protein Co	42	GC03M122	1.55
6163	GLRX2	Glutaredox	Protein Co	37	GC01M193	1.55
6164	CLEC5A	C-Type Lec	Protein Co	34	GC07M141	1.55
6165	HDAC7	Histone De	Protein Co	45	GC12M047	1.55
6166	SCAP	SREBF Cha	Protein Co	41	GC03M047	1.55
6167	DHTKD1	Dehydroge	Protein Co	42	GC10P012	1.55
6168	MIR575	MicroRNA	RNA Gene	16	GC04M082	1.55
6169	NMUR2	Neuromed	Protein Co	39	GC05M152	1.55
6170	GPD1	Glycerol-3-	Protein Co	45	GC12P050	1.55
6171	HNRNPAB	Heterogen	Protein Co	38	GC05P178	1.55
6172	PDK4	Pyruvate D	Protein Co	45	GC07M095	1.55
6173	BAHD1	Bromo Adj	Protein Co	35	GC15P040	1.55
6174	RGMB	Repulsive (	Protein Co	38	GC05P098	1.55
6175	YWHAG	Tyrosine 3-	Protein Co	49	GC07M076	1.55
6176	SRGN	Serglycin	Protein Co	38	GC10P069	1.55
6177	RBM20	RNA Bindi	Protein Co	35	GC10P110	1.55
6178	PTRH1	Peptidyl-TI	Protein Co	33	GC09M127	1.55
6179	XAF1	XIAP Asso	Protein Co	39	GC17P006	1.55
6180	DOCK2	Dedicator	Protein Co	44	GC05P169	1.55
6181	CPA6	Carboxype	Protein Co	43	GC08M067	1.55
6182	RPS6KA6	Ribosomal	Protein Co	43	GC0XM084	1.55
6183	NBR2	Neighbor (	RNA Gene	25	GC17P043	1.55
6184	CLIC5	Chloride Ir	Protein Co	43	GC06M045	1.55
6185	EEF1D	Eukaryotic	Protein Co	42	GC08M143	1.55
6186	LMX1B	LIM Home	Protein Co	45	GC09P126	1.55
6187	PDLIM7	PDZ And L	Protein Co	40	GC05M177	1.55
6188	GJB3	Gap Juncti	Protein Co	44	GC01P034	1.55
6189	CYP4F22	Cytochrom	Protein Co	39	GC19P015	1.55
6190	RIN3	Ras And R	Protein Co	37	GC14P092	1.54

6191	MARS2	Methionyl-	Protein Co	42	GC02P197	1.54
6192	DCHS2	Dachsous (	Protein Co	33	GC04M154	1.54
6193	CSH2	Chorionic !	Protein Co	32	GC17M063	1.54
6194	CEP164	Centrosom	Protein Co	40	GC11P117	1.54
6195	ACTL8	Actin Like	Protein Co	33	GC01P017	1.54
6196	MIR600	MicroRNA	RNA Gene	14	GC09M123	1.54
6197	SELENOW	Selenoprot	Protein Co	29	GC19P047	1.54
6198	DBI	Diazepam	Protein Co	45	GC02P119	1.54
6199	BBS9	Bardet-Bie	Protein Co	39	GC07P033	1.54
6200	B4GALNT1	Beta-1,4-N	Protein Co	44	GC12M057	1.54
6201	HLA-DQB2	Major Hist	Protein Co	36	GC06M032	1.54
6202	PSORS1C3	Psoriasis S	RNA Gene	21	GC06M031	1.54
6203	PITRM1	Pitrilysin M	Protein Co	39	GC10M003	1.54
6204	SIAH1	Siah E3 Ub	Protein Co	45	GC16M048	1.54
6205	GABRG3	Gamma-A	Protein Co	42	GC15P026	1.54
6206	CCDC144A	Coiled-Coi	Protein Co	27	GC17P016	1.54
6207	CHCHD10	Coiled-Coi	Protein Co	39	GC22M023	1.54
6208	COL4A4	Collagen T	Protein Co	43	GC02M226	1.54
6209	LILRB3	Leukocyte	Protein Co	39	GC19M054	1.54
6210	CA6	Carbonic A	Protein Co	42	GC01P008	1.54
6211	ELAVL4	ELAV Like	Protein Co	39	GC01P050	1.54
6212	LOXL3	Lysyl Oxid	Protein Co	41	GC02M074	1.54
6213	GPT2	Glutamic--	Protein Co	46	GC16P046	1.53
6214	MEOX2	Mesenchyr	Protein Co	41	GC07M015	1.53
6215	SOCS6	Suppresso	Protein Co	41	GC18P070	1.53
6216	ORC1	Origin Rec	Protein Co	43	GC01M052	1.53
6217	CORO1B	Coronin 1E	Protein Co	36	GC11M067	1.53
6218	INPP5A	Inositol Po	Protein Co	43	GC10P132	1.53
6219	COQ5	Coenzyme	Protein Co	39	GC12M120	1.53
6220	CALML5	Calmodulir	Protein Co	37	GC10M005	1.53
6221	HORMAD2	HORMA D	Protein Co	35	GC22P030	1.53
6222	CYGB	Cytoglobin	Protein Co	38	GC17M076	1.53
6223	ADAMTS8	ADAM Me	Protein Co	39	GC11M130	1.53
6224	YPEL2	Yippee Lik	Protein Co	33	GC17P059	1.53
6225	TRPM2	Transient F	Protein Co	43	GC21P044	1.53
6226	SPTSSB	Serine Palr	Protein Co	32	GC03M161	1.53
6227	SFRP4	Secreted F	Protein Co	43	GC07M037	1.53
6228	KCNH1	Potassium	Protein Co	46	GC01M210	1.53
6229	PLEKHM1	Pleckstrin I	Protein Co	41	GC17M045	1.53
6230	TRIM65	Tripartite M	Protein Co	36	GC17M075	1.53
6231	SLC25A46	Solute Car	Protein Co	39	GC05P110	1.53
6232	RARRES2	Retinoic A	Protein Co	39	GC07M150	1.53
6233	MIR92B	MicroRNA	RNA Gene	16	GC01P155	1.53

6234	RGL2	Ral Guanin	Protein Co	39	GC06M033	1.53
6235	LRIG3	Leucine Ri	Protein Co	37	GC12M058	1.53
6236	SKA2	Spindle An	Protein Co	36	GC17M059	1.53
6237	ZNF142	Zinc Finge	Protein Co	35	GC02M218	1.53
6238	RPS7	Ribosomal	Protein Co	42	GC02P003	1.53
6239	MZF1	Myeloid Zi	Protein Co	35	GC19M058	1.53
6240	PDK1	Pyruvate D	Protein Co	47	GC02P172	1.53
6241	SAP30BP	SAP30 Bin	Protein Co	37	GC17P075	1.53
6242	SEPTIN5	Septin 5	Protein Co	32	GC22P019	1.53
6243	ALX4	ALX Home	Protein Co	39	GC11M044	1.53
6244	SMUG1	Single-Stra	Protein Co	42	GC12M054	1.52
6245	TBCCD1	TBCC Dom	Protein Co	35	GC03M186	1.52
6246	ENSG00000231083		RNA Gene	7	GC02M008	1.52
6247	ENSG00000254205		RNA Gene	7	GC08P080	1.52
6248	LOC10537	Uncharacte	RNA Gene	3	GC02M008	1.52
6249	LHX2	LIM Home	Protein Co	36	GC09P124	1.52
6250	TUBGCP6	Tubulin Ga	Protein Co	39	GC22M050	1.52
6251	ASCC2	Activating	Protein Co	35	GC22M029	1.52
6252	HSD3B2	Hydroxy-D	Protein Co	46	GC01P119	1.52
6253	KLF9	Kruppel Lil	Protein Co	36	GC09M070	1.52
6254	SFMBT1	Scm Like V	Protein Co	37	GC03M052	1.52
6255	MIR503	MicroRNA	RNA Gene	17	GC0XM134	1.52
6256	DAGLA	Diacylglyce	Protein Co	41	GC11P061	1.52
6257	FTCD	Formimido	Protein Co	43	GC21M047	1.52
6258	ALG10B	ALG10 Alp	Protein Co	33	GC12P038	1.52
6259	DNMBP	Dynamin B	Protein Co	39	GC10M099	1.52
6260	MOSPD3	Motile Spe	Protein Co	33	GC07P100	1.52
6261	PTPRN2	Protein Ty	Protein Co	44	GC07M157	1.52
6262	TRHR	Thyrotropi	Protein Co	43	GC08P109	1.52
6263	NMB	Neuromed	Protein Co	41	GC15M084	1.52
6264	SEMA6D	Semaphori	Protein Co	39	GC15P047	1.52
6265	GRIN3B	Glutamate	Protein Co	40	GC19P001	1.52
6266	BLVRA	Biliverdin I	Protein Co	45	GC07P043	1.52
6267	AKAP1	A-Kinase A	Protein Co	39	GC17P057	1.52
6268	PYGL	Glycogen I	Protein Co	48	GC14M050	1.52
6269	GFM2	GTP Deper	Protein Co	39	GC05M074	1.52
6270	PCDH8	Protocadh	Protein Co	39	GC13M052	1.52
6271	TMX2	Thioredoxi	Protein Co	36	GC11P057	1.52
6272	TCF15	Transcripti	Protein Co	33	GC20M000	1.52
6273	FZR1	Fizzy And	Protein Co	42	GC19P003	1.52
6274	CA5A	Carbonic A	Protein Co	43	GC16M087	1.51
6275	EMG1	EMG1 N1-	Protein Co	41	GC12P006	1.51
6276	TIMM10	Translocas	Protein Co	36	GC11M061	1.51

6277	PAX4	Paired Box	Protein Co	43	GC07M127	1.51
6278	LDHB	Lactate De	Protein Co	46	GC12M021	1.51
6279	STRADA	STE20 Rela	Protein Co	45	GC17M063	1.51
6280	WNT8A	Wnt Family	Protein Co	41	GC05P138	1.51
6281	CYP4F2	Cytochrom	Protein Co	44	GC19M015	1.51
6282	SLC17A9	Solute Car	Protein Co	39	GC20P062	1.51
6283	HOXA11	Homeobox	Protein Co	41	GC07M027	1.51
6284	OTX1	Orthodent	Protein Co	41	GC02P063	1.51
6285	PKN1	Protein Kir	Protein Co	45	GC19P014	1.51
6286	RAB18	RAB18, Me	Protein Co	40	GC10P027	1.51
6287	KDM3A	Lysine Den	Protein Co	41	GC02P086	1.51
6288	C1orf194	Chromosor	Protein Co	29	GC01M109	1.51
6289	FOXN2	Forkhead E	Protein Co	33	GC02P048	1.51
6290	MAB21L1	Mab-21 Lik	Protein Co	38	GC13M035	1.51
6291	PHPT1	Phosphohi	Protein Co	37	GC09P136	1.51
6292	GAB1	GRB2 Assc	Protein Co	44	GC04P143	1.51
6293	CYB561D2	Cytochrom	Protein Co	33	GC03P050	1.51
6294	LMCD1-AS	LMCD1 An	RNA Gene	13	GC03M007	1.51
6295	MIR208A	MicroRNA	RNA Gene	18	GC14M023	1.51
6296	OTUB1	OTU Deub	Protein Co	39	GC11P063	1.51
6297	DGKI	Diacylglyce	Protein Co	41	GC07M137	1.5
6298	KANSL2	KAT8 Regu	Protein Co	34	GC12M048	1.5
6299	SATB1	SATB Hom	Protein Co	42	GC03M018	1.5
6300	VOPP1	VOPP1 WV	Protein Co	32	GC07M055	1.5
6301	HCRTR1	Hypocretin	Protein Co	43	GC01P031	1.5
6302	KRT86	Keratin 86	Protein Co	39	GC12P052	1.5
6303	H3C8	H3 Cluster	Protein Co	28	GC06M027	1.5
6304	LINC00996	Long Inter	RNA Gene	15	GC07P150	1.5
6305	FLRT3	Fibronectir	Protein Co	44	GC20M014	1.5
6306	PSPH	Phosphose	Protein Co	47	GC07M056	1.5
6307	SSH1	Slingshot F	Protein Co	41	GC12M108	1.5
6308	PLPP1	Phospholip	Protein Co	33	GC05M055	1.5
6309	CLP1	Cleavage F	Protein Co	40	GC11P057	1.5
6310	CHD6	Chromodo	Protein Co	37	GC20M041	1.5
6311	FAT1	FAT Atypic	Protein Co	39	GC04M186	1.5
6312	SSTR4	Somatosta	Protein Co	41	GC20P023	1.5
6313	ARPC5	Actin Relat	Protein Co	40	GC01M183	1.5
6314	PRR12	Proline Ric	Protein Co	32	GC19P049	1.5
6315	CYS1	Cystin 1	Protein Co	30	GC02M010	1.49
6316	PIK3C2B	Phosphatic	Protein Co	47	GC01M204	1.49
6317	RYR2	Ryanodine	Protein Co	47	GC01P237	1.49
6318	ARL6	ADP Ribos	Protein Co	42	GC03P097	1.49
6319	AGXT2	Alanine--G	Protein Co	40	GC05M034	1.49

6320	MAD2L1	Mitotic Arr	Protein Co	45	GC04M12(	1.49
6321	NFE2	Nuclear Fa	Protein Co	40	GC12M054	1.49
6322	GNB2	G Protein S	Protein Co	42	GC07P100(	1.49
6323	RPL4	Ribosomal	Protein Co	41	GC15M066	1.49
6324	ADGRG6	Adhesion (	Protein Co	38	GC06P142	1.49
6325	HSPBAP1	HSPB1 Ass	Protein Co	36	GC03M122	1.49
6326	EIF4G2	Eukaryotic	Protein Co	42	GC11M01(	1.49
6327	FGF3	Fibroblast	Protein Co	44	GC11M069	1.49
6328	NGB	Neuroglob	Protein Co	37	GC14M077	1.49
6329	RS1	Retinoschi	Protein Co	40	GC0XM018	1.49
6330	SMCR2	Smith-Mag	RNA Gene	13	GC17M017	1.49
6331	CCNI	Cyclin I	Protein Co	37	GC04M077	1.49
6332	DENR	Density Re	Protein Co	37	GC12P122	1.48
6333	TMED3	Transmem	Protein Co	35	GC15P079	1.48
6334	H3C10	H3 Cluster	Protein Co	29	GC06P028	1.48
6335	ELK3	ETS Transc	Protein Co	39	GC12P096	1.48
6336	ZC3H12D	Zinc Finge	Protein Co	33	GC06M149	1.48
6337	ZNF622	Zinc Finge	Protein Co	35	GC05M016	1.48
6338	ZGPAT	Zinc Finge	Protein Co	37	GC20P063	1.48
6339	EIF5A	Eukaryotic	Protein Co	43	GC17P007	1.48
6340	CRLF2	Cytokine R	Protein Co	39	GC0XM001	1.48
6341	TUT4	Terminal U	Protein Co	28	GC01M052	1.48
6342	SEMA6B	Semaphori	Protein Co	37	GC19M004	1.48
6343	NPY1R	Neuropept	Protein Co	45	GC04M163	1.48
6344	RABEP2	Rabaptin, I	Protein Co	35	GC16M028	1.48
6345	NDUFAF6	NADH:Ubi	Protein Co	35	GC08P094	1.48
6346	MACIR	Macropha	Protein Co	25	GC05P103	1.48
6347	KCND3	Potassium	Protein Co	46	GC01M111	1.48
6348	NDUFS5	NADH:Ubi	Protein Co	40	GC01P039(	1.48
6349	DCPS	Decapping	Protein Co	43	GC11P126	1.48
6350	ALOX5AP	Arachidon	Protein Co	44	GC13P030	1.48
6351	TTBK1	Tau Tubuli	Protein Co	35	GC06P043	1.48
6352	MYOZ2	Myozenin	Protein Co	39	GC04P119	1.48
6353	AVPR1A	Arginine V	Protein Co	45	GC12M063	1.48
6354	CDC34	Cell Divisic	Protein Co	47	GC19P000	1.48
6355	SPG21	SPG21 Abl	Protein Co	40	GC15M064	1.48
6356	PWWP2B	PWWP Do	Protein Co	31	GC10P132	1.48
6357	TRIM68	Tripartite M	Protein Co	37	GC11M004	1.48
6358	KCTD13	Potassium	Protein Co	35	GC16M029	1.48
6359	PREX1	Phosphatic	Protein Co	43	GC20M048	1.48
6360	KIF5A	Kinesin Fa	Protein Co	44	GC12P057	1.48
6361	SNORA4	Small Nucl	RNA Gene	16	GC03P186	1.48
6362	BPIFA1	BPI Fold C	Protein Co	36	GC20P033	1.48

6363	SH3GLB1	SH3 Doma	Protein Co	40	GC01P086	1.48
6364	LRRC15	Leucine Ri	Protein Co	36	GC03M194	1.47
6365	ADAMTS1!	ADAM Me	Protein Co	36	GC11P130	1.47
6366	PLSCR3	Phospholip	Protein Co	37	GC17M007	1.47
6367	TRIM24	Tripartite M	Protein Co	43	GC07P138	1.47
6368	CC2D1A	Coiled-Coi	Protein Co	39	GC19P013	1.47
6369	COLEC12	Collectin S	Protein Co	39	GC18M000	1.47
6370	JADE1	Jade Famil	Protein Co	35	GC04P128	1.47
6371	MYOZ3	Myozenin	Protein Co	33	GC05P150	1.47
6372	WTAP	WT1 Asso	Protein Co	39	GC06P159	1.47
6373	DNAJB1	DnaJ Heat	Protein Co	44	GC19M014	1.47
6374	DIO2	Iodothyror	Protein Co	40	GC14M080	1.47
6375	KISS1	KiSS-1 Me	Protein Co	41	GC01M204	1.47
6376	NUDC	Nuclear Di	Protein Co	41	GC01P026	1.47
6377	ZP3	Zona Pellu	Protein Co	41	GC07P076	1.47
6378	FOXD4	Forkhead I	Protein Co	33	GC09M000	1.47
6379	G3BP2	G3BP Stres	Protein Co	36	GC04M075	1.47
6380	NLRC5	NLR Family	Protein Co	39	GC16P056	1.47
6381	EDF1	Endothelia	Protein Co	39	GC09M136	1.47
6382	ZNF488	Zinc Finge	Protein Co	29	GC10M047	1.47
6383	SDR9C7	Short Chai	Protein Co	39	GC12M056	1.47
6384	SIM2	SIM BHLH	Protein Co	38	GC21P036	1.47
6385	TENM4	Teneurin T	Protein Co	34	GC11M078	1.47
6386	ZNF862	Zinc Finge	Protein Co	29	GC07P149	1.47
6387	NDUFA8	NADH:Ubi	Protein Co	41	GC09M122	1.47
6388	FKBP1B	FKBP Proly	Protein Co	40	GC02P024	1.47
6389	NSUN3	NOP2/Sun	Protein Co	36	GC03P094	1.47
6390	DMRT3	Doublesex	Protein Co	33	GC09P000	1.47
6391	GNA14	G Protein S	Protein Co	43	GC09M077	1.47
6392	MT4	Metallothi	Protein Co	33	GC16P056	1.47
6393	TIMMDC1	Translocas	Protein Co	36	GC03P119	1.47
6394	ASIC2	Acid Sensi	Protein Co	39	GC17M033	1.47
6395	NNMT	Nicotinami	Protein Co	45	GC11P114	1.47
6396	G3BP1	G3BP Stres	Protein Co	40	GC05P151	1.47
6397	DNASE2	Deoxyribo	Protein Co	39	GC19M012	1.47
6398	ENO3	Enolase 3	Protein Co	48	GC17P004	1.46
6399	SDCBP	Syndecan I	Protein Co	41	GC08P058	1.46
6400	DDHD1	DDHD Dor	Protein Co	38	GC14M053	1.46
6401	ZFYVE27	Zinc Finge	Protein Co	37	GC10P097	1.46
6402	TMCO6	Transmem	Protein Co	32	GC05P143	1.46
6403	BNC2	Basonuclin	Protein Co	39	GC09M016	1.46
6404	SLC7A3	Solute Car	Protein Co	38	GC0XM070	1.46
6405	CNN1	Calponin 1	Protein Co	41	GC19P011	1.46

6406	GABPB1	GA Bindin	Protein Co	38	GC15M050	1.46
6407	ULK1	Unc-51 Lik	Protein Co	45	GC12P131	1.46
6408	NKX3-1	NK3 Home	Protein Co	41	GC08M023	1.46
6409	MIR346	MicroRNA	RNA Gene	18	GC10M086	1.46
6410	FCGR2C	Fc Fragme	Protein Co	34	GC01P161	1.46
6411	CLEC2D	C-Type Le	Protein Co	36	GC12P010	1.46
6412	ZNF281	Zinc Finge	Protein Co	37	GC01M200	1.46
6413	MAT2B	Methionin	Protein Co	40	GC05P163	1.46
6414	PLGLB1	Plasminog	Protein Co	33	GC02M087	1.46
6415	TRMT44	TRNA Met	Protein Co	32	GC04P008	1.46
6416	DLX4	Distal-Less	Protein Co	39	GC17P049	1.46
6417	LRRC41	Leucine Ri	Protein Co	35	GC01M046	1.46
6418	SNORD94	Small Nucl	RNA Gene	16	GC02P086	1.46
6419	ACBD3	Acyl-CoA E	Protein Co	39	GC01M226	1.45
6420	MIR583	MicroRNA	RNA Gene	15	GC05P096	1.45
6421	CPA3	Carboxype	Protein Co	40	GC03P148	1.45
6422	CD53	CD53 Mol	Protein Co	39	GC01P110	1.45
6423	NR2C1	Nuclear Re	Protein Co	39	GC12M095	1.45
6424	CHAMP1	Chromoso	Protein Co	37	GC13P114	1.45
6425	TBC1D7	TBC1 Dom	Protein Co	41	GC06M013	1.45
6426	THUMPD1	THUMP D	Protein Co	36	GC16M020	1.45
6427	AAR2	AAR2 Splic	Protein Co	35	GC20P036	1.45
6428	TMEM219	Transmem	Protein Co	33	GC16P030	1.45
6429	SCG3	Secretogra	Protein Co	40	GC15P051	1.45
6430	ELK1	ETS Transc	Protein Co	44	GC0XM047	1.45
6431	KCNJ3	Potassium	Protein Co	45	GC02P154	1.45
6432	EXOSC9	Exosome C	Protein Co	40	GC04P121	1.45
6433	GPRIN1	G Protein I	Protein Co	33	GC05M176	1.45
6434	ADGRL2	Adhesion G	Protein Co	37	GC01P081	1.45
6435	PPP3R1	Protein Ph	Protein Co	45	GC02M068	1.45
6436	NBPF6	NBPF Mem	Protein Co	29	GC01P108	1.45
6437	PSTPIP2	Proline-Se	Protein Co	36	GC18M045	1.45
6438	ATP6V0A1	ATPase H+	Protein Co	43	GC17P042	1.45
6439	TMPRSS13	Transmem	Protein Co	37	GC11M117	1.44
6440	DONSON	DNA Repli	Protein Co	35	GC21M033	1.44
6441	GPR27	G Protein-	Protein Co	35	GC03P071	1.44
6442	CPM	Carboxype	Protein Co	42	GC12M068	1.44
6443	SPAAR	Small Regu	Protein Co	14	GC09P035	1.44
6444	NDUFAF4	NADH:Ubi	Protein Co	42	GC06M096	1.44
6445	GID4	GID Comp	Protein Co	31	GC17P018	1.44
6446	MIR708	MicroRNA	RNA Gene	17	GC11M079	1.44
6447	RN7SL41P	RNA, 7SL,	Pseudoger	7	GC08P080	1.44
6448	lnc-ZBTB10-6		RNA Gene	5	GC08P080	1.44

6449	RF00017-7145		RNA Gene	4	GC08P080	1.44
6450	FITM2	Fat Storag	Protein Co	35	GC20M044	1.44
6451	PGRMC1	Progesterc	Protein Co	44	GC0XP119	1.44
6452	RTKN	Rhotekin	Protein Co	39	GC02M074	1.44
6453	MIR383	MicroRNA	RNA Gene	15	GC08M014	1.44
6454	WDFY4	WDFY Fam	Protein Co	35	GC10P048	1.44
6455	MIR557	MicroRNA	RNA Gene	16	GC01P168	1.44
6456	TBX18	T-Box Tran	Protein Co	41	GC06M084	1.44
6457	VPS28	VPS28 Sub	Protein Co	39	GC08M144	1.44
6458	LIPI	Lipase I	Protein Co	37	GC21M014	1.44
6459	ZNF608	Zinc Finge	Protein Co	35	GC05M124	1.44
6460	TANK	TRAF Fami	Protein Co	43	GC02P161	1.44
6461	RNF6	Ring Finge	Protein Co	39	GC13M026	1.44
6462	TSPYL2	TSPY Like	Protein Co	36	GC0XP053	1.44
6463	NR4A3	Nuclear Re	Protein Co	45	GC09P099	1.44
6464	PAK2	P21 (RAC1	Protein Co	46	GC03P196	1.44
6465	CHRNA1	Cholinergi	Protein Co	45	GC02M174	1.44
6466	MAPK7	Mitogen-A	Protein Co	48	GC17P019	1.44
6467	CLCF1	Cardiotrop	Protein Co	41	GC11M067	1.44
6468	TMEM37	Transmem	Protein Co	32	GC02P119	1.43
6469	VSNL1	Visinin Lik	Protein Co	41	GC02P017	1.43
6470	H2BC13	H2B Cluste	Protein Co	27	GC06M027	1.43
6471	LAGE3	L Antigen	Protein Co	36	GC0XM154	1.43
6472	SIVA1	SIVA1 Apo	Protein Co	36	GC14P104	1.43
6473	GJA8	Gap Juncti	Protein Co	45	GC01P147	1.43
6474	ACACB	Acetyl-CoA	Protein Co	45	GC12P109	1.43
6475	GFPT1	Glutamine	Protein Co	46	GC02M069	1.43
6476	HBP1	HMG-Box	Protein Co	38	GC07P107	1.43
6477	CSRP2	Cysteine A	Protein Co	39	GC12M076	1.43
6478	ARHGEF9	Cdc42 Gua	Protein Co	41	GC0XM063	1.43
6479	NSMCE3	NSE3 Hom	Protein Co	31	GC15M029	1.43
6480	GPR4	G Protein-	Protein Co	39	GC19M045	1.43
6481	CELF4	CUGBP Ela	Protein Co	38	GC18M037	1.43
6482	CDH11	Cadherin 1	Protein Co	48	GC16M064	1.43
6483	LATS2	Large Tum	Protein Co	44	GC13M020	1.43
6484	PYCR2	Pyrroline-5	Protein Co	44	GC01M225	1.43
6485	TBC1D20	TBC1 Dom	Protein Co	38	GC20M000	1.43
6486	TULP4	TUB Like P	Protein Co	33	GC06P158	1.43
6487	ABTB2	Ankyrin Re	Protein Co	32	GC11M034	1.43
6488	SRY	Sex Deterr	Protein Co	35	GC0YM002	1.43
6489	HMGB3	High Mobi	Protein Co	41	GC0XP150	1.43
6490	SMARCA1	SWI/SNF R	Protein Co	39	GC0XM129	1.43
6491	RHOJ	Ras Homo	Protein Co	38	GC14P063	1.43

6492	DNAJC13	DnaJ Heat	Protein Co	37	GC03P132	1.43
6493	CBX7	Chromobo	Protein Co	37	GC22M045	1.43
6494	LCN1	Lipocalin 1	Protein Co	39	GC09P135	1.43
6495	PISD	Phosphatic	Protein Co	45	GC22M031	1.43
6496	DDT	D-Dopachı	Protein Co	39	GC22M023	1.43
6497	RNF182	Ring Finge	Protein Co	35	GC06P013	1.42
6498	ADAM11	ADAM Me	Protein Co	40	GC17P044	1.42
6499	ATP5F1B	ATP Synth	Protein Co	33	GC12M056	1.42
6500	ORC6	Origin Rec	Protein Co	40	GC16P046	1.42
6501	CCAR1	Cell Divisic	Protein Co	36	GC10P068	1.42
6502	PCDHGB4	Protocadh	Protein Co	32	GC05P143	1.42
6503	STAU1	Staufen Dc	Protein Co	37	GC20M049	1.42
6504	RCAN2	Regulator	Protein Co	40	GC06M046	1.42
6505	RSF1	Remodelin	Protein Co	35	GC11M077	1.42
6506	UROS	Uroporphy	Protein Co	43	GC10M125	1.42
6507	HDAC11	Histone De	Protein Co	42	GC03P013	1.42
6508	SERAC1	Serine Acti	Protein Co	36	GC06M158	1.42
6509	FGF14	Fibroblast	Protein Co	44	GC13M101	1.41
6510	CTNNBIP1	Catenin Be	Protein Co	41	GC01M009	1.41
6511	LILRB4	Leukocyte	Protein Co	39	GC19P054	1.41
6512	GNMT	Glycine N-	Protein Co	45	GC06P042	1.41
6513	AGPAT5	1-Acylglyc	Protein Co	41	GC08P006	1.41
6514	TBX20	T-Box Tran	Protein Co	41	GC07M035	1.41
6515	HTR2C	5-Hydroxy	Protein Co	48	GC0XP114	1.41
6516	PNPLA8	Patatin Lik	Protein Co	42	GC07M108	1.41
6517	TSPEAR	Thrombos	Protein Co	32	GC21M044	1.41
6518	MIR148B	MicroRNA	RNA Gene	20	GC12P054	1.41
6519	UBIAD1	UbiA Preny	Protein Co	41	GC01P011	1.41
6520	MAD1L1	Mitotic Arr	Protein Co	45	GC07M001	1.41
6521	NPY5R	Neuropept	Protein Co	42	GC04P163	1.41
6522	H3-4	H3.4 Histo	Protein Co	32	GC01M228	1.41
6523	PEA15	Proliferatic	Protein Co	43	GC01P160	1.41
6524	S100A2	S100 Calci	Protein Co	40	GC01M153	1.41
6525	OGFR	Opioid Grc	Protein Co	37	GC20P062	1.41
6526	FPR3	Formyl Pep	Protein Co	40	GC19P051	1.41
6527	GNPTG	N-Acetylgl	Protein Co	39	GC16P001	1.41
6528	POLR3B	RNA Polyn	Protein Co	43	GC12P106	1.41
6529	GLRX5	Glutaredox	Protein Co	40	GC14P095	1.41
6530	ISCA1	Iron-Sulfur	Protein Co	38	GC09M086	1.41
6531	ARRDC1-A	ARRDC1 A	RNA Gene	24	GC09M137	1.4
6532	INPP5J	Inositol Po	Protein Co	40	GC22P031	1.4
6533	SMAD5	SMAD Fan	Protein Co	41	GC05P136	1.4
6534	ZNF212	Zinc Finge	Protein Co	37	GC07P149	1.4

6535	CLCNKB	Chloride V	Protein Co	44	GC01P016	1.4
6536	TSR2	TSR2 Ribo	Protein Co	37	GC0XP054	1.4
6537	PPP4C	Protein Ph	Protein Co	45	GC16P030	1.4
6538	MC3R	Melanocor	Protein Co	43	GC20P056	1.4
6539	PLEKHB1	Pleckstrin	Protein Co	36	GC11P073	1.4
6540	ARV1	ARV1 Hom	Protein Co	36	GC01P230	1.4
6541	CHST1	Carbohydr	Protein Co	37	GC11M045	1.4
6542	CAMKV	CaM Kinas	Protein Co	38	GC03M049	1.4
6543	TRPC5	Transient F	Protein Co	41	GC0XM111	1.4
6544	CINP	Cyclin Dep	Protein Co	35	GC14M102	1.4
6545	KMT5C	Lysine Met	Protein Co	29	GC19P055	1.4
6546	MAP11	Microtubul	Protein Co	28	GC07M100	1.4
6547	H2BC12	H2B Cluste	Protein Co	28	GC06M027	1.4
6548	SPOCK1	SPARC (Os	Protein Co	39	GC05M136	1.4
6549	CELA2A	Chymotryp	Protein Co	39	GC01P015	1.4
6550	SV2B	Synaptic V	Protein Co	40	GC15P091	1.4
6551	PANX2	Pannexin 2	Protein Co	37	GC22P050	1.4
6552	TRAF4	TNF Recep	Protein Co	43	GC17P028	1.4
6553	PARG	Poly(ADP-I	Protein Co	39	GC10M049	1.4
6554	GMFB	Glia Matur	Protein Co	40	GC14M054	1.4
6555	TRIB3	Tribbles Ps	Protein Co	43	GC20P000	1.4
6556	MMRN1	Multimerin	Protein Co	39	GC04P089	1.39
6557	ZNF365	Zinc Finge	Protein Co	37	GC10P062	1.39
6558	HUS1	HUS1 Che	Protein Co	41	GC07M047	1.39
6559	FAM219B	Family Wit	Protein Co	30	GC15M074	1.39
6560	UPK3B	Uroplakin	Protein Co	27	GC07P076	1.39
6561	PLPP3	Phospholip	Protein Co	35	GC01M056	1.39
6562	lnc-RBL2-7		RNA Gene	4	GC16P053	1.39
6563	DERL3	Derlin 3	Protein Co	36	GC22M023	1.39
6564	VAV2	Vav Guanii	Protein Co	43	GC09M133	1.39
6565	PLPP2	Phospholip	Protein Co	32	GC19M000	1.39
6566	PURB	Purine Rich	Protein Co	36	GC07M044	1.39
6567	KNOP1	Lysine Rich	Protein Co	29	GC16M019	1.39
6568	APTR	Alu-Media	RNA Gene	15	GC07M077	1.39
6569	MYHAS	Myosin He	RNA Gene	10	GC17P010	1.39
6570	LMO4	LIM Doma	Protein Co	39	GC01P087	1.39
6571	SYNPO2	Synaptopo	Protein Co	35	GC04P118	1.39
6572	GJA3	Gap Juncti	Protein Co	41	GC13M020	1.38
6573	MIR101-1	MicroRNA	RNA Gene	18	GC01M065	1.38
6574	C4B_2	Compleme	Protein Co	16	GC06Po03	1.38
6575	MMP15	Matrix Me	Protein Co	45	GC16P058	1.38
6576	NRG2	Neuregulir	Protein Co	40	GC05M139	1.38
6577	RND2	Rho Family	Protein Co	36	GC17P043	1.38

6578	SYPL2	Synaptoph	Protein Co	36	GC01P109	1.38
6579	HCN2	Hyperpola	Protein Co	45	GC19P000	1.38
6580	STAC3	SH3 And C	Protein Co	36	GC12M057	1.38
6581	ZNF80	Zinc Finge	Protein Co	32	GC03M114	1.38
6582	EFNA5	Ephrin A5	Protein Co	43	GC05M107	1.38
6583	KRT24	Keratin 24	Protein Co	36	GC17M040	1.38
6584	PAWR	Pro-Apopt	Protein Co	40	GC12M079	1.38
6585	MPP1	Membrane	Protein Co	40	GC0XM154	1.38
6586	ERN2	Endoplasm	Protein Co	37	GC16M023	1.38
6587	CDC42SE2	CDC42 Sm	Protein Co	34	GC05P131	1.38
6588	INPP4A	Inositol Po	Protein Co	42	GC02P098	1.38
6589	FBXO38	F-Box Prot	Protein Co	37	GC05P148	1.38
6590	PLD2	Phospholip	Protein Co	47	GC17P004	1.37
6591	MIR26B	MicroRNA	RNA Gene	21	GC02P218	1.37
6592	EPHB1	EPH Recep	Protein Co	47	GC03P134	1.37
6593	SLC18A1	Solute Car	Protein Co	45	GC08M020	1.37
6594	ENSG00000251867		RNA Gene	7	GC08M080	1.37
6595	CDK5R1	Cyclin Dep	Protein Co	45	GC17P032	1.37
6596	SBF1	SET Bindin	Protein Co	41	GC22M050	1.37
6597	TSEN34	TRNA Splic	Protein Co	39	GC19P055	1.37
6598	SPRED1	Sprouty Re	Protein Co	42	GC15P038	1.37
6599	UCMA	Upper Zon	Protein Co	32	GC10M013	1.37
6600	CYP4A11	Cytochrom	Protein Co	43	GC01M046	1.37
6601	HPD	4-Hydroxy	Protein Co	45	GC12M121	1.37
6602	BLMH	Bleomycin	Protein Co	43	GC17M030	1.37
6603	RAB3GAP2	RAB3 GTPa	Protein Co	39	GC01M220	1.37
6604	DBT	Dihydrolip	Protein Co	43	GC01M100	1.37
6605	ARHGEF3	Rho Guani	Protein Co	41	GC03M056	1.37
6606	HSD17B4	Hydroxyste	Protein Co	46	GC05P119	1.37
6607	KCNK18	Potassium	Protein Co	36	GC10P117	1.37
6608	SRP9	Signal Rec	Protein Co	35	GC01P225	1.37
6609	KATNB1	Katanin Re	Protein Co	40	GC16P057	1.37
6610	ACOT12	Acyl-CoA T	Protein Co	38	GC05M081	1.37
6611	DGKQ	Diacylglyce	Protein Co	41	GC04M000	1.36
6612	HNRNPC	Heterogen	Protein Co	41	GC14M021	1.36
6613	SPAST	Spastin	Protein Co	40	GC02P032	1.36
6614	SIGMAR1	Sigma Nor	Protein Co	46	GC09M034	1.36
6615	PHF1	PHD Finge	Protein Co	40	GC06P047	1.36
6616	LRRC37A3	Leucine Ri	Protein Co	31	GC17M064	1.36
6617	PLA2R1	Phospholip	Protein Co	39	GC02M159	1.36
6618	CDKN2D	Cyclin Dep	Protein Co	40	GC19M010	1.36
6619	RORB	RAR Relate	Protein Co	46	GC09P074	1.36
6620	JPH1	Junctophili	Protein Co	39	GC08M074	1.36

6621	NRSN1	Neurensin	Protein Co	35	GC06P024	1.36
6622	SLC13A3	Solute Car	Protein Co	43	GC20M046	1.36
6623	CCDC88B	Coiled-Coi	Protein Co	33	GC11P064	1.36
6624	HEXIM1	HEXIM P-T	Protein Co	38	GC17P045	1.36
6625	CEP72	Centrosom	Protein Co	36	GC05P000	1.36
6626	MON2	MON2 Ho	Protein Co	35	GC12P062	1.36
6627	ZNF443	Zinc Finge	Protein Co	32	GC19M012	1.36
6628	CLEC11A	C-Type Le	Protein Co	39	GC19P050	1.36
6629	EPN3	Epsin 3	Protein Co	36	GC17P050	1.36
6630	ARID3A	AT-Rich In	Protein Co	39	GC19P000	1.36
6631	SNX13	Sorting Ne	Protein Co	36	GC07M017	1.36
6632	NONHSAG	034457.2	RNA Gene	4	GC03P012	1.36
6633	TTI2	TELO2 Inte	Protein Co	36	GC08M033	1.36
6634	SERPINA5	Serpin Fan	Protein Co	43	GC14P094	1.35
6635	MOBP	Myelin Ass	Protein Co	36	GC03P039	1.35
6636	BCKDK	Branched (	Protein Co	45	GC16P031	1.35
6637	HYAL2	Hyaluronic	Protein Co	41	GC03M050	1.35
6638	B4GALT6	Beta-1,4-G	Protein Co	40	GC18M031	1.35
6639	SPON2	Spondin 2	Protein Co	39	GC04M001	1.35
6640	PDK3	Pyruvate D	Protein Co	47	GC0XP024	1.35
6641	CELSR2	Cadherin E	Protein Co	40	GC01P109	1.35
6642	ZNF23	Zinc Finge	Protein Co	38	GC16M071	1.35
6643	GCFC2	GC-Rich Se	Protein Co	33	GC02M075	1.35
6644	USP5	Ubiquitin S	Protein Co	41	GC12P008	1.35
6645	EEF2	Eukaryotic	Protein Co	48	GC19M003	1.35
6646	CRB2	Crumbs Ce	Protein Co	40	GC09P123	1.35
6647	BAG5	BAG Cocha	Protein Co	39	GC14M103	1.35
6648	ASXL2	ASXL Trans	Protein Co	39	GC02M025	1.35
6649	FOXO4	Forkhead E	Protein Co	43	GC0XP071	1.35
6650	RHOT1	Ras Homo	Protein Co	43	GC17P032	1.35
6651	CTPS1	CTP Synth	Protein Co	44	GC01P040	1.35
6652	H3C11	H3 Cluster	Protein Co	28	GC06M028	1.35
6653	LINC00944	Long Inter	RNA Gene	13	GC12M126	1.35
6654	RNF2	Ring Finge	Protein Co	42	GC01P185	1.35
6655	ZNF354A	Zinc Finge	Protein Co	37	GC05M178	1.35
6656	GABRR3	Gamma-A	Protein Co	36	GC03M097	1.35
6657	MIR298	MicroRNA	RNA Gene	16	GC20M058	1.35
6658	USH2A-AS	USH2A An	RNA Gene	8	GC01P216	1.35
6659	DOCK7	Dedicator	Protein Co	41	GC01M062	1.35
6660	FBXO31	F-Box Prot	Protein Co	36	GC16M087	1.35
6661	IL1F10	Interleukin	Protein Co	39	GC02P113	1.35
6662	COMMD5	COMM Do	Protein Co	32	GC08M144	1.35
6663	PCM1	Pericentrio	Protein Co	41	GC08P017	1.34

6664	DALRD3	DALR Anti	Protein Co	34	GC03M049	1.34
6665	CNTD1	Cyclin N-T	Protein Co	33	GC17P042	1.34
6666	OLIG1	Oligodend	Protein Co	38	GC21P033	1.34
6667	CCPG1	Cell Cycle	Protein Co	35	GC15M055	1.34
6668	ADH6	Alcohol De	Protein Co	40	GC04M099	1.34
6669	ADCK2	AarF Dom	Protein Co	35	GC07P140	1.34
6670	CAMTA2	Calmodulir	Protein Co	33	GC17M004	1.34
6671	PIP5K1C	Phosphatic	Protein Co	48	GC19M003	1.34
6672	MOAP1	Modulator	Protein Co	36	GC14M093	1.34
6673	CYP21A1P	Cytochrom	Pseudoger	15	GC06P032	1.34
6674	UGDH-AS1	UGDH Ant	RNA Gene	13	GC04P039	1.34
6675	ENSG00000255458		RNA Gene	8	GC04P039	1.34
6676	MIR642A	MicroRNA	RNA Gene	16	GC19P045	1.34
6677	MIR500A	MicroRNA	RNA Gene	16	GC0XP050	1.34
6678	RGS2	Regulator	Protein Co	43	GC01P192	1.34
6679	TGIF2	TGFB Indu	Protein Co	39	GC20P036	1.34
6680	NPAT	Nuclear Pr	Protein Co	35	GC11M108	1.33
6681	HTR3C	5-Hydroxy	Protein Co	37	GC03P184	1.33
6682	HRK	Harakiri, B	Protein Co	39	GC12M116	1.33
6683	CHMP5	Charged M	Protein Co	40	GC09P033	1.33
6684	GLRA2	Glycine Re	Protein Co	44	GC0XP014	1.33
6685	STAC2	SH3 And C	Protein Co	36	GC17M039	1.33
6686	MIDEAS	Mitotic De	Protein Co	24	GC14M073	1.33
6687	IMMP2L	Inner Mito	Protein Co	39	GC07M110	1.33
6688	RD3	Retinal De	Protein Co	38	GC01M211	1.33
6689	AKAP4	A-Kinase A	Protein Co	37	GC0XM050	1.33
6690	FXYD4	FXYD Dom	Protein Co	35	GC10P043	1.33
6691	ARHGEF18	Rho/Rac G	Protein Co	43	GC19P007	1.33
6692	RNF8	Ring Finge	Protein Co	41	GC06P047	1.33
6693	SIRPB1	Signal Reg	Protein Co	39	GC20M001	1.33
6694	FUT1	Fucosyltrar	Protein Co	40	GC19M048	1.33
6695	ICMT	Isoprenylcy	Protein Co	41	GC01M006	1.33
6696	TRIM27	Tripartite M	Protein Co	41	GC06M028	1.33
6697	TFAP4	Transcripti	Protein Co	37	GC16M004	1.33
6698	ARL6IP1	ADP Ribos	Protein Co	40	GC16M018	1.33
6699	DDX6	DEAD-Box	Protein Co	45	GC11M118	1.32
6700	SELENOH	Selenoprot	Protein Co	27	GC11P057	1.32
6701	TRIM58	Tripartite M	Protein Co	35	GC01P247	1.32
6702	AOPEP	Aminopep	Protein Co	29	GC09P094	1.32
6703	SLC43A3	Solute Car	Protein Co	37	GC11M061	1.32
6704	TNNI3K	TNNI3 Inte	Protein Co	40	GC01P074	1.32
6705	EGLN2	Egl-9 Fami	Protein Co	44	GC19P040	1.32
6706	ACTL6B	Actin Like	Protein Co	41	GC07M100	1.32

6707	CEP63	Centrosom	Protein Co	40	GC03P134	1.32
6708	PLEKHA8	Pleckstrin I	Protein Co	36	GC07P030	1.32
6709	OR10H2	Olfactory F	Protein Co	32	GC19P015	1.32
6710	LINC00977	Long Inter	RNA Gene	14	GC08M128	1.32
6711	LINC00824	Long Inter	RNA Gene	12	GC08M128	1.32
6712	SRD5A1	Steroid 5 A	Protein Co	44	GC05P006	1.32
6713	IFITM3	Interferon	Protein Co	41	GC11M000	1.32
6714	ADAMTS2(	ADAM Me	Protein Co	36	GC12M043	1.32
6715	H2BC6	H2B Cluste	Protein Co	28	GC06P028	1.32
6716	MECR	Mitochond	Protein Co	44	GC01M029	1.32
6717	CELSR1	Cadherin E	Protein Co	41	GC22M046	1.32
6718	RUSC2	RUN And S	Protein Co	37	GC09P035	1.32
6719	LNP1	Leukemia I	Protein Co	31	GC03P100	1.32
6720	TVP23B	Trans-Golg	Protein Co	30	GC17P018	1.32
6721	EP400	E1A Bindin	Protein Co	36	GC12P131	1.32
6722	MIR4274	MicroRNA	RNA Gene	11	GC04P007	1.32
6723	TP53I3	Tumor Pro	Protein Co	41	GC02M024	1.32
6724	CYC1	Cytochrom	Protein Co	45	GC08P144	1.32
6725	PPP1R9A	Protein Ph	Protein Co	38	GC07P094	1.32
6726	UNC93B1	Unc-93 Ho	Protein Co	39	GC11M067	1.32
6727	FAM3A	FAM3 Met	Protein Co	35	GC0XM154	1.31
6728	HAO1	Hydroxyac	Protein Co	44	GC20M007	1.31
6729	ARID3B	AT-Rich In	Protein Co	38	GC15P074	1.31
6730	TMLHE	Trimethylly	Protein Co	43	GC0XM155	1.31
6731	HCRTR2	Hypocretin	Protein Co	44	GC06P055	1.31
6732	PROX2	Prospero H	Protein Co	31	GC14M074	1.31
6733	MMP25	Matrix Met	Protein Co	39	GC16P004	1.31
6734	CENPU	Centromer	Protein Co	37	GC04M184	1.31
6735	TNFSF8	TNF Super	Protein Co	37	GC09M114	1.31
6736	C2CD4A	C2 Calciun	Protein Co	30	GC15P062	1.31
6737	SLU7	SLU7 Hom	Protein Co	37	GC05M160	1.31
6738	GJB5	Gap Juncti	Protein Co	40	GC01P034	1.31
6739	PRDM8	PR/SET Do	Protein Co	36	GC04P080	1.31
6740	FCRL4	Fc Recepto	Protein Co	37	GC01M157	1.31
6741	TRMT10A	TRNA Met	Protein Co	36	GC04M099	1.31
6742	CCDC14	Coiled-Coi	Protein Co	35	GC03M123	1.31
6743	SLC6A9	Solute Car	Protein Co	48	GC01M043	1.31
6744	SEPTIN4	Septin 4	Protein Co	30	GC17M058	1.31
6745	OSCAR	Osteoclast	Protein Co	38	GC19M054	1.31
6746	SIPA1L2	Signal Indu	Protein Co	38	GC01M232	1.31
6747	ANKRD12	Ankyrin Re	Protein Co	35	GC18P009	1.31
6748	ISLR2	Immunogl	Protein Co	35	GC15P074	1.31
6749	RMDN3	Regulator	Protein Co	33	GC15M040	1.31

6750	GABRA3	Gamma-A	Protein Co	44	GC0XM152	1.3
6751	ATP5F1E	ATP Synth	Protein Co	32	GC20M059	1.3
6752	KIF5C	Kinesin Fa	Protein Co	40	GC02P148	1.3
6753	LINC00958	Long Inter	RNA Gene	16	GC11M012	1.3
6754	MCPH1	Microceph	Protein Co	40	GC08P006	1.3
6755	DSG4	Desmoglei	Protein Co	40	GC18P031	1.3
6756	KCNK4	Potassium	Protein Co	40	GC11P064	1.3
6757	ANKRD26	Ankyrin Re	Protein Co	39	GC10M026	1.3
6758	H2BS1	H2B.S Hist	Protein Co	22	GC21P043	1.3
6759	CELF3	CUGBP Ela	Protein Co	35	GC01M151	1.3
6760	LRRC37A	Leucine Ri	Protein Co	31	GC17P046	1.3
6761	ZNRD1ASP	Zinc Ribbo	Pseudoger	14	GC06M030	1.3
6762	LRRC32	Leucine Ri	Protein Co	37	GC11M076	1.3
6763	MYT1	Myelin Tra	Protein Co	40	GC20P064	1.3
6764	KLF3	Kruppel Lil	Protein Co	39	GC04P038	1.3
6765	HOPX	HOP Hom	Protein Co	37	GC04M056	1.3
6766	MIR484	MicroRNA	RNA Gene	18	GC16P015	1.3
6767	MIR658	MicroRNA	RNA Gene	14	GC22M045	1.3
6768	MIR611	MicroRNA	RNA Gene	14	GC11M061	1.3
6769	MIR596	MicroRNA	RNA Gene	13	GC08P001	1.3
6770	NEBL	Nebulette	Protein Co	39	GC10M020	1.3
6771	RAB33A	RAB33A, M	Protein Co	36	GC0XP130	1.29
6772	TASOR	Transcripti	Protein Co	27	GC03M056	1.29
6773	DACH1	Dachshunc	Protein Co	39	GC13M071	1.29
6774	EGFL7	EGF Like D	Protein Co	39	GC09P136	1.29
6775	KCP	Kielin Cyst	Protein Co	33	GC07M128	1.29
6776	AK1	Adenylate	Protein Co	47	GC09M127	1.29
6777	PLD4	Phospholip	Protein Co	39	GC14P104	1.29
6778	GJA4	Gap Juncti	Protein Co	43	GC01P034	1.29
6779	NAV3	Neuron Na	Protein Co	33	GC12P077	1.29
6780	TNNI1	Troponin I	Protein Co	40	GC01M201	1.29
6781	MASTL	Microtubul	Protein Co	42	GC10P027	1.29
6782	UPK2	Uroplakin	Protein Co	37	GC11P118	1.29
6783	PITPNC1	Phosphatic	Protein Co	36	GC17P067	1.29
6784	CDIP1	Cell Death	Protein Co	35	GC16M004	1.29
6785	RGCC	Regulator	Protein Co	34	GC13P041	1.29
6786	SAMD9L	Sterile Alp	Protein Co	36	GC07M093	1.29
6787	TCEAL1	Transcripti	Protein Co	37	GC0XP103	1.29
6788	G0S2	G0/G1 Swi	Protein Co	32	GC01P209	1.28
6789	IDDM15	Insulin Dep	Genetic Lo	3	GC06U990	1.28
6790	MIR7-1	MicroRNA	RNA Gene	18	GC09M084	1.28
6791	APOBEC3G	Apolipopro	Protein Co	41	GC22P039	1.28
6792	TSBP1	Testis Expr	Protein Co	23	GC06M032	1.28

6793	POLD2	DNA Polyr	Protein Co	40	GC07M044	1.28
6794	XPNPEP3	X-Prolyl Ar	Protein Co	41	GC22P040	1.28
6795	IRAK2	Interleukin	Protein Co	41	GC03P010	1.28
6796	FBRS	Fibrosin	Protein Co	32	GC16P030	1.28
6797	ENSG00000255018		Pseudoger	4	GC11P013	1.28
6798	RHNO1	RAD9-HUS	Protein Co	31	GC12P002	1.28
6799	BBS7	Bardet-Bie	Protein Co	38	GC04M121	1.28
6800	ENSG00000288061		RNA Gene	6	GC11P123	1.28
6801	ENSG00000233996		Pseudoger	4	GC02M189	1.28
6802	GADD45G	Growth Ar	Protein Co	40	GC09P089	1.28
6803	COX20	Cytochrom	Protein Co	39	GC01P244	1.28
6804	DIP2A	Disco Inter	Protein Co	39	GC21P046	1.28
6805	PDE12	Phosphodi	Protein Co	35	GC03P057	1.28
6806	KAZN	Kazrin, Per	Protein Co	33	GC01P013	1.28
6807	SDSL	Serine Del	Protein Co	40	GC12P113	1.28
6808	OLAH	Oleoyl-ACI	Protein Co	35	GC10P015	1.28
6809	ITSN1	Intersectin	Protein Co	42	GC21P033	1.28
6810	DSPP	Dentin Sial	Protein Co	37	GC04P087	1.28
6811	SRI	Sorcin	Protein Co	42	GC07M088	1.28
6812	SDC3	Syndecan 3	Protein Co	42	GC01M030	1.28
6813	HIF3A	Hypoxia In	Protein Co	39	GC19P046	1.28
6814	MIR518B	MicroRNA	RNA Gene	16	GC19P054	1.28
6815	MIR601	MicroRNA	RNA Gene	16	GC09M123	1.28
6816	MIR769	MicroRNA	RNA Gene	16	GC19P046	1.28
6817	ENSG00000261220		RNA Gene	8	GC08P133	1.27
6818	PAPOLG	Poly(A) Pol	Protein Co	39	GC02P060	1.27
6819	TFPT	TCF3 Fusic	Protein Co	35	GC19M054	1.27
6820	GPR17	G Protein-(	Protein Co	40	GC02P127	1.27
6821	PLA2G4C	Phospholip	Protein Co	43	GC19M048	1.27
6822	TSPAN32	Tetraspani	Protein Co	36	GC11P002	1.27
6823	HSBP1	Heat Shoc	Protein Co	34	GC16P083	1.27
6824	TPRKB	TP53RK Bi	Protein Co	39	GC02M073	1.27
6825	CKAP2L	Cytoskelet	Protein Co	37	GC02M112	1.27
6826	TMEM203	Transmem	Protein Co	31	GC09M137	1.27
6827	RNASE1	Ribonuclea	Protein Co	40	GC14M020	1.27
6828	TAS1R2	Taste 1 Re	Protein Co	37	GC01M018	1.27
6829	UBE2D3	Ubiquitin (	Protein Co	47	GC04M102	1.27
6830	MRPL28	Mitochond	Protein Co	37	GC16M000	1.27
6831	LOC11167	CFTR -35 k	Biological	1	GC07P117	1.27
6832	LOC11167	CFTR -44 k	Biological	1	GC07P117	1.27
6833	KCNJ18	Potassium	Protein Co	26	GC17P026	1.27
6834	RAE1	Ribonuclei	Protein Co	40	GC20P057	1.27
6835	LINGO1	Leucine Ri	Protein Co	44	GC15M077	1.27

6836	MYNN	Myoneurin	Protein Co	37	GC03P169	1.26
6837	POLI	DNA Polyr	Protein Co	41	GC18P054	1.26
6838	PHF3	PHD Finge	Protein Co	36	GC06P063	1.26
6839	BHLHE23	Basic Helix	Protein Co	32	GC20M063	1.26
6840	GSAP	Gamma-Se	Protein Co	32	GC07M077	1.26
6841	CRYGN	Crystallin (	Protein Co	31	GC07M151	1.26
6842	PHETA1	PH Domair	Protein Co	27	GC12M111	1.26
6843	RALBP1	RalA Bindi	Protein Co	44	GC18P009	1.26
6844	NREP	Neuronal F	Protein Co	35	GC05M111	1.26
6845	KCNK9	Potassium	Protein Co	46	GC08M139	1.26
6846	KREMEN1	Kringle Co	Protein Co	43	GC22P029	1.26
6847	TNMD	Tenomodu	Protein Co	33	GC0XP100	1.26
6848	SIRT2	Sirtuin 2	Protein Co	48	GC19M038	1.26
6849	PTGFRN	Prostaglan	Protein Co	38	GC01P116	1.26
6850	GH2	Growth Hc	Protein Co	39	GC17M063	1.26
6851	MIR325	MicroRNA	RNA Gene	13	GC0XM077	1.26
6852	TSPAN18	Tetraspani	Protein Co	35	GC11P044	1.26
6853	IL5RA	Interleukin	Protein Co	46	GC03M003	1.26
6854	ATG10	Autophagy	Protein Co	39	GC05P081	1.26
6855	CUEDC2	CUE Doma	Protein Co	38	GC10M102	1.26
6856	TRDN	Triadin	Protein Co	41	GC06M123	1.26
6857	SENP7	SUMO Spe	Protein Co	36	GC03M101	1.26
6858	PRAM1	PML-RARA	Protein Co	34	GC19M008	1.26
6859	IQCK	IQ Motif C	Protein Co	33	GC16P019	1.26
6860	TOR1AIP2	Torsin 1A I	Protein Co	33	GC01M181	1.26
6861	SPRED2	Sprouty Re	Protein Co	41	GC02M065	1.26
6862	DEFA3	Defensin A	Protein Co	36	GC08M007	1.26
6863	RPH3A	Rabphilin 3	Protein Co	39	GC12P112	1.25
6864	EXOG	Exo/Endon	Protein Co	37	GC03P038	1.25
6865	ACOD1	Aconitate I	Protein Co	26	GC13P076	1.25
6866	PPP1R13L	Protein Ph	Protein Co	39	GC19M045	1.25
6867	AFM	Afamin	Protein Co	38	GC04P073	1.25
6868	TRIM47	Tripartite M	Protein Co	34	GC17M075	1.25
6869	FRAT1	FRAT Regu	Protein Co	39	GC10P097	1.25
6870	NANP	N-Acetylne	Protein Co	37	GC20M025	1.25
6871	EIF4E3	Eukaryotic	Protein Co	36	GC03M071	1.25
6872	KBTBD13	Kelch Repe	Protein Co	32	GC15P072	1.25
6873	TIGD4	Tigger Tra	Protein Co	32	GC04M152	1.25
6874	OBP2A	Odorant B	Protein Co	33	GC09P135	1.25
6875	SNORD1C	Small Nucl	RNA Gene	13	GC17P076	1.25
6876	ENSG00000207300		RNA Gene	6	GC06P137	1.25
6877	TGM5	Transgluta	Protein Co	41	GC15M043	1.25
6878	THOP1	Thimet Oli	Protein Co	41	GC19P002	1.25

6879	BPIFB4	BPI Fold C	Protein Co	32	GC20P033	1.24
6880	NCK2	NCK Adap	Protein Co	43	GC02P105	1.24
6881	SIGLEC8	Sialic Acid	Protein Co	39	GC19M051	1.24
6882	TDG	Thymine D	Protein Co	44	GC12P103	1.24
6883	ARHGEF17	Rho Guani	Protein Co	37	GC11P073	1.24
6884	HCN4	Hyperpola	Protein Co	47	GC15M073	1.24
6885	PHF8	PHD Finge	Protein Co	41	GC0XM053	1.24
6886	DENND5A	DENN Dor	Protein Co	37	GC11M009	1.24
6887	PKHD1L1	PKHD1 Lik	Protein Co	34	GC08P109	1.24
6888	ASB14	Ankyrin Re	Protein Co	32	GC03M057	1.24
6889	TMEM150	Transmem	Protein Co	28	GC04M082	1.24
6890	CDRT1	CMT1A Du	Protein Co	27	GC17M015	1.24
6891	FNDC5	Fibronectir	Protein Co	34	GC01M032	1.24
6892	F2RL2	Coagulatio	Protein Co	41	GC05M076	1.24
6893	PHF6	PHD Finge	Protein Co	40	GC0XP134	1.24
6894	SAMD8	Sterile Alp	Protein Co	37	GC10P075	1.24
6895	SHROOM4	Shroom Fa	Protein Co	35	GC0XM050	1.24
6896	MIR199A2	MicroRNA	RNA Gene	19	GC01M172	1.24
6897	PTPRO	Protein Ty	Protein Co	45	GC12P015	1.24
6898	ADARB1	Adenosine	Protein Co	43	GC21P045	1.24
6899	NECTIN3	Nectin Cel	Protein Co	33	GC03P111	1.24
6900	PTPRB	Protein Ty	Protein Co	45	GC12M070	1.24
6901	SOCS4	Suppresso	Protein Co	37	GC14P055	1.24
6902	UBE2L6	Ubiquitin (	Protein Co	40	GC11M061	1.24
6903	KCTD9	Potassium	Protein Co	35	GC08M025	1.24
6904	LYRM4	LYR Motif	Protein Co	38	GC06M005	1.24
6905	NCAPG2	Non-SMC	Protein Co	37	GC07M158	1.24
6906	SRARP	Steroid Re	Protein Co	25	GC01P016	1.24
6907	MFAP5	Microfibril	Protein Co	41	GC12M008	1.23
6908	TAAR1	Trace Amir	Protein Co	37	GC06M132	1.23
6909	GAL3ST1	Galactose-	Protein Co	37	GC22M030	1.23
6910	AASS	Aminoadip	Protein Co	44	GC07M122	1.23
6911	PDZRN3	PDZ Doma	Protein Co	35	GC03M073	1.23
6912	ELP3	Elongator	Protein Co	39	GC08P028	1.23
6913	KCND2	Potassium	Protein Co	43	GC07P120	1.23
6914	BOLL	Boule Hon	Protein Co	36	GC02M197	1.23
6915	LDHAL6A	Lactate De	Protein Co	35	GC11P018	1.23
6916	PARPBP	PARP1 Bin	Protein Co	32	GC12P102	1.23
6917	CASQ2	Calsequest	Protein Co	44	GC01M115	1.23
6918	CD300C	CD300c M	Protein Co	35	GC17M074	1.23
6919	VPS13C	Vacuolar P	Protein Co	35	GC15M061	1.23
6920	TP73-AS1	TP73 Antis	RNA Gene	22	GC01M003	1.23
6921	LAMA5-AS	LAMA5 An	RNA Gene	13	GC20P062	1.23

6922	SKIL	SKI Like Pr	Protein Co	44	GC03P170	1.23
6923	PLXNB1	Plexin B1	Protein Co	44	GC03M048	1.23
6924	KRT6A	Keratin 6A	Protein Co	44	GC12M052	1.23
6925	USP18	Ubiquitin S	Protein Co	40	GC22P018	1.23
6926	SNPH	Syntaphilir	Protein Co	36	GC20P001	1.23
6927	EXOSC6	Exosome C	Protein Co	35	GC16M070	1.22
6928	INTS3	Integrator	Protein Co	37	GC01P153	1.22
6929	DFFB	DNA Fragr	Protein Co	43	GC01P003	1.22
6930	LOC10961(	Aristaless I	Biological	1	GC0XP025	1.22
6931	MAPK8IP3	Mitogen-A	Protein Co	40	GC16P001	1.22
6932	COLEC11	Collectin S	Protein Co	43	GC02P003	1.22
6933	MIR181B2	MicroRNA	RNA Gene	19	GC09P124	1.22
6934	SNCAIP	Synuclein /	Protein Co	44	GC05P122	1.22
6935	LILRA2	Leukocyte	Protein Co	39	GC19P054	1.22
6936	GPHA2	Glycoprote	Protein Co	35	GC11M065	1.22
6937	HTRA4	HtrA Serin	Protein Co	34	GC08P038	1.22
6938	POU6F2	POU Class	Protein Co	37	GC07P038	1.22
6939	CKAP2	Cytoskelet	Protein Co	35	GC13P052	1.22
6940	NSA2	NSA2 Ribo	Protein Co	35	GC05P074	1.22
6941	S100A7	S100 Calci	Protein Co	40	GC01M153	1.22
6942	CLEC1B	C-Type Le	Protein Co	40	GC12M013	1.22
6943	PACERR	PTGS2 Ant	RNA Gene	12	GC01P186	1.22
6944	ENSG00000266919		RNA Gene	5	GC17M030	1.22
6945	SNHG4	Small Nucl	RNA Gene	16	GC05P139	1.22
6946	FFAR1	Free Fatty	Protein Co	40	GC19P038	1.22
6947	GMEB1	Glucocorti	Protein Co	33	GC01P028	1.22
6948	ENSG00000267808		RNA Gene	8	GC19P051	1.22
6949	LOC10798	Uncharacte	RNA Gene	3	GC14M068	1.22
6950	CD200	CD200 Mo	Protein Co	39	GC03P112	1.22
6951	SYN1	Synapsin I	Protein Co	44	GC0XM047	1.22
6952	NMBR	Neuromed	Protein Co	42	GC06M142	1.22
6953	APOL2	Apolipopr	Protein Co	39	GC22M036	1.22
6954	USP13	Ubiquitin S	Protein Co	44	GC03P179	1.22
6955	RASGRF1	Ras Protei	Protein Co	41	GC15M078	1.22
6956	PRAME	PRAME Nu	Protein Co	37	GC22M022	1.21
6957	SMR3B	Submaxilla	Protein Co	31	GC04P070	1.21
6958	HSD17B13	Hydroxyste	Protein Co	36	GC04M087	1.21
6959	GNA12	G Protein S	Protein Co	41	GC07M002	1.21
6960	PDE1A	Phosphodi	Protein Co	43	GC02M182	1.21
6961	RAB33B	RAB33B, M	Protein Co	41	GC04P139	1.21
6962	GBP7	Guanylate	Protein Co	36	GC01M089	1.21
6963	HOXC10	Homeobox	Protein Co	36	GC12P054	1.21
6964	SNORA3A	Small Nucl	RNA Gene	14	GC11P008	1.21

6965	MIR663A	MicroRNA	RNA Gene	15	GC20M026	1.21
6966	UQCRQ	Ubiquinol-	Protein Co	41	GC05P132	1.21
6967	CD93	CD93 Mole	Protein Co	40	GC20M023	1.21
6968	LTC4S	Leukotrien	Protein Co	42	GC05P179	1.21
6969	NAIP	NLR Family	Protein Co	40	GC05M070	1.21
6970	SLC25A11	Solute Car	Protein Co	43	GC17M004	1.21
6971	TSN	Translin	Protein Co	41	GC02P121	1.21
6972	PRPF19	Pre-MRNA	Protein Co	37	GC11M060	1.21
6973	MYMK	Myomaker	Protein Co	23	GC09M133	1.21
6974	MIR199B	MicroRNA	RNA Gene	19	GC09M128	1.21
6975	NIBAN1	Niban Apo	Protein Co	29	GC01M184	1.21
6976	NCAPH	Non-SMC	Protein Co	40	GC02P096	1.2
6977	GDF9	Growth Dif	Protein Co	39	GC05M132	1.2
6978	ZBTB18	Zinc Finge	Protein Co	39	GC01P244	1.2
6979	GGACT	Gamma-Gl	Protein Co	35	GC13M100	1.2
6980	G6PC2	Glucose-6-	Protein Co	38	GC02P168	1.2
6981	PIGC	Phosphatic	Protein Co	39	GC01M172	1.2
6982	RTN2	Reticulon 2	Protein Co	40	GC19M045	1.2
6983	PPFIA1	PTPRF Inte	Protein Co	36	GC11P070	1.2
6984	NTM	Neurotrimi	Protein Co	41	GC11P131	1.2
6985	NMRAL1	NmrA Like	Protein Co	36	GC16M004	1.2
6986	SYCN	Syncollin	Protein Co	32	GC19M039	1.2
6987	MCRS1	Microsphe	Protein Co	37	GC12M049	1.2
6988	HCLS1	Hematopo	Protein Co	43	GC03M121	1.2
6989	TANGO2	Transport	Protein Co	35	GC22P020	1.2
6990	TOX4	TOX High	Protein Co	35	GC14P021	1.2
6991	ZNF384	Zinc Finge	Protein Co	36	GC12M006	1.2
6992	XYLT2	Xylosyltran	Protein Co	45	GC17P050	1.2
6993	LILRA4	Leukocyte	Protein Co	38	GC19M054	1.2
6994	C1orf112	Chromoso	Protein Co	32	GC01P169	1.2
6995	MROH8	Maestro H	Protein Co	29	GC20M037	1.2
6996	LOC11046	CYP1A1 5'	Biological	1	GC15P074	1.2
6997	L13714-130		RNA Gene	4	GC14P068	1.2
6998	UCP3	Uncouplin	Protein Co	43	GC11M074	1.2
6999	C4BPB	Compleme	Protein Co	40	GC01P207	1.2
7000	MCCC2	Methylcrot	Protein Co	44	GC05P071	1.19
7001	WWC1	WW And C	Protein Co	42	GC05P168	1.19
7002	ERP29	Endoplasm	Protein Co	35	GC12P112	1.19
7003	MPIG6B	Megakaryo	Protein Co	32	GC06P047	1.19
7004	CST5	Cystatin D	Protein Co	39	GC20M023	1.19
7005	RFC3	Replicatior	Protein Co	38	GC13P033	1.19
7006	SEMA4C	Semaphori	Protein Co	38	GC02M096	1.19
7007	H2BC4	H2B Cluste	Protein Co	29	GC06M027	1.19

7008	ITIH1	Inter-Alph	Protein Co	39	GC03P052	1.19
7009	AIFM3	Apoptosis	Protein Co	37	GC22P020	1.19
7010	COQ4	Coenzyme	Protein Co	39	GC09P128	1.19
7011	KCNIP3	Potassium	Protein Co	39	GC02P095	1.19
7012	TAPT1	Transmem	Protein Co	36	GC04M016	1.19
7013	STYX	Serine/Thr	Protein Co	36	GC14P052	1.19
7014	DENND4B	DENN Dor	Protein Co	32	GC01M153	1.19
7015	H1-1	H1.1 Linke	Protein Co	31	GC06M026	1.19
7016	MYSM1	Myb Like,	Protein Co	39	GC01M058	1.19
7017	GNB4	G Protein	Protein Co	43	GC03M179	1.19
7018	CCDC177	Coiled-Coi	Protein Co	24	GC14M069	1.19
7019	NAA35	N-Alpha-A	Protein Co	33	GC09P085	1.19
7020	ERG	ETS Transc	Protein Co	45	GC21M038	1.18
7021	SGCA	Sarcoglyca	Protein Co	41	GC17P050	1.18
7022	ETNK2	Ethanolam	Protein Co	40	GC01M204	1.18
7023	KRT2	Keratin 2	Protein Co	40	GC12M052	1.18
7024	PLAAT4	Phospholip	Protein Co	29	GC11P063	1.18
7025	RTRAF	RNA Trans	Protein Co	28	GC14P051	1.18
7026	SCN4B	Sodium Vc	Protein Co	43	GC11M118	1.18
7027	DDX10	DEAD-Box	Protein Co	39	GC11P108	1.18
7028	PDE7B	Phosphodi	Protein Co	39	GC06P135	1.18
7029	NAT14	N-Acetyltr	Protein Co	34	GC19P055	1.18
7030	DYDC2	DPY30 Dor	Protein Co	33	GC10P080	1.18
7031	CCDC122	Coiled-Coi	Protein Co	31	GC13M043	1.18
7032	CARNMT1	Carnosine	Protein Co	29	GC09M074	1.18
7033	RAB38	RAB38, Me	Protein Co	40	GC11M087	1.18
7034	MIR184	MicroRNA	RNA Gene	23	GC15P079	1.18
7035	CYP2U1	Cytochrom	Protein Co	44	GC04P107	1.18
7036	HTR1F	5-Hydroxy	Protein Co	44	GC03P087	1.18
7037	DEFA4	Defensin A	Protein Co	36	GC08M006	1.18
7038	NARS2	Asparagin	Protein Co	43	GC11M078	1.18
7039	FAIM2	Fas Apopt	Protein Co	36	GC12M049	1.18
7040	ITGB3BP	Integrin Su	Protein Co	41	GC01M063	1.18
7041	SIK3	SIK Family	Protein Co	41	GC11M116	1.18
7042	RAB11FIP5	RAB11 Fan	Protein Co	39	GC02M073	1.17
7043	BBS5	Bardet-Bie	Protein Co	39	GC02P169	1.17
7044	PRC1	Protein Re	Protein Co	39	GC15M090	1.17
7045	NOP58	NOP58 Rib	Protein Co	38	GC02P202	1.17
7046	KMT5A	Lysine Met	Protein Co	37	GC12P123	1.17
7047	SGCD	Sarcoglyca	Protein Co	45	GC05P155	1.17
7048	ADAM22	ADAM Me	Protein Co	41	GC07P087	1.17
7049	PUS7	Pseudouric	Protein Co	40	GC07M105	1.17
7050	CXXC4	CXXC Fing	Protein Co	39	GC04M104	1.17

7051	STRADB	STE20 Rela	Protein Co	39	GC02P201	1.17
7052	TIMM8B	Translocas	Protein Co	37	GC11M112	1.17
7053	DOLPP1	Dolichyldip	Protein Co	36	GC09P129	1.17
7054	SEC14L5	SEC14 Like	Protein Co	31	GC16P004	1.17
7055	JCAD	Junctional	Protein Co	26	GC10M030	1.17
7056	MIR6869	MicroRNA	RNA Gene	10	GC20M001	1.17
7057	CSGALNAC	Chondroiti	Protein Co	40	GC08M019	1.17
7058	SERPINB4	Serpin Fan	Protein Co	38	GC18M063	1.17
7059	KCNA4	Potassium	Protein Co	43	GC11M030	1.17
7060	RPL35	Ribosomal	Protein Co	44	GC09M124	1.17
7061	KIF14	Kinesin Fa	Protein Co	39	GC01M200	1.17
7062	UQCRC2	Ubiquinol-	Protein Co	46	GC16P021	1.17
7063	HPN	Hepsin	Protein Co	43	GC19P035	1.17
7064	SGMS1	Sphingomy	Protein Co	40	GC10M050	1.17
7065	MYL7	Myosin Lig	Protein Co	38	GC07M044	1.17
7066	CHRNA9	Cholinergi	Protein Co	42	GC04P040	1.17
7067	USP15	Ubiquitin S	Protein Co	47	GC12P062	1.17
7068	SMG1	SMG1 Nor	Protein Co	41	GC16M018	1.17
7069	OPN4	Opsin 4	Protein Co	40	GC10P086	1.17
7070	TPR	Translocat	Protein Co	42	GC01M186	1.17
7071	FGD2	FYVE, Rho	Protein Co	36	GC06P047	1.16
7072	CIT	Citron Rho	Protein Co	45	GC12M119	1.16
7073	LIPT1	Lipoyltrans	Protein Co	41	GC02P099	1.16
7074	NUP205	Nucleopor	Protein Co	37	GC07P135	1.16
7075	APBB2	Amyloid B	Protein Co	39	GC04M040	1.16
7076	GRIN3A	Glutamate	Protein Co	37	GC09M101	1.16
7077	MEDAG	Mesenteric	Protein Co	28	GC13P030	1.16
7078	RPS15A	Ribosomal	Protein Co	41	GC16M018	1.16
7079	ZFAT	Zinc Finge	Protein Co	36	GC08M134	1.16
7080	H2BC7	H2B Cluste	Protein Co	27	GC06P028	1.16
7081	PAX1	Paired Box	Protein Co	43	GC20P021	1.16
7082	PIGH	Phosphatic	Protein Co	40	GC14M067	1.16
7083	WDR72	WD Repea	Protein Co	37	GC15M061	1.16
7084	FAM160B1	Family Wit	Protein Co	35	GC10P114	1.16
7085	CCDC127	Coiled-Coi	Protein Co	33	GC05M000	1.16
7086	RNF103	Ring Finge	Protein Co	33	GC02M086	1.16
7087	MROH7	Maestro H	Protein Co	31	GC01P054	1.16
7088	NBPF12	NBPF Mem	Protein Co	28	GC01P146	1.16
7089	KCNT2	Potassium	Protein Co	39	GC01M196	1.15
7090	RBFOX1	RNA Bindi	Protein Co	37	GC16P005	1.15
7091	TRPV3	Transient F	Protein Co	43	GC17M003	1.15
7092	COCH	Cochlin	Protein Co	42	GC14P030	1.15
7093	HNF4A-AS	HNF4A An	RNA Gene	13	GC20M044	1.15

7094	HCAR1	Hydroxyca	Protein Co	37	GC12M122	1.15
7095	MCRIP2	MAPK Reg	Protein Co	25	GC16P000	1.15
7096	LY86-AS1	LY86 Antis	RNA Gene	17	GC06M006	1.15
7097	GNAL	G Protein !	Protein Co	45	GC18P011	1.15
7098	GPR143	G Protein-(	Protein Co	41	GC0XM009	1.15
7099	PLXNB3	Plexin B3	Protein Co	39	GC0XP153	1.15
7100	PNCK	Pregnancy	Protein Co	37	GC0XM153	1.15
7101	GAR1	GAR1 Ribc	Protein Co	36	GC04P109	1.15
7102	STOX2	Storkhead	Protein Co	35	GC04P183	1.15
7103	NBPF10	NBPF Mer	Protein Co	27	GC01M146	1.15
7104	CD200R1	CD200 Rec	Protein Co	40	GC03M112	1.15
7105	SLURP1	Secreted L	Protein Co	41	GC08M142	1.14
7106	SEMA3B	Semaphori	Protein Co	39	GC03P050	1.14
7107	KIF12	Kinesin Fa	Protein Co	35	GC09M114	1.14
7108	BCORL1	BCL6 Core	Protein Co	35	GC0XP129	1.14
7109	UBE2H	Ubiquitin (	Protein Co	44	GC07M129	1.14
7110	RAD17	RAD17 Ch	Protein Co	42	GC05P069	1.14
7111	RIMS1	Regulating	Protein Co	40	GC06P071	1.14
7112	CHMP3	Charged M	Protein Co	39	GC02M086	1.14
7113	AKIP1	A-Kinase In	Protein Co	33	GC11P008	1.14
7114	TRAIP	TRAF Inter	Protein Co	40	GC03M049	1.14
7115	CLUL1	Clusterin L	Protein Co	35	GC18P000	1.14
7116	DCSTAMP	Dendrocyt	Protein Co	35	GC08P104	1.14
7117	ZNF341	Zinc Finge	Protein Co	36	GC20P033	1.14
7118	GABRR2	Gamma-Ar	Protein Co	40	GC06M089	1.14
7119	THAP1	THAP Dom	Protein Co	39	GC08M042	1.14
7120	NR2E3	Nuclear Re	Protein Co	41	GC15P071	1.14
7121	ERVFRD-1	Endogenou	Protein Co	32	GC06M011	1.14
7122	BST2	Bone Marr	Protein Co	39	GC19M017	1.14
7123	CAD	Carbamoyl	Protein Co	50	GC02P027	1.14
7124	ENPP4	Ectonuclec	Protein Co	35	GC06P046	1.14
7125	SETD7	SET Domai	Protein Co	43	GC04M139	1.13
7126	CUL5	Cullin 5	Protein Co	44	GC11P108	1.13
7127	SCNM1	Sodium Ch	Protein Co	32	GC01P151	1.13
7128	MARCHF5	Membrane	Protein Co	31	GC10P092	1.13
7129	PELI3	Pellino E3	Protein Co	36	GC11P066	1.13
7130	SMIM19	Small Inte	Protein Co	31	GC08P042	1.13
7131	HLA-DQB1	HLA-DQB1	RNA Gene	12	GC06P032	1.13
7132	ENSG00000234427		RNA Gene	7	GC06P011	1.13
7133	PLET1	Placenta E	Protein Co	28	GC11M112	1.13
7134	RBBP7	RB Binding	Protein Co	40	GC0XM016	1.13
7135	ZNF750	Zinc Finge	Protein Co	36	GC17M082	1.13
7136	ADAMTS7	ADAM Me	Protein Co	40	GC15M078	1.12

7137	PPP2R2A	Protein Ph	Protein Co	44	GC08P026	1.12
7138	UBE2E1	Ubiquitin (	Protein Co	41	GC03P023	1.12
7139	CEBPG	CCAAT Enl	Protein Co	37	GC19P033	1.12
7140	GCSH	Glycine Cle	Protein Co	43	GC16M081	1.12
7141	CRIPT	CXXC Repe	Protein Co	40	GC02P046	1.12
7142	DCLRE1B	DNA Cross	Protein Co	35	GC01P113	1.12
7143	PJVK	Pejvakin	Protein Co	28	GC02P178	1.12
7144	NBPF9	NBPF Mem	Protein Co	25	GC01M149	1.12
7145	TCF4-AS1	TCF4 Antis	RNA Gene	11	GC18P055	1.12
7146	GPR182	G Protein-(	Protein Co	37	GC12P056	1.12
7147	SKOR1	SKI Family	Protein Co	33	GC15P067	1.12
7148	ACP3	Acid Phosp	Protein Co	35	GC03P132	1.12
7149	NT5C1A	5'-Nucleot	Protein Co	39	GC01M039	1.12
7150	SPPL2C	Signal Pep	Protein Co	32	GC17P045	1.12
7151	CCDC148	Coiled-Coi	Protein Co	35	GC02M158	1.12
7152	UBE2S	Ubiquitin (	Protein Co	41	GC19M055	1.12
7153	ATP1A4	ATPase Na	Protein Co	41	GC01P160	1.12
7154	EPB42	Erythrocyte	Protein Co	39	GC15M043	1.12
7155	SET	SET Nuclea	Protein Co	47	GC09P128	1.12
7156	MIR19B2	MicroRNA	RNA Gene	16	GC0XM134	1.12
7157	PPM1A	Protein Ph	Protein Co	45	GC14P060	1.12
7158	TRAK2	Trafficking	Protein Co	39	GC02M201	1.11
7159	PDE6B	Phosphodi	Protein Co	45	GC04P000	1.11
7160	TUBGCP4	Tubulin Ga	Protein Co	39	GC15P043	1.11
7161	ZGRF1	Zinc Finge	Protein Co	31	GC04M112	1.11
7162	RIPPLY1	Ripply Tra	Protein Co	29	GC0XM106	1.11
7163	LINC01080	Long Inter	RNA Gene	13	GC13P079	1.11
7164	LRP1-AS	LRP1 Antis	RNA Gene	11	GC12M057	1.11
7165	AGMAT	Agmatinas	Protein Co	39	GC01M015	1.11
7166	PPP1CC	Protein Ph	Protein Co	45	GC12M110	1.11
7167	FBXL19	F-Box And	Protein Co	36	GC16P030	1.11
7168	CTSV	Cathepsin	Protein Co	40	GC09M097	1.11
7169	CALU	Calumenin	Protein Co	40	GC07P128	1.1
7170	ENSG00000260482		RNA Gene	7	GC16P023	1.1
7171	KPNA4	Karyopheri	Protein Co	43	GC03M160	1.1
7172	CDH15	Cadherin 1	Protein Co	43	GC16P089	1.1
7173	SYTL4	Synaptotag	Protein Co	39	GC0XM100	1.1
7174	AQR	Aquarius I	Protein Co	36	GC15M034	1.1
7175	DACT3	Dishevelle	Protein Co	35	GC19M046	1.1
7176	TGDS	TDP-Gluco	Protein Co	37	GC13M094	1.1
7177	COQ3	Coenzyme	Protein Co	40	GC06M099	1.1
7178	JAGN1	Jagunal Ho	Protein Co	36	GC03P009	1.1
7179	ELP4	Elongator	Protein Co	39	GC11P031	1.1

7180	ALDH1L1	Aldehyde I	Protein Co	40	GC03M126	1.1
7181	GFRA3	GDNF Fam	Protein Co	40	GC05M138	1.1
7182	MIR124-3	MicroRNA	RNA Gene	17	GC20P063	1.1
7183	NKX6-2	NK6 Home	Protein Co	38	GC10M132	1.1
7184	RNF13	Ring Finge	Protein Co	41	GC03P149	1.1
7185	PRMT2	Protein Ar	Protein Co	41	GC21P046	1.1
7186	DCAF1	DDB1 And	Protein Co	31	GC03M051	1.1
7187	TREML1	Triggering	Protein Co	37	GC06M042	1.09
7188	SUV39H2	Suppresso	Protein Co	43	GC10P014	1.09
7189	RAB3A	RAB3A, M	Protein Co	44	GC19M018	1.09
7190	GPR3	G Protein-	Protein Co	38	GC01P027	1.09
7191	SYT6	Synaptota	Protein Co	36	GC01M114	1.09
7192	CDRT15	CMT1A Du	Protein Co	29	GC17M014	1.09
7193	MCPH1-AS	MCPH1 Ar	RNA Gene	11	GC08M006	1.09
7194	MS4A2	Membrane	Protein Co	41	GC11P060	1.09
7195	POMP	Proteasom	Protein Co	41	GC13P028	1.09
7196	PELATON	Plaque Enr	Protein Co	14	GC20P050	1.09
7197	PRKACG	Protein Kir	Protein Co	47	GC09M069	1.09
7198	C1QTNF1	C1q And T	Protein Co	39	GC17P079	1.09
7199	MIR181D	MicroRNA	RNA Gene	17	GC19P013	1.09
7200	PDCD6	Programm	Protein Co	39	GC05P000	1.09
7201	FGF6	Fibroblast	Protein Co	40	GC12M004	1.09
7202	NTN4	Netrin 4	Protein Co	39	GC12M095	1.09
7203	EVA1A	Eva-1 Hon	Protein Co	33	GC02M075	1.08
7204	SLC37A2	Solute Car	Protein Co	35	GC11P125	1.08
7205	CACNA2D3	Calcium V	Protein Co	40	GC03P054	1.08
7206	TSHZ3	Teashirt Zi	Protein Co	37	GC19M031	1.08
7207	VRK1	VRK Serine	Protein Co	48	GC14P096	1.08
7208	XPR1	Xenotropic	Protein Co	43	GC01P180	1.08
7209	CDK5R2	Cyclin Dep	Protein Co	39	GC02P218	1.08
7210	KIF20A	Kinesin Fa	Protein Co	39	GC05P138	1.08
7211	SORCS1	Sortilin Rel	Protein Co	38	GC10M106	1.08
7212	RPP40	Ribonuclea	Protein Co	35	GC06M004	1.08
7213	PDIA3P1	Protein Dis	Pseudoger	12	GC01P147	1.08
7214	PPP1R3B	Protein Ph	Protein Co	37	GC08M009	1.08
7215	KRT23	Keratin 23	Protein Co	37	GC17M040	1.08
7216	lnc-ADIPOQ-2		RNA Gene	5	GC03P186	1.08
7217	LAMTOR5	Late Endos	Protein Co	36	GC01M110	1.08
7218	OSTM1	Osteoclast	Protein Co	38	GC06M108	1.08
7219	PMM1	Phosphom	Protein Co	41	GC22M041	1.08
7220	TMEM233	Transmem	Protein Co	27	GC12P119	1.08
7221	NKILA	NF-KappaB	RNA Gene	11	GC20P057	1.08
7222	ASGR1	Asialoglyc	Protein Co	40	GC17M007	1.08

7223	MMP17	Matrix Me	Protein Co	44	GC12P131	1.08
7224	LINC00581	Long Inter	RNA Gene	10	GC06M021	1.08
7225	KIAA1549	KIAA1549	Protein Co	37	GC07M138	1.08
7226	C1orf54	Chromoso	Protein Co	28	GC01P150	1.08
7227	EPHA8	EPH Recep	Protein Co	45	GC01P022	1.07
7228	SIAH2	Siah E3 Ub	Protein Co	44	GC03M150	1.07
7229	AFAP1L2	Actin Filan	Protein Co	36	GC10M114	1.07
7230	HIC2	HIC ZBTB	Protein Co	35	GC22P024	1.07
7231	RPS6KA4	Ribosomal	Protein Co	47	GC11P064	1.07
7232	HFM1	Helicase F	Protein Co	39	GC01M091	1.07
7233	LDB2	LIM Doma	Protein Co	38	GC04M016	1.07
7234	ARHGEF15	Rho Guani	Protein Co	37	GC17P008	1.07
7235	HRC	Histidine R	Protein Co	36	GC19M049	1.07
7236	DYDC1	DPY30 Do	Protein Co	35	GC10M080	1.07
7237	SLC35E3	Solute Car	Protein Co	35	GC12P068	1.07
7238	PPAN-P2R	PPAN-P2R	Protein Co	24	GC19P010	1.07
7239	LINC01090	Long Inter	RNA Gene	13	GC02M187	1.07
7240	RTN4RL1	Reticulon 4	Protein Co	37	GC17M001	1.07
7241	SLC12A4	Solute Car	Protein Co	43	GC16M067	1.07
7242	PCLO	Piccolo Pre	Protein Co	41	GC07M082	1.07
7243	NEUROG2	Neurogeni	Protein Co	39	GC04M112	1.07
7244	H2BC8	H2B Cluste	Protein Co	27	GC06M027	1.07
7245	CAPNS2	Calpain Sn	Protein Co	36	GC16P055	1.07
7246	NR1D2	Nuclear Re	Protein Co	42	GC03P023	1.07
7247	FCN1	Ficolin 1	Protein Co	39	GC09M134	1.07
7248	ZBTB7C	Zinc Finge	Protein Co	33	GC18M048	1.07
7249	NSDHL	NAD(P) De	Protein Co	44	GC0XP152	1.07
7250	GPRC6A	G Protein-(	Protein Co	39	GC06M116	1.07
7251	ATP8A1	ATPase Ph	Protein Co	42	GC04M042	1.07
7252	ASTN2	Astrotactin	Protein Co	36	GC09M116	1.07
7253	HIF1AN	Hypoxia In	Protein Co	44	GC10P100	1.07
7254	BSND	Barttin CL(	Protein Co	40	GC01P054	1.07
7255	DNAJC15	DnaJ Heat	Protein Co	37	GC13P043	1.06
7256	ITGAD	Integrin Su	Protein Co	36	GC16P031	1.06
7257	AFG3L2	AFG3 Like	Protein Co	44	GC18M012	1.06
7258	PRKAG3	Protein Kir	Protein Co	43	GC02M218	1.06
7259	NR2E1	Nuclear Re	Protein Co	43	GC06P108	1.06
7260	ACOT11	Acyl-CoA T	Protein Co	39	GC01P054	1.06
7261	DOCK4	Dedicator	Protein Co	39	GC07M111	1.06
7262	MRPS9	Mitochond	Protein Co	38	GC02P105	1.06
7263	GCM1	Glial Cells	Protein Co	37	GC06M053	1.06
7264	HSPA12A	Heat Shoc	Protein Co	37	GC10M116	1.06
7265	TIMM10B	Translocas	Protein Co	35	GC11P006	1.06

7266	GSX2	GS Homec	Protein Co	32	GC04P054	1.06
7267	HIKESHI	Heat Shoc	Protein Co	32	GC11P086	1.06
7268	OSGIN2	Oxidative S	Protein Co	31	GC08P089	1.06
7269	FAM170B	Family Wit	Protein Co	27	GC10M049	1.06
7270	SLCO1C1	Solute Car	Protein Co	40	GC12P020	1.06
7271	TBCB	Tubulin Fo	Protein Co	39	GC19P038	1.06
7272	WWC2-AS	WWC2 An	RNA Gene	11	GC04M183	1.06
7273	EIF3E	Eukaryotic	Protein Co	38	GC08M108	1.06
7274	PCDH17	Protocadh	Protein Co	36	GC13P057	1.06
7275	BAG4	BAG Coch	Protein Co	39	GC08P038	1.06
7276	CCNDBP1	Cyclin D1 I	Protein Co	36	GC15P043	1.06
7277	MAL	Mal, T Cell	Protein Co	37	GC02P095	1.06
7278	MIR194-1	MicroRNA	RNA Gene	16	GC01M220	1.05
7279	WHRN	Whirlin	Protein Co	32	GC09M114	1.05
7280	EMILIN3	Elastin Mic	Protein Co	31	GC20M041	1.05
7281	ORC4	Origin Rec	Protein Co	41	GC02M147	1.05
7282	EN2	Engrailed I	Protein Co	40	GC07P155	1.05
7283	SNAP91	Synaptoso	Protein Co	40	GC06M083	1.05
7284	MFSD8	Major Faci	Protein Co	37	GC04M127	1.05
7285	KLHL14	Kelch Like	Protein Co	36	GC18M032	1.05
7286	GPKOW	G-Patch D	Protein Co	35	GC0XM049	1.05
7287	DET1	DET1 Partr	Protein Co	34	GC15M088	1.05
7288	AGMO	Alkylglycer	Protein Co	33	GC07M015	1.05
7289	CMC2	C-X9-C Mo	Protein Co	33	GC16M080	1.05
7290	SVOPL	SVOP Like	Protein Co	32	GC07M138	1.05
7291	PLPP7	Phospholip	Protein Co	28	GC09P131	1.05
7292	ASAH2B	N-Acylsphi	Protein Co	27	GC10P050	1.05
7293	MARCHF1	Membrane	Protein Co	23	GC05M016	1.05
7294	HNRNPUL	HNRNPUL	RNA Gene	15	GC11M063	1.05
7295	RAMP2	Receptor A	Protein Co	41	GC17P042	1.05
7296	PSMD10	Proteasom	Protein Co	40	GC0XM108	1.05
7297	GZF1	GDNF Indu	Protein Co	36	GC20P023	1.05
7298	LGALS13	Galectin 13	Protein Co	39	GC19P039	1.05
7299	GPR152	G Protein-(	Protein Co	31	GC11M067	1.05
7300	SPINK13	Serine Pep	Protein Co	31	GC05P148	1.05
7301	FMR1-AS1	FMR1 Anti	RNA Gene	16	GC0XM147	1.05
7302	GRIK3	Glutamate	Protein Co	43	GC01M036	1.05
7303	FOXK1	Forkhead I	Protein Co	35	GC07P004	1.05
7304	NTNG1	Netrin G1	Protein Co	41	GC01P107	1.05
7305	FOXO6	Forkhead I	Protein Co	33	GC01P041	1.05
7306	MSBP1	Minisatellit	Protein Co	4	GC00U990	1.05
7307	IKZF4	IKAROS Fa	Protein Co	36	GC12P056	1.05
7308	SNRK	SNF Relate	Protein Co	39	GC03P043	1.05

7309	IL20	Interleukin	Protein Co	39	GC01P206	1.05
7310	ANKRD1	Ankyrin Re	Protein Co	42	GC10M090	1.05
7311	FGF22	Fibroblast	Protein Co	38	GC19P000	1.05
7312	HECTD2	HECT Dom	Protein Co	35	GC10P091	1.04
7313	ADM2	Adrenome	Protein Co	33	GC22P050	1.04
7314	MLF2	Myeloid Le	Protein Co	35	GC12M006	1.04
7315	HPCA	Hippocalci	Protein Co	41	GC01P032	1.04
7316	NOVA2	NOVA Alte	Protein Co	36	GC19M045	1.04
7317	FKBP15	FKBP Proly	Protein Co	34	GC09M113	1.04
7318	H3C3	H3 Cluster	Protein Co	31	GC06P028	1.04
7319	ST3GAL6-A	ST3GAL6 A	RNA Gene	15	GC03M098	1.04
7320	SLC25A12	Solute Car	Protein Co	46	GC02M171	1.04
7321	DGKK	Diacylglyce	Protein Co	37	GC0XM050	1.04
7322	SSH2	Slingshot F	Protein Co	39	GC17M029	1.04
7323	IGKV2D-29	Immunogl	Protein Co	13	GC02P090	1.04
7324	AHRR	Aryl-Hydrc	Protein Co	35	GC05P000	1.04
7325	GNPAT	Glyceronep	Protein Co	45	GC01P231	1.04
7326	USP11	Ubiquitin S	Protein Co	40	GC0XP047	1.04
7327	SMPDL3B	Sphingomy	Protein Co	35	GC01P027	1.04
7328	TMBIM4	Transmem	Protein Co	33	GC12M066	1.04
7329	RNF125	Ring Finge	Protein Co	40	GC18P032	1.04
7330	IL17RD	Interleukin	Protein Co	42	GC03M057	1.04
7331	KLK2	Kallikrein F	Protein Co	43	GC19P050	1.03
7332	FCRL6	Fc Recepto	Protein Co	34	GC01P159	1.03
7333	RHPN1	Rhophilin I	Protein Co	37	GC08P143	1.03
7334	CASP14	Caspase 14	Protein Co	44	GC19P015	1.03
7335	NAA30	N-Alpha-A	Protein Co	37	GC14P057	1.03
7336	SLC38A8	Solute Car	Protein Co	36	GC16M084	1.03
7337	TMCO3	Transmem	Protein Co	35	GC13P113	1.03
7338	EPOP	Elongin BC	Protein Co	22	GC17M038	1.03
7339	MIR659	MicroRNA	RNA Gene	17	GC22M045	1.03
7340	TCAP	Titin-Cap	Protein Co	42	GC17P039	1.03
7341	GCKR	Glucokinas	Protein Co	40	GC02P027	1.03
7342	PRM2	Protamine	Protein Co	34	GC16M011	1.03
7343	CHRNB3	Cholinergi	Protein Co	41	GC08P042	1.03
7344	C19orf81	Chromoso	Protein Co	25	GC19P050	1.03
7345	ENSG00000274204		RNA Gene	5	GC13M106	1.03
7346	RF00017-7014		RNA Gene	3	GC08P042	1.03
7347	ENSG00000227766		Pseudoger	3	GC06M030	1.03
7348	ENSG00000233565		Pseudoger	3	GC03M186	1.03
7349	LOC10798	Uncharacte	RNA Gene	3	GC06M011	1.03
7350	BTG3	BTG Anti-F	Protein Co	37	GC21M017	1.02
7351	TRPM3	Transient F	Protein Co	41	GC09M070	1.02

7352	TSPAN33	Tetraspanii	Protein Co	36	GC07P129	1.02
7353	SASH1	SAM And !	Protein Co	39	GC06P148	1.02
7354	ZIC5	Zic Family	Protein Co	35	GC13M099	1.02
7355	KDF1	Keratinocy	Protein Co	33	GC01M026	1.02
7356	TUBA3D	Tubulin Al	Protein Co	37	GC02P132	1.02
7357	OXER1	Oxoeicosai	Protein Co	39	GC02M042	1.02
7358	OPHN1	Oligophrer	Protein Co	42	GC0XM068	1.02
7359	MTMR1	Myotubula	Protein Co	39	GC0XP150	1.02
7360	DLGAP2	DLG Assoc	Protein Co	37	GC08P000	1.02
7361	ZP2	Zona Pellu	Protein Co	37	GC16M021	1.02
7362	NOBOX	NOBOX O(	Protein Co	35	GC07M144	1.02
7363	ZNF451	Zinc Finge	Protein Co	35	GC06P057	1.02
7364	GDAP1L1	Gangliosid	Protein Co	34	GC20P044	1.02
7365	H3C1	H3 Cluster	Protein Co	32	GC06P026	1.01
7366	LARP6	La Ribonu(	Protein Co	33	GC15M070	1.01
7367	ATP1B2	ATPase Na	Protein Co	41	GC17P008	1.01
7368	CDH16	Cadherin 1	Protein Co	40	GC16M066	1.01
7369	KCTD1	Potassium	Protein Co	40	GC18M026	1.01
7370	RNF216	Ring Finge	Protein Co	42	GC07M005	1.01
7371	STAB1	Stabilin 1	Protein Co	39	GC03P052	1.01
7372	CHST3	Carbohydr	Protein Co	42	GC10P071	1.01
7373	BNC1	Basonuclin	Protein Co	36	GC15M083	1.01
7374	VPS4A	Vacuolar P	Protein Co	41	GC16P069	1.01
7375	ACTL7B	Actin Like	Protein Co	34	GC09M108	1.01
7376	GNRHR	Gonadotro	Protein Co	47	GC04M067	1.01
7377	MAFK	MAF BZIP	Protein Co	37	GC07P001	1.01
7378	KCNE2	Potassium	Protein Co	41	GC21P034	1.01
7379	ACADSB	Acyl-CoA [	Protein Co	46	GC10P123	1.01
7380	CACNG1	Calcium V(	Protein Co	41	GC17P067	1.01
7381	GPM6A	Glycoprote	Protein Co	40	GC04M175	1.01
7382	YOD1	YOD1 Deu	Protein Co	37	GC01M207	1.01
7383	CABP7	Calcium Bi	Protein Co	35	GC22P029	1.01
7384	NBPF15	NBPF Mem	Protein Co	30	GC01M144	1.01
7385	NGF-AS1	NGF Antis(	RNA Gene	9	GC01P115	1.01
7386	DPP8	Dipeptidyl	Protein Co	37	GC15M065	1.01
7387	MTNR1A	Melatonin	Protein Co	44	GC04M186	1.01
7388	IGFLR1	IGF Like Fa	Protein Co	33	GC19M042	1
7389	PEG3	Paternally	Protein Co	38	GC19M056	1
7390	PCTP	Phosphatic	Protein Co	40	GC17P055	1
7391	ATP11C	ATPase Ph	Protein Co	36	GC0XM139	1
7392	STIP1	Stress Indu	Protein Co	43	GC11P064	1
7393	ELF2	E74 Like E	Protein Co	38	GC04M139	1
7394	MAP1A	Microtubul	Protein Co	40	GC15P043	1

7395	UBE2G2	Ubiquitin (	Protein Co	44	GC21M044	1
7396	PYDC1	Pyrin Dom	Protein Co	33	GC16M031	1
7397	PLPP6	Phospholip	Protein Co	27	GC09P004	1
7398	MIR206	MicroRNA	RNA Gene	20	GC06P052	1
7399	FAHD1	Fumarylace	Protein Co	37	GC16P001	1
7400	MIR1256	MicroRNA	RNA Gene	12	GC01M020	1
7401	GPRC5B	G Protein-(	Protein Co	38	GC16M019	1
7402	NENF	Neudesin I	Protein Co	39	GC01P212	1
7403	PTGFR	Prostaglan	Protein Co	44	GC01P078	1
7404	CYB561	Cytochrom	Protein Co	41	GC17M063	1
7405	MTMR14	Myotubula	Protein Co	43	GC03P009	0.99
7406	UBE2G1	Ubiquitin (	Protein Co	43	GC17M004	0.99
7407	LRRTM1	Leucine Ri	Protein Co	39	GC02M080	0.99
7408	TRIM9	Tripartite M	Protein Co	39	GC14M050	0.99
7409	ANKRD6	Ankyrin Re	Protein Co	37	GC06P089	0.99
7410	METTL5	Methyltran	Protein Co	36	GC02M169	0.99
7411	CLEC6A	C-Type Le(	Protein Co	36	GC12P008	0.99
7412	TDRD3	Tudor Don	Protein Co	36	GC13P060	0.99
7413	MRPS26	Mitochond	Protein Co	35	GC20P003	0.99
7414	NMD3	NMD3 Rib	Protein Co	35	GC03P161	0.99
7415	PLAC1	Placenta E	Protein Co	35	GC0XM134	0.99
7416	RNF17	Ring Finge	Protein Co	33	GC13P024	0.99
7417	CGGBP1	CGG Triple	Protein Co	32	GC03M088	0.99
7418	ZNF385D	Zinc Finge	Protein Co	32	GC03M021	0.99
7419	MIR3193	MicroRNA	RNA Gene	13	GC20P031	0.99
7420	LRP1B	LDL Recep	Protein Co	39	GC02M140	0.99
7421	COP1	COP1 E3 L	Protein Co	33	GC01M175	0.99
7422	SYTL1	Synaptotag	Protein Co	36	GC01P027	0.99
7423	TMED7	Transmem	Protein Co	32	GC05M115	0.99
7424	KCNJ8	Potassium	Protein Co	43	GC12M021	0.99
7425	HERPUD1	Homocyste	Protein Co	39	GC16P056	0.99
7426	TMEM150E	Transmem	Protein Co	28	GC19M055	0.99
7427	LINC01266	Long Inter(	RNA Gene	12	GC03P000	0.99
7428	PTPMT1	Protein Ty	Protein Co	37	GC11P047	0.99
7429	SORCS2	Sortilin Rel	Protein Co	36	GC04P007	0.99
7430	CYP4B1	Cytochrom	Protein Co	43	GC01P046	0.99
7431	CALHM1	Calcium H(	Protein Co	33	GC10M103	0.99
7432	ENSG00000228655		RNA Gene	8	GC02M143	0.98
7433	MIR374A	MicroRNA	RNA Gene	16	GC0XM074	0.98
7434	RLBP1	Retinaldeh	Protein Co	44	GC15M089	0.98
7435	CENPJ	Centromer	Protein Co	42	GC13M024	0.98
7436	TAF13	TATA-Box	Protein Co	41	GC01M109	0.98
7437	DNAJC12	DnaJ Heat	Protein Co	39	GC10M067	0.98

7438	LOXHD1	Lipoxygen	Protein Co	36	GC18M046	0.98
7439	EXOC3L2	Exocyst Co	Protein Co	33	GC19M045	0.98
7440	CSRNP3	Cysteine A	Protein Co	32	GC02P165	0.98
7441	H2AC7	H2A Cluste	Protein Co	28	GC06M027	0.98
7442	KRTAP9-9	Keratin As	Protein Co	27	GC17P041	0.98
7443	NDUFV2-A	NDUFV2 A	RNA Gene	13	GC18M009	0.98
7444	F10-AS1	F10 Antise	RNA Gene	12	GC13M113	0.98
7445	MICU2	Mitochond	Protein Co	36	GC13M021	0.98
7446	TTC8	Tetratricop	Protein Co	40	GC14P089	0.98
7447	AMOTL1	Angiomoti	Protein Co	37	GC11P094	0.98
7448	HSPA12B	Heat Shocl	Protein Co	35	GC20P003	0.98
7449	EXOC6	Exocyst Co	Protein Co	39	GC10P092	0.98
7450	C19orf53	Chromoso	Protein Co	31	GC19P013	0.98
7451	KRT6B	Keratin 6B	Protein Co	41	GC12M052	0.98
7452	PIK3C2G	Phosphatic	Protein Co	44	GC12P018	0.97
7453	APLP1	Amyloid B	Protein Co	40	GC19P038	0.97
7454	RXFP1	Relaxin Fa	Protein Co	44	GC04P158	0.97
7455	KIRREL1	Kirre Like I	Protein Co	29	GC01P157	0.97
7456	FGF13	Fibroblast	Protein Co	41	GC0XM138	0.97
7457	PGAM2	Phosphogl	Protein Co	44	GC07M044	0.97
7458	TOR1AIP1	Torsin 1A I	Protein Co	39	GC01P179	0.97
7459	RIPPLY2	Ripply Tra	Protein Co	37	GC06P083	0.97
7460	TOMM20L	Translocas	Protein Co	30	GC14P058	0.97
7461	PCDH9	Protocadh	Protein Co	37	GC13M066	0.97
7462	lnc-ADIPOQ-3		RNA Gene	4	GC03P186	0.97
7463	MH708019		RNA Gene	3	GC11M123	0.97
7464	PRB1	Proline Ric	Protein Co	34	GC12M013	0.97
7465	USPL1	Ubiquitin S	Protein Co	32	GC13P030	0.97
7466	ZC3H14	Zinc Finge	Protein Co	40	GC14P088	0.96
7467	SUGP1	SURP And	Protein Co	35	GC19M019	0.96
7468	FAM168B	Family Wit	Protein Co	32	GC02M131	0.96
7469	EPHA7	EPH Recep	Protein Co	48	GC06M093	0.96
7470	RPL36A	Ribosomal	Protein Co	35	GC0XP101	0.96
7471	TFDP3	Transcripti	Protein Co	31	GC0XM133	0.96
7472	ROR1	Receptor T	Protein Co	47	GC01P063	0.96
7473	SPIDR	Scaffold Pr	Protein Co	32	GC08P047	0.96
7474	CBFA2T3	CBFA2/RUI	Protein Co	40	GC16M088	0.96
7475	MPP2	Membrane	Protein Co	37	GC17M043	0.96
7476	PCGF1	Polycomb	Protein Co	37	GC02M074	0.96
7477	ACAD8	Acyl-CoA I	Protein Co	45	GC11P134	0.96
7478	KIF4A	Kinesin Fa	Protein Co	40	GC0XP070	0.96
7479	ANKS1B	Ankyrin Re	Protein Co	39	GC12M098	0.96
7480	COL25A1	Collagen T	Protein Co	39	GC04M108	0.96

7481	SNX14	Sorting Ne	Protein Co	39	GC06M085	0.96
7482	ARC	Activity Re	Protein Co	38	GC08M142	0.96
7483	UBXN2B	UBX Doma	Protein Co	37	GC08P058	0.96
7484	TIMM9	Translocas	Protein Co	37	GC14M058	0.96
7485	CSNK1A1L	Casein Kin	Protein Co	36	GC13M037	0.96
7486	CNTNAP5	Contactin /	Protein Co	35	GC02P124	0.96
7487	ARRDC1	Arrestin Dc	Protein Co	33	GC09P137	0.96
7488	MIR561	MicroRNA	RNA Gene	16	GC02P188	0.96
7489	CDR1-AS	CDR1 Anti	RNA Gene	6	GC0XU902	0.95
7490	PDAP1	PDGFA Ass	Protein Co	35	GC07M099	0.95
7491	ABCB10	ATP Bindir	Protein Co	40	GC01M229	0.95
7492	FKBP3	FKBP Proly	Protein Co	40	GC14M045	0.95
7493	MUC21	Mucin 21,	Protein Co	32	GC06P047	0.95
7494	SNN	Stannin	Protein Co	33	GC16P011	0.95
7495	NADK2	NAD Kinas	Protein Co	38	GC05M036	0.95
7496	MARCO	Macropha	Protein Co	40	GC02P118	0.95
7497	CCSER1	Coiled-Coi	Protein Co	30	GC04P090	0.95
7498	APOO	Apolipopro	Protein Co	34	GC0XM023	0.94
7499	FSHR	Follicle Sti	Protein Co	49	GC02M048	0.94
7500	EARS2	Glutamyl-T	Protein Co	42	GC16M023	0.94
7501	EXOSC8	Exosome C	Protein Co	40	GC13P036	0.94
7502	DLX1	Distal-Less	Protein Co	39	GC02P172	0.94
7503	BAALC	BAALC Bin	Protein Co	36	GC08P103	0.94
7504	SORCS3	Sortilin Rel	Protein Co	36	GC10P104	0.94
7505	H3C2	H3 Cluster	Protein Co	32	GC06M026	0.94
7506	SPIN2B	Spindlin Fa	Protein Co	32	GC0XM057	0.94
7507	H2AC4	H2A Cluste	Protein Co	29	GC06M026	0.94
7508	KRTAP4-11	Keratin Ass	Protein Co	27	GC17M041	0.94
7509	HAR1A	Highly Acc	RNA Gene	20	GC20P063	0.94
7510	PICSAR	P38 Inhibit	RNA Gene	16	GC21M047	0.94
7511	RAB26	RAB26, Me	Protein Co	38	GC16P002	0.94
7512	PTPN5	Protein Ty	Protein Co	43	GC11M018	0.94
7513	SLC25A17	Solute Car	Protein Co	39	GC22M045	0.94
7514	SLFN14	Schlafen Fa	Protein Co	33	GC17M035	0.94
7515	SYT3	Synaptotag	Protein Co	37	GC19M050	0.94
7516	SNORD14(	Small Nucl	RNA Gene	15	GC11M123	0.94
7517	SNORD14[	Small Nucl	RNA Gene	14	GC11M123	0.94
7518	SNORD14E	Small Nucl	RNA Gene	12	GC11M123	0.94
7519	lnc-CCDC89-4		RNA Gene	5	GC11M085	0.94
7520	lnc-CLMP-3		RNA Gene	4	GC11M123	0.94
7521	piR-59038		RNA Gene	3	GC11M123	0.94
7522	piR-35858-006		RNA Gene	3	GC06M011	0.94
7523	piR-61486		RNA Gene	3	GC11M123	0.94

7524	lnc-SMIM14-5		RNA Gene	2	GC04M039	0.94
7525	LOC10192	Uncharacte	RNA Gene	2	GC11P123	0.94
7526	CD5L	CD5 Molec	Protein Co	39	GC01M157	0.94
7527	NCMAP	Non-Comp	Protein Co	30	GC01P024	0.94
7528	EDIL3	EGF Like R	Protein Co	40	GC05M083	0.94
7529	EEF2K	Eukaryotic	Protein Co	47	GC16P022	0.94
7530	TRPS1	Transcripti	Protein Co	45	GC08M115	0.94
7531	KPNA2	Karyopheri	Protein Co	45	GC17P068	0.94
7532	OTUD5	OTU Deub	Protein Co	35	GC0XM048	0.94
7533	ST2	Suppressic	Genetic Lo	6	GC11U990	0.93
7534	TMPRSS11	Transmem	Protein Co	39	GC04M067	0.93
7535	FHL5	Four And A	Protein Co	37	GC06P096	0.93
7536	C12orf4	Chromoso	Protein Co	36	GC12M004	0.93
7537	NT5DC2	5'-Nucleot	Protein Co	35	GC03M052	0.93
7538	KLHDC4	Kelch Dom	Protein Co	37	GC16M087	0.93
7539	DNM3	Dynamin 3	Protein Co	43	GC01P171	0.93
7540	STX6	Syntaxin 6	Protein Co	40	GC01M180	0.93
7541	ENOPH1	Enolase-Ph	Protein Co	39	GC04P082	0.93
7542	TIMM17B	Translocas	Protein Co	37	GC0XM048	0.93
7543	MKX	Mohawk H	Protein Co	36	GC10M027	0.93
7544	PRODH2	Proline De	Protein Co	36	GC19M035	0.93
7545	ASB18	Ankyrin Re	Protein Co	32	GC02M236	0.93
7546	CYLC1	Cylicin 1	Protein Co	32	GC0XP083	0.93
7547	LARGE-AS1	LARGE Ant	RNA Gene	14	GC22P033	0.93
7548	WFDC21P	WAP Four-	Pseudoger	12	GC17M060	0.93
7549	THCAT158	Thyroid Ca	RNA Gene	8	GC17M047	0.93
7550	STAM	Signal Trar	Protein Co	42	GC10P017	0.93
7551	CACNG2	Calcium Vo	Protein Co	44	GC22M036	0.93
7552	PRKRA	Protein Ac	Protein Co	42	GC02M178	0.93
7553	OVGP1	Oviductal (	Protein Co	40	GC01M111	0.93
7554	TATDN1	TatD DNas	Protein Co	35	GC08M124	0.93
7555	FUT9	Fucosyltrar	Protein Co	41	GC06P096	0.92
7556	MYL4	Myosin Lig	Protein Co	44	GC17P047	0.92
7557	P2RX6	Purinergic	Protein Co	40	GC22P021	0.92
7558	OCM2	Oncomodu	Protein Co	27	GC07M097	0.92
7559	ACSM3	Acyl-CoA S	Protein Co	39	GC16P020	0.92
7560	TRAPPC2L	Trafficking	Protein Co	37	GC16P088	0.92
7561	MTFP1	Mitochond	Protein Co	32	GC22P030	0.92
7562	LINC01629	Long Inter	RNA Gene	11	GC14P076	0.92
7563	SCUBE2	Signal Pep	Protein Co	39	GC11M009	0.92
7564	LACRT	Lacritin	Protein Co	34	GC12M054	0.92
7565	PSG1	Pregnancy	Protein Co	39	GC19M042	0.92
7566	PRPSAP2	Phosphoril	Protein Co	34	GC17P018	0.91

7567	CNNM3	Cyclin And	Protein Co	37	GC02P096	0.91
7568	GULP1	GULP PTB	Protein Co	36	GC02P188	0.91
7569	CCDC78	Coiled-Coi	Protein Co	36	GC16M001	0.91
7570	SLN	Sarcolipin	Protein Co	32	GC11M107	0.91
7571	SMARCAD	SWI/SNF-R	Protein Co	45	GC04P094	0.91
7572	LUZP2	Leucine Zi	Protein Co	34	GC11P024	0.91
7573	IGKC	Immunogl	Protein Co	32	GC02M089	0.91
7574	SLC8A2	Solute Car	Protein Co	40	GC19M047	0.91
7575	C9orf78	Chromoso	Protein Co	33	GC09M129	0.91
7576	APCS	Amyloid P	Protein Co	41	GC01P159	0.91
7577	TMEM97	Transmem	Protein Co	35	GC17P028	0.91
7578	TRIM32	Tripartite M	Protein Co	43	GC09P116	0.91
7579	IPCEF1	Interaction	Protein Co	35	GC06M154	0.91
7580	MIR3129	MicroRNA	RNA Gene	12	GC02M189	0.9
7581	piR-40250-007		RNA Gene	4	GC16M053	0.9
7582	WFIKKN2	WAP, Follis	Protein Co	35	GC17P050	0.9
7583	ZPLD1	Zona Pellu	Protein Co	32	GC03P102	0.9
7584	ESAM	Endothelia	Protein Co	40	GC11M124	0.9
7585	lnc-TRIL-3		RNA Gene	4	GC07M028	0.9
7586	HROB	Homologo	Protein Co	26	GC17P044	0.9
7587	CDNF	Cerebral D	Protein Co	36	GC10M014	0.9
7588	CDH10	Cadherin 1	Protein Co	39	GC05M024	0.9
7589	POGK	Pogo Tran	Protein Co	36	GC01P166	0.9
7590	PLD5	Phospholip	Protein Co	35	GC01M242	0.9
7591	GLRA4	Glycine Re	Pseudoger	33	GC0XM103	0.9
7592	LOC10192	Uncharacte	RNA Gene	9	GC05P016	0.9
7593	LOC10144	Uncharacte	RNA Gene	8	GC09M134	0.9
7594	CDC42EP3	CDC42 Eff	Protein Co	38	GC02M037	0.9
7595	COL6A5	Collagen T	Protein Co	33	GC03P130	0.89
7596	GRIA4	Glutamate	Protein Co	48	GC11P105	0.89
7597	CLEC4D	C-Type Le	Protein Co	37	GC12P008	0.89
7598	FGD1	FYVE, Rho	Protein Co	41	GC0XM054	0.89
7599	TBXT	T-Box Tran	Protein Co	32	GC06M166	0.89
7600	BCAT1	Branched	Protein Co	46	GC12M024	0.89
7601	SSX2IP	SSX Family	Protein Co	36	GC01M084	0.89
7602	ETV2	ETS Varian	Protein Co	34	GC19P038	0.89
7603	TPST1	Tyrosylpro	Protein Co	40	GC07P066	0.89
7604	USP49	Ubiquitin S	Protein Co	37	GC06M042	0.88
7605	SLC8A3	Solute Car	Protein Co	43	GC14M070	0.88
7606	RAD1	RAD1 Che	Protein Co	39	GC05M034	0.88
7607	TIAM2	TIAM Rac1	Protein Co	40	GC06P154	0.88
7608	ENTPD4	Ectonuclec	Protein Co	37	GC08M023	0.88
7609	ENTPD2	Ectonuclec	Protein Co	39	GC09M137	0.88

7610	VPS41	VPS41 Sub	Protein Co	38	GC07M038	0.88
7611	KCNC3	Potassium	Protein Co	44	GC19M050	0.88
7612	METTL21C	Methyltran	Protein Co	31	GC13M102	0.88
7613	SREK1IP1	SREK1 Inte	Protein Co	31	GC05M064	0.88
7614	TREML4	Triggering	Protein Co	31	GC06P041	0.88
7615	H3C6	H3 Cluster	Protein Co	28	GC06P028	0.88
7616	EIF5AL1	Eukaryotic	Protein Co	28	GC10P079	0.88
7617	DKFZP434I	Uncharacte	RNA Gene	10	GC16M056	0.88
7618	TIMM8AP1	Translocas	Pseudoger	7	GC02M162	0.88
7619	METTL4	Methyltran	Protein Co	33	GC18M002	0.88
7620	SERPINB12	Serpin Fan	Protein Co	36	GC18P063	0.88
7621	ENSG00000224063		RNA Gene	7	GC02P187	0.87
7622	BBS12	Bardet-Bie	Protein Co	35	GC04P122	0.87
7623	THUMPD3	THUMPD3	RNA Gene	13	GC03M009	0.87
7624	RMDN2	Regulator	Protein Co	32	GC02P037	0.87
7625	SYT14	Synaptotag	Protein Co	37	GC01P209	0.87
7626	CEP128	Centrosom	Protein Co	32	GC14M080	0.87
7627	LINC00443	Long Inter	RNA Gene	14	GC13P106	0.87
7628	LOC40112	WD Repea	Pseudoger	9	GC04P039	0.87
7629	ENSG00000239381		RNA Gene	8	GC03M181	0.87
7630	HSALNG0021037		RNA Gene	4	GC02P189	0.87
7631	lnc-KIDINS220-12		RNA Gene	4	GC02M008	0.87
7632	ENSG00000285653		RNA Gene	3	GC18M009	0.87
7633	MIR665	MicroRNA	RNA Gene	12	GC14P104	0.87
7634	AKAP6	A-Kinase A	Protein Co	40	GC14P032	0.86
7635	BRS3	Bombesin	Protein Co	43	GC0XP136	0.86
7636	PRSS12	Serine Pro	Protein Co	40	GC04M118	0.86
7637	SLC24A1	Solute Car	Protein Co	44	GC15P065	0.86
7638	TRIM38	Tripartite M	Protein Co	37	GC06P025	0.86
7639	ILRUN	Inflammati	Protein Co	26	GC06M042	0.86
7640	ADPRS	ADP-Ribos	Protein Co	30	GC01P036	0.86
7641	COL27A1	Collagen T	Protein Co	40	GC09P114	0.86
7642	GJC3	Gap Juncti	Protein Co	39	GC07M099	0.86
7643	MTMR6	Myotubula	Protein Co	39	GC13M025	0.86
7644	PIGF	Phosphatic	Protein Co	37	GC02M046	0.86
7645	MRPL36	Mitochond	Protein Co	35	GC05M001	0.86
7646	INSM2	INSM Tran	Protein Co	34	GC14P035	0.86
7647	OFCC1	Orofacial C	Protein Co	32	GC06M009	0.86
7648	ZBBX	Zinc Finge	Protein Co	32	GC03M167	0.86
7649	TGFB2-AS1	TGFB2 Ant	RNA Gene	12	GC01M218	0.86
7650	TMOD4	Tropomod	Protein Co	37	GC01M151	0.86
7651	KCNN2	Potassium	Protein Co	43	GC05P114	0.86
7652	LIN54	Lin-54 DRE	Protein Co	36	GC04M082	0.86

7653	GZMK	Granzyme	Protein Co	37	GC05P055	0.86
7654	MIR511	MicroRNA	RNA Gene	15	GC10P017	0.85
7655	SYTL5	Synaptotag	Protein Co	33	GC0XP037	0.85
7656	MIR1306	MicroRNA	RNA Gene	17	GC22P020	0.85
7657	FAAH2	Fatty Acid	Protein Co	37	GC0XP057	0.85
7658	MYL3	Myosin Lig	Protein Co	44	GC03M046	0.85
7659	MGAT4C	MGAT4 Fa	Protein Co	39	GC12M085	0.85
7660	JKAMP	JNK1/MAP	Protein Co	34	GC14P059	0.85
7661	POLR3K	RNA Polyn	Protein Co	39	GC16M000	0.85
7662	LINC01722	Long Inter	RNA Gene	9	GC20M012	0.85
7663	EPHA5	EPH Recep	Protein Co	45	GC04M065	0.85
7664	NOS1AP	Nitric Oxid	Protein Co	37	GC01P162	0.85
7665	ADAM33	ADAM Me	Protein Co	36	GC20M003	0.84
7666	CRHBP	Corticotrop	Protein Co	40	GC05P076	0.84
7667	TSPAN9	Tetraspanin	Protein Co	39	GC12P003	0.84
7668	ENSG00000212618		RNA Gene	5	GC17P018	0.84
7669	MIR4505	MicroRNA	RNA Gene	13	GC14P073	0.84
7670	SYT9	Synaptotag	Protein Co	36	GC11P007	0.84
7671	PHKA1	Phosphory	Protein Co	43	GC0XM072	0.84
7672	CALR3	Calreticulin	Protein Co	37	GC19M016	0.84
7673	THAP4	THAP Dom	Protein Co	33	GC02M241	0.84
7674	H3C7	H3 Cluster	Protein Co	28	GC06M027	0.84
7675	MIR4260	MicroRNA	RNA Gene	13	GC01M209	0.84
7676	LOC101928	Uncharacte	RNA Gene	9	GC11P009	0.84
7677	MIR6793	MicroRNA	RNA Gene	8	GC19P010	0.84
7678	SYNC	Syncoilin, I	Protein Co	33	GC01M032	0.84
7679	CNPY2	Canopy FG	Protein Co	35	GC12M056	0.84
7680	ABRAXAS2	Abraxas 2,	Protein Co	28	GC10P124	0.84
7681	AVEN	Apoptosis	Protein Co	36	GC15M033	0.84
7682	NPNT	Nephronec	Protein Co	38	GC04P105	0.83
7683	ISM1	Isthmin 1	Protein Co	33	GC20P013	0.83
7684	GUCY2D	Guanylate	Protein Co	43	GC17P008	0.83
7685	lnc-SHANK1-3		RNA Gene	2	GC19M050	0.83
7686	DYRK1B	Dual Speci	Protein Co	46	GC19M039	0.83
7687	MYO10	Myosin X	Protein Co	39	GC05M016	0.83
7688	STK3	Serine/Thr	Protein Co	46	GC08M098	0.83
7689	FGF16	Fibroblast	Protein Co	41	GC0XP077	0.83
7690	USP50	Ubiquitin S	Protein Co	33	GC15M050	0.83
7691	SPOCD1	SPOC Dom	Protein Co	31	GC01M031	0.82
7692	H2BC11	H2B Cluste	Protein Co	27	GC06M027	0.82
7693	COL22A1	Collagen T	Protein Co	36	GC08M138	0.82
7694	VASH1	Vasohibin	Protein Co	37	GC14P076	0.82
7695	ENAM	Enamelin	Protein Co	39	GC04P070	0.82

7696	JSRP1	Junctional	Protein Co	35	GC19M002	0.82
7697	KRT39	Keratin 39	Protein Co	33	GC17M040	0.82
7698	PET117	PET117 Cy	Protein Co	29	GC20P018	0.82
7699	PART1	Prostate A	RNA Gene	24	GC05P060	0.82
7700	LINC00575	Long Inter	RNA Gene	18	GC04M082	0.82
7701	SCARNA22	Small Caja	RNA Gene	16	GC04P001	0.82
7702	LINC00163	Long Inter	RNA Gene	16	GC21M044	0.82
7703	SKAP1-AS1	SKAP1 Ant	RNA Gene	9	GC17P048	0.82
7704	KCNRG	Potassium	Protein Co	35	GC13P050	0.82
7705	TMIGD3	Transmem	Protein Co	17	GC01M111	0.81
7706	MAS1	MAS1 Prot	Protein Co	40	GC06P159	0.81
7707	SLC32A1	Solute Car	Protein Co	42	GC20P038	0.81
7708	IGES	Immunogl	Genetic Lo	4	GC05U990	0.81
7709	CAMK2A	Calcium/C	Protein Co	49	GC05M150	0.81
7710	TRPM1	Transient F	Protein Co	43	GC15M031	0.81
7711	PROKR1	Prokinetici	Protein Co	37	GC02P068	0.81
7712	ANKFN1	Ankyrin Re	Protein Co	33	GC17P055	0.81
7713	THAP5	THAP Dom	Protein Co	36	GC07M108	0.81
7714	MIR136	MicroRNA	RNA Gene	19	GC14P104	0.8
7715	BBOX1	Gamma-Bu	Protein Co	41	GC11P027	0.8
7716	NGEF	Neuronal G	Protein Co	38	GC02M232	0.8
7717	CYP2A13	Cytochrom	Protein Co	41	GC19P041	0.8
7718	MUC19	Mucin 19,	Protein Co	29	GC12P040	0.8
7719	LARP4B	La Ribonuc	Protein Co	33	GC10M000	0.8
7720	TPP2	Tripeptidyl	Protein Co	41	GC13P102	0.8
7721	OR5H2	Olfactory F	Protein Co	29	GC03P098	0.8
7722	PARS2	Prolyl-TRN	Protein Co	41	GC01M054	0.8
7723	MAP3K19	Mitogen-A	Protein Co	32	GC02M134	0.8
7724	HLA-T	Major Hist	Pseudoger	9	GC06P047	0.79
7725	L13713-141		RNA Gene	4	GC03P012	0.79
7726	CYP26B1	Cytochrom	Protein Co	45	GC02M072	0.79
7727	TMEM241	Transmem	Protein Co	31	GC18M023	0.79
7728	PRDM9	PR/SET Do	Protein Co	39	GC05P023	0.79
7729	LINC00470	Long Inter	RNA Gene	21	GC18M001	0.79
7730	SNORD1A	Small Nucl	RNA Gene	13	GC17P076	0.79
7731	TMPO-AS1	TMPO Ant	RNA Gene	13	GC12M098	0.79
7732	SEPT5-GP1	SEPT5-GP1	RNA Gene	11	GC22P019	0.79
7733	BEX4	Brain Expr	Protein Co	29	GC0XP103	0.79
7734	GABPA	GA Bindin	Protein Co	39	GC21P025	0.79
7735	PTH2R	Parathyroi	Protein Co	43	GC02P208	0.79
7736	UBE2O	Ubiquitin C	Protein Co	40	GC17M076	0.79
7737	C7orf50	Chromoso	Protein Co	32	GC07M000	0.79
7738	EDARADD	EDAR Assc	Protein Co	39	GC01P236	0.78

7739	GYS2	Glycogen S	Protein Co	44	GC12M021	0.78
7740	NDNF	Neuron De	Protein Co	33	GC04M121	0.78
7741	APCDD1	APC Down	Protein Co	40	GC18P010	0.78
7742	PGAP1	Post-GPI A	Protein Co	40	GC02M196	0.78
7743	RTN4RL2	Reticulon 4	Protein Co	35	GC11P057	0.78
7744	KCNS1	Potassium	Protein Co	39	GC20M045	0.78
7745	KLK5	Kallikrein F	Protein Co	41	GC19M050	0.77
7746	DNAJB4	DnaJ Heat	Protein Co	37	GC01P077	0.77
7747	LINC01619	Long Inter	RNA Gene	17	GC12M091	0.77
7748	DGKH	Diacylglyce	Protein Co	41	GC13P042	0.77
7749	MIR30C2	MicroRNA	RNA Gene	19	GC06M071	0.77
7750	CASP12	Caspase 12	Protein Co	32	GC11M104	0.77
7751	IL36G	Interleukin	Protein Co	38	GC02P112	0.77
7752	SYT17	Synaptotag	Protein Co	33	GC16P019	0.77
7753	HBB-LCR	Beta-Globi	Biological	4	GC11P005	0.76
7754	COL20A1	Collagen T	Protein Co	35	GC20P063	0.76
7755	SSPOP	SCO-Spon	Pseudoger	20	GC07P149	0.76
7756	NMNAT2	Nicotinami	Protein Co	41	GC01M183	0.76
7757	TP53INP2	Tumor Pro	Protein Co	35	GC20P034	0.76
7758	POLR2L	RNA Polyn	Protein Co	41	GC11M000	0.76
7759	EPS15L1	Epidermal	Protein Co	40	GC19M016	0.76
7760	BRDT	Bromodom	Protein Co	42	GC01P091	0.76
7761	PCDH15	Protocadh	Protein Co	39	GC10M053	0.76
7762	TREML2	Triggering	Protein Co	39	GC06M042	0.76
7763	KIF2B	Kinesin Fa	Protein Co	37	GC17P053	0.76
7764	LRIT1	Leucine Ri	Protein Co	35	GC10M084	0.76
7765	MAGEC1	MAGE Fam	Protein Co	33	GC0XP141	0.76
7766	TUBGCP5	Tubulin Ga	Protein Co	32	GC15M022	0.76
7767	TAAR9	Trace Amir	Protein Co	32	GC06P132	0.76
7768	SDHAF3	Succinate I	Protein Co	31	GC07P097	0.76
7769	SPIN2A	Spindlin Fa	Protein Co	30	GC0XM057	0.76
7770	XAGE3	X Antigen	Protein Co	28	GC0XM052	0.76
7771	MIR3651	MicroRNA	RNA Gene	15	GC09M092	0.76
7772	MIR548AW	MicroRNA	RNA Gene	11	GC09P132	0.76
7773	LINC00165	Long Inter	RNA Gene	11	GC21M047	0.76
7774	FLT1P1	FLT1 Pseu	Pseudoger	6	GC03M046	0.76
7775	MELTF-AS1	MELTF Ant	RNA Gene	12	GC03P197	0.76
7776	NHLRC2	NHL Repea	Protein Co	36	GC10P113	0.76
7777	LHCGR	Luteinizing	Protein Co	47	GC02M048	0.76
7778	SCUBE1	Signal Pep	Protein Co	36	GC22M043	0.76
7779	P2RY14	Purinergic	Protein Co	42	GC03M151	0.76
7780	TNIP3	TNFAIP3 In	Protein Co	36	GC04M121	0.76
7781	CCN6	Cellular Co	Protein Co	33	GC06P112	0.76

7782	KIR2DS1	Killer Cell I	Protein Co	22	GC19Mr00	0.75
7783	SEZ6L	Seizure Re	Protein Co	38	GC22P026	0.75
7784	TIMM50	Translocas	Protein Co	38	GC19P039	0.75
7785	LINC02159	Long Inter	RNA Gene	9	GC05M160	0.75
7786	lnc-TEX35-4		RNA Gene	4	GC01P178	0.75
7787	lnc-TEX35-5		RNA Gene	4	GC01P178	0.75
7788	lnc-SHANK1-2		RNA Gene	4	GC19M050	0.75
7789	lnc-SMIM14-4		RNA Gene	2	GC04M039	0.75
7790	HAS3	Hyaluronai	Protein Co	40	GC16P069	0.75
7791	GUCY1B1	Guanylate	Protein Co	33	GC04P155	0.75
7792	TECTB	Tectorin Be	Protein Co	31	GC10P112	0.74
7793	OBSCN	Obscurin, (	Protein Co	40	GC01P228	0.74
7794	FBXO3	F-Box Prot	Protein Co	37	GC11M033	0.74
7795	ASIC3	Acid Sensi	Protein Co	39	GC07P151	0.74
7796	NRG4	Neuregulir	Protein Co	39	GC15M075	0.74
7797	LOC10783	SIRT1 Pror	Biological	1	GC10P067	0.74
7798	PRDM4	PR/SET Do	Protein Co	35	GC12M107	0.74
7799	JAM2	Junctional	Protein Co	41	GC21P025	0.74
7800	KCNC4	Potassium	Protein Co	41	GC01P110	0.74
7801	B3GALT4	Beta-1,3-G	Protein Co	40	GC06P033	0.73
7802	RENBP	Renin Bind	Protein Co	41	GC0XM153	0.73
7803	ZDHHC17	Zinc Finge	Protein Co	37	GC12P076	0.73
7804	APOL4	Apolipopro	Protein Co	36	GC22M036	0.73
7805	ARHGAP24	Rho GTPas	Protein Co	39	GC04P085	0.73
7806	FGL1	Fibrinogen	Protein Co	40	GC08M017	0.73
7807	MCCC1	Methylcrot	Protein Co	44	GC03M183	0.73
7808	ARHGEF11	Rho Guani	Protein Co	41	GC01M156	0.73
7809	XCL2	X-C Motif	Protein Co	31	GC01M168	0.73
7810	KCNQ4	Potassium	Protein Co	44	GC01P040	0.72
7811	ZNF331	Zinc Finge	Protein Co	40	GC19P053	0.72
7812	RDH12	Retinol De	Protein Co	45	GC14P067	0.72
7813	TANC1	Tetratricop	Protein Co	33	GC02P158	0.72
7814	PDE1C	Phosphodi	Protein Co	45	GC07M031	0.72
7815	MIR26A2	MicroRNA	RNA Gene	20	GC12M057	0.72
7816	ABCA4	ATP Bindir	Protein Co	44	GC01M093	0.72
7817	GPC5	Glypican 5	Protein Co	40	GC13P091	0.72
7818	MAP1LC3C	Microtubul	Protein Co	33	GC01M241	0.72
7819	OR51F1	Olfactory F	Protein Co	28	GC11M004	0.72
7820	LINC02258	Long Inter	RNA Gene	9	GC12P087	0.72
7821	RNU12-2P	RNA, U12	Pseudoger	8	GC0XP047	0.72
7822	CKAP4	Cytoskelet	Protein Co	37	GC12M106	0.72
7823	INTS10	Integrator	Protein Co	35	GC08P019	0.72
7824	GTF2A1	General Tr	Protein Co	36	GC14M081	0.72

7825	ANGPTL6	Angiopoie	Protein Co	39	GC19M01(	0.72
7826	RSRC1	Arginine A	Protein Co	39	GC03P158	0.72
7827	CNDP1	Carnosine	Protein Co	44	GC18P074	0.71
7828	MYH1	Myosin He	Protein Co	40	GC17M01(	0.71
7829	CDK17	Cyclin Dep	Protein Co	39	GC12M096	0.71
7830	IGFBPL1	Insulin Like	Protein Co	36	GC09M038	0.71
7831	CD300E	CD300e M	Protein Co	35	GC17M074	0.71
7832	MIR320C1	MicroRNA	RNA Gene	16	GC18P021(	0.71
7833	ATOH8	Atonal BHI	Protein Co	33	GC02P085	0.71
7834	LINC01146	Long Inter	RNA Gene	12	GC14P088(	0.71
7835	LECT2	Leukocyte	Protein Co	36	GC05M135	0.71
7836	SDF2	Stromal Ce	Protein Co	39	GC17M029	0.71
7837	MYRIP	Myosin VII	Protein Co	39	GC03P039	0.71
7838	CHADL	Chondroac	Protein Co	35	GC22M045	0.71
7839	NR0B1	Nuclear Re	Protein Co	46	GC0XM030	0.71
7840	RFC1	Replicatior	Protein Co	45	GC04M039	0.71
7841	CTNND2	Catenin De	Protein Co	41	GC05M01(	0.71
7842	PUS3	Pseudouric	Protein Co	37	GC11M125	0.71
7843	ACTR1B	Actin Relat	Protein Co	39	GC02M097	0.7
7844	AMOTL2	Angiomoti	Protein Co	35	GC03M134	0.7
7845	MIR219A1	MicroRNA	RNA Gene	19	GC06P033	0.7
7846	NLRP4	NLR Family	Protein Co	37	GC19P055	0.7
7847	MRC2	Mannose F	Protein Co	40	GC17P062(	0.7
7848	DCTN6	Dynactin S	Protein Co	35	GC08P030	0.7
7849	ALKBH8	AlkB Hom(	Protein Co	37	GC11M107	0.7
7850	ZBED1	Zinc Finge	Protein Co	34	GC0XM002	0.7
7851	CRLS1	Cardiolipin	Protein Co	39	GC20P006(	0.7
7852	MIR300	MicroRNA	RNA Gene	13	GC14P104	0.7
7853	FGD3	FYVE, Rho(	Protein Co	39	GC09P092	0.7
7854	RESP18	Regulated	Protein Co	31	GC02M219	0.7
7855	ENSG00000288587		RNA Gene	4	GC06P047	0.7
7856	lnc-RNF39-8		RNA Gene	4	GC06M030	0.7
7857	lnc-HLA-A-2		RNA Gene	4	GC06P047	0.7
7858	RDH5	Retinol De	Protein Co	46	GC12P055	0.7
7859	BMX	BMX Non-	Protein Co	44	GC0XP015	0.7
7860	CAPS	Calcyphosi	Protein Co	37	GC19P005	0.7
7861	MIR1307	MicroRNA	RNA Gene	17	GC10M103	0.69
7862	NOX3	NADPH O>	Protein Co	40	GC06M155	0.69
7863	MAP7D1	MAP7 Dor	Protein Co	32	GC01P036	0.69
7864	POLR1G	RNA Polyn	Protein Co	27	GC19P045	0.69
7865	FUT7	Fucosyltrar	Protein Co	41	GC09M137	0.69
7866	TSPAN15	Tetraspani	Protein Co	38	GC10P069	0.69
7867	JPH2	Junctophili	Protein Co	40	GC20M044	0.69

7868	ZNF667	Zinc Finge	Protein Co	36	GC19M056	0.69
7869	AK5	Adenylate	Protein Co	42	GC01P077	0.69
7870	B3GALT5-A	B3GALT5 A	RNA Gene	20	GC21M039	0.69
7871	DCLK2	Doublecor	Protein Co	40	GC04P150	0.68
7872	MIR153-1	MicroRNA	RNA Gene	14	GC02M219	0.68
7873	FAM126A	Family Wit	Protein Co	37	GC07M022	0.68
7874	FAM168A	Family Wit	Protein Co	33	GC11M073	0.68
7875	CD2-LCR	CD2 Locus	Biological	1	GC01P116	0.68
7876	SLC16A12	Solute Car	Protein Co	37	GC10M089	0.68
7877	OLFML3	Olfactome	Protein Co	37	GC01P113	0.68
7878	QPRT	Quinolinat	Protein Co	43	GC16P029	0.68
7879	CD300LB	CD300 Mo	Protein Co	36	GC17M074	0.68
7880	SIGLEC14	Sialic Acid	Protein Co	32	GC19M051	0.68
7881	MIR544A	MicroRNA	RNA Gene	14	GC14P104	0.68
7882	EFNB3	Ephrin B3	Protein Co	42	GC17P008	0.68
7883	HBG1	Hemoglob	Protein Co	42	GC11M005	0.67
7884	EOLA1	Endotheliu	Protein Co	26	GC0XP149	0.67
7885	CDC14A	Cell Divisic	Protein Co	43	GC01P100	0.67
7886	ANKRD2	Ankyrin Re	Protein Co	36	GC10P097	0.67
7887	CRYBB2	Crystallin E	Protein Co	40	GC22P025	0.67
7888	IL20RB	Interleukin	Protein Co	37	GC03P136	0.67
7889	ZNF32	Zinc Finge	Protein Co	34	GC10M043	0.67
7890	VASN	Vasorin	Protein Co	36	GC16P004	0.67
7891	GRM4	Glutamate	Protein Co	44	GC06M042	0.67
7892	KLF3-AS1	KLF3 Antis	RNA Gene	17	GC04M038	0.67
7893	AMD1P4	Adenosylm	Pseudoger	5	GC06M011	0.67
7894	ENSG00000273384		RNA Gene	5	GC01P178	0.67
7895	TRPC3	Transient F	Protein Co	48	GC04M121	0.67
7896	PAFAH2	Platelet Ac	Protein Co	39	GC01M025	0.66
7897	MYF6	Myogenic	Protein Co	41	GC12P080	0.66
7898	SPNS2	Sphingolip	Protein Co	35	GC17P004	0.66
7899	SCPEP1	Serine Carl	Protein Co	37	GC17P056	0.65
7900	SERPINA10	Serpin Fan	Protein Co	39	GC14M094	0.65
7901	TLL1	Tolloid Lik	Protein Co	43	GC04P165	0.65
7902	TSBP1-AS1	TSBP1 Anc	RNA Gene	9	GC06P049	0.65
7903	PPP1R9B	Protein Ph	Protein Co	39	GC17M050	0.65
7904	PRKRIP1	PRKR Inter	Protein Co	32	GC07P102	0.65
7905	BDNF-AS	BDNF Anti	RNA Gene	18	GC11P027	0.65
7906	MYH15	Myosin He	Protein Co	38	GC03M108	0.65
7907	CAPN8	Calpain 8	Protein Co	37	GC01M223	0.65
7908	MC2R	Melanocor	Protein Co	47	GC18M017	0.65
7909	GNG2	G Protein S	Protein Co	43	GC14P051	0.65
7910	CCN3	Cellular Cc	Protein Co	33	GC08P119	0.64

7911	MIR1290	MicroRNA	RNA Gene	13	GC01M018	0.64
7912	LRP3	LDL Recep	Protein Co	37	GC19P033	0.64
7913	UTS2B	Urotensin	Protein Co	31	GC03M191	0.64
7914	ART3	ADP-Ribos	Protein Co	40	GC04P076	0.64
7915	FAM111B	Family Wit	Protein Co	36	GC11P059	0.64
7916	FGD5-AS1	FGD5 Anti	RNA Gene	14	GC03M015	0.64
7917	UTS2R	Urotensin	Protein Co	41	GC17P082	0.64
7918	LINC01322	Long Inter	RNA Gene	11	GC03P165	0.64
7919	KRT18P19	Keratin 18	Pseudoger	8	GC02M189	0.64
7920	ENSG00000276690		RNA Gene	5	GC03P181	0.64
7921	lnc-CCDC89-6		RNA Gene	2	GC11M085	0.64
7922	piR-36393-007		RNA Gene	2	GC01M021	0.64
7923	piR-31432-008		RNA Gene	2	GC01P021	0.64
7924	CHODL	Chondrole	Protein Co	36	GC21P017	0.64
7925	STK32B	Serine/Thr	Protein Co	33	GC04P005	0.64
7926	LINC00520	Long Inter	RNA Gene	17	GC14M055	0.64
7927	MIR133A1	MicroRNA	RNA Gene	17	GC18M021	0.63
7928	PRPH2	Peripherin	Protein Co	39	GC06M043	0.63
7929	DDN	Dendrin	Protein Co	32	GC12M048	0.63
7930	S100A3	S100 Calci	Protein Co	39	GC01M153	0.63
7931	PDZD7	PDZ Doma	Protein Co	37	GC10M101	0.63
7932	CCL4L1	C-C Motif	Protein Co	26	GC17Pj002	0.63
7933	IFN1@	Interferon,	Gene Clust	4	GC09U990	0.63
7934	KRT76	Keratin 76	Protein Co	34	GC12M052	0.63
7935	NIFK	Nucleolar I	Protein Co	33	GC02M121	0.63
7936	FSTL3	Follistatin	Protein Co	37	GC19P000	0.63
7937	CAMK2N1	Calcium/C	Protein Co	32	GC01M020	0.63
7938	GNL1	G Protein I	Protein Co	36	GC06M030	0.63
7939	DPH6-DT	DPH6 Dive	RNA Gene	11	GC15P035	0.63
7940	GRHL1	Grainyhea	Protein Co	35	GC02P009	0.62
7941	MIR320B1	MicroRNA	RNA Gene	14	GC01P116	0.62
7942	CST1	Cystatin SI	Protein Co	39	GC20M023	0.62
7943	MIR429	MicroRNA	RNA Gene	19	GC01P001	0.62
7944	MYH4	Myosin He	Protein Co	39	GC17M010	0.61
7945	STAB2	Stabilin 2	Protein Co	39	GC12P103	0.61
7946	ASIP	Agouti Sig	Protein Co	37	GC20P034	0.61
7947	HRNR	Hornerin	Protein Co	34	GC01M152	0.61
7948	MIR376C	MicroRNA	RNA Gene	17	GC14P104	0.61
7949	KLF15	Kruppel Lil	Protein Co	41	GC03M126	0.61
7950	CYP2F1	Cytochrom	Protein Co	41	GC19P041	0.6
7951	DCBLD2	Discoidin,	Protein Co	40	GC03M098	0.6
7952	KCNMB4	Potassium	Protein Co	40	GC12P070	0.6
7953	TAFA2	TAFA Cher	Protein Co	28	GC12M061	0.6

7954	MIRLET7A2	MicroRNA	RNA Gene	20	GC11M122	0.6
7955	KCNH8	Potassium	Protein Co	40	GC03P019	0.6
7956	RRAD	RRAD, Ras	Protein Co	39	GC16M066	0.6
7957	RLN3	Relaxin 3	Protein Co	36	GC19P014	0.6
7958	SNORA40	Small Nucl	RNA Gene	16	GC11M094	0.6
7959	LINC00895	Long Inter	RNA Gene	12	GC22M019	0.6
7960	MIR4460	MicroRNA	RNA Gene	10	GC05M129	0.6
7961	ENSG00000233611		RNA Gene	8	GC02P236	0.6
7962	SERPINA11	Serpin Fam	Protein Co	33	GC14M094	0.6
7963	RNASE7	Ribonuclea	Protein Co	35	GC14P021	0.59
7964	RFC5	Replicatior	Protein Co	40	GC12P118	0.59
7965	KIAA1143	KIAA1143	Protein Co	30	GC03M044	0.59
7966	MMP16	Matrix Met	Protein Co	44	GC08M088	0.59
7967	IGFL3	IGF Like Fa	Protein Co	31	GC19M046	0.59
7968	PA2G4P2	Proliferatic	Pseudoger	7	GC20P012	0.59
7969	SLC26A11	Solute Car	Protein Co	38	GC17P080	0.59
7970	ENHO	Energy Ho	Protein Co	30	GC09M034	0.59
7971	MTRNR2L1	MT-RNR2	Protein Co	25	GC17P026	0.59
7972	KIR3DL2	Killer Cell I	Protein Co	37	GC19P055	0.59
7973	AIFM2	Apoptosis	Protein Co	39	GC10M070	0.59
7974	H2BC10	H2B Cluste	Protein Co	28	GC06P028	0.59
7975	CDH4	Cadherin 4	Protein Co	41	GC20P061	0.58
7976	CCN5	Cellular Co	Protein Co	31	GC20P044	0.58
7977	LINC01079	Long Inter	RNA Gene	8	GC13P027	0.58
7978	ARHGAP4	Rho GTPas	Protein Co	41	GC0XM153	0.58
7979	FBLN7	Fibulin 7	Protein Co	35	GC02P115	0.58
7980	STXBP3	Syntaxin B	Protein Co	40	GC01P108	0.58
7981	TP53AIP1	Tumor Pro	Protein Co	36	GC11M128	0.58
7982	MIR766	MicroRNA	RNA Gene	16	GC0XM119	0.58
7983	SUCNR1	Succinate I	Protein Co	40	GC03P151	0.57
7984	WNK3	WNK Lysin	Protein Co	39	GC0XM054	0.57
7985	CSRP3	Cysteine A	Protein Co	41	GC11M019	0.57
7986	STK35	Serine/Thr	Protein Co	36	GC20P002	0.57
7987	CCDC124	Coiled-Coi	Protein Co	31	GC19P023	0.57
7988	GRK1	G Protein-(	Protein Co	42	GC13P113	0.57
7989	INTS2	Integrator	Protein Co	35	GC17M061	0.56
7990	CLDN10	Claudin 10	Protein Co	41	GC13P095	0.56
7991	SPRR1A	Small Proli	Protein Co	36	GC01P152	0.56
7992	NKPD1	NTPase KA	Protein Co	30	GC19M045	0.56
7993	KRTAP5-5	Keratin Ass	Protein Co	27	GC11P001	0.56
7994	CNTFR	Ciliary Neu	Protein Co	43	GC09M034	0.56
7995	SNX12	Sorting Ne	Protein Co	35	GC0XM071	0.56
7996	LOC10050	Putative D	Protein Co	14	GC09P026	0.56

7997	RNA5SP44	RNA, 5S Ri	Pseudoger	8	GC18M009	0.56
7998	ENSG00000212273		RNA Gene	6	GC08M133	0.56
7999	HLA-X	Major Hist	Pseudoger	5	GC06M031	0.56
8000	lnc-HLA-DRB1-6		RNA Gene	4	GC06M032	0.56
8001	ENSG00000233902		Pseudoger	4	GC06M031	0.56
8002	RF00001-149		RNA Gene	4	GC18M009	0.56
8003	lnc-ATP6V1G2-DDX39		RNA Gene	3	GC06M032	0.56
8004	lnc-HLA-DQA1-8		RNA Gene	3	GC06P047	0.56
8005	NONHSAG043568.2		RNA Gene	3	GC06P047	0.56
8006	BA000025-001		RNA Gene	2	GC06P047	0.56
8007	BA000025-002		RNA Gene	2	GC06P047	0.56
8008	piR-56480-034		RNA Gene	2	GC20M003	0.56
8009	RF00017-3754		RNA Gene	2	GC22M019	0.56
8010	piR-33103-027		RNA Gene	2	GC08P018	0.56
8011	RF00017-6865		RNA Gene	2	GC08M018	0.56
8012	LOC100129	Chromosor	Pseudoger	2	GC22M019	0.56
8013	piR-45035-119		RNA Gene	2	GC20M003	0.56
8014	OCM	Oncomodu	Protein Co	32	GC07P005	0.56
8015	KLHDC1	Kelch Dom	Protein Co	34	GC14P049	0.56
8016	HSPA13	Heat Shocl	Protein Co	36	GC21M014	0.55
8017	GGTLC1	Gamma-Gl	Protein Co	31	GC20M023	0.55
8018	IGFL1	IGF Like Fa	Protein Co	27	GC19P046	0.55
8019	SERPINA12	Serpin Fan	Protein Co	38	GC14M094	0.55
8020	UBE2E3	Ubiquitin C	Protein Co	42	GC02P180	0.55
8021	GPR37	G Protein-(	Protein Co	44	GC07M124	0.54
8022	SYT16	Synaptotag	Protein Co	33	GC14P061	0.54
8023	HCG22	HLA Comp	Protein Co	20	GC06P031	0.54
8024	NMI	N-Myc An	Protein Co	38	GC02M151	0.54
8025	MIR1225	MicroRNA	RNA Gene	15	GC16M002	0.54
8026	LRRC4C	Leucine Ri	Protein Co	40	GC11M040	0.54
8027	ACBD6	Acyl-CoA E	Protein Co	35	GC01M181	0.54
8028	NLRP10	NLR Family	Protein Co	38	GC11M007	0.54
8029	GLRA3	Glycine Re	Protein Co	41	GC04M174	0.54
8030	HYAL4	Hyaluronic	Protein Co	37	GC07P123	0.53
8031	FCRLB	Fc Recepto	Protein Co	35	GC01P161	0.53
8032	TUT7	Terminal U	Protein Co	29	GC09M086	0.53
8033	HCG26	HLA Comp	RNA Gene	14	GC06P047	0.53
8034	TEMN3-AS	TENM3 An	RNA Gene	8	GC04M181	0.53
8035	CADPS	Calcium D	Protein Co	41	GC03M062	0.53
8036	KCNIP4	Potassium	Protein Co	39	GC04M020	0.53
8037	TUSC1	Tumor Sup	Protein Co	28	GC09M025	0.53
8038	SCGB3A1	Secretoglo	Protein Co	35	GC05M180	0.53
8039	ZMYM2	Zinc Finge	Protein Co	39	GC13P019	0.53

8040	HOXB1	Homeobo>	Protein Co	43	GC17M048	0.53
8041	OTUB2	OTU Deub	Protein Co	38	GC14P094	0.53
8042	SLC49A3	Solute Car	Protein Co	24	GC04M000	0.53
8043	LINC00887	Long Inter	RNA Gene	16	GC03M194	0.52
8044	CPEB1	Cytoplasm	Protein Co	40	GC15M082	0.52
8045	MIR1224	MicroRNA	RNA Gene	18	GC03P184	0.52
8046	RPRD1A	Regulation	Protein Co	36	GC18M035	0.52
8047	PEAR1	Platelet En	Protein Co	36	GC01P156	0.52
8048	ADGRE1	Adhesion (	Protein Co	34	GC19P006	0.52
8049	AGTPBP1	ATP/GTP B	Protein Co	41	GC09M085	0.52
8050	VTCN1	V-Set Dom	Protein Co	40	GC01M117	0.52
8051	ZDHHC21	Zinc Finge	Protein Co	36	GC09M014	0.52
8052	PF4V1	Platelet Fa	Protein Co	35	GC04P073	0.52
8053	MIR3175	MicroRNA	RNA Gene	14	GC15P092	0.52
8054	RABEP1	Rabaptin, I	Protein Co	39	GC17P005	0.51
8055	INTS6	Integrator	Protein Co	37	GC13M051	0.51
8056	MIR3610	MicroRNA	RNA Gene	13	GC08M116	0.51
8057	ELMO2	Engulfmen	Protein Co	40	GC20M046	0.51
8058	MPHOSPH	M-Phase P	Protein Co	37	GC02P071	0.51
8059	NPBWR1	Neuropept	Protein Co	37	GC08P052	0.51
8060	TMTC2	Transmem	Protein Co	36	GC12P082	0.51
8061	OIP5	Opa Intera	Protein Co	34	GC15M041	0.51
8062	PRM3	Protamine	Protein Co	29	GC16M011	0.51
8063	NELL1	Neural EGI	Protein Co	39	GC11P020	0.51
8064	MGC27382	Uncharacte	RNA Gene	12	GC01P078	0.5
8065	RNA5SP69	RNA, 5S Ri	Pseudoger	6	GC01P178	0.5
8066	RNY1P1	RNY1 Pseu	Pseudoger	6	GC13M027	0.5
8067	ENSG00000201412		RNA Gene	6	GC10M092	0.5
8068	ENSG00000287948		RNA Gene	4	GC04M181	0.5
8069	NBPF14	NBPF Mem	Protein Co	24	GC01M148	0.5
8070	PROZ	Protein Z,	Protein Co	39	GC13P113	0.5
8071	SRD5A2	Steroid 5 A	Protein Co	43	GC02M031	0.5
8072	ZFP42	ZFP42 Zinc	Protein Co	36	GC04P187	0.5
8073	MIR210HG	MIR210 Ho	RNA Gene	16	GC11M000	0.5
8074	FLRT2	Fibronectir	Protein Co	38	GC14P085	0.5
8075	KIR3DL3	Killer Cell I	Protein Co	36	GC19P055	0.49
8076	ZNF536	Zinc Finge	Protein Co	36	GC19P030	0.49
8077	MIR1-2	MicroRNA	RNA Gene	17	GC18M021	0.49
8078	RBMS3	RNA Bindi	Protein Co	35	GC03P028	0.49
8079	IGFL2	IGF Like Fa	Protein Co	28	GC19P046	0.49
8080	FANK1	Fibronectir	Protein Co	33	GC10P125	0.49
8081	ADAMTS12	ADAM Me	Protein Co	39	GC05M033	0.49
8082	GABRA4	Gamma-Ar	Protein Co	44	GC04M046	0.48

8083	MIR548C	MicroRNA	RNA Gene	13	GC12P064	0.48
8084	PJA2	Praja Ring	Protein Co	36	GC05M109	0.48
8085	UBFD1	Ubiquitin F	Protein Co	33	GC16P023	0.48
8086	SCGB1C1	Secretoglo	Protein Co	29	GC11P000	0.48
8087	BHMT2	Betaine--H	Protein Co	41	GC05P079	0.47
8088	FBXO15	F-Box Prot	Protein Co	37	GC18M074	0.47
8089	ZNF292	Zinc Finge	Protein Co	35	GC06P087	0.47
8090	PDZD11	PDZ Doma	Protein Co	37	GC0XM070	0.47
8091	MIR9-2	MicroRNA	RNA Gene	18	GC05M088	0.47
8092	PIAS2	Protein Inh	Protein Co	43	GC18M046	0.47
8093	SCUBE3	Signal Pep	Protein Co	37	GC06P047	0.47
8094	MXRA5	Matrix Ren	Protein Co	32	GC0XM003	0.47
8095	CCDC170	Coiled-Coi	Protein Co	32	GC06P151	0.47
8096	MIR219A2	MicroRNA	RNA Gene	15	GC09M128	0.46
8097	ENSG00000286274		RNA Gene	2	GC05P129	0.46
8098	ENSG00000228585		Pseudoger	2	GC02M005	0.46
8099	S1PR4	Sphingosir	Protein Co	42	GC19P003	0.46
8100	SCYL2	SCY1 Like	Protein Co	36	GC12P100	0.46
8101	CMYA5	Cardiomyc	Protein Co	33	GC05P079	0.46
8102	MIR613	MicroRNA	RNA Gene	13	GC12P012	0.46
8103	DERPC	DERPC Prc	Protein Co	10	GC16M069	0.46
8104	SAP130	Sin3A Assc	Protein Co	36	GC02M127	0.46
8105	DIPK2A	Divergent	Protein Co	27	GC03P143	0.46
8106	MIR135A2	MicroRNA	RNA Gene	17	GC12P097	0.46
8107	FGF5	Fibroblast	Protein Co	45	GC04P080	0.46
8108	RASGRF2	Ras Protei	Protein Co	38	GC05P080	0.46
8109	NCR3LG1	Natural Kil	Protein Co	30	GC11P017	0.46
8110	MIR208B	MicroRNA	RNA Gene	19	GC14M023	0.46
8111	RGS16	Regulator	Protein Co	40	GC01M182	0.46
8112	SERPINB7	Serpin Fan	Protein Co	40	GC18P063	0.45
8113	ATP2B2	ATPase Pla	Protein Co	45	GC03M010	0.45
8114	CYP11B2	Cytochrom	Protein Co	47	GC08M142	0.45
8115	PPP2R2B	Protein Ph	Protein Co	45	GC05M146	0.45
8116	GRID2	Glutamate	Protein Co	44	GC04P092	0.45
8117	CARD18	Caspase R	Protein Co	32	GC11M105	0.45
8118	AKAP5	A-Kinase A	Protein Co	39	GC14P064	0.44
8119	PPP1R16B	Protein Ph	Protein Co	35	GC20P038	0.44
8120	GSTM3P2	Glutathion	Pseudoger	7	GC02M159	0.44
8121	SIGLEC7	Sialic Acid	Protein Co	39	GC19P051	0.44
8122	LELP1	Late Cornil	Protein Co	31	GC01P153	0.44
8123	SIX4	SIX Homec	Protein Co	39	GC14M060	0.44
8124	SPAG5-AS	SPAG5 Ant	RNA Gene	13	GC17P028	0.44
8125	HK3	Hexokinas	Protein Co	44	GC05M176	0.43

8126	CAMK1	Calcium/C	Protein Co	43	GC03M009	0.43
8127	EPHA6	EPH Recep	Protein Co	41	GC03P096	0.43
8128	NAT8	N-Acetyltr	Protein Co	37	GC02M073	0.43
8129	PAK5	P21 (RAC1	Protein Co	36	GC20M009	0.43
8130	TTC29	Tetratricop	Protein Co	34	GC04M146	0.43
8131	PLPPR5	Phospholip	Protein Co	27	GC01M098	0.43
8132	MIR138-2	MicroRNA	RNA Gene	20	GC16P056	0.43
8133	SMILR	Smooth M	RNA Gene	9	GC08M122	0.43
8134	RPL28P3	Ribosomal	Pseudoger	6	GC05M124	0.43
8135	RNY5	RNA, Ro60	RNA Gene	12	GC07P148	0.43
8136	MIR3195	MicroRNA	RNA Gene	10	GC20P062	0.43
8137	LINC01857	Long Inter	RNA Gene	9	GC02P207	0.43
8138	RLN2	Relaxin 2	Protein Co	37	GC09M005	0.43
8139	TTTY15	Testis-Spe	RNA Gene	20	GC0YP012	0.42
8140	FABP3P2	Fatty Acid	Pseudoger	8	GC13M042	0.42
8141	DEFB118	Defensin B	Protein Co	31	GC20P031	0.42
8142	DEFB127	Defensin B	Protein Co	30	GC20P000	0.42
8143	SRPK3	SRSF Prote	Protein Co	39	GC0XP153	0.42
8144	CSMD1	CUB And S	Protein Co	37	GC08M002	0.42
8145	SIAH3	Siah E3 Ub	Protein Co	30	GC13M045	0.42
8146	AVPR1B	Arginine V	Protein Co	43	GC01M206	0.42
8147	RDH8	Retinol De	Protein Co	38	GC19P010	0.41
8148	SPINK6	Serine Pep	Protein Co	31	GC05P148	0.41
8149	GRM3	Glutamate	Protein Co	45	GC07P086	0.41
8150	SLC25A10	Solute Car	Protein Co	42	GC17P081	0.41
8151	ZNF362	Zinc Finge	Protein Co	31	GC01P033	0.41
8152	TRAPPC2	Trafficking	Protein Co	38	GC0XM013	0.41
8153	SCGB3A2	Secretoglo	Protein Co	40	GC05P147	0.41
8154	CSN3	Casein Kap	Protein Co	35	GC04P070	0.41
8155	IGSF21	Immunogl	Protein Co	36	GC01P018	0.4
8156	MIR1283-2	MicroRNA	RNA Gene	14	GC19P053	0.4
8157	MIR4295	MicroRNA	RNA Gene	12	GC10P112	0.4
8158	NDUFA4L2	NDUFA4 M	Protein Co	36	GC12M057	0.4
8159	AMOT	Angiomoti	Protein Co	40	GC0XM112	0.4
8160	MIR1185-1	MicroRNA	RNA Gene	16	GC14P104	0.4
8161	MIR1185-2	MicroRNA	RNA Gene	14	GC14P104	0.4
8162	SULT6B1	Sulfotransf	Protein Co	33	GC02M037	0.4
8163	TIPRL	TOR Signa	Protein Co	33	GC01P168	0.4
8164	PRM1	Protamine	Protein Co	31	GC16M011	0.4
8165	MIR1247	MicroRNA	RNA Gene	13	GC14M101	0.39
8166	MIR371A	MicroRNA	RNA Gene	16	GC19P053	0.39
8167	PIP4K2C	Phosphatic	Protein Co	39	GC12P057	0.39
8168	KCNMB3	Potassium	Protein Co	37	GC03M179	0.39

8169	TRIM23	Tripartite M	Protein Co	36	GC05M065	0.39
8170	SOX15	SRY-Box Ti	Protein Co	35	GC17M007	0.39
8171	GRAP	GRB2 Rela	Protein Co	41	GC17M019	0.39
8172	CCL4L2	C-C Motif	Protein Co	26	GC17P036	0.39
8173	TFAP2B	Transcripti	Protein Co	44	GC06P050	0.39
8174	MIR99AHG	Mir-99a-Le	RNA Gene	21	GC21P015	0.39
8175	MYBPC2	Myosin Bir	Protein Co	39	GC19P050	0.39
8176	RLN1	Relaxin 1	Protein Co	35	GC09M005	0.38
8177	BCL6B	BCL6B Trai	Protein Co	36	GC17P007	0.38
8178	SDS	Serine Del	Protein Co	41	GC12M113	0.37
8179	LEPROT	Leptin Rec	Protein Co	33	GC01P065	0.37
8180	LOC10537	Uncharacte	RNA Gene	4	GC02M023	0.37
8181	MIR320B2	MicroRNA	RNA Gene	14	GC01M224	0.37
8182	KY	Kyphoscoli	Protein Co	35	GC03M134	0.36
8183	MIR877	MicroRNA	RNA Gene	16	GC06P030	0.36
8184	DNAJB1P1	DnaJ Heat	Pseudoger	8	GC02M191	0.36
8185	RMST	Rhabdomy	RNA Gene	21	GC12P097	0.36
8186	ZNF626	Zinc Finge	Protein Co	30	GC19M020	0.35
8187	MIR1283-1	MicroRNA	RNA Gene	15	GC19P053	0.35
8188	MIR4463	MicroRNA	RNA Gene	12	GC06P075	0.35
8189	TMEM147	Transmem	Protein Co	34	GC19P035	0.34
8190	TNNC1	Troponin C	Protein Co	45	GC03M052	0.34
8191	MIR3188	MicroRNA	RNA Gene	13	GC19P018	0.34
8192	RIDA	Reactive In	Protein Co	31	GC08M098	0.34
8193	NCAM2	Neural Cel	Protein Co	39	GC21P020	0.34
8194	BTF3L4P2	Basic Trans	Pseudoger	8	GC02P159	0.34
8195	SLC25A21	Solute Car	Protein Co	41	GC14M036	0.33
8196	SLC25A14	Solute Car	Protein Co	40	GC0XP130	0.33
8197	GDF10	Growth Dif	Protein Co	37	GC10P047	0.33
8198	MIR133A2	MicroRNA	RNA Gene	18	GC20P062	0.33
8199	MANCR	Mitotically	RNA Gene	12	GC10M004	0.33
8200	CHST13	Carbohydr	Protein Co	36	GC03P126	0.33
8201	SRXN1	Sulfiredoxi	Protein Co	35	GC20M000	0.33
8202	MRGPRX1	MAS Relat	Protein Co	37	GC11M018	0.33
8203	H2BC3	H2B Cluste	Protein Co	27	GC06M026	0.33
8204	PXMP2	Peroxisom	Protein Co	36	GC12P132	0.32
8205	C3orf38	Chromoso	Protein Co	32	GC03P088	0.32
8206	AGAP11	ArfGAP Wi	Protein Co	27	GC10P086	0.32
8207	CLEC4G	C-Type Le	Protein Co	37	GC19M007	0.32
8208	SCML2	Scm Polyc	Protein Co	34	GC0XM018	0.32
8209	RADIL	Rap Associ	Protein Co	32	GC07M004	0.32
8210	MIR1303	MicroRNA	RNA Gene	14	GC05P154	0.32
8211	MIR4437	MicroRNA	RNA Gene	13	GC02M181	0.32

8212	LOC11059	MS1 Minis	Biological		1	GC01P031	0.32
8213	CIDEA	Cell Death	Protein Co		41	GC18P012	0.31
8214	PTCHD1-A	PTCHD1 A	RNA Gene		12	GC0XM022	0.31
8215	MIR1246	MicroRNA	RNA Gene		13	GC02M176	0.31
8216	SMPX	Small Mus	Protein Co		36	GC0XM021	0.3
8217	MIR570HG	MIR570 Ho	RNA Gene		10	GC03P195	0.3
8218	WHSC1L2P	Wolf-Hirsc	Pseudoger		8	GC17M043	0.3
8219	FOXD3-AS	FOXD3 An	RNA Gene		14	GC01M063	0.3
8220	ANKRD23	Ankyrin Re	Protein Co		31	GC02M096	0.28
8221	DCAF8L1	DDB1 And	Protein Co		27	GC0XM027	0.28
8222	TMEM182	Transmem	Protein Co		32	GC02P102	0.28
8223	CES1P1	Carboxyles	Pseudoger		22	GC16P055	0.28
8224	KIRREL2	Kirre Like I	Protein Co		40	GC19P038	0.26
8225	GPR32	G Protein-(	Protein Co		33	GC19P050	0.26
8226	RTN3P1	Reticulon 3	Pseudoger		10	GC04P145	0.26
8227	MIR568	MicroRNA	RNA Gene		10	GC03M114	0.26
8228	MIR3941	MicroRNA	RNA Gene		9	GC10P122	0.26
8229	BRCC3	BRCA1/BR(	Protein Co		43	GC0XP155	0.26
8230	CLEC9A	C-Type Le(	Protein Co		32	GC12P010	0.26
8231	PPM1K	Protein Ph	Protein Co		41	GC04M088	0.25
8232	RPS27P4	Ribosomal	Pseudoger		5	GC03P040	0.25
8233	PPP1R1A	Protein Ph	Protein Co		41	GC12M054	0.24
8234	LRRC7	Leucine Ri(	Protein Co		38	GC01P069	0.24
8235	MIR939	MicroRNA	RNA Gene		16	GC08M144	0.24
8236	FZD10-AS1	FZD10 Ant	RNA Gene		13	GC12M130	0.22
8237	CHRNA6	Cholinergi(	Protein Co		42	GC08M042	0.22
8238	CLEC12B	C-Type Le(	Protein Co		32	GC12P010	0.22
8239	GFRAL	GDNF Fam	Protein Co		29	GC06P055	0.22
8240	MIR519B	MicroRNA	RNA Gene		16	GC19P053	0.22
8241	MIR495	MicroRNA	RNA Gene		16	GC14P104	0.22
8242	GSTA6P	Glutathion	Pseudoger		7	GC06M052	0.22
8243	RGS18	Regulator	Protein Co		38	GC01P192	0.22
8244	MIR942	MicroRNA	RNA Gene		14	GC01P117	0.22
8245	LOC11059	CYP7A1 5'	Biological		1	GC08P058	0.21
8246	MAGEB18	MAGE Fam	Protein Co		32	GC0XP026	0.21
8247	MIR551B	MicroRNA	RNA Gene		16	GC03P168	0.21
8248	MUC8	Mucin 8	Protein Co		15	GC12M132	0.21
8249	POU4F2	POU Class	Protein Co		38	GC04P146	0.2
8250	CAVIN4	Caveolae A	Protein Co		26	GC09P100	0.2
8251	FAAHP1	Fatty Acid	Pseudoger		9	GC01P046	0.2
8252	MBL3P	Mannose-I	Pseudoger		8	GC10M079	0.2
8253	MIR217	MicroRNA	RNA Gene		18	GC02M055	0.19
8254	LOC10537	Uncharacte	RNA Gene		3	GC20M024	0.18

8255	SPRR1B	Small Proli	Protein Co	37	GC01P153	0.18
8256	SRMP1	SRM Pseuc	Pseudoger	7	GC20P047	0.18
8257	ADAM3A	ADAM Me	Pseudoger	14	GC08M039	0.17
8258	MIR124-2	MicroRNA	RNA Gene	18	GC08P064	0.15
8259	TMEM47	Transmem	Protein Co	35	GC0XM034	0.13
8260	LOC10272	Uncharacte	RNA Gene	8	GC12M013	0.13
8261	RPL6P25	Ribosomal	Pseudoger	6	GC12P083	0.13
8262	KIR2DS3	Killer Cell I	Protein Co	24	GC19MR0(	0.13
